# An isoxazole strategy for the synthesis of 4-oxo-1,4-dihydropyridine-3-carboxylates

**DOI:** 10.3762/bjoc.18.74

**Published:** 2022-06-23

**Authors:** Timur O Zanakhov, Ekaterina E Galenko, Mikhail S Novikov, Alexander F Khlebnikov

**Affiliations:** 1 Saint Petersburg State University, Institute of Chemistry, 7/9 Universitetskaya Naberezhnaya, St. Petersburg 199034, Russia

**Keywords:** isoxazole, methyl nicotinate, molybdenum hexacarbonyl, 4-pyridone, ring expansion

## Abstract

A method has been developed for the preparation of 2-alkyl-6-aryl-, 2-aryl-6-aryl and 2,6-diaryl-5-aryl/hetaryl-substituted methyl 4-oxo-1,4-dihydropyridine-3-carboxylates by Mo(CO)_6_-mediated ring expansion of methyl 2-(isoxazol-5-yl)-3-oxopropanoates. The high reactivity of 4-oxo-1,4-dihydropyridine-3-carboxylates synthesized provide easy access to 2,4,6-triaryl-substituted and 1,2,5,6-tetrasubstituted nicotinates.

## Introduction

**Scheme 1:**
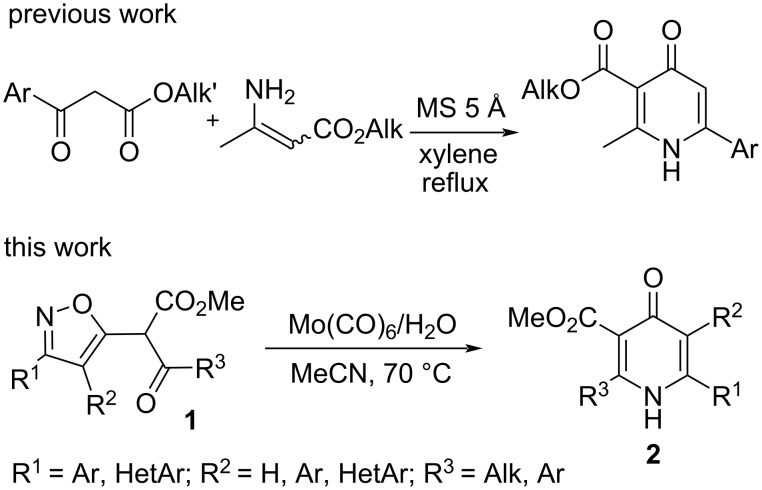
Approaches to the synthesis of alkyl 4-oxo-1,4-dihydropyridine-3-carboxylates.

Pyridine moieties are present in many natural products, drugs, pesticides and industrial materials. Pyridine fragments are used in drugs due to their specific characteristics such as basicity, hydrogen bond forming ability, water solubility, and especially because of pyridine rings are bioisosteres of amines, amides, N-heterocyclic rings and benzene rings [1–5]. A special type of pyridine, the 4-pyridones, is also fairly well represented among drugs [5–12]. Some of bioactive compounds, such as ciprofloxacin, levofloxacin, delafloxacin, and elvitegravir, contain the fragment of 4-oxo-1,4-dihydropyridine-3-carboxylic acid [6,8] and some others, ivacaftor, dolutegravir, bictegravir, aspernigrin B, and 4PYR, contain the fragment of 4-oxo-1,4-dihydropyridine-3-carboxamide [5–7]. Finding new synthetic methods for the preparation of derivatives of 4-oxo-1,4-dihydropyridine-3-carboxylic acid are therefore relevant. Some alkyl 6-aryl-2-methyl-4-oxo-1,4-dihydropyridine-3-carboxylates were prepared by refluxing a xylene solution of methyl/ethyl 3-aminobut-2-enoates and methyl/ethyl 3-aryl-3-oxopropanoates in the presence of molecular sieves, with methyl 3-aryl-3-oxopropanoates giving higher product yields (37–46%) than ethyl derivatives (13–25%) (Scheme 1) [13]. General methods for the preparation of 2,6-diaryl- and 2,5,6-triaryl-substituted derivatives of 4-oxo-1,4-dihydropyridine-3-carboxylic acid, to the best of our knowledge, have not been published to date. We would like to present here a method for the preparation of 2-alkyl-6-aryl-, 2-aryl-6-aryl/hetaryl and 2,6-diaryl-5-aryl-substituted derivatives of 4-oxo-1,4-dihydropyridine-3-carboxylic acid **2** via Mo(CO)_6_-mediated rearrangement of methyl 2-(isoxazol-5-yl)-3-oxopropanoates **1** (Scheme 1).

## Results and Discussion

**Scheme 2:**
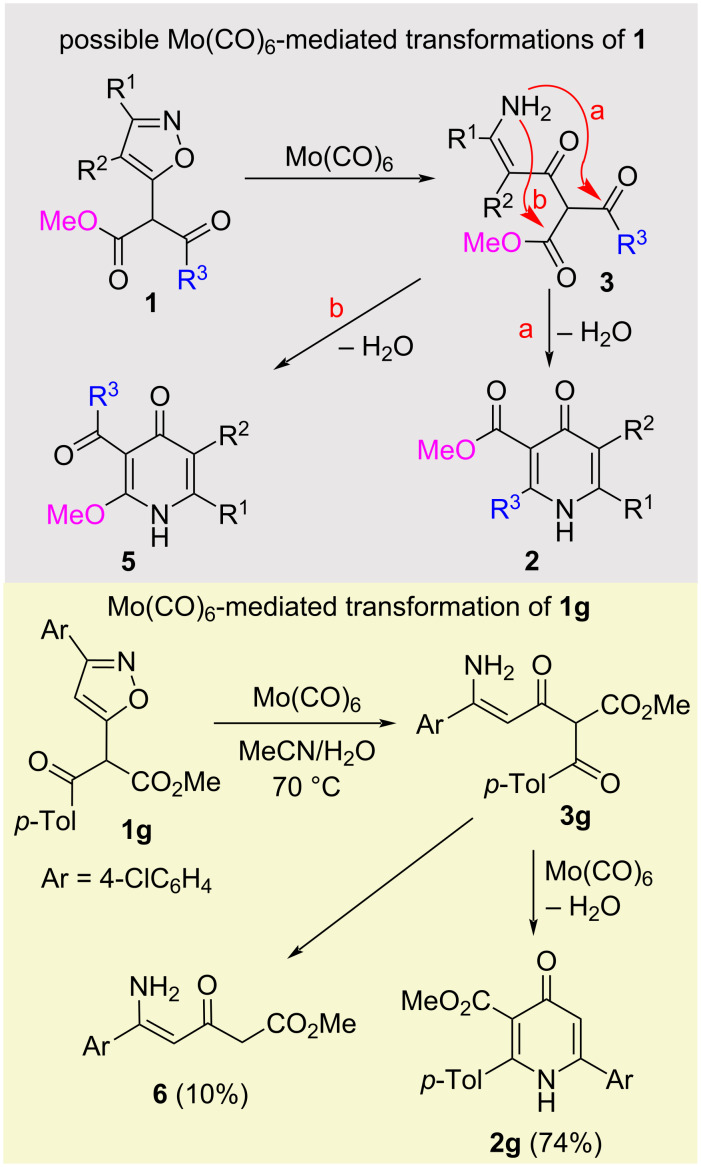
Synthesis of 4-oxo-1,4-dihydropyridine-3-carboxylates.

Based on our experience of using isoxazoles in the synthesis of heterocyclic compounds [14–18], we hypothesized that isoxazoles **1** can undergo reductive ring opening under the action of Mo(CO)_6_/H_2_O/MeCN [14,19–23] to enamines **3**, which can be cyclized on acyl R^3^C(O) group to form pyridones **2** (Scheme 2, route a). The alternative cyclization scenario, which may involve the ester group of enamine **3** and lead to the formation of pyridone **5** (route b), is less likely due to the lower electrophilicity of the ester carbon compared to the acyl carbon. To check this, isoxazole **1g** was synthesized and reacted with Mo(CO)_6_ in wet acetonitrile at 60 °C for 2 d to give pyridone **2g** in 45% yield. The reaction at 70 °C was completed in a day to give pyridone **2g** in 74% yield. A further increase in temperature, although it led to a reduction in the reaction time, was accompanied by resinification of the reaction mixture and, as a result, led to a decrease in the yields of pyridone **2g** (80 °C, 3 h, 63%; 85 °C, 3 h, 42%). Enamine **6**, resulted from the deacylation of intermediate **3g**, was isolated in the reaction carried out at 70 °C. This side process is probably the reason for the decrease in the yield of the target pyridone **2g**. Considering that isoxazoles were previously reduced to enaminones by other reducing systems [24–28], we checked the possibility of using NaBH_4_/NiSO_4_·7H_2_O [25], H_2_/PtO_2_/Ni_Raney_ [26–27] and H_2_/Ni_Raney_ [28] to obtain pyridone **2g** from isoxazole **1g**. However, analysis of reaction mixtures, obtained by using the above reductants, showed the formation of a large amount of products, but pyridone **2g** was not detected. It is most probable that, in addition to the reductive opening of isoxazole, Mo(CO)_6_ catalyzes the cyclization of enaminone **3g** to pyridine **2g** [14]. In the absence of such catalysis, the unstable enaminone intermediate undergoes deacylation and decomposes through other destructive processes.

**Scheme 3:**
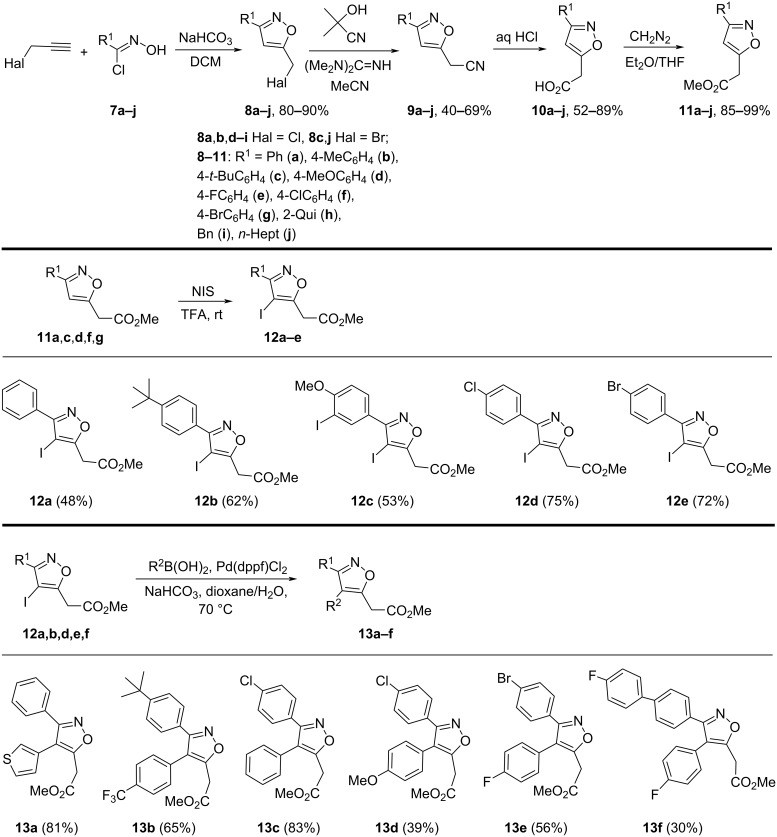
Synthesis of Isoxazoles **11**–**13**.

**Scheme 4:**
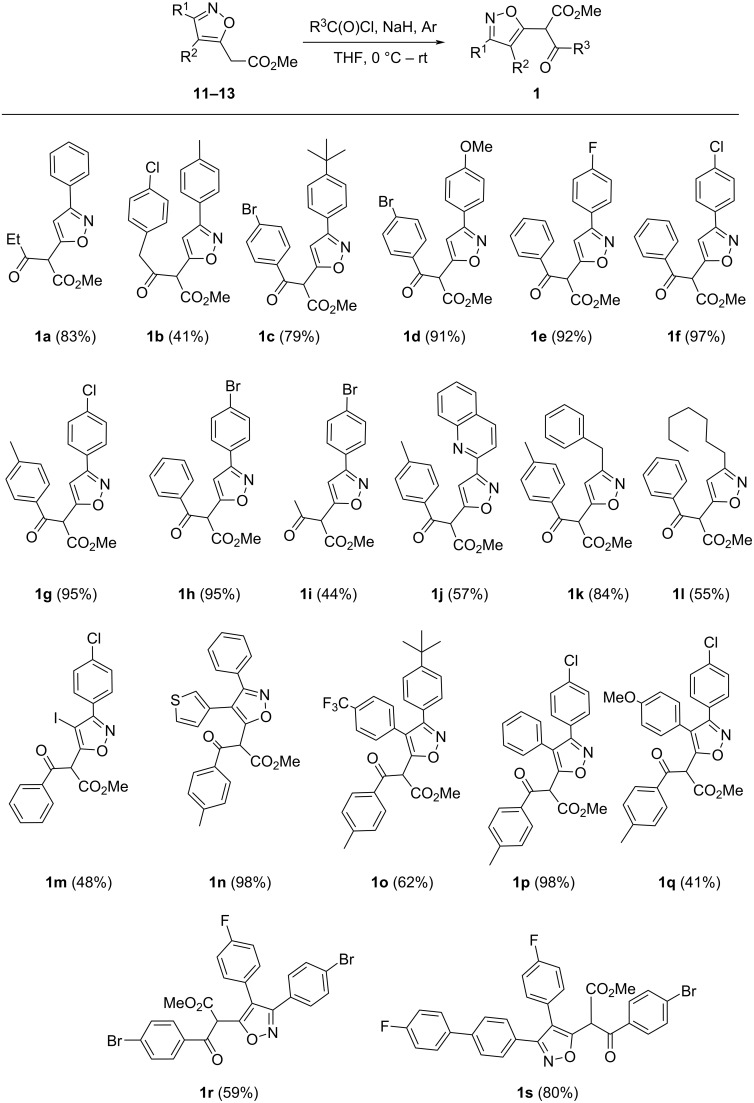
Synthesis of isoxazoles **1**.

To evaluate the scope of the disclosed reaction a set of isoxazoles **1** was prepared using approaches shown in the Scheme 3 and Scheme 4. Isoxazoles **11a**–**f** were prepared by cycloaddition of nitrile oxides, generated from *N*-hydroxyimidoyl chlorides **7a**–**f**, to propargyl halides [29], followed by the cyanation of the resulted isoxazoles **8a**–**f** to cyanides **9a**–**f** using Me_2_C(OH)CN/(Me_2_N)_2_C=NH [29], their acid hydrolysis, followed by esterification of the resulting acids **9a**–**f** with diazomethane. 4-Iodoisoxazoles **12a**–**f**, necessary for the preparation of 3,4-disubstituted isoxazoles, were obtained by iodination of **11a**,**c**,**e**–**g** with NIS/TFA [30]. The reaction of **11d** gave the product of double iodination **12c**. 4-Iodoisoxazoles **12a**,**b**,**d**–**f** were transformed into 3,4-substituted isoxazoles **13a**–**g** by Suzuki reaction using a published procedure with some modifications [31].

Isoxazoles **1**, except for isoxazole **1a**, were synthesized in 44–98% yield by acylation of isoxazoles **11**, **12** and **13** with acyl chlorides in the presence of NaH (Scheme 4). For the preparation of isoxazole **1a** propionic anhydride was used.

**Scheme 5:**
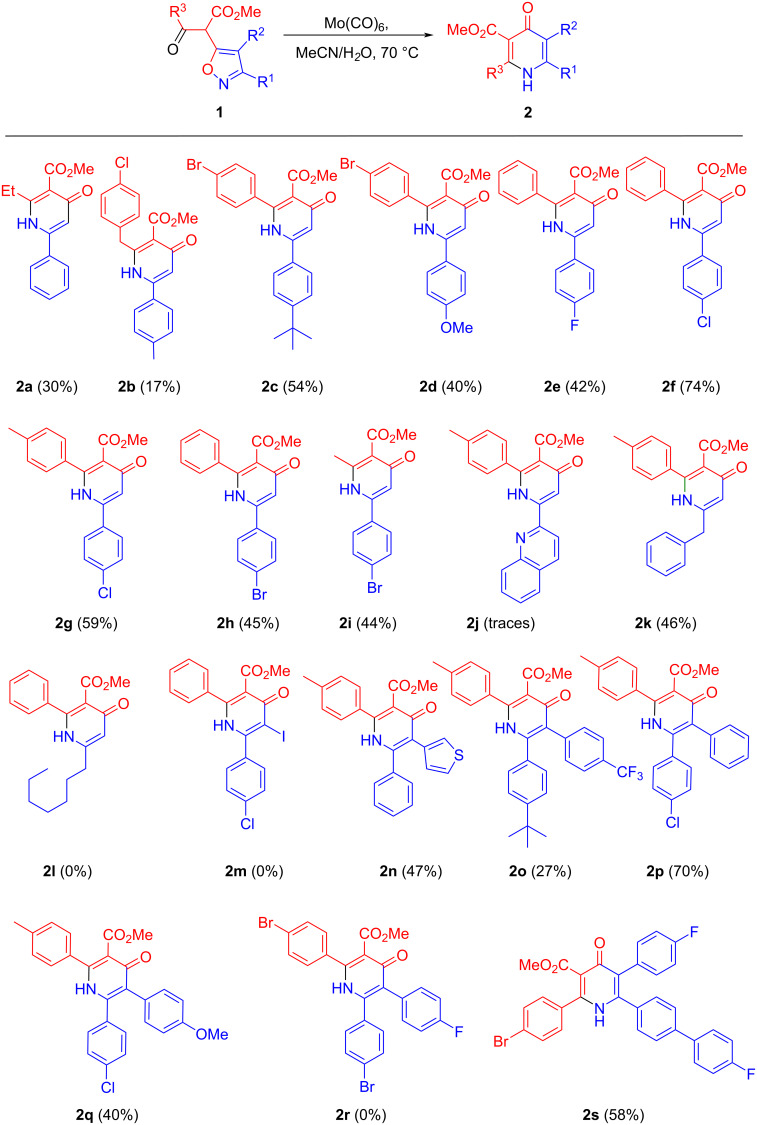
Synthesis of pyridones **2**.

Further, isoxazoles **1** were reacted with Mo(CO)_6_/H_2_O/MeCN under the optimal conditions to give pyridones **2** (Scheme 5). Isoxazole **1j** with Mo(CO)_6_/H_2_O/MeCN gives the corresponding pyridone **2j** in trace amounts, while the reaction of isoxazoles **1l**,**m**,**r** leads to complete resinification. It is likely that one of the reasons for the decrease in the yields of pyridones **2** is the occurrence of a side reaction of deacylation of enamine intermediate **3**, as shown for the reaction of isoxazole **1g** (Scheme 2).

Taking into account that compounds **2** can potentially exist in pyridone and pyridole tautomeric forms, we performed calculations at the DFT B3LYP-D3/6-311+G(d,p) level of theory with SMD solvent model to evaluate this tautomeric equilibrium. According to the calculation results compounds **2** exist in the pyridone form in acetonitrile or chloroform solution (Table 1).

**Table 1:** Gibbs free energies of the pyridole tautomer relatively to the pyridone tautomer of compounds **2** (in kcal/mol, 298 K).

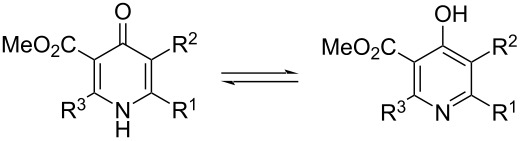			

Solvent	R^1^ = Ph, R^2^ = H, R ^3^ = Me	R^1^ = R^3^ = Ph, R^2^ = H	R^1^ = R^2^ = R^3^ = Ph

MeCN	6.1	4.4	4.7
CHCl_3_	3.5	3.1	2.6

All new compounds were characterized by ^1^H, ^13^C NMR and HRMS methods. Moreover, the structure of **2k** was also confirmed by single-crystal X-ray diffraction analysis (Figure S1, Supporting Information File 1).

**Scheme 6:**
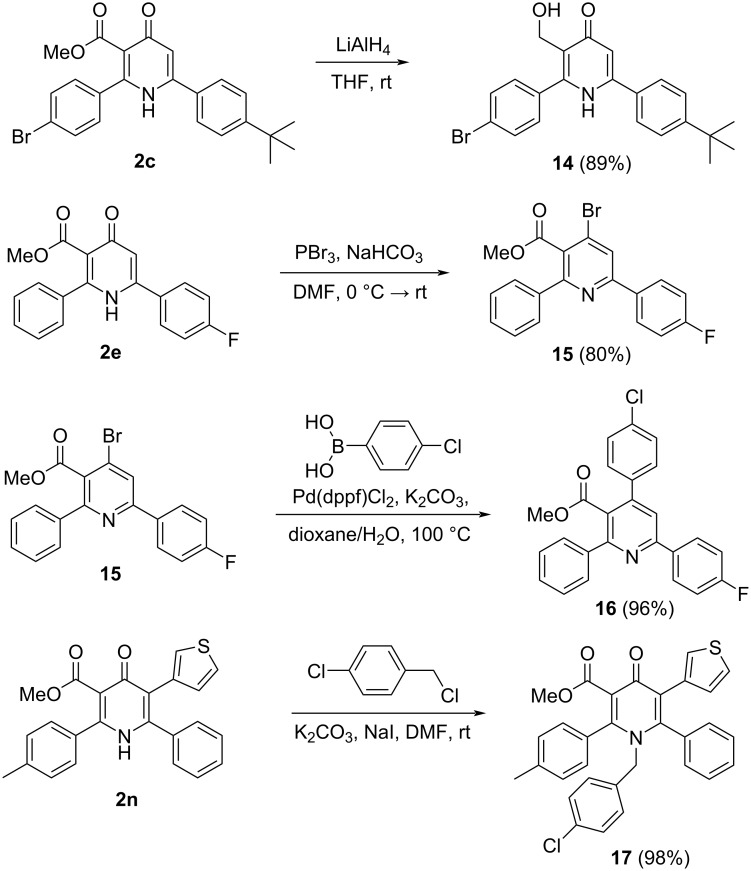
Transformations of pyridones **2**.

Compounds **2** have several reactive centers that make them attractive starting materials for the synthesis of new libraries of 4-pyridone and pyridine derivatives (Scheme 6). The methoxycarbonyl group of compound **2c** was selectively reduced to give 3-(hydroxymethyl)pyridin-4(1*H*)-one **14** in 89% yield. The reaction of pyridone **2e** with PBr_3_ afforded methyl 4-bromo-6-(4-fluorophenyl)-2-phenylnicotinate (**15**) in 80% yield. The latter was employed in the Suzuki reaction in the high-yield synthesis of 2,4,6-triaryl-substituted nicotinate **16**. The nitrogen of 2,3,4,6-tetrasubstituted pyridone **2n** can be quantitatively benzylated by benzyl chloride to give derivative **17**.

## Conclusion

A method has been developed for the preparation of 2-alkyl-6-aryl-, 2-aryl-6-aryl and 2,6-diaryl-5-aryl/hetaryl-substituted derivatives of 4-oxo-1,4-dihydropyridine-3-carboxylic acid **2** via Mo(CO)_6_-mediated rearrangement of methyl 2-(isoxazol-5-yl)-3-oxopropanoates **1**. High yield transformations of compound **2** provide easy access to 2,4,6-triaryl-substituted and 1,2,5,6-tetrasubstituted nicotinates.

## Supporting Information

File 1 Experimental procedures, compound characterization data, X-ray diffraction experiment, and copies of NMR spectra of new compounds.

File 2 Crystallographic information file for compound **2k**.
