# Supplementary material for: An isoxazole strategy for the synthesis of 4-oxo-1,4-dihydropyridine-3-carboxylates
Source: Beilstein J Org Chem. 2022 Jun 23;18:738–45. doi: 10.3762/bjoc.18.74 (PMC9235835; doi:10.3762/bjoc.18.74)

BEILSTEIN JOURNAL OF ORGANIC CHEMISTRY

Supporting Information

for

An isoxazole strategy for the synthesis of 4-oxo-1,4-dihydropyridine-3-carboxylates

Timur O. Zanakhov, Ekaterina E. Galenko, Mikhail S. Novikov and Alexander F. Khlebnikov

Beilstein J. Org. Chem. **2022,** *18,* 738–745. doi:10.3762/bjoc.18.74

Experimental procedures, compound characterization data, X-ray diffraction experiment, and copies of NMR spectra of new compounds

License and Terms: This is a supporting information file under the terms of the Creative Commons Attribution License (https://creativecommons.org/licenses/by/4.0). Please note that the reuse, redistribution and reproduction in particular requires that the author(s) and source are credited and that individual graphics may be subject to special legal provisions.
The license is subject to the *Beilstein Journal of Organic Chemistry* terms and conditions: (https://www.beilstein-journals.org/bjoc/terms)

Table of contents

Experimental	S2
X-ray diffraction experiments	S22
NMR spectra of compounds **8**	S27
NMR spectra of compounds **9**	S36
NMR spectra of compounds **10**	S45
NMR spectra of compounds **11**	S57
NMR spectra of compounds **12**	S69
NMR spectra of compounds **13**	S84
NMR spectra of compounds **1**	S102
NMR spectra of compounds **2**	S159
NMR spectra of compounds **6**	S204
NMR spectra of compounds **14**	S207
NMR spectra of compounds **15**	S210
NMR spectra of compounds **16**	S213
NMR spectra of compounds **17**	S216

Experimental

Melting points were determined on a melting point apparatus SMP30. ^{1}H (400 MHz) and ^{13}C (100 MHz) NMR spectra were recorded on a Bruker AVANCE 400 spectrometer in $CDCl_3$ or DMSO-d_6. Chemical shifts (δ) are reported in ppm downfield from tetramethylsilane. Electrospray ionization (ESI), positive mode, mass spectra were measured on a Bruker MaXis mass spectrometer, HRMS-ESI-QTOF. Single-crystal X-ray data were collected by means of a diffractometer. Crystallographic data for the structure **2k** (CCDC 2154972) have been deposited with the Cambridge Crystallographic Data Centre. Thin-layer chromatography (TLC) was conducted on aluminum sheets precoated with SiO_2 ALUGRAM SIL G/UV254. Column chromatography was performed on Macherey-Nagel silica gel 60M (0.04–0.063 mm). Physical and spectral data of 5-(halomethyl)isoxazoles **8a**,**b**,**d–f** [1], **8c** [2], **8g** [3]; 2-(isoxazol-5-yl)acetonitriles **9a**–**c**,**e–g** [2]; 2-(isoxazol-5-yl) acetic **10a**–**c**,**e–g** [2]; methyl 2-(isoxazol-5-yl)acetates **11a**–**c**,**e–g** [2] prepared according to the published procedures, were in agreement with previously reported values.

General procedure A for the preparation of 5-(halomethyl)isoxazoles 8.

Compounds **8** were prepared according to modified published procedure [4]. A mixture of propargyl chloride or propargyl bromide (3-3.5 mmol), sat. aq. $NaHCO_3$ (2 mL) and DCM (1–2 mL) was added dropwise to a solution of substituted *N*-hydroxyformimidoyl chloride **7** (1 mmol) in dichloromethane (DCM) (1–2 mL) at 0 °C. The reaction mixture was stirred for 1 d (TLC control, light petroleum/ethyl acetate 10:1 (v/v)). The organic layer was separated, the inorganic layer was extracted with DCM, the combined organic layer was dried over Na_2SO_4 and after filtration concentrated under reduced pressure. The resulted solid was crystallized from hexanes or purified by column chromatography on silica with light petroleum/ethyl acetate (8:1, v/v) as an eluent.

*5-(Chloromethyl)-3-(quinolin-2-yl)isoxazole (**8h**).* Compound **8h** was prepared following the general procedure **A** from oxime **7h** (640 mg, 3.1 mmol), propargyl chloride (1.4 g, 19 mmol) and $NaHCO_3$ (16 mL) in DCM (30 mL) in 674 mg (89% yield) as a dark brown solid: mp 102-105 °C (dichloromethane). ^{1}H NMR ($CDCl_3$, 400 MHz): δ 4.72 (s, 2H), 7.20 (s, 1H), 7.58-7.62 (m, 1H), 7.64-7.78 (m, 1H), 7.85-7.88 (m, 1H), 8.14-8.19 (m, 2H), 8.25-8.27 (m, 1H). ^{13}C NMR ($CDCl_3$, 101 MHz): δ 34.4 (CH_2), 103.1 (CH), 119.0 (CH), 127.4 (CH), 127.7 (CH), 128.4 (C), 129.7 (CH), 130.0 (CH), 137.0 (CH), 148.0 (C), 148.1 (C), 163.9 (C), 168.1 (C). HRMS (ESI) m/z: 267.0296 calcd for $C_{13}H_9ClN_2ONa^+$ $[M + Na]^+$; found 267.0292.

*3-Benzyl-5-(chloromethyl)isoxazole (**8i**).* Compound **8i** was prepared following the general procedure **A** from oxime **7i** (5.00 g, 30 mmol), propargyl chloride (13.5 g, 178 mmol) and $NaHCO_3$ (147 mL) in DCM (200 mL) in 5.00 g (82% yield) after column chromatography on silica (light petroleum/ethyl acetate, 8:1, v/v) as a light brown oil. ^{1}H NMR ($CDCl_3$, 400 MHz): δ 4.01 (s, 2H), 4.54 (s, 2H), 6.08 (s, 1H), 7.24-7.28 (m, 3H), 7.30-7.35 (m, 2H). ^{13}C NMR ($CDCl_3$, 101 MHz): δ 32.4 (CH_2), 34.4 (CH_2), 103.6 (CH), 127.0

(CH), 128.8 (CH), 128.8 (CH), 136.8 (C), 163.3 (C), 167.4 (C). HRMS (ESI) m/z: 230.0343 calcd for $C_{11}H_{10}ClNNaO^+$ [M + Na]$^+$; found 230.0343.

*5-(Bromomethyl)-3-heptylisoxazole (**8j**).* Compound **8j** was prepared following the general procedure **A** from oxime **7i** (2.0 mg, 11.0 mmol), propargyl bromide (5 mL, 45 mmol) and $NaHCO_3$ (55 mL) in DCM (60 mL) in 2.25 g (79% yield) after column chromatography on silica (light petroleum/ethyl acetate, 8:1, v/v) as a light yellow oil. ^{1}H NMR ($CDCl_3$, 400 MHz): δ 0.86-0.89 (m, 3H), 1.24-1.37 (m, 8H), 1.65 (p, J = 7.4 Hz, 2H), 2.64 (t, J = 7.4 Hz, 2H), 4.43 (s, 2H), 6.16 (s, 1H). ^{13}C NMR ($CDCl_3$, 101 MHz): δ 14.0 (CH_3), 18.8 (CH_2), 22.6 (CH_2), 26.0 (CH_2), 28.2 (CH_2), 28.9 (CH_2), 29.1 (CH_2), 31.7 (CH_2), 103.4 (CH), 164.5 (C), 166 (C). HRMS (ESI) m/z: 260.0645 calcd for $C_{11}H_{19}BrNO^+$ [M + H]$^+$; found 260.0652.

General procedure B for the preparation of 2-(isoxazol-5-yl)acetonitriles 9.

Nitriles **9** were prepared according to modified published procedure [5]. 5-(Halomethyl)isoxazole **8** (1 mmol) and acetone cyanohydrin (4 mmol) were dissolved in dried acetonitrile (10 mL per 1 mmol of **8**). A solution of tetramethylguanidine (1.5 mmol) in acetonitrile was added dropwise to the reaction mixture at room temperature and stirred for 1 h (for bromomethyl substituted isoxazoles) or at 50 °C (oil bath temperature) for 24 h (for chloromethyl substituted isoxazoles) (TLC control, light petroleum/ethyl acetate = 8:1, v/v). 2-(Isoxazol-5-yl)acetonitriles **9** were purified by column chromatography on silica with light petroleum/ethyl acetate (5:1, v/v) as an eluent.

*2-(3-(4-Methoxyphenyl)isoxazol-5-yl)acetonitrile (**9d**).* Compound **9d** was prepared following the general procedure **B** from isoxazole **8d** (1.0 g, 4.5 mmol), acetone cyanohydrin (1.53 g, 18 mmol), tetramethylguanidine (782 mg, 6.8 mmol) in MeCN (45 mL) in 660 mg (69% yield) after column chromatography on silica (light petroleum/ethyl acetate, 8:1, v/v) as a colorless solid: mp 97-99 °C (light petroleum/ethyl acetate). ^{1}H NMR ($CDCl_3$, 400 MHz): δ 3.86 (s, 3H), 3.93 (s, 2H), 6.62 (s, 1H), 6.98 (d, J = 8.9 Hz, 2H), 7.73 (d, J = 8.8 Hz, 2H). ^{13}C NMR ($CDCl_3$, 101 MHz): δ 16.7 (CH_2), 55.4 (CH_3), 101.6 (CH), 114.0 (C), 114.5 (CH), 120.6 (C), 128.3 (CH), 161.1 (C), 161.4 (C), 162.6 (C). HRMS (ESI) m/z: 237.0634 calcd for $C_{12}H_{10}N_2O_2Na^+$ [M + Na]$^+$; found 237.0634.

*2-(3-(Quinolin-2-yl)isoxazol-5-yl)acetonitrile (**9h**).* Compound **9h** was prepared following the general procedure **B** from 5-(chloromethyl)isoxazole **8h** (655 mg, 2.7 mmol), acetone cyanohydrin (918 mg, 11 mmol), tetramethylguanidine (470 mg, 4.1 mmol) in MeCN (27 mL) in 254 mg (40% yield) after column chromatography on silica (light petroleum/ethyl acetate, 8:1, v/v) as a colorless solid: mp 118-120 °C (light petroleum/ethyl acetate). ^{1}H NMR ($CDCl_3$, 400 MHz): δ 4.01 (s, 2H), 7.25 (s, 1H), 7.59-7.63 (m, 1H), 7.75-7.79 (m, 1H), 7.86-7.88 (m, 1H), 8.14-8.17 (m, 2H), 8.26-8.28 (m, 1H). ^{13}C NMR ($CDCl_3$, 101 MHz): δ 16.7 (CH_2), 103.2 (CH), 113.8 (C), 118.9 (CH), 127.6 (CH), 127.7 (CH), 128.5 (C), 129.8 (CH), 130.1 (CH), 137.1 (CH), 147.7 (C), 148.0 (C), 161.4 (C), 164.2 (C). HRMS (ESI) m/z: 258.0638 calcd for $C_{14}H_9N_3ONa^+$ [M + Na]$^+$; found 258.0640.

*2-(3-Benzylisoxazol-5-yl)acetonitrile (**9i**).* Compound **9i** was prepared following the general procedure **B** from 5-(chloromethyl)isoxazole **8i** (1.5 g, 7.3 mmol), acetone cyanohydrin (2.48 g, 29.2 mmol), tetramethylguanidine (1.26 g, 1.1 mmol) in MeCN (70 mL) in 778 mg (54% yield) after column

chromatography on silica (light petroleum/ethyl acetate, 7:1, v/v) as an yellow oil. ^{1}H NMR ($CDCl_3$, 400 MHz): δ 3.83 (s, 2H), 4.01 (s, 2H), 6.12 (s, 1H), 7.23-7.29 (m, 3H), 7.31-7.36 (m, 2H). ^{13}C NMR ($CDCl_3$, 101 MHz): δ 16.6 (CH_2), 32.3 (CH_2), 103.6 (CH), 113.9 (C), 127.1 (CH), 128.8 (CH), 128.9 (CH), 136.5 (C), 160.9 (C), 163.7 (C). HRMS (ESI) m/z: 221.0685 calcd for $C_{12}H_{10}N_2ONa^+$ [M + Na]$^+$; found 221.0689.

General procedure C for the preparation of 2-(isoxazol-5-yl)acetic acids 10.

The compounds **10** were prepared according to modified published procedure [6]. Conc. aq. HCl (4 mL) was added to a solution of 2-(isoxazol-5-yl)acetonitrile **9** (1 mmol) in 1,4-dioxane (4 mL). The mixture was heated at 105 °C (oil bath tempetarure) for 1.5 h (TLC control, light petroleum/ethyl acetate = 8:1, (v/v)). The reaction mixture was cooled to room temperature and diluted with water. The solid precipitated was filtered off, washed with water and dried on air.

*2-(3-(4-Methoxyphenyl)isoxazol-5-yl)acetic acid (**10d**).* Compound **10d** was prepared following the general procedure **C** from acetonitrile **9d** (640 mg, 3.0 mmol), hydrochloric acid (4 mL) in 1,4-dioxane (4 mL) in 553 mg (79% yield) as a colorless solid: mp 166-168 °C (water/1,4-dioxane). ^{1}H NMR (DMSO-d_6, 400 MHz): δ 3.82 (s, 3H), 3.94 (s, 2H), 6.86 (s, 1H), 7.05-7.07 (m, 2H), 7.79-7.81 (m, 2H), 12.89 (br. s, 1H). ^{13}C NMR (DMSO-d_6, 101 MHz): δ 32.4 (CH_2), 55.3 (CH_3), 101.3 (CH), 114.5 (CH), 121.0 (C), 128.0 (CH), 160.7 (C), 161.5 (C), 166.9 (C), 169.2 (C). HRMS (ESI) m/z: 256.0580 calcd for $C_{12}H_{11}NO_4Na^+$ [M + Na]$^+$; found 256.0580.

*2-(3-(Quinolin-2-yl)isoxazol-5-yl)acetic acid (**10h**).* Compound **10h** was prepared following the general procedure **C** from acetonitrile **9h** (254 mg, 1.1 mmol), hydrochloric acid (4 mL) in 1,4-dioxane (4 mL) in 200 mg (72% yield) as a colorless solid: mp 178-180 °C (water/1,4-dioxane). ^{1}H NMR (DMSO-d_6, 400 MHz): δ 4.06 (s, 2H), 7.10 (s, 1H), 7.68-7.72 (m, 1H), 7.83-7.88 (m, 1H), 8.06-8.16 (m, 3H), 8.53-8.55 (m, 1H), 12.94 (br. s, 1H). ^{13}C NMR (DMSO-d_6, 101 MHz): δ 32.3 (CH_2), 102.3 (CH), 118.7 (CH), 127.6 (CH), 128.0 (C), 128.1 (CH), 129.1 (CH), 130.4 (CH), 137.6 (CH), 147.4 (C), 148.0 (C), 163.1 (C), 167.9 (C), 169.2 (C). HRMS (ESI) m/z: 277.0584 calcd for $C_{14}H_{10}N_2O_3Na^+$ [M + Na]$^+$; found 277.0581.

*2-(3-Benzylisoxazol-5-yl)acetic acid (**10i**).* Compound **10i** was prepared following the general procedure **C** from acetonitrile **9i** (550 mg, 2.78 mmol), hydrochloric acid (4 mL) in 1,4-dioxane (4 mL) in 314 mg (52% yield) as a colorless solid: mp 111-113 °C (water/1,4-dioxane). ^{1}H NMR (DMSO-d_6, 400 MHz): δ 3.84 (s, 2H), 3.97 (s, 2H), 6.21 (s, 1H), 7.23-7.35 (m, 5H), 12.80 (br. s, 1H). ^{13}C NMR (DMSO-d_6, 101 MHz): δ 31.4 (CH_2), 32.2 (CH_2), 103.4 (CH), 126.6 (CH), 128.6 (CH), 128.7 (CH), 137.6 (C), 162.8 (C), 166.3 (C), 169.2 (C). HRMS (ESI) m/z: 240.0631 calcd for $C_{12}H_{11}NO_3Na^+$ [M + Na]$^+$; found 240.0634.

*2-(3-Heptylisoxazol-5-yl)acetic acid (**10j**).* Compound **10j** was prepared following the general procedures **B and C** without purification of intermediate acetonitrile. Isoxazole **8j** (2.0 g, 7.6 mmol) and acetone cyanohydrin (2.58 g, 30.4 mmol) was dissolved in dried acetonitrile (75 mL). The solution of tetramethylguanidine (1.31 g, 11.4 mmol) in acetonitrile (5 mL) was added dropwise to the reaction mixture at room temperature and stirred for 24 h (TLC control, light petroleum/ethyl acetate = 8:1, v/v).

The reaction mixture was evaporated and the residue was dissolved in 1,4-dioxane (10 mL), conc. aq. HCl (10 mL) was added, and the mixture was heated at 105 °C (oil bath temperature) for 1.5 h (TLC control, light petroleum/ethyl acetate = 8:1, (v/v)). The reaction mixture was cooled to room temperature and diluted with water, alkalized to pH 10–12 with aq. NaOH and washed with diethyl ether twice. The inorganic layer was acidified with conc. aq. HCl to pH 2 and the solid precipitated filtered off, washed with water and dried on air to give pure compound **10j** in 1.05 g (61% on to 2 steps) as a light yellow solid: mp 65-67 °C (water). ^{1}H NMR (DMSO-d_6, 400 MHz): δ 0.85-0.88 (m, 3H), 1.25-1.31 (m, 8H), 1.59 (p, J = 7.0 Hz, 2H), 2.58 (t, J = 7.6 Hz, 2H), 3.84 (s, 2H), 6.28 (s, 1H), 12.74 (s, 1H). ^{13}C NMR (DMSO-d_6, 101 MHz): δ 13.9 (CH_3), 22.0 (CH_2), 25.2 (CH_2), 27.6 (CH_2), 28.3 (CH_2), 28.5 (CH_2), 31.1 (CH_2), 32.3 (CH_2), 103.0 (CH), 163.6 (C), 165.9 (C), 169.3 (C). HRMS (ESI) m/z: 226.1438 calcd for $C_{12}H_{20}NO_3^+$ [M + H]$^+$; found 226.1437.

General procedure D for the preparation of methyl 2-(isoxazol-5-yl)acetates 11.

Methyl 2-(isoxazol-5-yl)acetates **11** were prepared according to modified published procedure [7]. A suspension or a solution of 2-(isoxazol-5-yl)acetic acid **10** (1 mmol) in THF (5 mL) was slowly added under stirring and ice-cooling to a solution of diazomethane, prepared from *N*-nitroso-*N*-methylurea (NMU) (3 mmol), 40% aq. KOH (15 mmol) and ether (10 mL). The reaction mixture was stirred for 1 h at room temperature (TLC control, light petroleum/ethyl acetate, 8:1, (v/v)), the excess of diazomethane was quenched with acetic acid and the solvents were removed in vacuo.

*Methyl 2-(3-(4-methoxyphenyl)isoxazol-5-yl)acetate (**11d**).* Compound **11d** was prepared following the general procedure **D** from acid **10d** (553 mg, 2.37 mmol) and NMU (733 mg, 7.12 mmol) in THF (10 mL) and ether (20 mL) in 523 mg (89% yield) as a light yellow oil. ^{1}H NMR ($CDCl_3$, 400 MHz): δ 3.78 (s, 3H), 3.85 (s, 3H), 3.87 (s, 2H), 6.54 (s, 1H), 6.96-6.98 (m, 2H), 7.72-7.76 (m, 2H). ^{13}C NMR ($CDCl_3$, 101 MHz): δ 32.6 (CH_2), 52.6 (CH_3), 55.3 (CH_3), 101.3 (CH), 113.3 (CH), 121.5 (C), 128.2 (CH), 161.0 (C), 162.3 (C), 165.2 (C), 168.1 (C). HRMS (ESI) m/z: 270.0737 calcd for $C_{13}H_{13}NO_4Na^+$ [M + Na]$^+$; found 270.0740.

*Methyl 2-(3-(quinolin-2-yl)isoxazol-5-yl)acetate (**11h**).* Compound **11h** was prepared following the general procedure **D** from acid **10h** (200 mg, 0.79 mmol) and NMU (243 mg, 2.36 mmol) in THF (5 mL) and ether (10 mL) in 180 mg (85% yield) as a colorless solid: mp 80-82 °C (diethyl ether/THF). ^{1}H NMR ($CDCl_3$, 400 MHz): δ 3.80 (s, 3H), 3.95 (s, 2H), 7.15 (s, 1H), 7.58-7.61 (m, 1H), 7.74-7.78 (m, 1H), 7.85-7.88 (m, 1H), 8.16-8.19 (m, 2H), 8.25-8.27 (m, 1H). ^{13}C NMR ($CDCl_3$, 101 MHz): δ 32.8 (CH_2), 52.7 (CH_3), 102.7 (CH), 119.1 (CH), 127.3 (CH), 127.7 (CH), 129.8 (CH), 129.9 (CH), 136.9 (CH), 148.0 (C), 148.5 (C), 163.9 (C), 165.7 (C), 167.9 (C). HRMS (ESI) m/z: 291.0740 calcd for $C_{15}H_{12}N_2O_3Na^+$ [M + Na]$^+$; found 291.0738.

*Methyl 2-(3-benzylisoxazol-5-yl)acetate (**11i**).* Compound **11i** was prepared following the general procedure **D** from acid **10i** (300 mg, 1.38 mmol) and NMU (427 mg, 4.15 mmol) in THF (10 mL) and ether (20 mL) in 374 mg (99% yield) as an yellow oil. ^{1}H NMR ($CDCl_3$, 400 MHz): δ 3.73 (s, 3H), 3.77 (s, 3H), 4.00 (s, 2H), 6.04 (s, 1H), 7.23-7.26 (m, 3H), 7.30-3.33 (m, 2H). ^{13}C NMR ($CDCl_3$, 101 MHz): δ

32.4 (CH_2), 32.6 (CH_2), 52.5 (CH_3), 103.3 (CH), 126.9 (CH), 128.7 (CH), 128.8 (CH), 137.1 (C), 163.2 (C), 165.0 (C), 168.0 (C). HRMS (ESI) m/z: 254.0788 calcd for $C_{13}H_{13}NO_3Na^+$ $[M + Na]^+$; found 254.0795.

*Methyl 2-(3-heptylisoxazol-5-yl)acetate (**11j**).* Compound **11j** was prepared following the general procedure **D** from acid **10j** (1.05 g, 4.7 mmol) and NMU (1.44 g, 14.0 mmol) in THF (15 mL) and ether (40 mL) in 1.14 g (93% yield) as a light yellow oil. ^{1}H NMR ($CDCl_3$, 400 MHz): δ 0.86-0.89 (m, 3H), 1.24-1.37 (m, 8H), 1.65 (p, J = 7.5 Hz, 2H), 2.64 (t, J = 7.7 Hz, 2H), 3.76 (s, 3H), 3.79 (s, 2H), 6.11 (s, 1H). ^{13}C NMR ($CDCl_3$, 101 MHz): δ 14.0 (CH_3), 22.6 (CH_2), 26.0 (CH_2), 28.2 (CH_2), 28.9 (CH_2), 29.2 (CH_2), 31.7 (CH_2), 32.6 (CH_2), 52.5 (CH_3), 103.0 (CH), 164.4 (C), 164.5 (C), 168.2 (C). HRMS (ESI) m/z: 240.1594 calcd for $C_{13}H_{22}NO_3^+$ $[M + H]^+$; found 240.1597.

General procedure E for the preparation of methyl 2-(4-iodoisoxazol-5-yl)acetates 12.

To a solution of methyl 2-(isoxazol-5-yl)acetates **11** (1 mmol) in TFA (5 ml per 1 mmol of **11**) N-iodosuccinimide (1.5 mmol) was added and the reaction mixture stirred at rt for 24 h, with water, basified with sat. aq. $NaHCO_3$, neutralized with sat. aq. NaS_2O_3 and extracted with ethyl acetate. The solvent was evaporated, and the residue was purified by column chromatography on silica with light petroleum/ethyl acetate (20:1, v/v) as an eluent.

*Methyl 2-(4-iodo-3-phenylisoxazol-5-yl)acetate (**12a**).* Compound **12a** was prepared following the general procedure **E** from methyl acetate **11a** (1000 mg, 4.6 mmol) and *N*-iodosuccinimide (1552 mg, 6.9 mmol) in TFA (23 mL) in 754 mg (48% yield) after column chromatography on silica (light petroleum/ethyl acetate, 20:1, v/v) as a light yellow solid: mp 48-50 °C (light petroleum/ethyl acetate). ^{1}H NMR ($CDCl_3$, 400 MHz): δ 3.79 (s, 3H), 3.97 (s, 2H), 7.48-7.51 (m, 3H), 7.79-7.81 (m, 2H). ^{13}C NMR ($CDCl_3$, 101 MHz): δ 33.0 (CH_2), 52.8 (CH_3), 60.4 (C), 128.5 (CH), 128.6 (CH), 130.1 (CH), 137.8 (C), 163.0 (C), 167.0 (C), 167.2 (C). HRMS (ESI) m/z: 365.9598 calcd for $C_{12}H_{10}INO_3Na^+$ $[M + Na]^+$; found 365.9604.

*Methyl 2-(3-(4-(tert-butyl)phenyl)-4-iodoisoxazol-5-yl)acetate (**12b**).* Compound **12b** was prepared following the general procedure **E** from methyl acetate **11c** (355 mg, 1.3 mmol) and *N*-iodosuccinimide (439 mg, 1.95 mmol) in TFA (7 mL) in 321 mg (62% yield) after column chromatography on silica (light petroleum/ethyl acetate, 20:1, v/v) as a light yellow oil. ^{1}H NMR ($CDCl_3$, 400 MHz): δ 1.36 (s, 9H), 3.78 (s, 3H), 3.96 (s, 2H), 7.50-7.52 (m, 2H), 7.75-7.77 (m, 2H). ^{13}C NMR ($CDCl_3$, 101 MHz): δ 31.2 (CH_3), 33.1 (CH_2), 34.9 (CH_3), 52.8 (C), 60.3 (C), 125.4 (CH), 125.6 (C), 128.1 (CH), 153.4 (C), 162.8 (C), 167.0 (C), 167.1 (C). HRMS (ESI) m/z: 400.0404 calcd for $C_{16}H_{19}INO_3^+$ $[M + H]^+$; found 400.0410.

*Methyl 2-(4-iodo-3-(4-methoxyphenyl)isoxazol-5-yl)acetate (**12c**).* Compound **12c** was prepared following the general procedure **E** from methyl acetate **11d** (1045 mg, 4.2 mmol) and *N*-iodosuccinimide (1.426 g, 6.3 mmol) in TFA (25 mL) in 844 mg (53% yield) after column chromatography on silica (light petroleum/ethyl acetate, 20:1, v/v) as a light yellow solid: mp 123-125 °C (light petroleum/ethyl acetate). ^{1}H NMR ($CDCl_3$, 400 MHz): δ 3.78 (s, 3H), 3.95 (s, 5H), 6.91-6.93 (m, 1H), 7.79-7.81 (m, 1H), 8.24-8.25 (m, 1H). ^{13}C NMR ($CDCl_3$, 101 MHz): δ 33.0 (CH_2), 52.8 (CH_3), 56.5 (CH_3), 60.2 (C), 85.9 (C),

110.5 (CH), 122.5 (C), 129.9 (CH), 139.4 (CH), 159.6 (C), 161.3 (C), 167.0 (C), 167.3 (C). HRMS (ESI) m/z: 521.8670 calcd for $C_{13}H_{11}I_2NO_4Na^+$ [M + Na]$^+$; found 521.8664.

*Methyl 2-(3-(4-chlorophenyl)-4-iodoisoxazol-5-yl)acetate (**12d**).* Compound **12d** was prepared following the general procedure **E** from methyl acetate **11f** (753 mg, 3 mmol) and *N*-iodosuccinimide (1.012 g, 4.5 mmol) in TFA (15 mL) in 848 mg (75% yield) after column chromatography on silica (light petroleum/ethyl acetate, 20:1, v/v) as a light yellow solid: mp 54-56 °C (light petroleum/ethyl acetate). ^{1}H NMR ($CDCl_3$, 400 MHz): δ 3.79 (s, 3H), 3.96 (s, 2H), 7.46-7.49 (m, 2H), 7.74-7.78 (m, 2H). ^{13}C NMR ($CDCl_3$, 101 MHz): δ 33.0 (CH_2), 52.8 (CH_3), 60.1 (C), 126.8 (C), 128.9 (CH), 129.8 (CH), 136.4 (C), 162.1 (C), 166.9 (C), 167.5 (C). HRMS (ESI) m/z: 399.9208 calcd for $C_{12}H_9ClINO_3Na^+$ [M + Na]$^+$; found 399.9209.

*Methyl 2-(3-(4-bromophenyl)-4-iodoisoxazol-5-yl)acetate (**12e**).* Compound **12e** was prepared following the general procedure **E** from methyl acetate **11g** (840 mg, 2.8 mmol) and *N*-iodosuccinimide (957 mg, 4.3 mmol) in TFA (15 mL) in 862 mg (72% yield) after column chromatography on silica (light petroleum/ethyl acetate, 20:1, v/v) as a light yellow solid: mp 83-85 °C (light petroleum/ethyl acetate). ^{1}H NMR ($CDCl_3$, 400 MHz): δ 3.79 (s, 3H), 3.96 (s, 2H), 7.62-7.65 (m, 2H), 7.68-7.72 (m, 2H). ^{13}C NMR ($CDCl_3$, 101 MHz): δ 33.0 (CH_2), 52.8 (CH_3), 60.1 (C), 124.8 (C), 127.3 (C), 130.0 (CH), 131.9 (CH), 162.2 (C), 166.9 (C), 167.5 (C). HRMS (ESI) m/z: 443.8703 calcd for $C_{12}H_9BrINO_3Na^+$ [M + Na]$^+$; found 443.8708.

General procedure F for the preparation of methyl 2-(3-aryl-4-arylisoxazol-5-yl)acetates 13.

To a solution of methyl 2-(4-iodo-3-(aryl)isoxazol-5-yl)acetate **12** (1 mmol) and boronic acid (1.5 mmol) in dioxane/water (3:1, v/v) (3 mmol) and Pd(dppf)Cl_2 (0.05 mmol) was added $NaHCO_3$ under argon atmosphere and the mixture was stirred at 70 °C (oil bath temperature) for 24 h (TLC control, light petroleum/ethyl acetate, 8:1, v/v). The reaction mixture was cooled to room temperature, diluted with water and extracted with ethyl acetate. The solvent was evaporated, and the residue was purified by column chromatography on silica with light petroleum/ethyl acetate (20:1, v/v) as an eluent.

*Methyl 2-(3-phenyl-4-(thiophen-3-yl)isoxazol-5-yl)acetate (**13a**).* Compound **13a** was prepared following the general procedure **F** from methyl 2-(4-iodo-3-(aryl)isoxazol-5-yl)acetate **12a** (343 mg, 1.0 mmol) and 3-thiophenylboronic acid (256 mg, 2.0 mmol), $NaHCO_3$ (252 mg, 3.0 mmol) and Pd(dppf)Cl_2 (37 mg, 0.05 mmol) in dioxane (6 mL) and water (2 mL) in 242 mg (81% yield) after column chromatography on silica (light petroleum/ethyl acetate, 20:1, v/v) as a colorless solid: mp 71-72 °C (light petroleum/ethyl acetate). ^{1}H NMR ($CDCl_3$, 400 MHz): δ 3.76 (s, 3H), 3.84 (s, 2H), 6.87-6.89 (m, 1H), 7.26-7.27 (m, 1H), 7.33-7.37 (m, 3H), 7.38-7.42 (m, 1H), 7.47-7.50 (m, 2H). ^{13}C NMR ($CDCl_3$, 101 MHz): δ 32.0 (CH_2), 52.7 (CH_3), 113.4 (C), 124.6 (CH), 126.4 (CH), 128.3 (CH), 128.4 (CH), 128.5 (CH), 128.7 (C), 129.2 (C), 129.6 (CH), 161.3 (C), 162.1 (C), 168.1 (C). HRMS (ESI) m/z: 300.0689 calcd for $C_{16}H_{14}NO_3S^+$ [M + H]$^+$; found 300.0693.

*Methyl 2-(3-(4-(tert-butyl)phenyl)-4-(4-(trifluoromethyl)phenyl)isoxazol-5-yl)acetate (**13b**).* Compound **13b** was prepared following the general procedure **F** from methyl 2-(4-iodo-3-(aryl)isoxazol-5-yl)acetate

12b (300 mg, 0.75 mmol), (4-(trifluoromethyl)phenyl)boronic acid (214 mg, 1.13 mmol), $NaHCO_3$ (189 mg, 2.25 mmol) and Pd(dppf)Cl_2 (27 mg, 0.038 mmol) in dioxane (7 mL) and water (3 mL) in 205 mg (65% yield) after column chromatography on silica (light petroleum/ethyl acetate, 30:1, v/v) as a colorless oil. ^{1}H NMR ($CDCl_3$, 400 MHz): δ 1.31 (s, 9H), 3.75 (s, 3H), 3.80 (s, 2H), 7.35 (s, 4H), 7.37-7.39 (m, 2H), 7.65-7.67 (m, 2H). ^{13}C NMR ($CDCl_3$, 101 MHz): δ 31.2 (CH_3), 31.9 (CH_2), 34.8 (C), 52.8 (CH_3), 116.9 (C), 122.6 (C), 125.2 (C), 125.7 (CH), 125.8 (q, CH, J = 3.7 Hz), 128.0 (CH), 130.2 (CH), 130.5 (C), 133.6 (C), 153.1 (C), 161.0 (C), 162.5 (C), 167.9 (C). HRMS (ESI) m/z: 418.1625 calcd for $C_{23}H_{23}F_3NO_3^+$ [M + H]$^+$; found 418.1630.

*Methyl 2-(3-(4-chlorophenyl)-4-phenylisoxazol-5-yl)acetate (**13c**).* Compound **13c** was prepared following the general procedure **F** from methyl 2-(4-iodo-3-(aryl)isoxazol-5-yl)acetate **12d** (377 mg, 1.0 mmol) and phenylboronic acid (244 mg, 2.0 mmol), $NaHCO_3$ (252 mg, 3.0 mmol) and Pd(dppf)Cl_2 (37 mg, 0.05 mmol) in dioxane (6 mL) and water (2 mL) in 271 mg (83% yield) after column chromatography on silica (light petroleum/ethyl acetate, 20:1, v/v) as a colorless solid: mp 74-75 °C (light petroleum/ethyl acetate). ^{1}H NMR ($CDCl_3$, 400 MHz): δ 3.74 (s, 3H), 3.81 (s, 2H), 7.19-7.21 (m, 2H), 7.27-7.31 (m, 2H), 7.37-7.40 (m, 3H). ^{13}C NMR ($CDCl_3$, 101 MHz): δ 31.8 (CH_2), 52.7 (CH_3), 118.0 (C), 127.2 (C), 128.4 (CH), 128.8 (CH), 129.0 (CH), 129.1 (C), 129.68 (CH), 129.74 (CH), 135.7 (C), 160.2 (C), 162.4 (C), 168.0 (C). HRMS (ESI) m/z: 328.0735 calcd for $C_{18}H_{15}ClNO_3^+$ [M + H]$^+$; found 328.0738.

*Methyl 2-(3-(4-chlorophenyl)-4-(4-methoxyphenyl)isoxazol-5-yl)acetate (**13d**).* Compound **13d** was prepared from methyl 2-(4-iodo-3-(aryl)isoxazol-5-yl)acetate **12d** (100 mg, 0.27 mmol), (4-methoxyphenyl)boronic acid (61 mg, 0.40 mmol), $NaHCO_3$ (68 mg, 0.281 mmol) and $PdCl_2(PPh_3)_2$ (5.6 mg, 0.068 mmol) in THF (4 mL) and water (1.3 mL). The reaction mixture was stirred at 70 °C (oil bath temperature) for 24 h. After purification by column chromatography on silica (light petroleum/ethyl acetate, 20:1, v/v) the product was obtained in 37 mg (39% yield) as a colorless oil. ^{1}H NMR ($CDCl_3$, 400 MHz): δ 3.74 (s, 3H), 3.79 (s, 2H), 3.84 (s, 3H), 6.90-6.94 (m, 2H), 7.10-7.14 (m, 2H), 7.28-7.31 (m, 2H), 7.38-7.42 (m, 2H). ^{13}C NMR ($CDCl_3$, 101 MHz): δ 31.8 (CH_2), 52.7 (CH_3), 55.3 (CH_3), 114.5 (CH), 117.6 (C), 121.1 (C), 127.3 (C), 128.8 (CH), 129.7 (CH), 131.0 (CH), 135.6 (C), 159.6 (C), 160.3 (C), 162.2 (C), 168.2 (C). HRMS (ESI) m/z: 358.0841 calcd for $C_{19}H_{17}ClNO_4^+$ [M + H]$^+$; found 358.0844.

*Methyl 2-(3-(4-bromophenyl)-4-(4-fluorophenyl)isoxazol-5-yl)acetate (**13e**).* Compound **13e** was prepared following the general procedure **F** from methyl 2-(4-iodo-3-(aryl)isoxazol-5-yl)acetate **12e** (422 mg, 1.0 mmol) and (4-fluorophenyl)boronic acid (245 mg, 1.75 mmol), $NaHCO_3$ (252 mg, 3.0 mmol) and Pd(dppf)Cl_2 (37 mg, 0.05 mmol) in dioxane (6 mL) and water (2 mL) in 220 mg (56% yield) after column chromatography on silica (light petroleum/ethyl acetate, 30:1, v/v) as a colorless oil. ^{1}H NMR ($CDCl_3$, 400 MHz): δ 3.75 (s, 3H), 3.79 (s, 2H), 7.07-7.12 (m, 2H), 7.17-7.21 (m, 2H), 7.28-7.31 (m, 2H), 7.45-7.48 (m, 2H). ^{13}C NMR ($CDCl_3$, 101 MHz): δ 31.7 (CH_2), 52.7 (CH_3), 116.1 (d, CH, J = 21.7 Hz), 117.0 (C), 124.2 (C), 125.00 (d, C, J = 3.7 Hz), 127.4 (C), 129.8 (CH), 131.5 (d, CH, J = 8.5 Hz), 131.8 (CH), 160.3 (C), 162.5 (C), 162.7 (d, C, J = 248.0 Hz), 167.9 (C). HRMS (ESI) m/z: 390.0136 calcd for $C_{18}H_{14}BrFNO_3^+$ [M + H]$^+$; found 390.0134.

*Methyl 2-(3-(4'-fluoro-[1,1'-biphenyl]-4-yl)-4-(4-fluorophenyl)isoxazol-5-yl)acetate (**13f**).* Compound **13f** was prepared following the general procedure **F** from methyl 2-(4-iodo-3-(aryl)isoxazol-5-yl)acetate **12e** (422 mg, 1.0 mmol) and (4-fluorophenyl)boronic acid (245 mg, 1.75 mmol), $NaHCO_3$ (252 mg, 3.0 mmol) and Pd(dppf)Cl_2 (37 mg, 0.05 mmol) in dioxane (6 mL) and water (2 mL) in 123 mg (30% yield) after column chromatography on silica (light petroleum/ethyl acetate, 30:1, v/v) as a brown solid: mp 89-90 °C (light petroleum/ethyl acetate). ^{1}H NMR ($CDCl_3$, 400 MHz): δ 3.76 (s, 3H), 3.80 (s, 2H), 7.08-7.15 (m, 4H), 7.22-7.25 (m, 2H), 7.46-7.50 (m, 4H), 7.52-7.57 (m, 2H). ^{13}C NMR ($CDCl_3$, 101 MHz): δ 31.8 (CH_2), 52.7 (CH_3), 115.7 (d, CH, J = 21.5 Hz), 116.0 (d, CH, J = 21.6 Hz), 117.1 (C), 125.4 (d, C, J = 3.2 Hz), 127.0 (CH), 127.4 (C), 128.6 (d, CH, J = 8.2 Hz), 128.8 (CH), 131.6 (d, CH, J = 8.4 Hz), 136.3 (d, C, J = 3.2 Hz), 141.3 (C), 160.8 (C), 162.3 (C), 162.6 (d, C, J = 248.9 Hz), 162.7 (d, C, J = 248.9), 168.0 (C). HRMS (ESI) m/z: 406.1249 calcd for $C_{24}H_{17}F_2NO_3^+$ $[M + H]^+$; found 406.1248.

General procedure G for the preparation of methyl 3-oxo-2-(isoxazol-5-yl)propanoates 1.

To a solution of methyl 2-(isoxazol-5-yl)acetate **11**, **12** or **13** (1 mmol) in dry THF (10 ml per 1 mmol of **11-13**) cooled to 0 °C (ice-water bath temperature) was added NaH (3 mmol) under Ar and the mixture was stirred at 0 °C for 10 min and then a solution of anhydride or acid chloride (1.25 mmol) in dry THF was added dropwise. The mixture was stirred for 24 h at rt (TLC control, light petroleum/ethyl acetate = 10:1, (v/v)), neutralized with sat. aq. $NaHSO_4$, and extracted with ethyl acetate. The solvent was evaporated, and the residue was purified by column chromatography on silica with light petroleum/ethyl acetate (20-10:1, v/v) as an eluent.

*Methyl 3-oxo-2-(3-phenylisoxazol-5-yl)pentanoate (**1a**).* Compound **1a** was prepared following the general procedure **G** from methyl 2-(3-(aryl)isoxazol-5-yl)acetate **11a** (282 mg, 1.3 mmol), propionic anhydride (212 mg, 1.62 mmol), NaH (160 mg, 4 mmol) in THF (15 mL) in 293 mg (83% yield) after column chromatography on silica (light petroleum/ethyl acetate, 20:1, v/v) as an yellow oil. According to NMR data, the compound in solution is approximately 100% in the enol form. ^{1}H NMR ($CDCl_3$, 400 MHz): δ 1.20 (t, J = 7.5 Hz, 3H), 2.37 (q, J = 7.6 Hz, 2H), 3.79 (s, 3H), 6.52 (s, 1H), 7.45-7.47 (m, 3H), 7.83-7.85 (m, 2H), 13.58 (s, 1H). ^{13}C NMR ($CDCl_3$, 101 MHz): δ 11.1 (CH_3), 27.2 (CH_2), 52.2 (CH_3), 91.7 (C), 103.3 (CH), 126.7 (CH), 128.9 (CH), 129.2 (C), 129.9 (CH), 162.5 (C), 166.0 (C), 171.6 (C), 183.6 (C). HRMS (ESI) m/z: 296.0893 calcd for $C_{15}H_{15}NO_4Na^+$ $[M + Na]^+$; found 296.0896.

*Methyl 2-(3-benzylisoxazol-5-yl)-3-oxo-3-(p-tolyl)propanoate (**1b**).* Compound **1b** was prepared following the general procedure **G** from methyl 2-(3-(aryl)isoxazol-5-yl)acetate **11b** (231 mg, 1.0 mmol), 2-(4-chlorophenyl)acetyl chloride (236 mg, 1.25 mmol), NaH (120 mg, 3.0 mmol) in THF (10 mL) in 159 mg (41% yield) after column chromatography on silica (light petroleum/ethyl acetate, 20:1, v/v) as a light yellow oil. According to NMR data, the compound in solution is approximately 100% in the enol form.^{1}H NMR ($CDCl_3$, 400 MHz): δ 2.42 (s, 3H), 3.62 (s, 2H), 3.79 (s, 3H), 6.49 (s, 1H), 7.18-7.20 (m, 2H), 7.25-7.29 (m, 4H), 7.71-7.74 (m, 2H), 13.57 (s, 1H). ^{13}C NMR ($CDCl_3$, 101 MHz): δ 21.4 (CH_3), 39.0 (CH_2), 52.5 (CH_3), 93.1 (C), 103.8 (CH), 126.1 (C), 126.6 (CH), 128.7 (CH), 129.6 (CH), 130.4

(CH), 133.1 (C), 133.6 (C), 140.2 (C), 162.6 (C), 165.1 (C), 171.5 (C), 179.0 (C). HRMS (ESI) m/z: 406.0817 calcd for $C_{21}H_{18}ClNO_4Na^+$ $[M + Na]^+$; found 406.0814.

*Methyl 3-(4-bromophenyl)-2-(3-(4-(tert-butyl)phenyl)isoxazol-5-yl)-3-oxopropanoate (**1c**).* Compound **1c** was prepared following the general procedure **G** from methyl 2-(3-(aryl)isoxazol-5-yl)acetate **11c** (355 mg, 1.3 mmol), 4-bromobenzoyl chloride (357 mg, 1.63 mmol), NaH (156 mg, 3.9 mmol) in THF (13 mL) in 470 mg (79% yield) after column chromatography on silica (light petroleum/ethyl acetate, 20:1, v/v) as a colorless solid: mp 98-100 °C (light petroleum/ethyl acetate). ^{1}H NMR ($CDCl_3$, 400 MHz): δ 1.34 (s, 9H), 3.85 (s, 3H), 6.34 (s, 1H), 7.23-7.26 (m, 2H), 7.39-7.43 (m, 2H), 7.45-7.49 (m, 2H), 7.69-7.64 (m, 2H), 13.85 (s, 1H). Spectra show ~20% keto form. ^{1}H NMR ($CDCl_3$, 400 MHz): δ 1.34 (s, 9H), 3.82 (s, 3H), 5.87 (s, 1H), 6.80 (s, 1H), 7.44-7.48 (m, 2H), 7.63-7.66 (m, 2H), 7.69-7.74 (m, 2H), 7.85-7.89 (m, 2H). ^{13}C NMR ($CDCl_3$, 101 MHz): δ 31.2 (CH_3), 34.8 (C), 52.7 (CH_3), 53.0 (CH_3), 53.5 (CH), 92.2 (C), 103.1 (CH), 103.9 (CH), 125.6 (C), 125.8 (CH), 125.9 (CH), 126.0 (C), 126.1 (C), 126.4 (CH), 126.6 (CH), 129.8 (CH), 130.5 (CH), 131.5 (CH), 132.3 (C), 132.4 (CH), 133.5 (C), 153.4 (C), 153.6 (C), 162.5 (C), 163.6 (C), 165.5 (C), 165.7 (C), 171.1 (C), 172.1 (C), 174.5 (C), 188.5 (C) (all forms signals are reported). HRMS (ESI) m/z: 456.0805 calcd for $C_{23}H_{23}BrNO_4^+$ $[M + H]^+$; found 456.0809.

*Methyl 3-(4-bromophenyl)-2-(3-(4-methoxyphenyl)isoxazol-5-yl)-3-oxopropanoate (**1d**).* Compound **1d** was prepared following the general procedure **G** from methyl 2-(3-(aryl)isoxazol-5-yl)acetate **11d** (321 mg, 1.3 mmol), 4-bromobenzoyl chloride (357 mg, 1.63 mmol), NaH (156 mg, 3.9 mmol) in THF (13 mL) in 507 mg (91% yield) after column chromatography on silica (light petroleum/ethyl acetate, 20:1, v/v) as a colorless solid: mp 121-123 °C (light petroleum/ethyl acetate). ^{1}H NMR ($CDCl_3$, 400 MHz): δ 3.85 (s, 6H), 6.30 (s, 1H), 6.93-6.98 (m, 2H), 7.24-7.27 (m, 2H), 7.40-7.44 (m, 2H), 7.68-7.72 (m, 2H), 13.86 (s, 1H). Spectra show ~13% keto form. ^{1}H NMR ($CDCl_3$, 400 MHz): δ 3.82 (s, 3H), 3.85 (s, 3H), 5.86 (s, 1H), 6.76 (s, 1H), 7.60-7.63 (m, 2H), 7.64-7.67 (m, 2H), 7.86-7.90 (m, 2H), 7.93-7.97 (m, 2H). ^{13}C NMR ($CDCl_3$, 101 MHz): δ 52.7 (CH_3), 55.4 (CH_3), 92.2 (C), 103.7 (CH), 114.3 (CH), 121.5 (C), 126.0 (C), 128.1 (CH), 129.8 (CH), 131.5 (CH), 132.4 (C), 161.0 (C), 162.2 (C), 165.5 (C), 172.0 (C), 174.5 (C) (only major enol form signals are reported). HRMS (ESI) m/z: 452.0104 calcd for $C_{20}H_{16}BrNO_5Na^+$ $[M + Na]^+$; found 452.0107.

*Methyl 2-(3-(4-fluorophenyl)isoxazol-5-yl)-3-oxo-3-phenylpropanoate (**1e**).* Compound **1e** was prepared following the general procedure **G** from methyl 2-(3-(aryl)isoxazol-5-yl)acetate **11e** (306 mg, 1.3 mmol), benzoyl chloride (230 mg, 1.63 mmol), NaH (156 mg, 3.9 mmol) in THF (13 mL) in 404 mg (92% yield) after column chromatography on silica (light petroleum/ethyl acetate, 20:1, v/v) as a colorless solid: mp 90-92 °C (light petroleum/ethyl acetate). According to NMR data, the compound in solution is approximately 100% in the enol form.^{1}H NMR ($CDCl_3$, 400 MHz): δ 3.85 (S, 3H), 6.27 (s, 1H), 7.08-7.14 (m, 2H), 7.26-7.31 (m, 2H), 7.36 (m, 3H), 7.71-7.76 (m, 2H), 13.88 (s, 1H). ^{13}C NMR ($CDCl_3$, 101 MHz): δ 52.6 (CH_3), 103.6 (CH), 115.9 (d, CH, J = 21.7 Hz), 125.3 (d, C, J = 3.1 Hz), 128.22 (CH), 128.24 (CH), 128.5 (CH), 128.6 (d, CH, J = 8.5 Hz), 133.5 (C), 161.6 (C), 163.7 (d, C, J = 249.8 Hz),

166.5 (C), 170.8 (C), 172.1 (C), 176.2 (C). HRMS (ESI) m/z: 362.0799 calcd for $C_{19}H_{14}FNO_4Na^+$ [M + Na]$^+$; found 362.0804.

*Methyl 2-(3-(4-chlorophenyl)isoxazol-5-yl)-3-oxo-3-phenylpropanoate (**1f**).* Compound **1f** was prepared following the general procedure **G** from methyl 2-(3-(aryl)isoxazol-5-yl)acetate **11f** (189 mg, 0.75 mmol), benzoyl chloride (131 mg, 0.94 mmol), NaH (90 mg, 2.25 mmol) in THF (8 mL) in 257 mg (97% yield) after column chromatography on silica (light petroleum/ethyl acetate, 20:1, v/v) as a colorless solid: mp 106-108 °C (light petroleum/ethyl acetate). According to NMR data, the compound in solution is approximately 100% in the enol form.^{1}H NMR ($CDCl_3$, 400 MHz): δ 3.85 (s, 3H), 6.28 (s, 1H), 7.26-7.30 (m, 2H), 7.36-7.41 (m, 5H), 7.67-7.70 (m, 2H), 13.89 (s, 1H). ^{13}C NMR ($CDCl_3$, 101 MHz): δ 52.6 (CH_3), 91.8 (C), 103.6 (CH), 127.6 (C), 128.0 (CH), 128.2 (CH), 128.2 (CH), 129.1 (CH), 131.3 (CH), 133.5 (C), 135.9 (C), 161.5 (C), 166.6 (C), 172.0 (C), 176.2 (C). HRMS (ESI) m/z: 378.0504 calcd for $C_{19}H_{14}ClNO_4Na^+$ [M + Na]$^+$; found 378.0501.

*Methyl 2-(3-(4-chlorophenyl)isoxazol-5-yl)-3-oxo-3-(p-tolyl)propanoate (**1g**).* Compound **1g** was prepared following the general procedure **G** from methyl 2-(3-(aryl)isoxazol-5-yl)acetate **11f** (326 mg, 1.3 mmol), 4-methylbenzoyl chloride (251 mg, 1.63 mmol), NaH (156 mg, 3.9 mmol) in THF (13 mL) in 456 mg (95% yield) after column chromatography on silica (light petroleum/ethyl acetate, 20:1, v/v) as a colorless solid: mp 108-110 °C (light petroleum/ethyl acetate). ^{1}H NMR ($CDCl_3$, 400 MHz): δ 2.31 (s, 3H), 3.84 (s, 3H), 6.31 (s, 1H), 7.07-7.09 (m, 2H), 7.26-7.28 (m, 2H), 7.38-7.43 (m, 2H), 7.69-7.71 (m, 2H), 13.89 (s, 1H). Spectra show ~23% keto form. ^{1}H NMR ($CDCl_3$, 400 MHz): δ 2.42 (s, 3H), 3.81 (s, 3H), 5.94 (s, 1H), 6.81 (s, 1H), 7.28-7.31 (m, 2H), 7.38-7.43 (m, 2H), 7.72-7.75 (m, 2H), 7.91-7.93 (m, 2H). ^{13}C NMR ($CDCl_3$, 101 MHz): δ 21.5 (CH_3), 21.7 (CH_3), 52.6 (CH_3), 52.7 (CH_3), 53.5 (CH), 102.9 (CH), 103.5 (CH), 127.2 (C), 127.7 (C), 128.0 (CH), 128.1 (CH), 128.3 (CH), 128.9 (CH), 129.1 (CH), 129.16 (CH), 129.19 (CH), 129.3 (CH), 130.6 (C), 132.2 (C), 135.9 (C), 136.2 (C), 141.9 (C), 144.5 (C), 145.8 (C), 161.5 (C), 161.9 (C), 164.9 (C), 166.0 (C), 166.9 (C), 172.1 (C), 176.3 (C), 188.8 (C) (all forms signals are reported). HRMS (ESI) m/z: 392.0660 calcd for $C_{20}H_{16}ClNO_4Na^+$ [M + Na]$^+$; found 392.0656.

*Methyl 2-(3-(4-bromophenyl)isoxazol-5-yl)-3-oxo-3-phenylpropanoate (**1h**).* Compound **1h** was prepared following the general procedure **G** from methyl 2-(3-(aryl)isoxazol-5-yl)acetate **11g** (157 mg, 0.53 mmol), benzoyl chloride (91 mg, 0.65 mmol), NaH (60 mg, 1.5 mmol) in THF (7 mL) in 201 mg (95% yield) after column chromatography on silica (light petroleum/ethyl acetate, 20:1, v/v) as a colorless solid: mp 118-119 °C (light petroleum/ethyl acetate). According to NMR data, the compound in solution is approximately 100% in the enol form.^{1}H NMR ($CDCl_3$, 400 MHz): δ 3.85 (s, 3H), 6.28 (s, 1H), 7.26-7.30 (m, 2H), 7.36-7.40 (m, 3H), 7.54-7.57 (m, 2H), 7.60-7.63 (m, 2H), 13.89 (s, 1H). ^{13}C NMR ($CDCl_3$, 101 MHz): δ 52.6 (CH_3), 91.8 (C), 103.6 (CH), 124.2 (C), 128.06 (C), 128.19 (CH), 128.23 (CH), 128.23 (CH), 131.3 (CH), 132.1 (CH), 133.5 (C), 161.6 (C), 166.6 (C), 172.0 (C), 176.2 (C). HRMS (ESI) m/z: 421.9998 calcd for $C_{19}H_{14}BrNO_4Na^+$ [M + Na]$^+$; found 421.9992.

*Methyl 2-(3-(4-bromophenyl)isoxazol-5-yl)-3-oxobutanoate (**1i**).* Compound **1i** was prepared following the general procedure **G** from methyl 2-(3-(aryl)isoxazol-5-yl)acetate **11g** (320 mg, 1.3 mmol), acetyl

chloride (0.12 mL, 1.62 mmol), NaH (160 mg, 4 mmol) in THF (16 mL) in 192 mg (44% yield) after column chromatography on silica (light petroleum/ethyl acetate, 20:1, v/v) as a colorless solid: mp 81-83 °C (light petroleum/ethyl acetate). Spectra show ~100% enol form ^{1}H NMR ($CDCl_3$, 400 MHz): δ 2.14 (s, 3H), 3.80 (s, 3H), 6.50 (s, 1H), 7.59-7.62 (m, 2H), 7.69-7.72 (m, 2H), 13.58 (s, 1H). ^{13}C NMR ($CDCl_3$, 101 MHz): δ 20.6 (CH_3), 52.3 (CH_3), 92.7 (C), 102.9 (CH), 124.2 (C), 128.1 (C), 128.2 (CH), 132.1 (CH), 161.5 (C), 166.3 (C), 171.2 (C), 179.7 (C). HRMS (ESI) m/z: 359.9842 calcd for $C_{14}H_{12}BrNO_4Na^+$ [M + Na]$^+$; found 359.9843.

Methyl 3-oxo-2-(3-(quinolin-2-yl)isoxazol-5-yl)-3-(p-tolyl)propanoate (1j). Compound **1j** was prepared following the general procedure **G** from methyl 2-(3-(aryl)isoxazol-5-yl)acetate **11h** (348 mg, 1.3 mmol), 4-methylbenzoyl chloride (251 mg, 1.63 mmol), NaH (156 mg, 3.9 mmol) in THF (13 mL) in 287 mg (57% yield) after column chromatography on silica (light petroleum/ethyl acetate, 20:1, v/v) as a colorless oil. ^{1}H NMR ($CDCl_3$, 400 MHz): δ 2.29 (s, 3H), 3.83 (s, 3H), 5.97 (s, 1H), 7.06-7.08 (m, 2H), 7.28-7.34 (m, 2H), 7.56-7.59 (m, 1H), 7.72-7.75 (m, 1H), 7.83-7.86 (m, 1H), 8.10-8.14 (m, 1H), 8.16-8.20 (m, 1H), 8.22-8.25 (m, 1H), 13.92 (s, 1H). Spectra show ~50% keto form. ^{1}H NMR ($CDCl_3$, 400 MHz): δ 2.41 (s, 3H), 3.84 (s, 3H), 6.93 (s, 1H), 7.28-7.34 (m, 3H), 7.56-7.59 (m, 1H), 7.71-7.76 (m, 1H), 7.83-7.86 (m, 1H), 7.92-7.94 (m, 2H), 8.10-8.14 (m, 1H), 8.16-8.20 (m, 1H), 8.22-8.25 (m, 1H). ^{13}C NMR ($CDCl_3$, 101 MHz): δ 21.4 (CH_3), 21.7 (CH_3), 52.5 (CH), 53.1 (CH_3), 53.4 (CH_3), 91.2 (C), 104.2 (CH), 104.9 (CH), 119.0 (CH), 119.1 (CH), 127.2 (CH), 127.3 (CH), 127.6 (CH), 127.7 (CH), 128.31 (CH), 128.31 (C), 128.34, 129.0 (CH), 129.2 (CH), 129.6 (CH), 129.7 (CH), 129.82 (CH), 128.85 (CH), 129.9 (CH), 130.7 (C), 132.3 (C), 136.8 (CH), 136.9 (CH), 141.8 (C), 145.5 (C), 147.9 (C), 148.0 (C), 148.2 (C), 148.8 (C), 163.8 (C), 164.0 (C), 164.7 (C), 166.0 (C), 166.7 (C), 172.2 (C), 176.2 (C), 188.7 (C) (all forms signals are reported). HRMS (ESI) m/z: 387.1339 calcd for $C_{23}H_{19}N_2O_4^+$ [M + H]$^+$; found 387.1338.

Methyl 2-(3-benzylisoxazol-5-yl)-3-oxo-3-(p-tolyl)propanoate (1k). Compound **1k** was prepared following the general procedure **G** from methyl 2-(3-(alkyl)isoxazol-5-yl)acetate **11i** (300 mg, 1.3 mmol), 4-methylbenzoyl chloride (251 mg, 1.63 mmol), NaH (156 mg, 3.9 mmol) in THF (13 mL) in 380 mg (84% yield) after column chromatography on silica (light petroleum/ethyl acetate, 20:1, v/v) as a colorless solid: mp 69-71 °C (light petroleum/ethyl acetate). ^{1}H NMR ($CDCl_3$, 400 MHz): δ 2.33 (s, 3H), 3.76 (s, 3H), 3.95-3.99 (m, 2H), 5.73 (s, 1H), 7.16-7.18 (m, 2H), 7.27-7.31 (m, 5H), 7.85-7.88 (m, 2H), 13.77 (s, 1H). Spectra show ~50% keto form. ^{1}H NMR ($CDCl_3$, 400 MHz): δ 2.42 (s, 3H), 3.80 (s, 3H), 3.95-3.99 (m, 2H), 5.84 (s, 1H), 6.28 (s, 1H), 7.03-7.05 (m, 2H), 7.11-7.14 (m, 2H), 7.20-7.25 (m, 5H). ^{13}C NMR ($CDCl_3$, 101 MHz): δ 21.5 (CH_3), 21.7 (CH_3), 32.4 (CH_2), 32.5 (CH_2), 52.5 (CH_3), 52.8 (CH), 53.3 (CH_3), 91.4 (C), 104.8 (CH), 105.9 (CH), 126.6 (CH), 126.9 (CH), 128.2 (CH), 128.5 (CH), 128.6 (CH), 128.72 (CH), 128.75 (CH), 128.8 (CH), 129.2 (CH), 129.7 (CH), 130.2 (CH), 130.6 (C), 132.3 (C), 136.8 (C), 137.4 (C), 141.6 (C), 145.6 (C), 166.1 (C), 172.1 (C), 176.0 (C), 188.9 (C) (all forms signals are reported). HRMS (ESI) m/z: 372.1206 calcd for $C_{21}H_{19}NO_4Na^+$ [M + Na]$^+$; found 372.1211.

Methyl 2-(3-heptylisoxazol-5-yl)-3-oxo-3-(p-tolyl)propanoate (1l). Compound **1l** was prepared following the general procedure **G** from methyl 2-(3-(alkyl)isoxazol-5-yl)acetate **11j** (311 mg, 1.3 mmol), benzoyl

chloride (251 mg, 1.63 mmol), NaH (156 mg, 3.9 mmol) in THF (13 mL) in 253 mg (55% yield) after column chromatography on silica (light petroleum/ethyl acetate, 20:1, v/v) as a light yellow oil. ^{1}H NMR ($CDCl_3$, 400 MHz): δ 0.88-0.89 (m, 3H), 1.24-1.34 (m, 8H), 1.54-1.67 (m, 2H), 2.32 (s, 3H), 2.58-2.64 (m, 2H), 3.81 (s, 1H), 5.81 (s, 1H), 7.05-7.08 (m, 2H), 7.20-7.22 (m, 2H), 13.79 (s, 1H). Spectra show ~44% keto form. ^{1}H NMR ($CDCl_3$, 400 MHz): δ 0.88-0.89 (m, 3H), 1.24-1.34 (m, 8H), 1.54-1.67 (m, 2H), 2.42 (s, 3H), 2.58-2.64 (m, 2H), 3.79 (s, 1H), 5.84 (s, 1H), 6.32 (s, 1H), 7.27-7.29 (m, 2H), 7.87-7.89 (m, 2H). ^{13}C NMR ($CDCl_3$, 101 MHz): δ 14.0 (CH_3), 14.1 (CH_3), 21.4 (CH_3), 21.7 (CH_3), 22.56 (CH_2), 22.60 (CH_2), 26.0 (CH_2), 26.1 (CH_2), 28.0 (CH_2), 28.1 (CH_2), 28.86 (CH_2), 28.93 (CH_2), 28.93 (CH_2), 29.1 (CH_2), 31.6 (CH_2), 31.7 (CH_2), 52.4 (CH_3), 52.8 (CH_3), 53.3 (CH), 91.5 (C), 104.5 (CH), 105.4 (CH), 128.3 (CH), 128.8 (CH), 129.2 (CH), 129.7 (CH), 130.7 (C), 132.3 (C), 141.6 (C), 145.5 (C), 163.5 (C), 164.3 (C), 164.6 (C), 165.5 (C), 166.2 (C), 172.3 (C), 175.7 (C), 189.1 (C) (all forms signals are reported). HRMS (ESI) m/z: 358.2013 calcd for $C_{21}H_{28}NO_4^+$ [M + H]$^+$; found 358.2011.

*Methyl 2-(3-(4-chlorophenyl)-4-iodoisoxazol-5-yl)-3-oxo-3-phenylpropanoate (**1m**).* Compound **1m** was prepared following the general procedure **G** from methyl 2-(3-(aryl)isoxazol-5-yl)acetate **12d** (245 mg, 0.65 mmol), benzoyl chloride (114 mg, 0.81 mmol), NaH (70 mg, 1.95 mmol) in THF (7 mL) in 149 mg (48% yield) after column chromatography on silica (light petroleum/ethyl acetate, 20:1, v/v) as a colorless oil. ^{1}H NMR ($CDCl_3$, 400 MHz): δ 3.86 (s, 3H), 7.26-7.31 (m, 2H), 7.36-7.39 (m, 2H), 7.40-7.45 (m, 3H), 7.70-7.74 (m, 2H), 13.78 (s, 1H). ^{13}C NMR ($CDCl_3$, 101 MHz): δ 52.6 (CH_3), 64.1 (C), 90.9 (C), 127.1 (C), 128.0 (CH), 128.3 (CH), 128.8 (CH), 129.7 (CH), 131.6 (CH), 133.2 (C), 136.3 (C), 162.3 (C), 169.1 (C), 171.6 (C), 177.2 (C). HRMS (ESI) m/z: 503.9470 calcd for $C_{19}H_{13}ClINO_4Na^+$ [M + Na]$^+$; found 503.9477.

*Methyl 3-oxo-2-(3-phenyl-4-(thiophen-3-yl)isoxazol-5-yl)-3-(p-tolyl)propanoate (**1n**).* Compound **1n** was prepared following the general procedure **G** from methyl 2-(3-(aryl)isoxazol-5-yl)acetate **13a** (227 mg, 0.76 mmol), 4-methylbenzoyl chloride (146 mg, 0.95 mmol), NaH (91 mg, 2.28 mmol) in THF (8 mL) in 312 mg (98% yield) after column chromatography on silica (light petroleum/ethyl acetate, 20:1, v/v) as a light yellow solid: mp 95-97 °C (light petroleum/ethyl acetate). According to NMR data, the compound in solution is approximately 100% in the enol form. ^{1}H NMR ($CDCl_3$, 400 MHz): δ 2.30 (s, 3H), 3.79 (s, 3H), 6.47-6.49 (m, 1H), 6.61-6.63 (m, 1H), 7.02-7.04 (m, 2H), 7.14-7.17 (m, 3H), 7.32-7.33 (m, 2H), 7.35-7.38 (m, 1H), 7.46-7.49 (m, 2H), 13.72 (s, 1H). ^{13}C NMR ($CDCl_3$, 101 MHz): δ 21.4 (CH_3), 52.4 (CH_3), 90.6 (C), 114.2 (C), 123.6 (CH), 125.4 (CH), 127.8 (CH), 127.9 (CH), 128.3 (CH), 128.4 (CH), 128.8 (CH), 129.1 (C), 129.49 (CH), 129.54 (C), 130.6 (C), 141.8 (C), 161.4 (C), 163.5 (C), 172.3 (C), 176.6 (C). HRMS (ESI) m/z: 418.1108 calcd for $C_{24}H_{20}NO_4S^+$ [M + H]$^+$; found 418.1106.

*Methyl 2-(3-(4-(tert-butyl)phenyl)-4-(4-(trifluoromethyl)phenyl)isoxazol-5-yl)-3-oxo-3-(p-tolyl)propanoate (**1o**).* Compound **1o** was prepared following the general procedure **G** from methyl 2-(3-(aryl)isoxazol-5-yl)acetate **13b** (91 mg, 0.22 mmol), 4-methylbenzoyl chloride (43 mg, 0.27 mmol), NaH (26 mg, 0.65 mmol) in THF (3 mL) in 72 mg (62% yield) after column chromatography on silica (light petroleum/ethyl acetate, 20:1, v/v) as a colorless solid: mp 138-140 °C (light petroleum/ethyl acetate).

According to NMR data, the compound in solution is approximately 100% in the enol form.^{1}H NMR ($CDCl_3$, 400 MHz): δ 1.29 (s, 9H), 2.29 (s, 3H), 3.81 (s, 3H), 6.77-6.79 (m, 2H), 6.96-7.03 (m, 4H), 7.30-7.35 (m, 4H), 7.38-7.40 (m, 2H), 13.65 (s, 1H). ^{13}C NMR ($CDCl_3$, 101 MHz): δ 21.4 (CH_3), 31.2 (CH_3), 34.8 (C), 52.5 (CH_3), 90.3 (C), 117.3 (C), 122.7 (C), 125.2 (q, CH, J = 3.7 Hz), 125.6 (CH), 127.78 (CH), 127.84 (CH), 128.8 (CH), 129.3 (C), 129.5 (CH), 129.6 (C), 130.4 (C), 134.1 (C), 141.8 (C), 153.0 (C), 160.8 (C), 164.3 (C), 172.3 (C), 176.7 (C). HRMS (ESI) m/z: 536.2043 calcd for $C_{31}H_{29}F_3NO_4^+$ [M + H]$^+$; found 536.2048.

*Methyl 2-(3-(4-chlorophenyl)-4-phenylisoxazol-5-yl)-3-oxo-3-(p-tolyl)propanoate (**1p**).* Compound **1p** was prepared following the general procedure **G** from methyl 2-(3-(aryl)isoxazol-5-yl)acetate **13c** (250 mg, 0.76 mmol), 4-methylbenzoyl chloride (147 mg, 0.96 mmol), NaH (92 mg, 2.3 mmol) in THF (13 mL) in 334 mg (98% yield) after column chromatography on silica (light petroleum/ethyl acetate, 20:1, v/v) as a colorless solid: mp 103-105 °C (light petroleum/ethyl acetate). According to NMR data, the compound in solution is approximately 100% in the enol form.^{1}H NMR ($CDCl_3$, 400 MHz): δ 2.30 (s, 3H), 3.79 (s, 3H), 6.63-6.65 (m, 2H), 6.99-7.01 (m, 2H), 7.05-7.08 (m, 2H), 7.13-7.18 (m, 2H), 7.20-7.26 (m, 3H), 7.35-7.39 (m, 2H), 11.64 (s, 1H). ^{13}C NMR ($CDCl_3$, 101 MHz): δ 21.4 (CH_3), 52.4 (CH_3), 90.5 (C), 118.7 (C), 127.5 (C), 127.6 (CH), 127.9 (CH), 128.4 (CH), 128.69 (CH), 128.73 (CH), 129.1 (CH), 129.5 (CH), 129.6 (C), 130.6 (C), 135.5 (C), 141.7 (C), 160.2 (C), 164.0 (C), 172.3 (C), 176.7 (C). HRMS (ESI) m/z: 446.1154 calcd for $C_{26}H_{21}ClNO_4^+$ [M + H]$^+$; found 446.1158.

*Methyl 2-(3-(4-chlorophenyl)-4-(4-methoxyphenyl)isoxazol-5-yl)-3-oxo-3-(p-tolyl)propanoate (**1q**).* Compound **1q** was prepared following the general procedure **G** from methyl 2-(3-(aryl)isoxazol-5-yl)acetate **13d** (118 mg, 0.33 mmol), 4-methylbenzoyl chloride (76 mg, 0.55 mmol), NaH (40 mg, 0.99 mmol) in THF (4 mL) in 64 mg (41% yield) after column chromatography on silica (light petroleum/ethyl acetate, 20:1, v/v) as a colorless oil. According to NMR data, the compound in solution is approximately 100% in the enol form.^{1}H NMR ($CDCl_3$, 400 MHz): δ 2.30 (s, 3H), 3.78 (s, 3H), 3.79 (s, 3H), 6.53-6.57 (m, 2H), 6.67-6.71 (m, 2H), 7.00-7.04 (m, 2H), 7.08-7.10 (m, 2H), 7.24-7.27 (m, 2H), 7.35-7.40 (m, 2H), 13.64 (s, 1H). ^{13}C NMR ($CDCl_3$, 101 MHz): δ 21.5 (CH_3), 52.4 (CH_3), 55.2 (CH_3), 90.5 (C), 113.9 (CH), 118.4 (C), 121.8 (C), 127.7 (C), 127.9 (CH), 128.69 (CH), 128.74 (CH), 129.5 (CH), 130.3 (CH), 130.6 (C), 135.5 (C), 141.7 (C), 159.1 (C), 160.3 (C), 163.8 (C), 172.4 (C), 176.6 (C). HRMS (ESI) m/z: 476.1259 calcd for $C_{27}H_{23}ClNO_5^+$ [M + H]$^+$; found 476.1266.

*Methyl 3-(4-bromophenyl)-2-(3-(4-bromophenyl)-4-(4-fluorophenyl)isoxazol-5-yl)-3-oxopropanoate (**1r**).* Compound **1r** was prepared following the general procedure **G** from methyl 2-(3-(aryl)isoxazol-5-yl)acetate **13e** (69 mg, 0.18 mmol), 4-bromobenzoyl chloride (48 mg, 0.22 mmol), NaH (21 mg, 0.53 mmol) in THF (3 mL) in 59 mg (59% yield) after column chromatography on silica (light petroleum/ethyl acetate, 20:1, v/v) as a colorless solid: mp 143-145 °C (light petroleum/ethyl acetate). According to NMR data, the compound in solution is approximately 100% in the enol form. ^{1}H NMR ($CDCl_3$, 400 MHz): δ 3.84 (s, 3H), 6.56-6.61 (m, 2H), 6.85-6.91 (m, 2H), 6.96-6.99 (m, 2H), 7.26-7.29 (m, 2H), 7.31-7.34 (m, 2H), 7.42-7.46 (m, 2H), 13.62 (s, 1H). ^{13}C NMR ($CDCl_3$, 101 MHz): δ 52.7 (CH_3), 91.2 (C), 115.73 (d,

CH, *J* = 21.6 Hz), 117.8 (C), 124.2 (C), 125.3 (d, C, *J* = 3.5 Hz), 125.9 (C), 127.5 (C), 129.3 (CH), 129.7 (CH), 130.7 (d, CH, *J* = 8.3 Hz), 131.3 (CH), 131.8 (CH), 132.1 (C), 160.3 (C), 162.3 (d, C, *J* = 248.3 Hz), 163.6 (C), 172.1 (C), 175.2 (C). HRMS (ESI) m/z: 573.9484 calcd for $C_{25}H_{17}Br_2FNO_4^+$ [M + H]$^+$; found 573.9489.

*Methyl 3-(4-bromophenyl)-2-(3-(4'-fluoro-[1,1'-biphenyl]-4-yl)-4-(4-fluorophenyl)isoxazol-5-yl)-3-oxopropanoate (**1s**).* Compound **1s** was prepared following the general procedure **G** from methyl 2-(3-(aryl)isoxazol-5-yl)acetate **13f** (69 mg, 0.17 mmol), 4-bromobenzoyl chloride (47 mg, 0.21 mmol), NaH (20 mg, 0.5 mmol) in THF (3 mL) in 80 mg (80% yield) after column chromatography on silica (light petroleum/ethyl acetate, 20:1, v/v) as a colorless solid: mp 124-126 °C (light petroleum/ethyl acetate). According to NMR data, the compound in solution is approximately 100% in the enol form. ^{1}H NMR ($CDCl_3$, 400 MHz): *δ* 3.86 (s, 3H), 6.63-6.67 (m, 2H), 6.86-6.91 (m, 2H), 6.98-7.02 (m, 2H), 7.09-7.15 (m, 2H), 7.31-7.35 (m, 2H), 7.45-7.48 (m, 4H), 7.50-7.56 (m, 2H), 13.63 (s, 1H). ^{13}C NMR ($CDCl_3$, 101 MHz): *δ* 52.7 (CH_3), 91.34 (C), 115.7 (d, CH, *J* = 21.6 Hz), 115.8 (d, CH, *J* = 21.6 Hz), 117.9 (C), 125.7 (d, C, *J* = 3.2 Hz), 125.8 (C), 127.1 (CH), 127.4 (C), 128.63 (d, CH, *J* = 8.2 Hz), 128.66 (CH), 129.3 (CH), 130.8 (d, CH, *J* = 7.9 Hz), 131.4 (CH), 132.2 (C), 136.3 (d, C, *J* = 3.2 Hz), 141.4 (C), 160.8 (C), 162.3 (d, C, *J* = 248.1), 162.7 (d, C, *J* = 247.4), 163.4 (C), 172.2 (C), 175.2 (C). HRMS (ESI) m/z: 610.0436 calcd for $C_{31}H_{20}BrF_2NO_4Na^+$ [M + Na]$^+$; found 610.0433.

General procedure H for the preparation of methyl 4-oxo-1,4-dihydropyridine-3-carboxylates 2.

A mixture methyl 3-oxo-2-(isoxazol-5-yl)propanoate **1** (1 mmol) and $Mo(CO)_6$ (0.5 mmol) in acetonitrile was heated at 75 ºC (oil bath temperature) for 14 h (TLC control, light petroleum/ethyl acetate = 8:1, (v/v)), the solvent was evaporated, and the residue was purified by column chromatography on silica with light petroleum/ethyl acetate, 8:1 (v/v) as an eluent.

*Methyl 2-ethyl-4-oxo-6-phenyl-1,4-dihydropyridine-3-carboxylate (**2a**).* Compound **2a** was prepared following the general procedure **H** from isoxazole **1a** (260 mg, 0.95 mmol), $Mo(CO)_6$ (138 mg, 0.52 mmol) in acetonitrile (10 mL) in 72 mg (30% yield) after column chromatography on silica (light petroleum/ethyl acetate, 8:1, v/v) as a brown solid: mp 156-158 °C (light petroleum/ethyl acetate). ^{1}H NMR ($CDCl_3$, 400 MHz): *δ* 1.35 (t, *J* = 7.4 Hz, 3H), 2.37 (q, *J* = 7.4 Hz, 2H), 4.02 (s, 3H), 7.20 (s, 1H), 7.43-7.49 (m, 3H), 8.04-8.07 (m, 2H), 11.75 (s, 1H). ^{13}C NMR ($CDCl_3$, 101 MHz): *δ* 13.6 (CH_3), 32.0 (CH_2), 52.5 (CH_3), 107.1 (CH), 108.1 (C), 127.2 (CH), 128.7 (CH), 129.8 (CH), 138.1 (C), 159.4 (C), 165.7 (C), 169.6 (C), 171.4 (C). HRMS (ESI) m/z: 280.0944 calcd for $C_{15}H_{15}NO_3Na^+$ [M + Na]$^+$; found 280.0949.

*Methyl 2-(4-chlorobenzyl)-4-oxo-6-(p-tolyl)-1,4-dihydropyridine-3-carboxylate (**2b**).* Compound **2b** was prepared following the general procedure **H** from isoxazole **1b** (135 mg, 0.35 mmol), $Mo(CO)_6$ (51 mg, 0.19 mmol) in acetonitrile (6 mL) in 22 mg (17% yield) after column chromatography on silica (light petroleum/ethyl acetate, 8:1 + 5% chloroform, v/v) as a colorless solid: mp 171-173 °C (light petroleum/ethyl acetate). ^{1}H NMR ($CDCl_3$, 400 MHz): *δ* 2.42 (s, 3H), 3.90 (s, 3H), 4.52 (s, 2H), 7.14-

7.18 (m, 2H), 7.22 (s, 1H), 7.24-7.25 (m, 2H), 7.27-7.29 (m, 2H), 7.95-7.97 (m, 2H), 11.74 (s, 1H). ^{13}C NMR ($CDCl_3$, 101 MHz): δ 21.4 (CH_3), 44.3 (CH_2), 52.4 (CH_3), 106.9 (CH), 127.2 (CH), 128.3 (CH), 129.5 (CH), 129.9 (CH), 131.8 (C), 135.1 (C), 138.4 (C), 140.3 (C), 159.8 (C), 162.1 (C), 169.6 (C), 179.9 (C), 179.0 (C). HRMS (ESI) m/z: 386.1048 calcd for $C_{21}H_{19}ClNO_3^+$ [M + H]$^+$; found 368.1046.

*Methyl 2-(4-bromophenyl)-6-(4-(tert-butyl)phenyl)-4-oxo-1,4-dihydropyridine-3-carboxylate (**2c**).* Compound **2c** was prepared following the general procedure **H** from isoxazole **1c** (290 mg, 0.64 mmol), $Mo(CO)_6$ (84 mg, 0.32 mmol) in acetonitrile (7 mL) in 155 mg (54% yield) after column chromatography on silica (light petroleum/ethyl acetate, 8:1 + 5% chloroform, v/v) as a colorless solid: mp 137-139 °C (light petroleum/ethyl acetate). ^{1}H NMR ($CDCl_3$, 400 MHz): δ 1.35 (s, 9H), 3.63 (s, 3H), 7.32 (s, 1H), 7.38-7.42 (m, 2H), 7.47-7.50 (m, 2H), 7.55-7.57 (m, 2H), 7.99-8.01 (m, 2H), 11.20 (s, 1H). ^{13}C NMR ($CDCl_3$, 101 MHz): δ 32.2 (CH_3), 34.8 (C), 52.1 (CH_3), 106.9 (CH), 122.7 (C), 125.7 (CH), 125.8 (CH), 126.4 (C), 127.1 (CH), 129.8 (C), 130.4 (CH), 130.8 (CH), 135.0 (C), 140.8 (C), 153.5 (C), 160.0 (C), 160.9 (C), 168.4 (C), 170.9 (C). HRMS (ESI) m/z: 440.0856 calcd for $C_{23}H_{22}BrNO_3^+$ [M + H]$^+$; found 440.0863.

*Methyl 2-(4-bromophenyl)-6-(4-methoxyphenyl)-4-oxo-1,4-dihydropyridine-3-carboxylate (**2d**).* Compound **2d** was prepared following the general procedure **H** from isoxazole **1d** (447 mg, 1.04 mmol), $Mo(CO)_6$ (137 mg, 0.52 mmol) in acetonitrile (10 mL) in 172 mg (40% yield) after column chromatography on silica (light petroleum/ethyl acetate, 8:1, v/v) as a colorless solid: mp 133-135 °C (light petroleum/ethyl acetate). ^{1}H NMR ($CDCl_3$, 400 MHz): δ 3.62 (s, 3H), 3.86 (s, 3H), 6.96-6.99 (m, 3H), 7.27 (s, 1H), 7.37-7.41 (m, 2H), 7.54-7.57 (m, 2H), 8.02-8.05 (m, 2H), 11.22 (s, 1H). ^{13}C NMR ($CDCl_3$, 101 MHz): δ 52.1 (CH_3), 55.4 (CH_3), 106.1 (CH), 114.1 (CH), 122.7 (C), 128.8 (CH), 130.3 (C), 130.4 (CH), 130.8 (CH), 140.3 (C), 140.9 (C), 159.5 (C), 160.8 (C), 161.4 (C), 168.4 (C), 170.6 (C). HRMS (ESI) m/z: 414.0335 calcd for $C_{20}H_{17}BrNO_4^+$ [M + H]$^+$; found 414.0332.

*Methyl 6-(4-fluorophenyl)-4-oxo-2-phenyl-1,4-dihydropyridine-3-carboxylate (**2e**).* Compound **2e** was prepared following the general procedure **H** from isoxazole **1e** (404 mg, 1.19 mmol), $Mo(CO)_6$ (157 mg, 0.60 mmol) in acetonitrile (12 mL) in 163 mg (42% yield) after column chromatography on silica (light petroleum/ethyl acetate, 8:1, v/v) as a light brown solid: mp 169-172 °C (light petroleum/ethyl acetate). ^{1}H NMR ($CDCl_3$, 400 MHz): δ 3.59 (s, 3H), 7.12-7.16 (m, 2H), 7.29 (s, 1H), 7.42-7.46 (m, 2H), 7.48-7.52 (m, 2H), 8.06-8.10 (m, 2H), 11.17 (s, 1H). ^{13}C NMR ($CDCl_3$, 101 MHz): δ 52.1 (CH_3), 106.6 (CH), 115.7 (d, CH, J = 21.6 Hz), 127.7 (CH), 128.5 (CH), 128.6 (CH), 129.4 (d, CH, J = 8.5 Hz), 130.1 (C), 134.2 (C), 141.7 (C), 158.7 (C), 162.2 (C), 164.1 (d, C, J = 249.8 Hz), 168.4 (C), 171.1 (C). HRMS (ESI) m/z: 324.1030 calcd for $C_{19}H_{15}FNO_3^+$ [M + H]$^+$; found 324.1036.

*Methyl 6-(4-chlorophenyl)-4-oxo-2-phenyl-1,4-dihydropyridine-3-carboxylate (**2f**).* Compound **2f** was prepared following the general procedure **H** from isoxazole **1f** (50 mg, 0.14 mmol), $Mo(CO)_6$ (19 mg, 0.07 mmol) in acetonitrile (2 mL) in 35 mg (74% yield) after column chromatography on silica (light petroleum/ethyl acetate, 8:1 + 5% chloroform, v/v) as a colorless solid: mp 68-70 °C (light petroleum/ethyl acetate). ^{1}H NMR ($CDCl_3$, 400 MHz): δ 3.59 (s, 3H), 7.30 (s, 1H), 7.41-7.45 (m, 5H),

7.50-7.52 (m, 2H), 8.01-8.04 (m, 2H), 11.17 (br. s, 1H). ^{13}C NMR (CDCl$_3$, 101 MHz): δ 52.1 (CH$_3$), 106.9 (CH), 127.7 (CH), 128.5 (CH), 128.6 (CH), 128.7 (CH), 128.9 (CH), 133.6 (C), 136.2 (C), 136.4 (C), 141.6 (C), 158.4 (C), 162.2 (C), 168.4 (C), 171.0 (C). HRMS (ESI) m/z: 340.0735 calcd for $C_{19}H_{15}ClNO_3^+$ [M + H]$^+$; found 340.0742.

*Methyl 6-(4-chlorophenyl)-4-oxo-2-(p-tolyl)-1,4-dihydropyridine-3-carboxylate (**2g**).* Compound **2g** was prepared following the general procedure **H** from isoxazole **1g** (450 mg, 1.21 mmol), Mo(CO)$_6$ (160 mg, 0.61 mmol) in acetonitrile (12 mL) in 252 mg (59% yield) after column chromatography on silica (light petroleum/ethyl acetate, 8:1 + 5% chloroform, v/v) as a colorless solid: mp 146-149 °C (light petroleum/ethyl acetate). ^{1}H NMR (CDCl$_3$, 400 MHz): δ 2.42 (s, 3H), 3.62 (s, 3H), 7.22-7.24 (m, 2H), 7.27-7.28 (m, 2H), 7.41-7.43 (m, 3H), 7.98-8.04 (m, 2H), 11.10 (br. s, 1H). ^{13}C NMR (CDCl$_3$, 101 MHz): δ 21.3 (CH$_3$), 52.1 (CH$_3$), 126.6 (C), 128.4 (CH), 128.61 (CH), 128.64 (CH), 128.9 (CH), 129.2 (CH), 135.1 (C), 136.4 (C), 138.5 (C), 144.5 (C), 158.1 (C), 168.5 (C), 171.0 (C), 171.4 (C). HRMS (ESI) m/z: 376.0711 calcd for $C_{20}H_{16}ClNO_3Na^+$ [M + Na]$^+$; found 376.0716.

*Methyl 6-(4-bromophenyl)-4-oxo-2-phenyl-1,4-dihydropyridine-3-carboxylate (**2h**).* Compound **2h** was prepared following the general procedure **H** from isoxazole **1h** (65 mg, 0.16 mmol), Mo(CO)$_6$ (31 mg, 0.12 mmol) in acetonitrile (2 mL) in 28 mg (45% yield) after column chromatography on silica (light petroleum/ethyl acetate, 8:1, v/v) as a colorless solid: mp 65-67 °C (light petroleum/ethyl acetate). ^{1}H NMR (CDCl$_3$, 400 MHz): δ 3.59 (s, 3H), 7.31 (s, 1H), 7.42-7.44 (m, 3H), 7.50-7.52 (m, 2H), 7.57-7.60 (m, 2H), 7.95-7.97 (m, 2H), 11.16 (s, 1H). ^{13}C NMR (CDCl$_3$, 101 MHz): δ 52.1 (CH$_3$), 106.8 (CH), 124.6 (C), 127.7 (CH), 128.5 (CH), 128.6 (CH), 128.9 (CH), 131.9 (CH), 136.8 (C), 141.6 (C), 158.5 (C), 162.2 (C), 168.4 (C), 170.00 (C), 171.00 (C). HRMS (ESI) m/z: 384.0236 calcd for $C_{19}H_{15}BrNO_3^+$ [M + H]$^+$; found 384.0230.

*Methyl 6-(4-bromophenyl)-2-methyl-4-oxo-1,4-dihydropyridine-3-carboxylate (**2i**).* Compound **2i** was prepared following the general procedure **H** from isoxazole **1i** (160 mg, 0.47 mmol), Mo(CO)$_6$ 69 mg, 0.26 mmol) in acetonitrile (5 mL) in 67 mg (44% yield) after column chromatography on silica (light petroleum/ethyl acetate, 10:1, v/v) as a colorless solid: mp 65-66 °C (light petroleum/ethyl acetate). ^{1}H NMR (CDCl$_3$, 400 MHz): δ 2.83 (s, 3H), 4.02 (s, 3H), 7.17 (s, 1H), 7.58-7.61 (m, 2H), 7.90-7.93 (m, 2H), 11.88 (s, 1H). ^{13}C NMR (CDCl$_3$, 101 MHz): δ 27.4 (CH$_3$), 52.6 (CH$_3$), 106.9 (CH), 108.1 (C), 124.4 (C), 128.8 (CH), 131.9 (CH), 137.0 (C), 158.6 (C), 161.8 (C), 169.5 (C), 171.6 (C). HRMS (ESI) m/z: 322.0073 calcd for $C_{14}H_{13}BrNO_3^+$ [M + H]$^+$; found 322.0072.

*Methyl 6-benzyl-4-oxo-2-(p-tolyl)-1,4-dihydropyridine-3-carboxylate (**2k**).* Compound **2k** was prepared following the general procedure **H** from isoxazole **1k** (321 mg, 0.92 mmol), Mo(CO)$_6$ (121 mg, 0.46 mmol) in acetonitrile (9 mL) in 141 mg (46% yield) after column chromatography on silica (light petroleum/ethyl acetate, 8:1, v/v) as a colorless solid: mp 228-230 °C (light petroleum/ethyl acetate). ^{1}H NMR (DMSO-d_6, 400 MHz): δ 2.37 (s, 3H), 3.50 (s, 3H), 3.90 (s, 2H), 5.97 (s, 1H), 7.29-7.38 (m, 9H), 11.66 (s, 1H). ^{13}C NMR (DMSO-d_6, 101 MHz): δ 20.9 (CH$_3$), 37.7 (CH$_2$), 51.7 (CH$_3$), 115.4 (CH), 121.7 (C), 126.8 (CH), 127.9 (CH), 128.6 (CH), 128.9 (CH), 129.2 (CH), 130.0 (C), 137.4 (C), 140.0 (C), 146.9

(C), 151.1 (C), 167.0 (C), 174.9 (C). HRMS (ESI) m/z: 334.1438 calcd for $C_{21}H_{20}NO_3^+$ [M + H]$^+$; found 334.1443.

*Methyl 4-oxo-6-phenyl-5-(thiophen-3-yl)-2-(p-tolyl)-1,4-dihydropyridine-3-carboxylate (**2n**).* Compound **2n** was prepared following the general procedure **H** from isoxazole **1n** (300 mg, 0.72 mmol), $Mo(CO)_6$ (95 mg, 0.36 mmol) in acetonitrile (7 mL) in 138 mg (47% yield) after column chromatography on silica (light petroleum/ethyl acetate, 8:1 + 5% chloroform, v/v) as a beige solid: mp 253-255 °C (light petroleum/ethyl acetate). ^{1}H NMR (DMSO-d_6, 400 MHz): δ 2.39 (s, 3H), 3.56 (s, 3H), 6.70-6.71 (m, 1H), 7.18-7.19 (m, 1H), 7.27-7.40 (m, 8H), 7.45-7.47 (m, 2H), 11.83 (s, 1H). ^{13}C NMR (DMSO-d_6, 101 MHz): δ 20.9 (CH_3), 51.7 (CH_3), 122.0 (C), 122.3 (C), 123.5 (CH), 125.4 (CH), 127.9 (CH), 128.3 (CH), 128.9 (CH), 129.0 (CH), 129.6 (CH), 129.8 (C), 129.9 (CH), 133.7 (C), 134.2 (C), 139.7 (C), 146.0 (C), 147.3 (C), 167.0 (C), 173.2 (C). HRMS (ESI) m/z: 402.1158 calcd for $C_{24}H_{20}NO_3S^+$ [M + H]$^+$; found 402.1160.

*Methyl 6-(4-(tert-butyl)phenyl)-4-oxo-2-(p-tolyl)-5-(4-(trifluoromethyl)phenyl)-1,4-dihydropyridine-3-carboxylate (**2o**).* Compound **2o** was prepared following the general procedure **H** from isoxazole **1o** (50 mg, 0.09 mmol), $Mo(CO)_6$ (12 mg, 0.05 mmol) in acetonitrile (3 mL) in 13 mg (27% yield) after column chromatography on silica (light petroleum/ethyl acetate, 8:1 + 5% chloroform, v/v) as a colorless solid: mp 285-287 °C (light petroleum/ethyl acetate). ^{1}H NMR (DMSO-d_6, 400 MHz): δ 1.23 (s, 9H), 2.38 (s, 3H), 3.56 (s, 3H), 7.24-7.34 (m, 8H), 7.46-7.48 (m, 2H), 7.53-7.55 (m, 2H), 11.89 (s, 1H). ^{13}C NMR (DMSO-d_6, 101 MHz): δ 20.9 (CH_3), 30.9 (CH_3), 34.4 (C), 51.7 (CH_3), 122.2 (C), 124.0 (q, CH, J = 3.5 Hz), 124.7 (CH), 125.7 (C), 125.8 (C), 128.3 (CH), 128.9 (CH), 129.6 (CH), 129.7 (C), 130.6 (C), 132.0 (CH), 139.3 (C), 139.8 (C), 143.7 (C), 146.7 (C), 147.7 (C), 151.8 (C), 166.9 (C), 173.0 (C). HRMS (ESI) m/z: 520.2094 calcd for $C_{31}H_{19}F_3NO_3^+$ [M + H]$^+$; found 520.2100.

*Methyl 6-(4-chlorophenyl)-4-oxo-5-phenyl-2-(p-tolyl)-1,4-dihydropyridine-3-carboxylate (**2p**).* Compound **2p** was prepared following the general procedure **H** from isoxazole **1p** (295 mg, 0.66 mmol), $Mo(CO)_6$ (96 mg, 0.36 mmol) in acetonitrile (7 mL) in 199 mg (70% yield) after column chromatography on silica (light petroleum/ethyl acetate, 10-4:1 + 5% chloroform, v/v) as a beige solid: mp 222-224 °C (light petroleum/ethyl acetate). ^{1}H NMR ($CDCl_3$, 400 MHz): δ 2.42 (s, 3H), 3.65 (s, 3H), 7.14-7.16 (m, 2H), 7.21-7.25 (m, 4H), 7.28-7.34 (m, 5H), 7.44-7.46 (m, 2H), 11.27 (s, 1H). ^{13}C NMR ($CDCl_3$, 101 MHz): δ 21.4 (CH_3), 52.3 (CH_3), 126.5 (C), 127.7 (CH), 128.0 (CH), 128.4 (CH), 128.6 (CH), 128.6 (CH), 130.9 (CH), 131.5 (CH), 134.0 (C), 134.5 (C), 137.8 (C), 138.6 (C), 144.5 (C), 154.2 (C), 154.8 (C), 164.6 (C), 168.5 (C), 170.6 (C). HRMS (ESI) m/z: 430.1205 calcd for $C_{26}H_{21}ClNO_3^+$ [M + H]$^+$; found 430.1210.

*Methyl 6-(4-chlorophenyl)-5-(4-methoxyphenyl)-4-oxo-2-(p-tolyl)-1,4-dihydropyridine-3-carboxylate (**2q**).* Compound **2q** was prepared following the general procedure **H** from isoxazole **1q** (36 mg, 0.076 mmol), $Mo(CO)_6$ (10 mg, 0.038 mmol) in acetonitrile (1.5 mL) in 14 mg (40% yield) after column chromatography on silica (light petroleum/ethyl acetate, 10:1 + 5% chloroform, v/v) as a colorless solid: mp 218-220 °C (light petroleum/ethyl acetate). ^{1}H NMR ($CDCl_3$, 400 MHz): δ 2.42 (s, 3H), 3.64 (s, 3H), 3.82 (s, 3H), 6.87-6.89 (m, 2H), 7.13-7.17 (m, 4H), 7.22-7.24 (m, 2H), 7.32-7.34 (m, 2H), 7.43-7.45 (m, 2H), 11.23 (s, 1H). ^{13}C NMR ($CDCl_3$, 101 MHz): δ 21.3 (CH_3), 52.2 (CH_3), 55.2 (CH_3), 107.7 (C), 113.9

(CH), 121.9 (C), 125.9 (C), 127.9 (CH), 128.5 (CH), 128.6 (CH), 131.5 (CH), 132.0 (CH), 134.3 (C), 138.1 (C), 138.4 (C), 138.6 (C), 158.3 (C), 159.0 (C), 160.0 (C), 165.4 (C), 171.6 (C). HRMS (ESI) m/z: 460.1310 calcd for $C_{27}H_{23}ClNO_4^+$ [M + H]$^+$; found 460.1314.

*Methyl 2-(4-bromophenyl)-6-(4'-fluoro-[1,1'-biphenyl]-4-yl)-5-(4-fluorophenyl)-4-oxo-1,4-dihydropyridine-3-carboxylate (**2s**).* Compound **2s** was prepared following the general procedure **H** from isoxazole **1s** (40 mg, 0.068 mmol), $Mo(CO)_6$ (9 mg, 0.034 mmol) in acetonitrile (2 mL) in 22 mg (58% yield) after column chromatography on silica (light petroleum/ethyl acetate, 8:1 + 5% chloroform, v/v) as a brown solid: mp 193-195 °C (light petroleum/ethyl acetate). ^{1}H NMR ($CDCl_3$, 400 MHz): δ 3.67 (s, 3H), 7.03-7.12 (m, 4H), 7.37-7.45 (m, 4H), 7.48-7.53 (m, 2H), 7.56-7.58 (m, 2H), 11.54 (s, 1H). ^{13}C NMR ($CDCl_3$, 101 MHz): δ 52.4 (CH_3), 107.3 (C), 115.4 (d, CH, J = 21.4 Hz), 115.7 (d, CH, J = 20.9 Hz), 121.8 (C), 122.9 (C), 126.4 (CH), 128.6 (d, CH, J = 8.3 Hz), 129.9 (d, C, J = 3.4 Hz), 130.4 (CH), 130.7 (CH), 131.0 (CH), 132.7 (d, CH, J = 7.9 Hz), 136.5 (d, C, J = 3.2 Hz), 138.1 (C), 140.2 (C), 140.6 (C), 159.4 (C), 159.5 (C), 162.2 (d, C, J = 247.5 Hz), 162.6 (d, C, J = 246.6 Hz), 166.0 (C), 171.3 (C). HRMS (ESI) m/z: 572.0667 calcd for $C_{31}H_{21}BrF_2NO_3^+$ [M + H]$^+$; found 572.0675.

*Methyl 5-amino-5-(4-chlorophenyl)-3-oxopent-4-enoate (**6**).* Compound **6** was prepared following the general procedure **H** from isoxazole **1g** (70 mg, 0.20 mmol), $Mo(CO)_6$ (31 mg, 0.12 mmol) in acetonitrile (3 mL) in 5 mg (10% yield) after column chromatography on silica (light petroleum/ethyl acetate, 8:1-3:1) as a light yellow oil. ^{1}H NMR ($CDCl_3$, 400 MHz): δ 3.46 (s, 2H), 3.74 (s, 3H), 5.35 (br. s, 1H), 5.45 (s, 1H), 7.40-7.43 (m, 2H), 7.47-7.51 (m, 2H), 9.91 (br. s, 1H). ^{13}C NMR ($CDCl_3$, 101 MHz): δ 48.9 (CH_2), 52.2 (CH_3), 94.3 (CH), 127.7 (CH), 129.3 (CH), 135.1 (C), 137.1 (C), 161.3 (C), 169.1 (C), 190.3 (C). HRMS (ESI) m/z: 254.0579 calcd for $C_{12}H_{13}ClNO_3^+$ [M + H]$^+$; found 254.0586.

*2-(4-Bromophenyl)-6-(4-(tert-butyl)phenyl)-3-(hydroxymethyl)pyridin-4(1H)-one (**14**).* Methyl 4-oxo-6-aryl-1,4-dihydropyridine-3-carboxylate **2c** (115 mg, 0.26 mmol) was dissolved in dry THF (5 mL) cooled to 0 °C (ice-water bath temperature) and then $LiAlH_4$ (32 mg, 0.84 mmol) was added portionwise. The reaction mixture was stirred at rt for 1 h, treated with sat. aq. NH_4Cl and the product was extracted with ethyl acetate. The organic layers were dried over Na_2SO_4, filtrated, and after evaporation of the solvent the residue was washed with hexanes/ether (1:1, v/v) and dried on air to give pure product after evaporation of solvents in 95 mg (89% yield) as a colorless solid: mp 308-310 °C (ethyl acetate). ^{1}H NMR (DMSO-d_6, 400 MHz): δ 1.32 (s, 9H), 4.42 (s, 2H), 5.06 (s, 1H), 7.31 (s, 1H), 7.50-7.54 (m, 2H), 7.67-7.69 (m, 2H), 7.76-7.78 (m, 2H), 7.90-7.92 (m, 2H), 10.82 (s, 1H). ^{13}C NMR (DMSO-d_6, 101 MHz): δ 31.0 (CH_3), 34.4 (C), 54.9 (CH_2), 105.9 (CH), 120.0 (C), 121.6 (C), 125.4 (CH), 126.2 (CH), 130.7 (CH), 131.4 (CH), 136.0 (C), 139.5 (C), 151.5 (C), 155.8 (C), 158.3 (C), 164.4 (C). HRMS (ESI) m/z: 412.0907 calcd for $C_{22}H_{23}BrNO_2^+$ [M + H]$^+$; found 412.0899.

*Methyl 4-bromo-6-(4-fluorophenyl)-2-phenylnicotinate (**15**).* To a solution of 4-oxo-6-aryl-1,4-dihydropyridine-3-carboxylate **2e** (110 mg, 0.34 mmol) in DMF (1 mL) PBr_3(0.15 mL, 1.58 mmol) was added at 0 ºC (ice-water bath). The reaction mixture was then stirred at room temperature for 3 d. The reaction was diluted with cold water, basified with sat. aq. $NaHCO_3$, and extracted with ethyl acetate. The

solvent was evaporated and the residue was purified by column chromatography on silica with light petroleum/ethyl acetate (15:1, v/v) as an eluent to give pure product after evaporation of solvents in 105 mg (80% yield) as a colorless oil. ^{1}H NMR ($CDCl_3$, 400 MHz): δ 3.77 (s, 3H), 7.14-7.20 (m, 2H), 7.44-7.48 (m, 3H), 7.69-7.72 (m, 2H), 7.91 (s, 1H), 8.05-8.11 (m, 2H). ^{13}C NMR ($CDCl_3$, 101 MHz): δ 52.8 (CH_3), 115.9 (d, CH, J = 21.6 Hz), 121.9 (CH), 128.4 (CH), 128.5 (CH), 128.8 (C), 129.2 (d, CH, J = 8.5 Hz), 129.4 (CH), 131.7 (C), 133.4 (d, C, J = 3.1 Hz), 138.9 (C), 157.0 (C), 157.3 (C), 164.2 (d, C, J = 250.4 Hz), 167.6 (C). HRMS (ESI) m/z: 386.0186 calcd for $C_{19}H_{14}BrNO_2^+$ $[M + H]^+$; found 386.0180.

*Methyl 4-(4-chlorophenyl)-6-(4-fluorophenyl)-2-phenylnicotinate (**16**).* Methyl 4-bromonicotinate **15** (87 mg, 0.23 mmol), (4-chlorophenyl)boronic acid (53 mg, 0.54 mmol), K_2CO_3 (63 mg, 0.45 mmol) and Pd(dppf)Cl_2 (8.3 mg, 0.011 mmol) in dioxane (6 mL) and water (2 mL) was stirred at 100 °C (oil bath temperature) for 2 h, diluted with water and extracted with ethyl acetate. The solvent was evaporated and the residue was purified by column chromatography on silica with light petroleum/ethyl acetate (20:1 , v/v) as an eluent to give pure product after evaporation of solvents in 90 mg (96% yield) as a light yellow oil. ^{1}H NMR ($CDCl_3$, 400 MHz): δ 3.51 (s, 3H), 7.14-7.20 (m, 2H), 7.38-7.43(m, 2H), 7.44-7.49 (m, 5H), 7.63 (s, 1H), 7.70-7.73 (m, 2H), 8.10-8.15 (m, 2H). ^{13}C NMR ($CDCl_3$, 101 MHz): δ 52.3 (CH_3), 115.8 (d, CH, J = 21.8 Hz), 118.7 (CH), 126.3 (C), 128.4 (CH), 128.5 (CH), 128.9 (CH), 129.0 (CH), 129.1 (d, CH, J = 8.5 Hz), 129.4 (CH), 134.4 (d, C, J = 3.1 Hz), 135.0 (C), 136.8 (C), 139.7 (C), 148.5 (C), 156.4 (C), 156.8 (C), 163.0 (d, C, J = 249.7 Hz), 169.0 (C). HRMS (ESI) m/z: 418.1009 calcd for $C_{25}H_{18}ClFNO_2^+$ $[M + H]^+$; found 418.1009.

*Methyl 1-(4-chlorobenzyl)-4-oxo-6-phenyl-5-(thiophen-3-yl)-2-(p-tolyl)-1,4-dihydropyridine-3-carboxylate (**17**).* To a solution of 4-oxo-6-aryl-1,4-dihydropyridine-3-carboxylate **2n** (31 mg, 0.077 mmol) and 4-chlorobenzyl chloride (24 mg, 0.116 mmol) in DMF (0.5 mL) K_2CO_3 (22 mg, 0.154 mmol) and NaI (2 mg, 0.013 mmol) were added. The reaction mixture stirred at rt for 24 h and the precipitate was filtered off. The solvent was evaporated, and the residue was purified by column chromatography on silica with light petroleum/ethyl acetate (10:1, v/v) as an eluent to give pure product after evaporation of solvents in 39 mg (98% yield) as a colorless solid: mp 176-178 °C (light petroleum/ethyl acetate). ^{1}H NMR ($CDCl_3$, 400 MHz): δ 2.40 (s, 3H), 3.71 (s, 3H), 4.53 (s, 2H), 6.91-6.93 (m, 1H), 7.02-7.04 (m, 2H), 7.18-7.20 (s, 1H), 7.22 (m, 8H), 7.37-7.39 (m, 2H), 7.65-7.67 (m, 2H). ^{13}C NMR ($CDCl_3$, 101 MHz): δ 21.3 (CH_3), 52.6 (CH_3), 75.2 (CH_2), 122.5 (C), 123.2 (C), 125.3 (CH), 125.8 (CH), 127.8 (CH), 128.1 (CH), 128.3 (CH), 128.5 (CH), 129.2 (CH), 129.66 (CH), 129.71 (CH), 129.9 (CH), 133.6 (C), 134.2 (C), 134.6 (C), 136.3 (C), 139.1 (C), 139.7 (C), 156.2 (C), 160.2 (C), 161.9 (C), 167.9 (C). HRMS (ESI) m/z: 526.1238 calcd for $C_{31}H_{25}ClNO_3S^+$ $[M + H]^+$; found 526.1243.

References

1. Chen, H.-L.; Wei, D.; Zhang, J.-W.; Li, C.-L.; Yu, W.; Han, B. *Org. Lett.* **2018**, *20*, 2906−2910.
2. Serebryannikova, A. V.; Galenko, E. E.; Novikov, M. S.; Khlebnikov, A. F. *Tetrahedron* **2021**, *77*, 132153.

3. Leivers, M. R.; Keicher, J. D.; Schmitz, F. U.; Rai R.; Lauchli, R.; Liehr, S. R. J.; Chan, S. A.; Ton, T. L. WO Patent 2009011787, January 22, **2009**.
4. Chiarino, D.; Napoletano, M.; Sala, A. *Synth. Commun*. **1988**, *18*, 1171-1176.
5. Dowd, P.; Wilk, B. K.; Wlostowski, M. A. *Synth. Commun*. **1993**, *23*, 2323-2329.
6. Allegretti, M.; Bertini, R.; Cesta, M. C.; Bizzarri, C.; Bitondo, R. D.; Cioccio, V. D.; Galliera, E.; Berdini, V.; Topai, A.; Zampella, G.; Russo, V.; Bello, N. D.; Nano, G.; Nicolini, L.; Locati, M.; Fantucci, P.; Florio, S.; Colotta, F. *J. Med. Chem*. **2005**, *48*, 4312-4331.
7. Galenko, E. E.; Ivanov, V. K.; Novikov, M. S.; Khlebnikov, A. F. *Tetrahedron* **2018**, *74*, 6288-6298.

X-ray diffraction experiments

Crystal structure of **2k** was determined by single crystal X-ray diffraction analysis. Suitable crystals were selected and fixed on micro-amounts and the diffraction data were collected on diffractometer. The crystals **2k** were measured at temperature 100 K, using monochromated CuKα radiation. The unit cell parameters and refinement characteristics of the crystal structure of **2k** is given below. Using Olex2[1], the structure was solved with the ShelXT [2] structure solution program using Intrinsic Phasing and refined with the ShelXL [3] refinement package using least squares minimization.

References

1. Dolomanov, O. V.; Bourhis, L. J.; Gildea, R. J.; Howard, J .A. K.; Puschmann, H. *J. Appl. Cryst.* **2009**, *42*, 339.

2. Sheldrick, G. M. *Acta Cryst*. **2015**, *A71*, 3.

3. Sheldrick, G. M. *Acta Cryst*. **2015**, *C71*, 3.

Methyl 6-benzyl-4-oxo-2-(*p*-tolyl)-1,4-dihydropyridine-3-carboxylate 2k

Single crystals of **2k** were obtained by slow recrystallization from ethyl acetate at room temperature (CCDC 2154972)

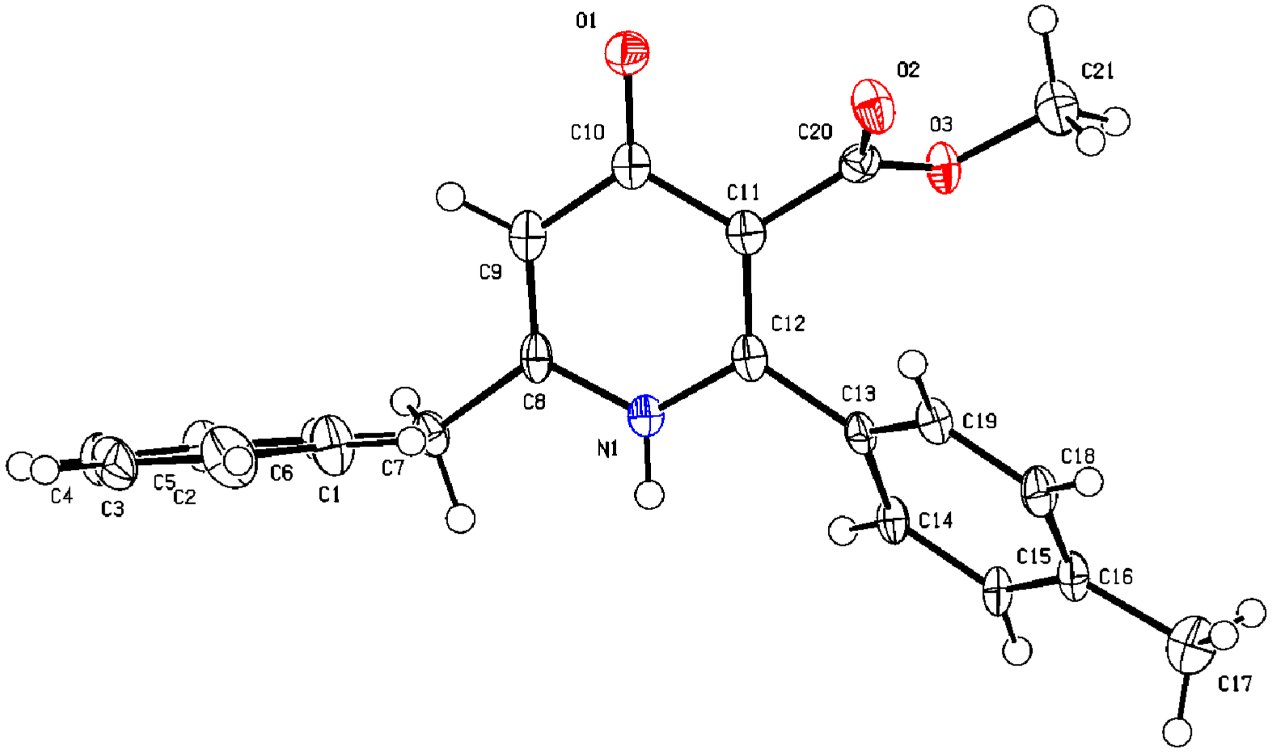

Figure S1. Molecular structure of compound **2k**, displacement parameters are drawn at 50% probability level.

Table 1. Crystal data and structure refinement for 2k.

Identification code	**2k** (TZ_247)
Empirical formula	$C_{21}H_{19}NO_3$
Formula weight	333.37
Temperature/K	99.9(8)
Crystal system	orthorhombic
Space group	$P2_12_12_1$
a/Å	10.3185(4)
b/Å	12.3484(4)
c/Å	13.2278(5)
α/°	90
β/°	90
γ/°	90
Volume/Å^3	1685.45(11)
Z	4
ρ_{calc}g/cm^3	1.314
μ/mm^{-1}	0.708
F(000)	704.0
Crystal size/mm^3	0.28 × 0.16 × 0.12
Radiation	CuKα (λ = 1.54184)
2Θ range for data collection/°	9.798 to 124.968
Index ranges	$-11 \leq h \leq 11$, $-14 \leq k \leq 11$, $-15 \leq l \leq 9$
Reflections collected	7447
Independent reflections	2595 [R_{int} = 0.1165, R_{sigma} = 0.1015]
Data/restraints/parameters	2595/0/228
Goodness-of-fit on F^2	1.062
Final R indexes [I>=2σ (I)]	R_1 = 0.0850, wR_2 = 0.2142
Final R indexes [all data]	R_1 = 0.1046, wR_2 = 0.2236
Largest diff. peak/hole / e Å^{-3}	0.46/-0.48
Flack parameter	-0.2(5)

Table 2. Fractional atomic coordinates (×10^4) and equivalent isotropic displacement parameters (Å^2×10^3) for 2k. U_{eq} is defined as 1/3 of of the trace of the orthogonalised U_{IJ} tensor.

Atom	*x*	*y*	*z*	U(eq)
O_1	-7275(4)	-4782(3)	-3097(2)	25.1(10)
O_3	-7058(3)	-2026(3)	-4284(3)	20.9(9)
O_2	-5421(4)	-3017(3)	-3670(3)	26.4(10)
N_1	-7930(4)	-4732(4)	-6115(3)	16.7(10)
C_{10}	-7473(5)	-4781(4)	-4033(4)	17.7(12)
C_{12}	-7265(5)	-3896(4)	-5697(4)	17.1(11)
C_{16}	-5837(5)	-1443(5)	-7703(4)	21.1(13)
C_{18}	-5051(5)	-1884(5)	-6954(4)	22.0(12)
C_{20}	-6411(5)	-2943(5)	-4156(3)	19.8(12)
C_{13}	-6779(5)	-3056(4)	-6390(4)	17.6(11)
C_{14}	-7554(5)	-2638(4)	-7154(4)	17.9(12)
C_8	-8392(5)	-5578(4)	-5564(4)	18.7(12)
C_{19}	-5494(5)	-2680(5)	-6306(4)	18.3(12)
C_{15}	-7095(5)	-1848(4)	-7807(4)	20.0(12)
C_7	-9162(5)	-6419(5)	-6133(4)	20.7(12)
C_6	-8663(5)	-7572(5)	-6076(4)	18.1(12)
C_1	-7314(6)	-7778(5)	-6092(4)	28.2(14)
C_9	-8169(5)	-5617(5)	-4552(4)	20.8(12)
C_{11}	-7063(5)	-3882(4)	-4662(4)	17.9(12)
C_4	-9058(6)	-9491(5)	-6081(4)	27.7(14)
C_5	-9494(5)	-8429(5)	-6065(4)	23.3(13)
C_3	-7721(6)	-9687(5)	-6081(4)	29.4(14)
C_2	-6881(6)	-8837(6)	-6094(4)	33.8(15)
C_{21}	-6446(6)	-1084(5)	-3859(4)	31.5(14)
C_{17}	-5353(6)	-530(6)	-8348(4)	32.3(14)

Table 3. Anisotropic displacement parameters (Å^2×10^3) for 2k. The anisotropic displacement factor exponent takes the form: -2π^2[h^{2}a*^{2}U$_{11}$+2hka*b*U$_{12}$+…].

Atom	U_{11}	U_{22}	U_{33}	U_{23}	U_{13}	U_{12}
O_1	26.8(19)	25(2)	23.2(18)	1.4(15)	-2.5(15)	-2(2)
O_3	17.4(18)	13(2)	32.4(17)	0.2(16)	-3.2(15)	0.5(18)
O_2	21.5(19)	21(2)	36.7(19)	-2.0(17)	-11.3(17)	0.4(19)
N_1	12.6(19)	16(2)	21.2(18)	0.1(17)	0.2(16)	0(2)
C_{10}	9(2)	17(3)	27(3)	0(2)	1.2(18)	4(2)
C_{12}	11(2)	13(3)	28(2)	-1(2)	0.0(19)	4(2)
C_{16}	19(3)	13(3)	32(3)	-2(2)	6(2)	-3(3)
C_{18}	15(2)	18(3)	33(3)	-2(2)	4(2)	-3(3)
C_{20}	18(2)	22(3)	19(2)	1(2)	4.7(19)	0(3)
C_{13}	19(2)	11(3)	23(2)	-3(2)	2(2)	0(3)
C_{14}	13(3)	13(3)	28(2)	0(2)	-1.5(19)	-1(2)
C_8	13(2)	11(3)	32(3)	0(2)	3(2)	2(2)
C_{19}	11(2)	16(3)	28(2)	-3(2)	-0.9(19)	0(2)
C_{15}	19(3)	11(3)	30(2)	2(2)	-2(2)	-2(3)
C_7	17(2)	19(3)	26(2)	-3(2)	0(2)	-4(3)
C_6	16(2)	13(3)	24(2)	-1(2)	3.2(19)	3(3)
C_1	14(3)	25(3)	46(3)	-3(3)	1(2)	-3(3)
C_9	14(2)	16(3)	32(3)	1(2)	2(2)	1(3)
C_{11}	13(2)	16(3)	25(2)	-1(2)	1.2(19)	5(3)
C_4	26(3)	22(3)	35(3)	-1(3)	1(2)	-6(3)
C_5	15(2)	22(3)	33(3)	0(2)	-1(2)	0(3)
C_3	36(3)	17(3)	36(3)	-5(2)	-3(3)	10(3)
C_2	20(3)	32(4)	49(3)	-9(3)	0(3)	11(3)
C_{21}	32(3)	23(4)	39(3)	-1(3)	-6(3)	-2(3)
C_{17}	26(3)	30(4)	41(3)	5(3)	1(3)	3(3)

Table 4. Bond lengths for 2k.

Atom	Atom	Length/Å	Atom	Atom	Length/Å
O_1	C_{10}	1.255(6)	C_{20}	C_{11}	1.498(8)
O_3	C_{20}	1.325(7)	C_{13}	C_{14}	1.388(7)
O_3	C_{21}	1.437(7)	C_{13}	C_{19}	1.410(7)
O_2	C_{20}	1.211(6)	C_{14}	C_{15}	1.386(7)
N_1	C_{12}	1.357(7)	C_8	C_7	1.509(8)
N_1	C_8	1.360(7)	C_8	C_9	1.360(7)
C_{10}	C_9	1.433(8)	C_7	C_6	1.515(9)
C_{10}	C_{11}	1.450(7)	C_6	C_1	1.415(8)
C_{12}	C_{13}	1.473(7)	C_6	C_5	1.363(8)
C_{12}	C_{11}	1.386(7)	C_1	C_2	1.382(10)
C_{16}	C_{18}	1.391(8)	C_4	C_5	1.386(9)
C_{16}	C_{15}	1.398(8)	C_4	C_3	1.401(9)
C_{16}	C_{17}	1.499(8)	C_3	C_2	1.361(9)
C_{18}	C_{19}	1.382(8)			

Table 5. Bond angles for 2k.

Atom	Atom	Atom	Angle/°	Atom	Atom	Atom	Angle/°
C_{20}	O_3	C_{21}	114.9(4)	N_1	C_8	C_7	116.5(4)
C_{12}	N_1	C_8	122.9(4)	C_9	C_8	N_1	119.7(5)
O_1	C_{10}	C_9	123.6(5)	C_9	C_8	C_7	123.8(5)
O_1	C_{10}	C_{11}	121.3(5)	C_{18}	C_{19}	C_{13}	119.8(5)
C_9	C_{10}	C_{11}	115.1(4)	C_{14}	C_{15}	C_{16}	120.5(5)
N_1	C_{12}	C_{13}	117.0(4)	C_8	C_7	C_6	116.3(4)
N_1	C_{12}	C_{11}	119.2(5)	C_1	C_6	C_7	120.2(5)
C_{11}	C_{12}	C_{13}	123.7(5)	C_5	C_6	C_7	121.1(4)
C_{18}	C_{16}	C_{15}	118.1(5)	C_5	C_6	C_1	118.6(5)
C_{18}	C_{16}	C_{17}	120.4(5)	C_2	C_1	C_6	119.3(6)
C_{15}	C_{16}	C_{17}	121.5(5)	C_8	C_9	C_{10}	122.0(5)
C_{19}	C_{18}	C_{16}	121.8(5)	C_{10}	C_{11}	C_{20}	117.9(4)
O_3	C_{20}	C_{11}	112.3(4)	C_{12}	C_{11}	C_{10}	120.9(5)
O_2	C_{20}	O_3	123.8(5)	C_{12}	C_{11}	C_{20}	121.3(5)
O_2	C_{20}	C_{11}	123.9(5)	C_5	C_4	C_3	118.9(6)
C_{14}	C_{13}	C_{12}	121.3(5)	C_6	C_5	C_4	122.0(5)
C_{14}	C_{13}	C_{19}	118.5(5)	C_2	C_3	C_4	119.6(6)
C_{19}	C_{13}	C_{12}	120.2(5)	C_3	C_2	C_1	121.6(5)
C_{15}	C_{14}	C_{13}	121.2(5)				

Table 6. Hydrogen atom coordinates (Å×10^4) and isotropic displacement parameters (Å^2×10^3) for 2k.

Atom	*x*	*y*	*z*	U(eq)
H_1	-8068.56	-4726.11	-6771.32	20
H_{18}	-4185.93	-1631.34	-6886.45	26
H_{14}	-8415.21	-2897.4	-7231.15	21
H_{19}	-4934.08	-2973.4	-5806.3	22
H_{15}	-7639.36	-1579.95	-8329.5	24
H_{7A}	-9195.21	-6203.45	-6853.71	25
H_{7B}	-10062.06	-6409.94	-5873.82	25
H_{1A}	-6713.35	-7195.53	-6101.47	34
H_9	-8484.08	-6217.92	-4176.46	25
H_4	-9655.84	-10075.5	-6092.18	33
H_5	-10400.6	-8295.6	-6044.56	28
H_3	-7402.1	-10408.31	-6071.74	35
H_2	-5975.32	-8975.1	-6103.75	41
H_{21A}	-5566.82	-1014.98	-4132.24	47
H_{21B}	-6403.82	-1157.66	-3122.47	47
H_{21C}	-6950.07	-438.34	-4034.16	47
H_{17A}	-5778.53	144.62	-8143.09	48
H_{17B}	-5550.38	-681.19	-9058.35	48
H_{17C}	-4413.52	-457.23	-8262.41	48

5-(Chloromethyl)-3-(quinolin-2-yl)isoxazole (8h), ^{1}H NMR, $CDCl_3$, 400 MHz

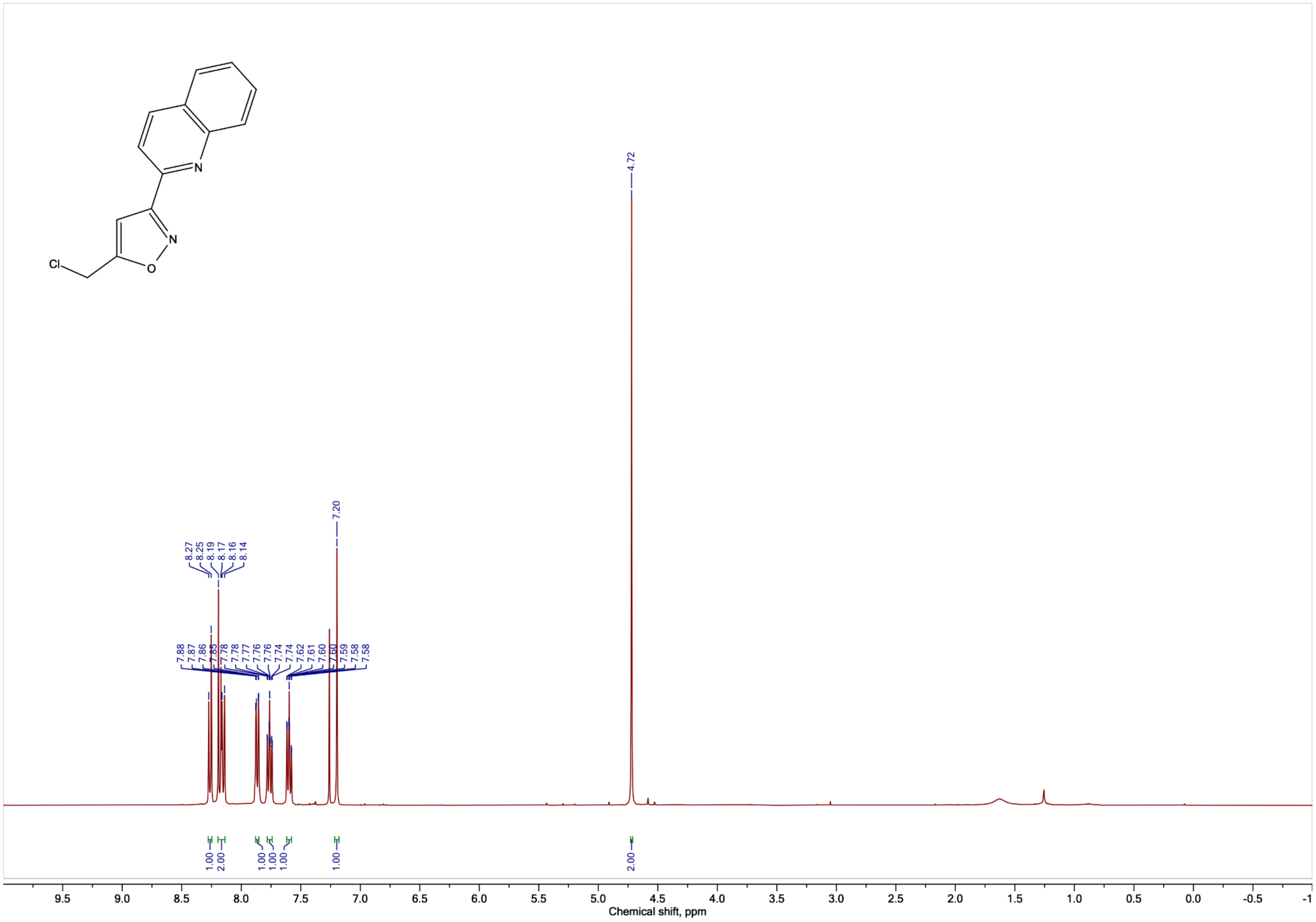

5-(Chloromethyl)-3-(quinolin-2-yl)isoxazole (8h), $^{13}C\{^1H\}$ NMR, $CDCl_3$, 101 MHz

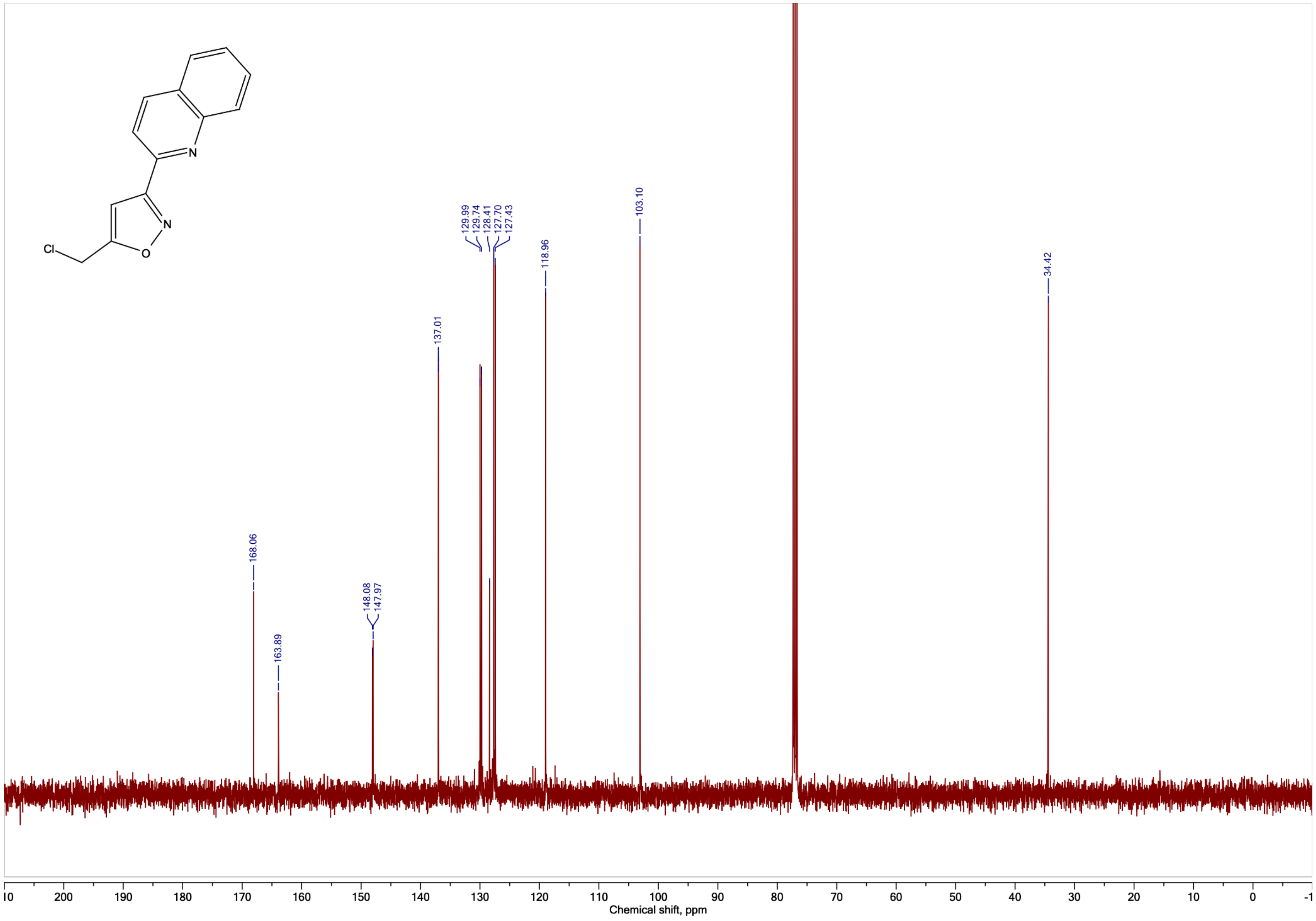

5-(Chloromethyl)-3-(quinolin-2-yl)isoxazole (8h), DEPT, $CDCl_3$, 101 MHz

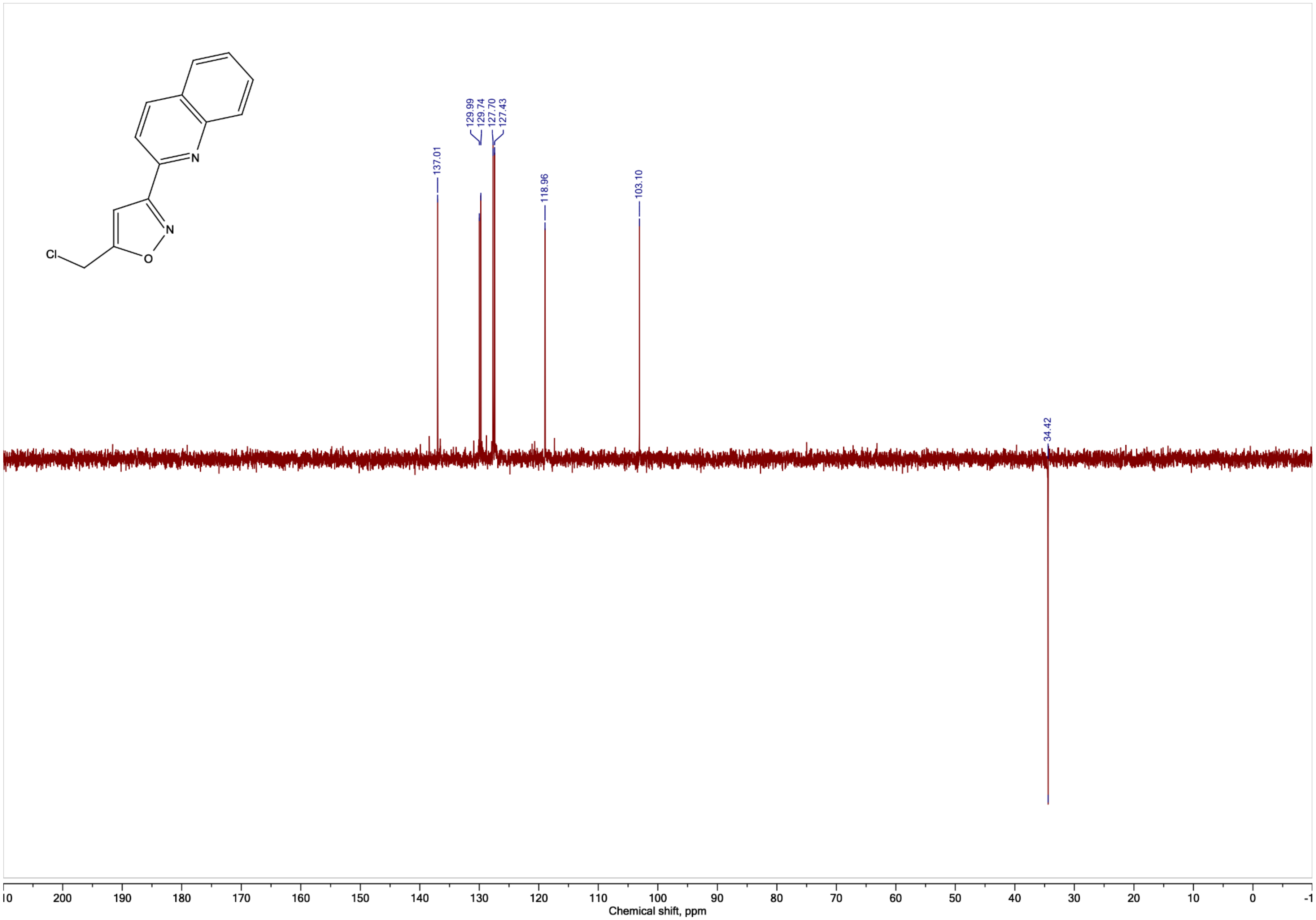

3-Benzyl-5-(chloromethyl)isoxazole (8i), ^{1}H NMR, $CDCl_3$, 400 MHz

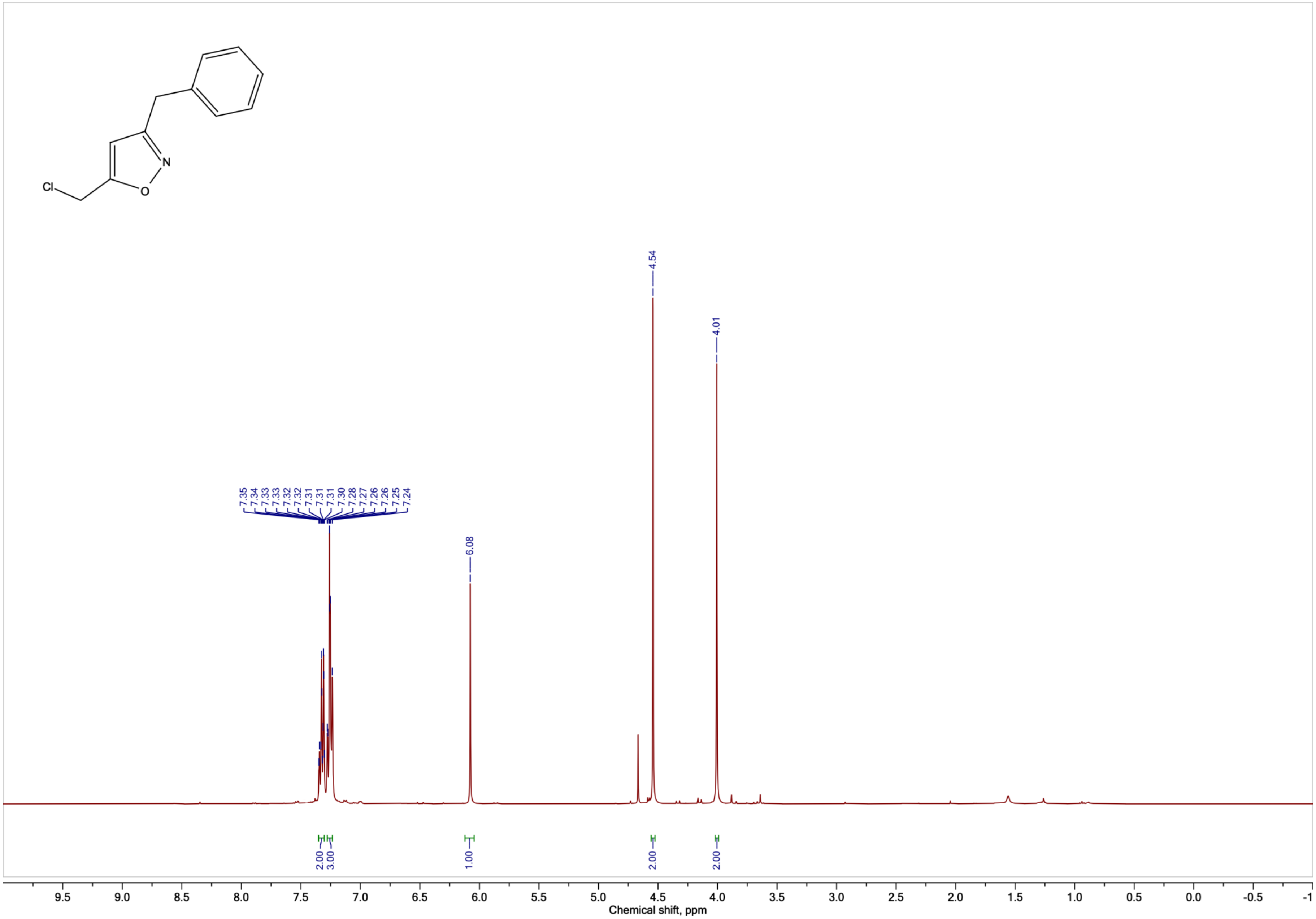

3-Benzyl-5-(chloromethyl)isoxazole (8i), $^{13}C\{^{1}H\}$ NMR, $CDCl_3$, 101 MHz

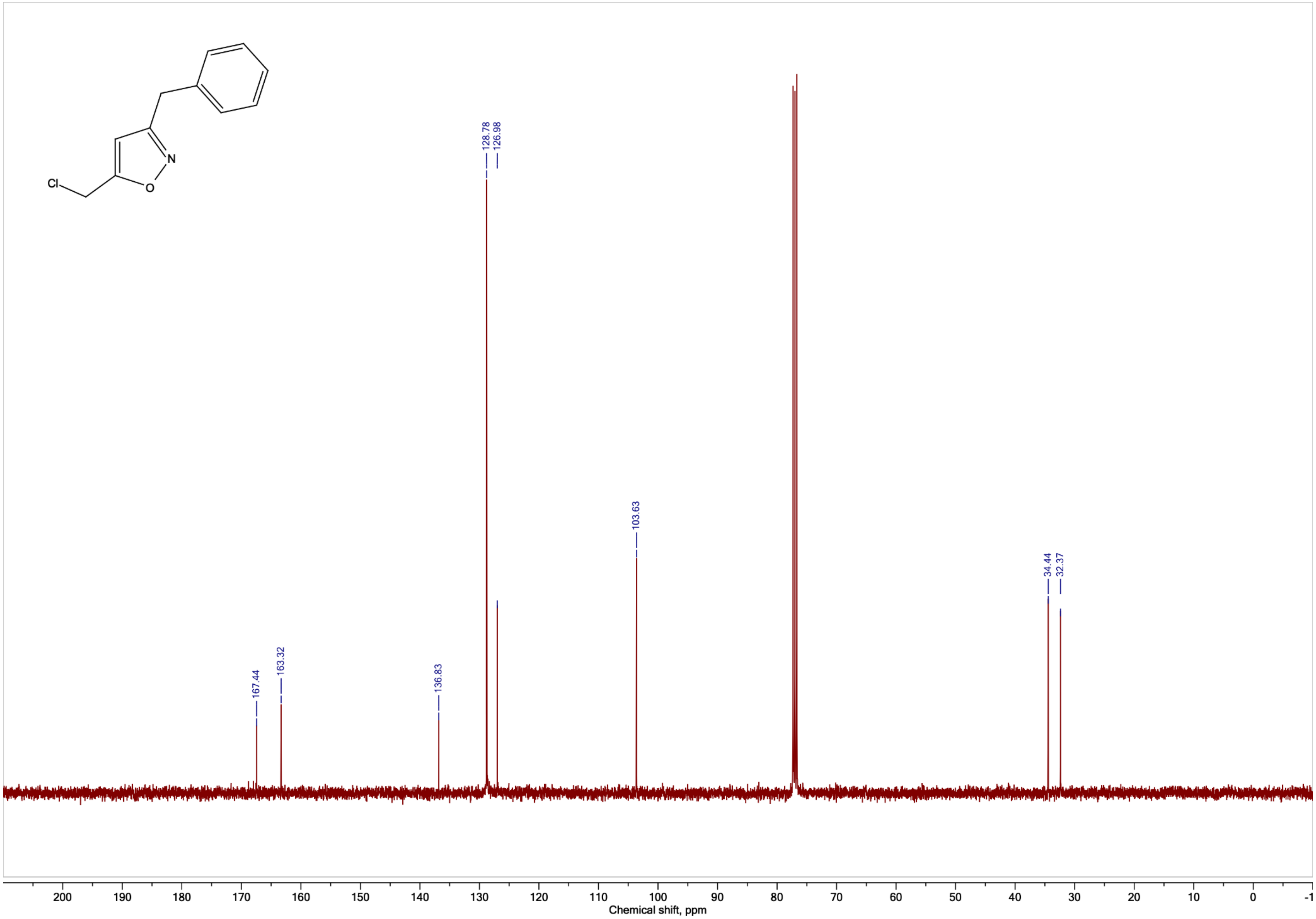

3-Benzyl-5-(chloromethyl)isoxazole (8i), DEPT, $CDCl_3$, 101 MHz

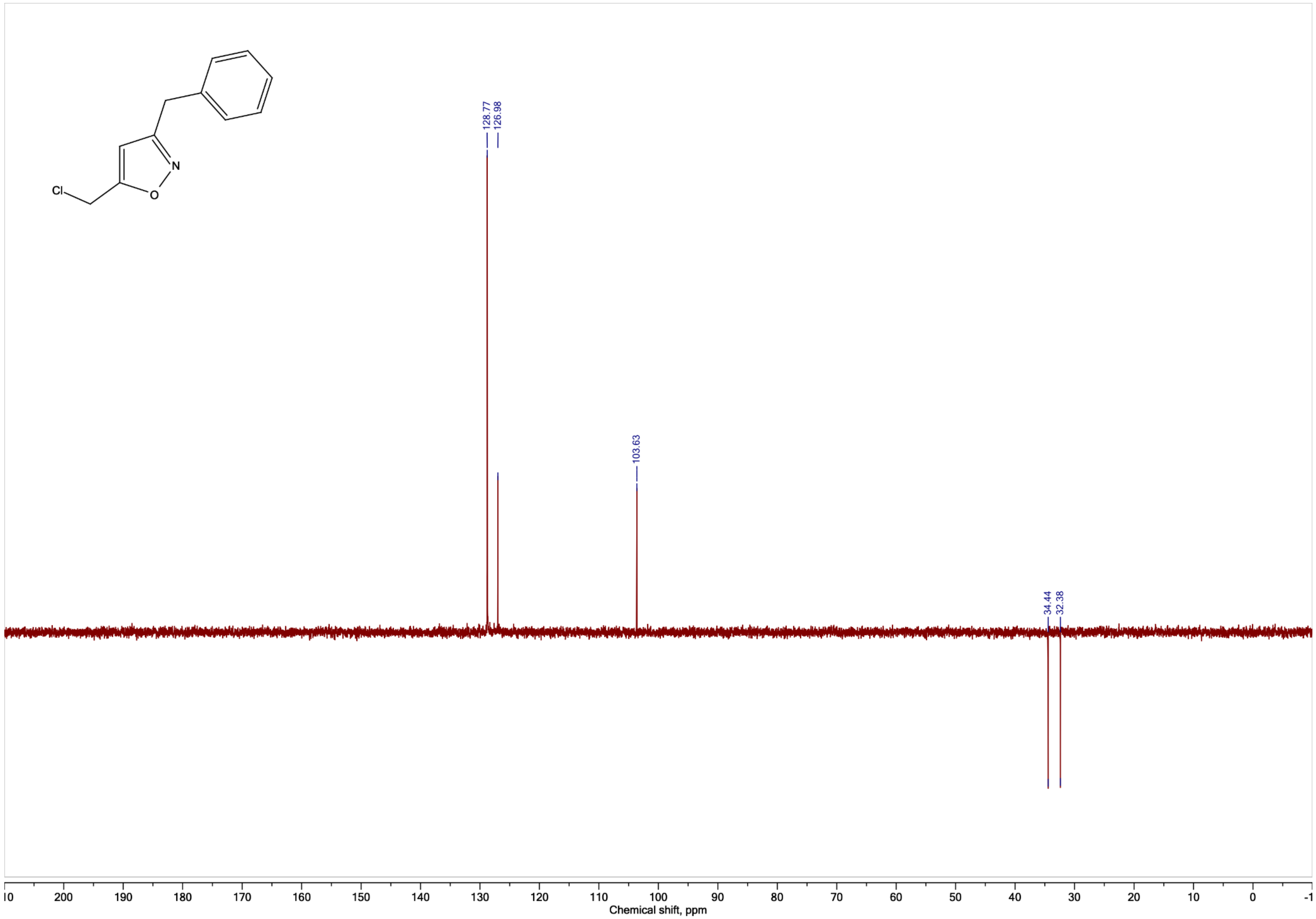

5-(Bromomethyl)-3-heptylisoxazole (8j), ^1H NMR, $CDCl_3$, 400 MHz

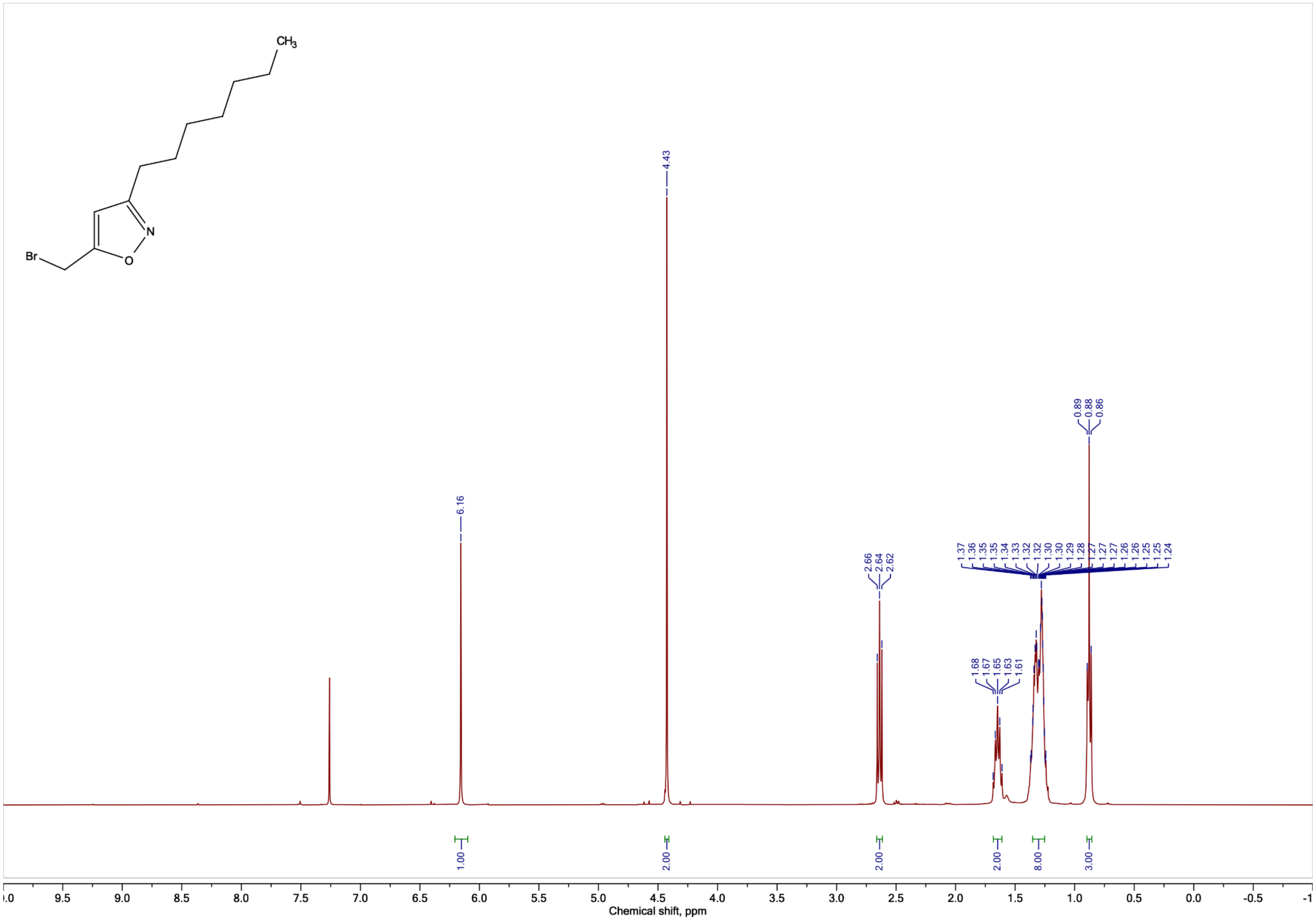

5-(Bromomethyl)-3-heptylisoxazole (8j), $^{13}C\{^{1}H\}$ NMR, $CDCl_3$, 101 MHz

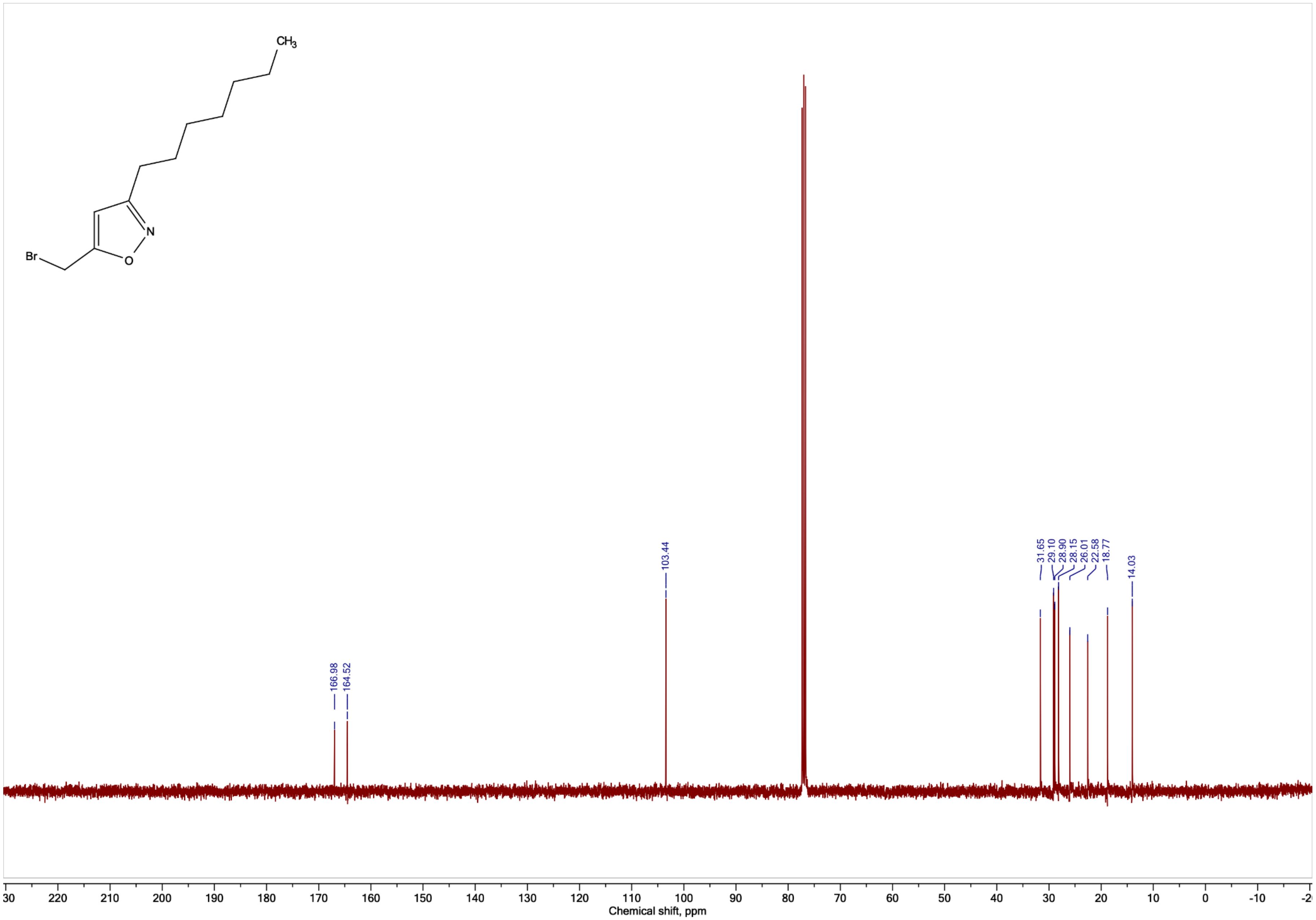

5-(Bromomethyl)-3-heptylisoxazole (8j), DEPT, $CDCl_3$, 101 MHz

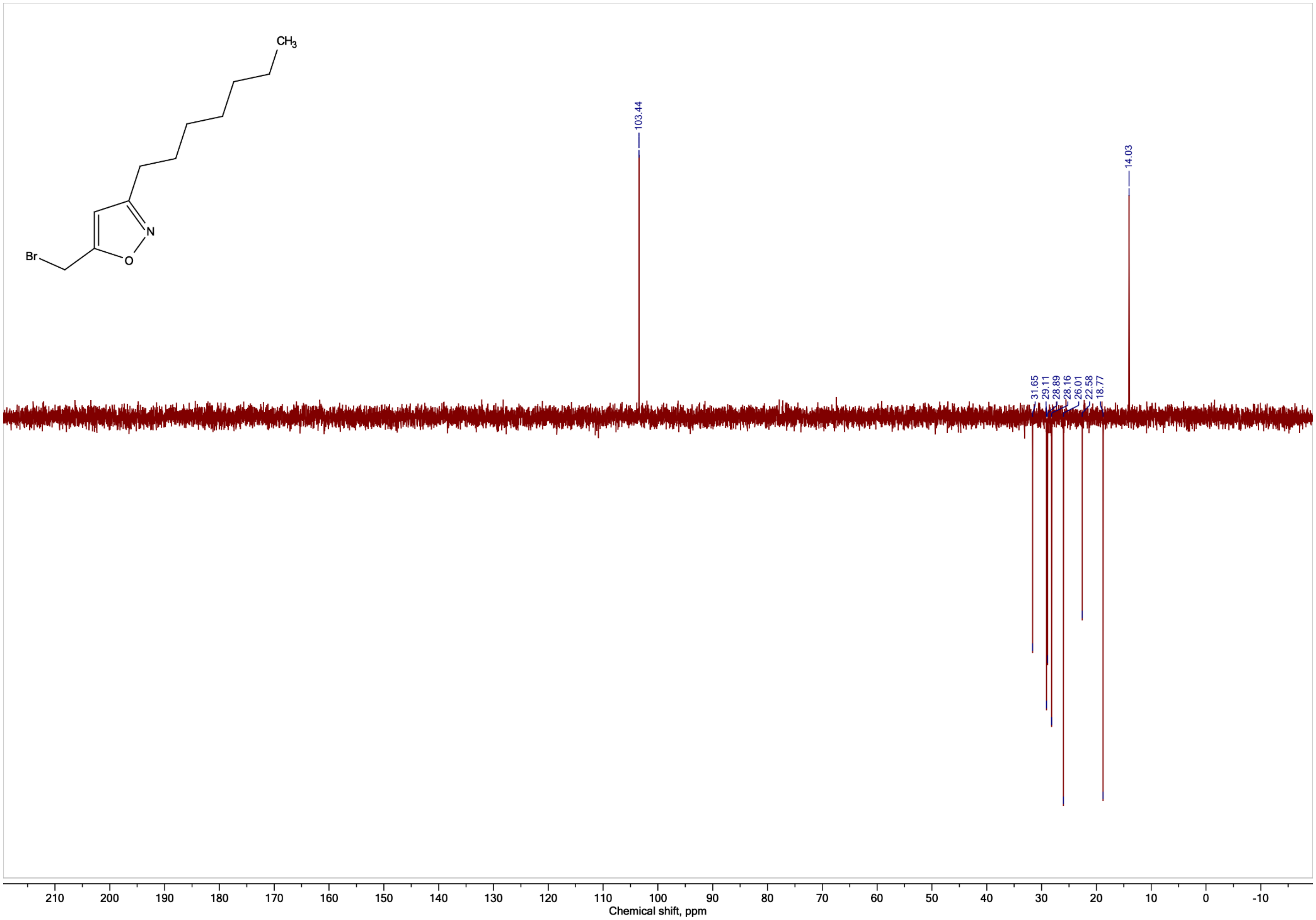

2-(3-(4-Methoxyphenyl)isoxazol-5-yl)acetonitrile (9d), ^{1}H NMR, $CDCl_3$, 400 MHz

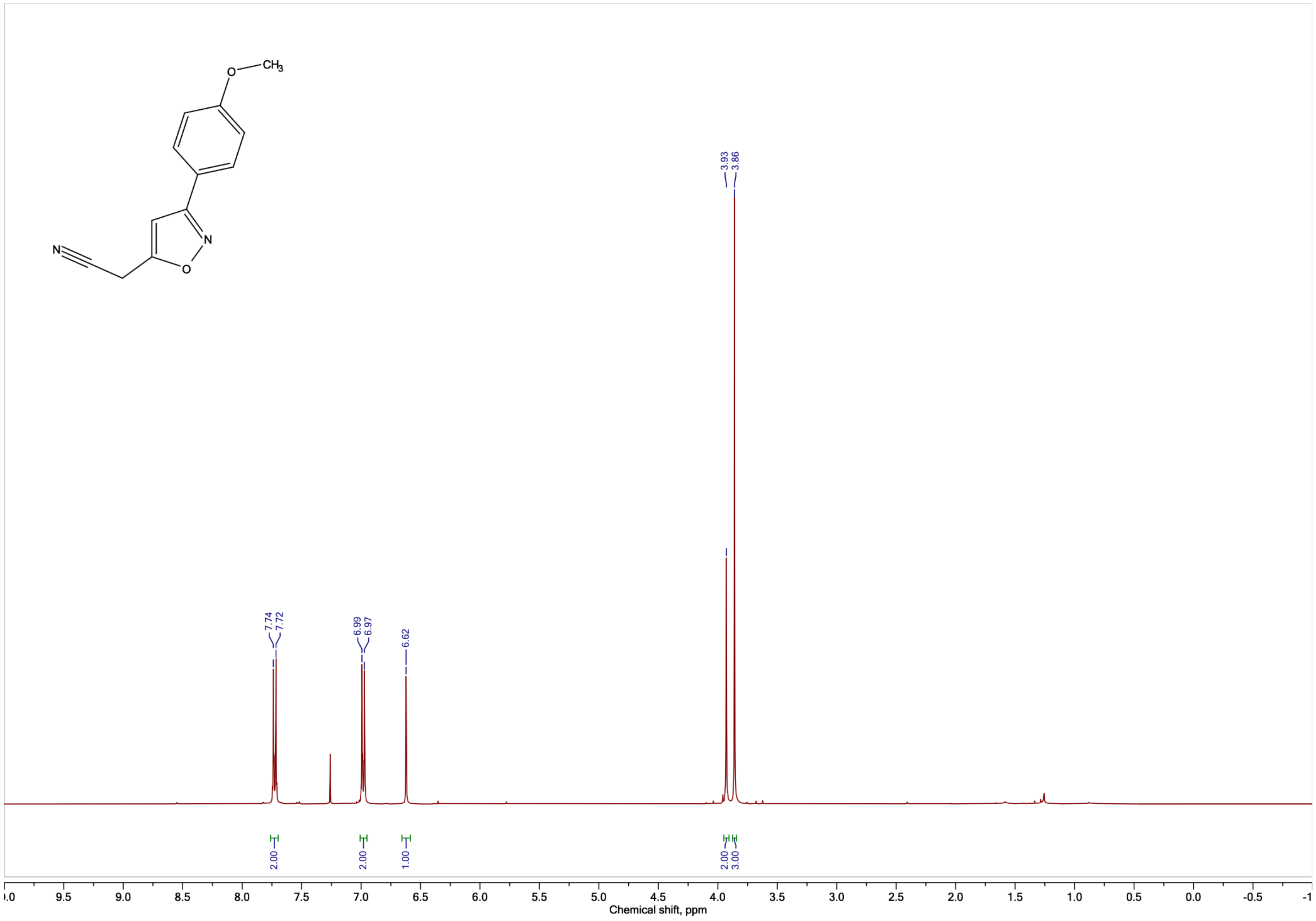

2-(3-(4-Methoxyphenyl)isoxazol-5-yl)acetonitrile (9d), $^{13}C\{^{1}H\}$ NMR, $CDCl_3$, 101 MHz

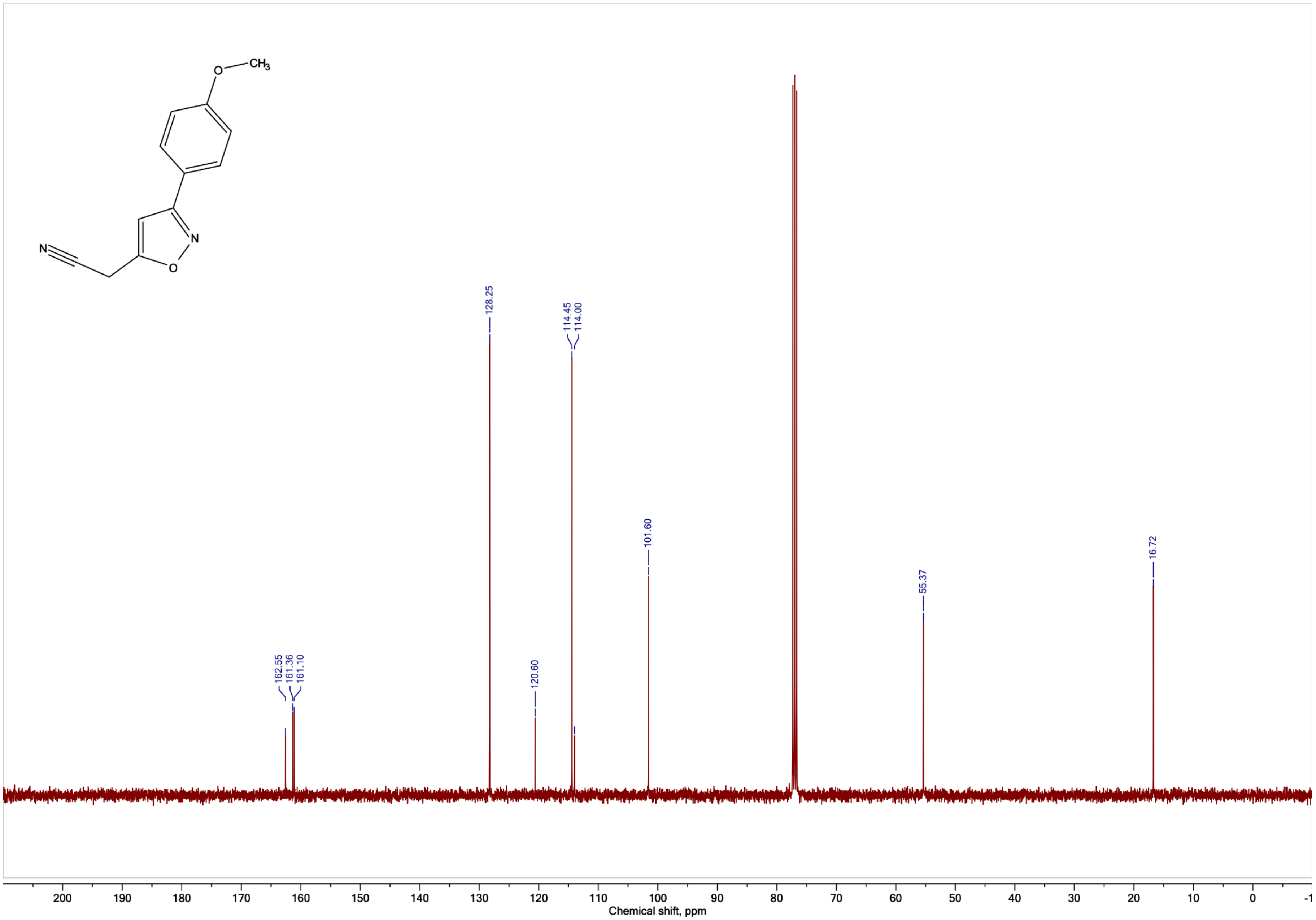

2-(3-(4-Methoxyphenyl)isoxazol-5-yl)acetonitrile (9d), DEPT, $CDCl_3$, 101 MHz

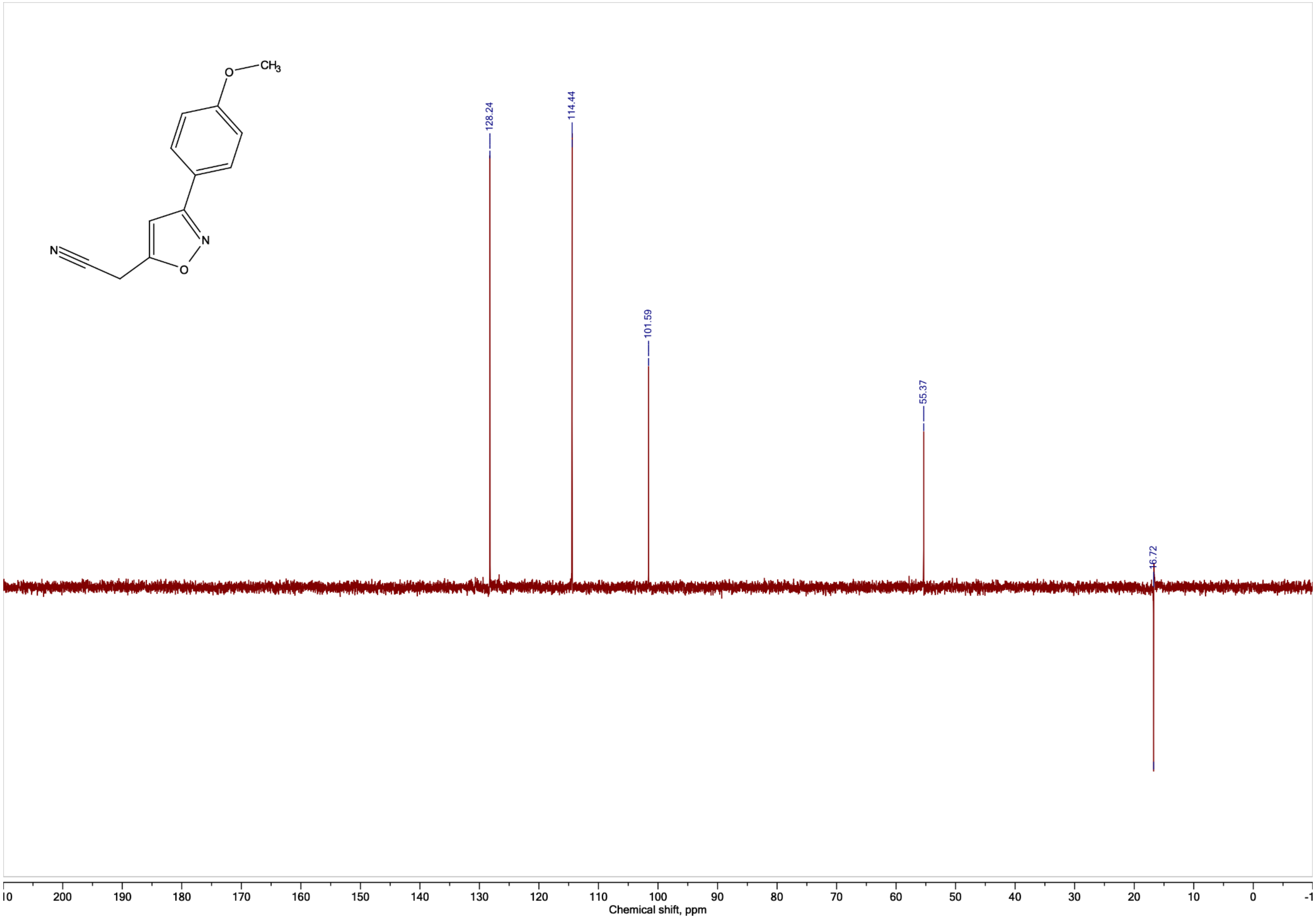

2-(3-(Quinolin-2-yl)isoxazol-5-yl)acetonitrile (9h), ^{1}H NMR, $CDCl_3$, 400 MHz

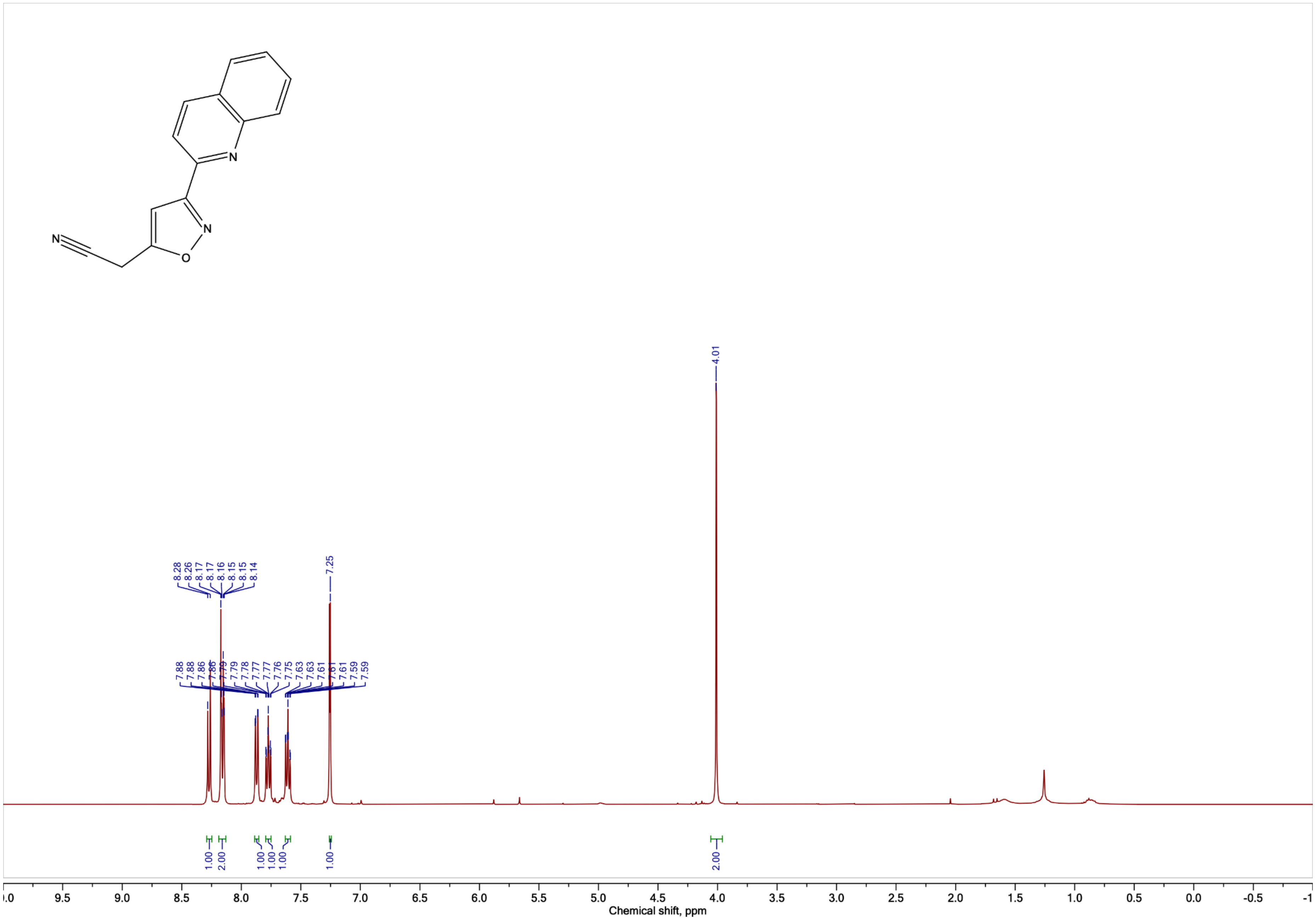

2-(3-(Quinolin-2-yl)isoxazol-5-yl)acetonitrile (9h), $^{13}C\{^{1}H\}$ NMR, $CDCl_3$, 101 MHz

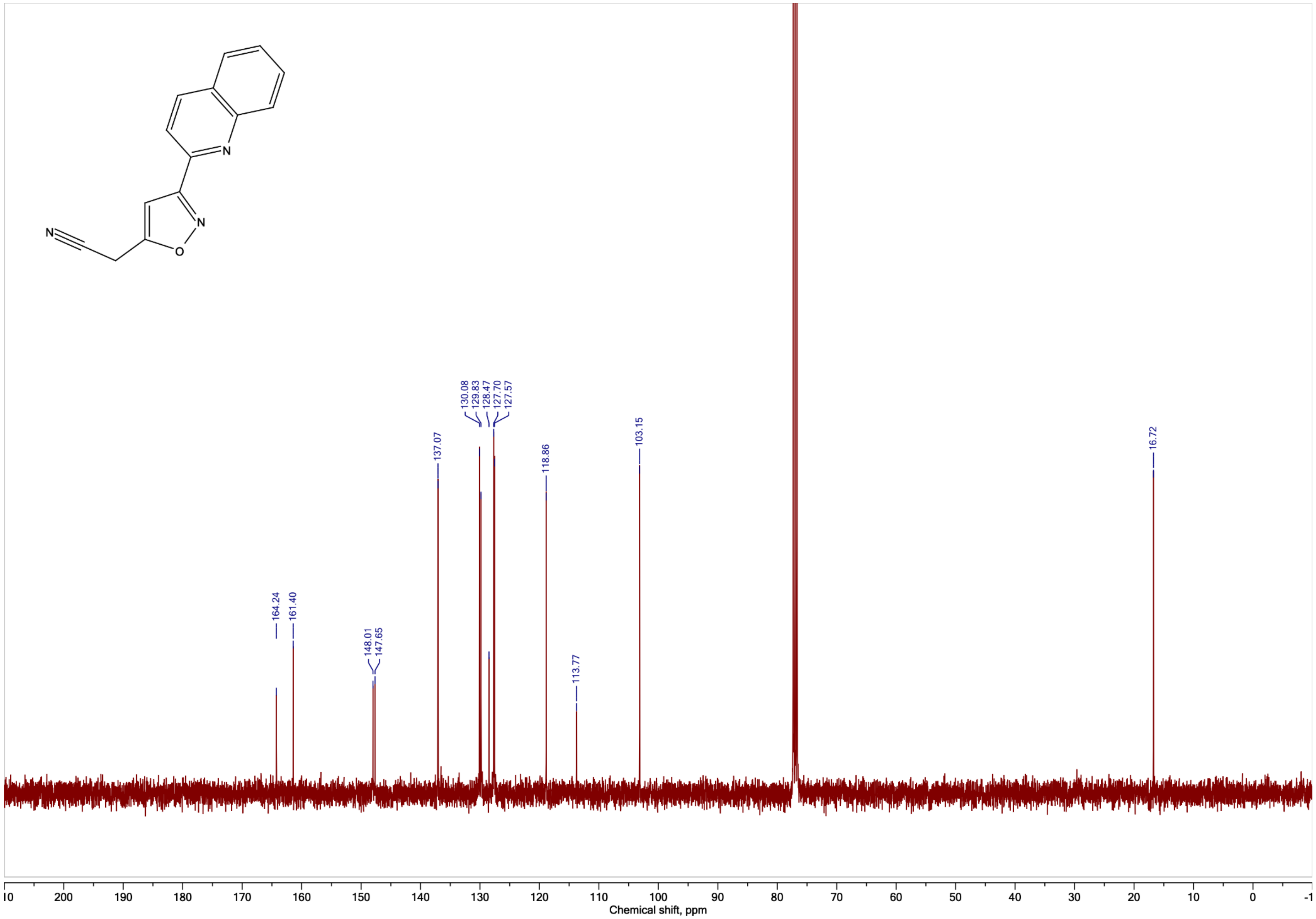

2-(3-(Quinolin-2-yl)isoxazol-5-yl)acetonitrile (9h), DEPT, $CDCl_3$, 101 MHz

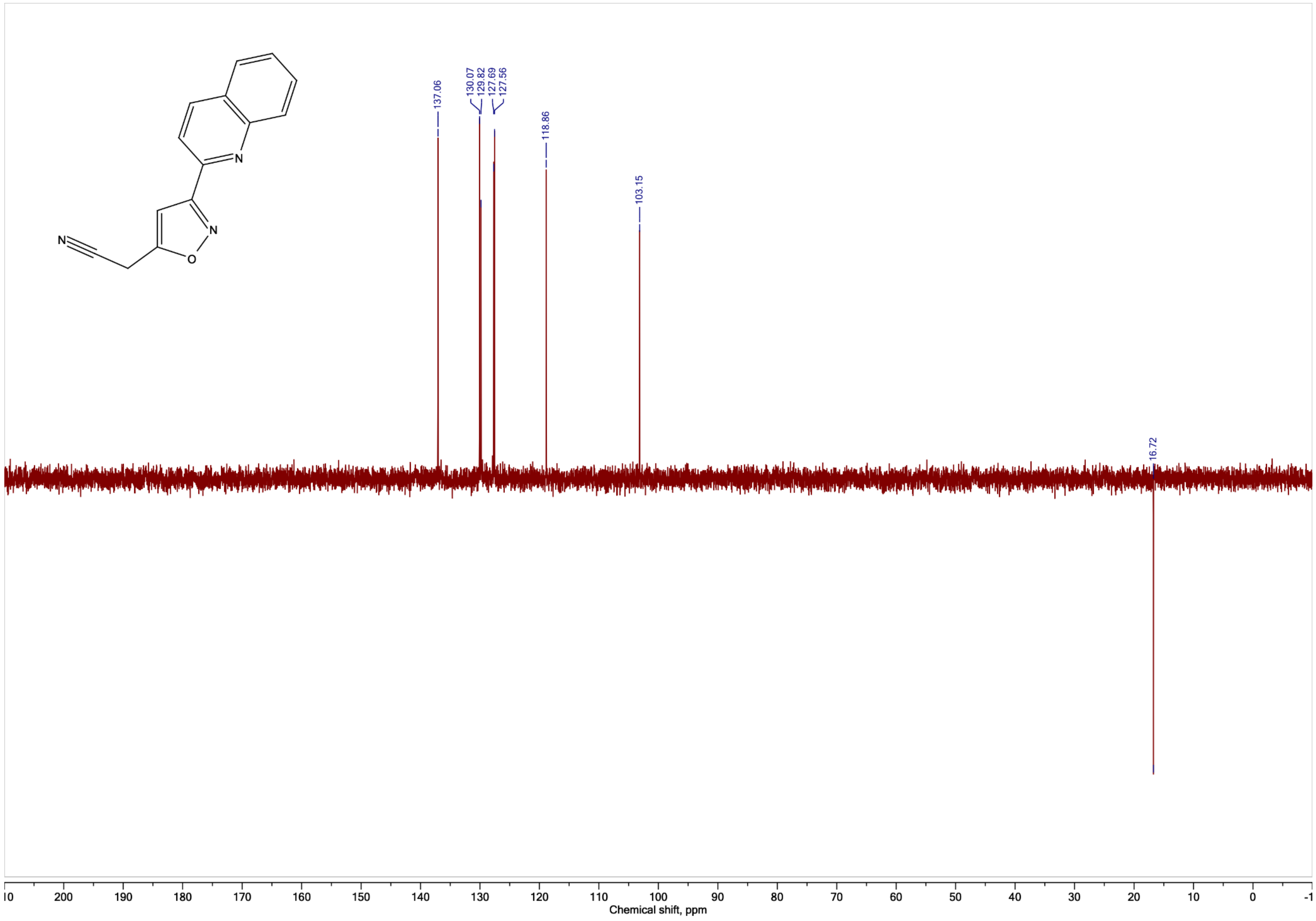

2-(3-Benzylisoxazol-5-yl)acetonitrile (9i), ^{1}H NMR, $CDCl_3$, 400 MHz

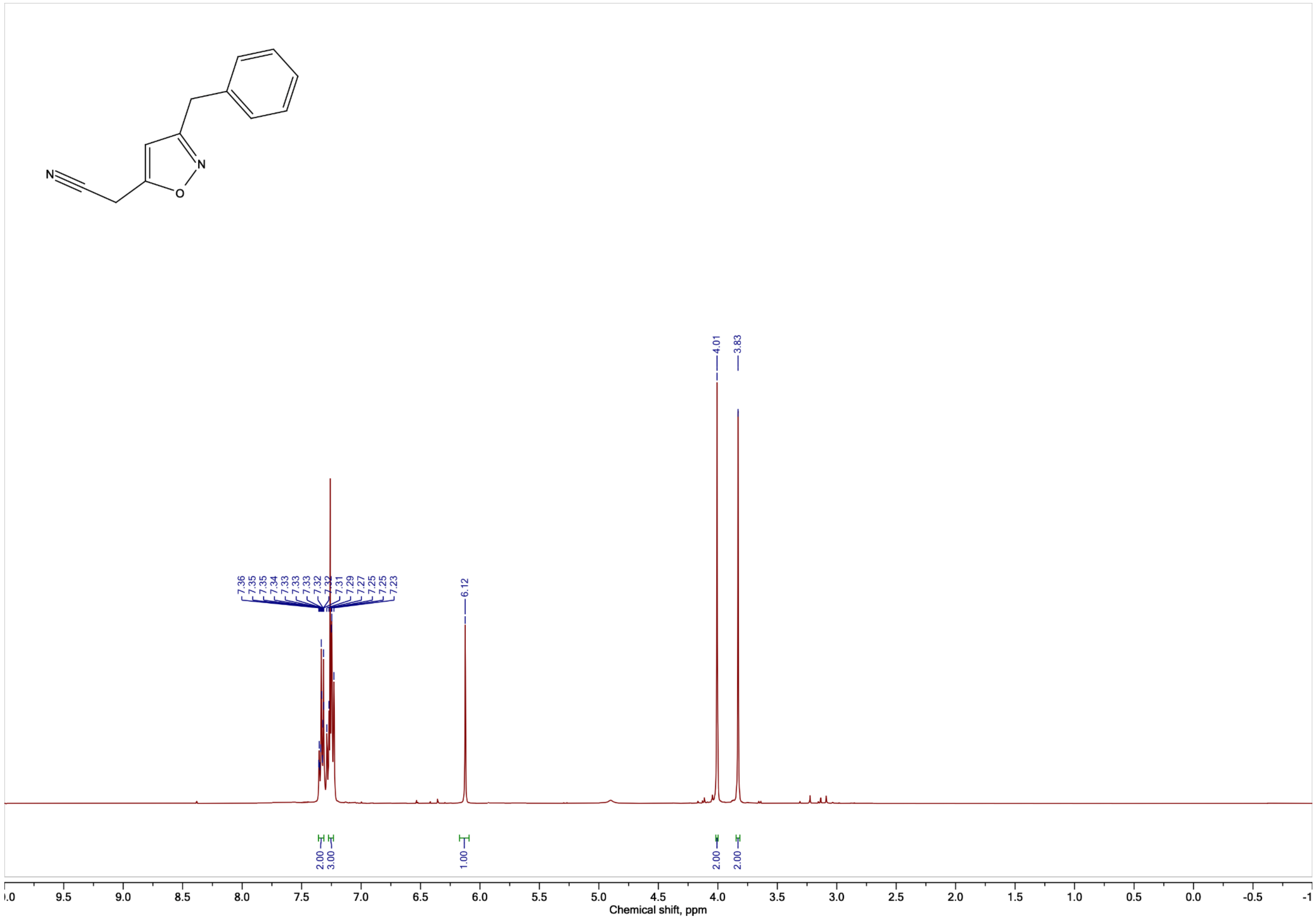

2-(3-Benzylisoxazol-5-yl)acetonitrile (9i), $^{13}C\{^{1}H\}$ NMR, $CDCl_3$, 101 MHz

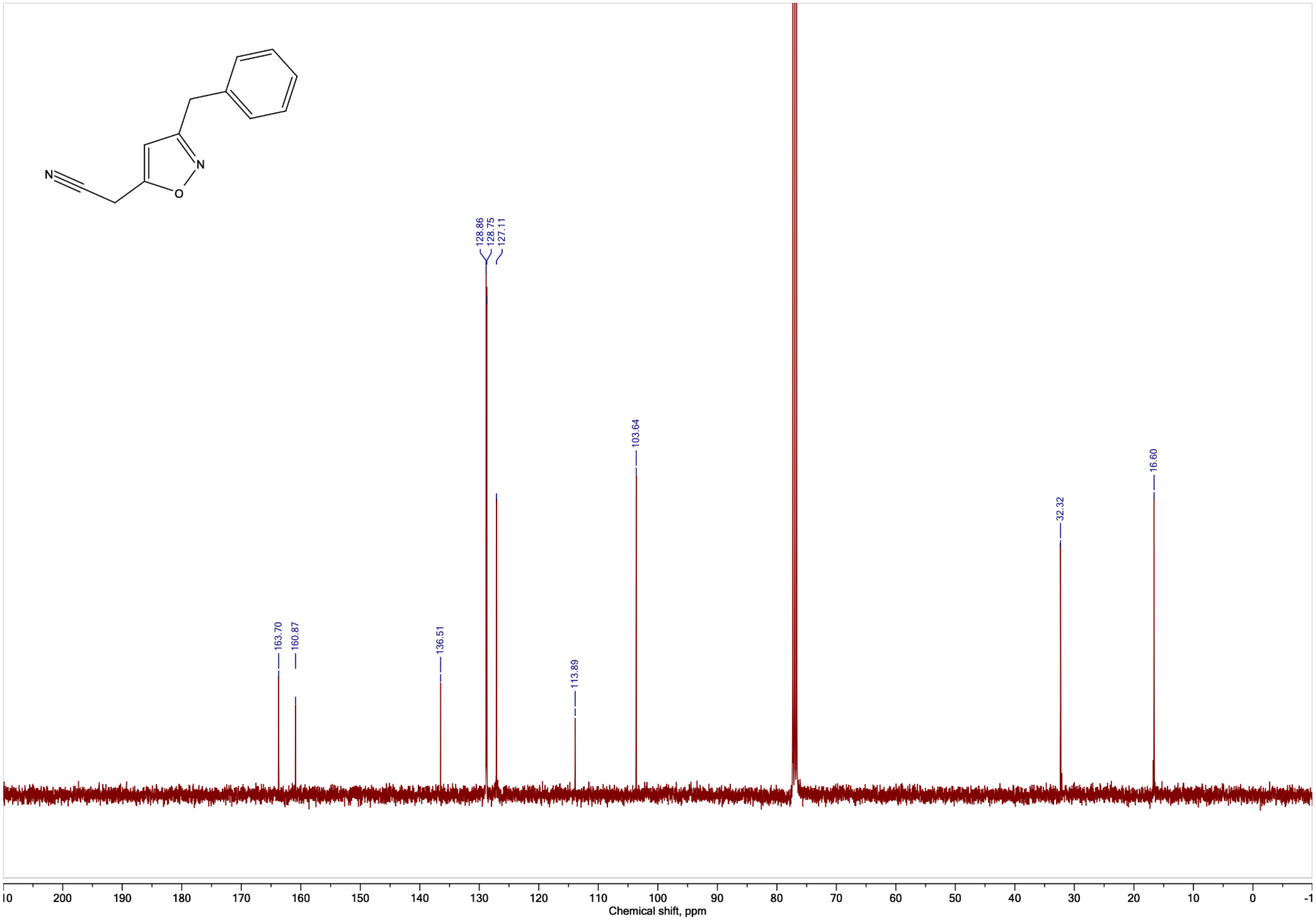

2-(3-Benzylisoxazol-5-yl)acetonitrile (9i), DEPT, $CDCl_3$, 101 MHz

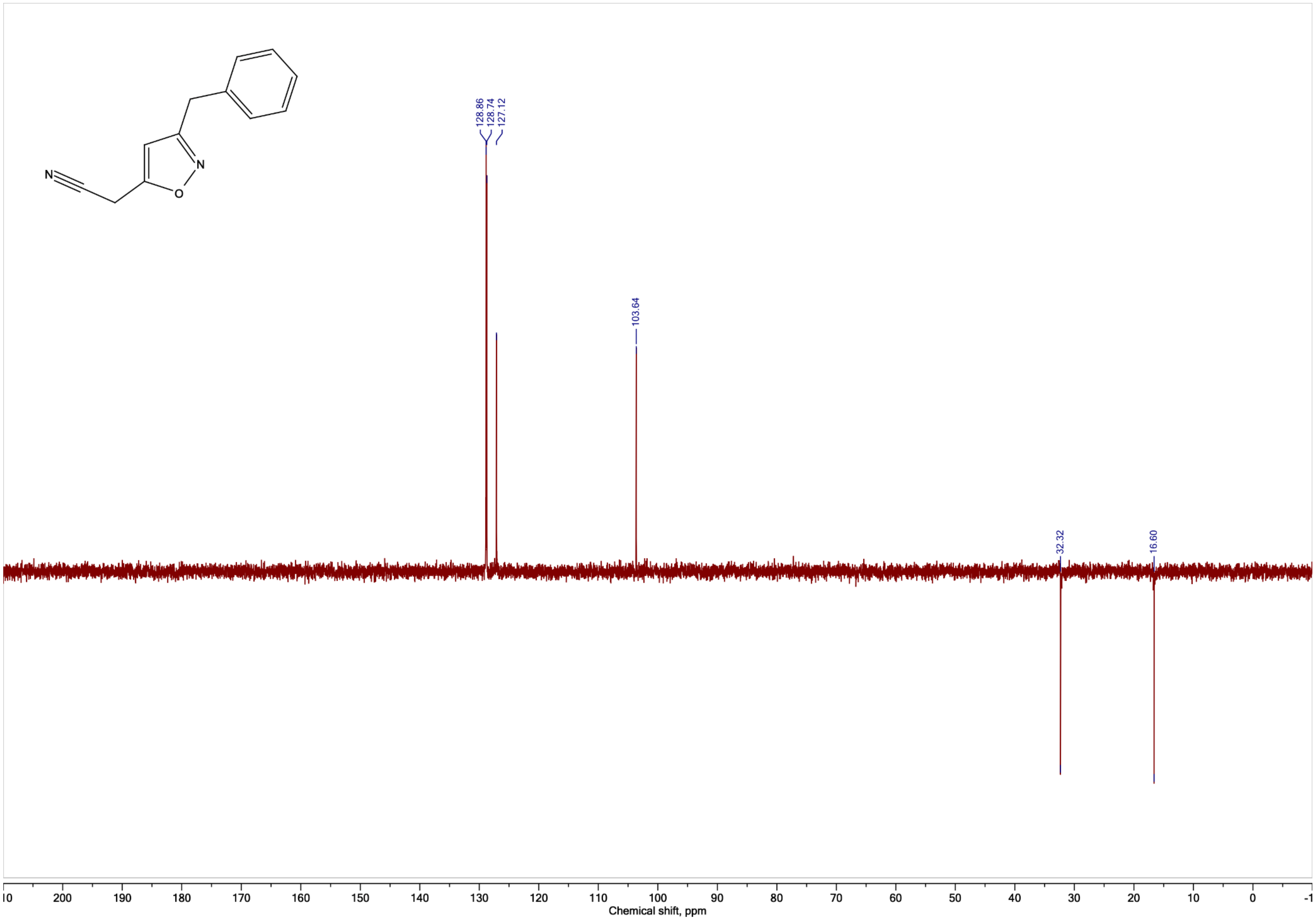

2-(3-(4-Methoxyphenyl)isoxazol-5-yl)acetic acid (10d), ^{1}H NMR, DMSO-d$_6$, 400 MHz

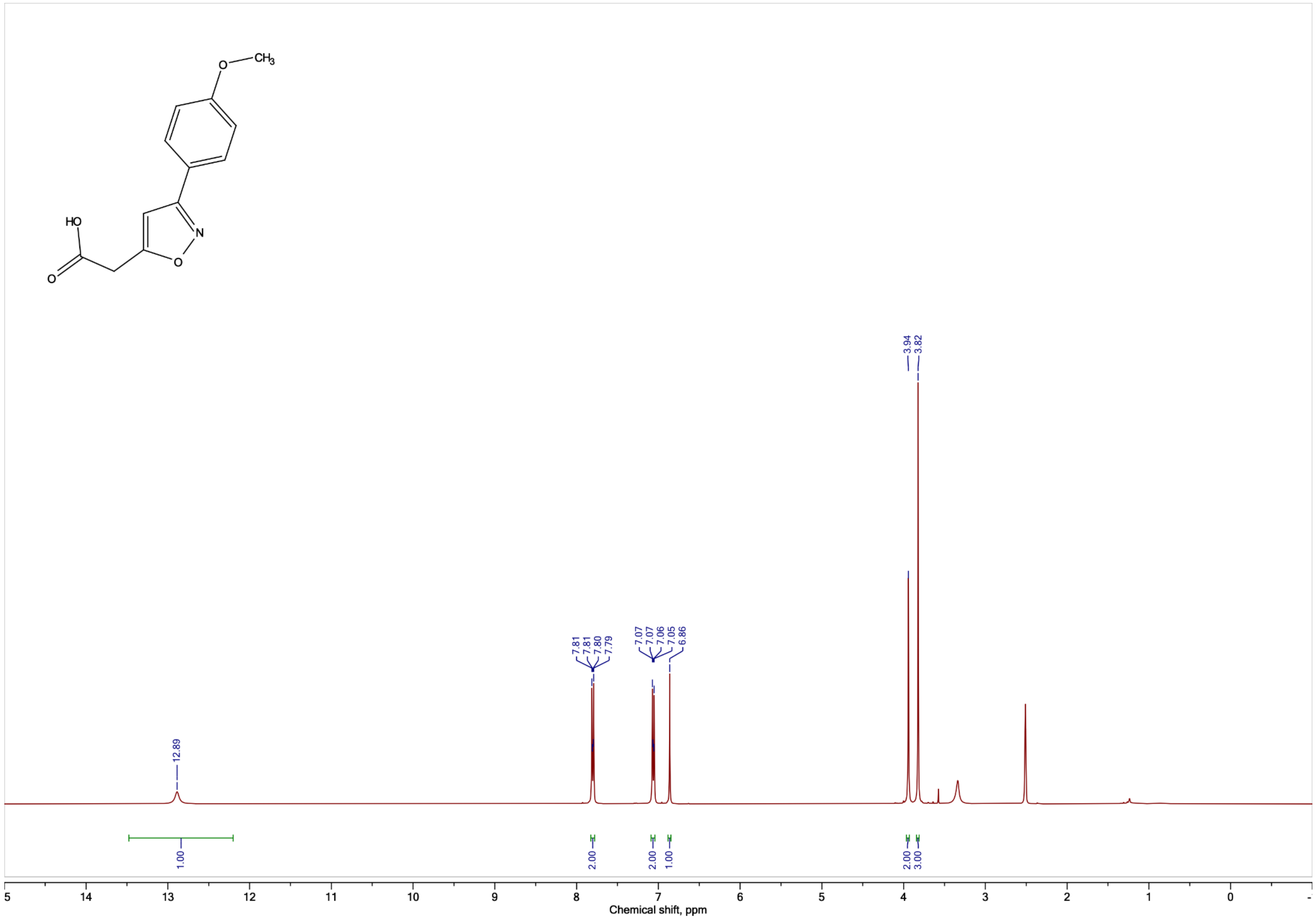

2-(3-(4-Methoxyphenyl)isoxazol-5-yl)acetic acid (10d), $^{13}C\{^1H\}$ NMR, DMSO-d_6, 101 MHz

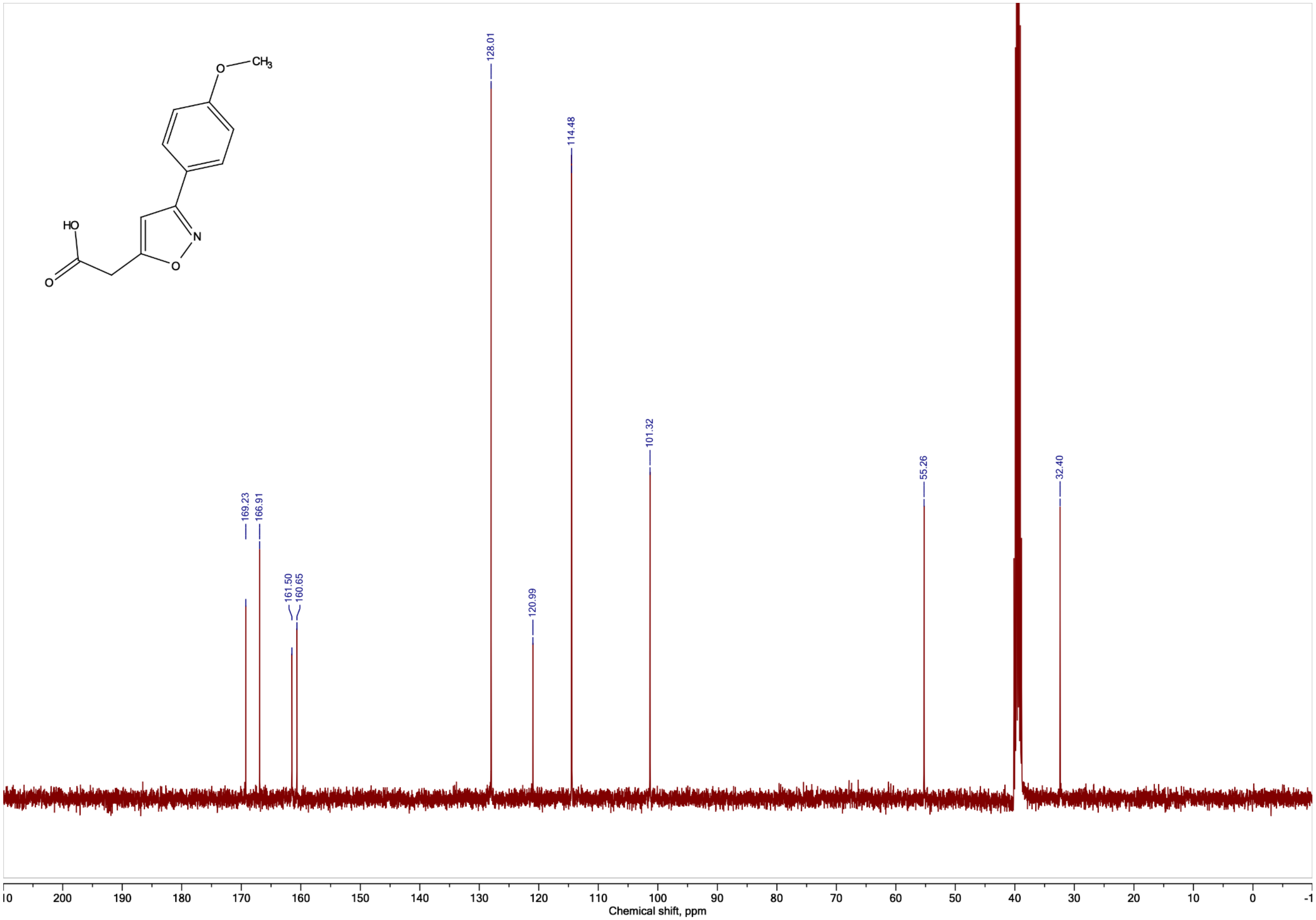

2-(3-(4-Methoxyphenyl)isoxazol-5-yl)acetic acid (10d), DEPT, DMSO-d_6, 101 MHz

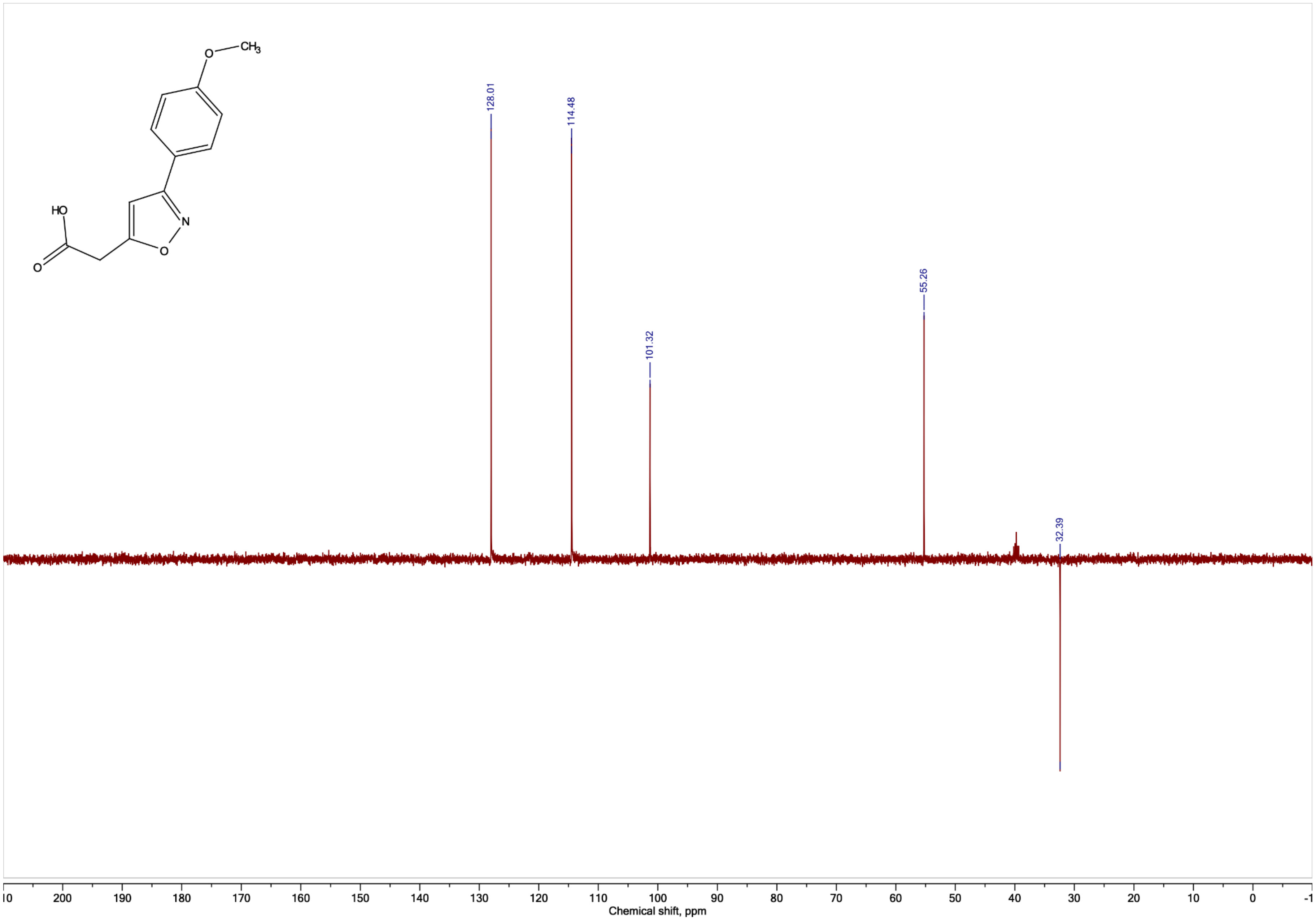

2-(3-(Quinolin-2-yl)isoxazol-5-yl)acetic acid (10h), ^{1}H NMR, DMSO-d_6, 400 MHz

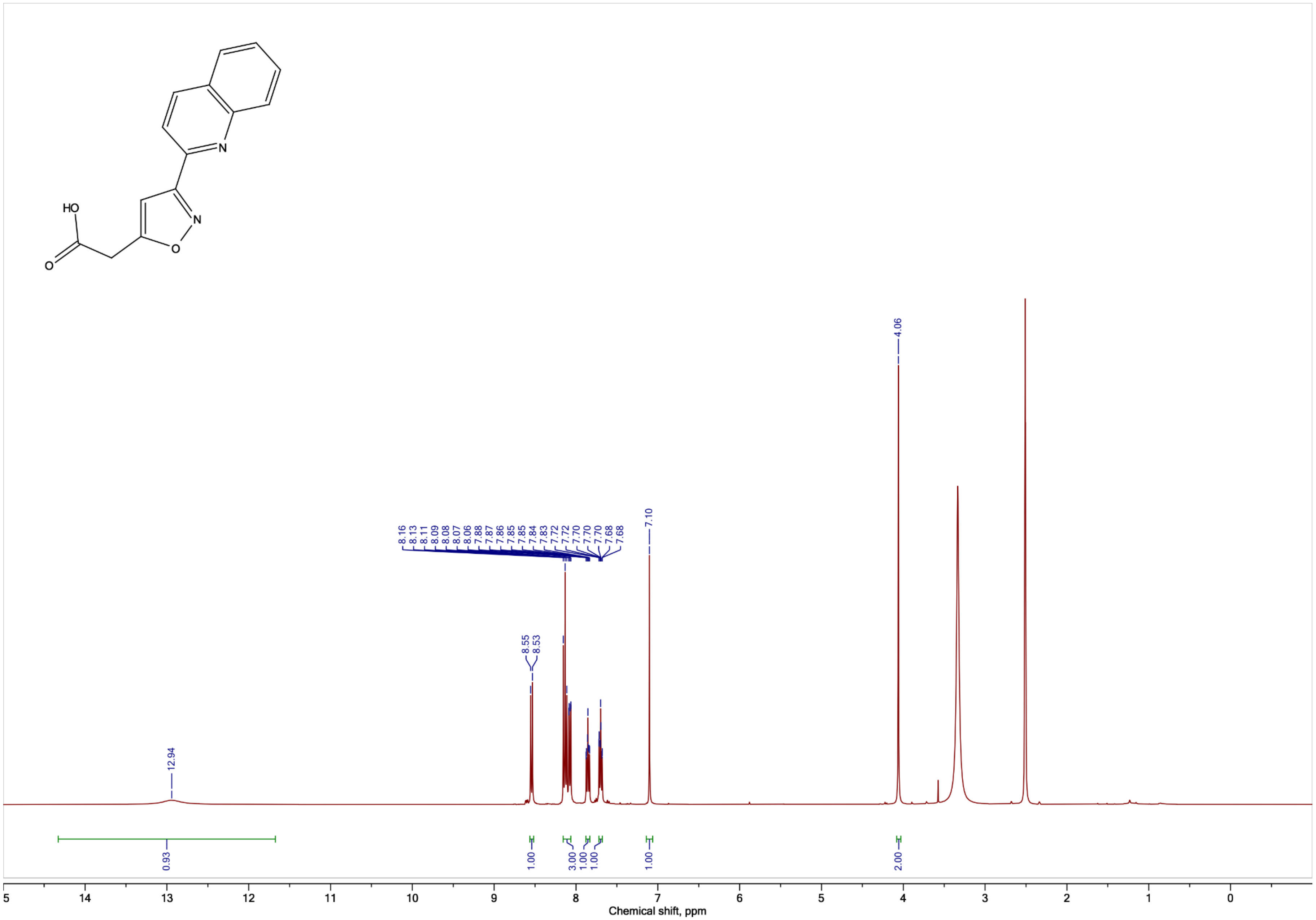

2-(3-(Quinolin-2-yl)isoxazol-5-yl)acetic acid (10h), $^{13}C\{^{1}H\}$ NMR, DMSO-d_6, 101 MHz

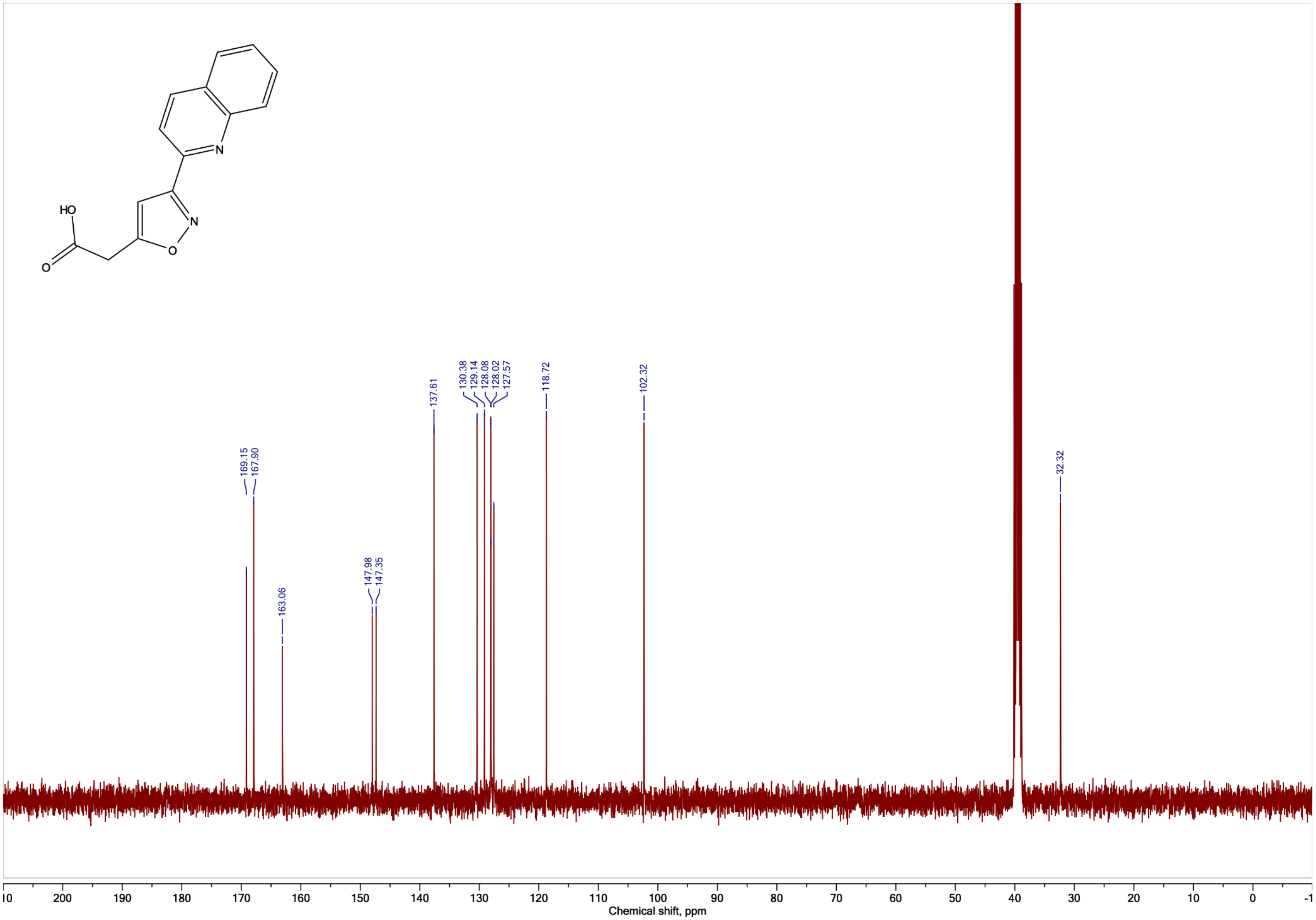

2-(3-(Quinolin-2-yl)isoxazol-5-yl)acetic acid (10h), DEPT, DMSO-d_6, 101 MHz

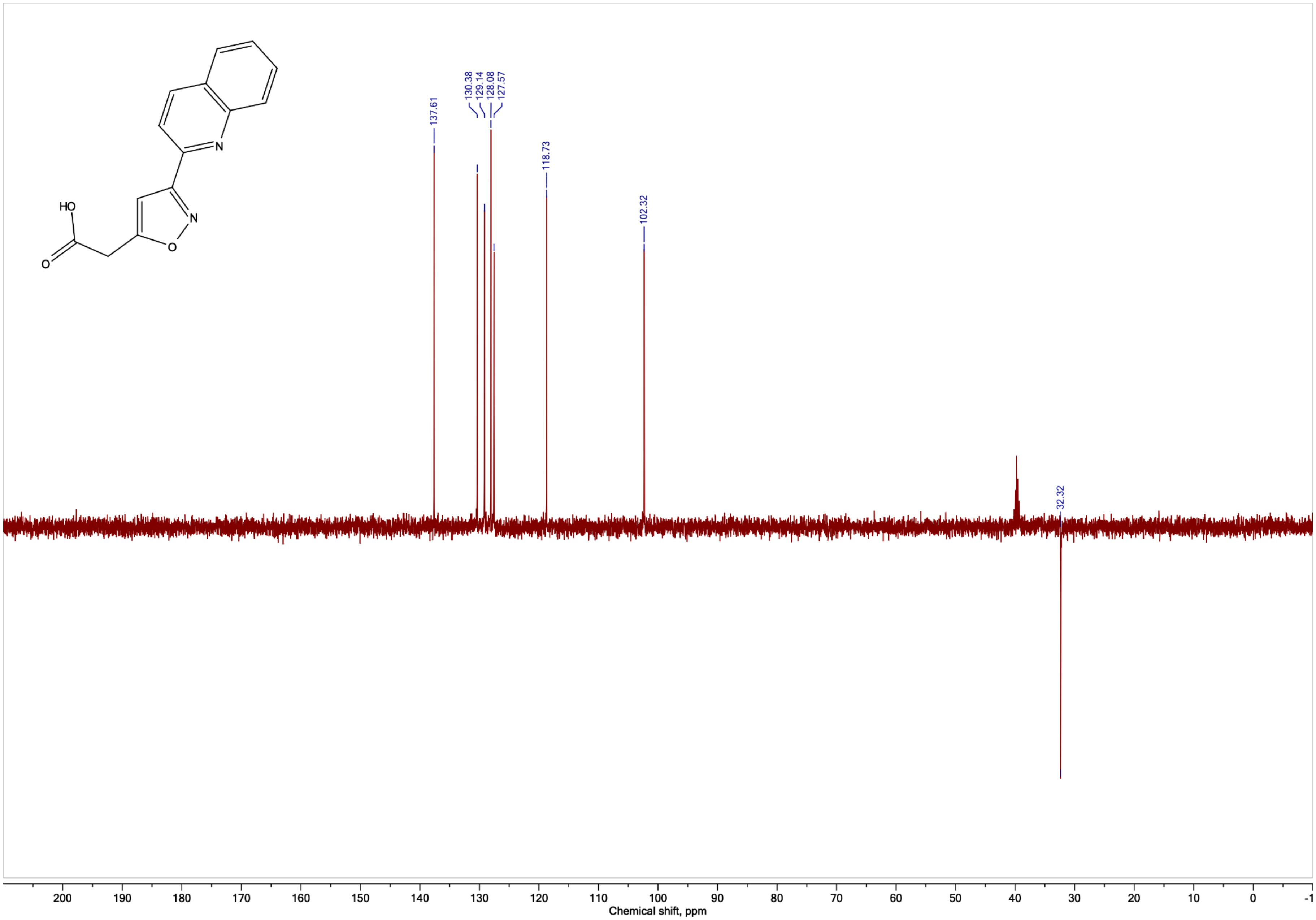

2-(3-Benzylisoxazol-5-yl)acetic acid (10i), ^{1}H NMR, DMSO-d_6, 400 MHz

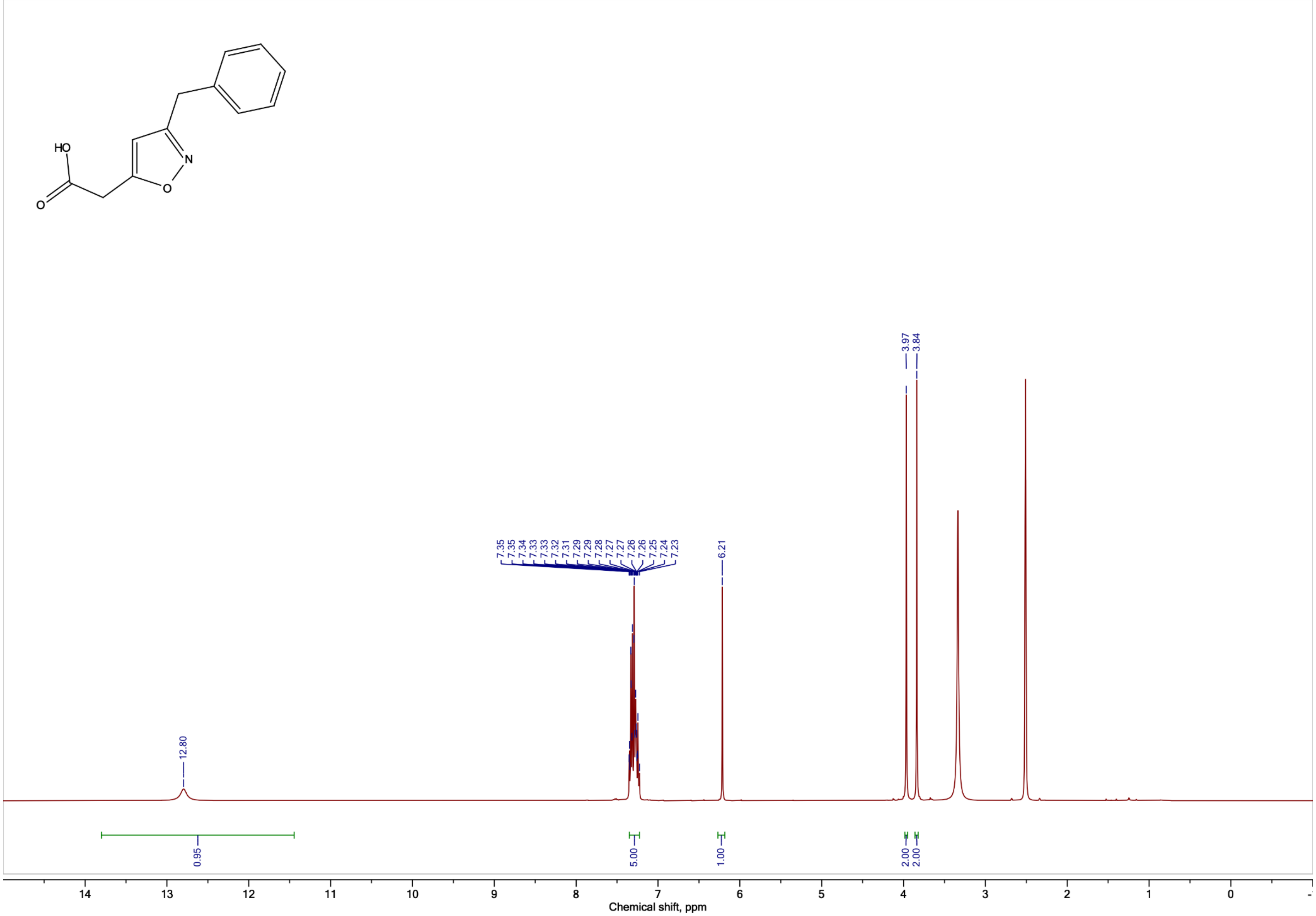

2-(3-Benzylisoxazol-5-yl)acetic acid (10i), $^{13}C\{^1H\}$ NMR, DMSO-d_6, 101 MHz

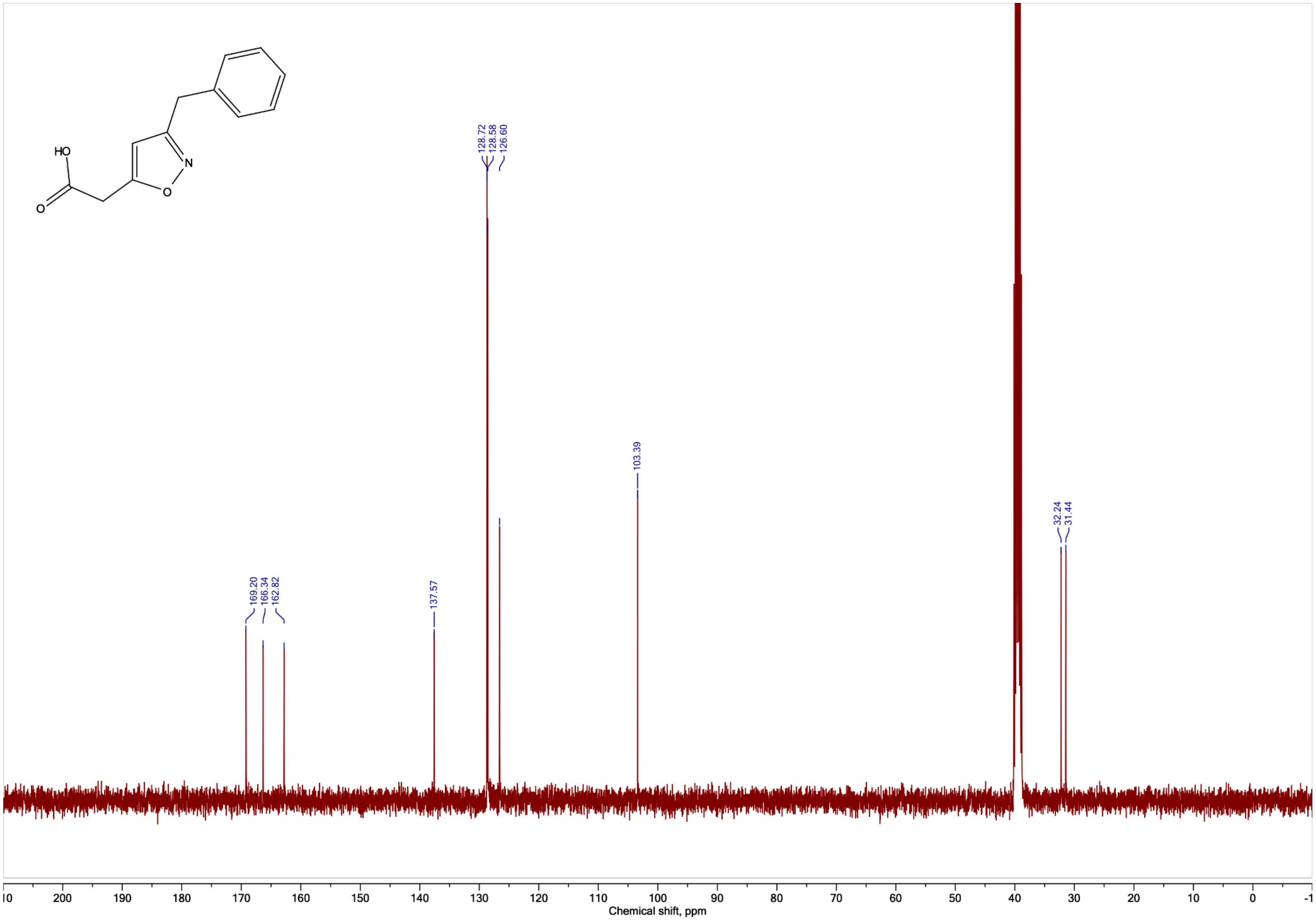

2-(3-Benzylisoxazol-5-yl)acetic acid (10i), DEPT, DMSO-d_6, 101 MHz

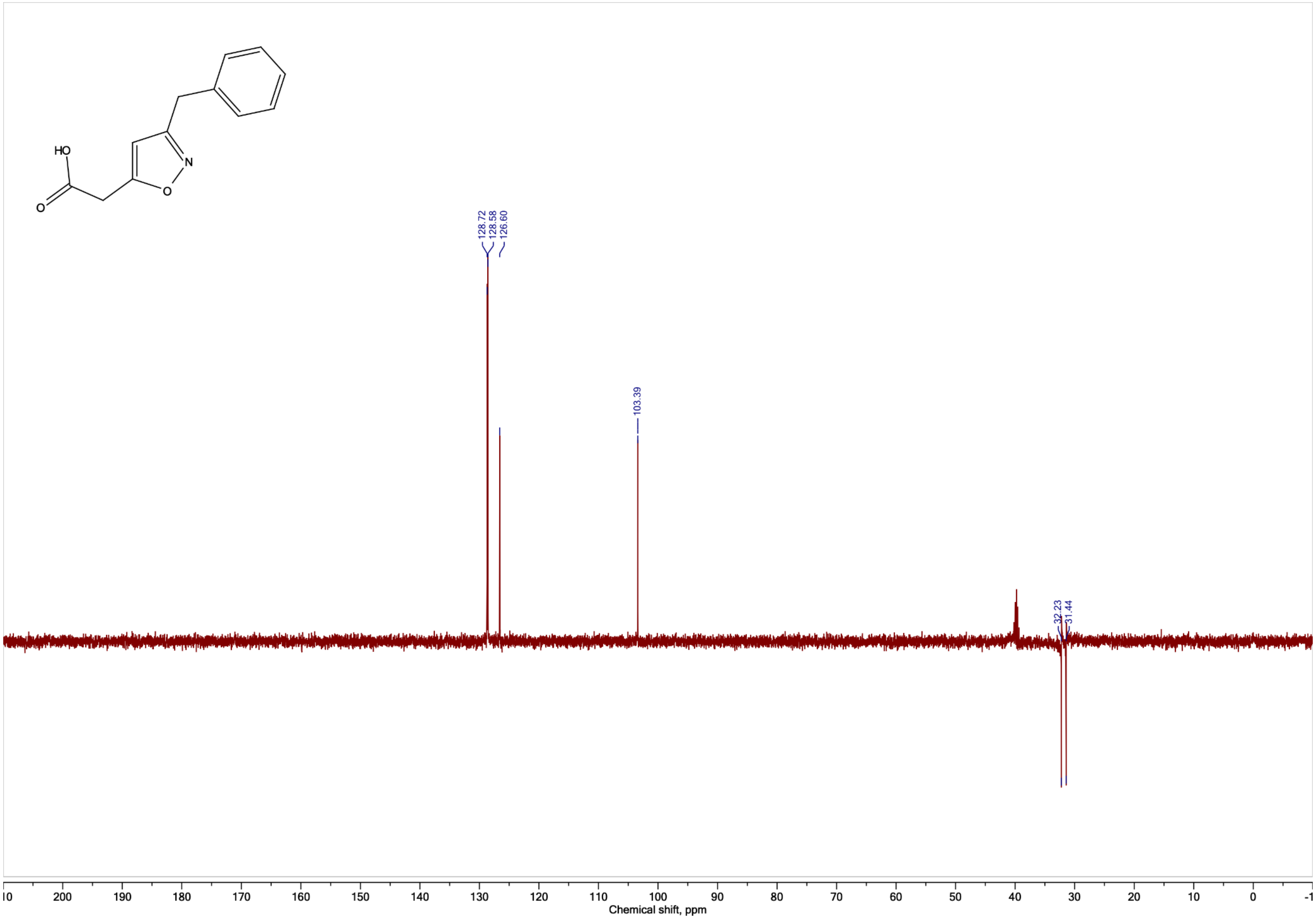

2-(3-Heptylisoxazol-5-yl)acetic acid (10j), ^{1}H NMR, DMSO-d_6, 400 MHz

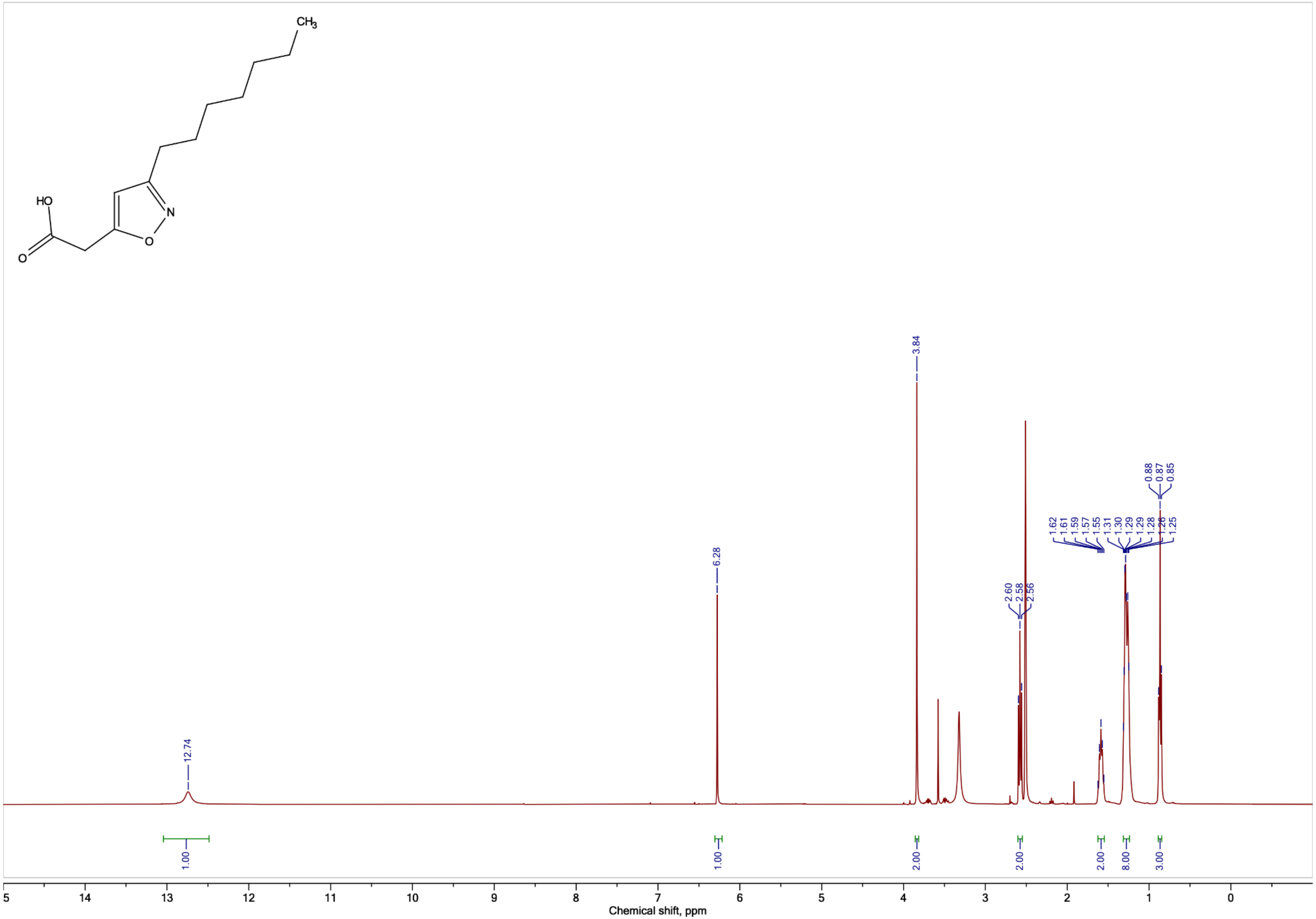

2-(3-Heptylisoxazol-5-yl)acetic acid (10j), $^{13}C\{^{1}H\}$ NMR, DMSO-d_6, 101 MHz

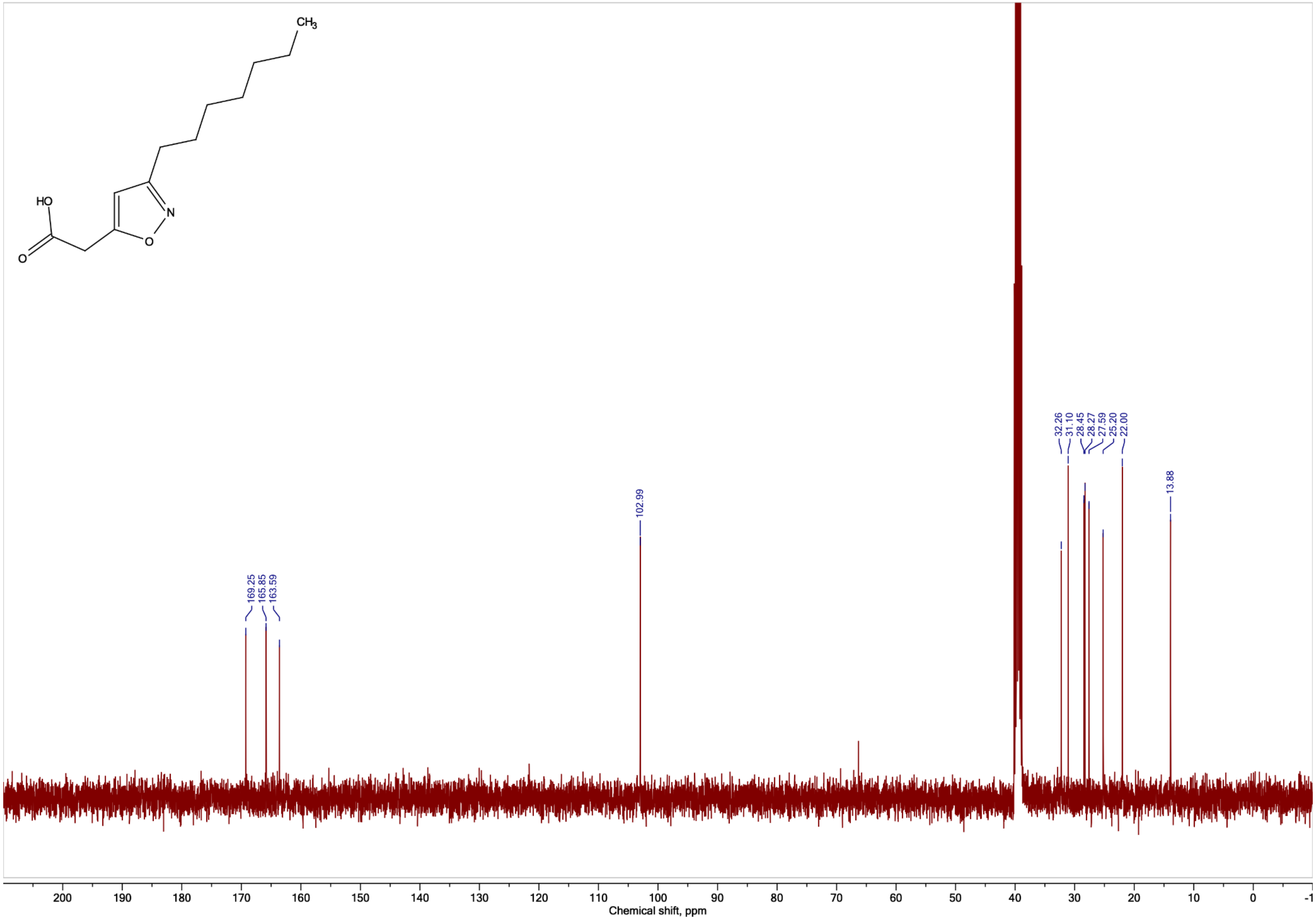

2-(3-Heptylisoxazol-5-yl)acetic acid (10j), DEPT, DMSO-d_6, 101 MHz

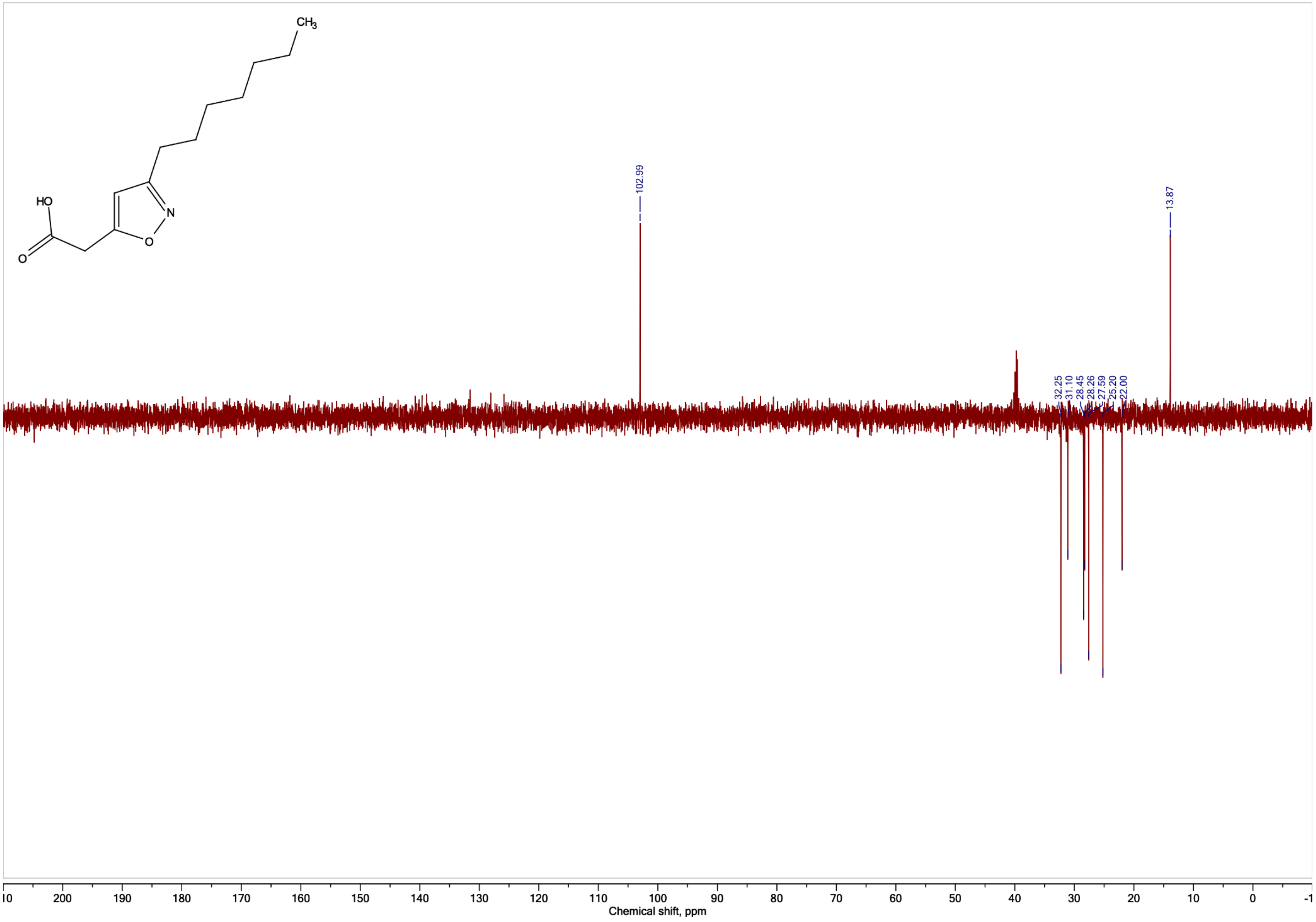

Methyl 2-(3-(4-methoxyphenyl)isoxazol-5-yl)acetate (11d), ^{1}H NMR, $CDCl_3$, 400 MHz

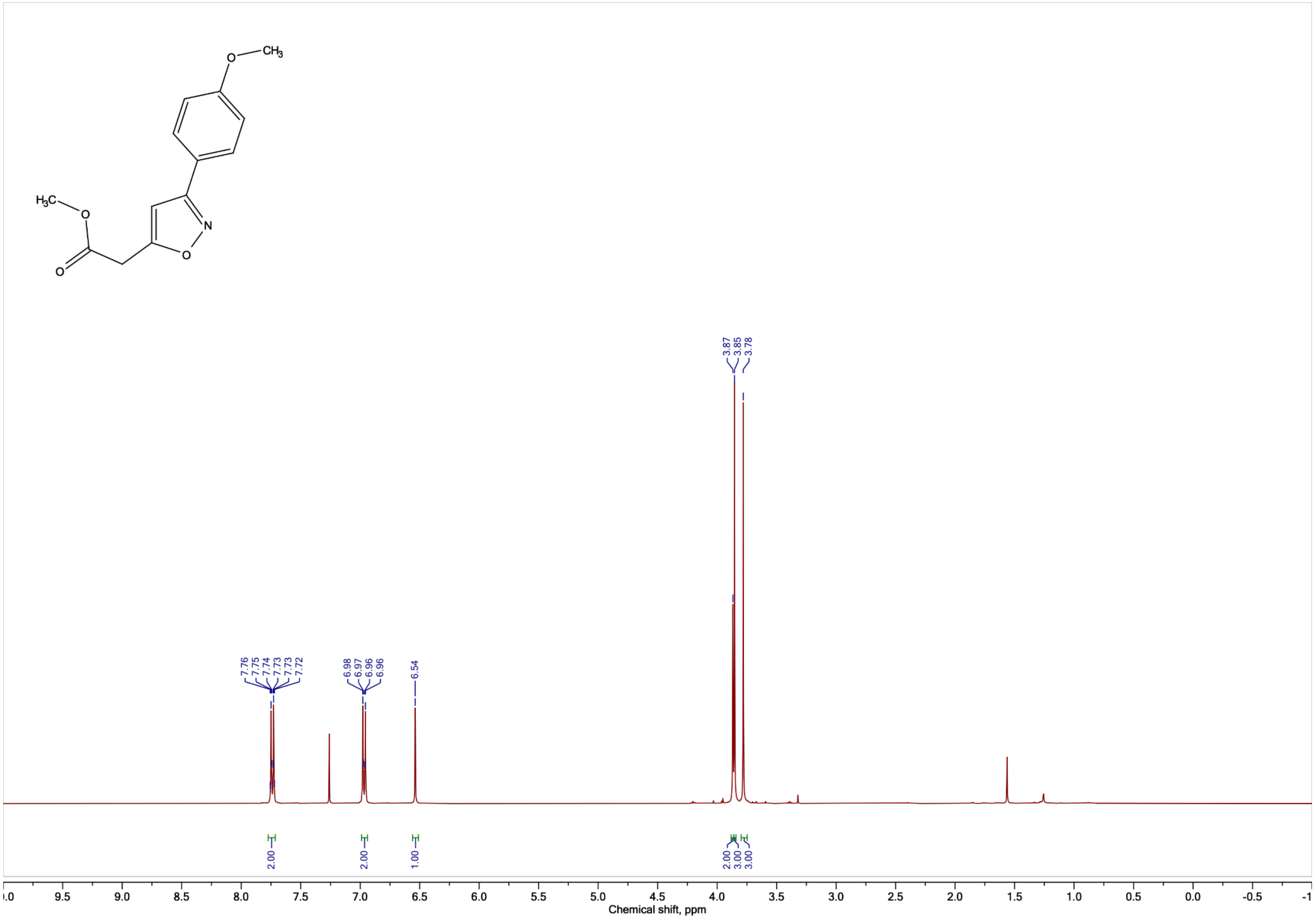

Methyl 2-(3-(4-methoxyphenyl)isoxazol-5-yl)acetate (11d), $^{13}C\{^1H\}$ NMR, $CDCl_3$, 101 MHz

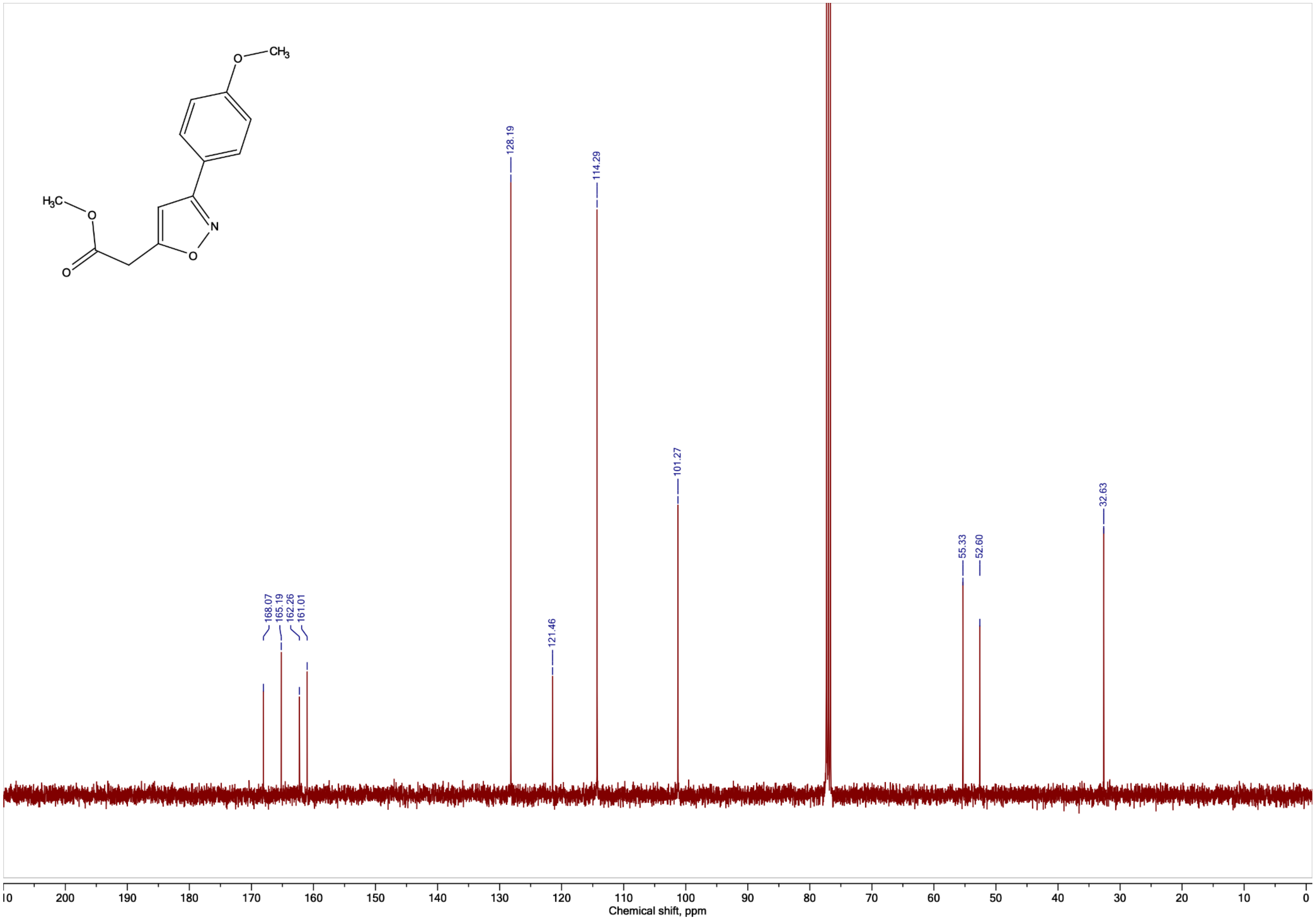

Methyl 2-(3-(4-methoxyphenyl)isoxazol-5-yl)acetate (11d), DEPT, $CDCl_3$, 101 MHz

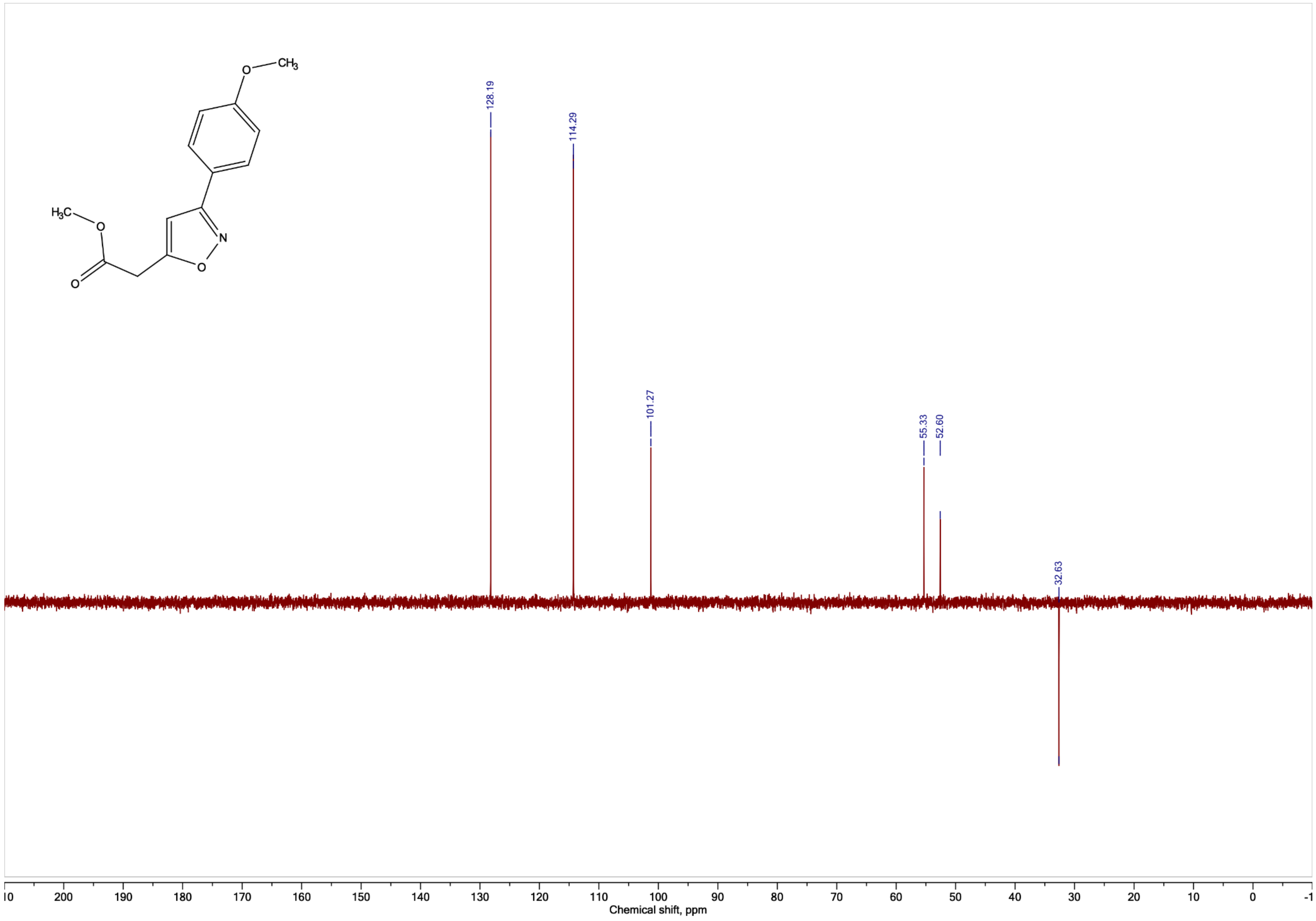

Methyl 2-(3-(quinolin-2-yl)isoxazol-5-yl)acetate (11h), ^{1}H NMR, $CDCl_3$, 400 MHz

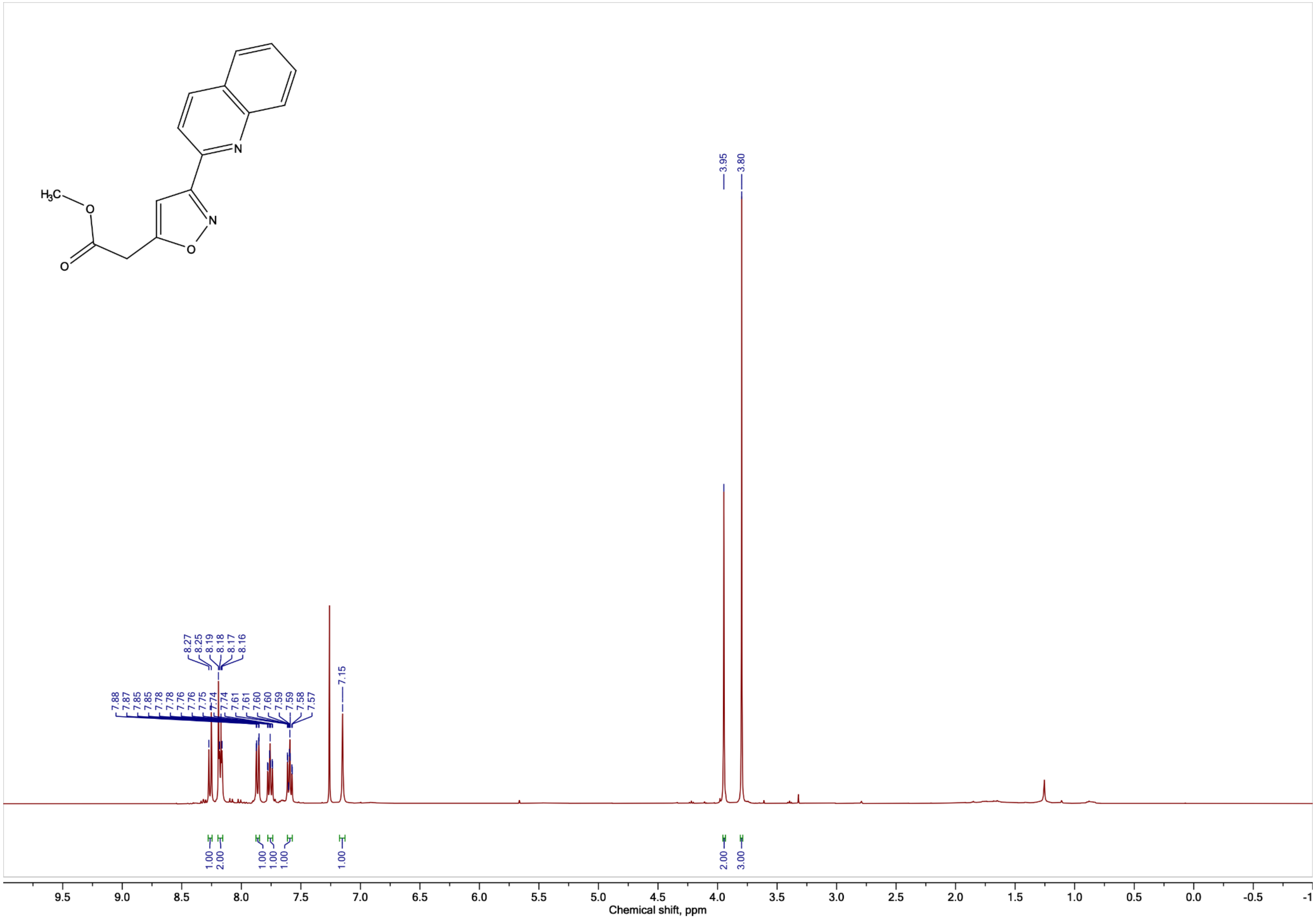

Methyl 2-(3-(quinolin-2-yl)isoxazol-5-yl)acetate (11h), $^{13}C\{^{1}H\}$ NMR, $CDCl_3$, 101 MHz

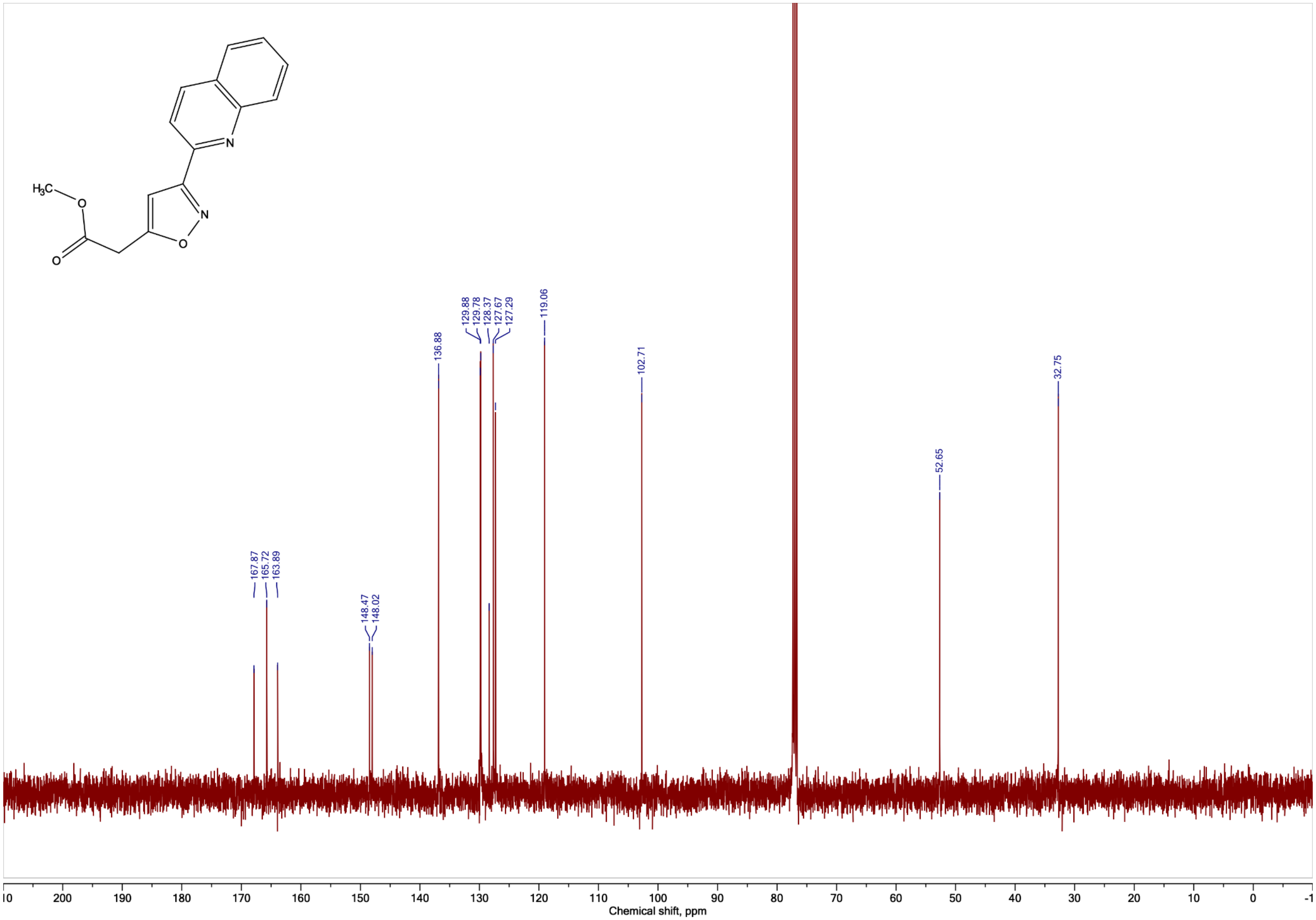

Methyl 2-(3-(quinolin-2-yl)isoxazol-5-yl)acetate (11h), DEPT, $CDCl_3$, 101 MHz

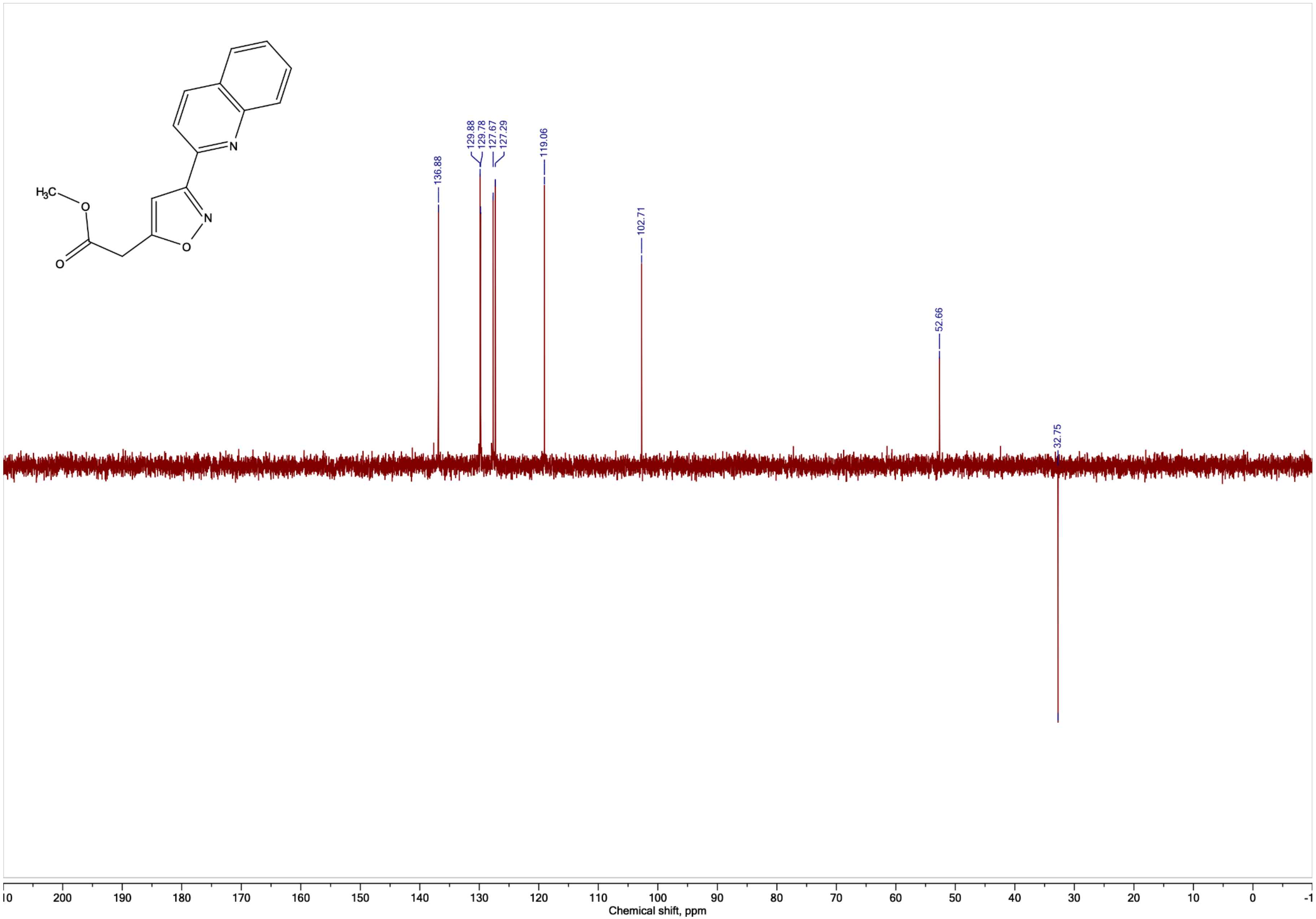

Methyl 2-(3-benzylisoxazol-5-yl)acetate (11i), ^{1}H NMR, $CDCl_3$, 400 MHz

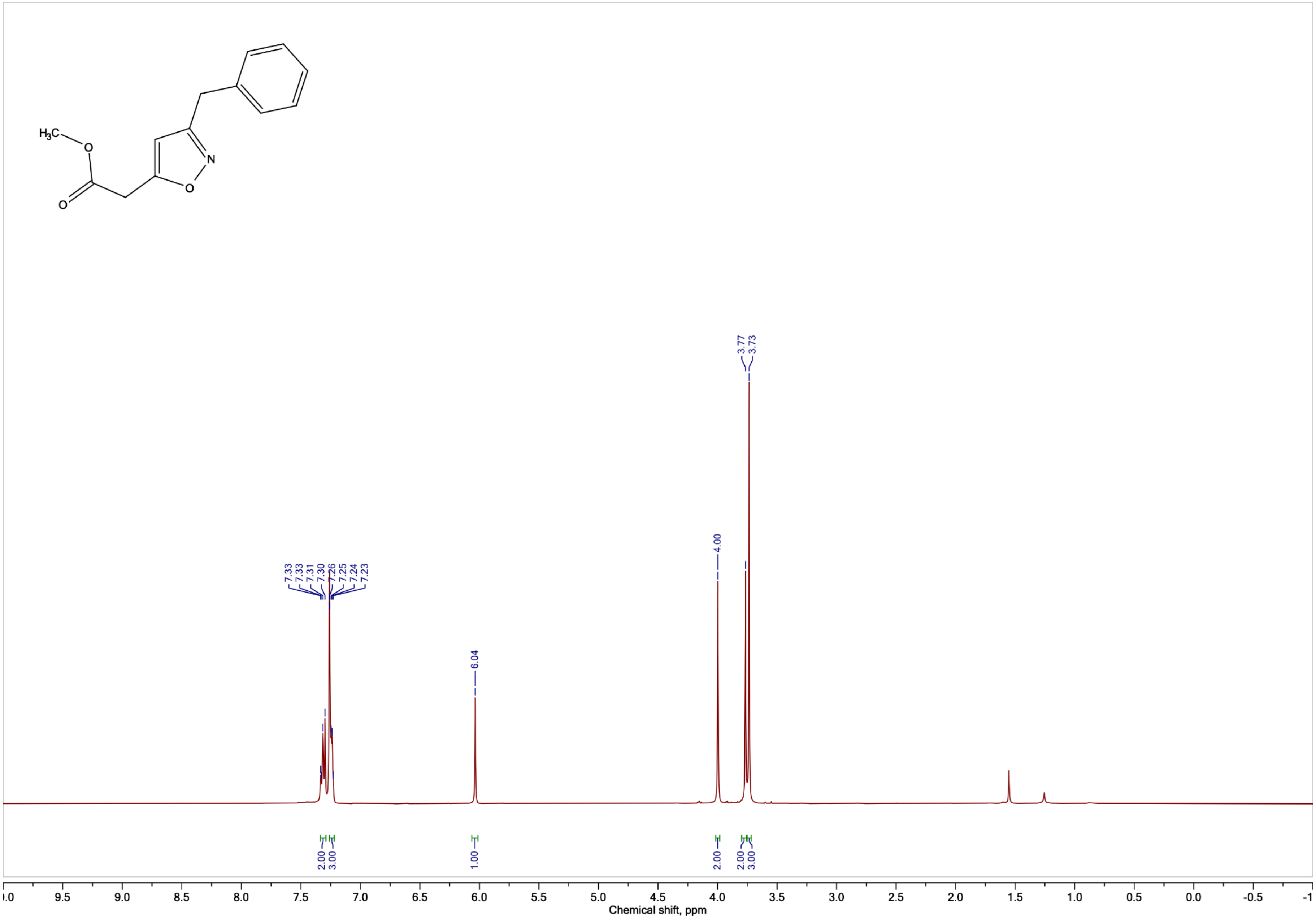

Methyl 2-(3-benzylisoxazol-5-yl)acetate (11i), $^{13}C\{^{1}H\}$ NMR, $CDCl_3$, 101 MHz

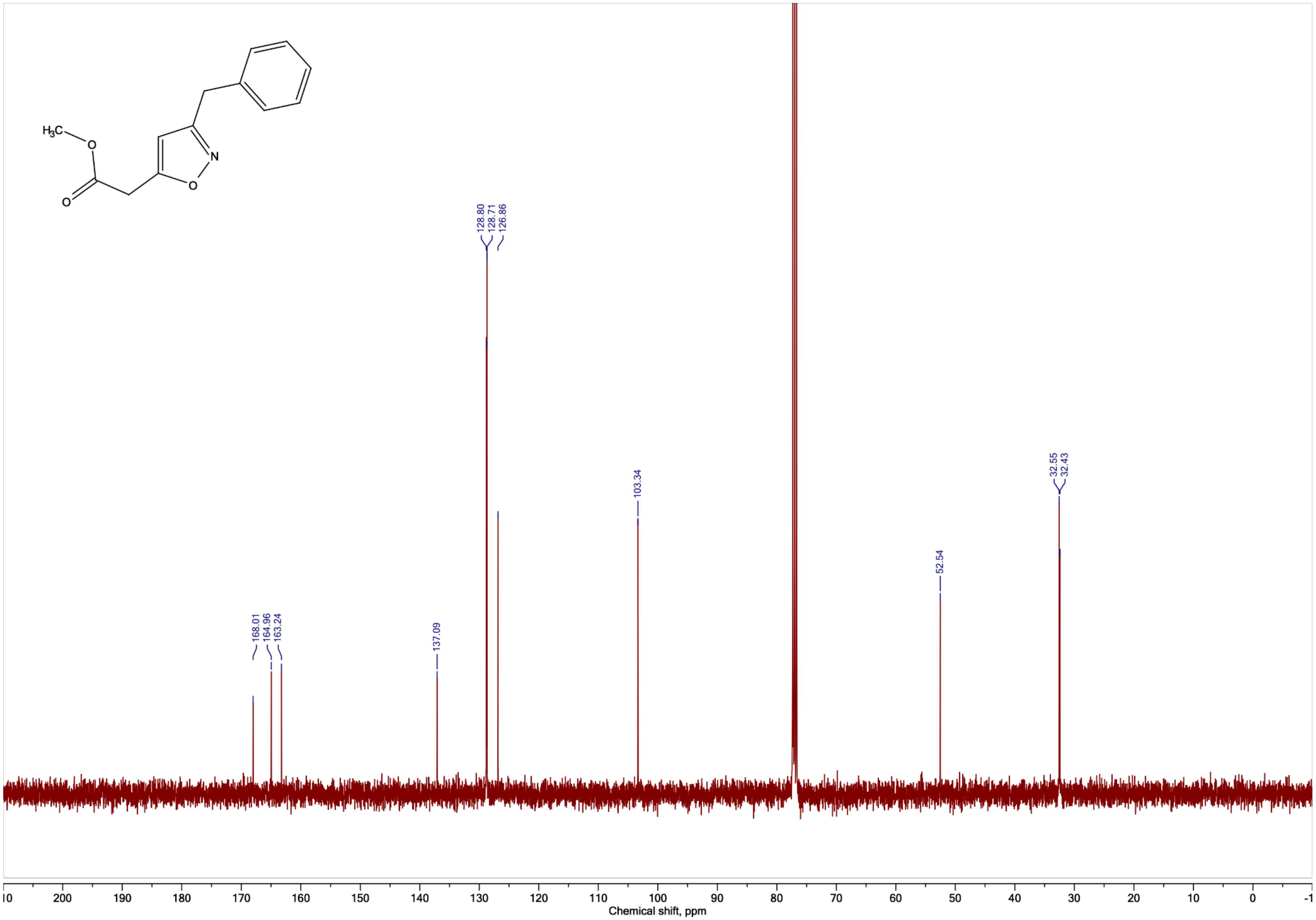

Methyl 2-(3-benzylisoxazol-5-yl)acetate (11i), DEPT, $CDCl_3$, 101 MHz

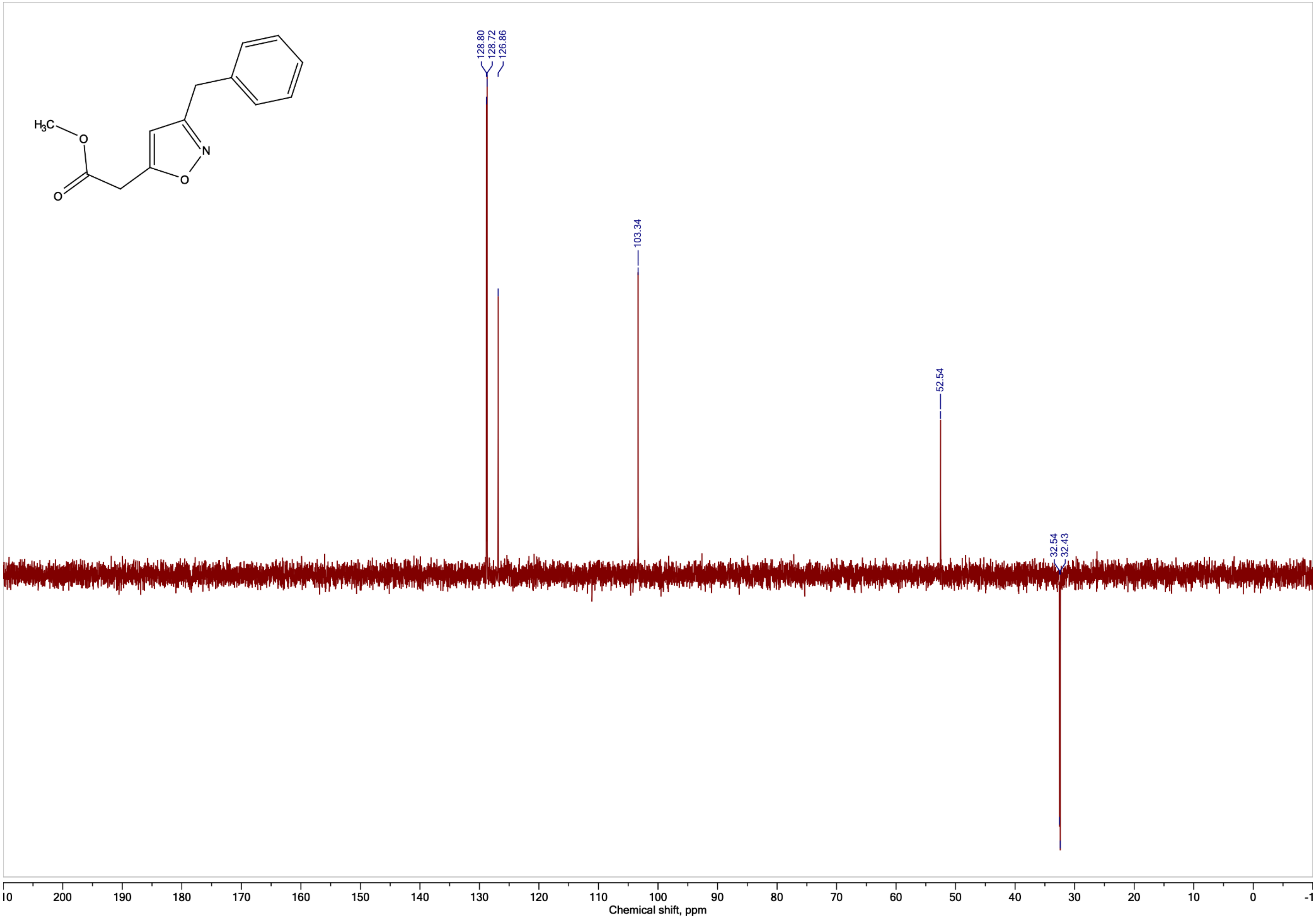

Methyl 2-(3-heptylisoxazol-5-yl)acetate (11j), ^{1}H NMR, $CDCl_3$, 400 MHz

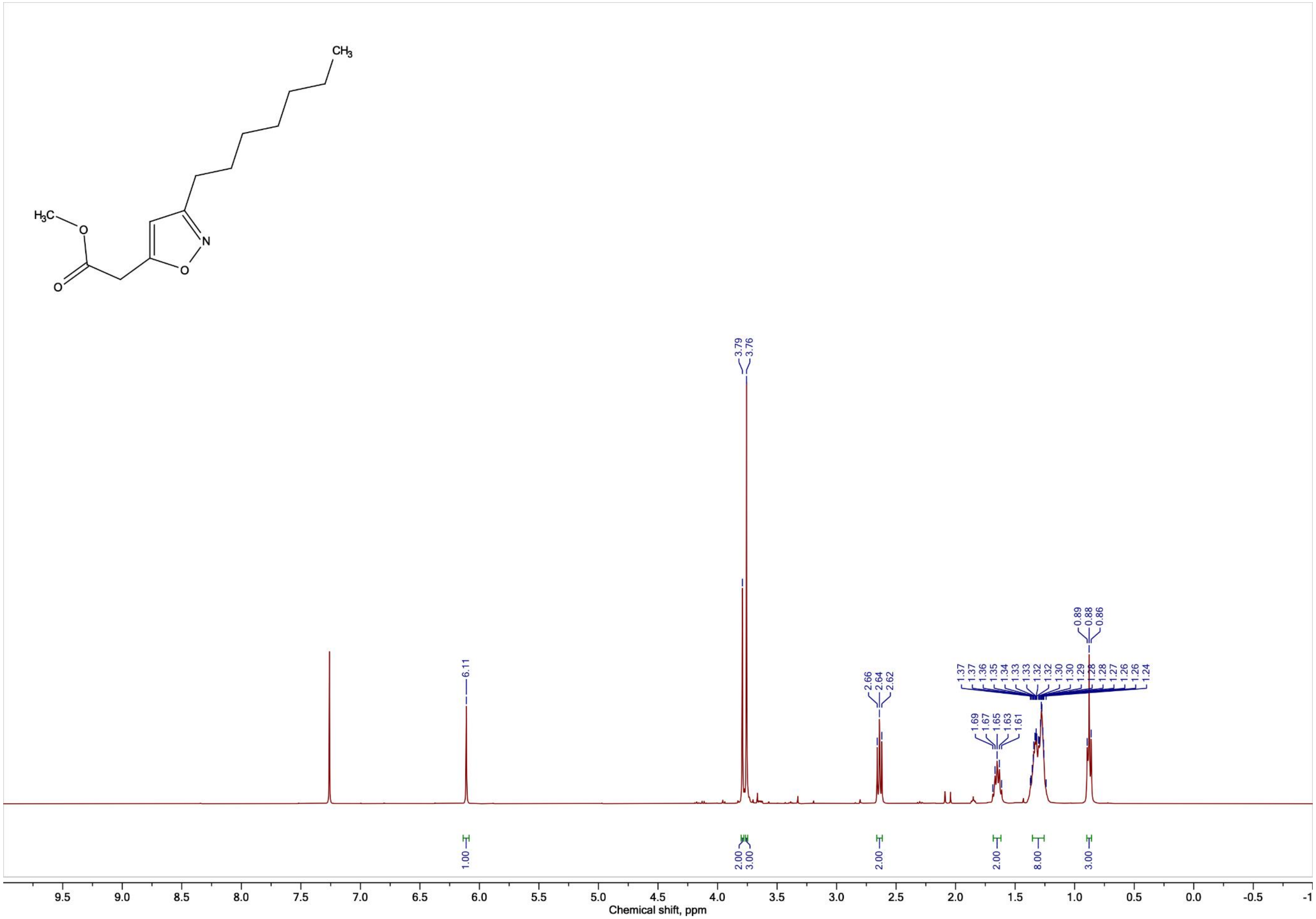

Methyl 2-(3-heptylisoxazol-5-yl)acetate (11j), $^{13}C\{^1H\}$ NMR, $CDCl_3$, 101 MHz

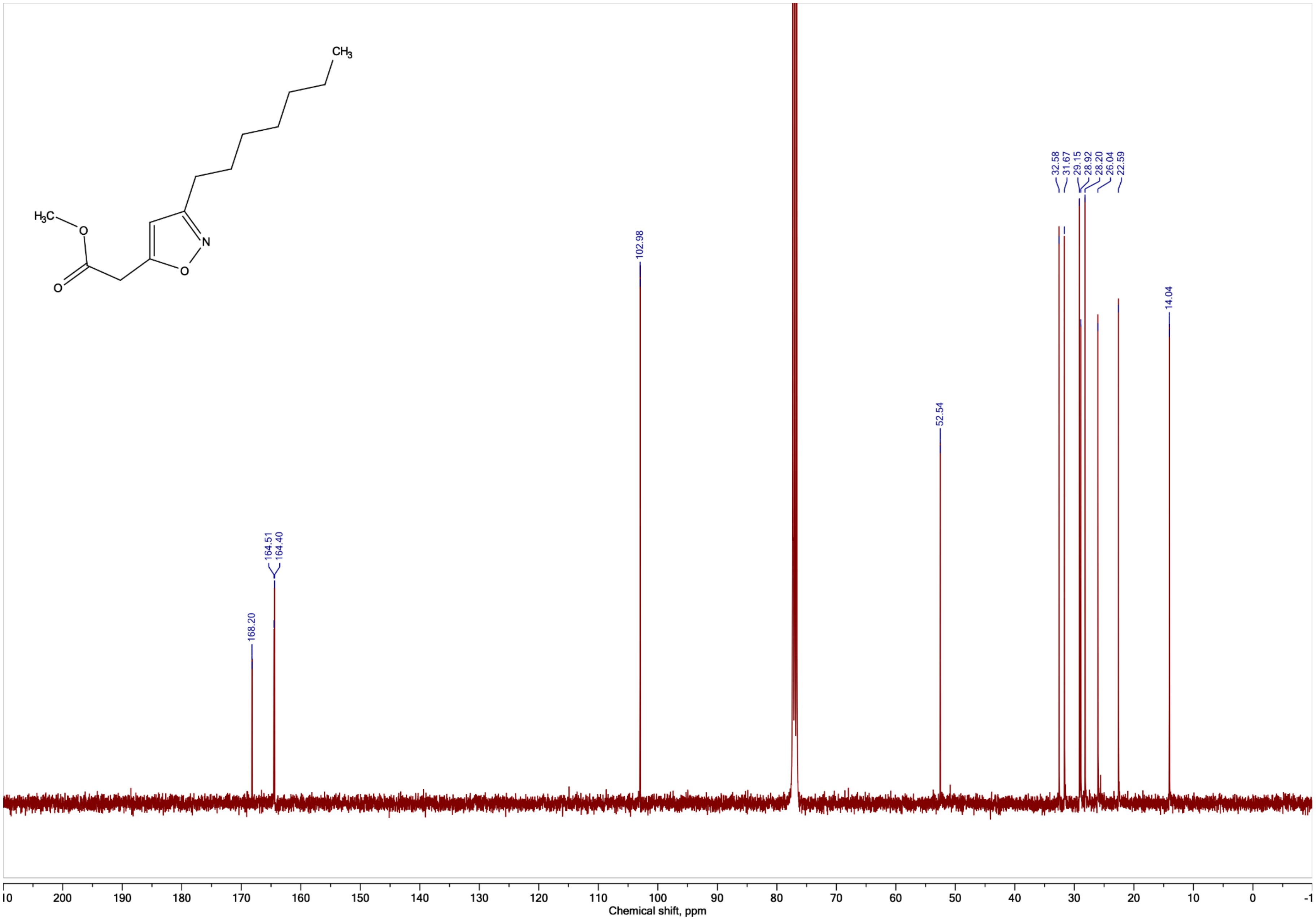

Methyl 2-(3-heptylisoxazol-5-yl)acetate (11j), DEPT, $CDCl_3$, 101 MHz

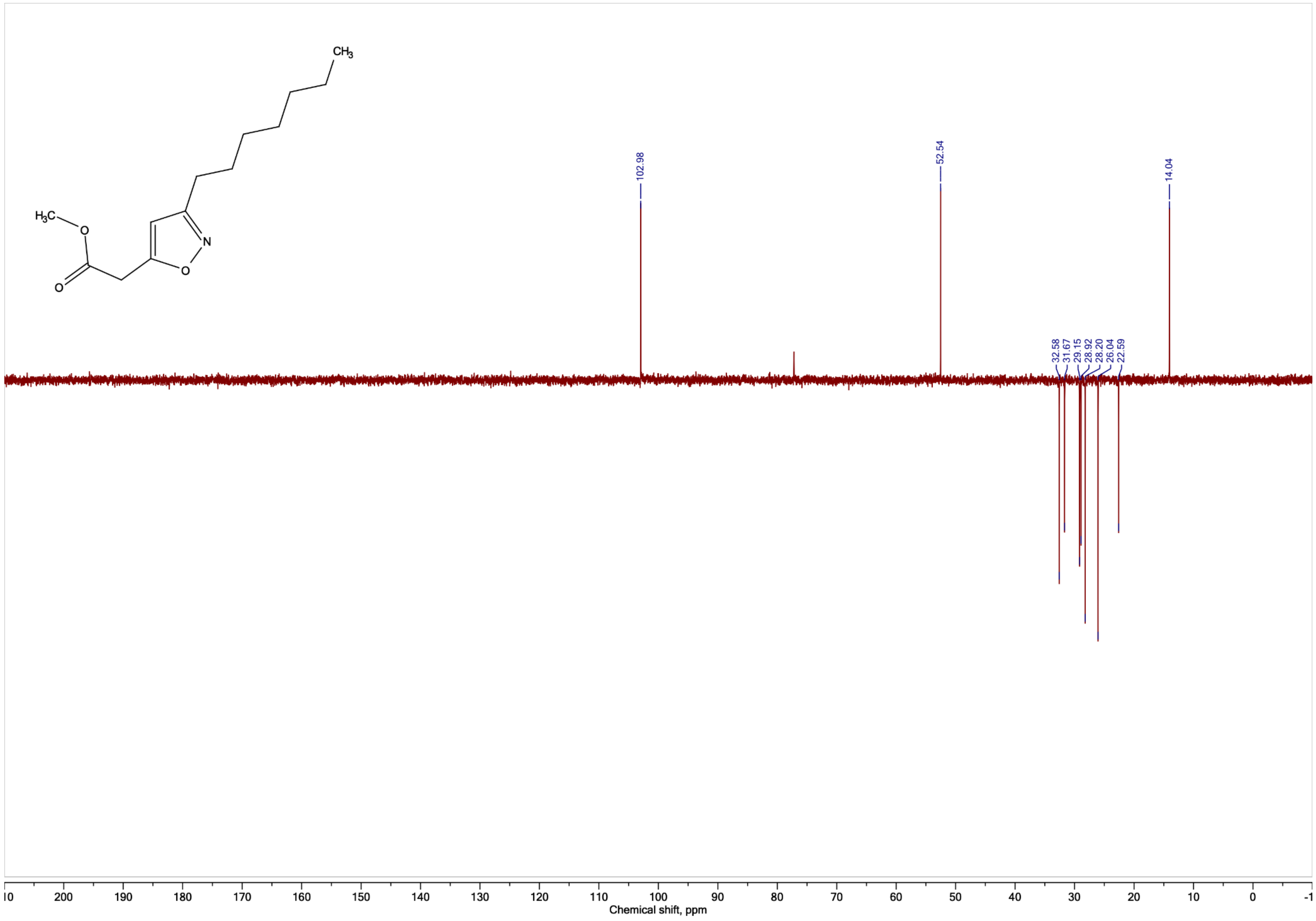

Methyl 2-(4-iodo-3-phenylisoxazol-5-yl)acetate (12a), ^{1}H NMR, $CDCl_3$, 400 MHz

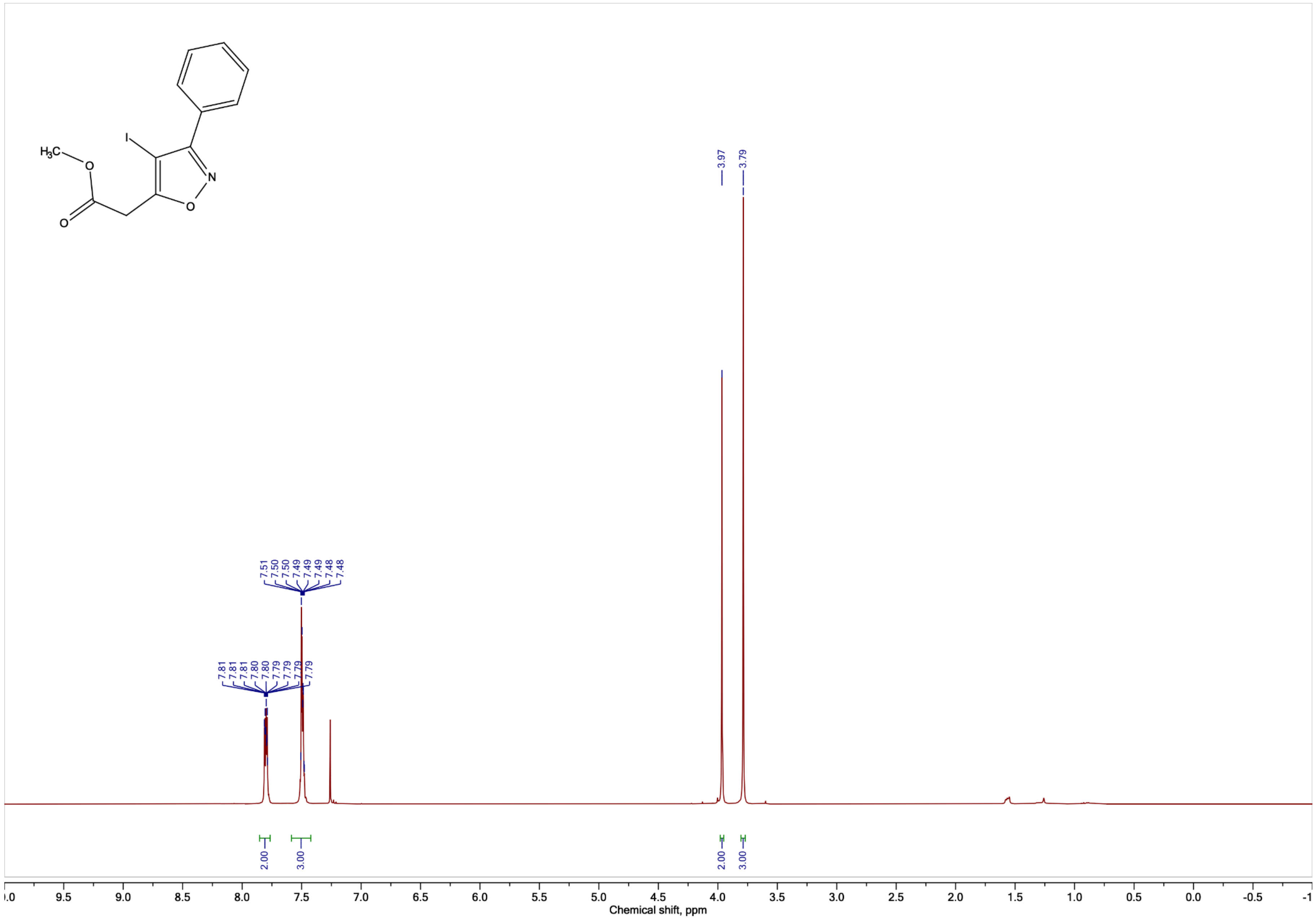

Methyl 2-(4-iodo-3-phenylisoxazol-5-yl)acetate (12a), $^{13}C\{^{1}H\}$ NMR, $CDCl_3$, 101 MHz

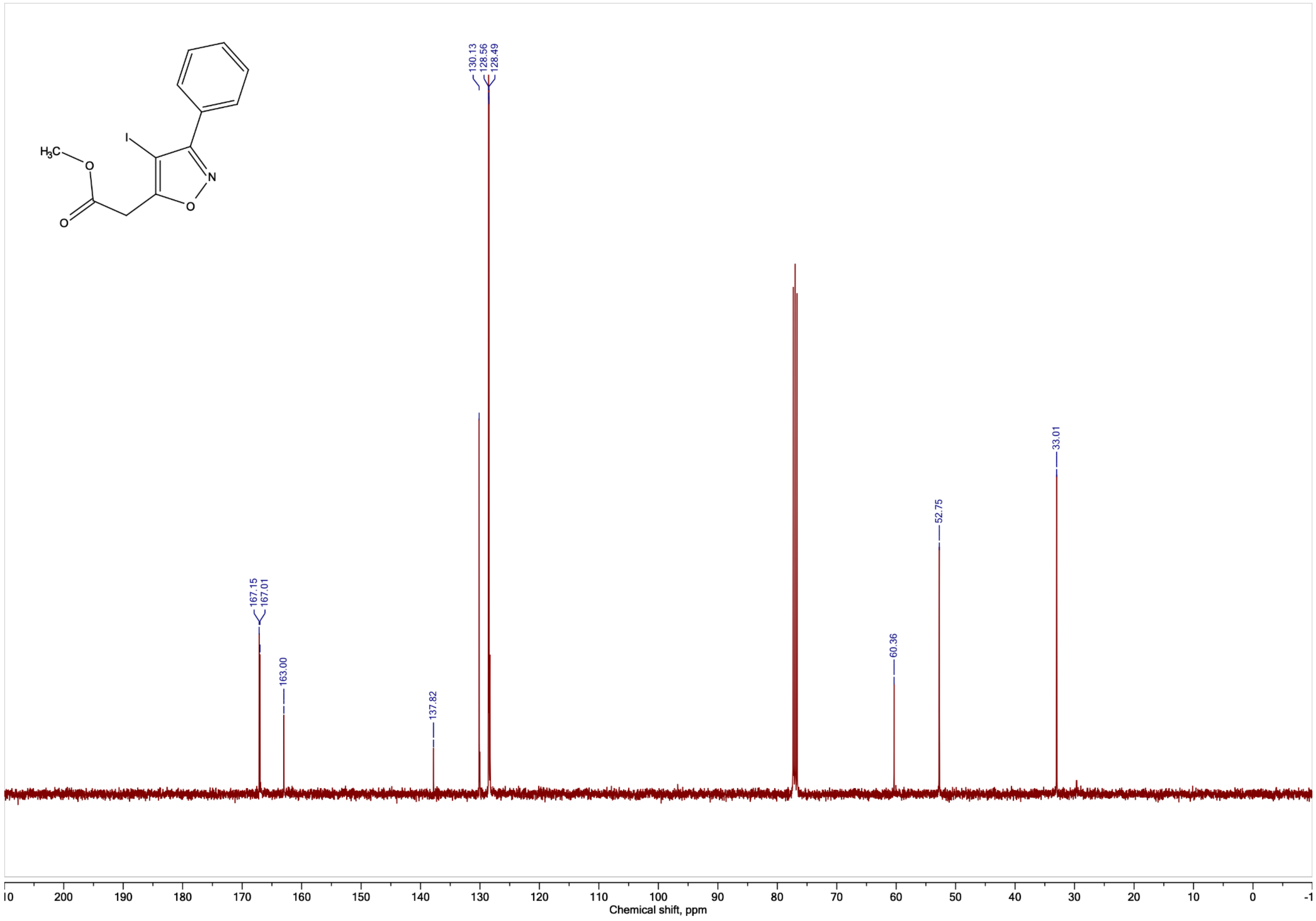

Methyl 2-(4-iodo-3-phenylisoxazol-5-yl)acetate (12a), DEPT, $CDCl_3$, 101 MHz

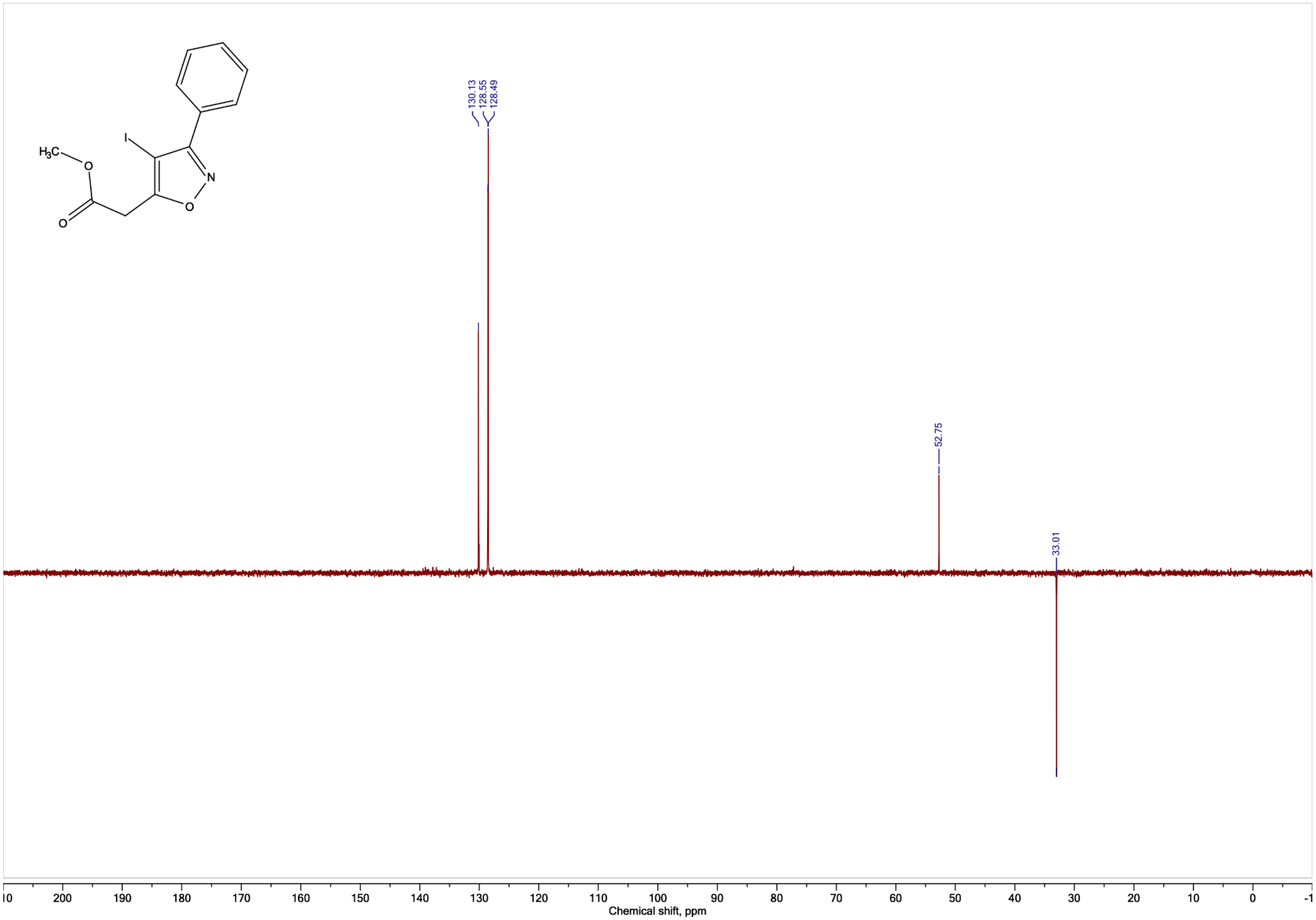

Methyl 2-(3-(4-(*tert*-butyl)phenyl)-4-iodoisoxazol-5-yl)acetate (12b), ^{1}H NMR, $CDCl_3$, 400 MHz

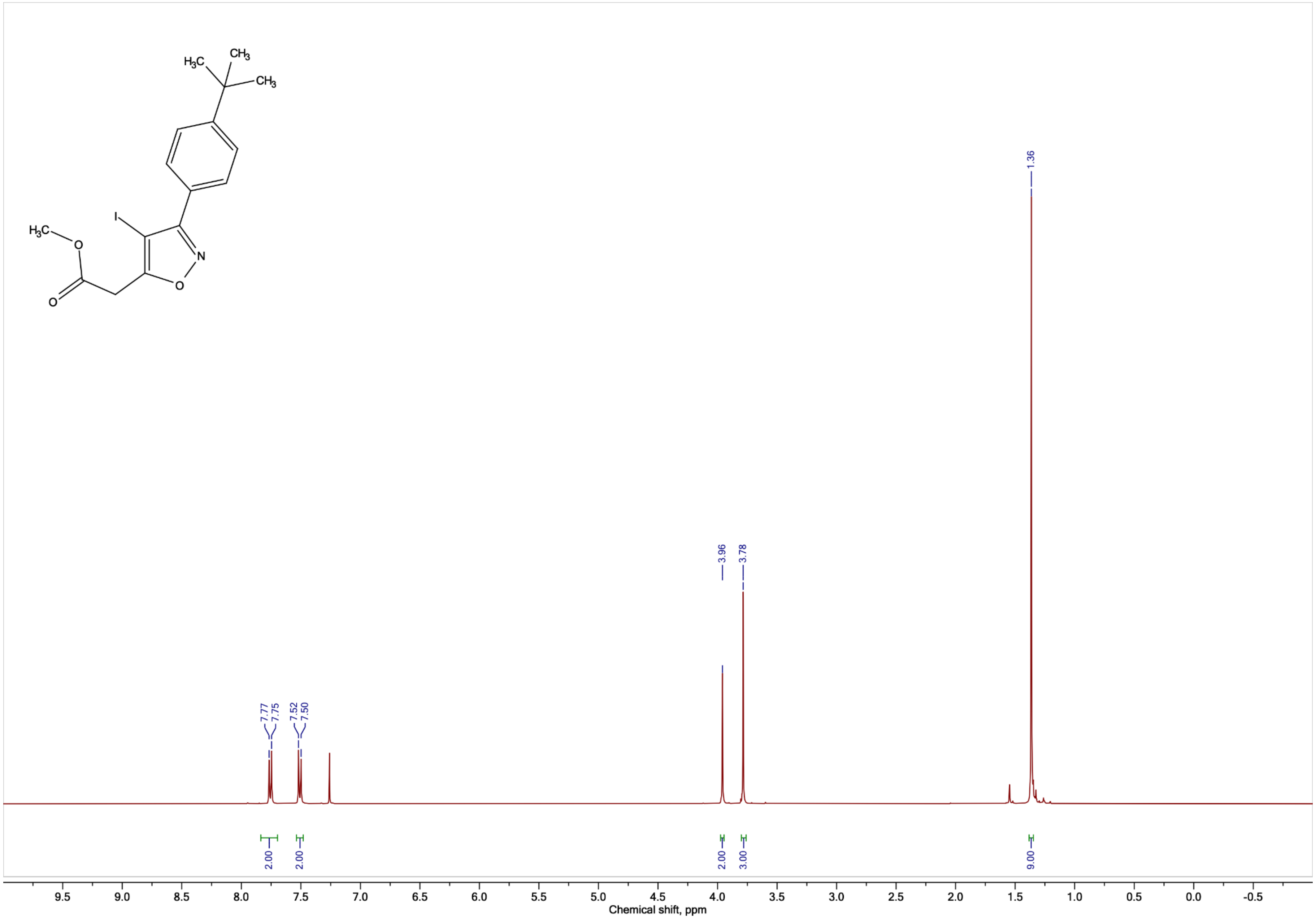

Methyl 2-(3-(4-(*tert*-butyl)phenyl)-4-iodoisoxazol-5-yl)acetate (12b), $^{13}C\{^{1}H\}$ NMR, $CDCl_3$, 101 MHz

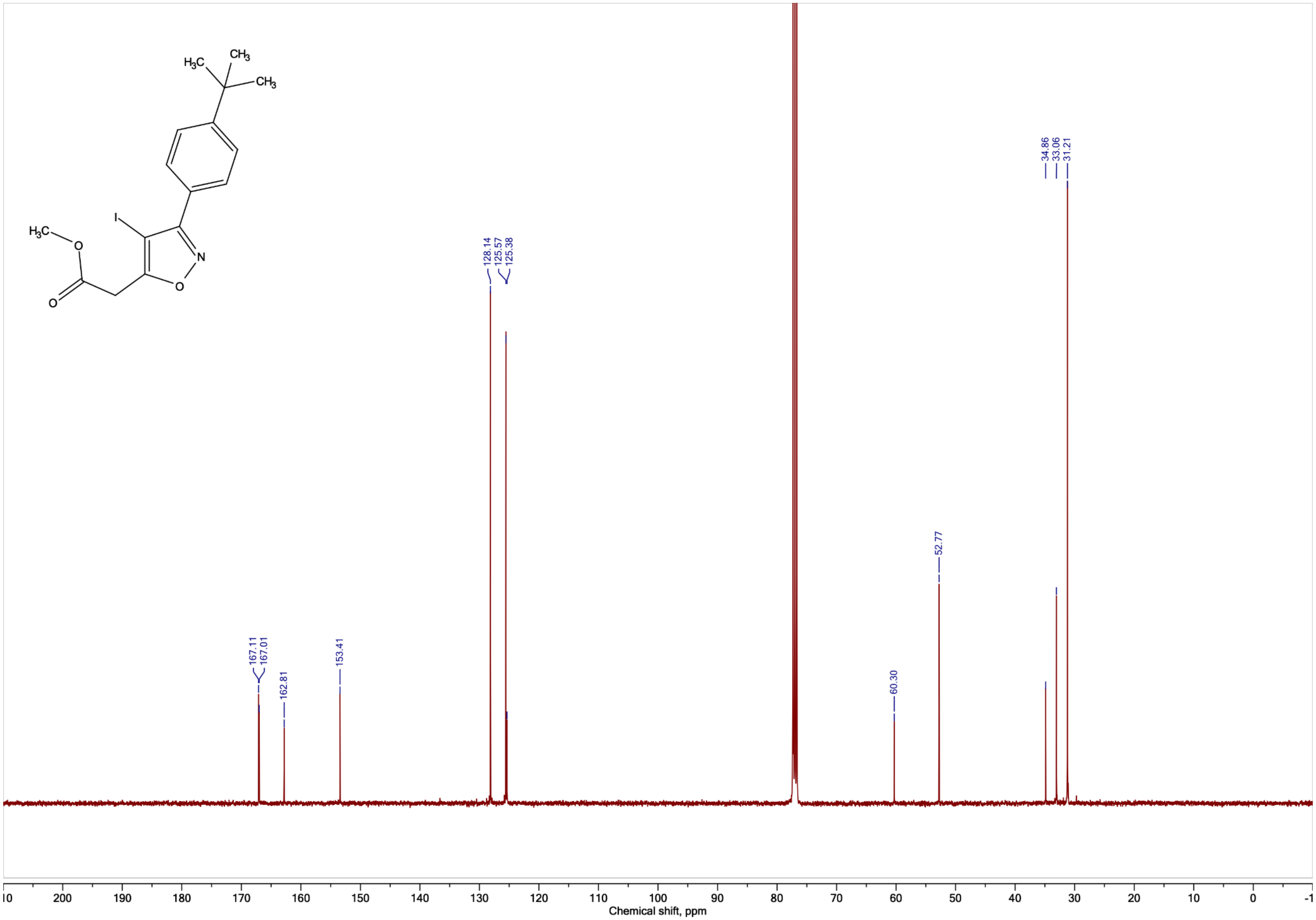

Methyl 2-(3-(4-(*tert*-butyl)phenyl)-4-iodoisoxazol-5-yl)acetate (12b), DEPT, $CDCl_3$, 101 MHz

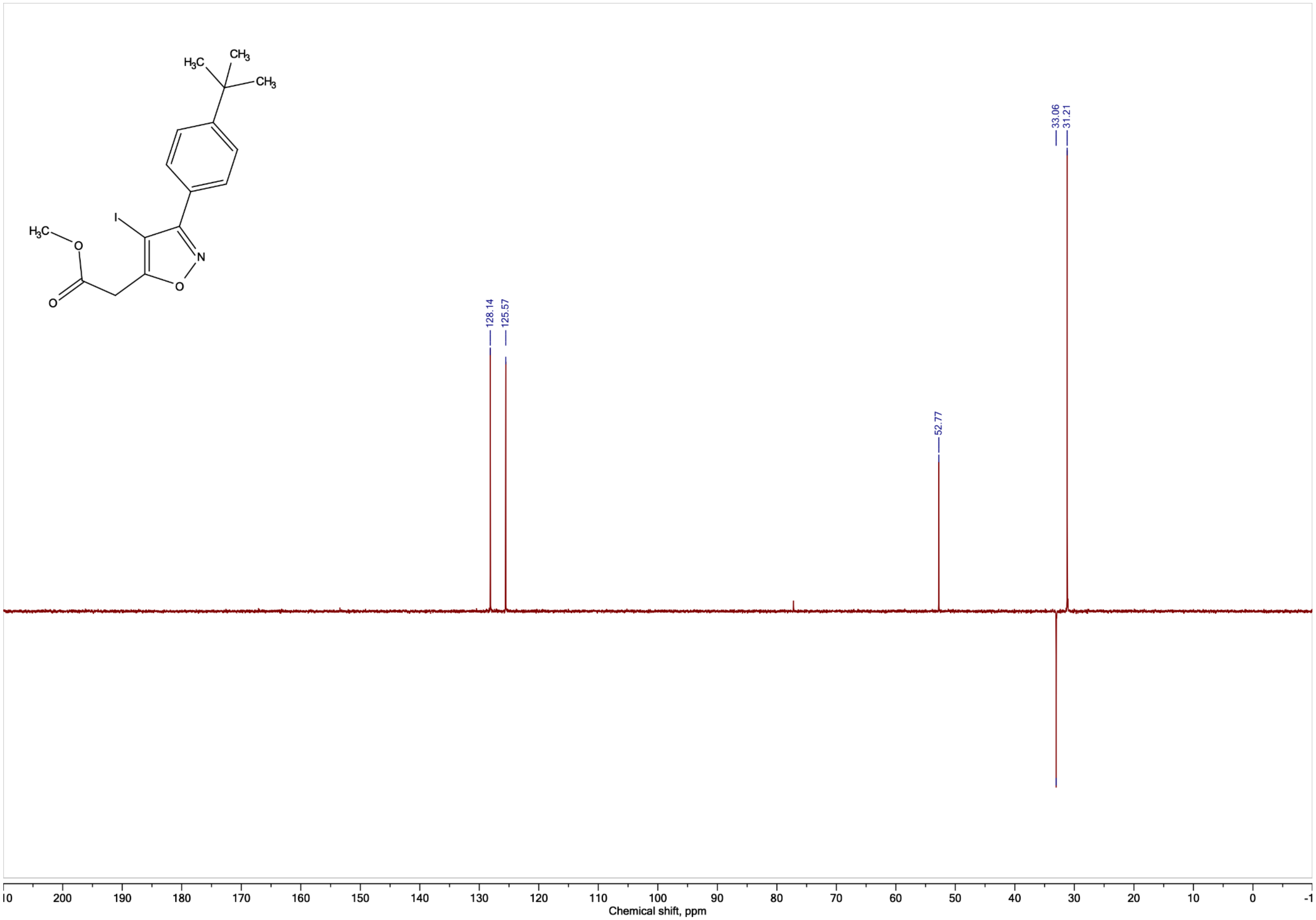

Methyl 2-(4-iodo-3-(4-methoxyphenyl)isoxazol-5-yl)acetate (12c), ^{1}H NMR, $CDCl_3$, 400 MHz

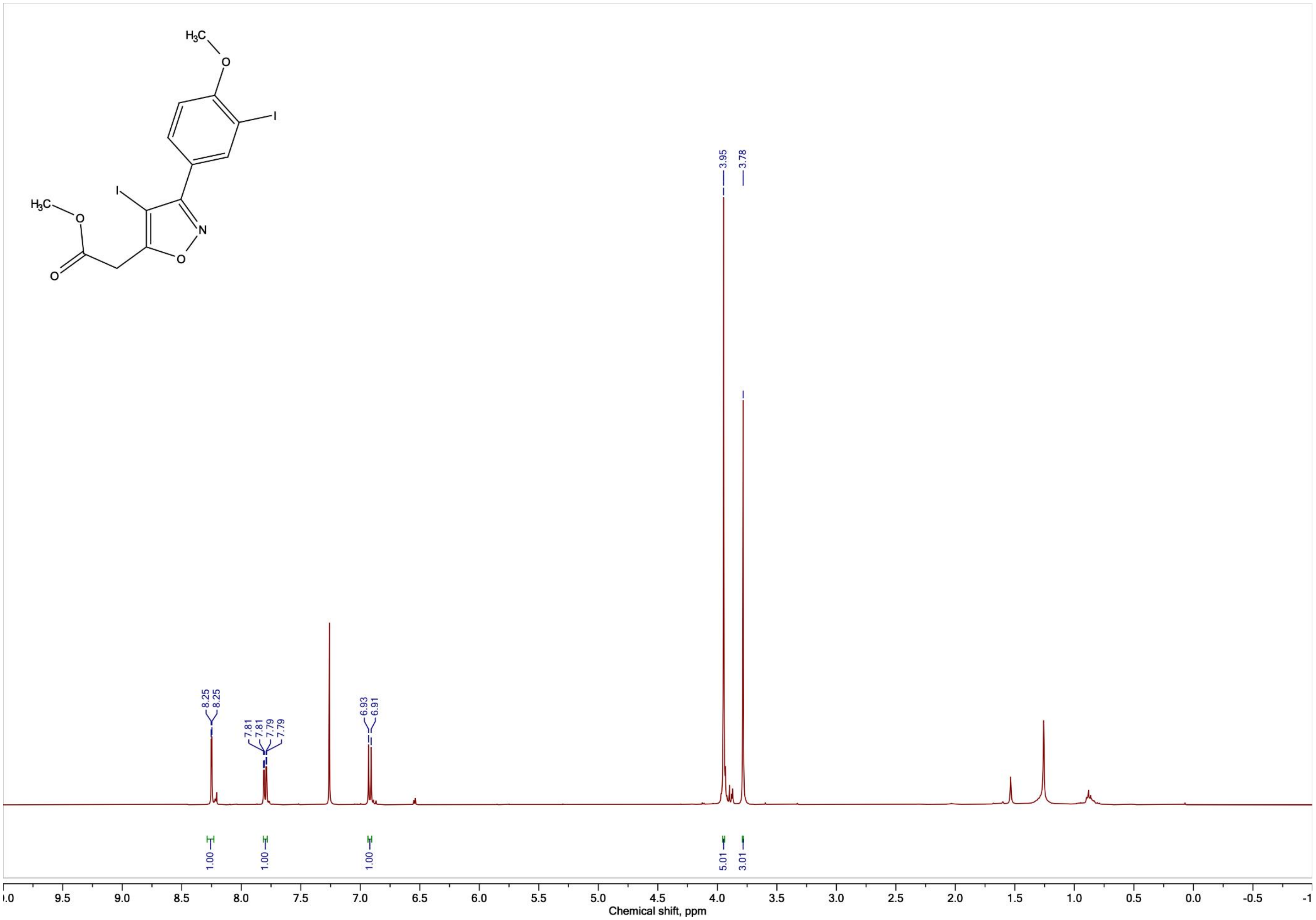

Methyl 2-(4-iodo-3-(4-methoxyphenyl)isoxazol-5-yl)acetate (12c), $^{13}C\{^{1}H\}$ NMR, $CDCl_3$, 101 MHz

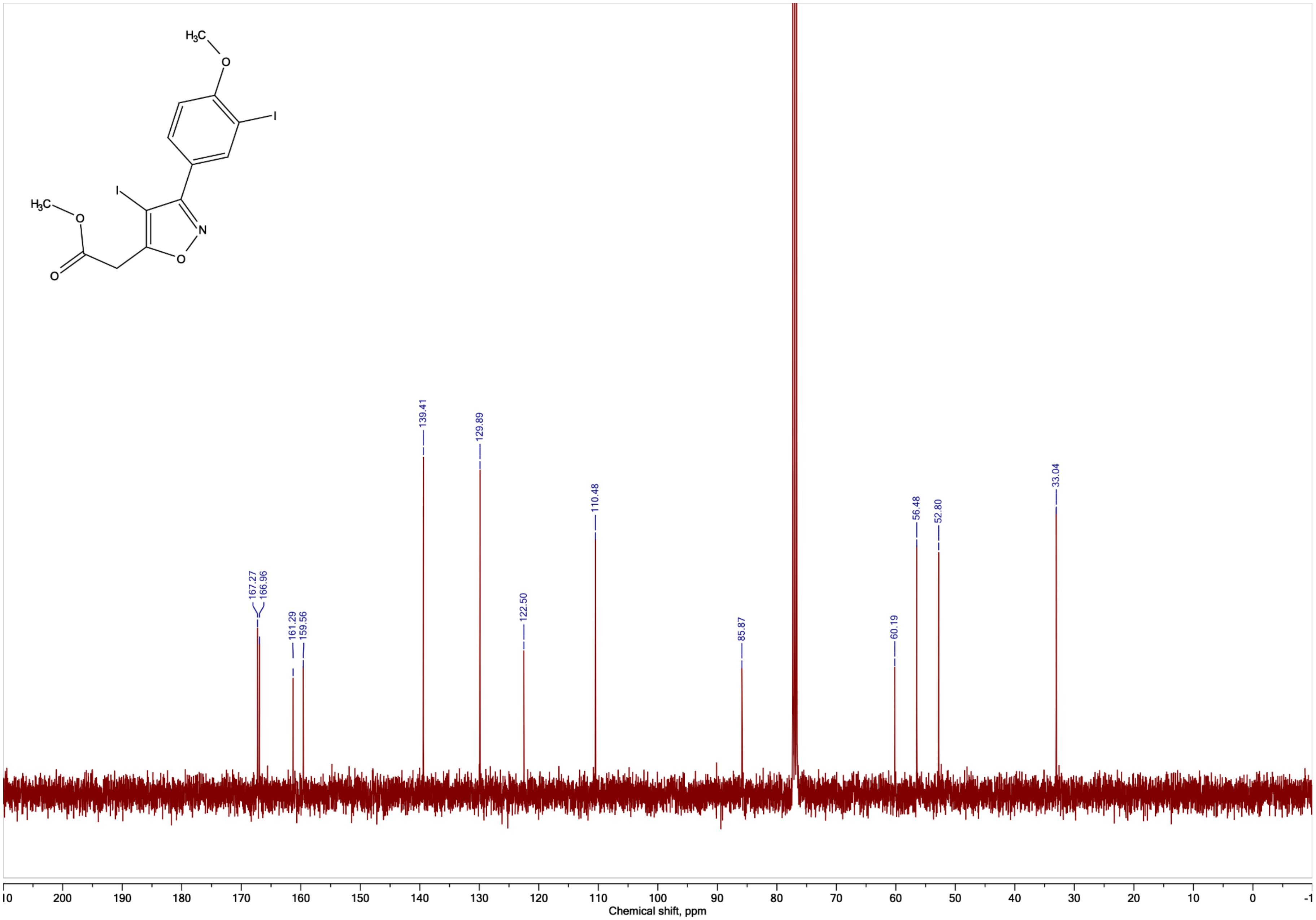

Methyl 2-(4-iodo-3-(4-methoxyphenyl)isoxazol-5-yl)acetate (12c), DEPT, $CDCl_3$, 101 MHz

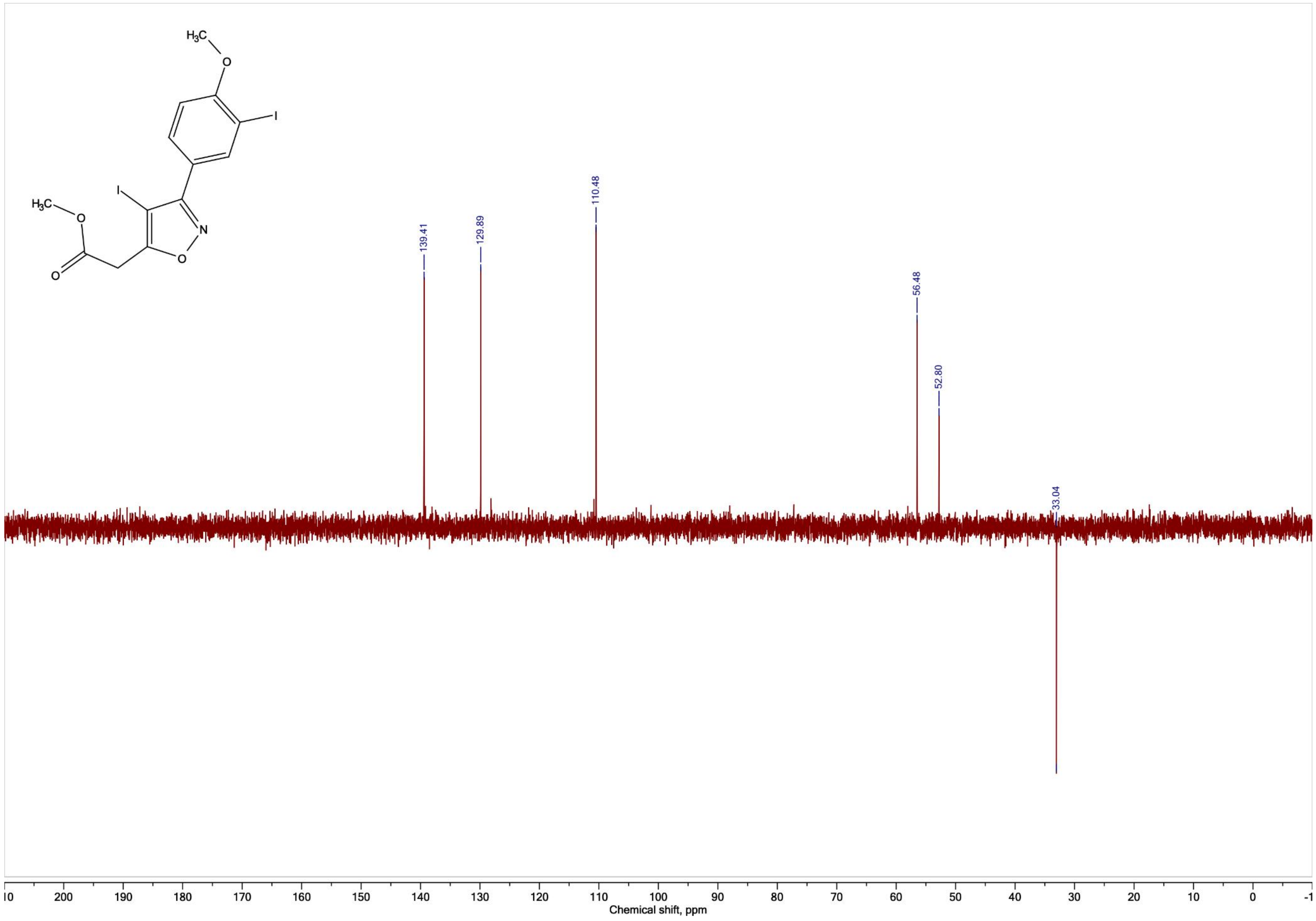

Methyl 2-(3-(4-chlorophenyl)-4-iodoisoxazol-5-yl)acetate (12d), ^{1}H NMR, $CDCl_3$, 400 MHz

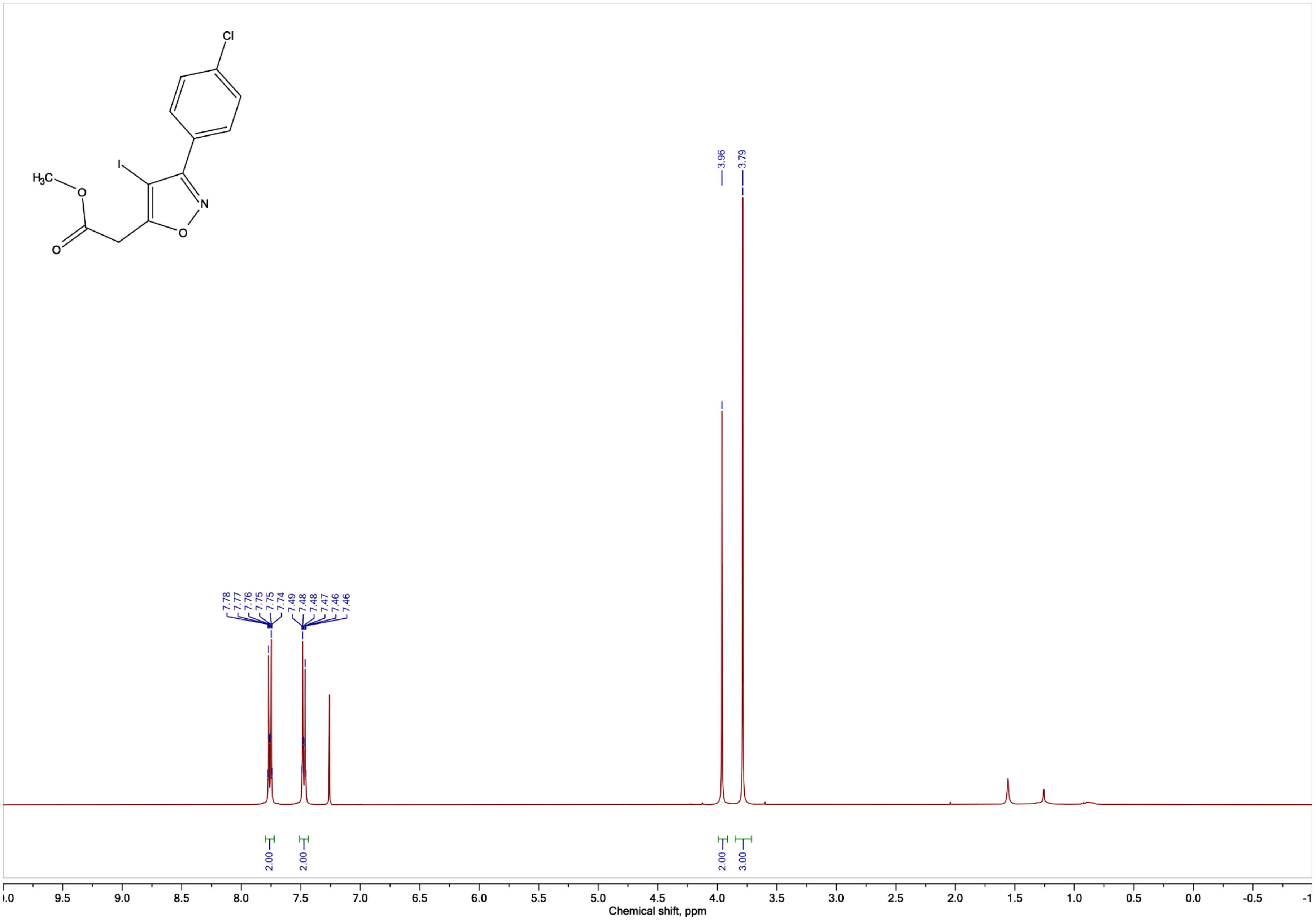

Methyl 2-(3-(4-chlorophenyl)-4-iodoisoxazol-5-yl)acetate (12d), $^{13}C\{^1H\}$ NMR, $CDCl_3$, 101 MHz

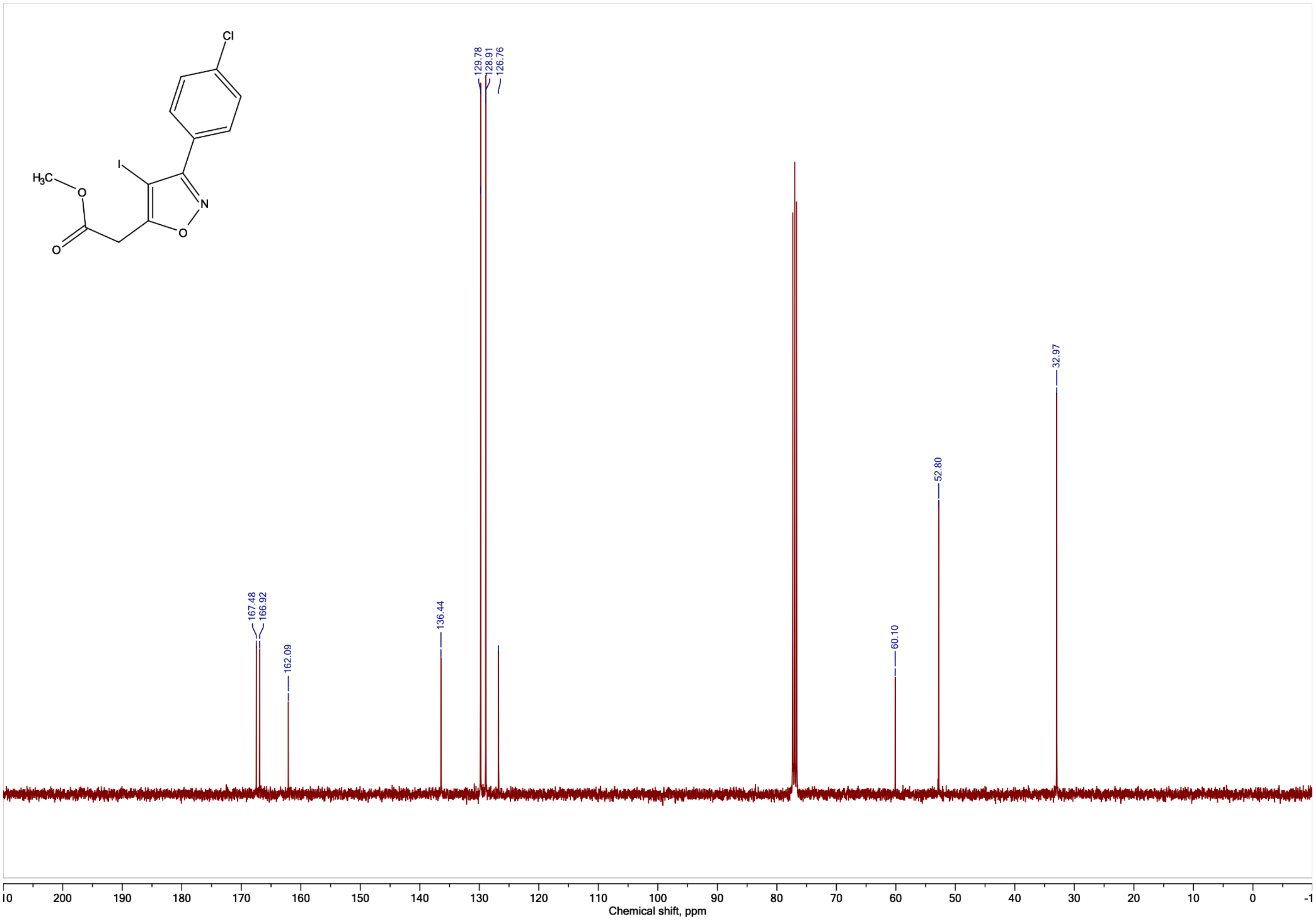

Methyl 2-(3-(4-chlorophenyl)-4-iodoisoxazol-5-yl)acetate (12d), DEPT, $CDCl_3$, 101 MHz

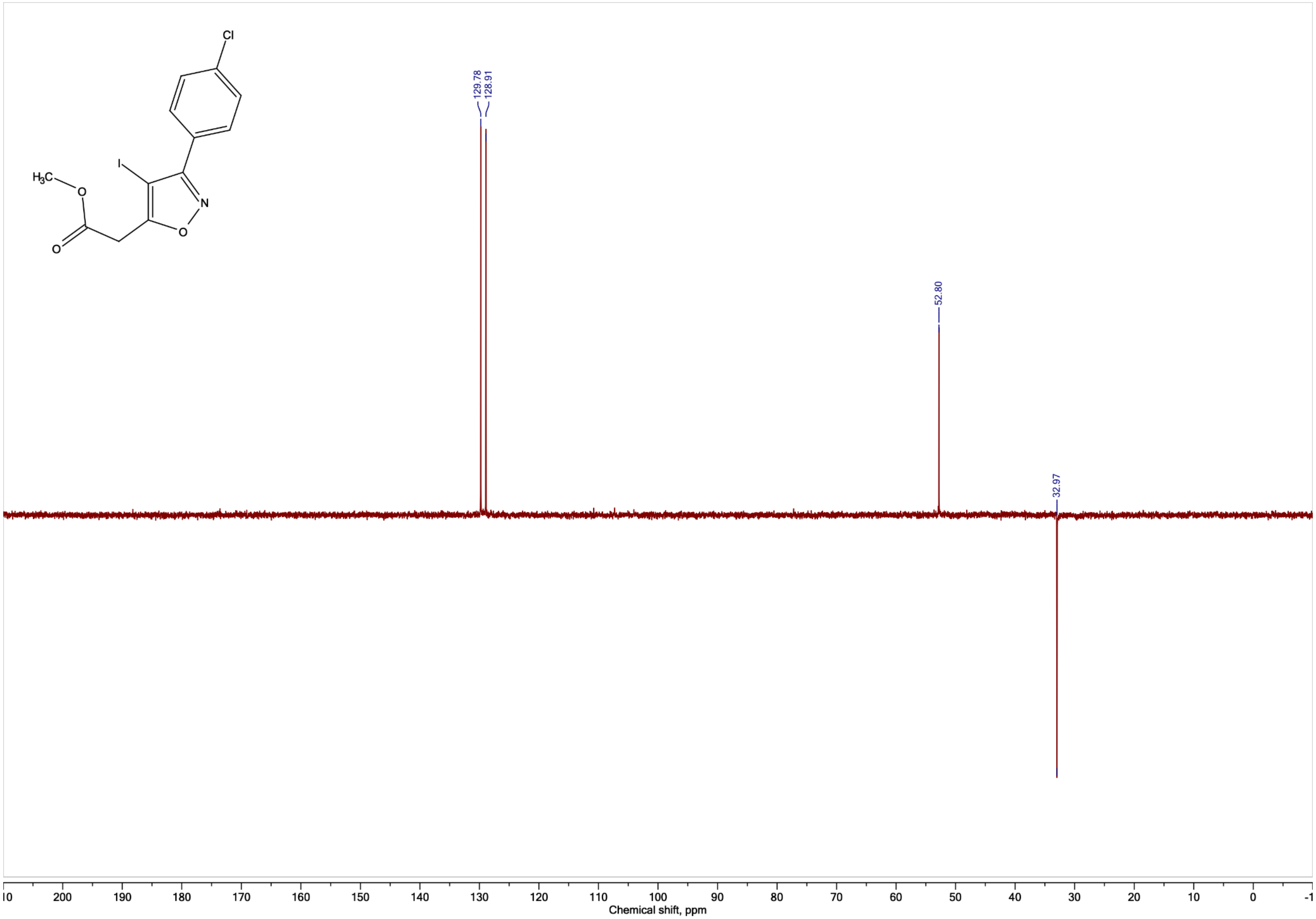

Methyl 2-(3-(4-bromophenyl)-4-iodoisoxazol-5-yl)acetate (12e), ^{1}H NMR, $CDCl_3$, 400 MHz

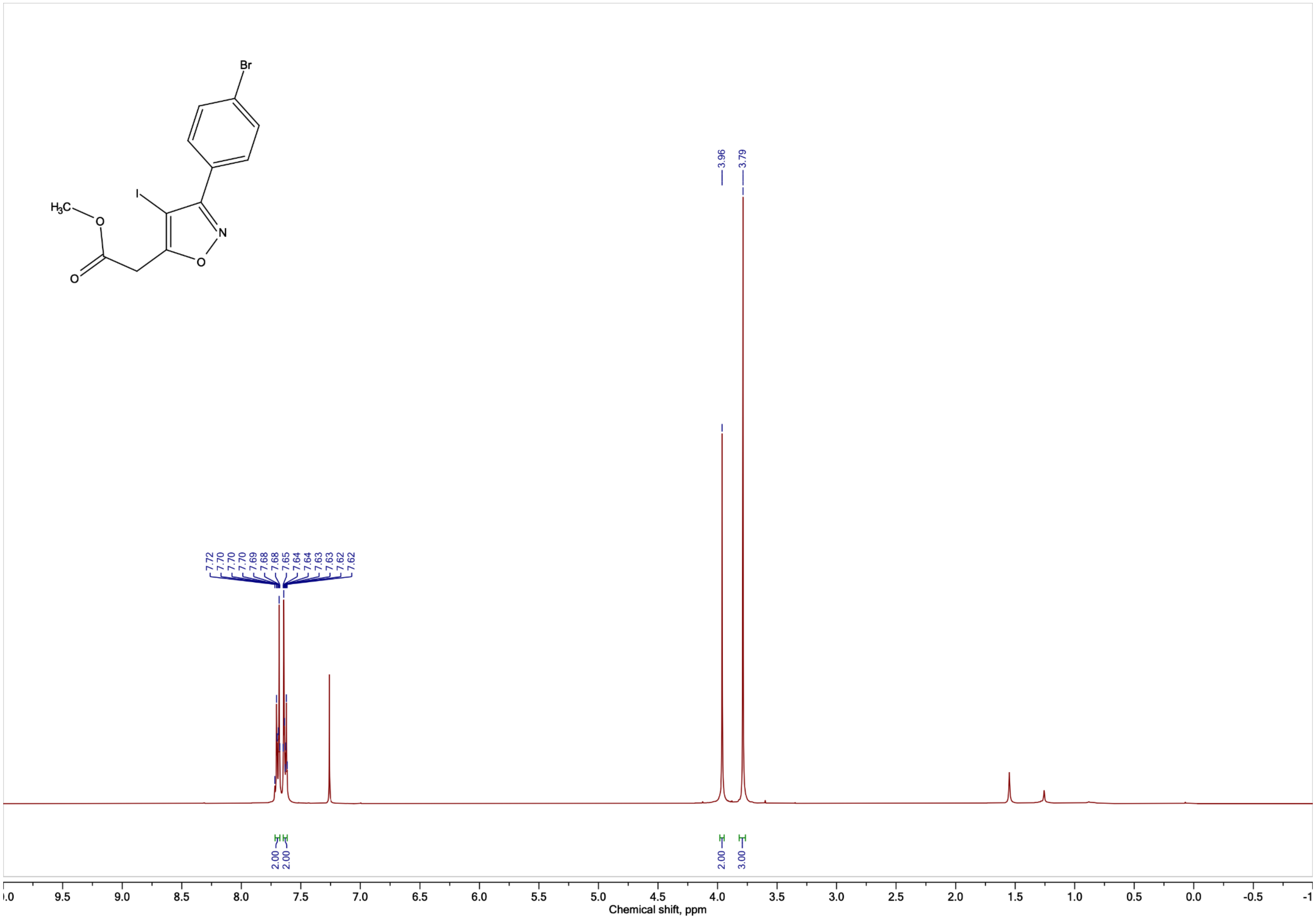

Methyl 2-(3-(4-bromophenyl)-4-iodoisoxazol-5-yl)acetate (12e), $^{13}C\{^{1}H\}$ NMR, $CDCl_3$, 101 MHz

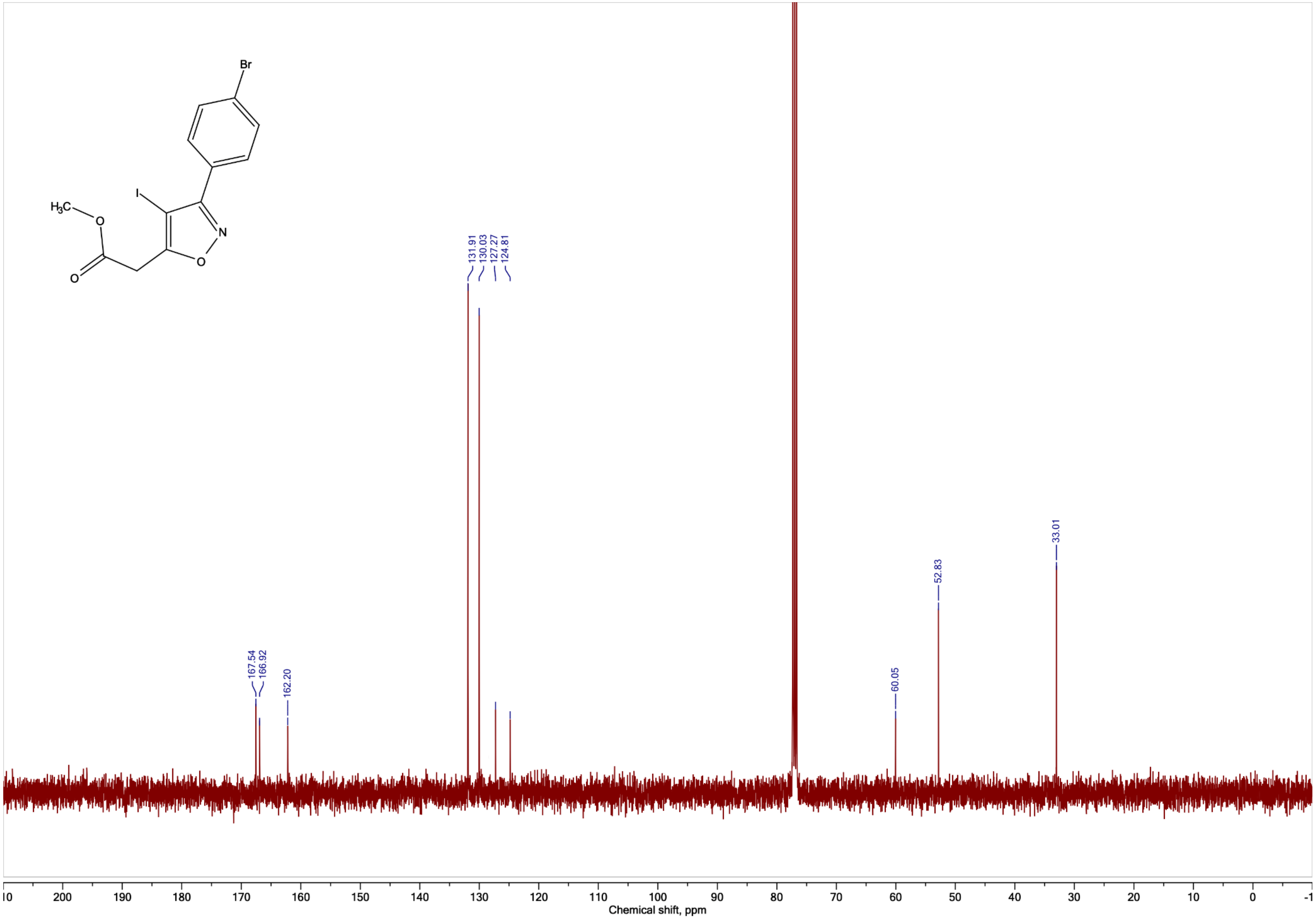

Methyl 2-(3-(4-bromophenyl)-4-iodoisoxazol-5-yl)acetate (12e), DEPT, $CDCl_3$, 101 MHz

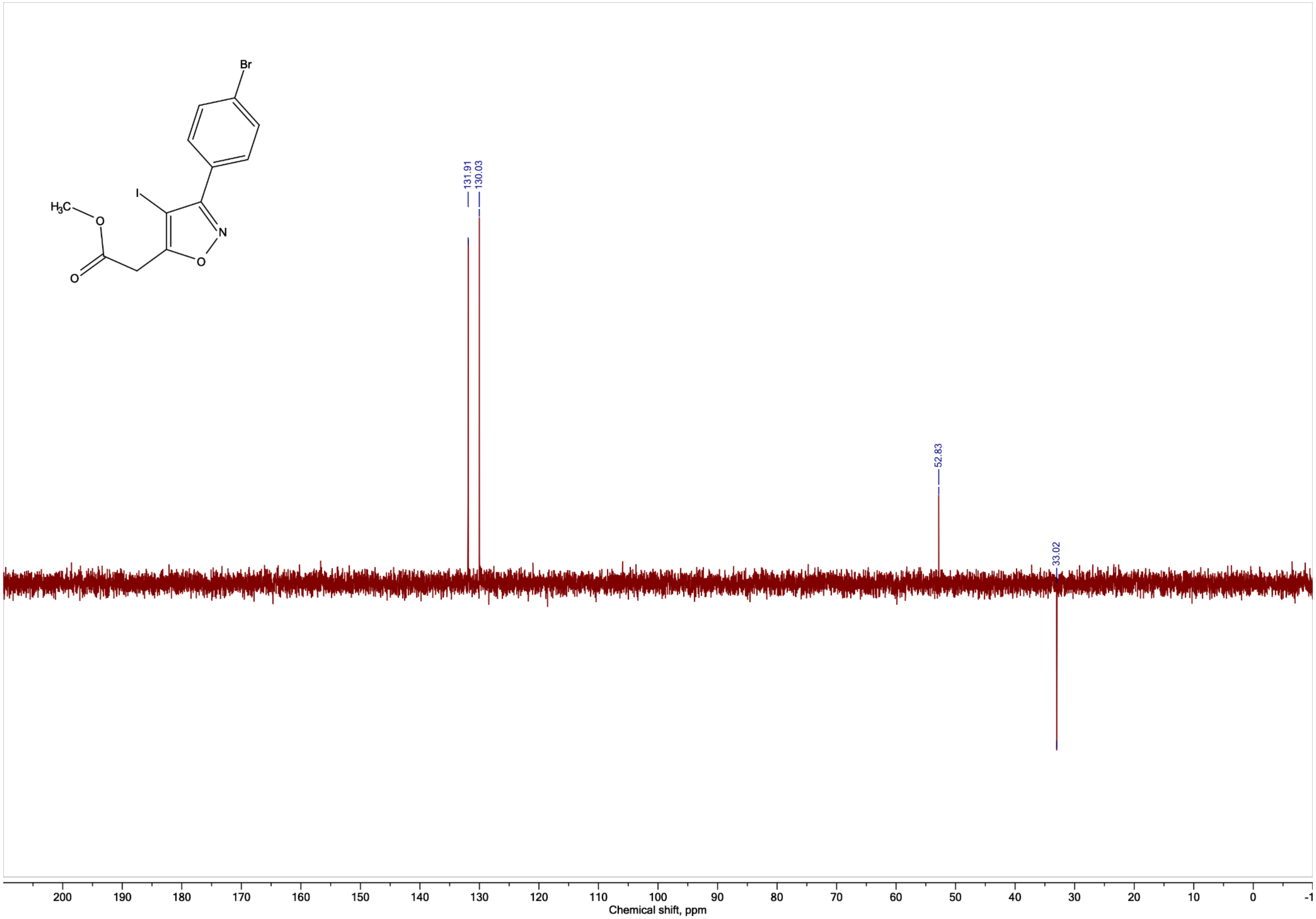

Methyl 2-(3-phenyl-4-(thiophen-3-yl)isoxazol-5-yl)acetate (13a), ^{1}H NMR, $CDCl_3$, 400 MHz

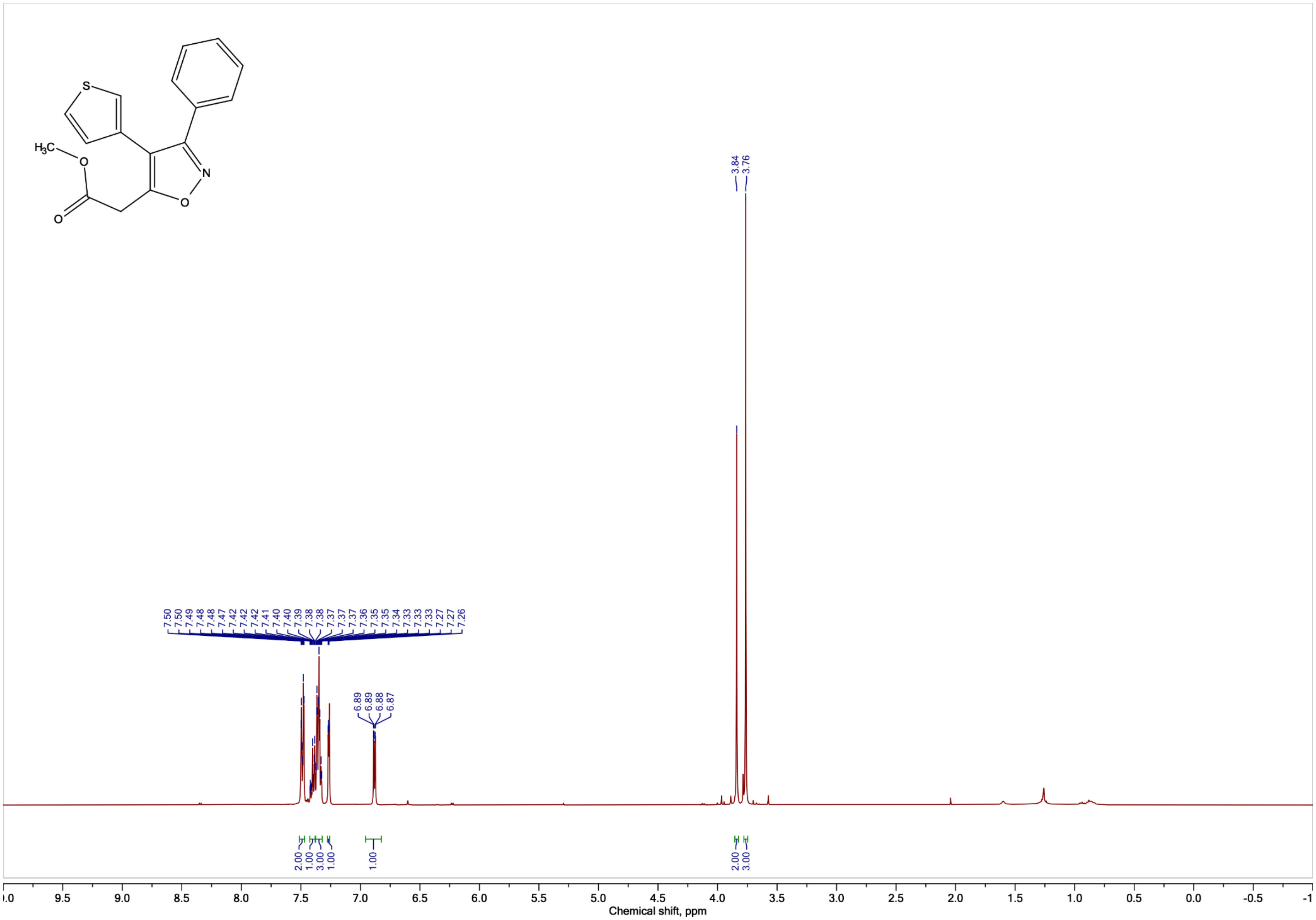

Methyl 2-(3-phenyl-4-(thiophen-3-yl)isoxazol-5-yl)acetate (13a), $^{13}C\{^1H\}$ NMR, $CDCl_3$, 101 MHz

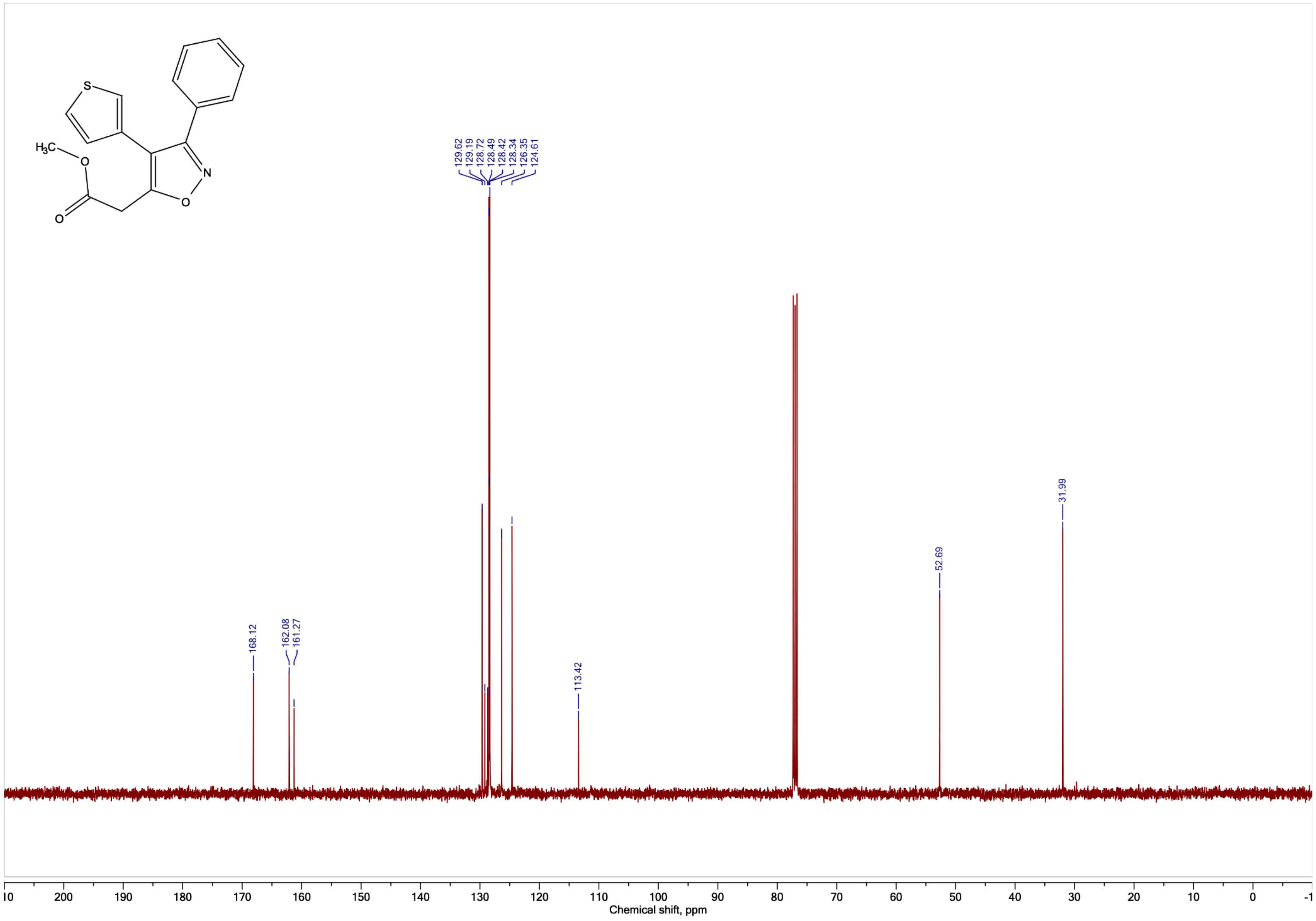

Methyl 2-(3-phenyl-4-(thiophen-3-yl)isoxazol-5-yl)acetate (13a), DEPT, $CDCl_3$, 101 MHz

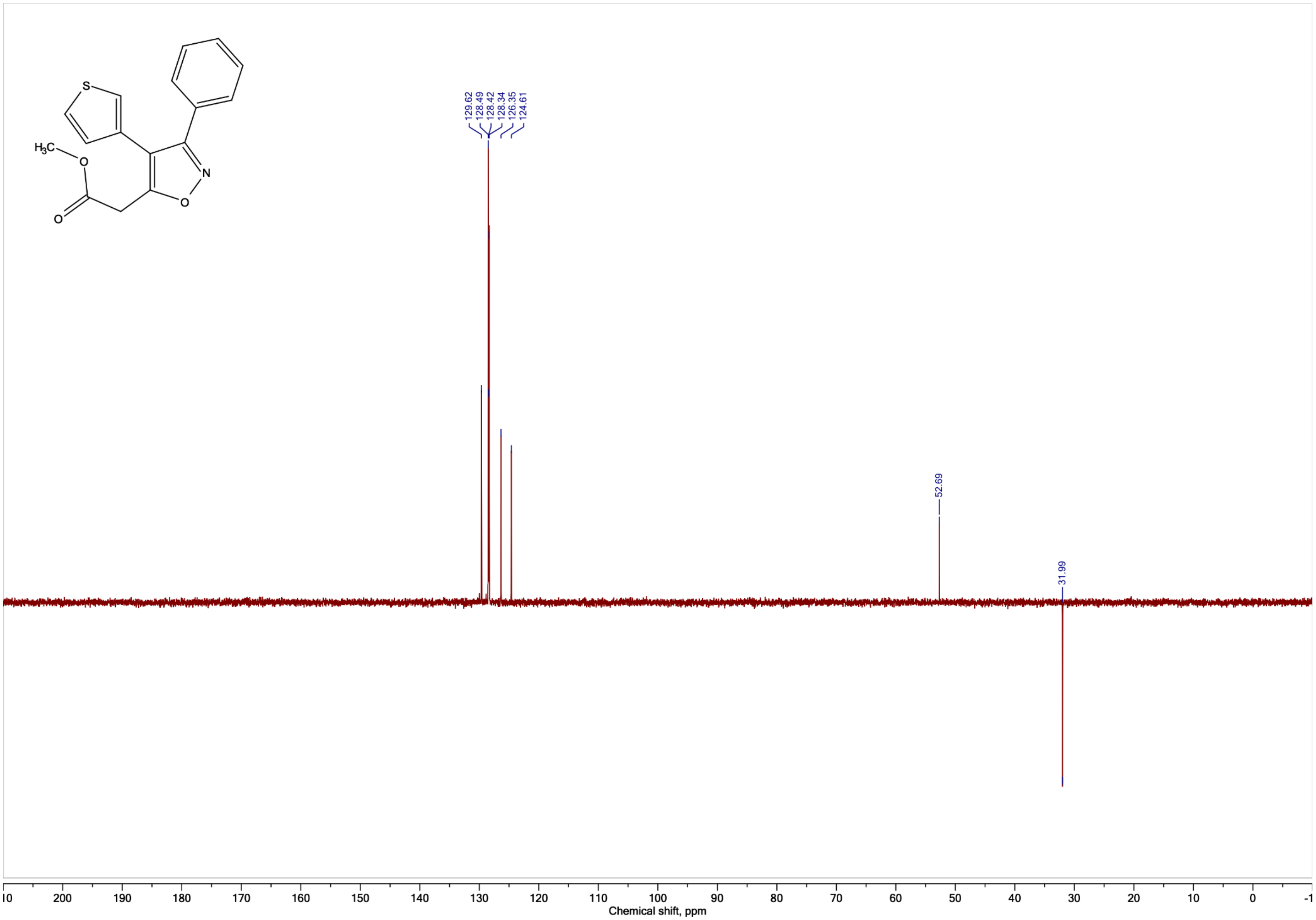

Methyl 2-(3-(4-(tert-butyl)phenyl)-4-(4-(trifluoromethyl)phenyl)isoxazol-5-yl)acetate (13b), ^{1}H NMR, CDCl$_3$, 400 MHz

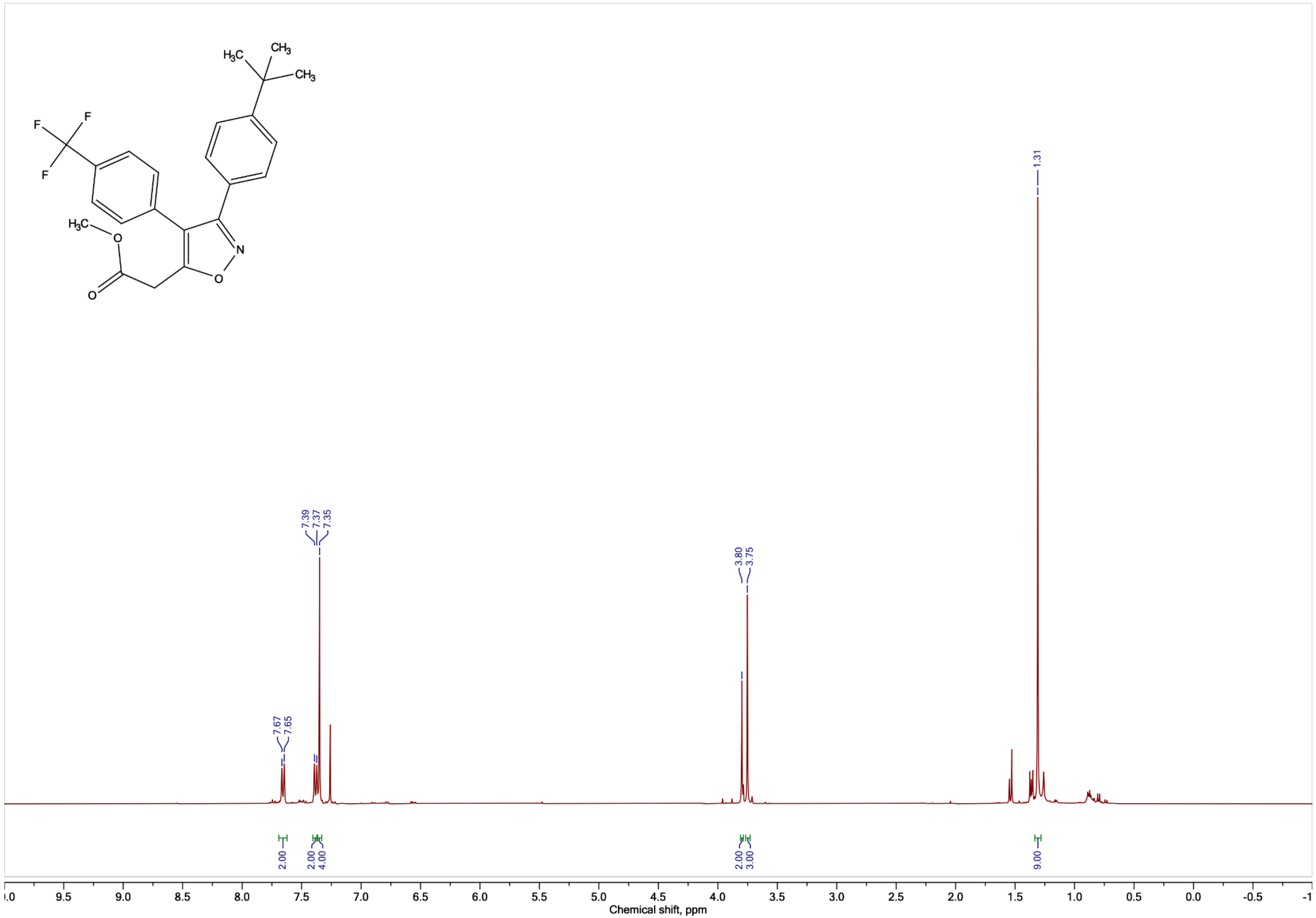

Methyl 2-(3-(4-(tert-butyl)phenyl)-4-(4-(trifluoromethyl)phenyl)isoxazol-5-yl)acetate (13b), $^{13}C\{^1H\}$ NMR, $CDCl_3$, 101 MHz

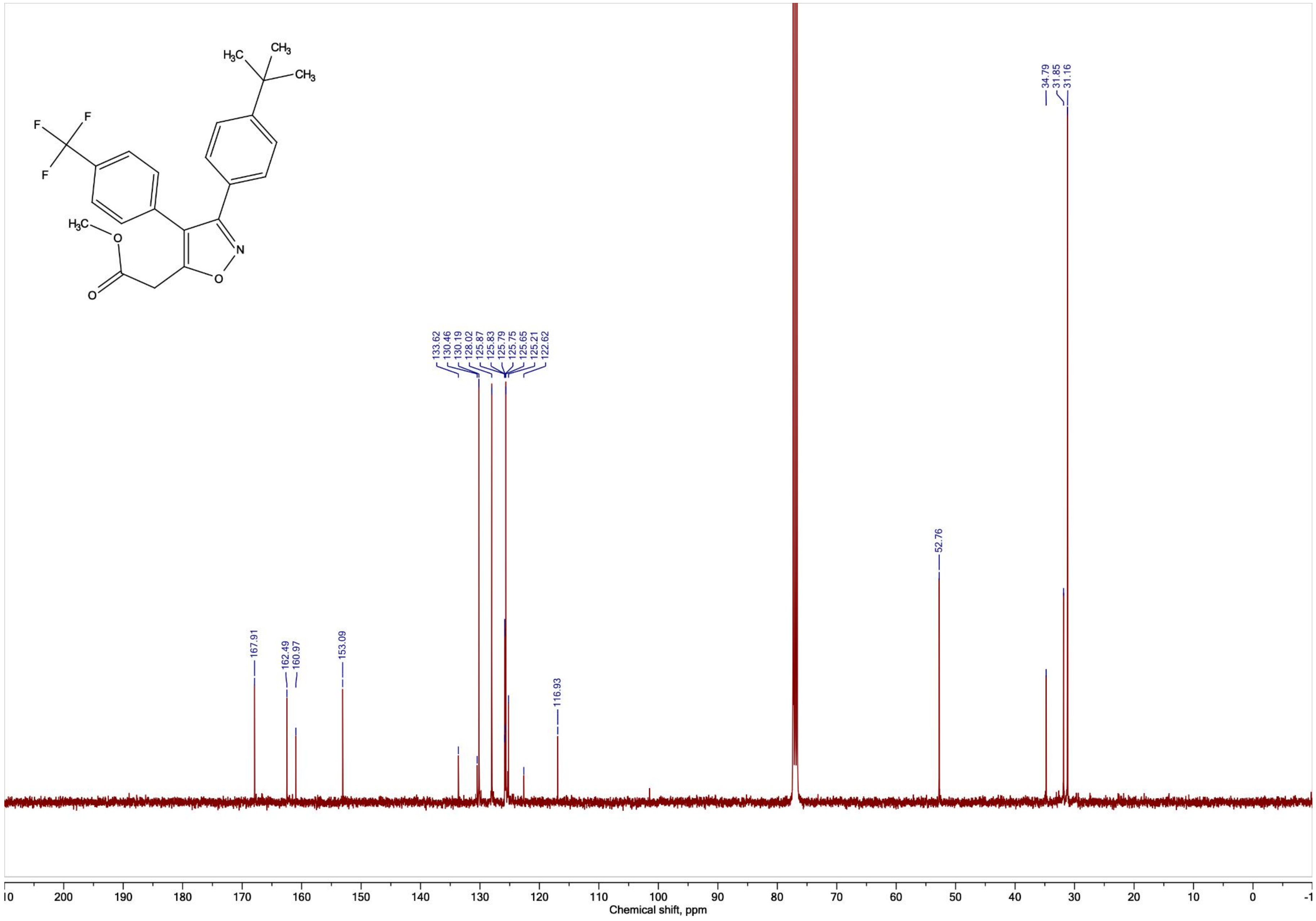

Methyl 2-(3-(4-(tert-butyl)phenyl)-4-(4-(trifluoromethyl)phenyl)isoxazol-5-yl)acetate (13b), DEPT, $CDCl_3$, 101 MHz

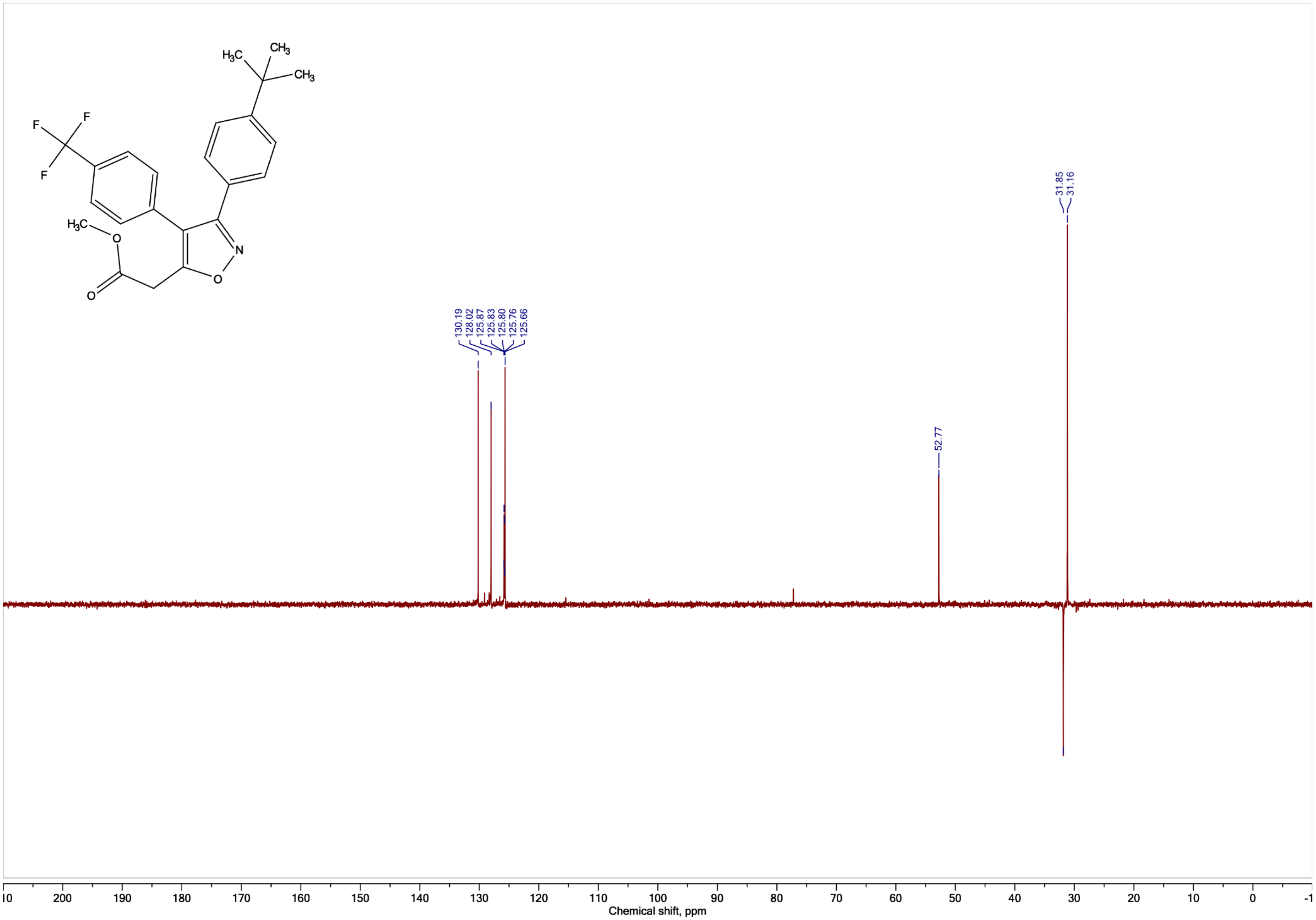

Methyl 2-(3-(4-chlorophenyl)-4-phenylisoxazol-5-yl)acetate (13c), ^{1}H NMR, $CDCl_3$, 400 MHz

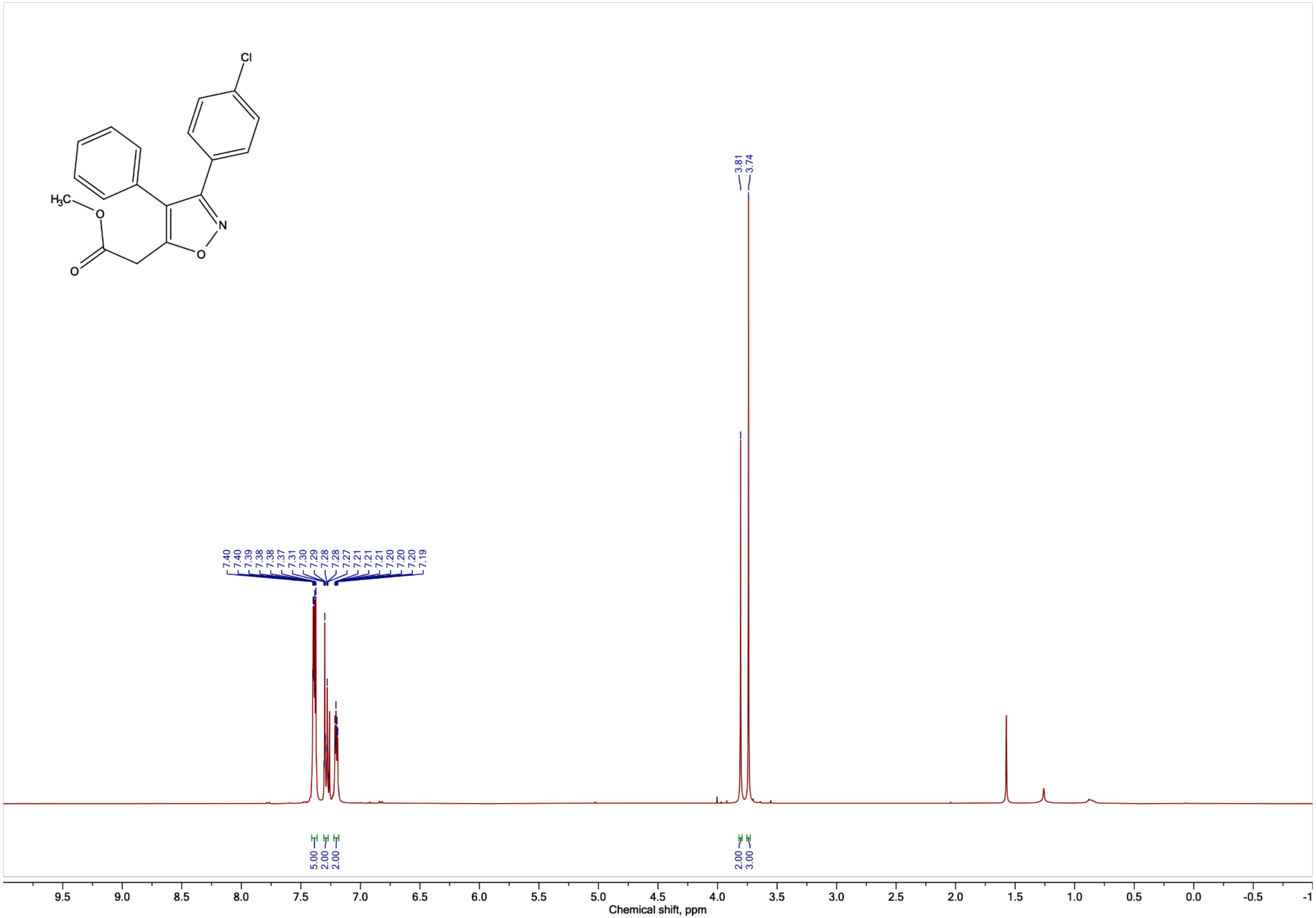

Methyl 2-(3-(4-chlorophenyl)-4-phenylisoxazol-5-yl)acetate (13c), $^{13}C\{^{1}H\}$ NMR, $CDCl_3$, 101 MHz

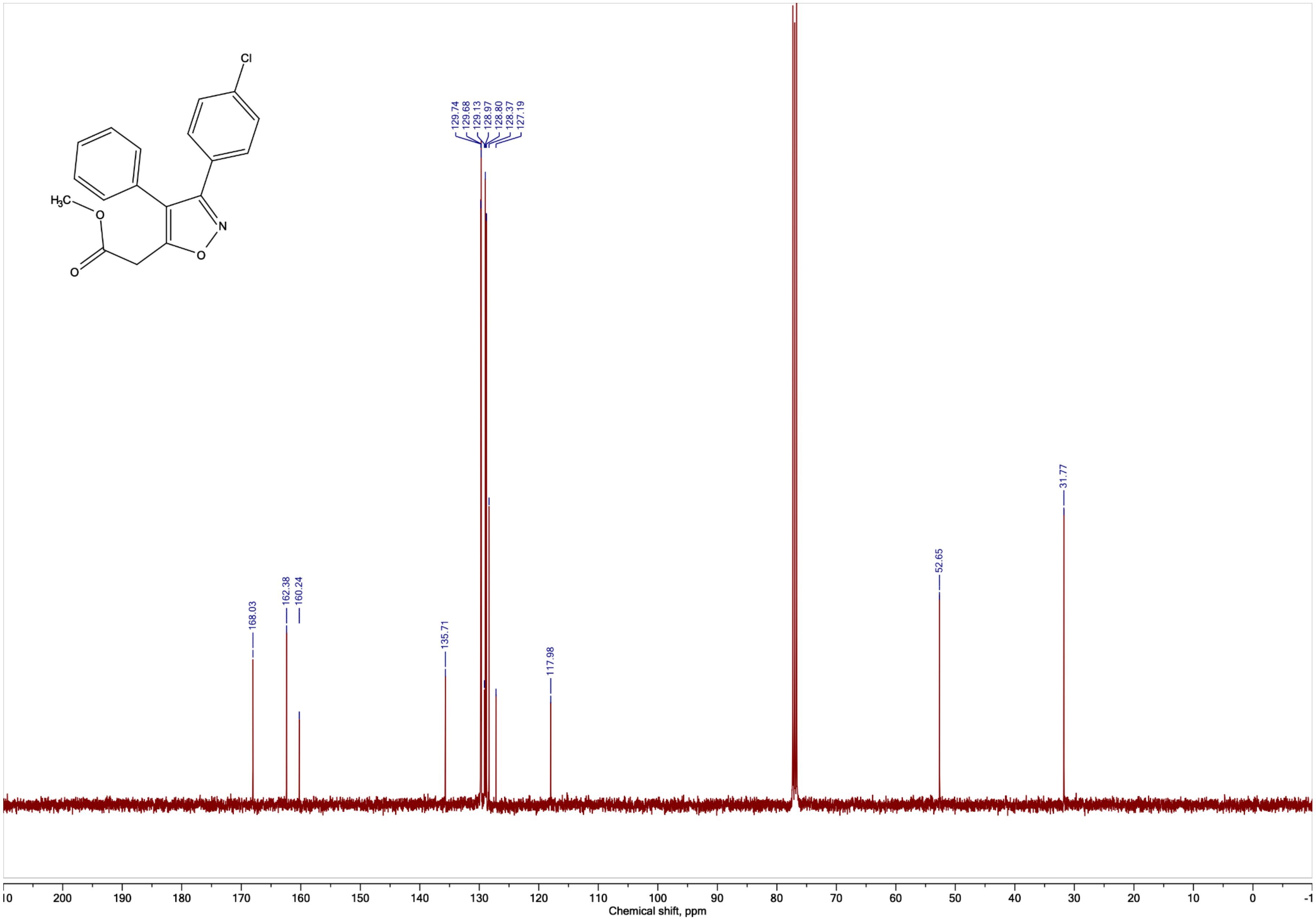

Methyl 2-(3-(4-chlorophenyl)-4-phenylisoxazol-5-yl)acetate (13c), DEPT, $CDCl_3$, 101 MHz

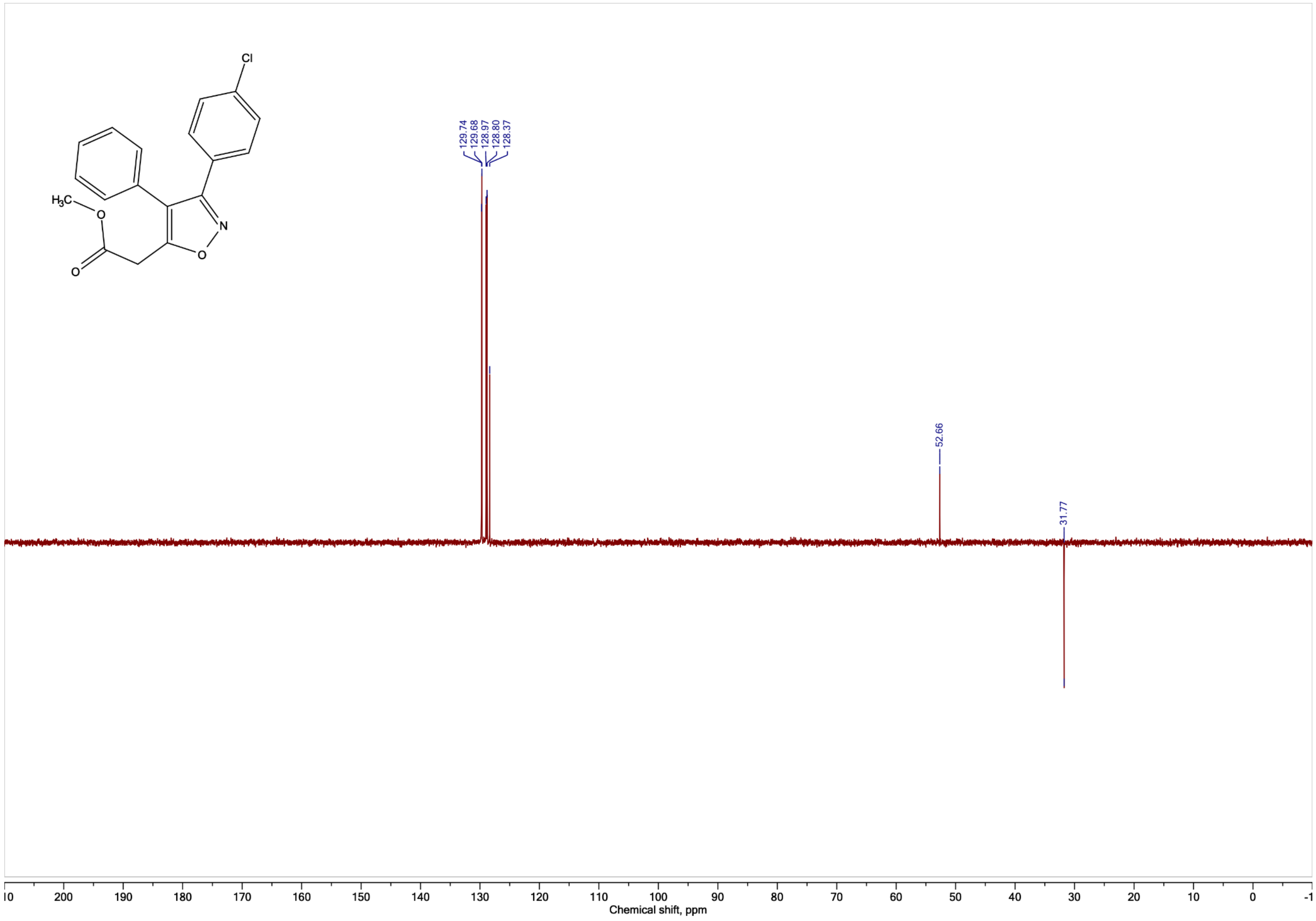

Methyl 2-(3-(4-chlorophenyl)-4-(4-methoxyphenyl)isoxazol-5-yl)acetate (13d), ^{1}H NMR, $CDCl_3$, 400 MHz

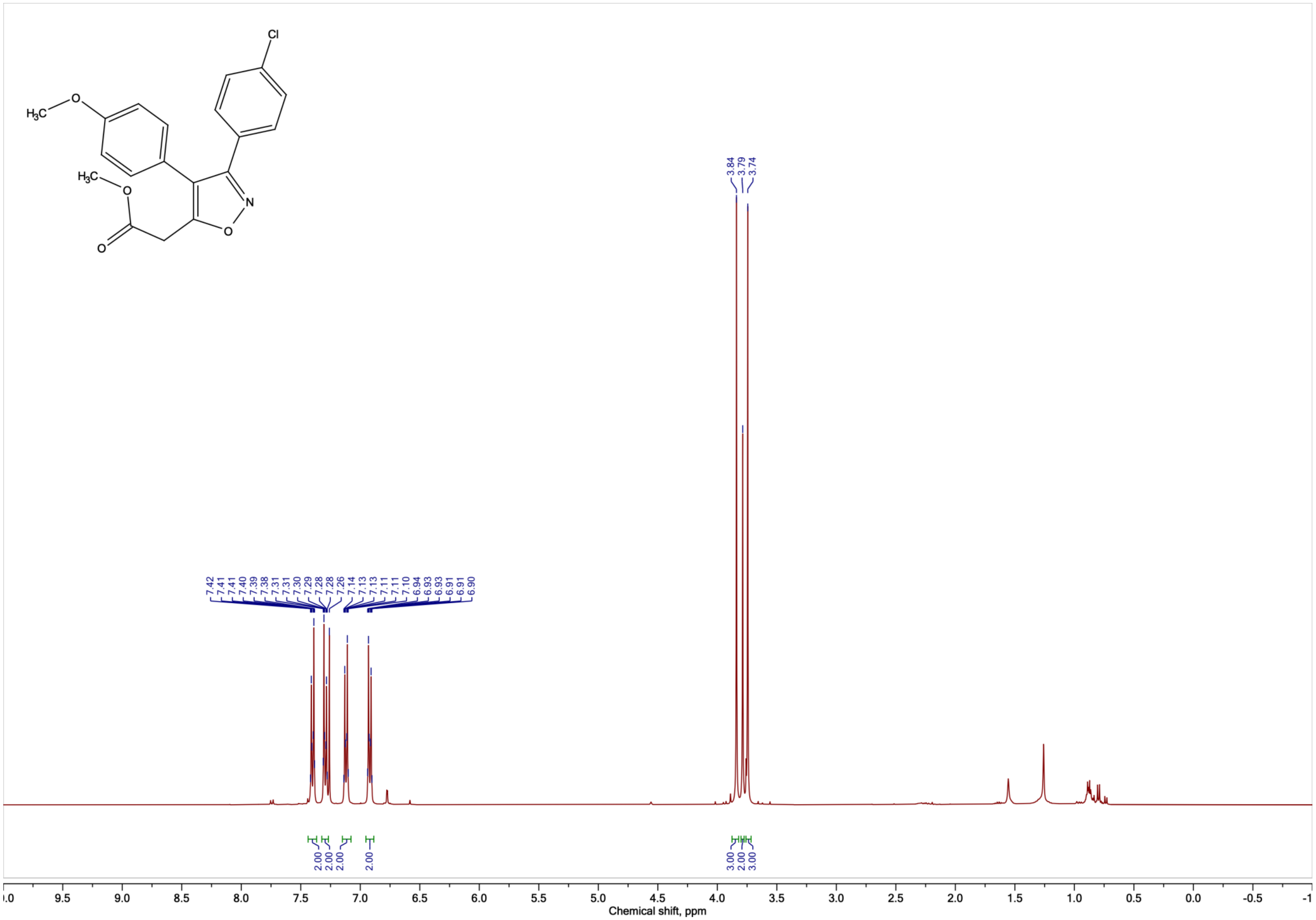

Methyl 2-(3-(4-chlorophenyl)-4-(4-methoxyphenyl)isoxazol-5-yl)acetate (13d), $^{13}C\{^{1}H\}$ NMR, $CDCl_3$, 101 MHz

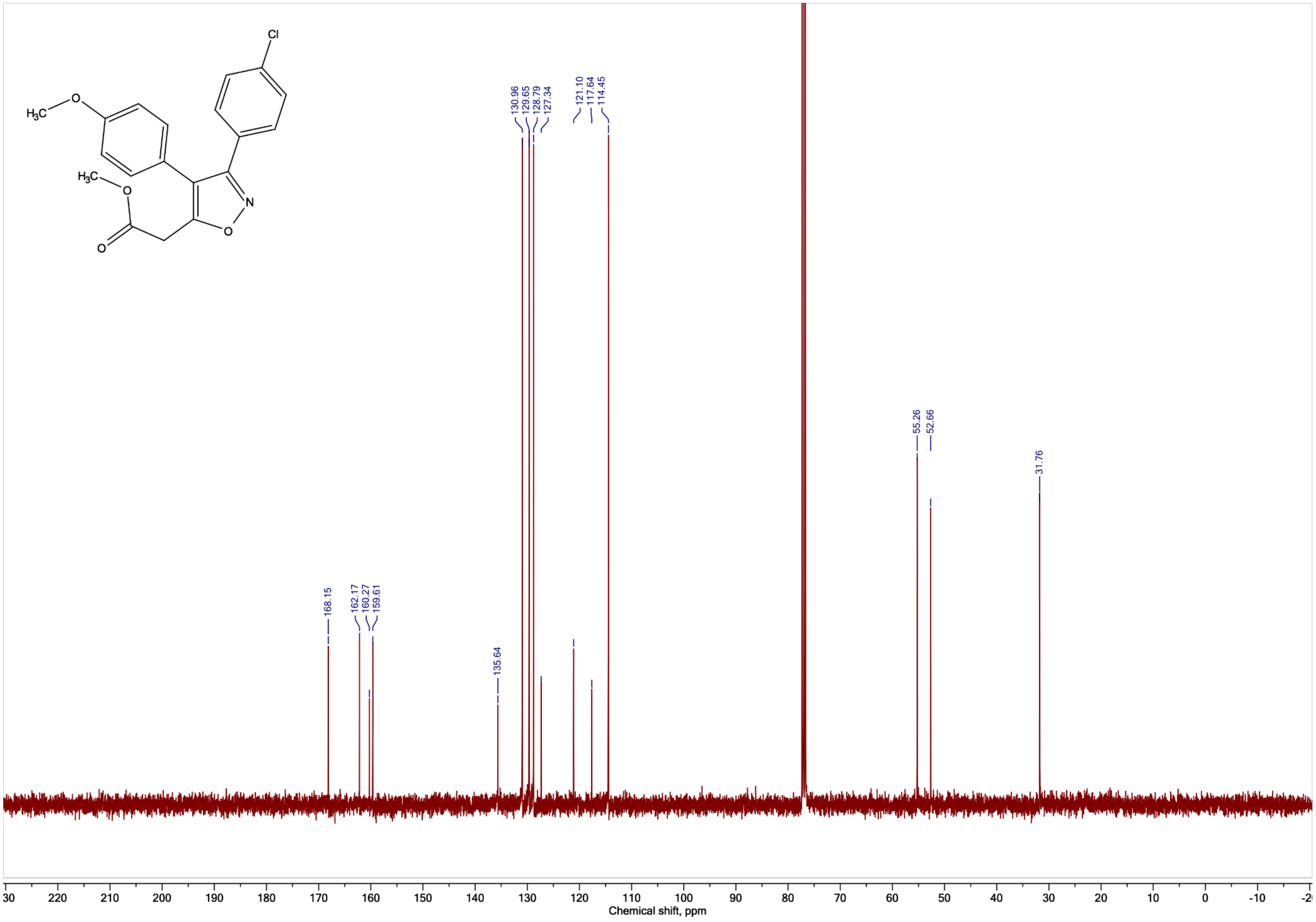

Methyl 2-(3-(4-chlorophenyl)-4-(4-methoxyphenyl)isoxazol-5-yl)acetate (13d), DEPT, $CDCl_3$, 101 MHz

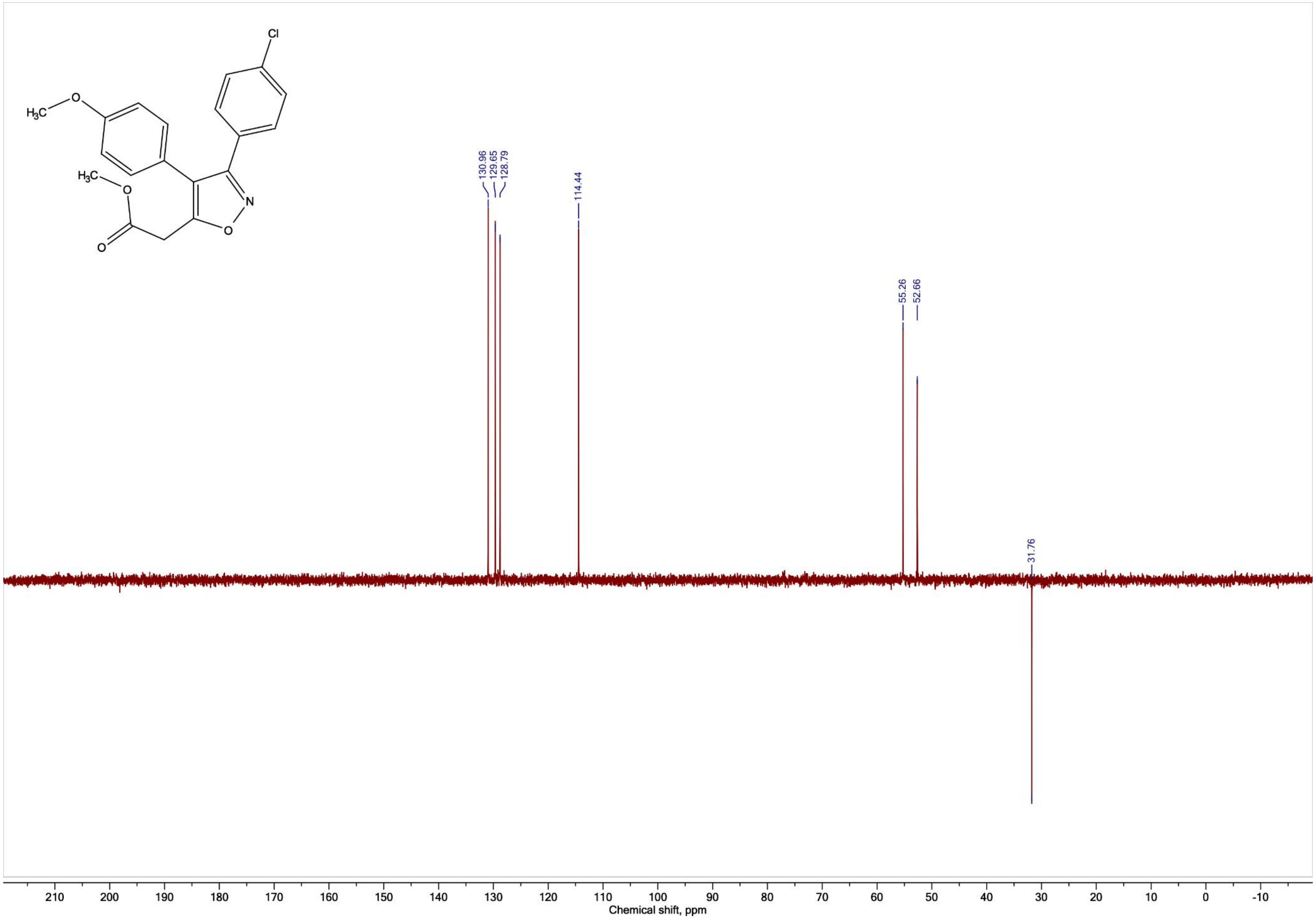

Methyl 2-(3-(4-bromophenyl)-4-(4-fluorophenyl)isoxazol-5-yl)acetate (13e), ^{1}H NMR, $CDCl_3$, 400 MHz

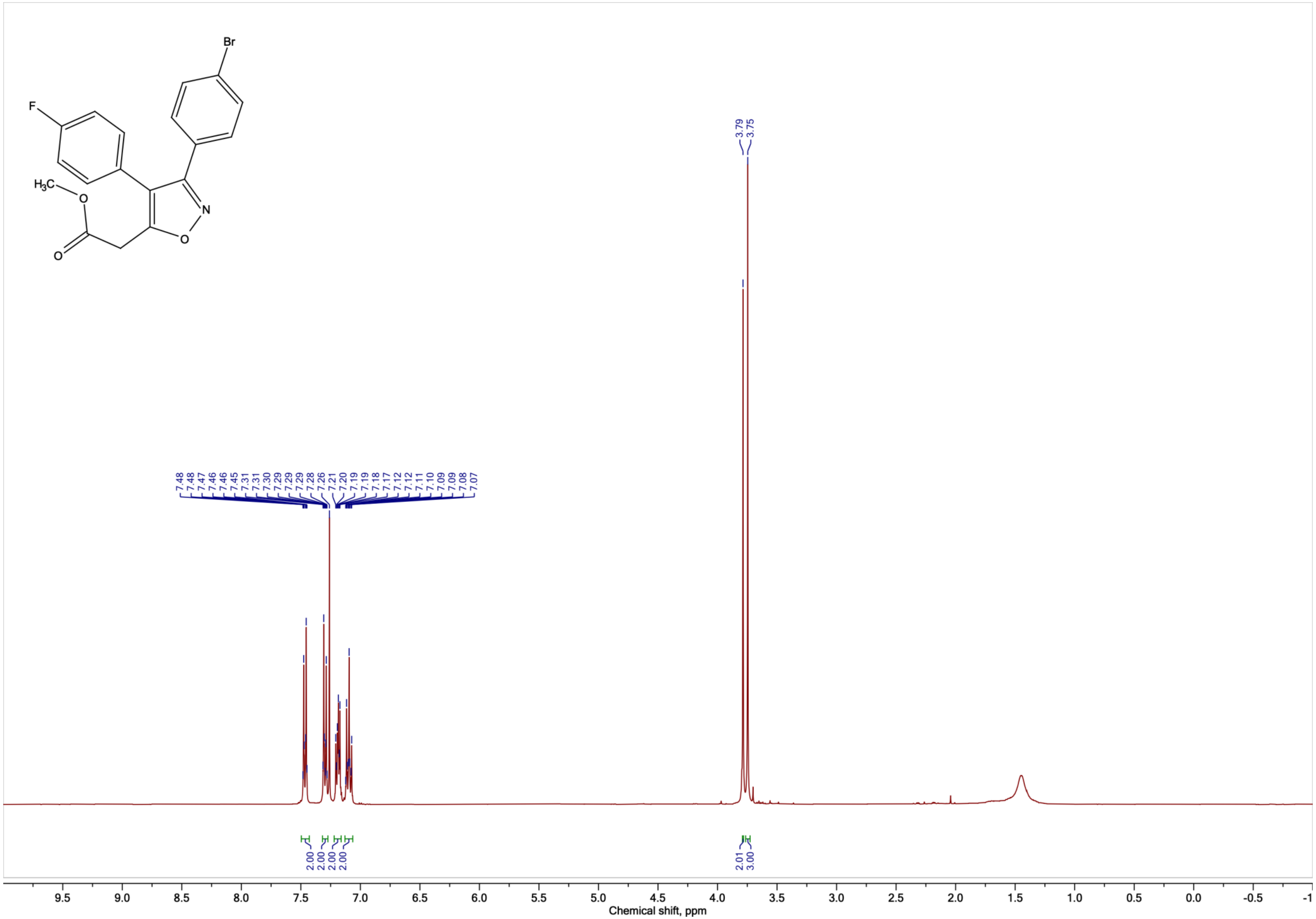

Methyl 2-(3-(4-bromophenyl)-4-(4-fluorophenyl)isoxazol-5-yl)acetate (13e), $^{13}C\{^{1}H\}$ NMR, $CDCl_3$, 101 MHz

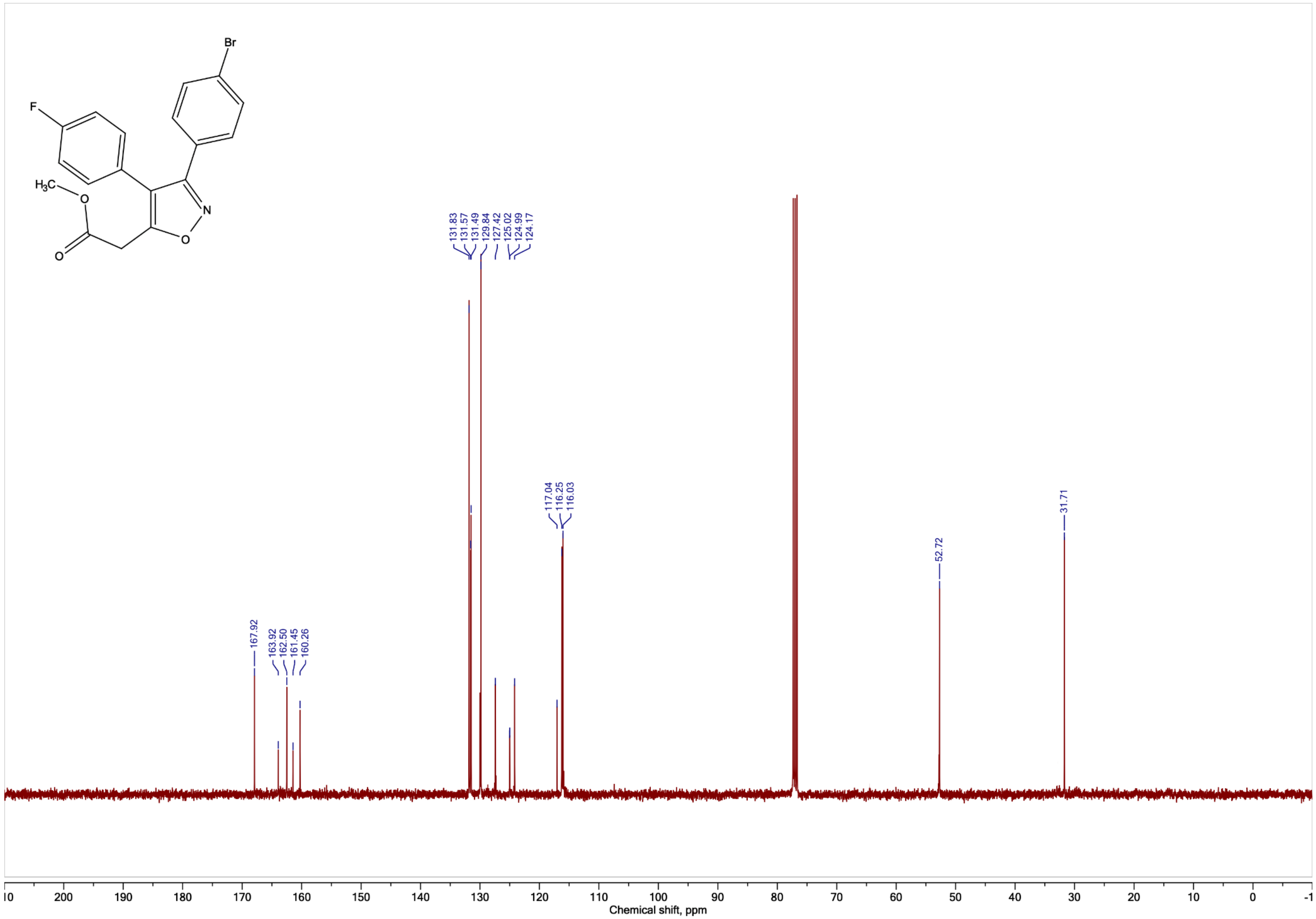

Methyl 2-(3-(4-bromophenyl)-4-(4-fluorophenyl)isoxazol-5-yl)acetate (13e), DEPT, $CDCl_3$, 101 MHz

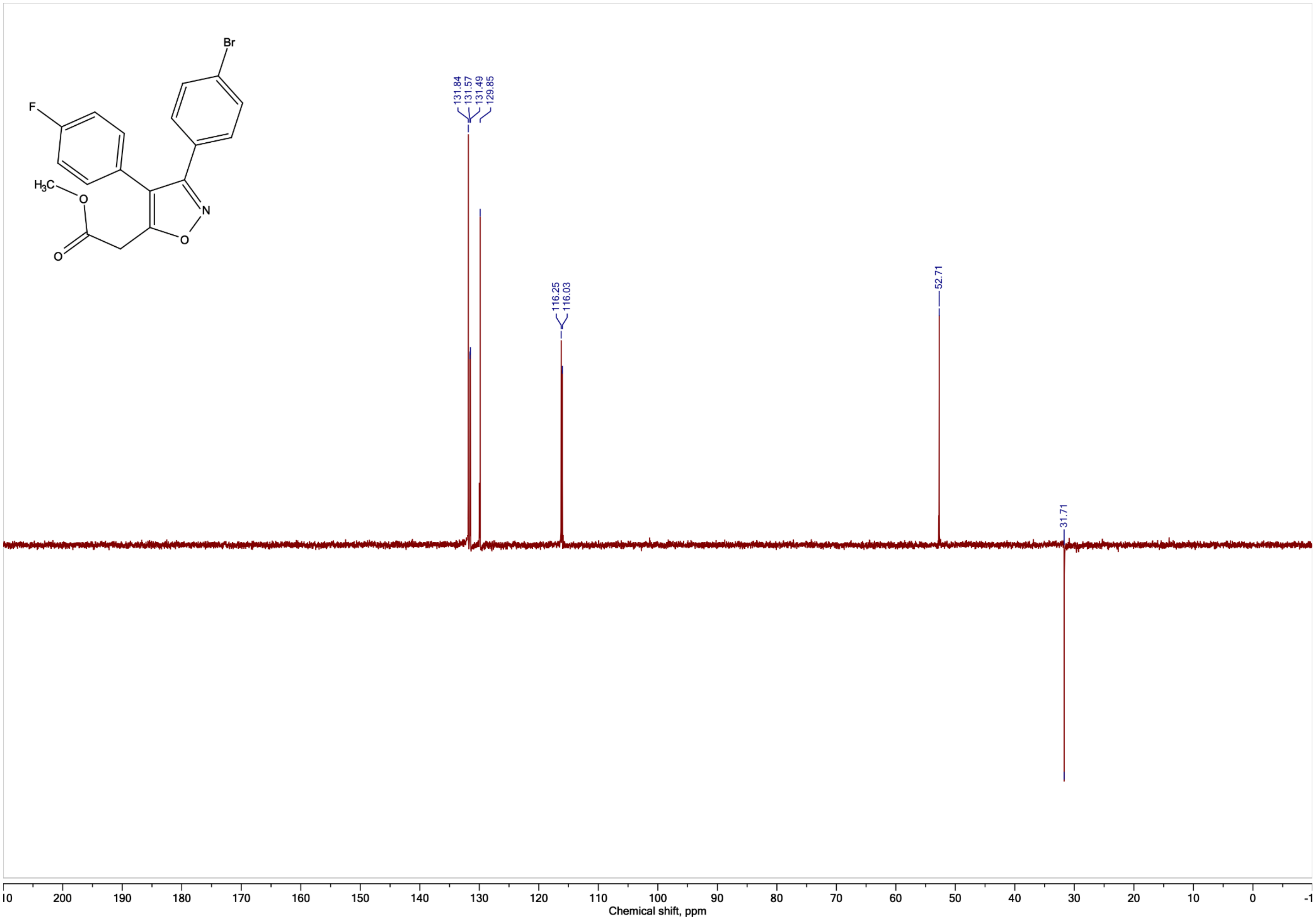

Methyl 2-(3-(4'-fluoro-[1,1'-biphenyl]-4-yl)-4-(4-fluorophenyl)isoxazol-5-yl)acetate (13f), ^{1}H NMR, $CDCl_3$, 400 MHz

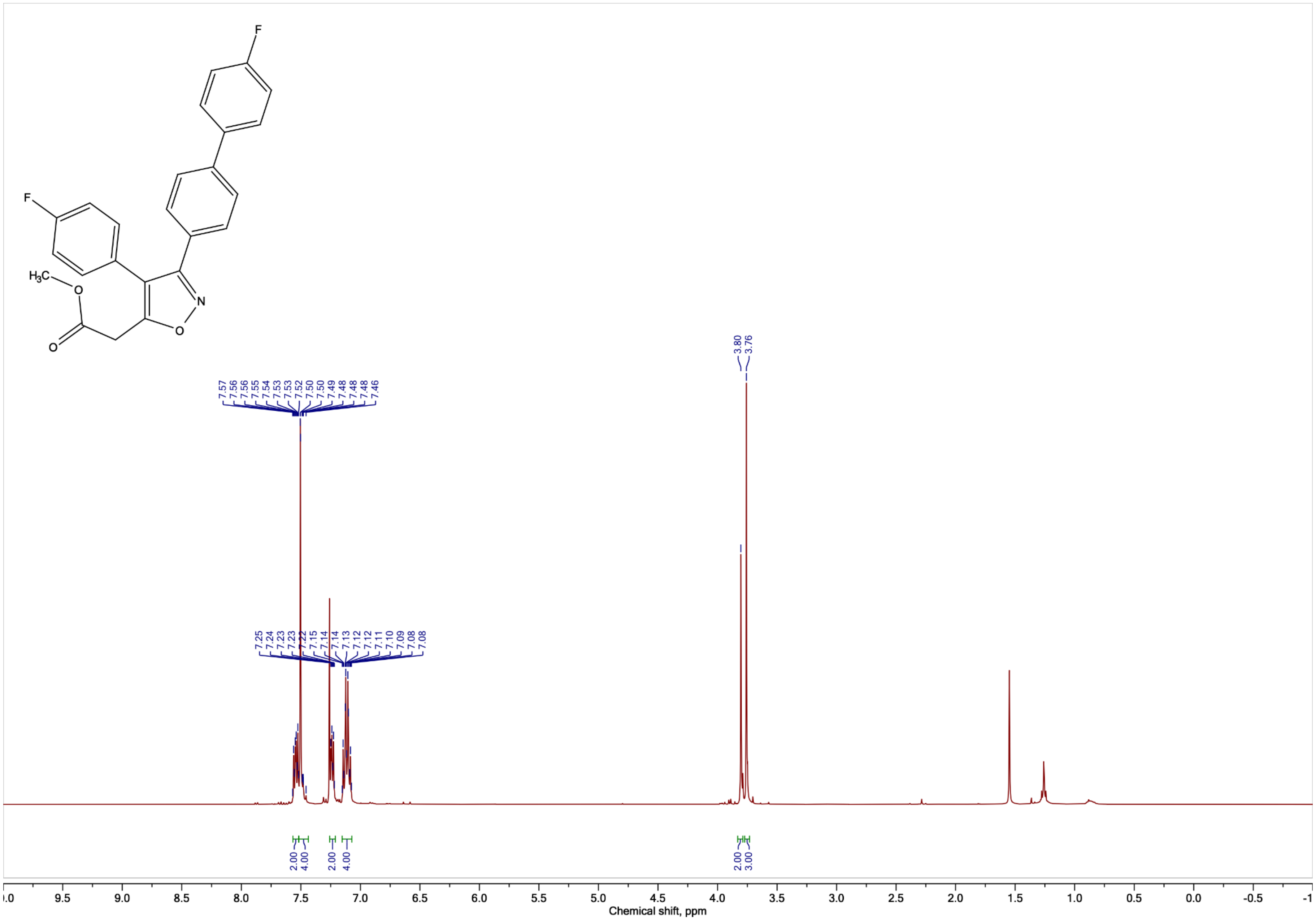

Methyl 2-(3-(4'-fluoro-[1,1'-biphenyl]-4-yl)-4-(4-fluorophenyl)isoxazol-5-yl)acetate (13f), $^{13}C\{^1H\}$ NMR, $CDCl_3$, 101 MHz

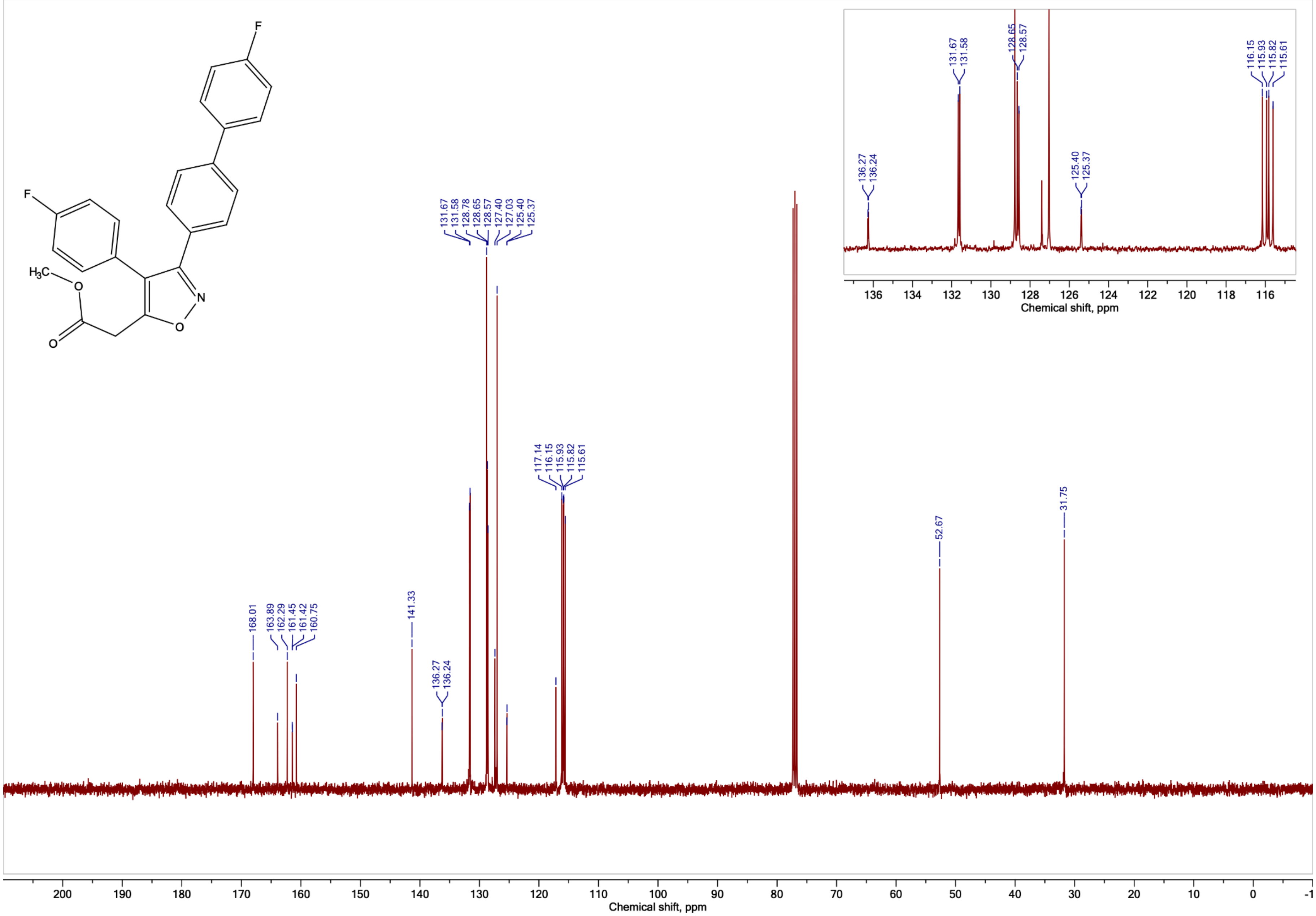

Methyl 2-(3-(4'-fluoro-[1,1'-biphenyl]-4-yl)-4-(4-fluorophenyl)isoxazol-5-yl)acetate (13f), DEPT, $CDCl_3$, 101 MHz

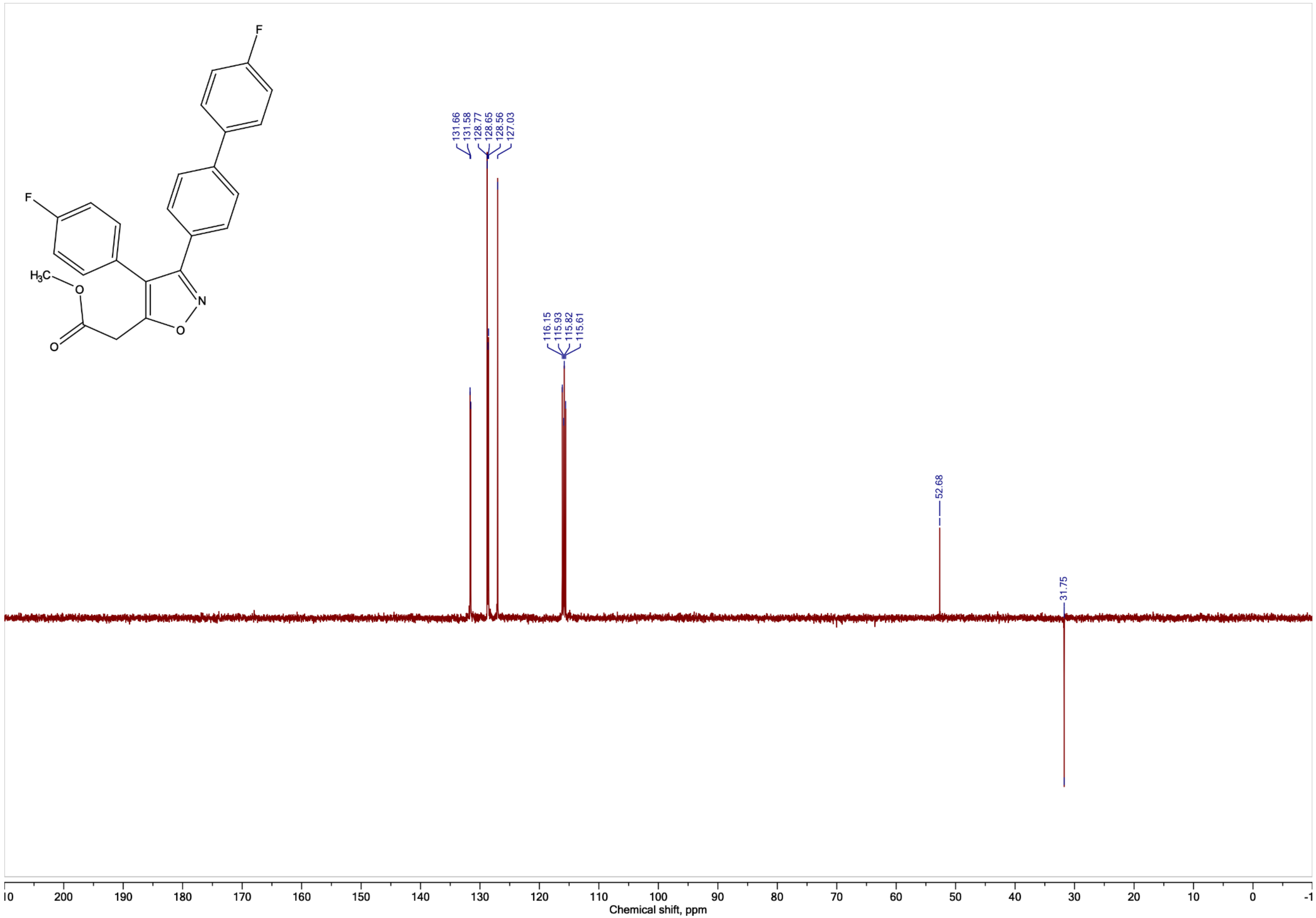

Methyl 3-oxo-2-(3-phenylisoxazol-5-yl)pentanoate (1a), ^{1}H NMR, $CDCl_3$, 400 MHz

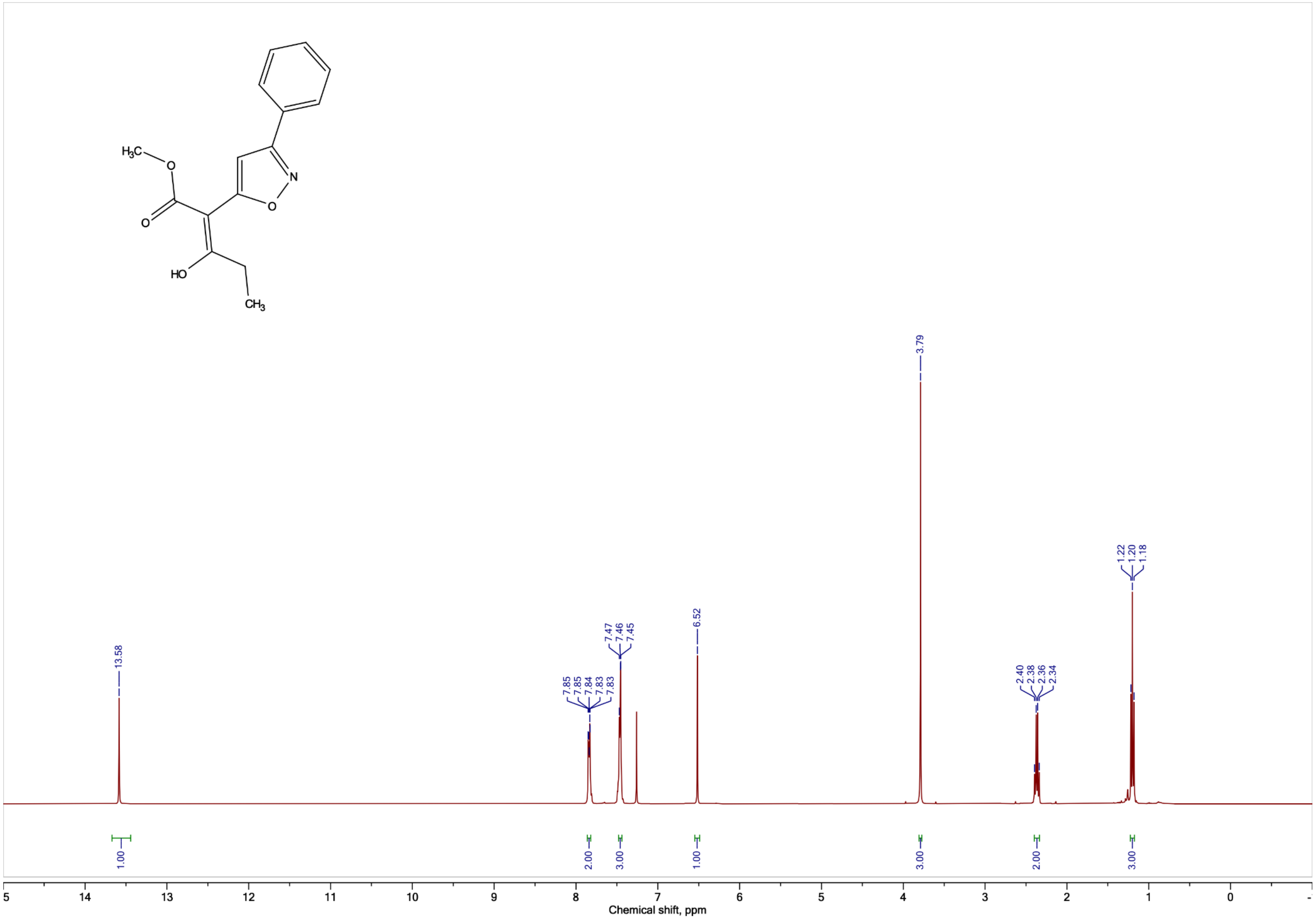

Methyl 3-oxo-2-(3-phenylisoxazol-5-yl)pentanoate (1a), $^{13}C\{^{1}H\}$ NMR, $CDCl_3$, 101 MHz

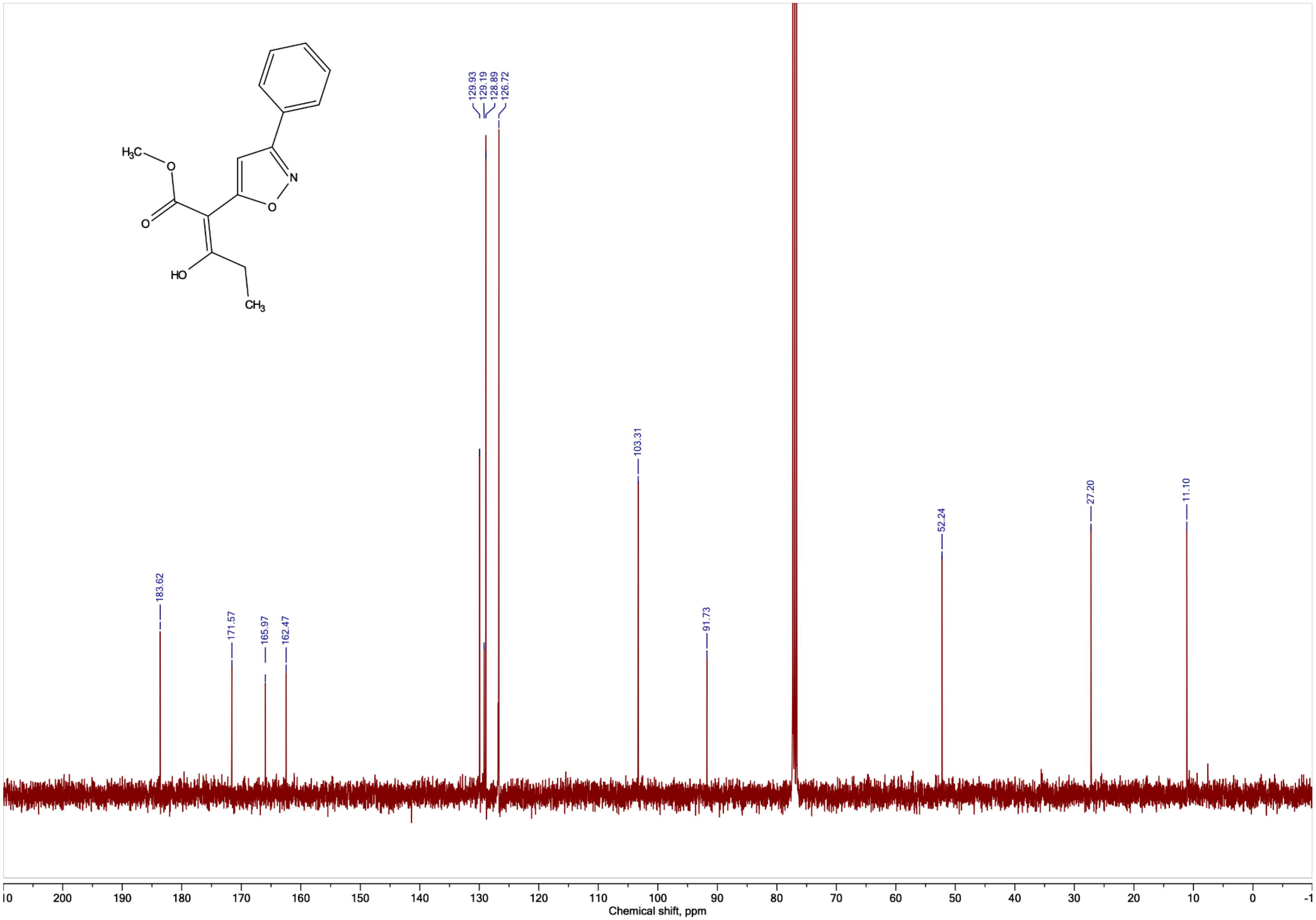

Methyl 3-oxo-2-(3-phenylisoxazol-5-yl)pentanoate (1a), DEPT, $CDCl_3$, 101 MHz

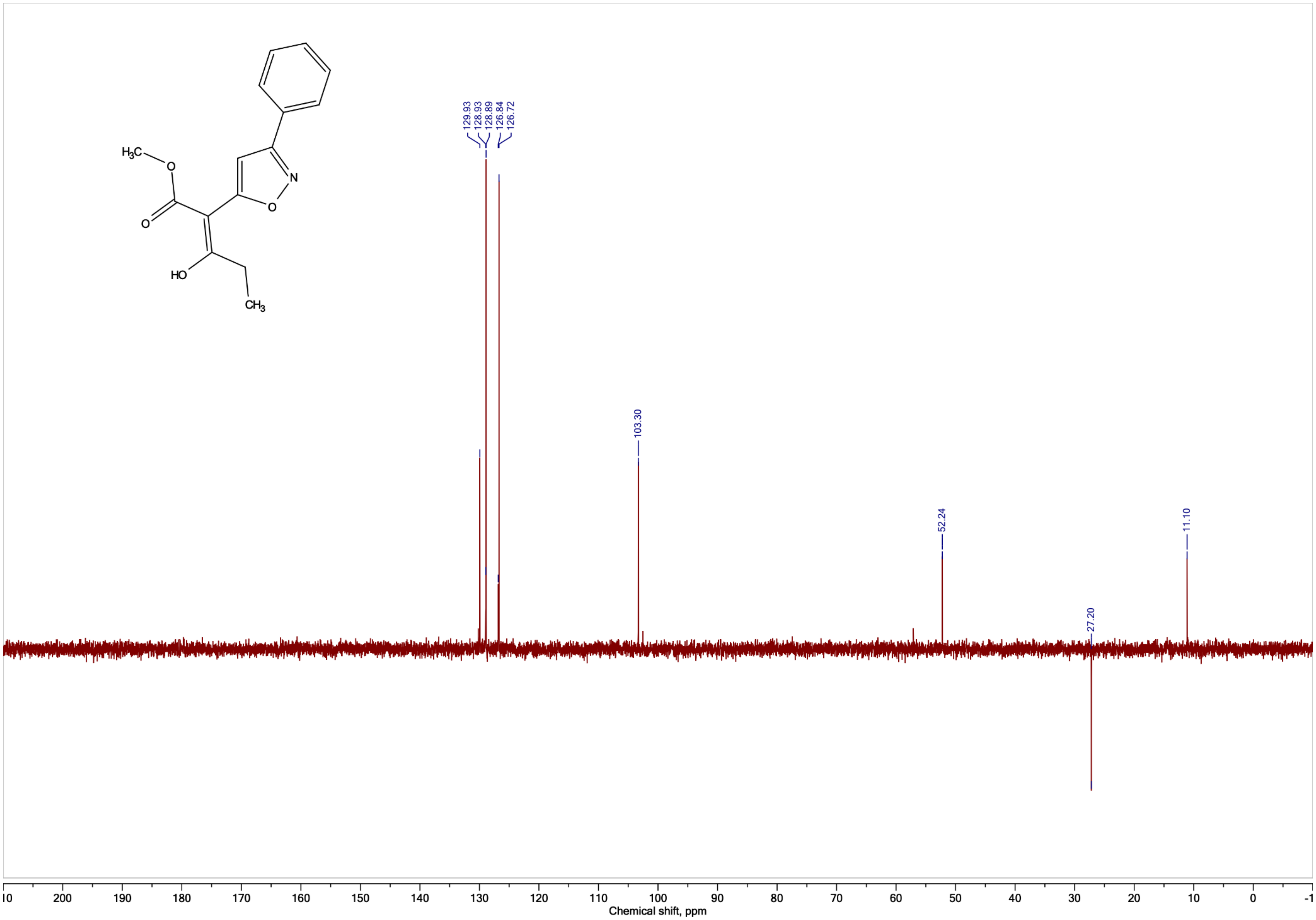

Methyl 2-(3-benzylisoxazol-5-yl)-3-oxo-3-(*p*-tolyl)propanoate (1b), ^{1}H NMR, $CDCl_3$, 400 MHz

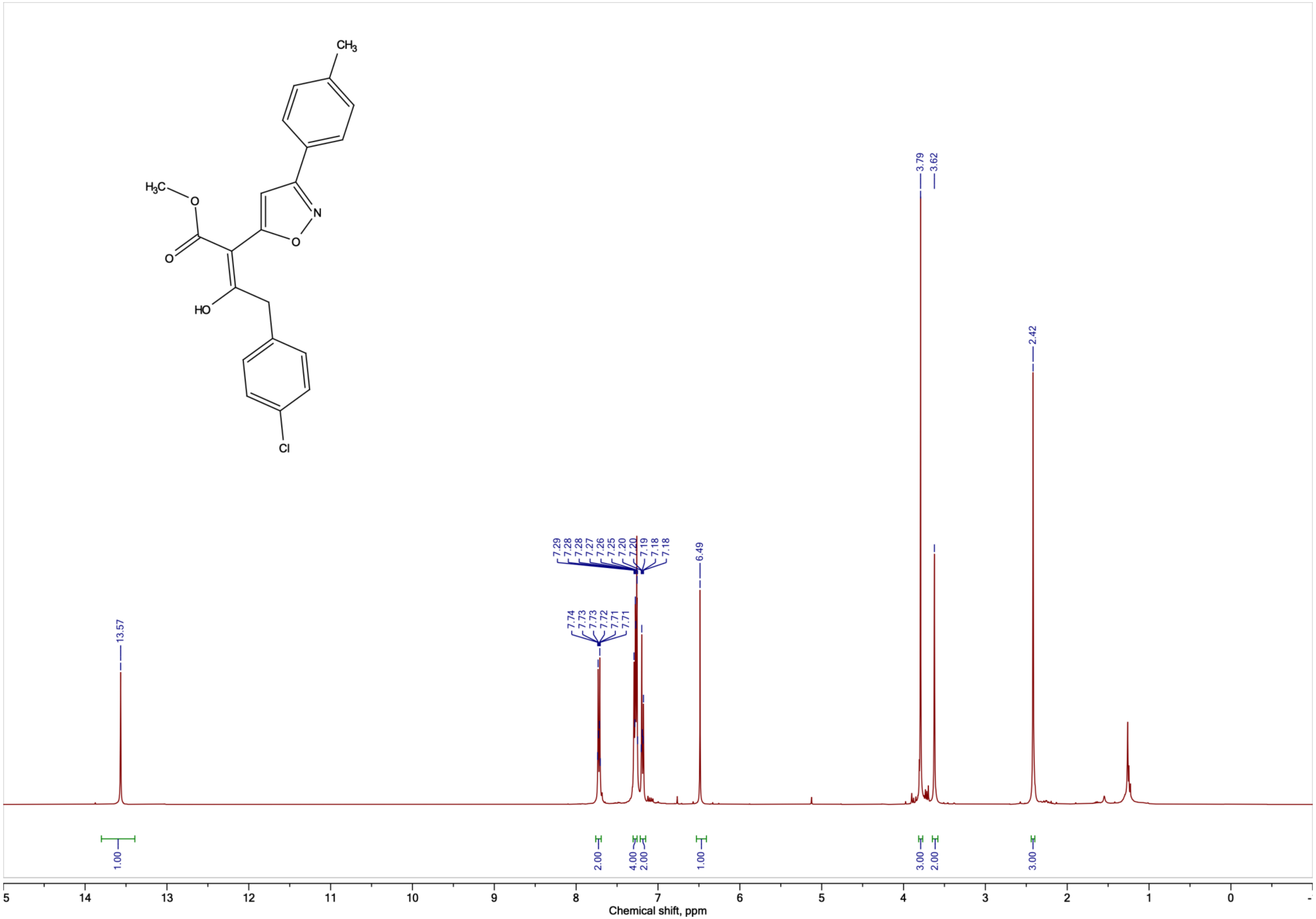

Methyl 2-(3-benzylisoxazol-5-yl)-3-oxo-3-(*p*-tolyl)propanoate (1b), $^{13}C\{^1H\}$ NMR, $CDCl_3$, 101 MHz

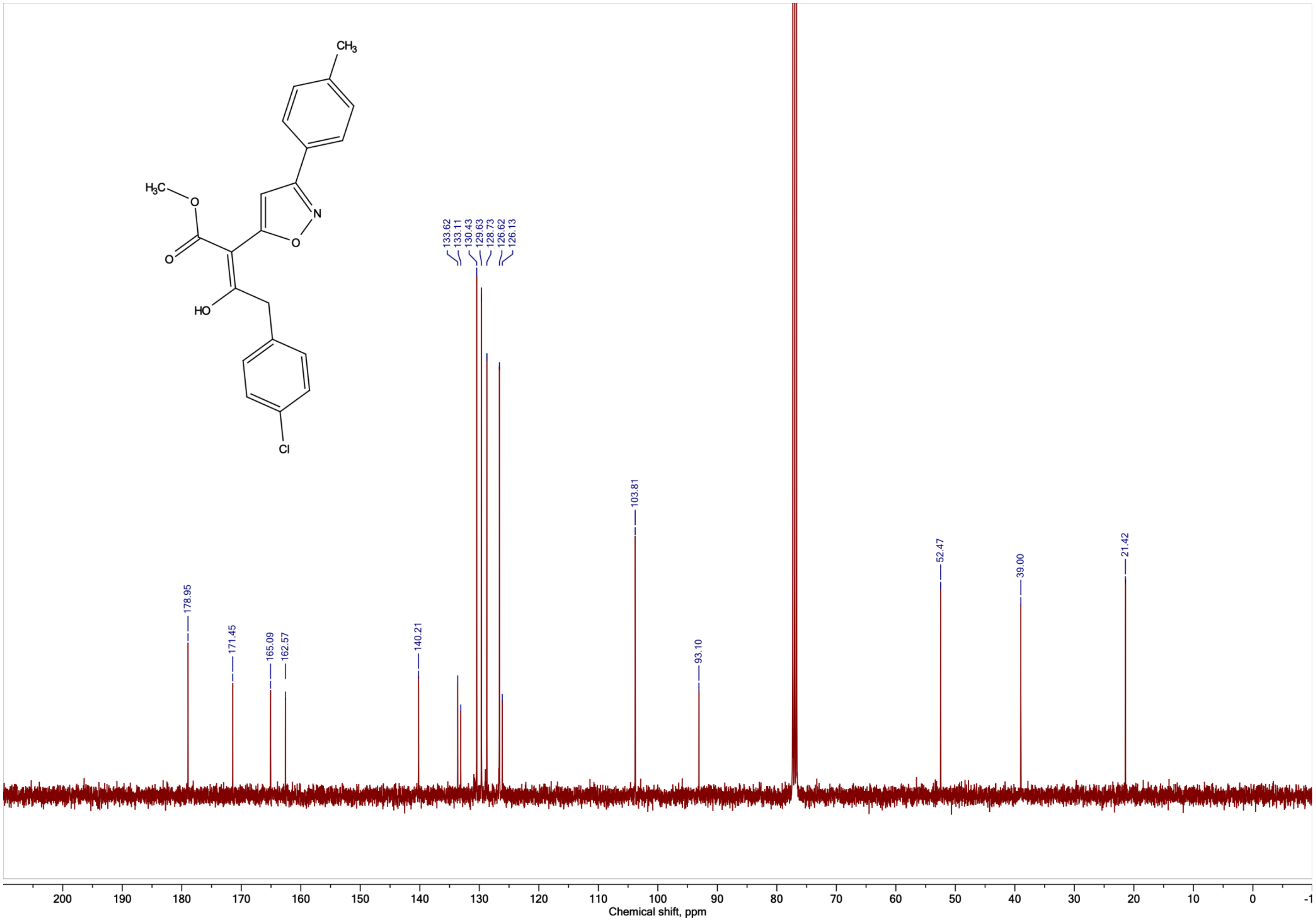

Methyl 2-(3-benzylisoxazol-5-yl)-3-oxo-3-(*p*-tolyl)propanoate (1b), DEPT, $CDCl_3$, 101 MHz

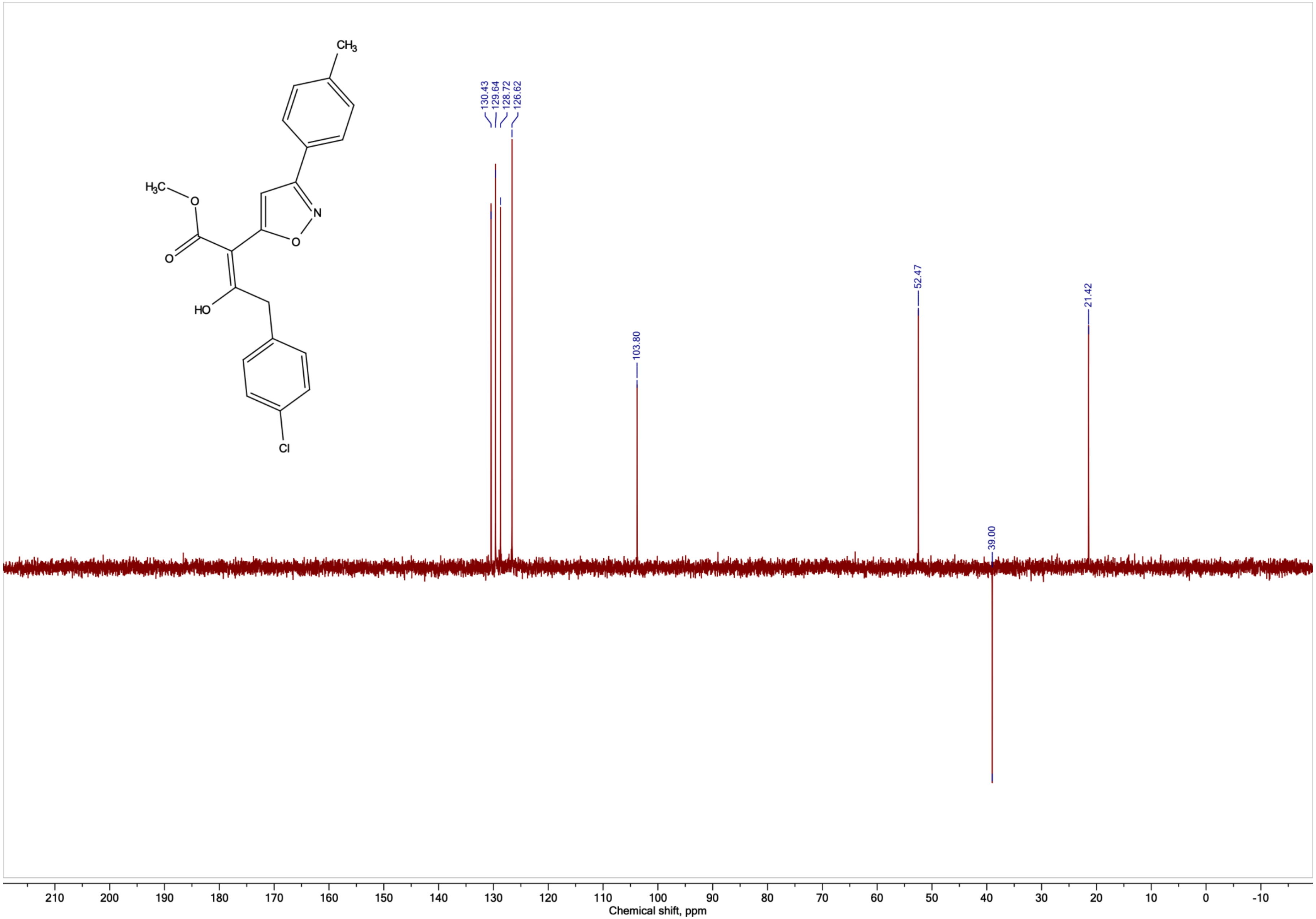

Methyl 3-(4-bromophenyl)-2-(3-(4-(*tert*-butyl)phenyl)isoxazol-5-yl)-3-oxopropanoate (1c), ^{1}H NMR, $CDCl_3$, 400 MHz

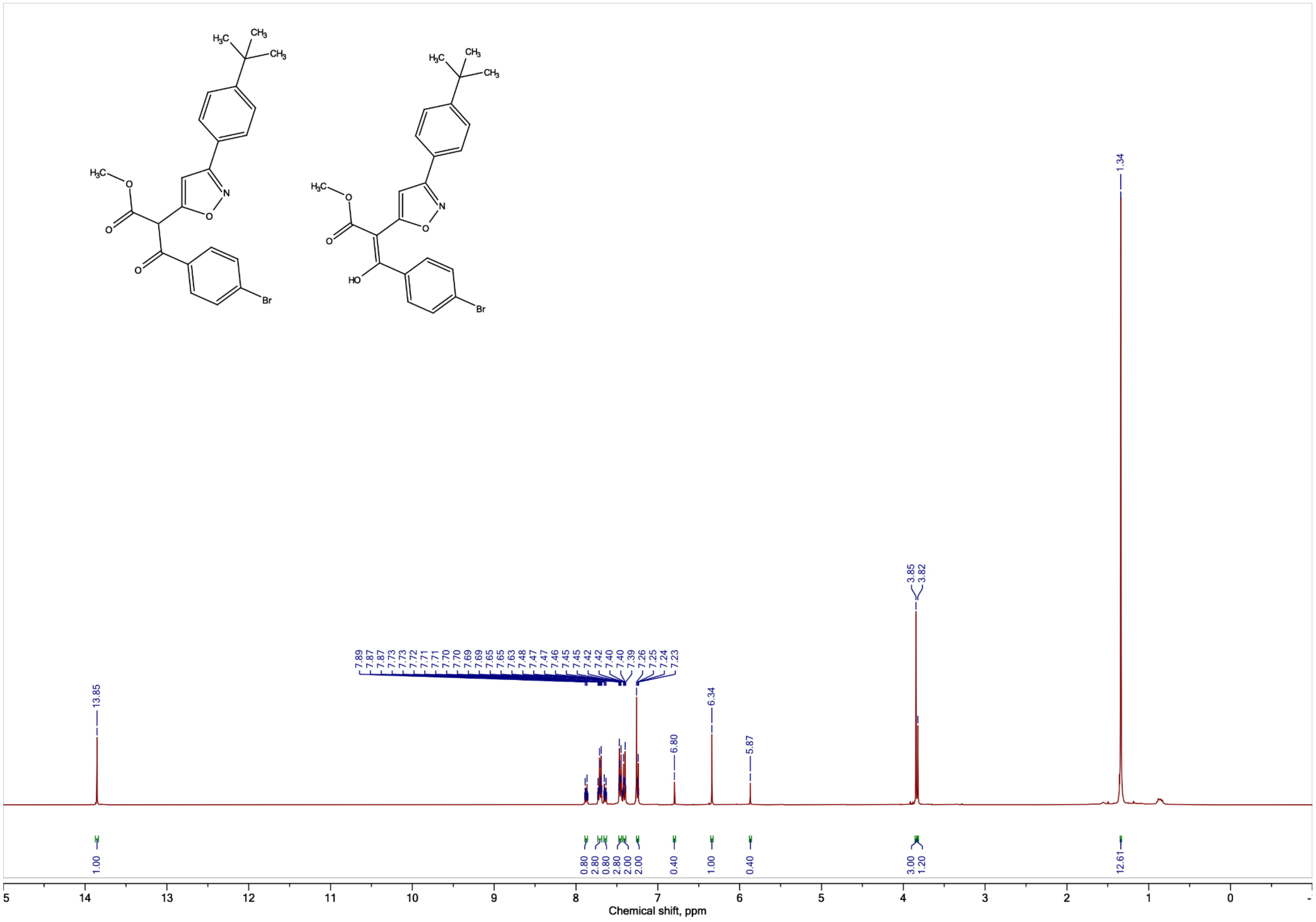

Methyl 3-(4-bromophenyl)-2-(3-(4-(*tert*-butyl)phenyl)isoxazol-5-yl)-3-oxopropanoate (1c), $^{13}C\{^1H\}$ NMR, $CDCl_3$, 101 MHz

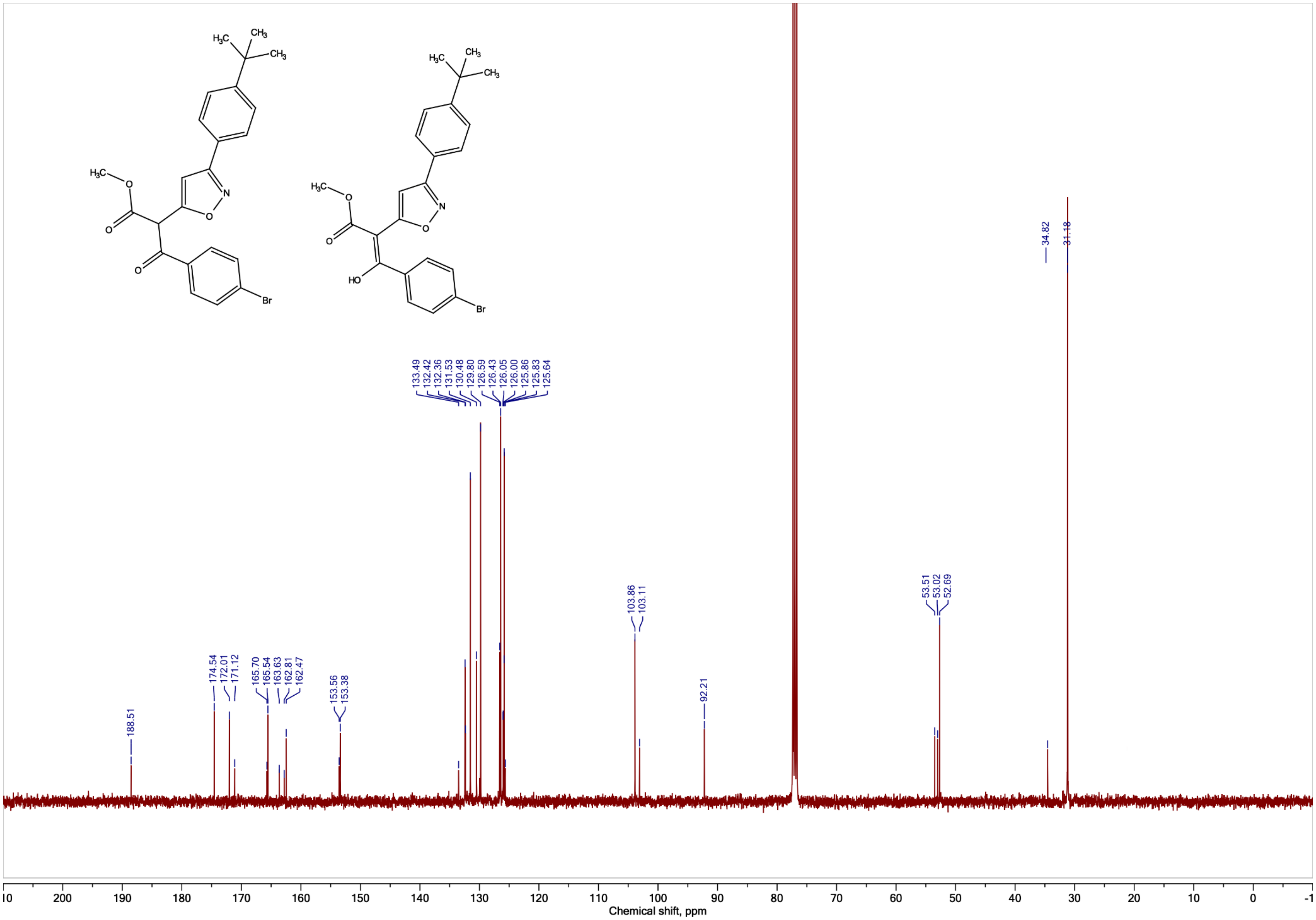

Methyl 3-(4-bromophenyl)-2-(3-(4-(*tert*-butyl)phenyl)isoxazol-5-yl)-3-oxopropanoate (1c), DEPT, $CDCl_3$, 101 MHz

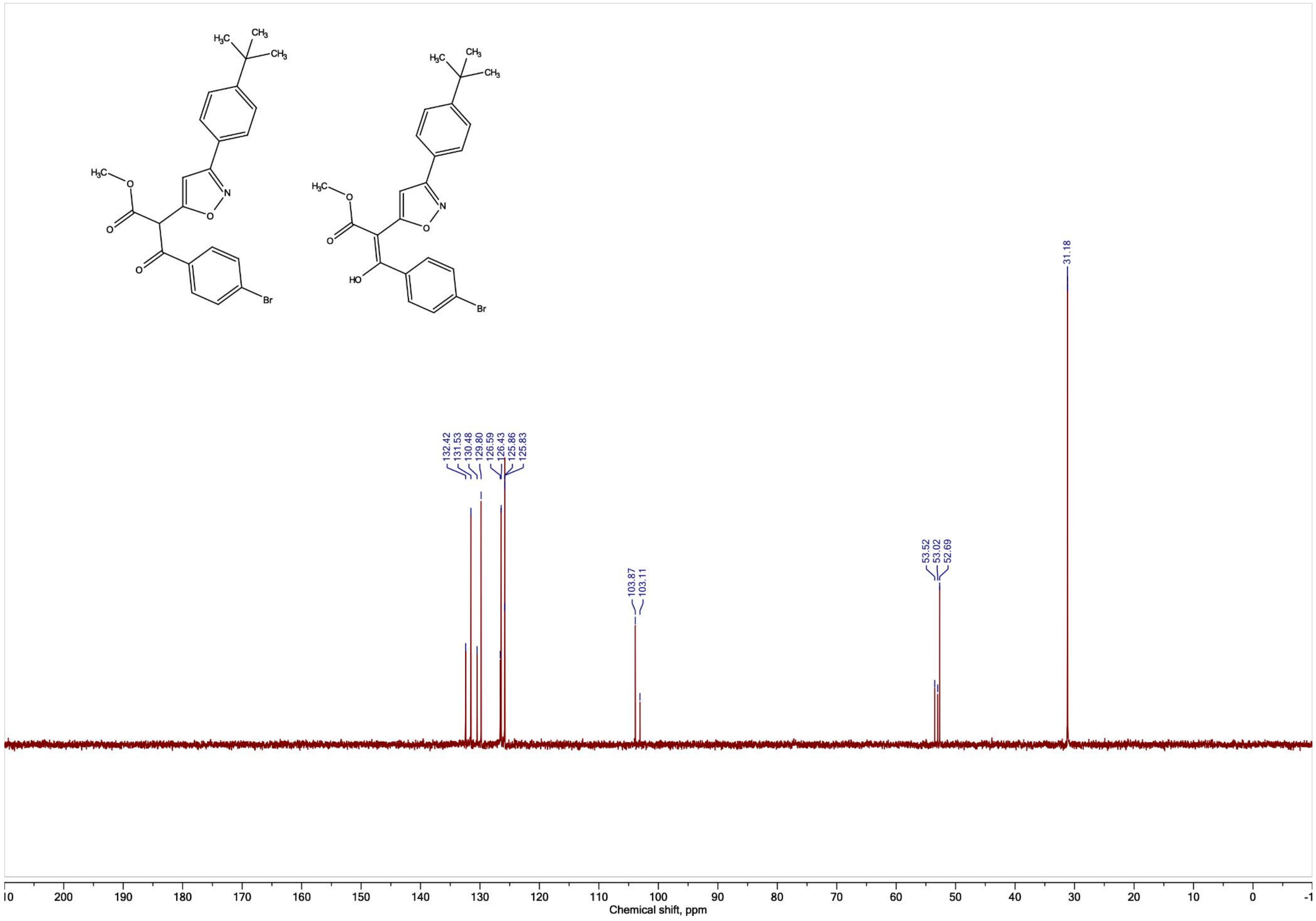

Methyl 3-(4-bromophenyl)-2-(3-(4-methoxyphenyl)isoxazol-5-yl)-3-oxopropanoate (1d), ^{1}H NMR, $CDCl_3$, 400 MHz

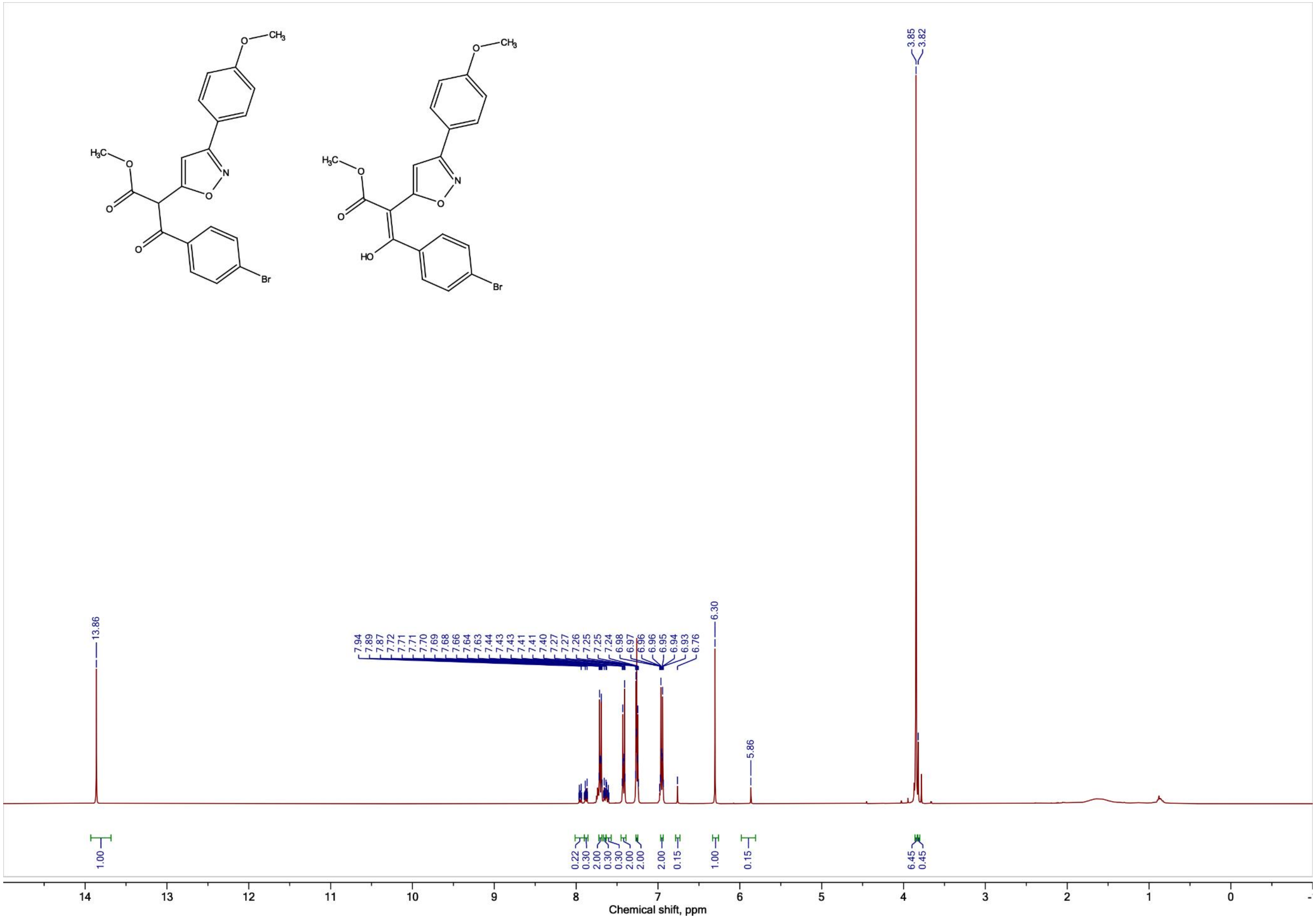

Methyl 3-(4-bromophenyl)-2-(3-(4-methoxyphenyl)isoxazol-5-yl)-3-oxopropanoate (1d), $^{13}C\{^{1}H\}$ NMR, $CDCl_3$, 101 MHz

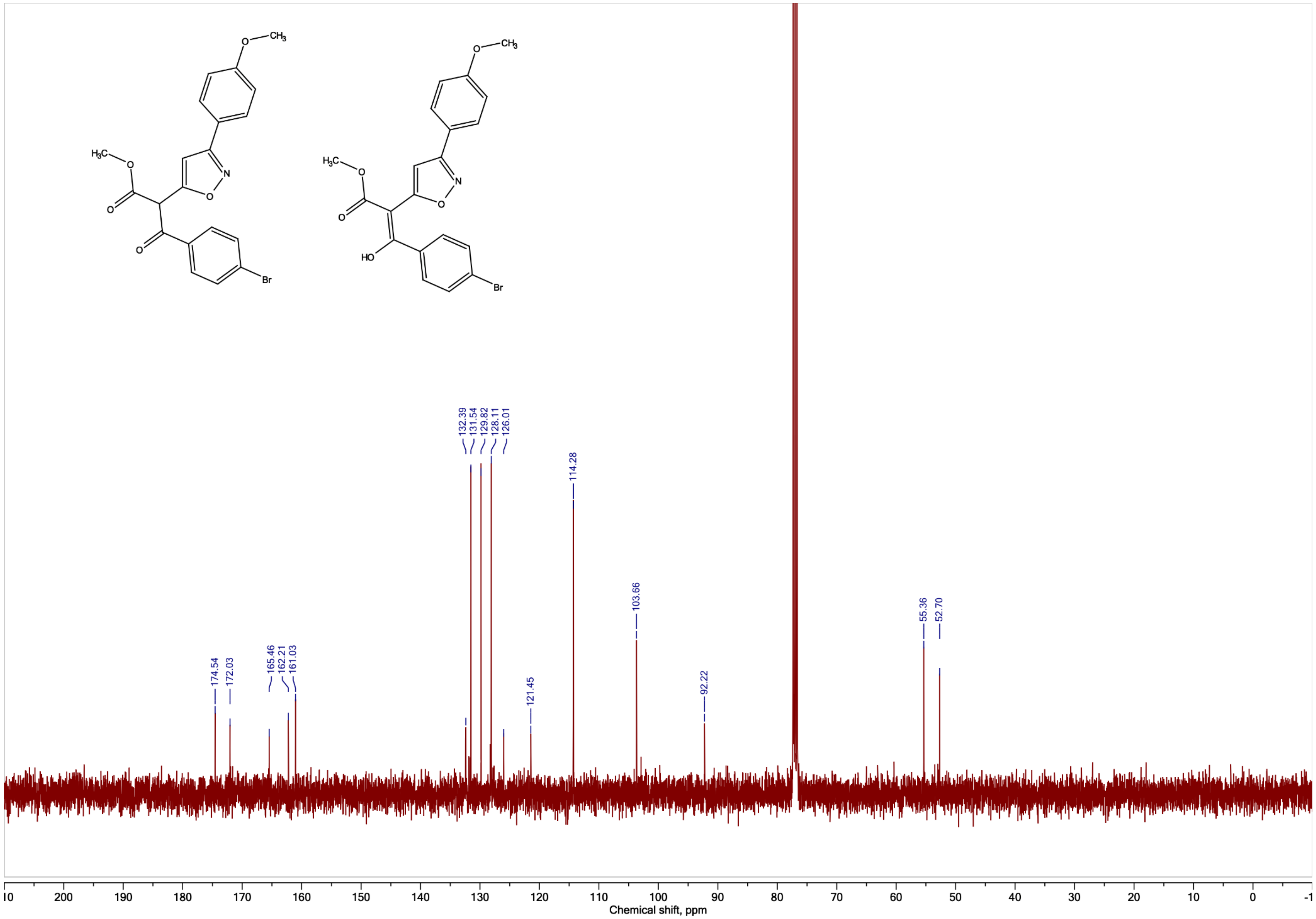

Methyl 3-(4-bromophenyl)-2-(3-(4-methoxyphenyl)isoxazol-5-yl)-3-oxopropanoate (1d), DEPT, $CDCl_3$, 101 MHz

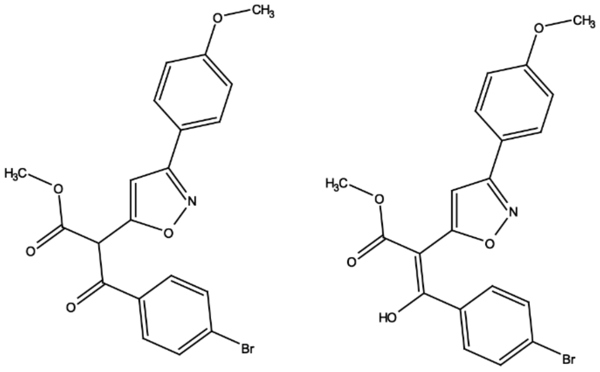

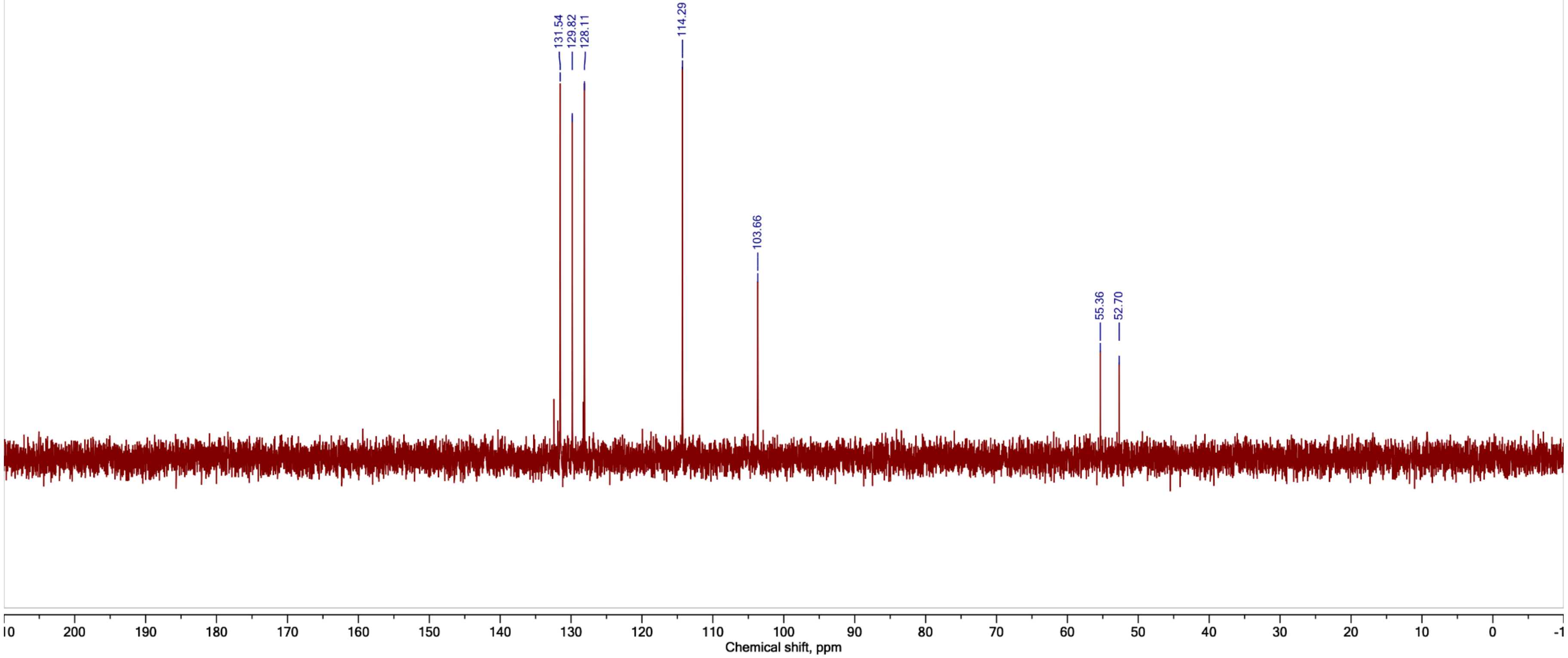

Methyl 2-(3-(4-fluorophenyl)isoxazol-5-yl)-3-oxo-3-phenylpropanoate (1e), ^{1}H NMR, $CDCl_3$, 400 MHz

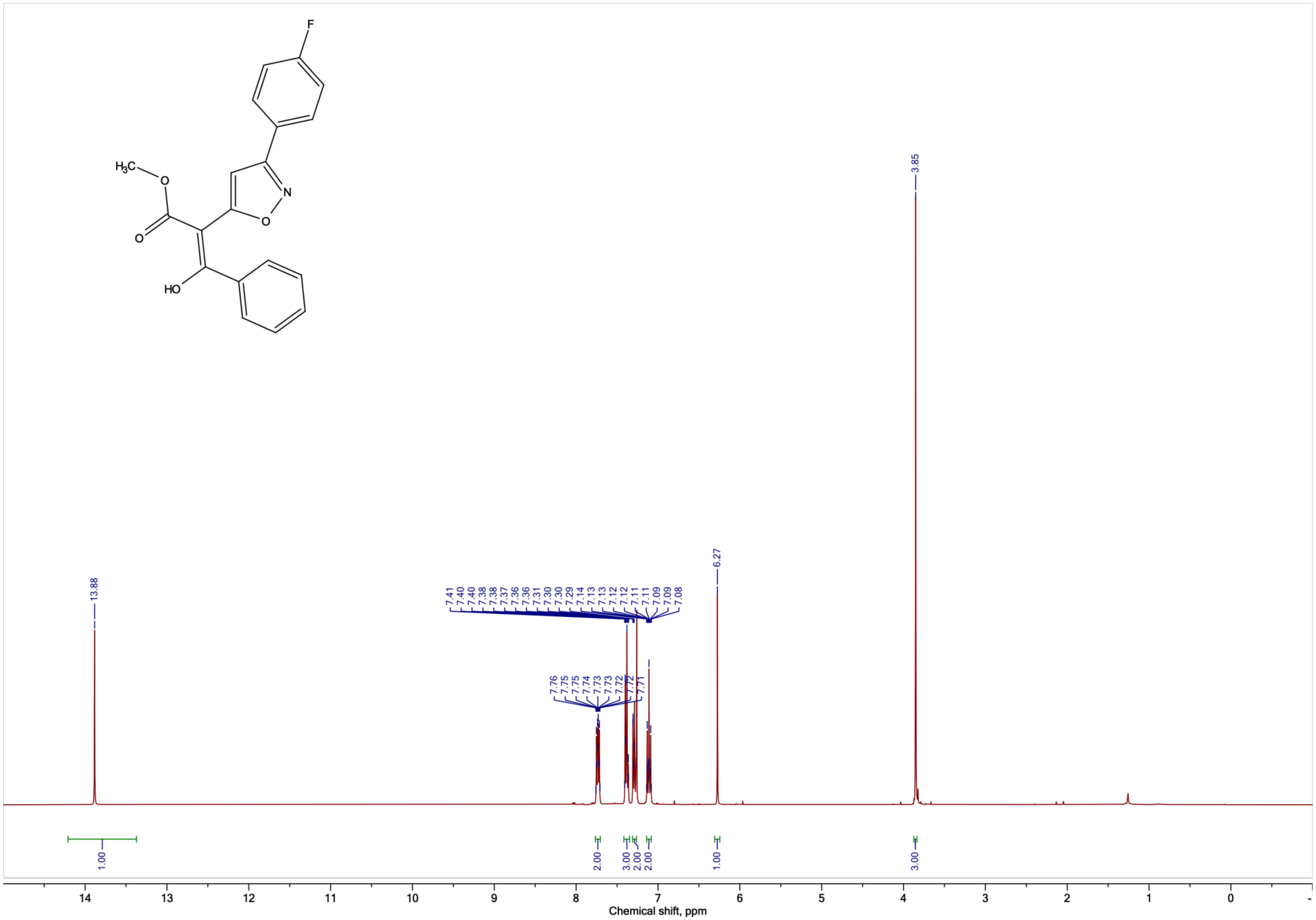

Methyl 2-(3-(4-fluorophenyl)isoxazol-5-yl)-3-oxo-3-phenylpropanoate (1e), $^{13}C\{^{1}H\}$ NMR, $CDCl_3$, 101 MHz

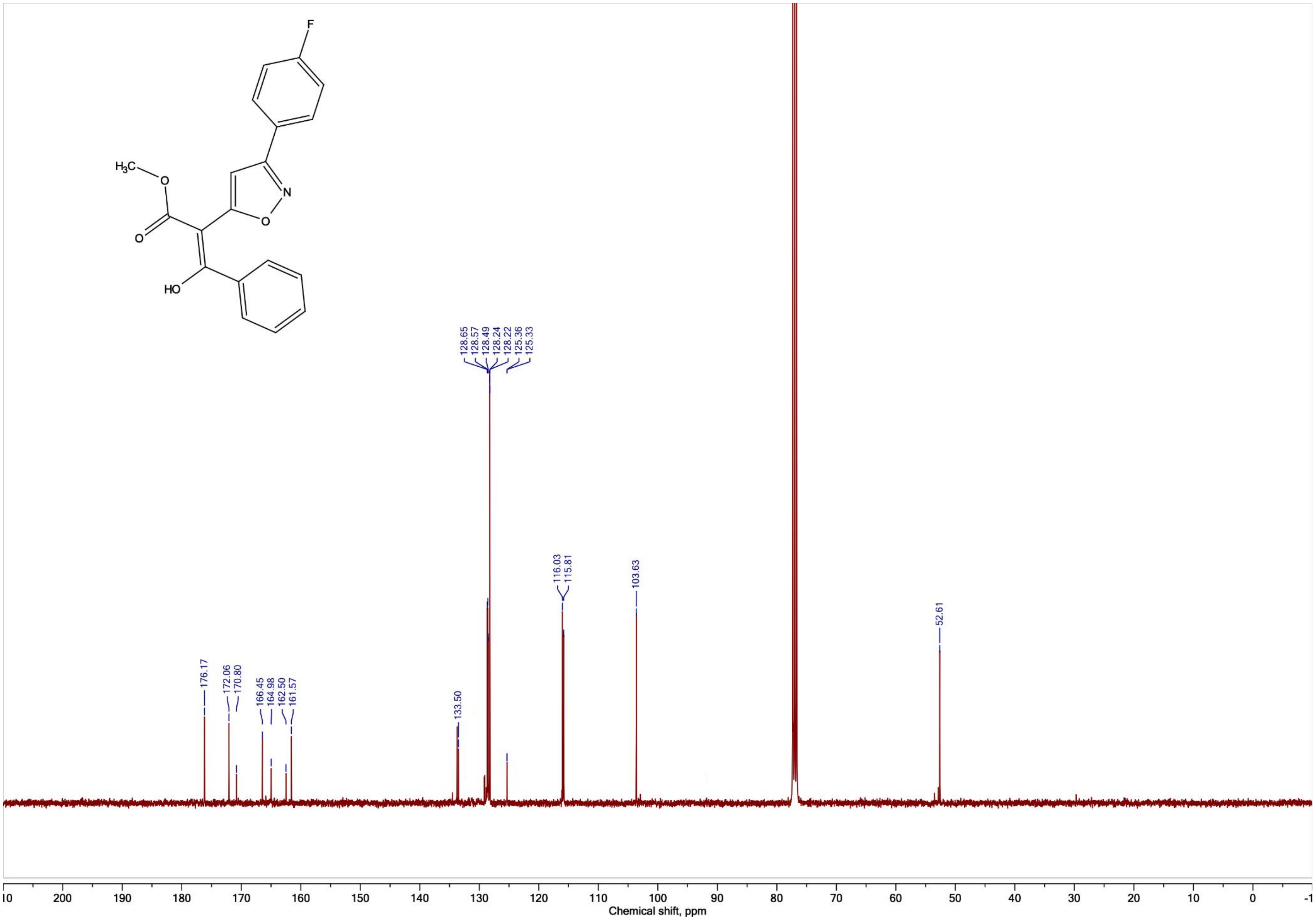

Methyl 2-(3-(4-fluorophenyl)isoxazol-5-yl)-3-oxo-3-phenylpropanoate (1e), DEPT, $CDCl_3$, 101 MHz

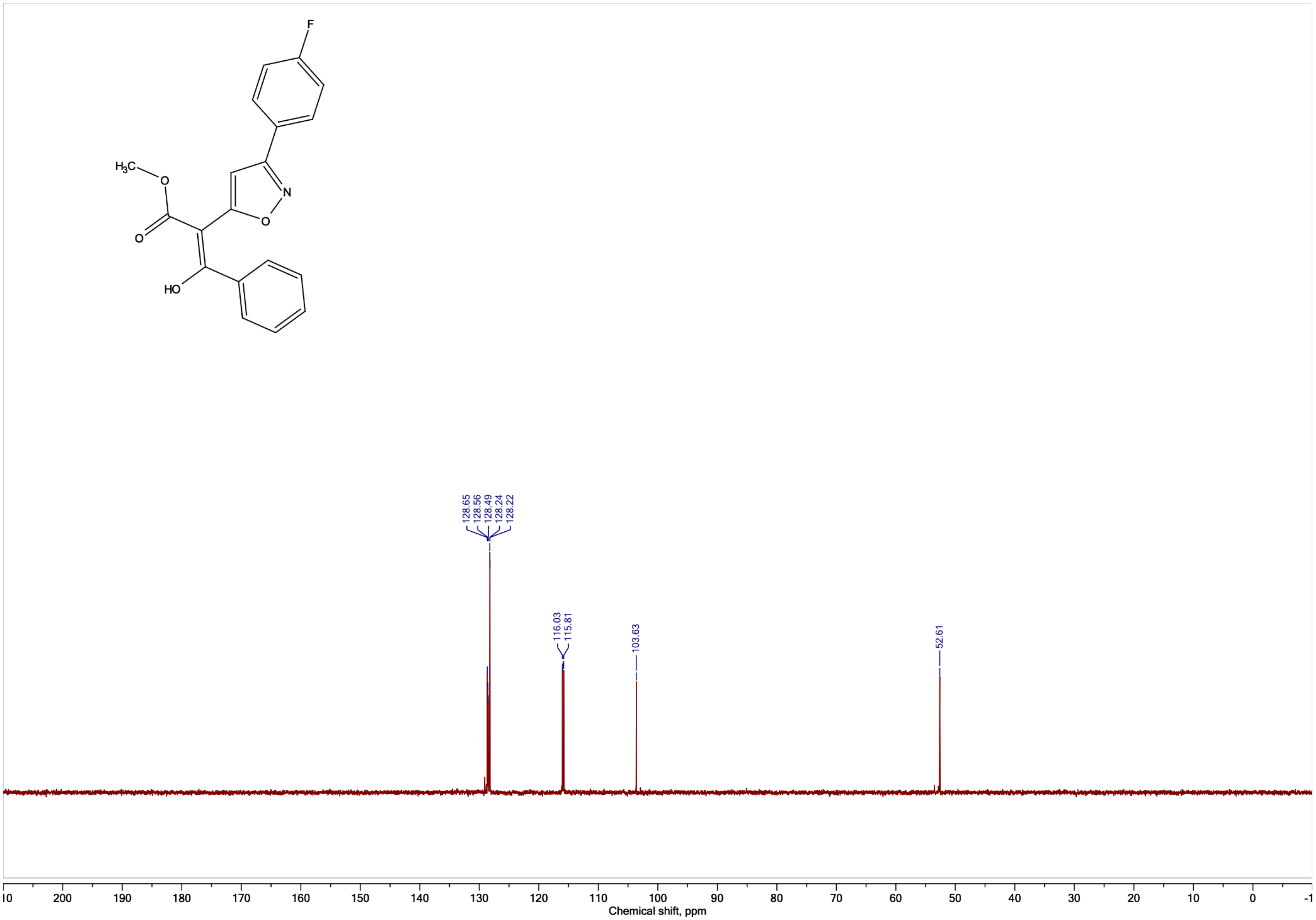

Methyl 2-(3-(4-chlorophenyl)isoxazol-5-yl)-3-oxo-3-phenylpropanoate (1f), ^1H NMR, $CDCl_3$, 400 MHz

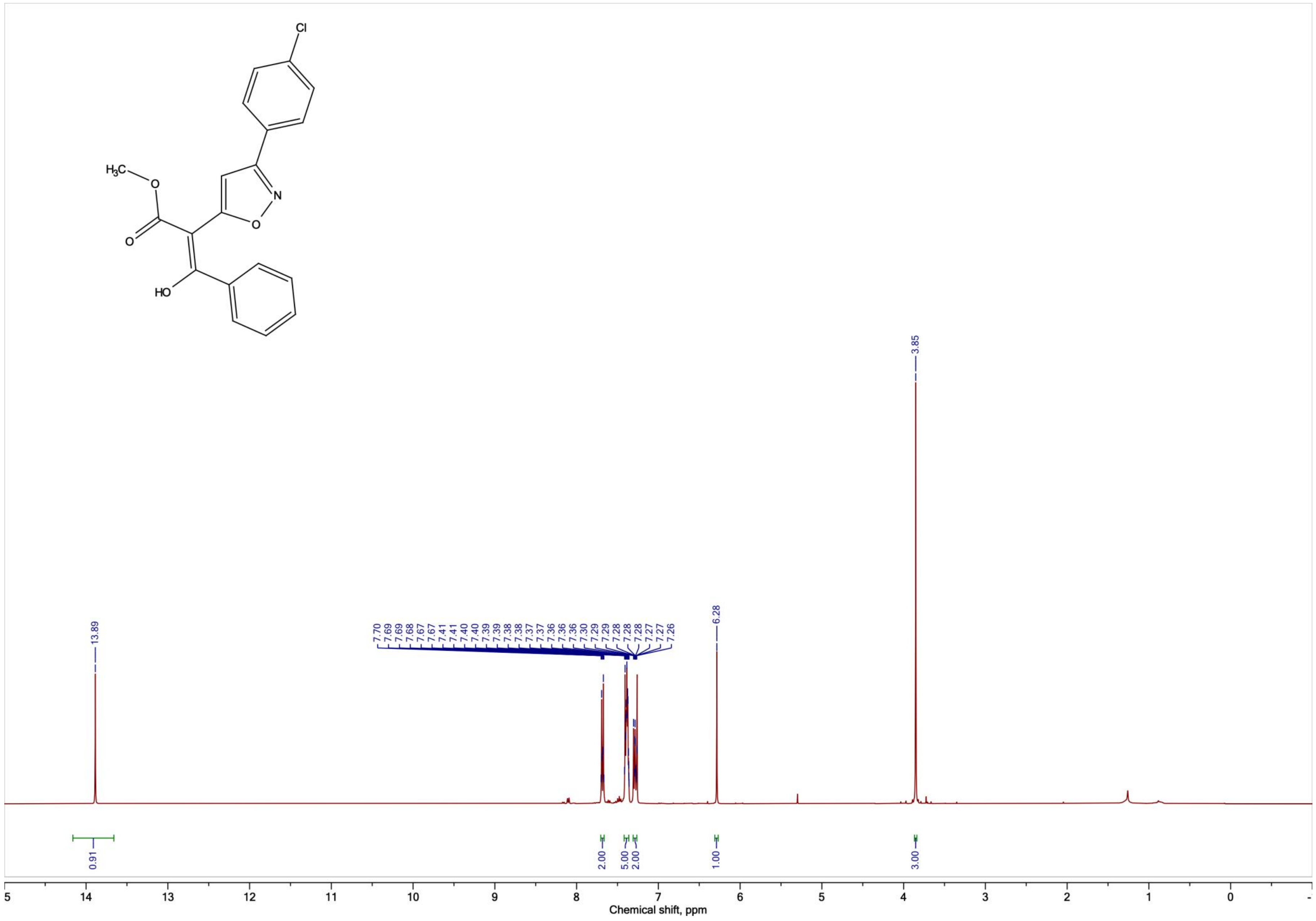

Methyl 2-(3-(4-chlorophenyl)isoxazol-5-yl)-3-oxo-3-phenylpropanoate (1f), $^{13}C\{^{1}H\}$ NMR, $CDCl_3$, 101 MHz

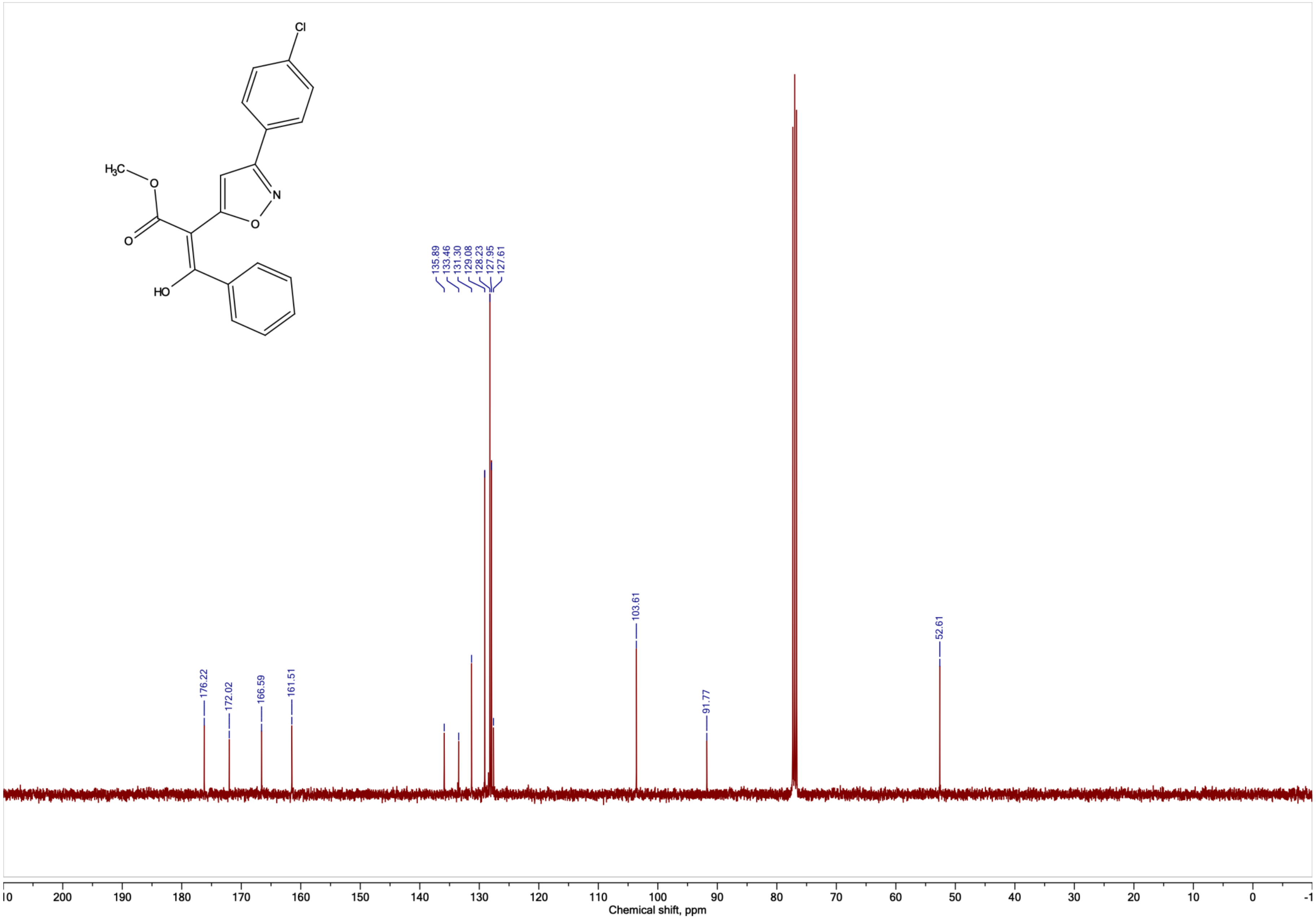

Methyl 2-(3-(4-chlorophenyl)isoxazol-5-yl)-3-oxo-3-phenylpropanoate (1f), DEPT, $CDCl_3$, 101 MHz

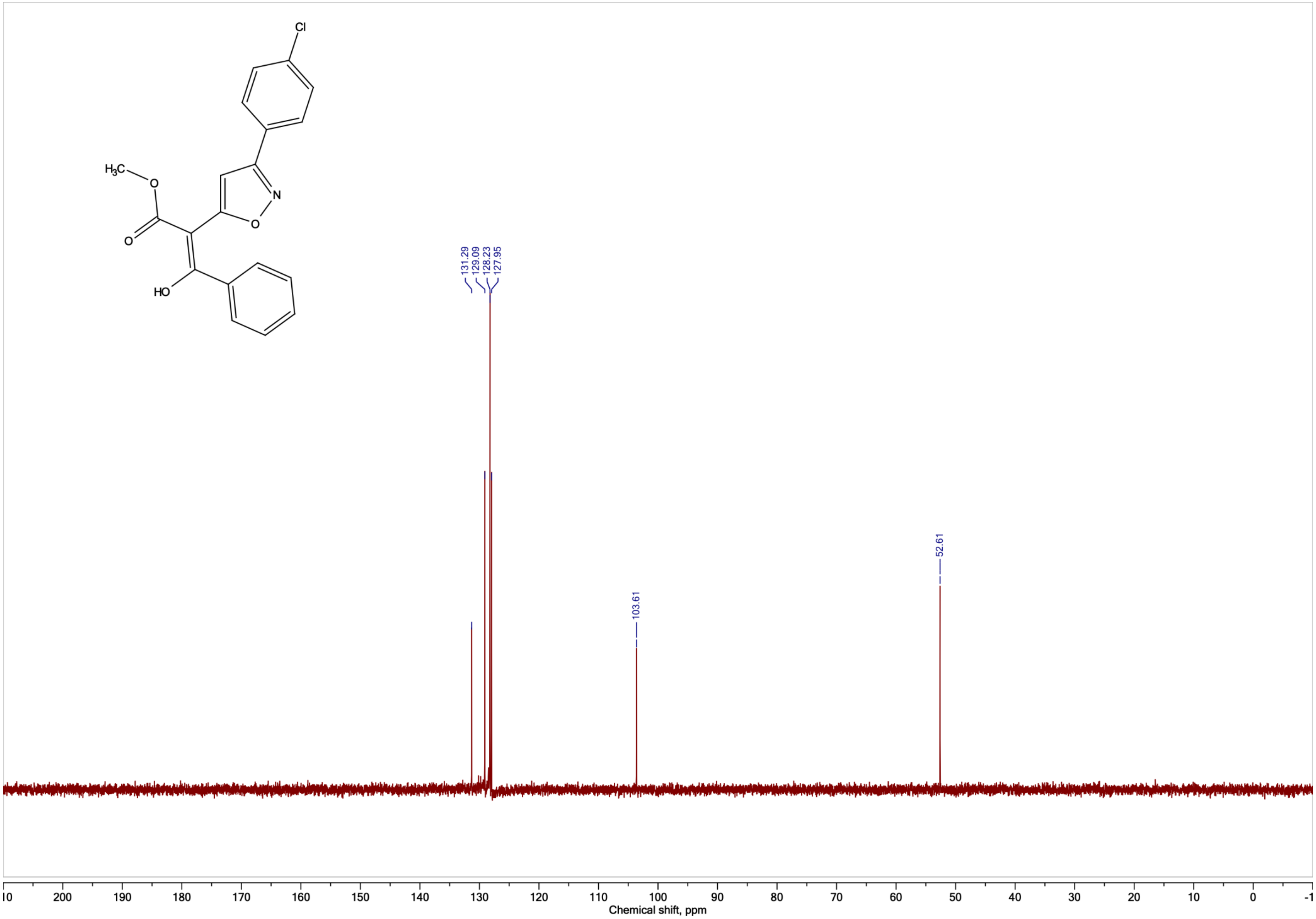

Methyl 2-(3-(4-chlorophenyl)isoxazol-5-yl)-3-oxo-3-(*p*-tolyl)propanoate (1g), ^{1}H NMR, CDCl$_3$, 400 MHz

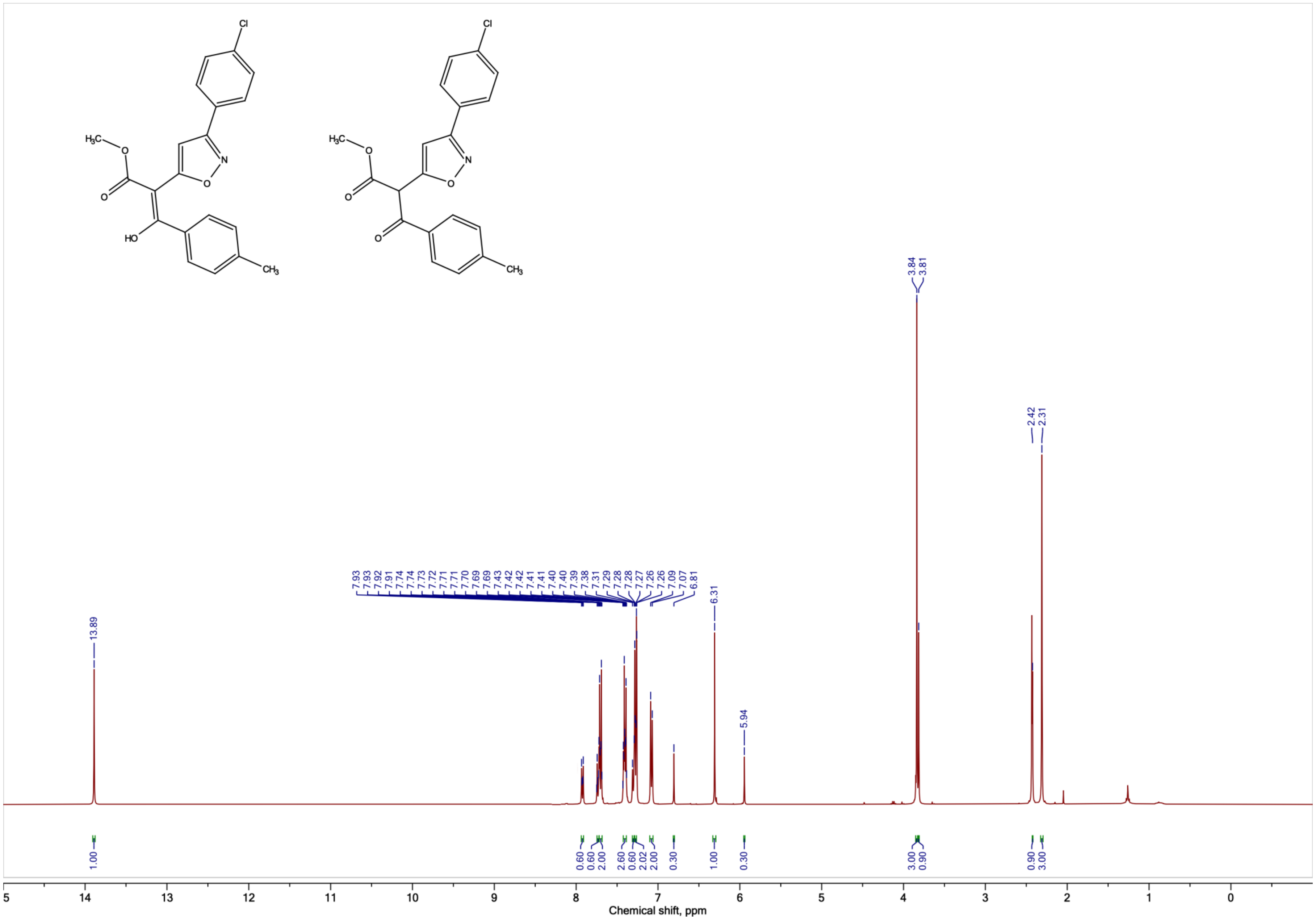

Methyl 2-(3-(4-chlorophenyl)isoxazol-5-yl)-3-oxo-3-(*p*-tolyl)propanoate (1g), $^{13}C\{^1H\}$ NMR, $CDCl_3$, 101 MHz

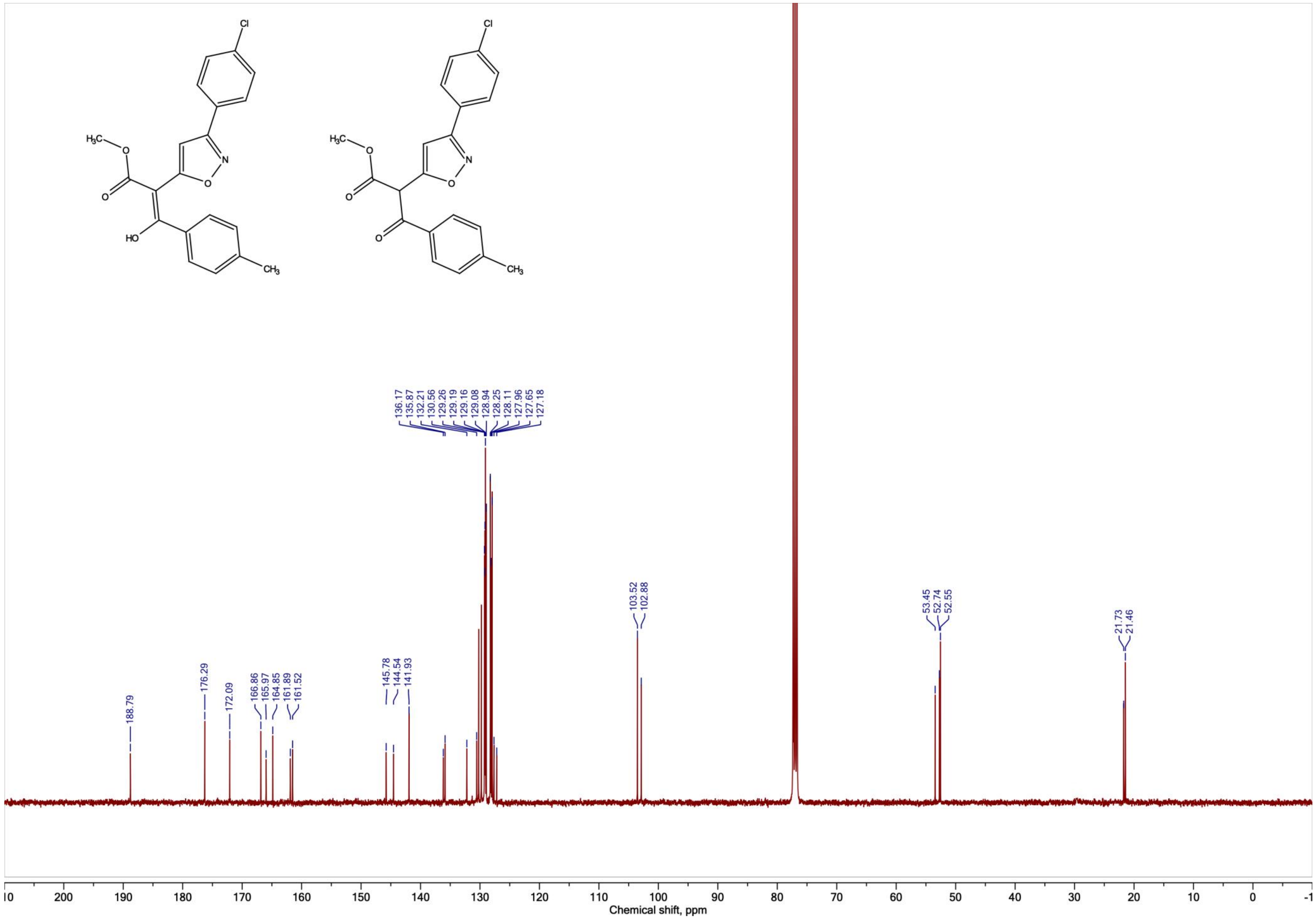

Methyl 2-(3-(4-chlorophenyl)isoxazol-5-yl)-3-oxo-3-(*p*-tolyl)propanoate (1g), DEPT, $CDCl_3$, 101 MHz

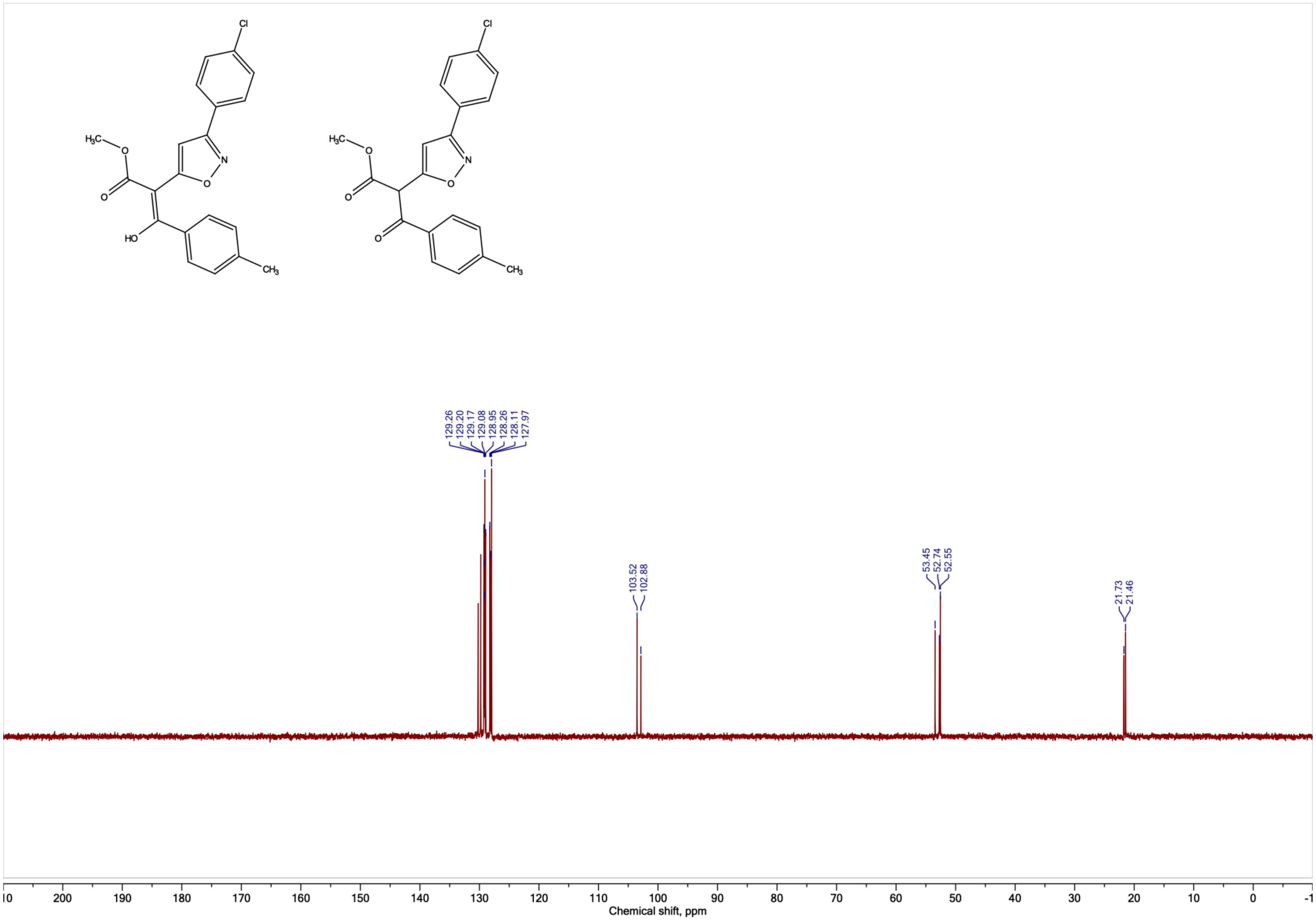

Methyl 2-(3-(4-bromophenyl)isoxazol-5-yl)-3-oxo-3-phenylpropanoate (1h), ^1H NMR, $CDCl_3$, 400 MHz

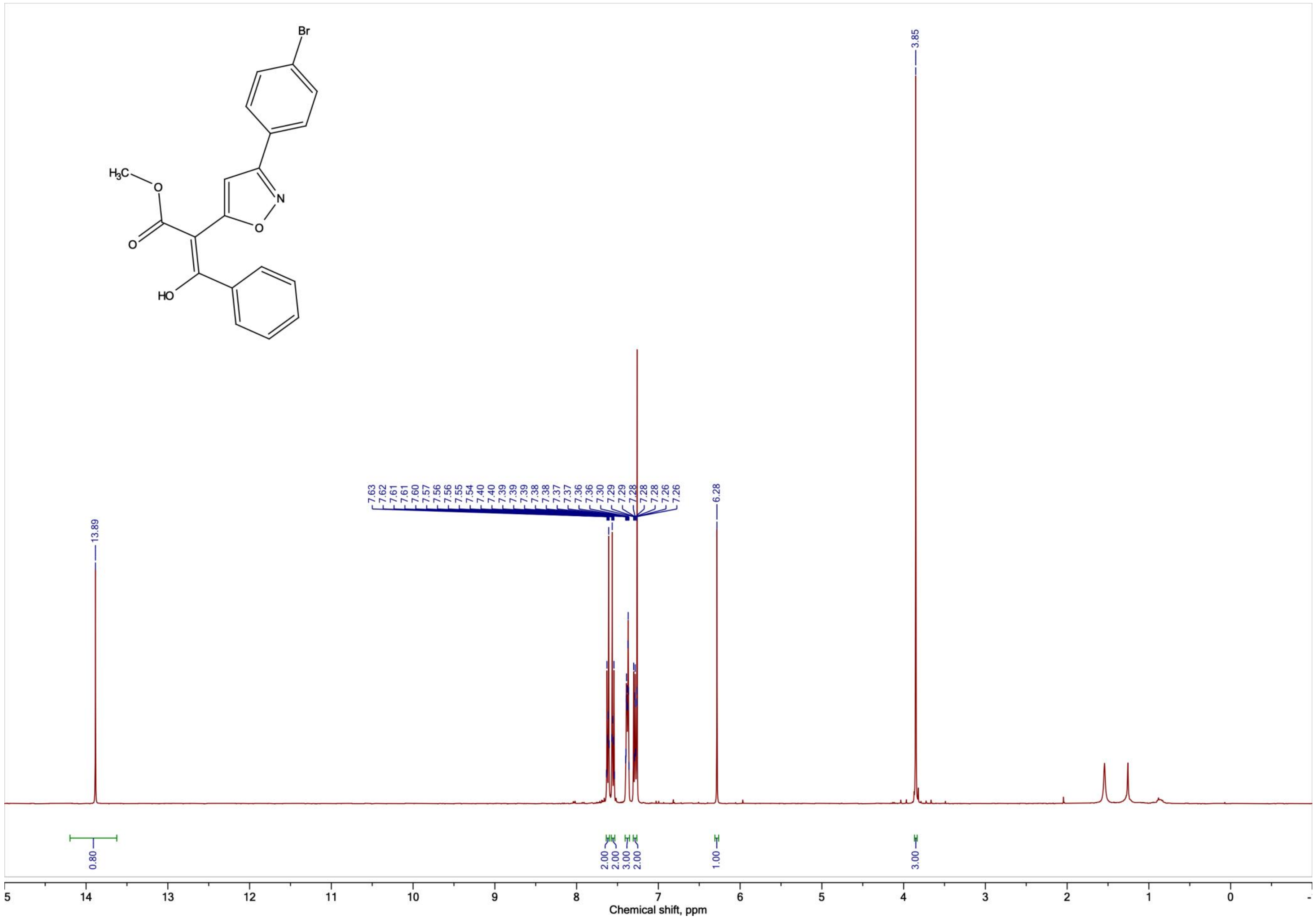

Methyl 2-(3-(4-bromophenyl)isoxazol-5-yl)-3-oxo-3-phenylpropanoate (1h), $^{13}C\{^1H\}$ NMR, $CDCl_3$, 101 MHz

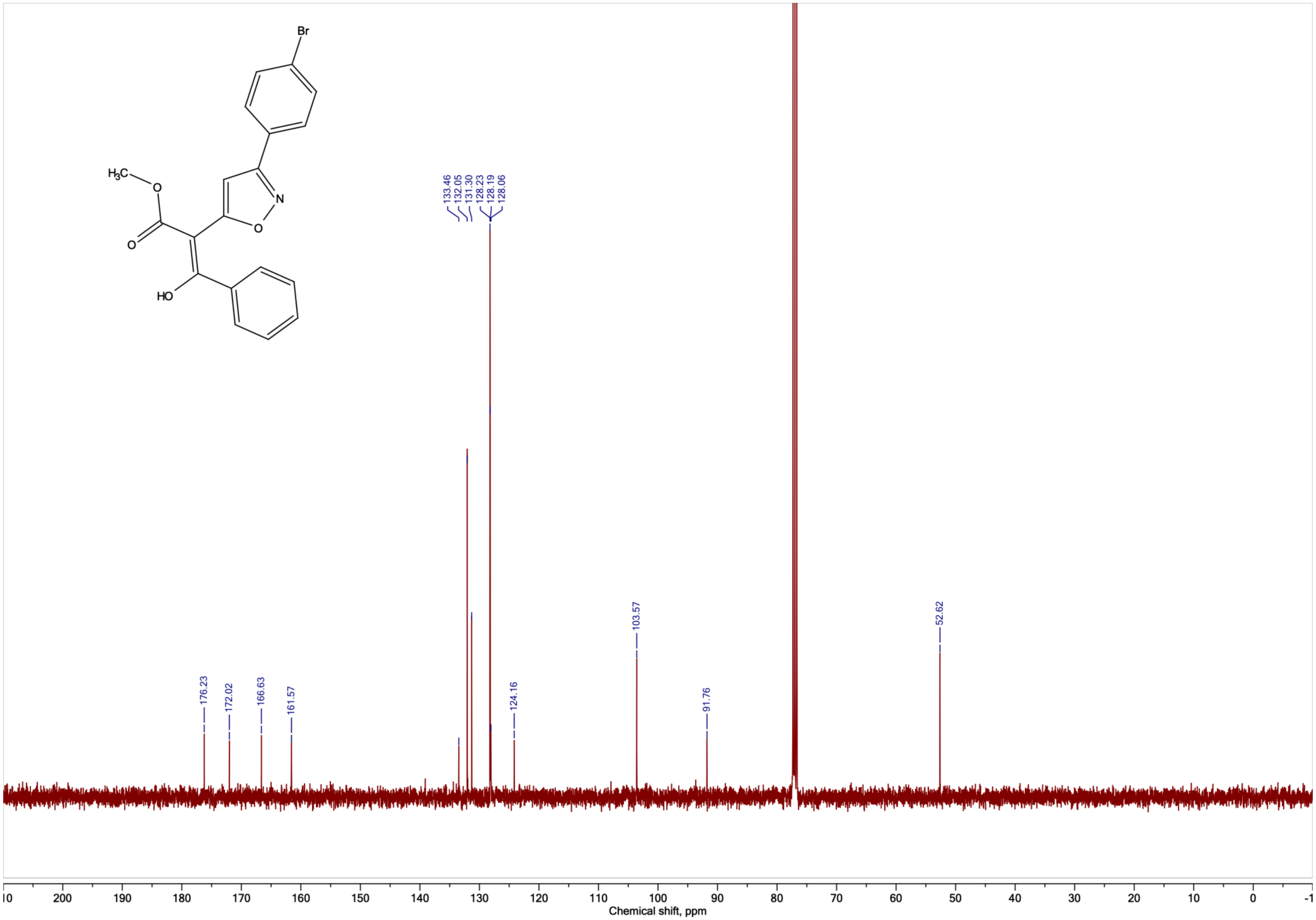

Methyl 2-(3-(4-bromophenyl)isoxazol-5-yl)-3-oxo-3-phenylpropanoate (1h), DEPT, $CDCl_3$, 101 MHz

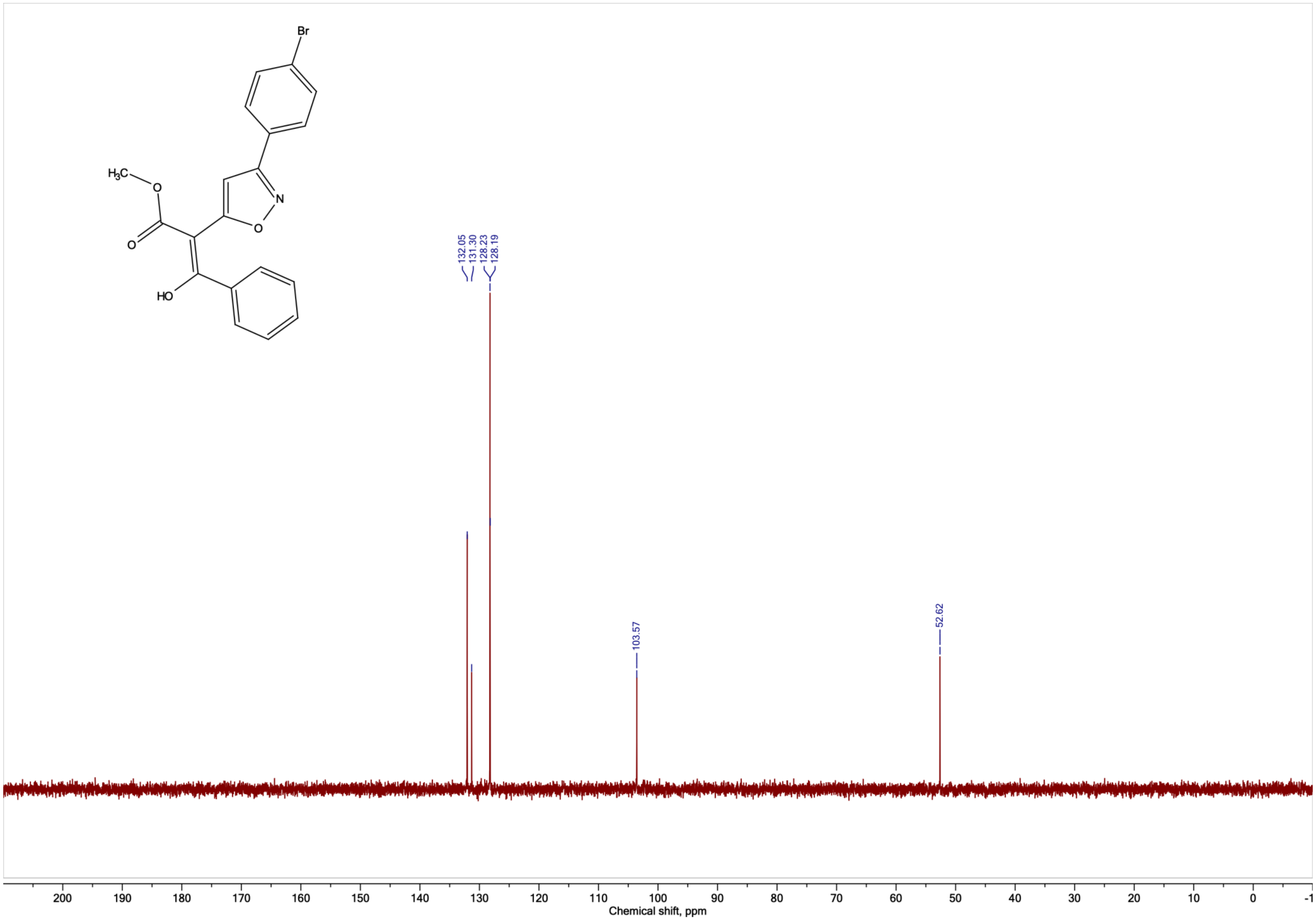

Methyl 2-(3-(4-bromophenyl)isoxazol-5-yl)-3-oxobutanoate (1i), ^{1}H NMR, $CDCl_3$, 400 MHz

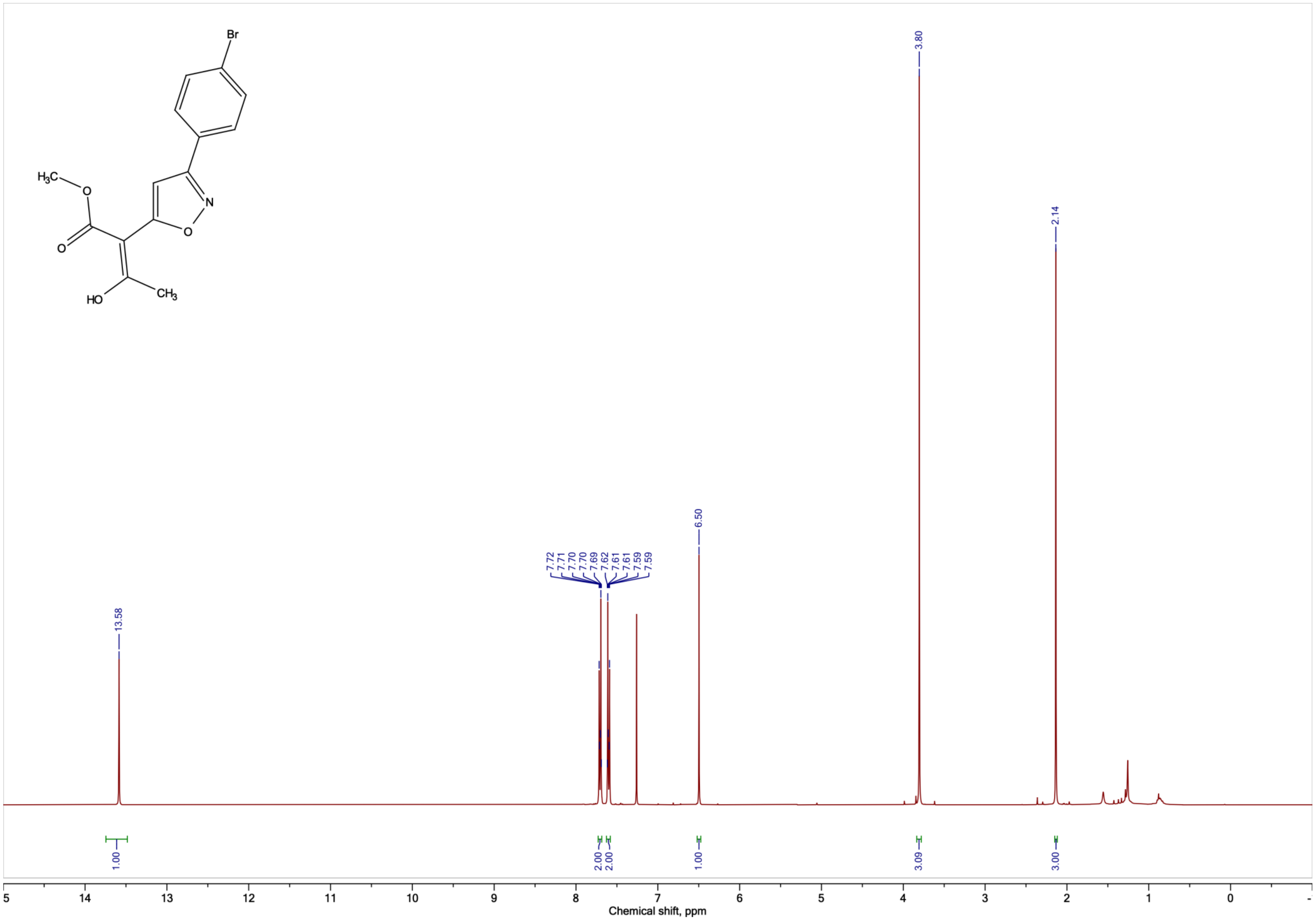

Methyl 2-(3-(4-bromophenyl)isoxazol-5-yl)-3-oxobutanoate (1i), $^{13}C\{^1H\}$ NMR, $CDCl_3$, 101 MHz

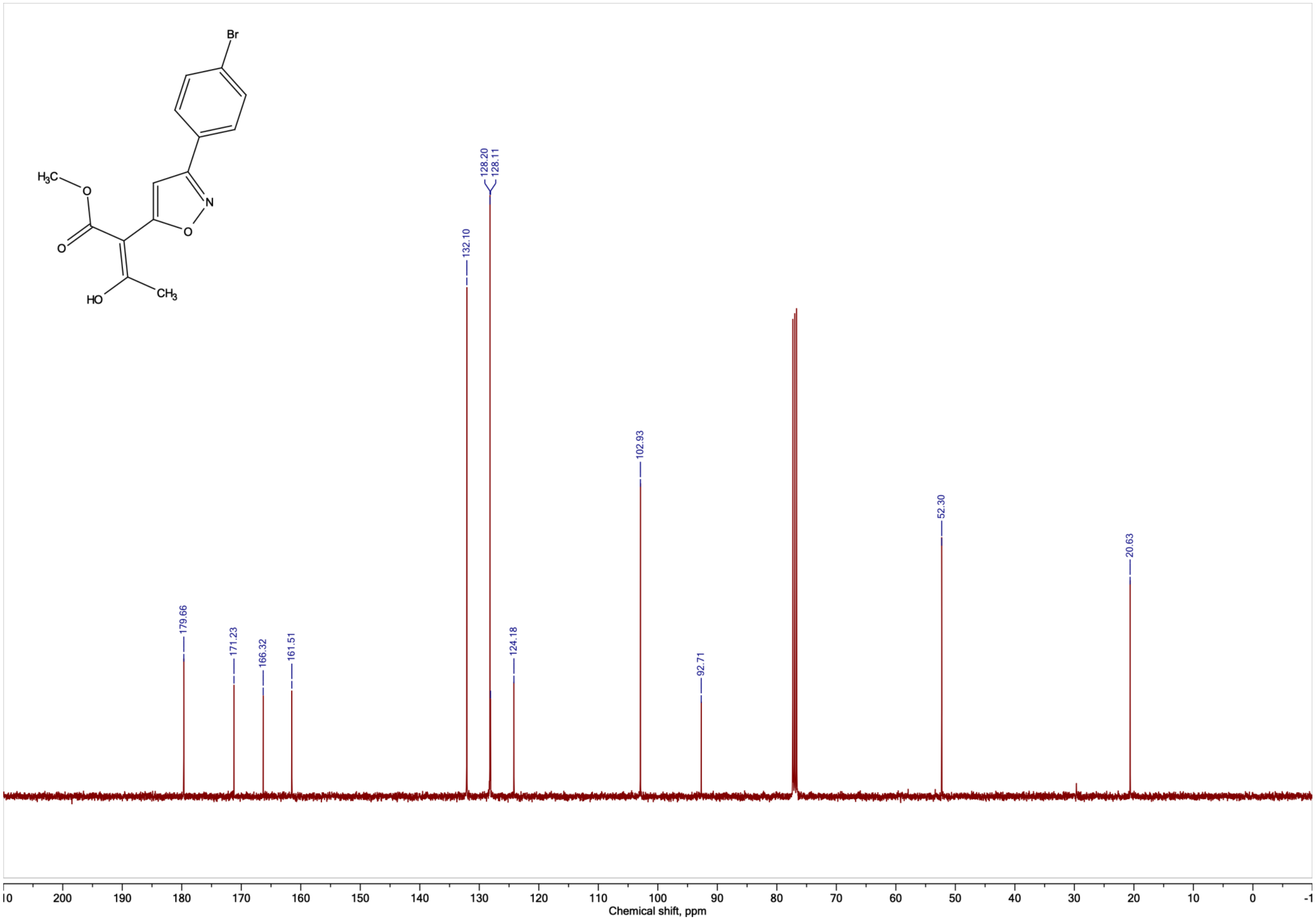

Methyl 2-(3-(4-bromophenyl)isoxazol-5-yl)-3-oxobutanoate (1i), DEPT, $CDCl_3$, 101 MHz

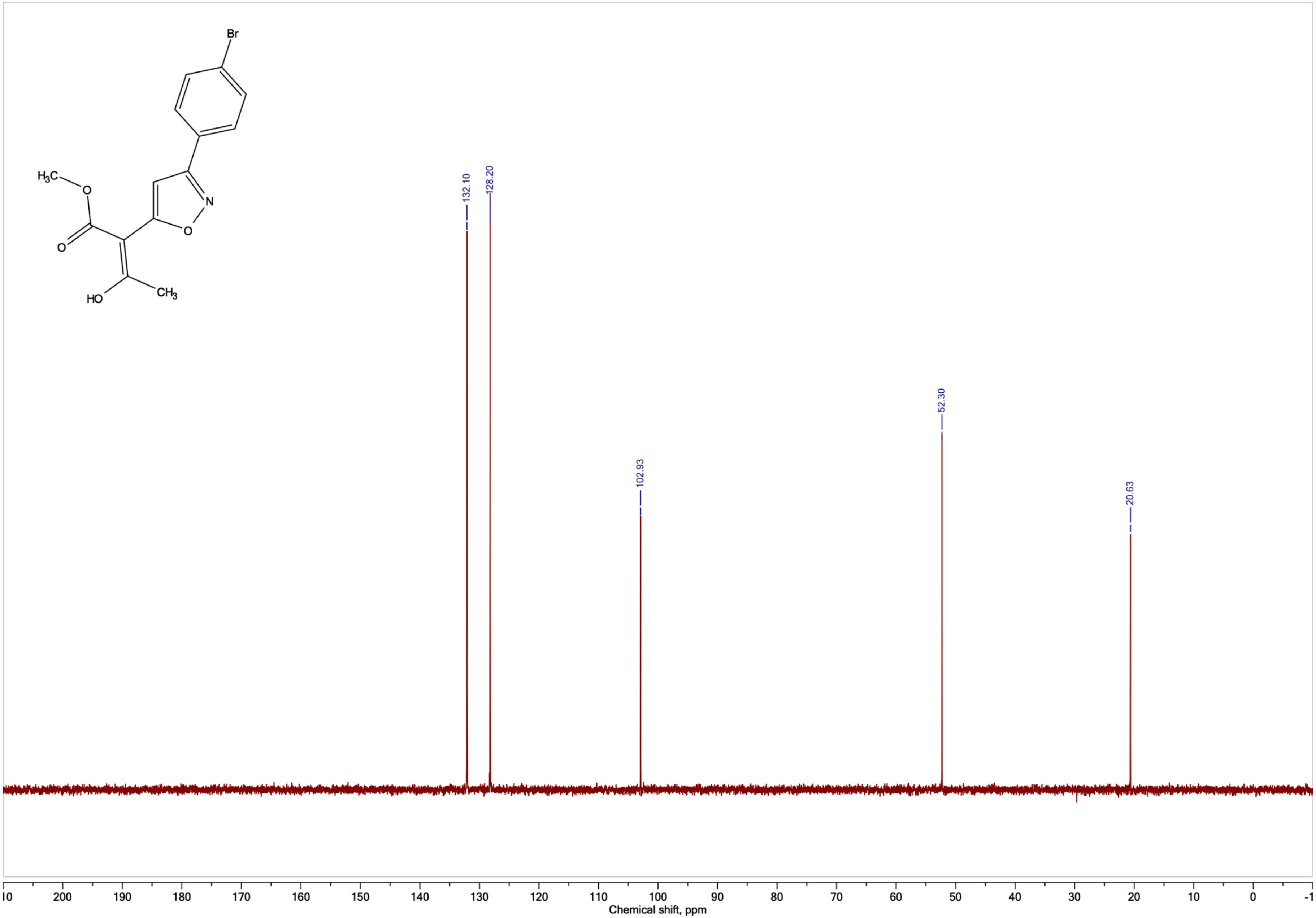

Methyl 3-oxo-2-(3-(quinolin-2-yl)isoxazol-5-yl)-3-(*p*-tolyl)propanoate (1j), ^{1}H NMR, $CDCl_3$, 400 MHz

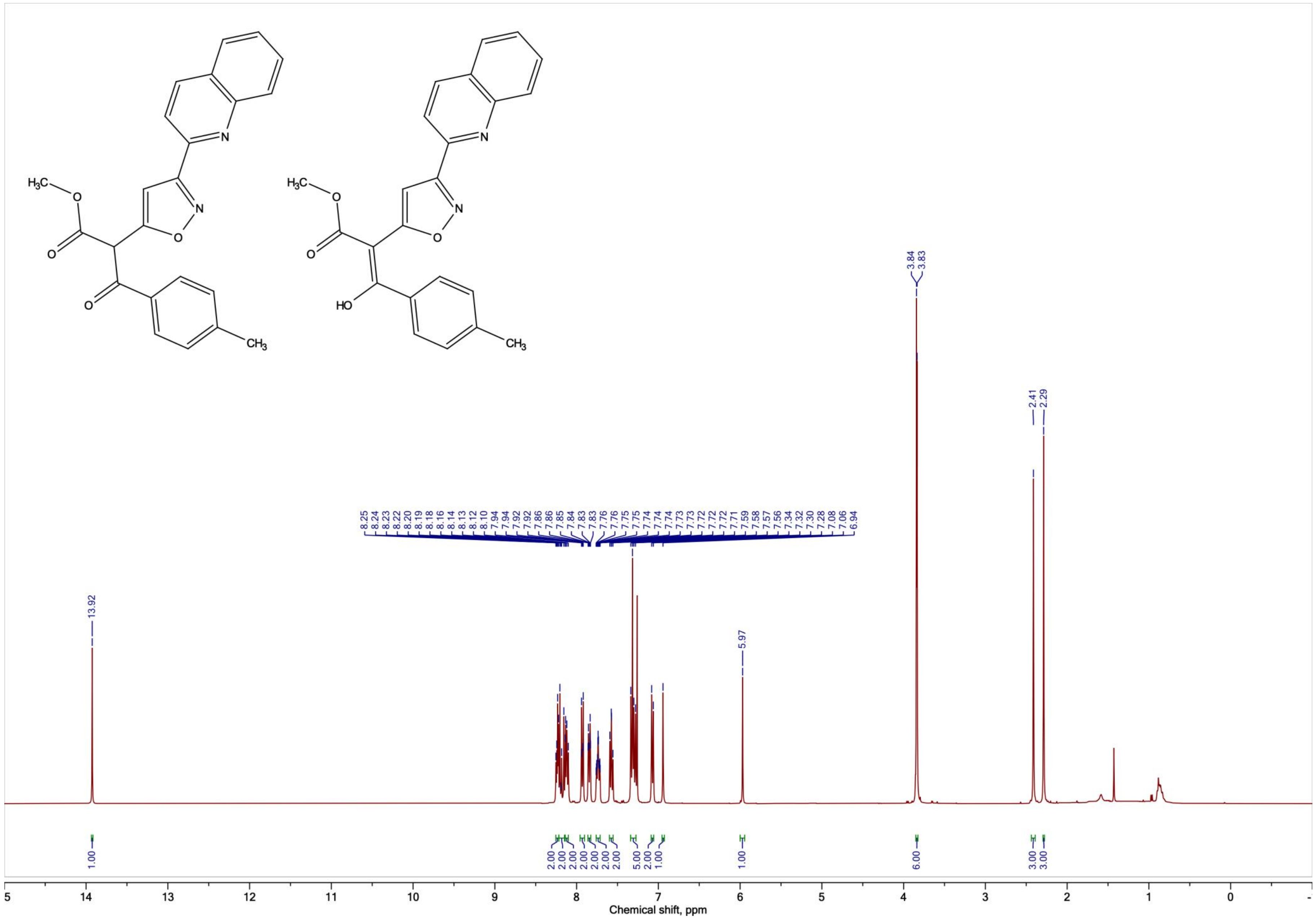

Methyl 3-oxo-2-(3-(quinolin-2-yl)isoxazol-5-yl)-3-(*p*-tolyl)propanoate (1j), $^{13}C\{^{1}H\}$ NMR, $CDCl_3$, 101 MHz

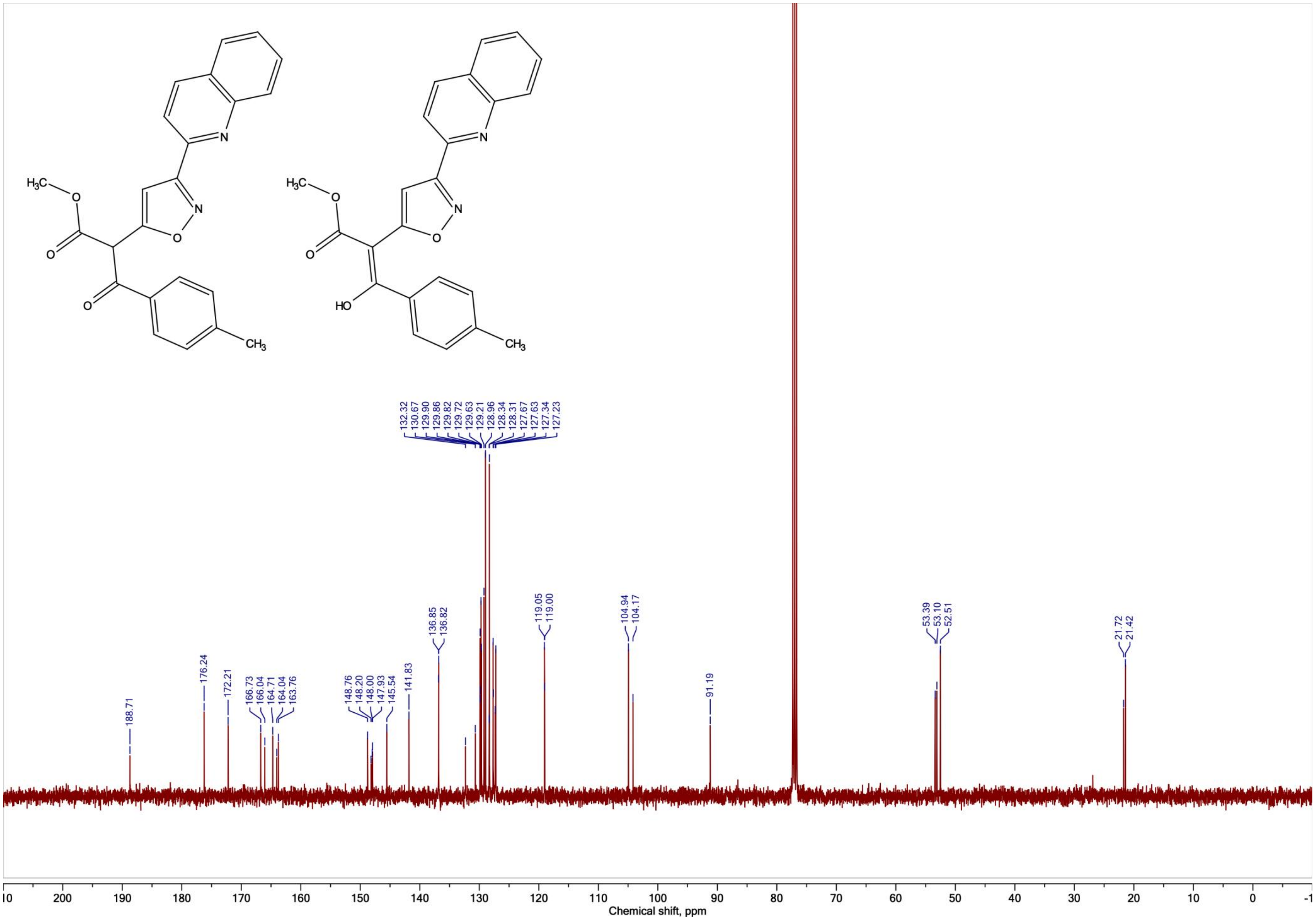

Methyl 3-oxo-2-(3-(quinolin-2-yl)isoxazol-5-yl)-3-(*p*-tolyl)propanoate (1j), DEPT, $CDCl_3$, 101 MHz

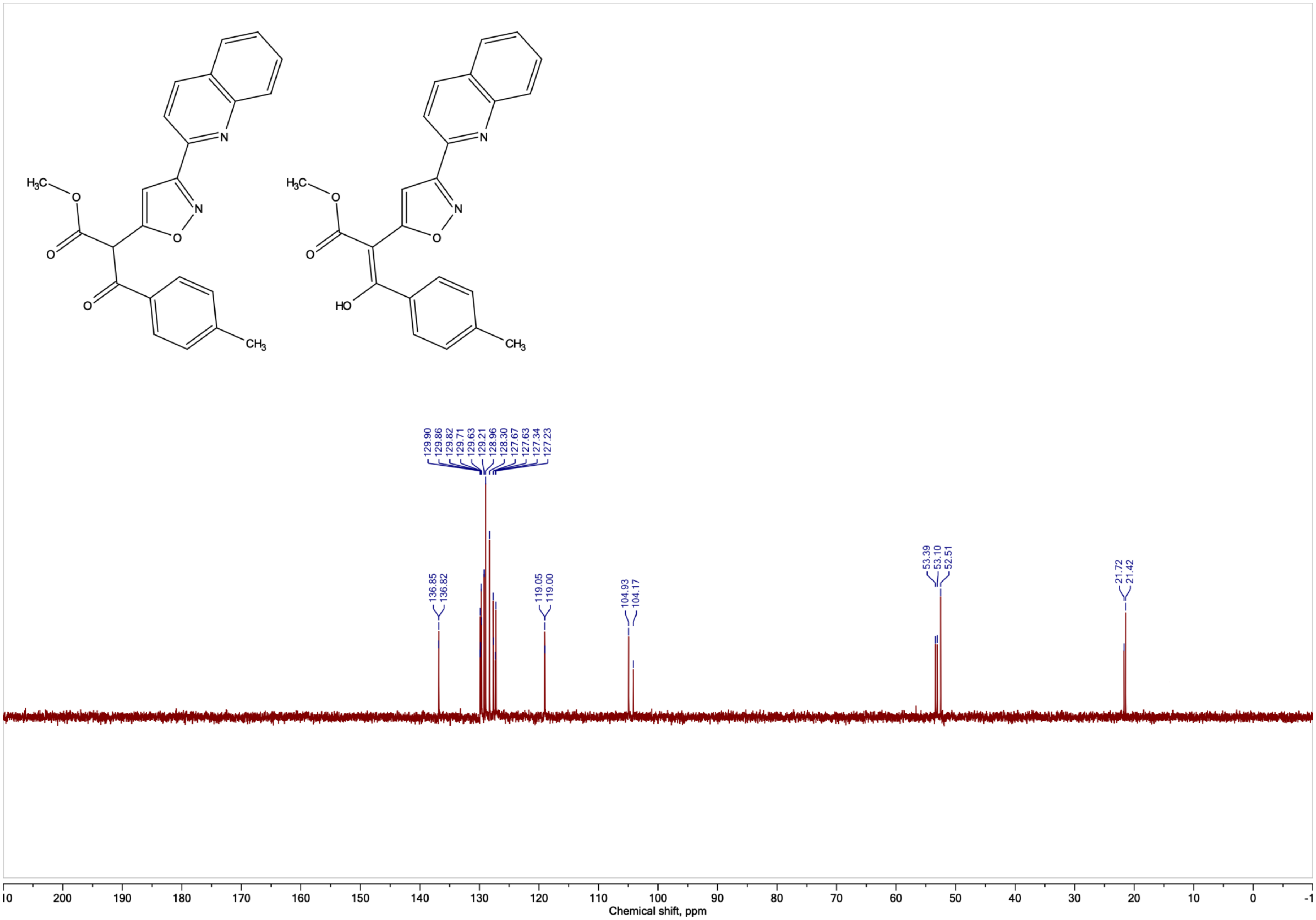

Methyl 2-(3-benzylisoxazol-5-yl)-3-oxo-3-(*p*-tolyl)propanoate (1k), ^{1}H NMR, $CDCl_3$, 400 MHz

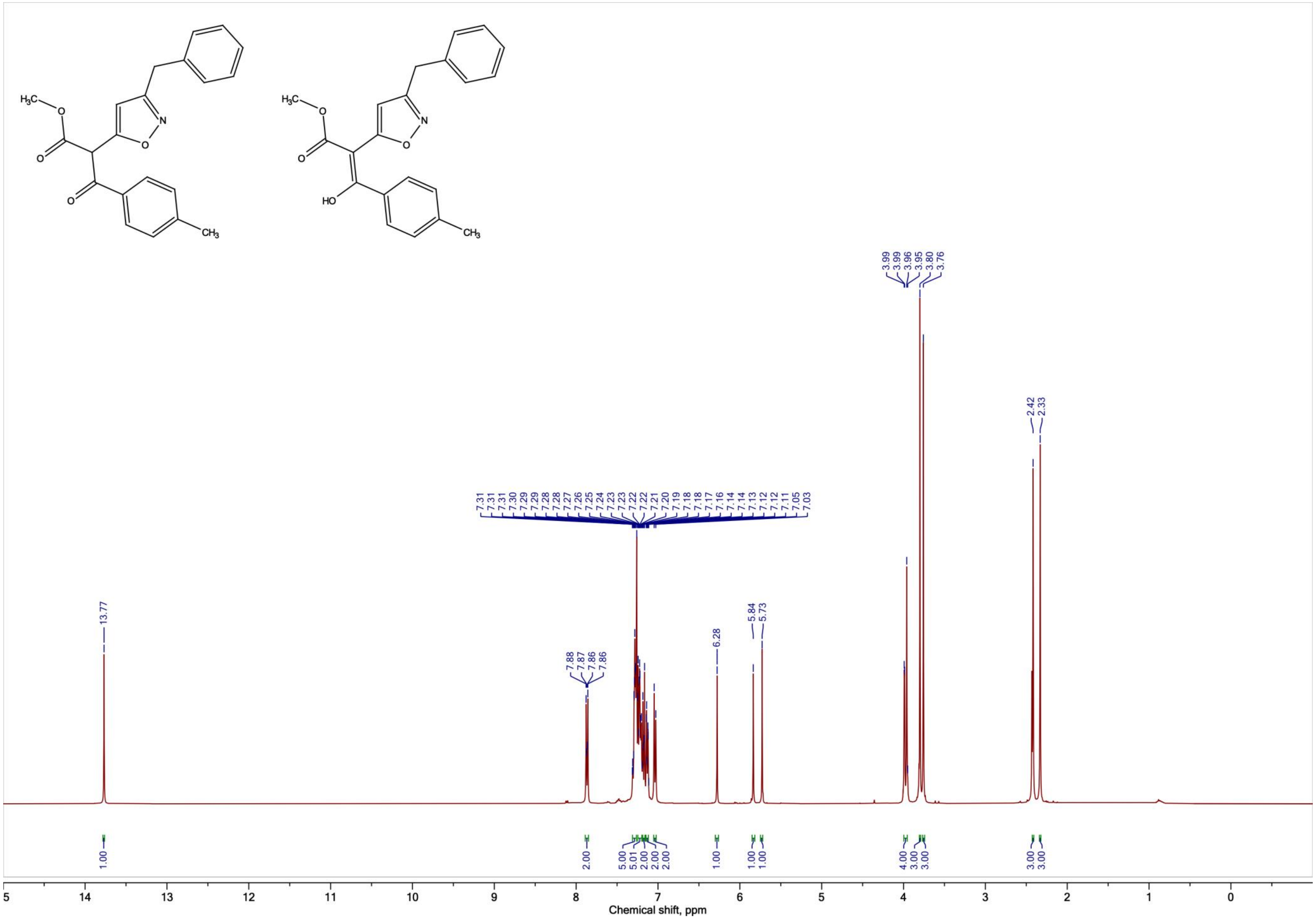

Methyl 2-(3-benzylisoxazol-5-yl)-3-oxo-3-(*p*-tolyl)propanoate (1k), $^{13}C\{^1H\}$ NMR, $CDCl_3$, 101 MHz

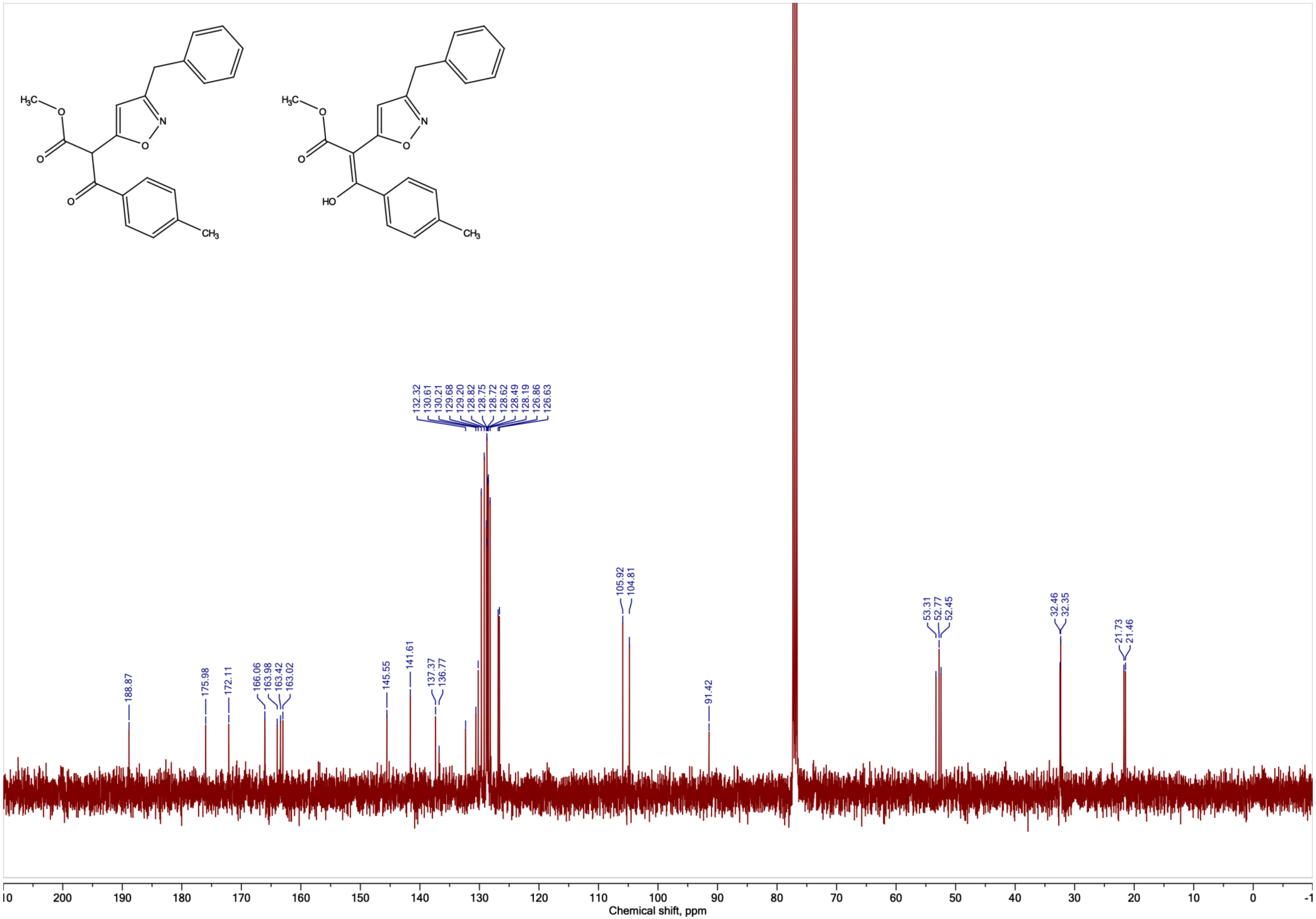

Methyl 2-(3-benzylisoxazol-5-yl)-3-oxo-3-(*p*-tolyl)propanoate (1k), DEPT, $CDCl_3$, 101 MHz

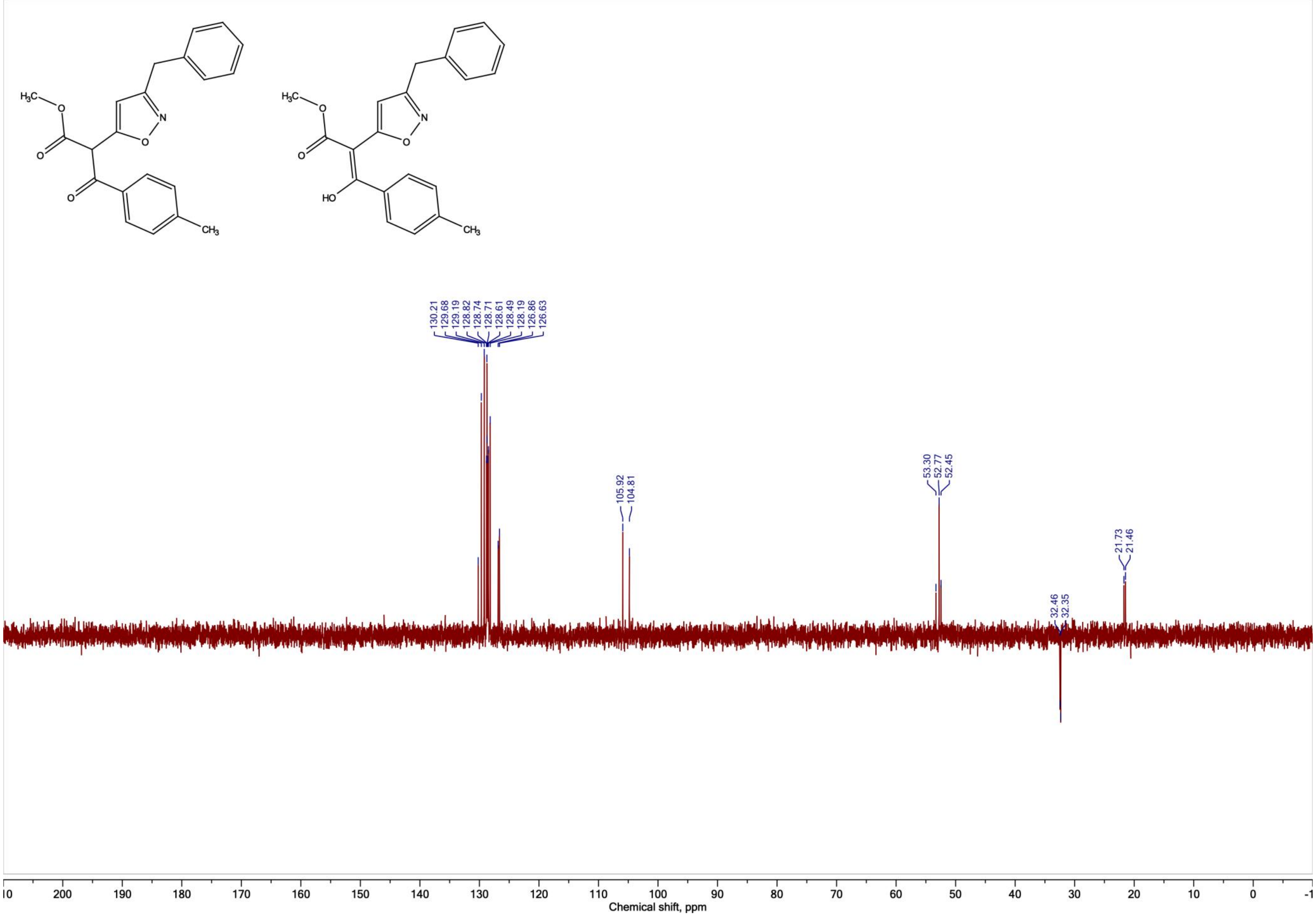

Methyl 2-(3-heptylisoxazol-5-yl)-3-oxo-3-(*p*-tolyl)propanoate (1l), ^{1}H NMR, CDCl$_3$, 400 MHz

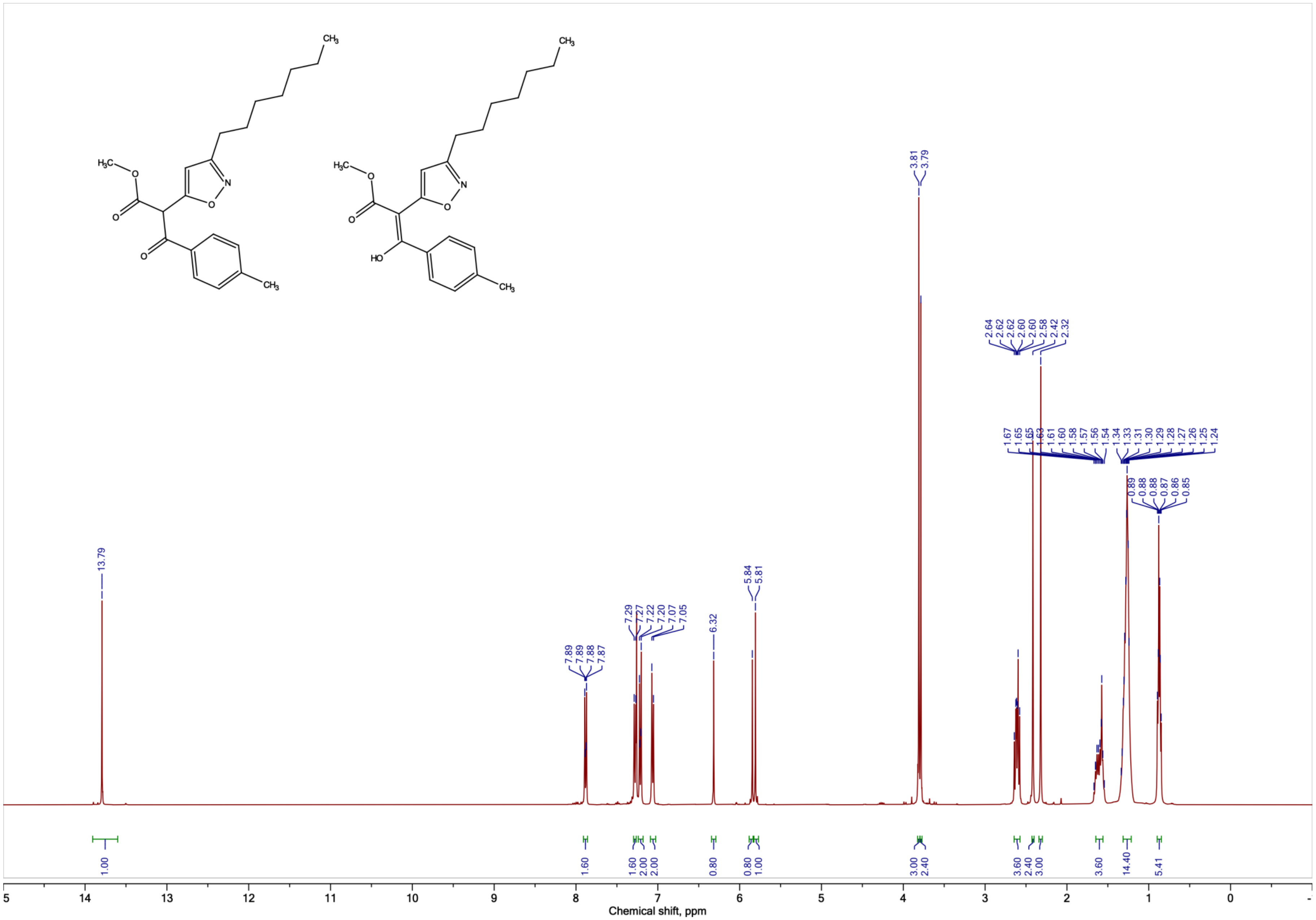

Methyl 2-(3-heptylisoxazol-5-yl)-3-oxo-3-(*p*-tolyl)propanoate (1l), $^{13}C\{^{1}H\}$ NMR, $CDCl_3$, 101 MHz

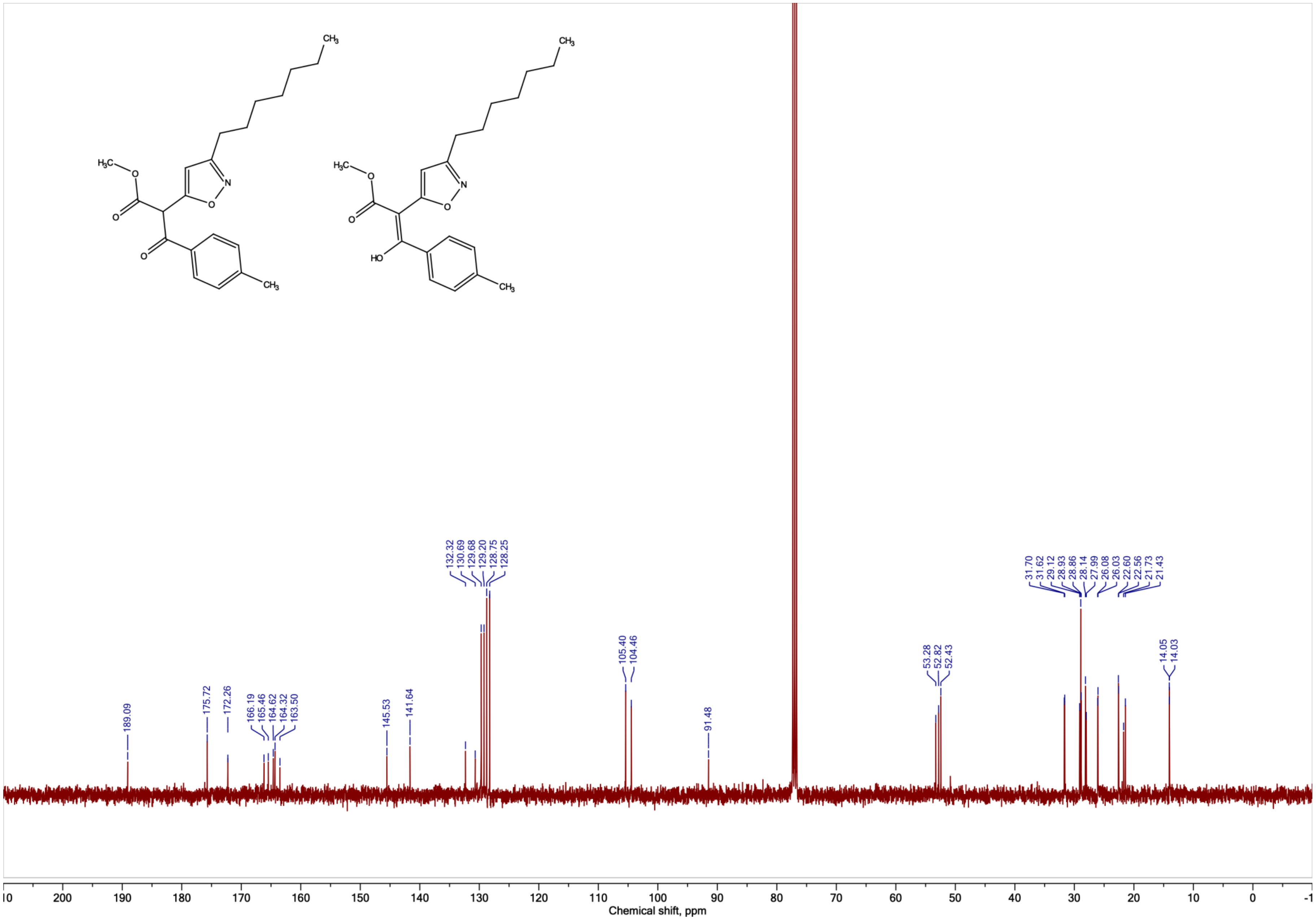

Methyl 2-(3-heptylisoxazol-5-yl)-3-oxo-3-(*p*-tolyl)propanoate (1l), DEPT, $CDCl_3$, 101 MHz

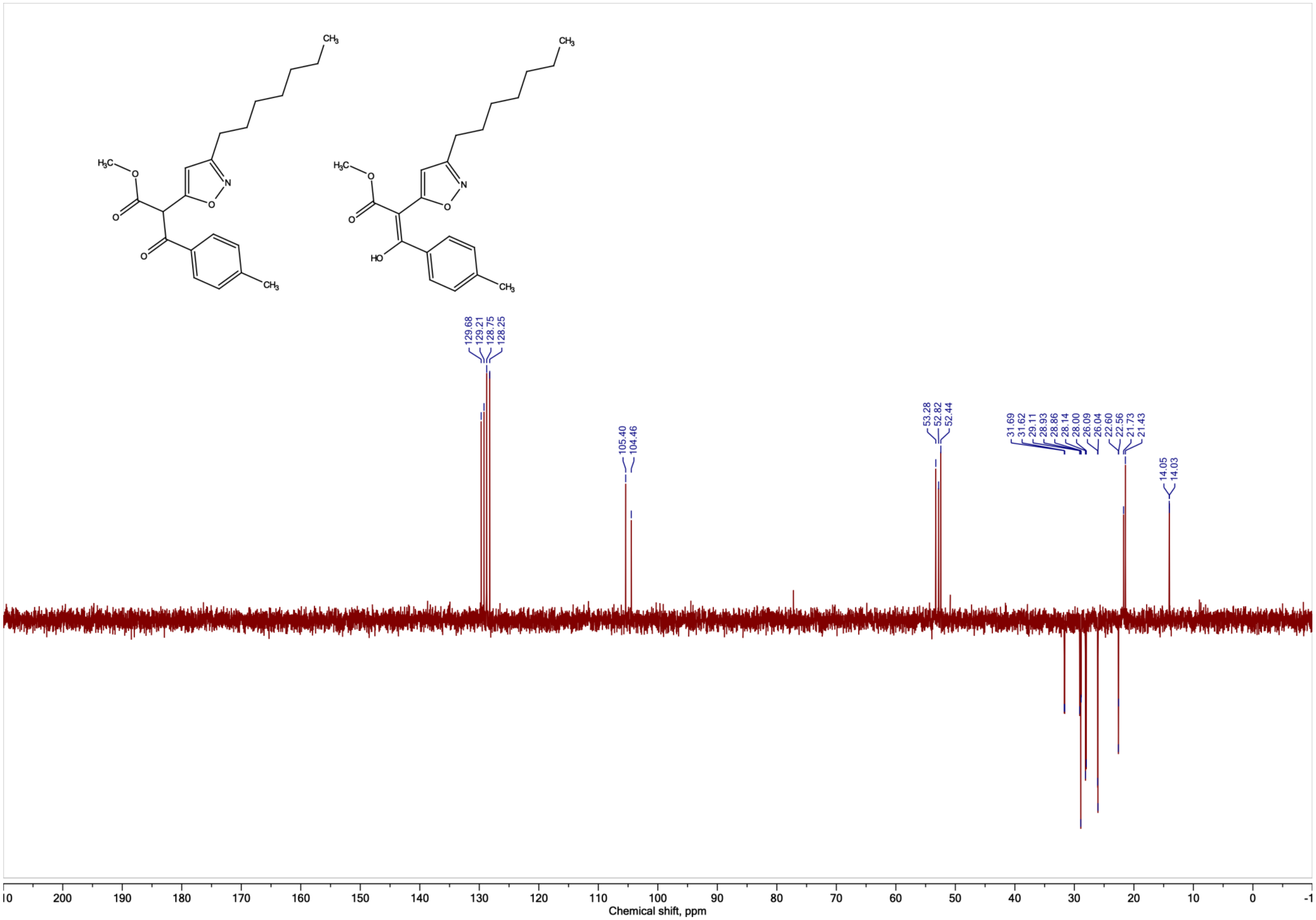

Methyl 2-(3-(4-chlorophenyl)-4-iodoisoxazol-5-yl)-3-oxo-3-phenylpropanoate (1m), ^{1}H NMR, $CDCl_3$, 400 MHz

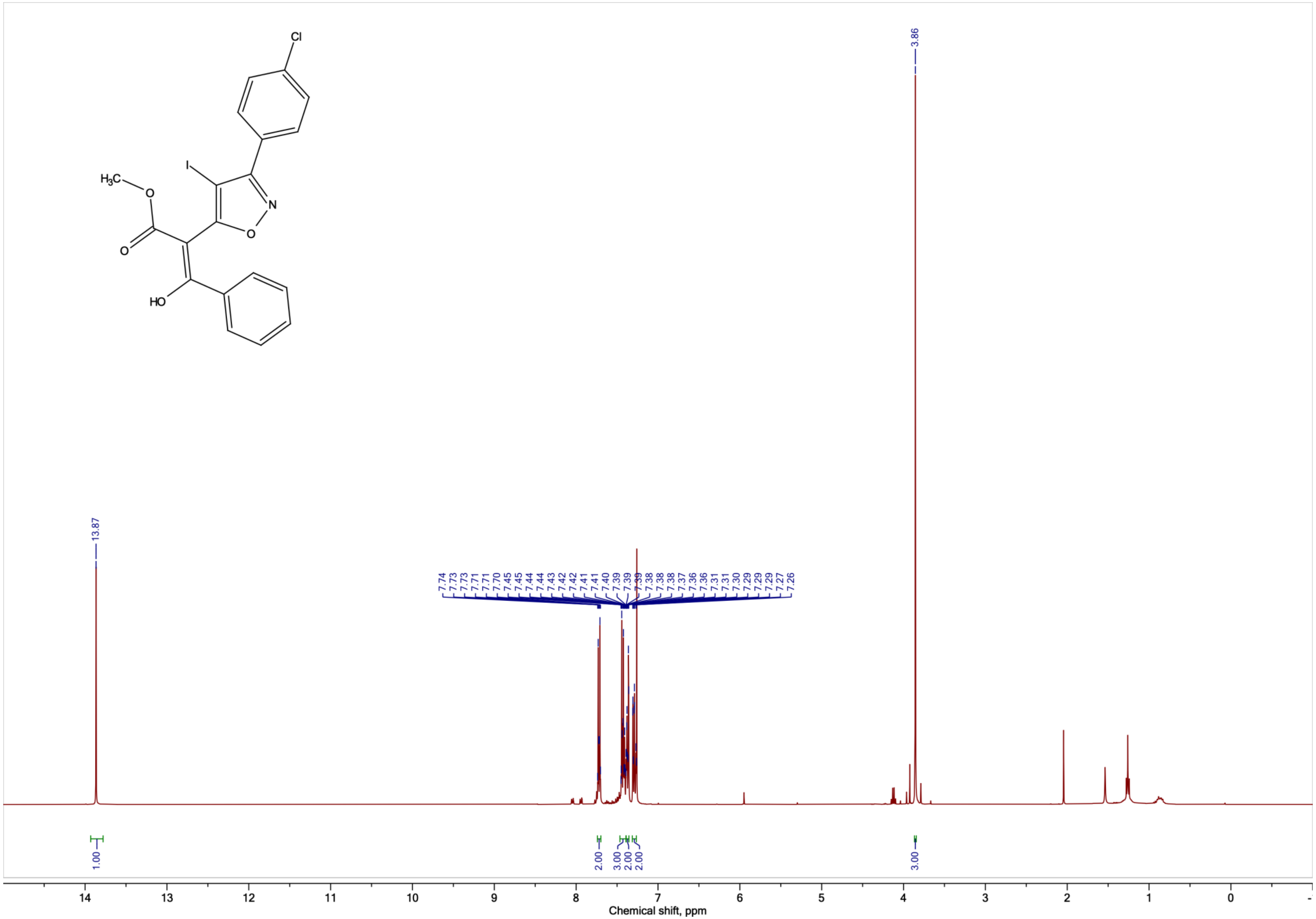

Methyl 2-(3-(4-chlorophenyl)-4-iodoisoxazol-5-yl)-3-oxo-3-phenylpropanoate (1m), $^{13}C\{^1H\}$ NMR, $CDCl_3$, 101 MHz

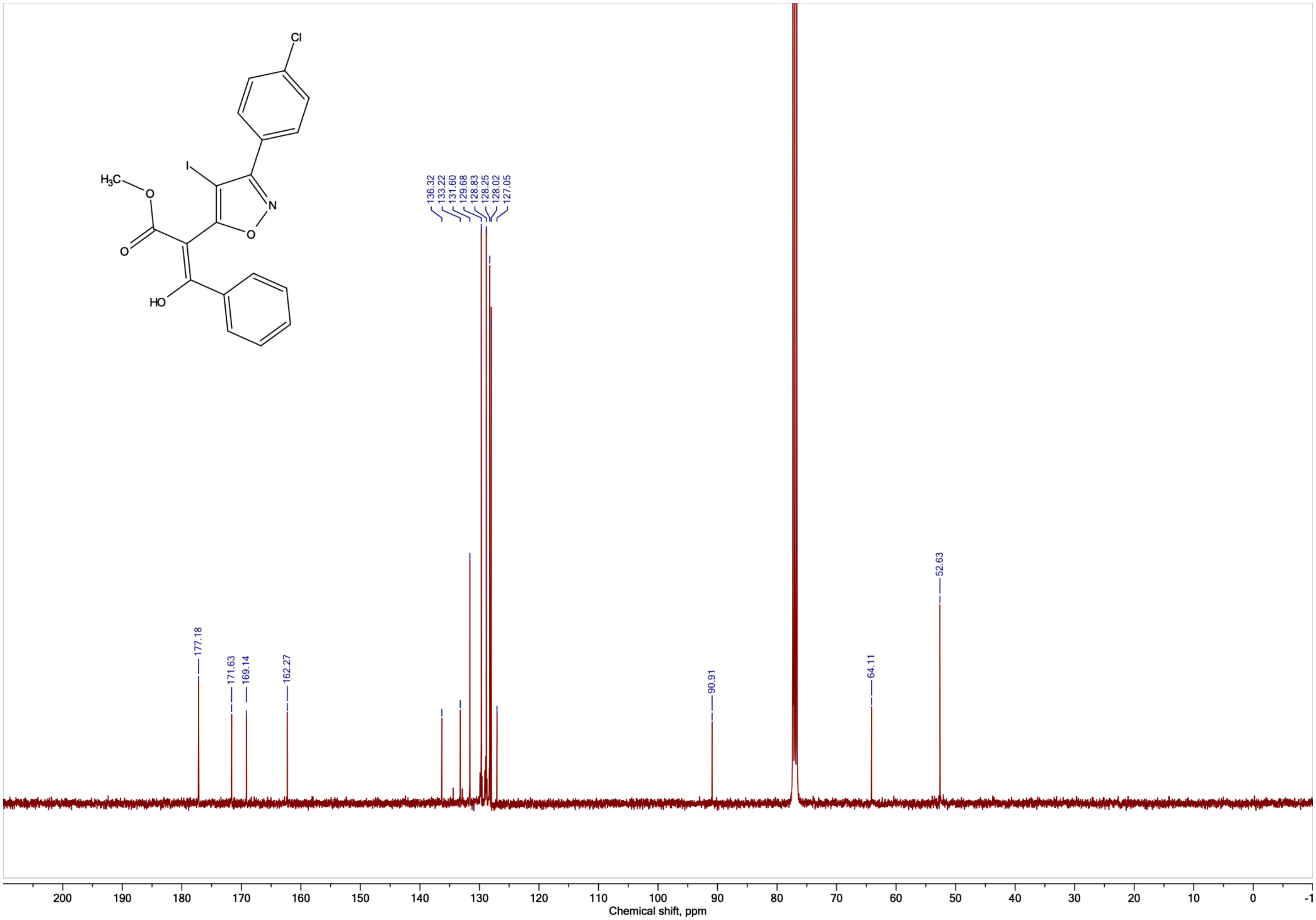

Methyl 2-(3-(4-chlorophenyl)-4-iodoisoxazol-5-yl)-3-oxo-3-phenylpropanoate (1m), DEPT, $CDCl_3$, 101 MHz

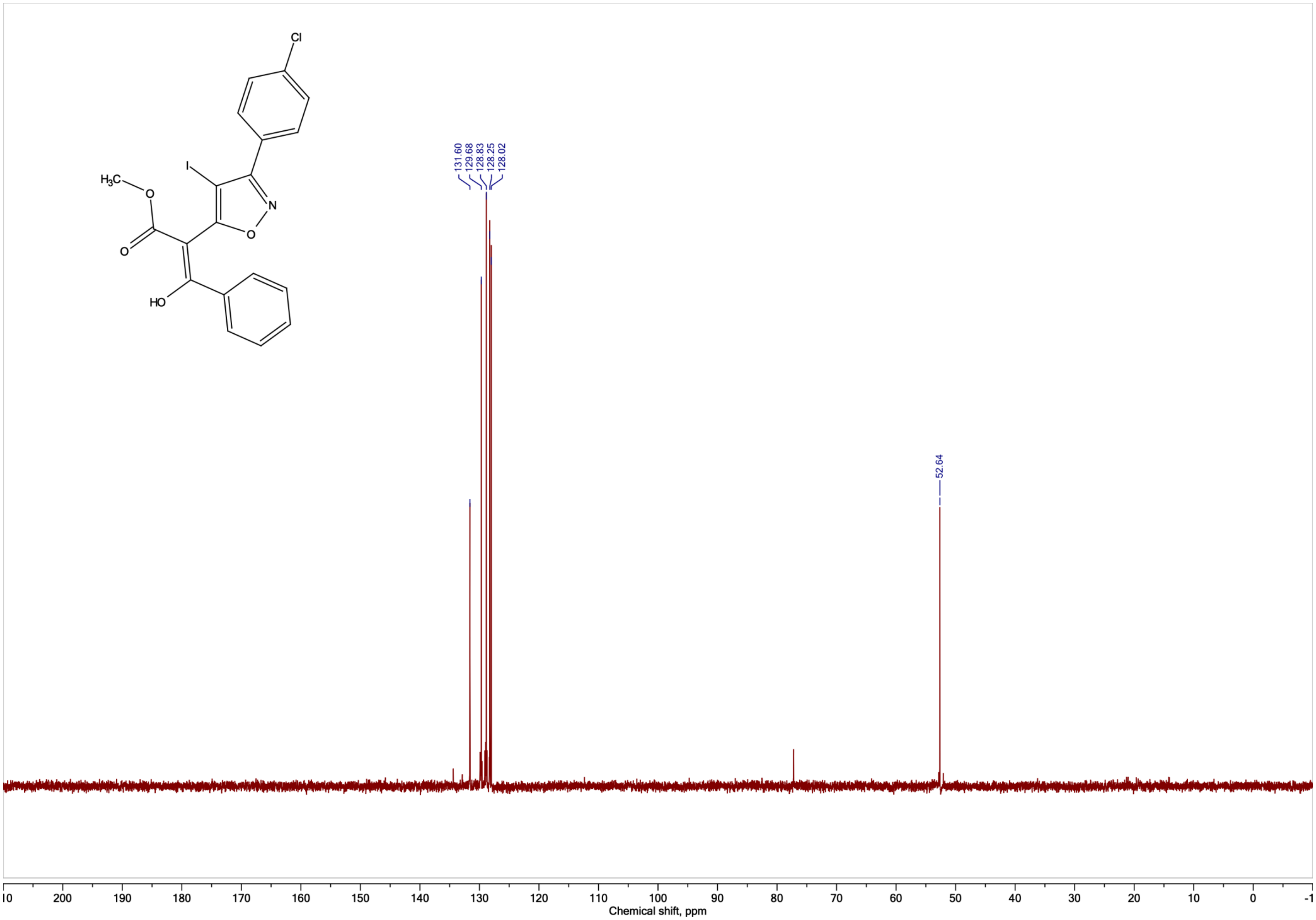

Methyl 3-oxo-2-(3-phenyl-4-(thiophen-3-yl)isoxazol-5-yl)-3-(*p*-tolyl)propanoate (1n), ^{1}H NMR, $CDCl_3$, 400 MHz

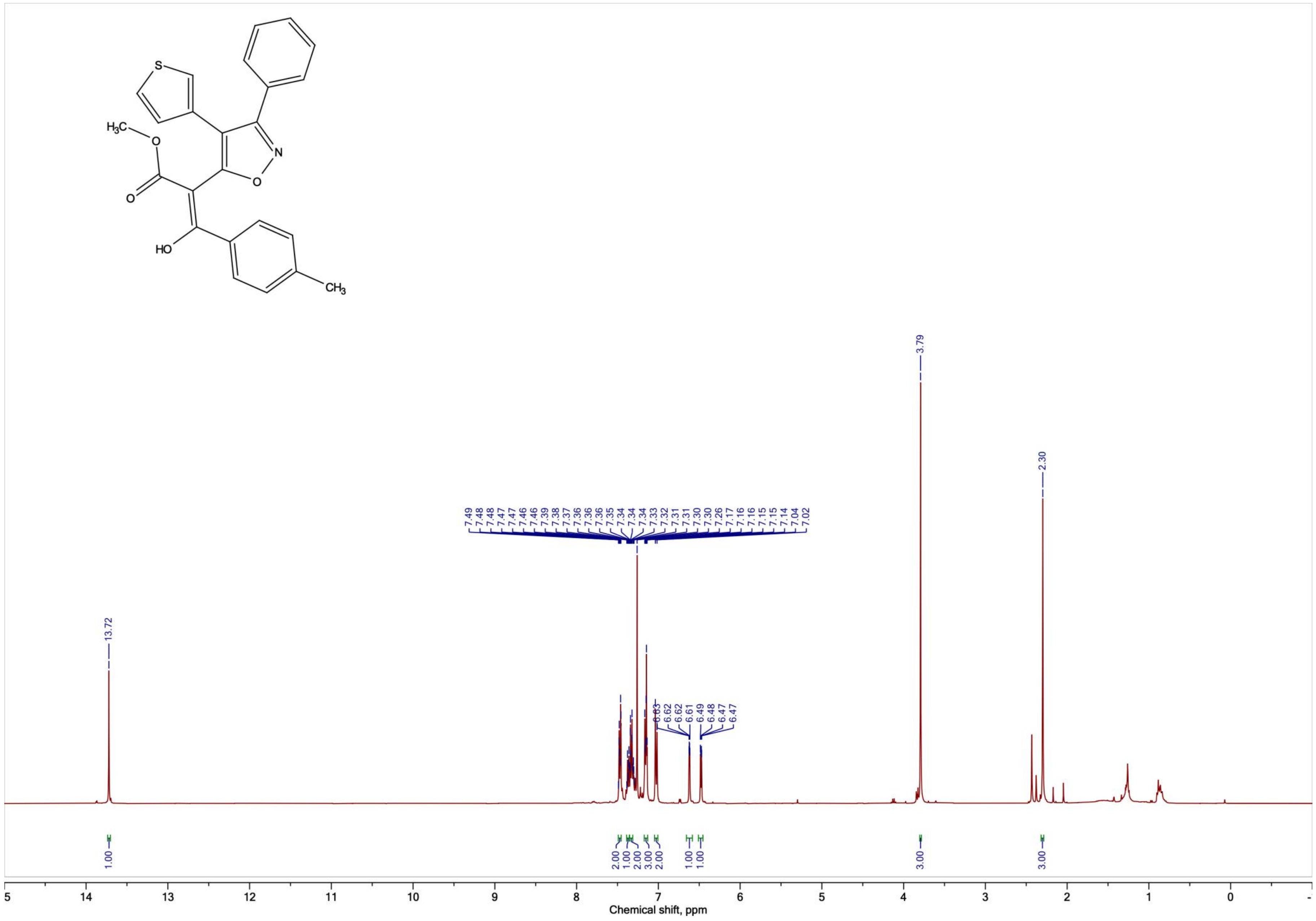

Methyl 3-oxo-2-(3-phenyl-4-(thiophen-3-yl)isoxazol-5-yl)-3-(*p*-tolyl)propanoate (1n), $^{13}C\{^{1}H\}$ NMR, $CDCl_3$, 101 MHz

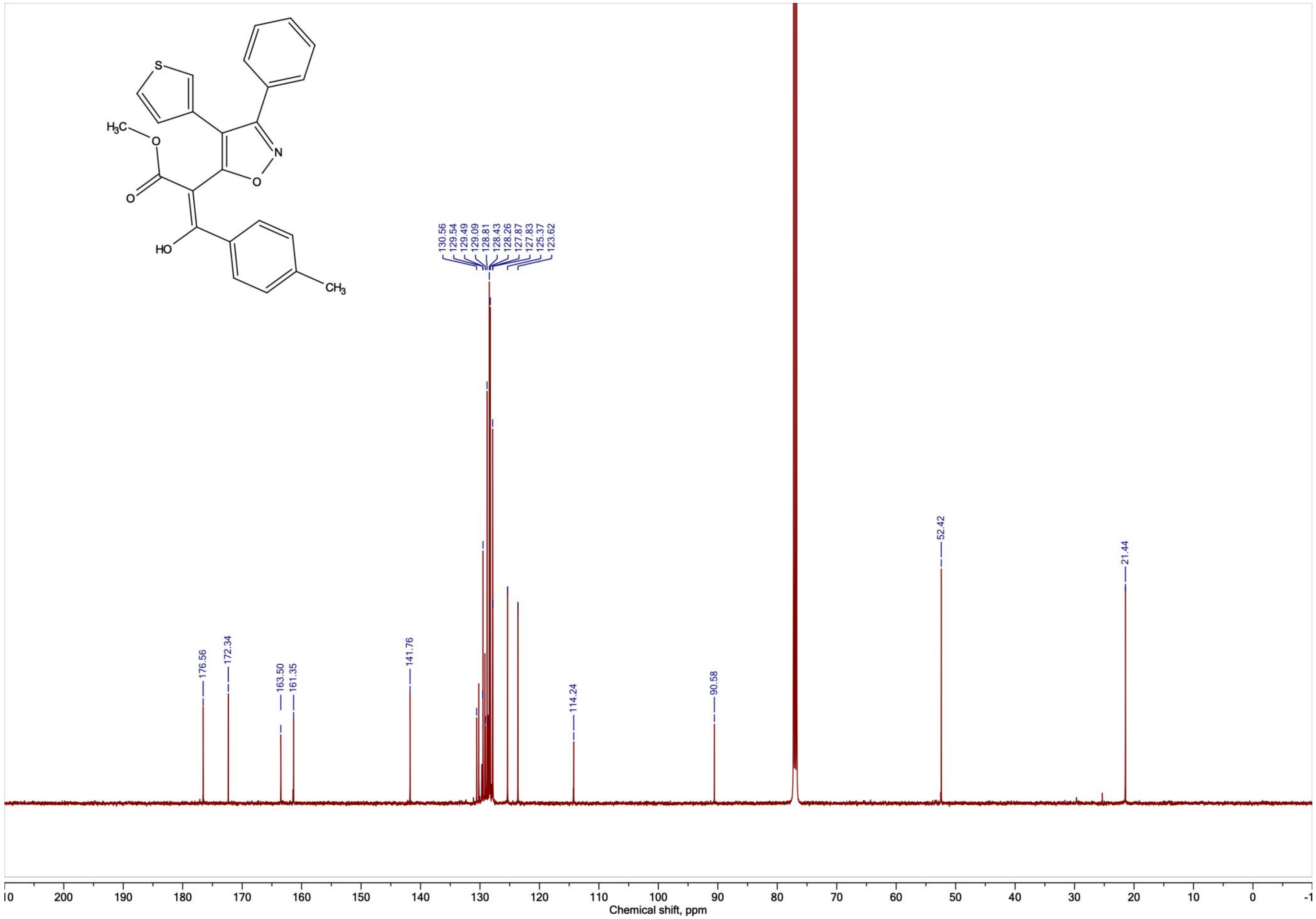

Methyl 3-oxo-2-(3-phenyl-4-(thiophen-3-yl)isoxazol-5-yl)-3-(*p*-tolyl)propanoate (1n), DEPT, $CDCl_3$, 101 MHz

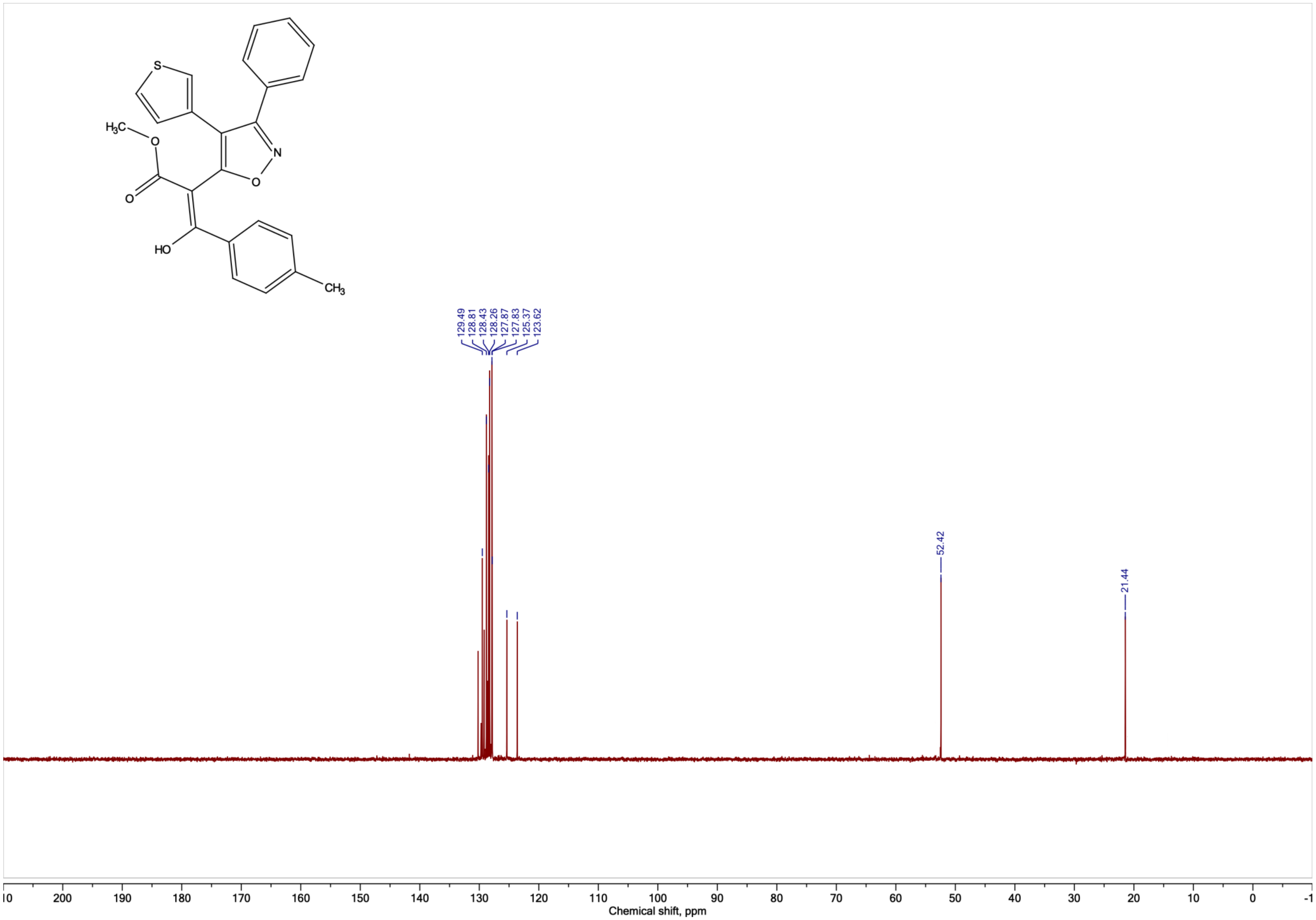

Methyl 2-(3-(4-(*tert*-butyl)phenyl)-4-(4-(trifluoromethyl)phenyl)isoxazol-5-yl)-3-oxo-3-(*p*-tolyl)propanoate (1o), ^{1}H NMR, $CDCl_3$, 400 MHz

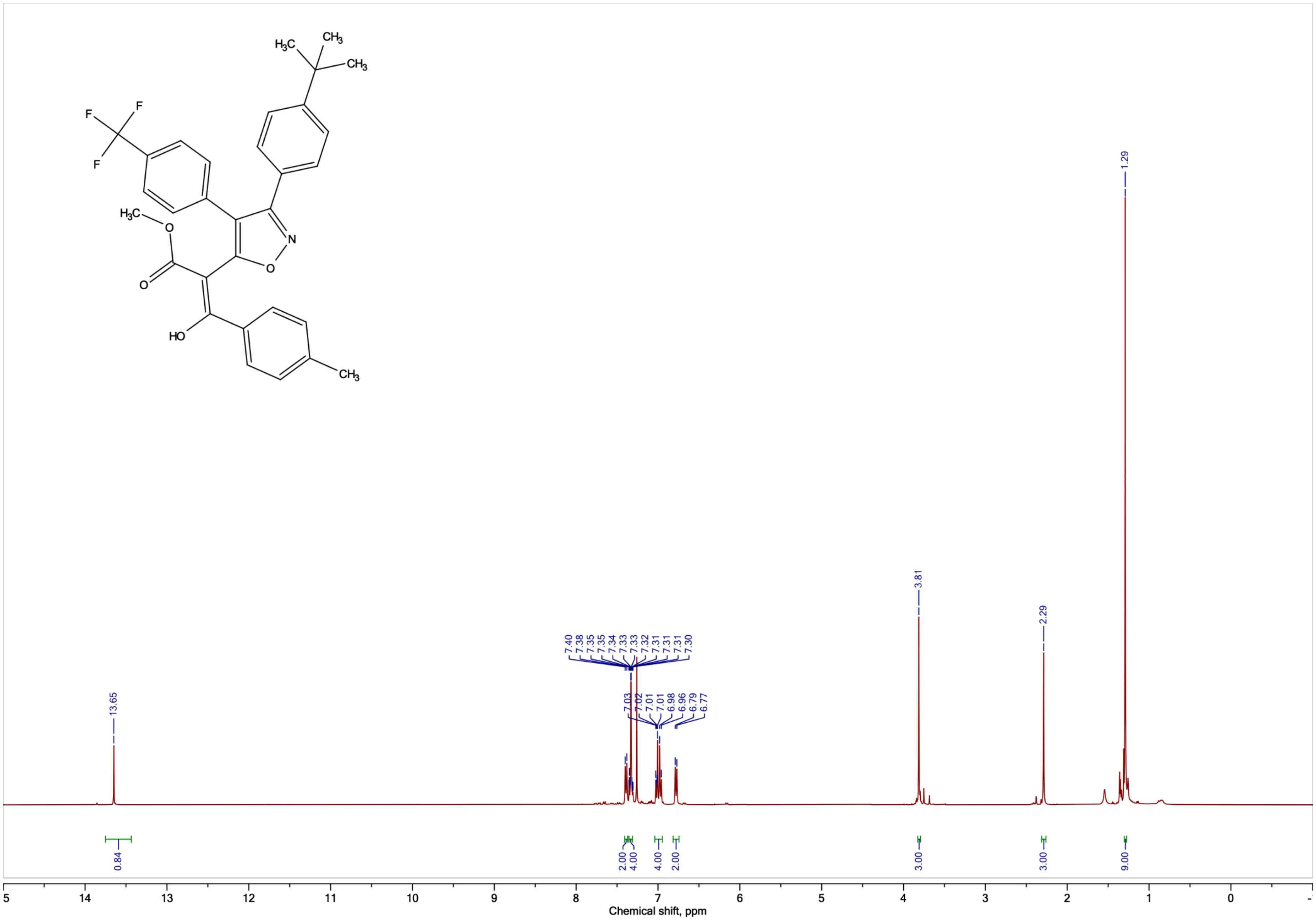

Methyl 2-(3-(4-(*tert*-butyl)phenyl)-4-(4-(trifluoromethyl)phenyl)isoxazol-5-yl)-3-oxo-3-(*p*-tolyl)propanoate (1o), $^{13}C\{^{1}H\}$ NMR, $CDCl_3$, 101 MHz

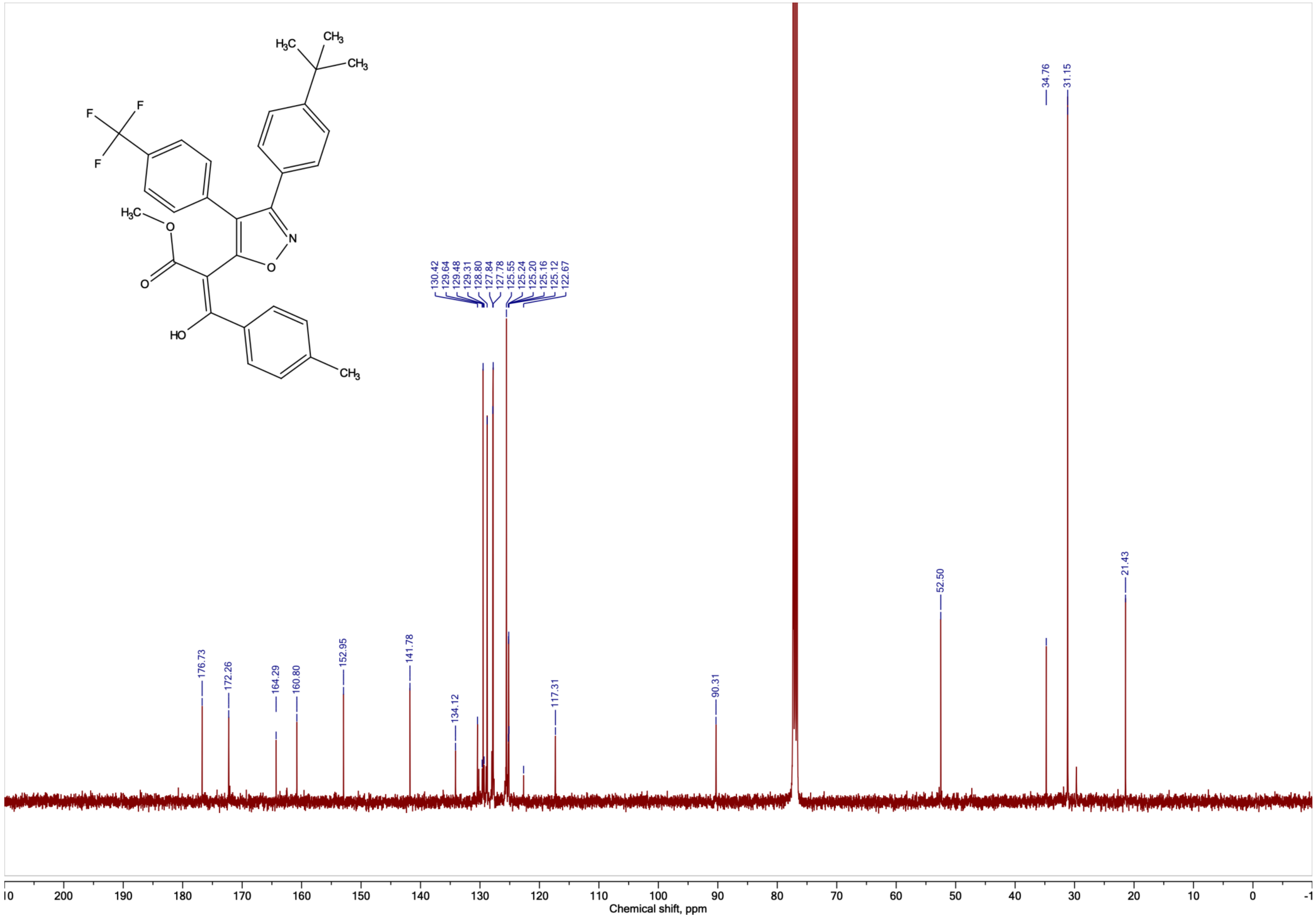

Methyl 2-(3-(4-(*tert*-butyl)phenyl)-4-(4-(trifluoromethyl)phenyl)isoxazol-5-yl)-3-oxo-3-(*p*-tolyl)propanoate (1o), DEPT, $CDCl_3$, 101 MHz

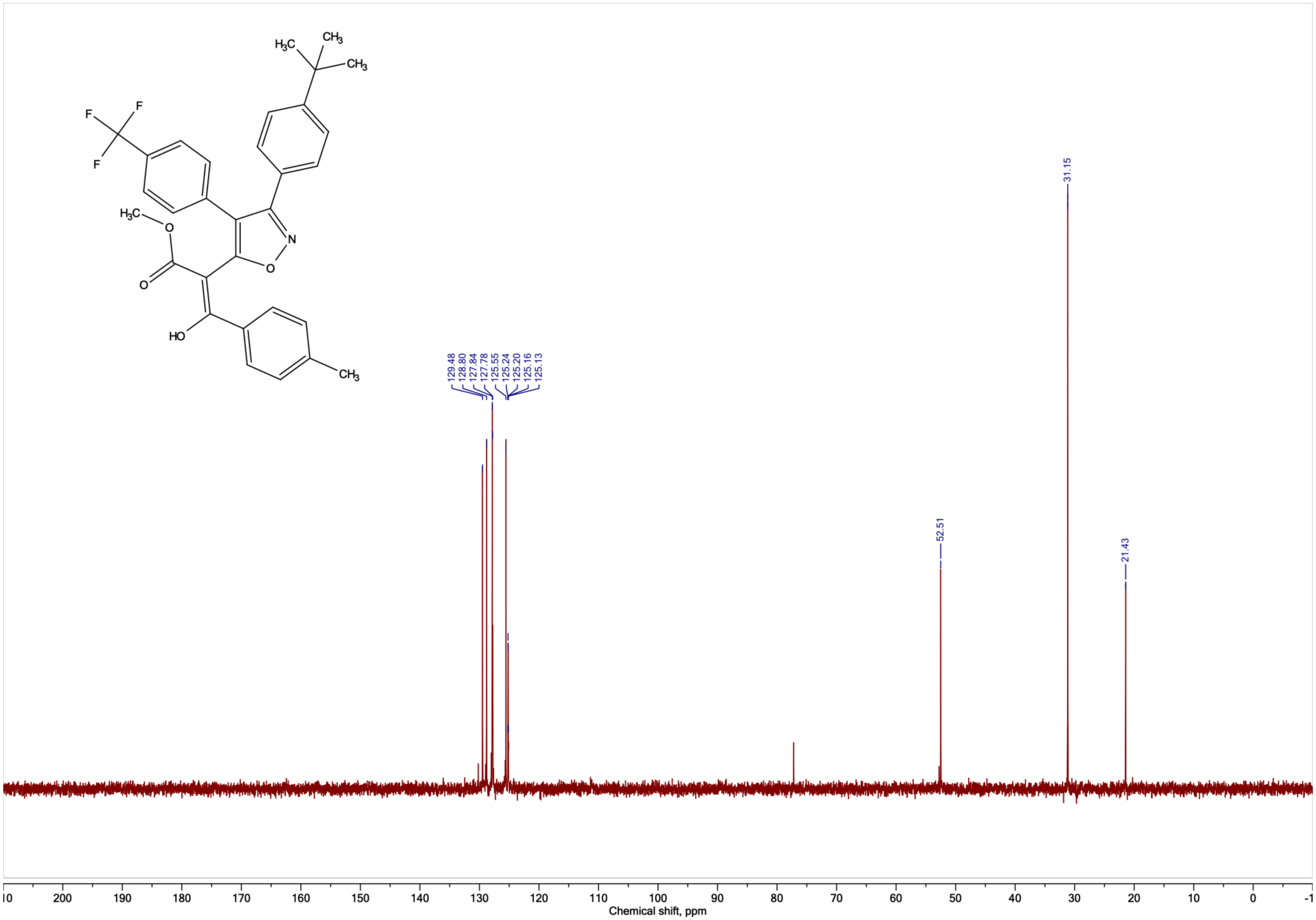

Methyl 2-(3-(4-chlorophenyl)-4-phenylisoxazol-5-yl)-3-oxo-3-(*p*-tolyl)propanoate (1p), ^{1}H NMR, $CDCl_3$, 400 MHz

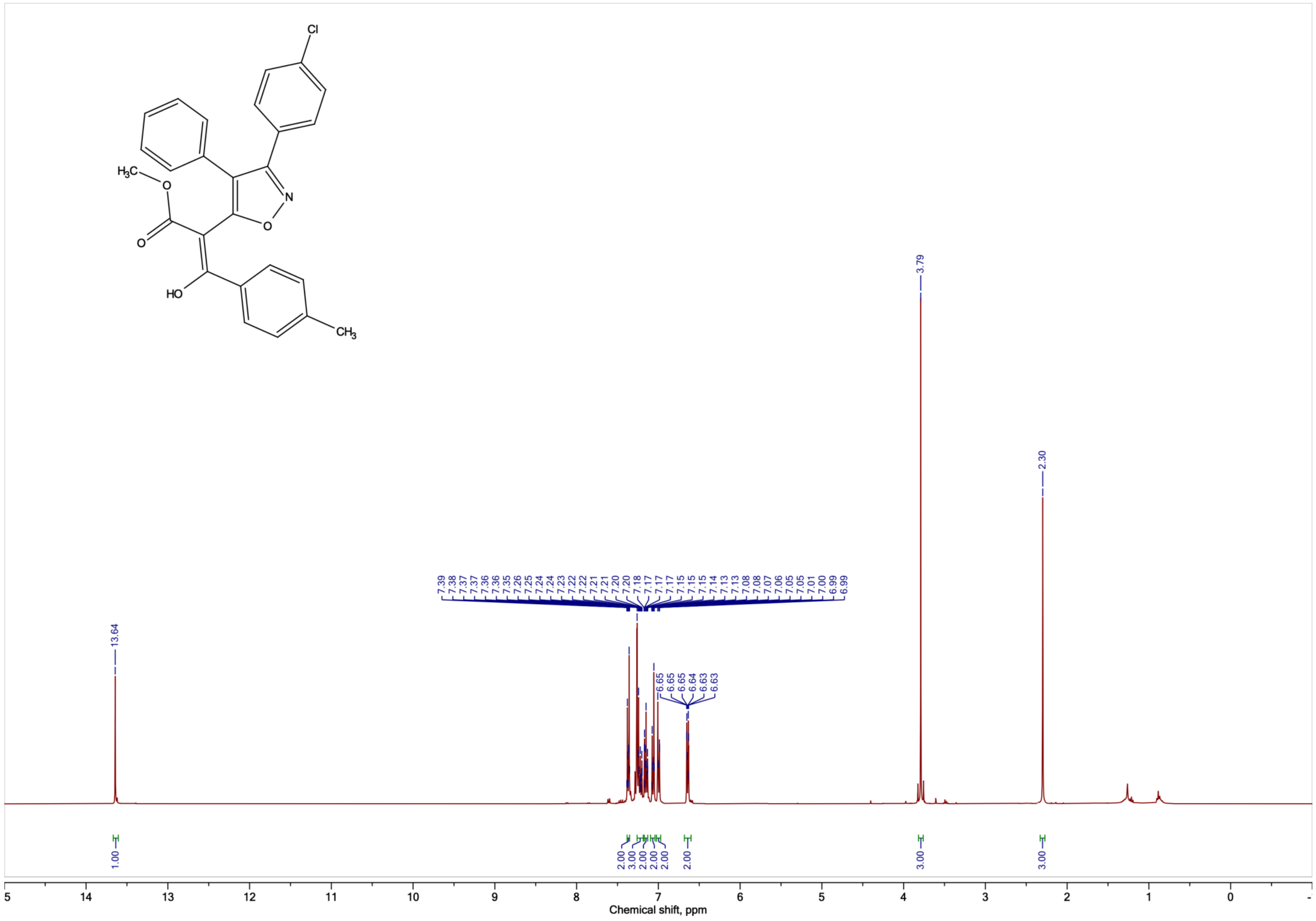

Methyl 2-(3-(4-chlorophenyl)-4-phenylisoxazol-5-yl)-3-oxo-3-(*p*-tolyl)propanoate (1p), $^{13}C\{^{1}H\}$ NMR, $CDCl_3$, 101 MHz

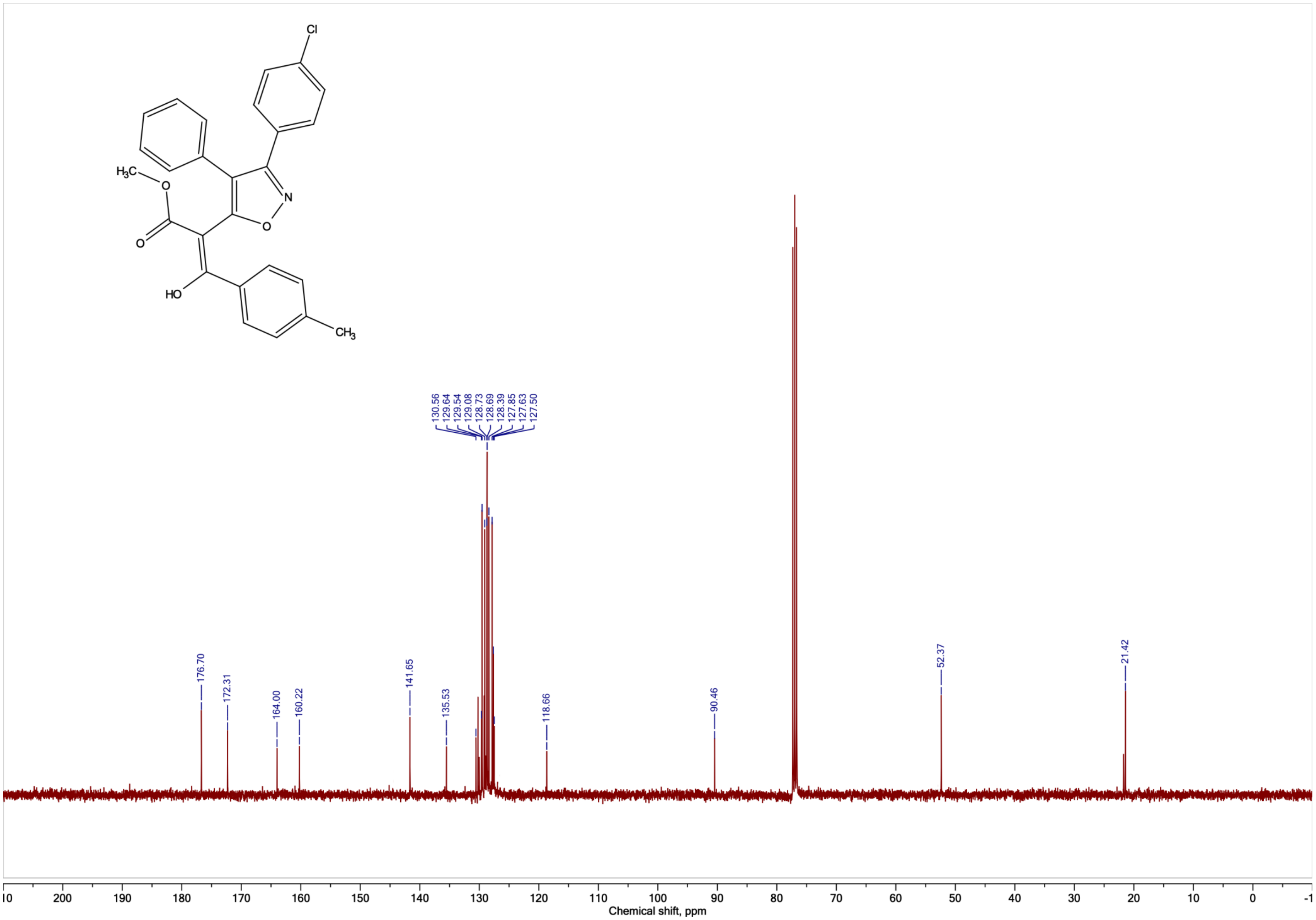

Methyl 2-(3-(4-chlorophenyl)-4-phenylisoxazol-5-yl)-3-oxo-3-(*p*-tolyl)propanoate (1p), DEPT, $CDCl_3$, 101 MHz

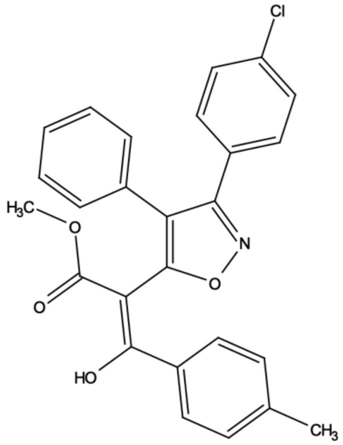

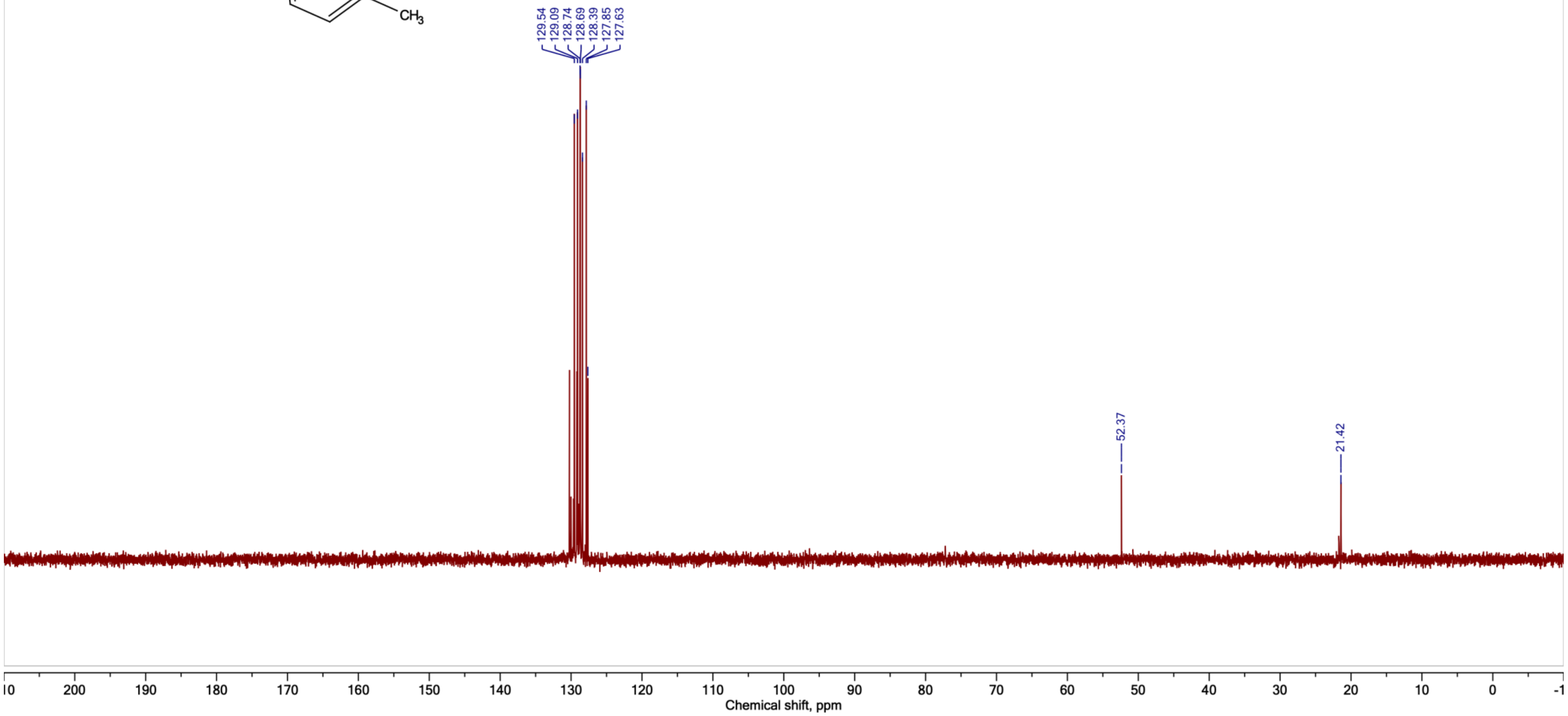

Methyl 2-(3-(4-chlorophenyl)-4-(4-methoxyphenyl)isoxazol-5-yl)-3-oxo-3-(*p*-tolyl)propanoate (1q), ^{1}H NMR, $CDCl_3$, 400 MHz

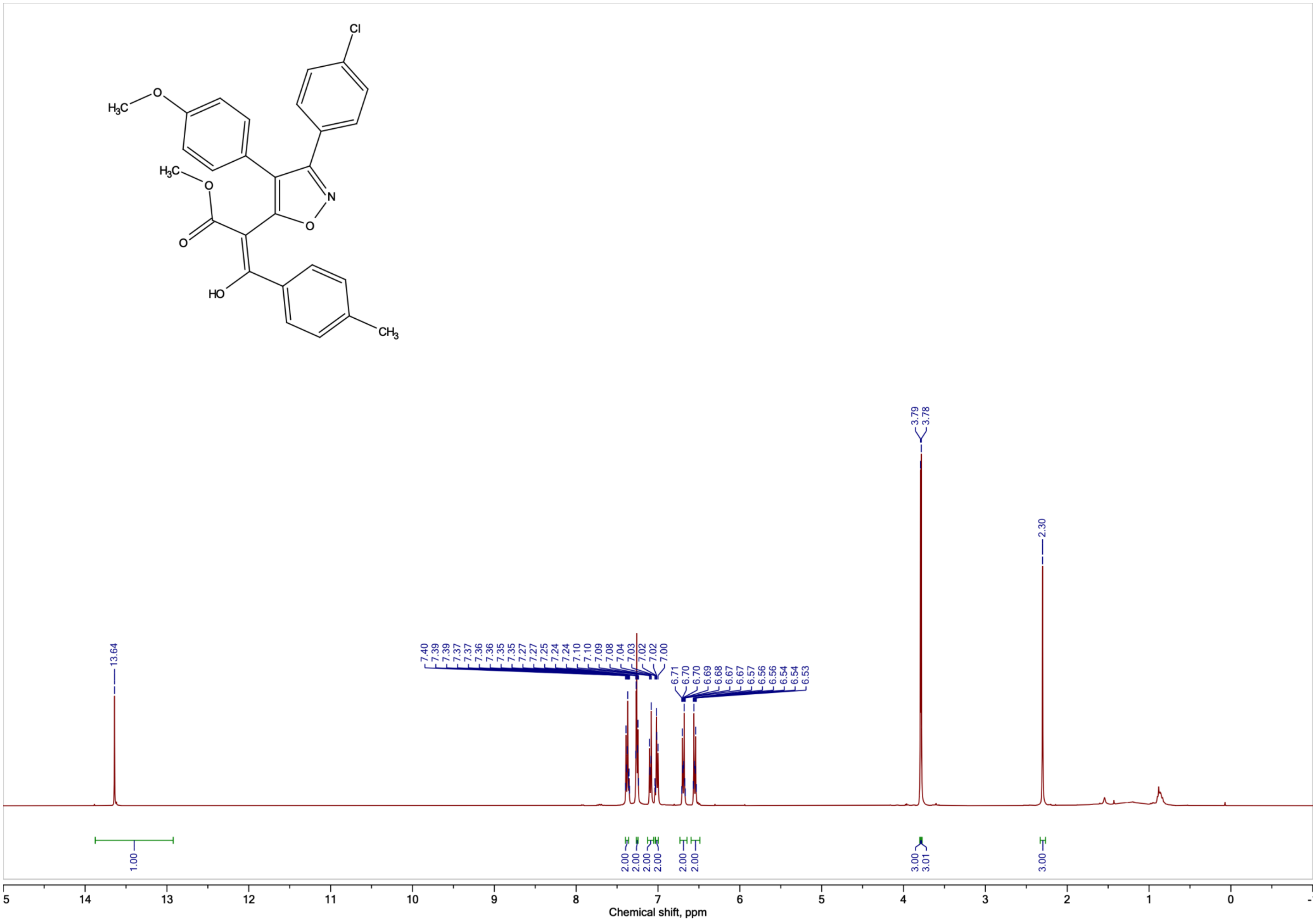

Methyl 2-(3-(4-chlorophenyl)-4-(4-methoxyphenyl)isoxazol-5-yl)-3-oxo-3-(*p*-tolyl)propanoate (1q), $^{13}C\{^{1}H\}$ NMR, $CDCl_3$, 101 MHz

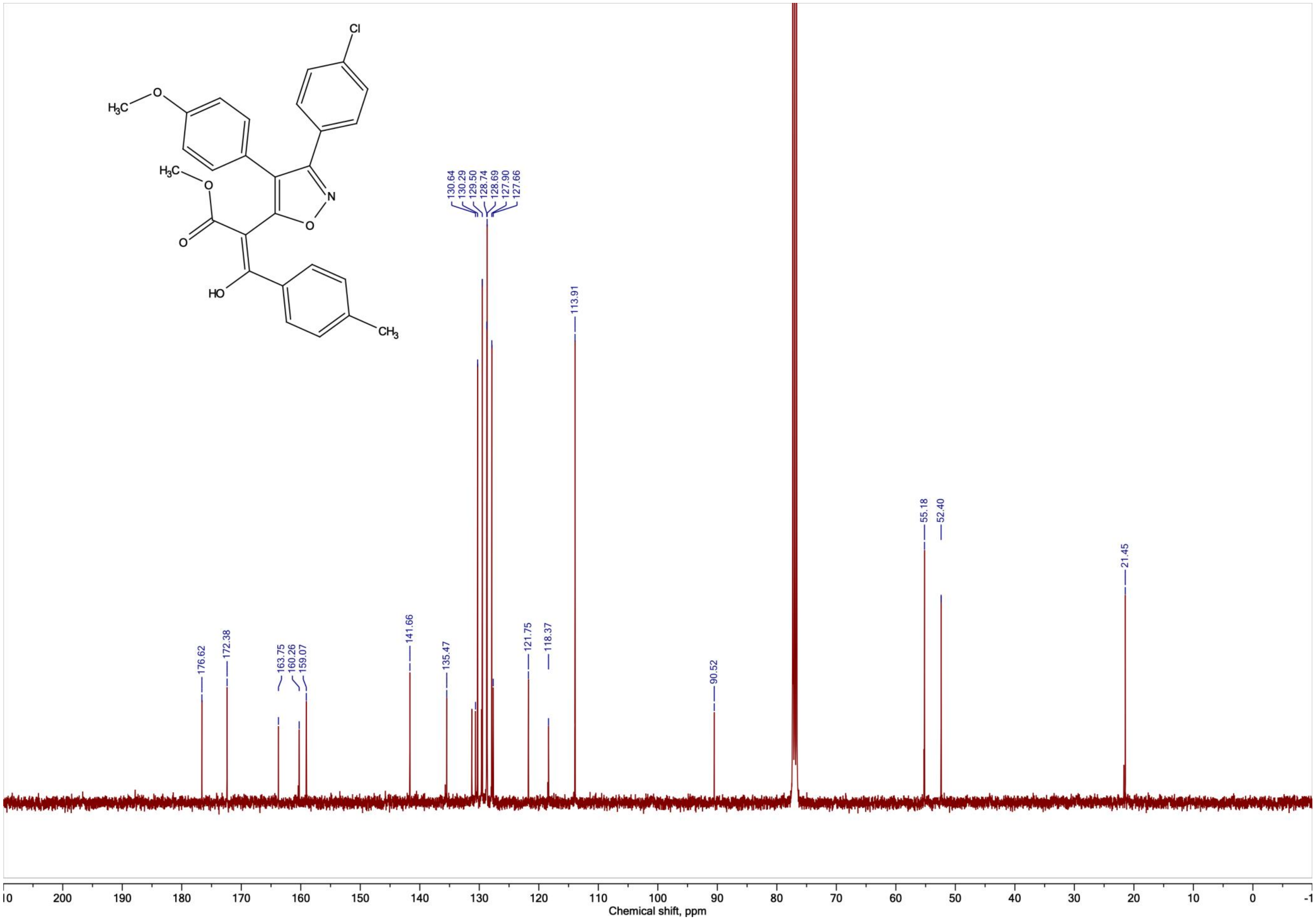

Methyl 2-(3-(4-chlorophenyl)-4-(4-methoxyphenyl)isoxazol-5-yl)-3-oxo-3-(*p*-tolyl)propanoate (1q), DEPT, $CDCl_3$, 101 MHz

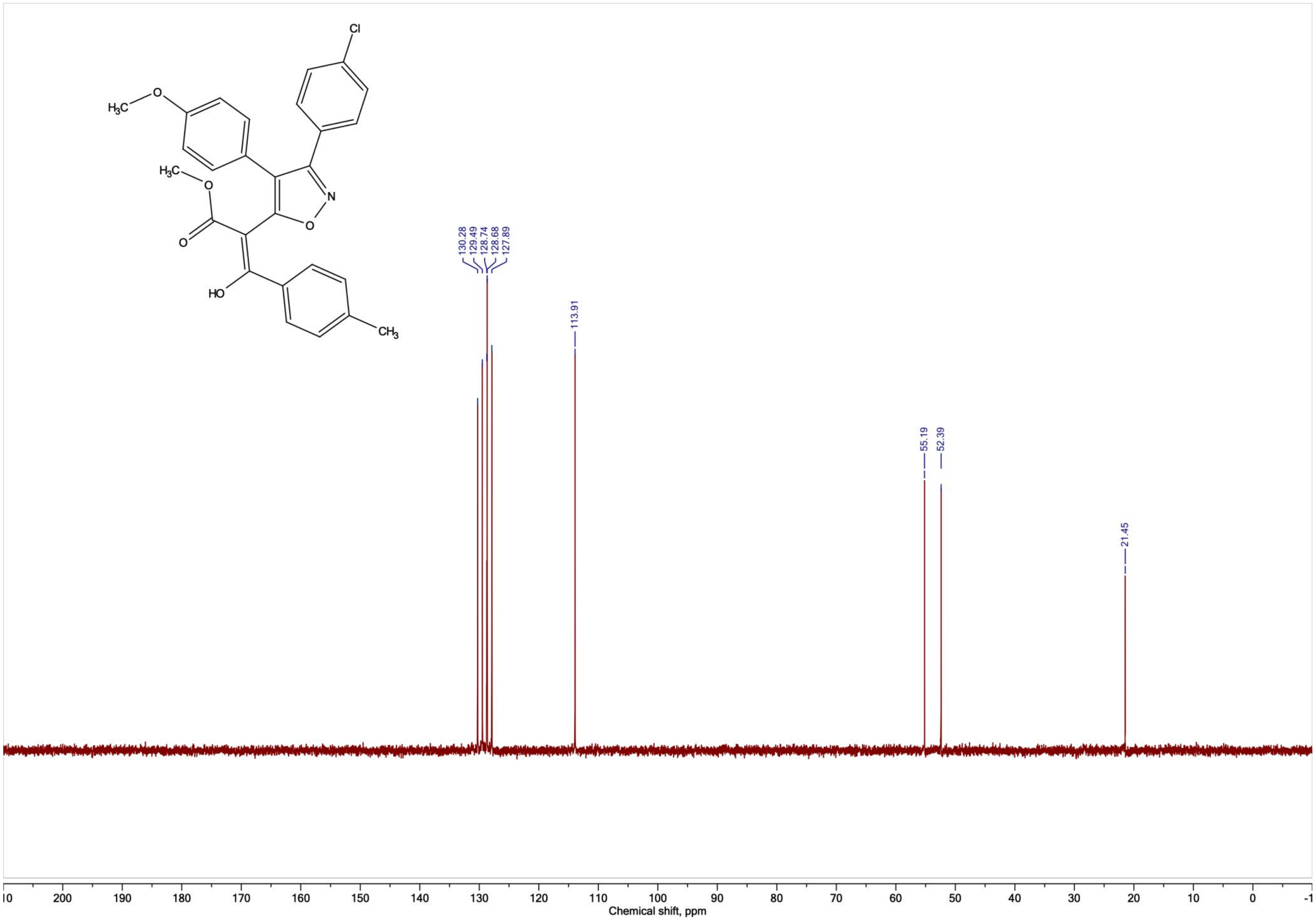

Methyl 3-(4-bromophenyl)-2-(3-(4-bromophenyl)-4-(4-fluorophenyl)isoxazol-5-yl)-3-oxopropanoate (1r), ^{1}H NMR, $CDCl_3$, 400 MHz

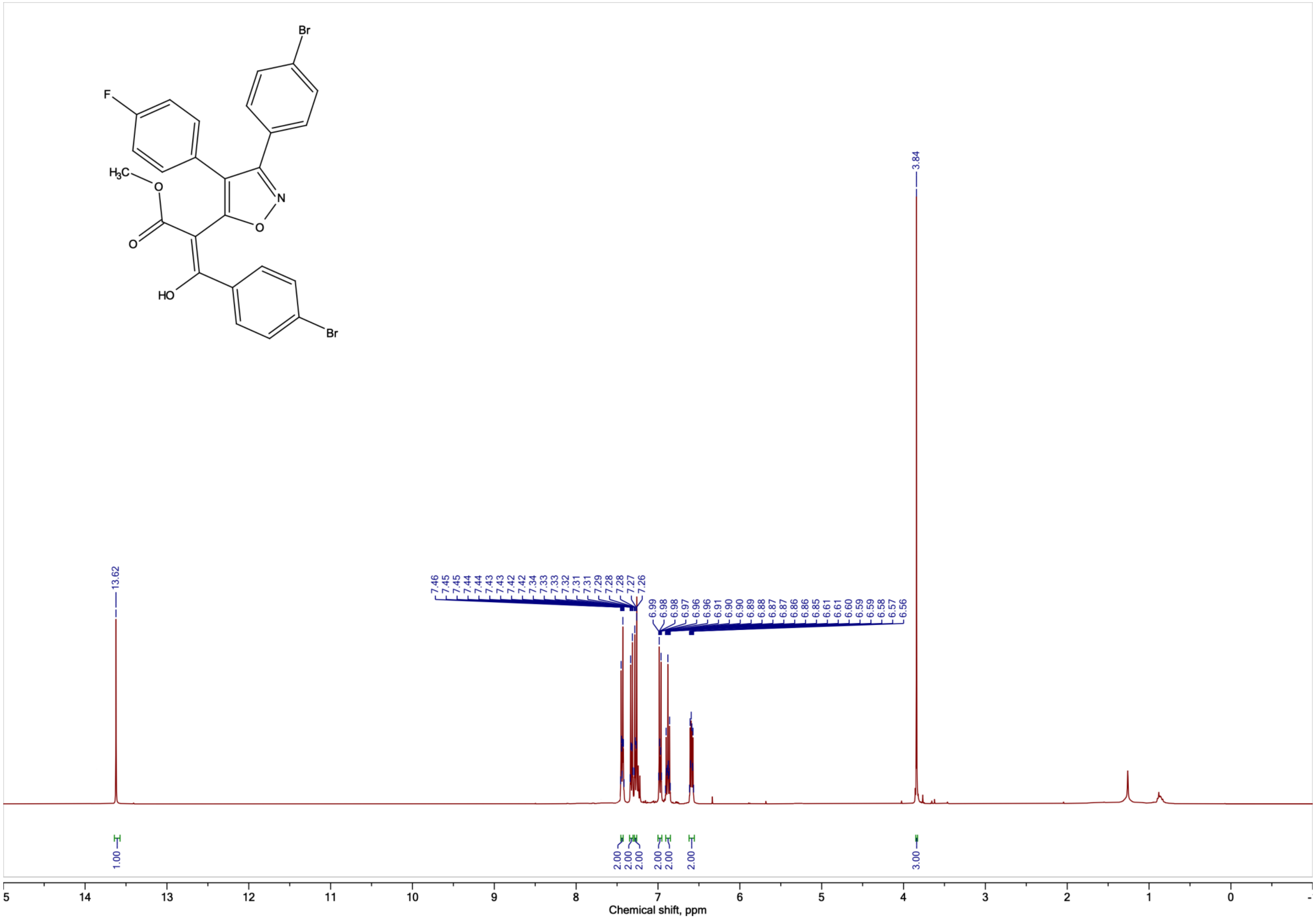

Methyl 3-(4-bromophenyl)-2-(3-(4-bromophenyl)-4-(4-fluorophenyl)isoxazol-5-yl)-3-oxopropanoate (1r), $^{13}C\{^{1}H\}$ NMR, $CDCl_3$, 101 MHz

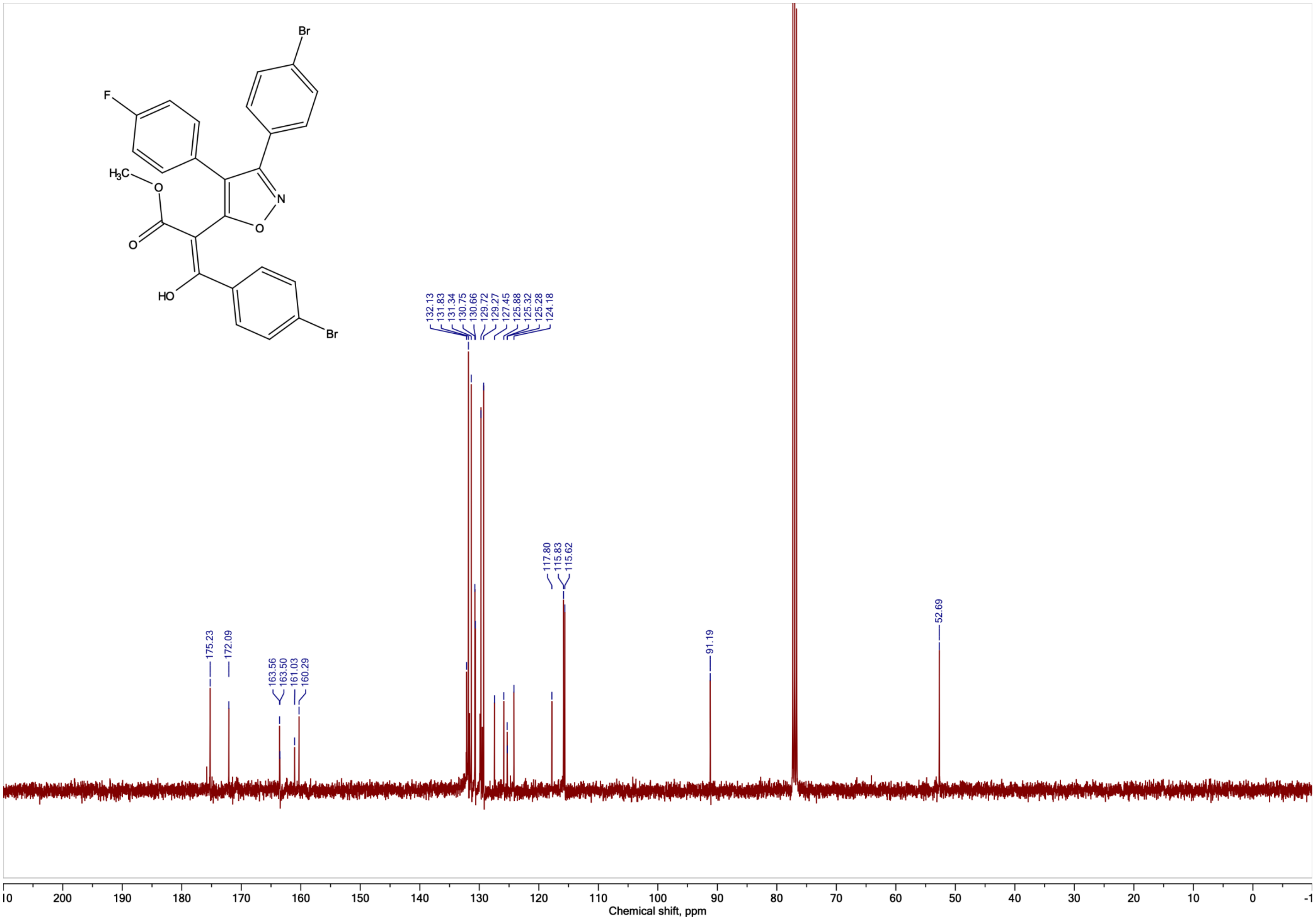

Methyl 3-(4-bromophenyl)-2-(3-(4-bromophenyl)-4-(4-fluorophenyl)isoxazol-5-yl)-3-oxopropanoate (1r), DEPT, $CDCl_3$, 101 MHz

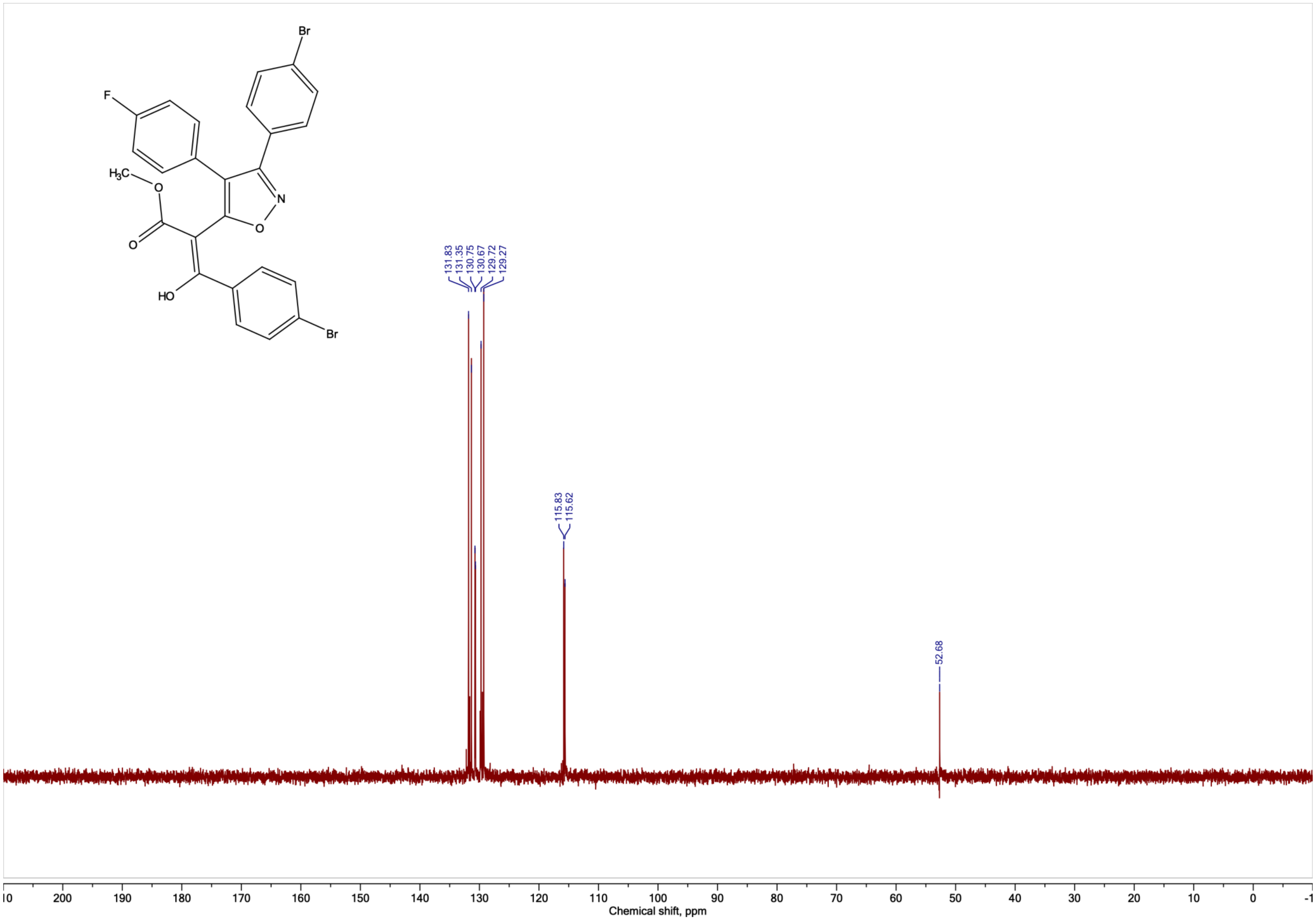

Methyl 3-(4-bromophenyl)-2-(3-(4'-fluoro-[1,1'-biphenyl]-4-yl)-4-(4-fluorophenyl)isoxazol-5-yl)-3-oxopropanoate (13f), ^{1}H NMR, $CDCl_3$, 400 MHz

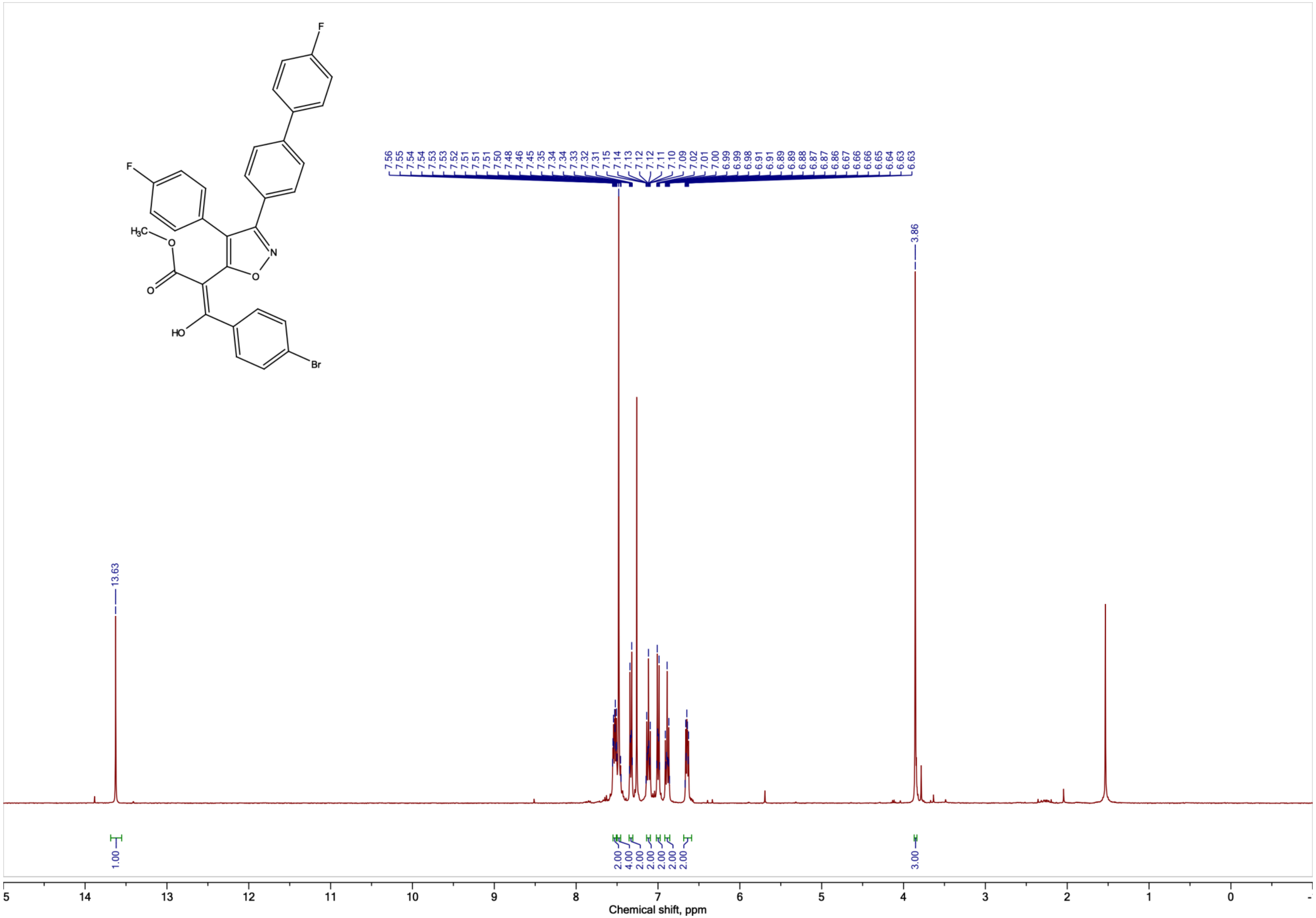

Methyl 3-(4-bromophenyl)-2-(3-(4'-fluoro-[1,1'-biphenyl]-4-yl)-4-(4-fluorophenyl)isoxazol-5-yl)-3-oxopropanoate (13f), $^{13}C\{^1H\}$ NMR, $CDCl_3$, 101 MHz

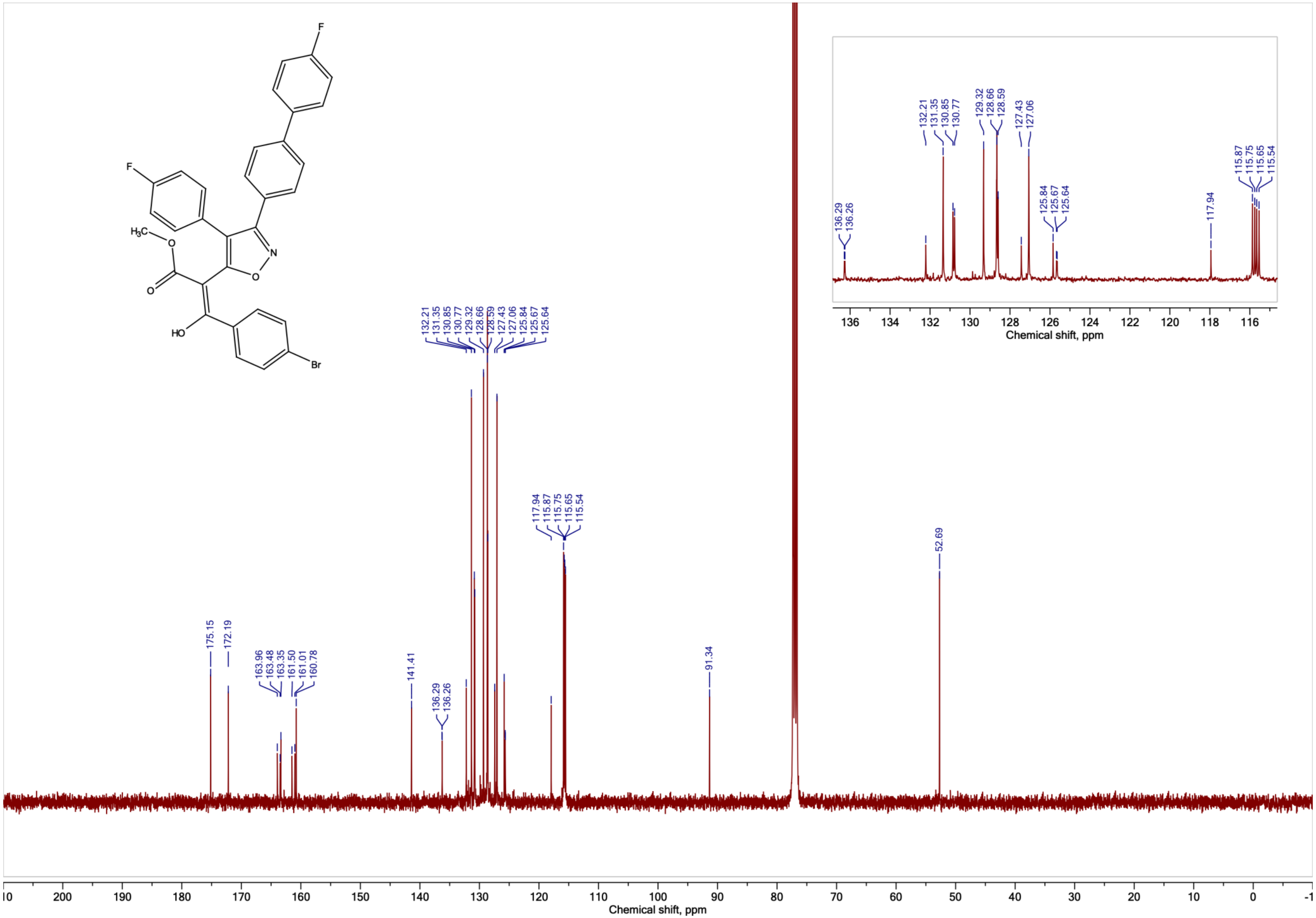

Methyl 3-(4-bromophenyl)-2-(3-(4'-fluoro-[1,1'-biphenyl]-4-yl)-4-(4-fluorophenyl)isoxazol-5-yl)-3-oxopropanoate (13f), DEPT, $CDCl_3$, 101 MHz

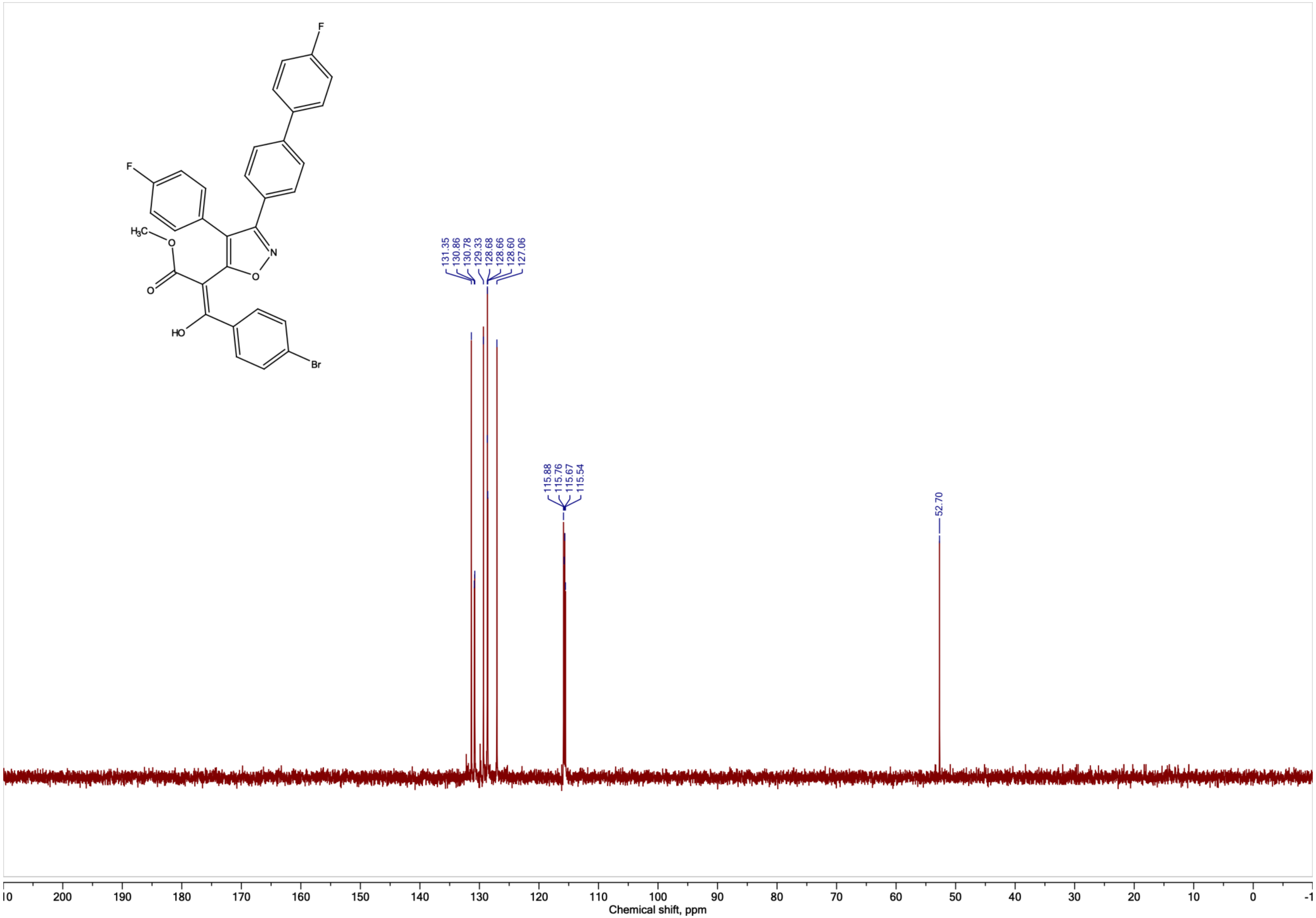

Methyl 2-ethyl-4-oxo-6-phenyl-1,4-dihydropyridine-3-carboxylate (2a), ^{1}H NMR, $CDCl_3$, 400 MHz

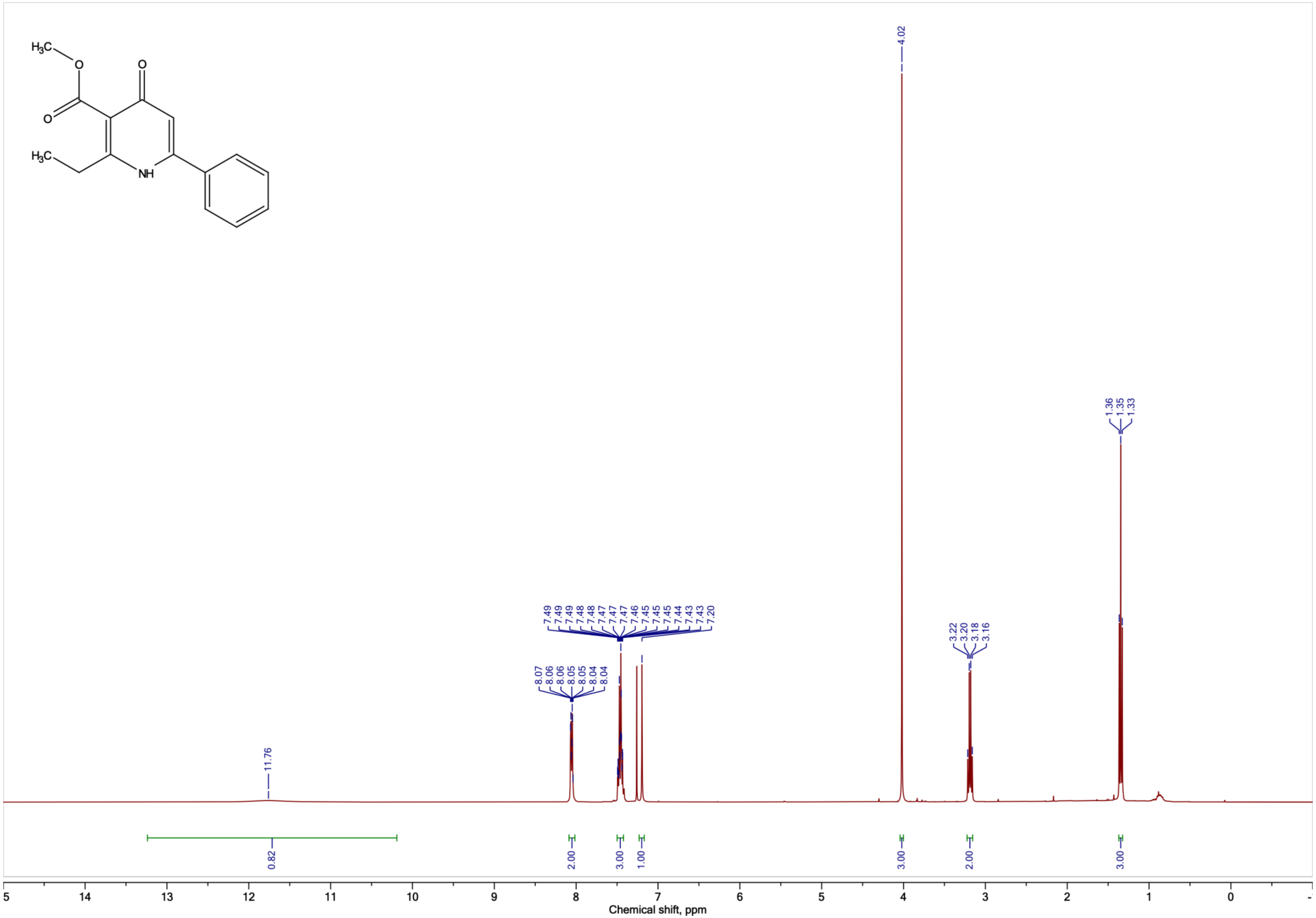

Methyl 2-ethyl-4-oxo-6-phenyl-1,4-dihydropyridine-3-carboxylate (2a), $^{13}C\{^1H\}$ NMR, $CDCl_3$, 101 MHz

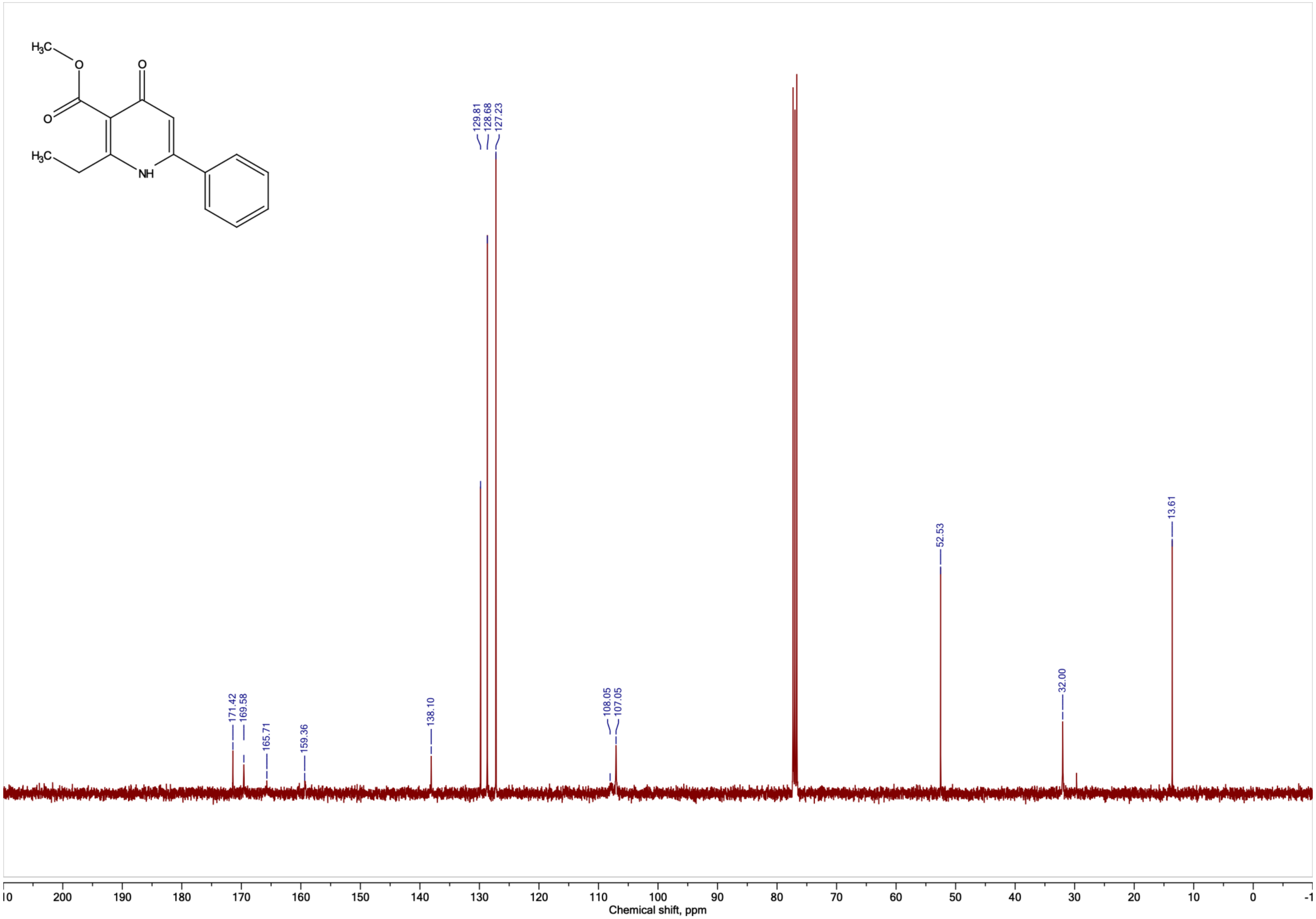

Methyl 2-ethyl-4-oxo-6-phenyl-1,4-dihydropyridine-3-carboxylate (2a), DEPT, $CDCl_3$, 101 MHz

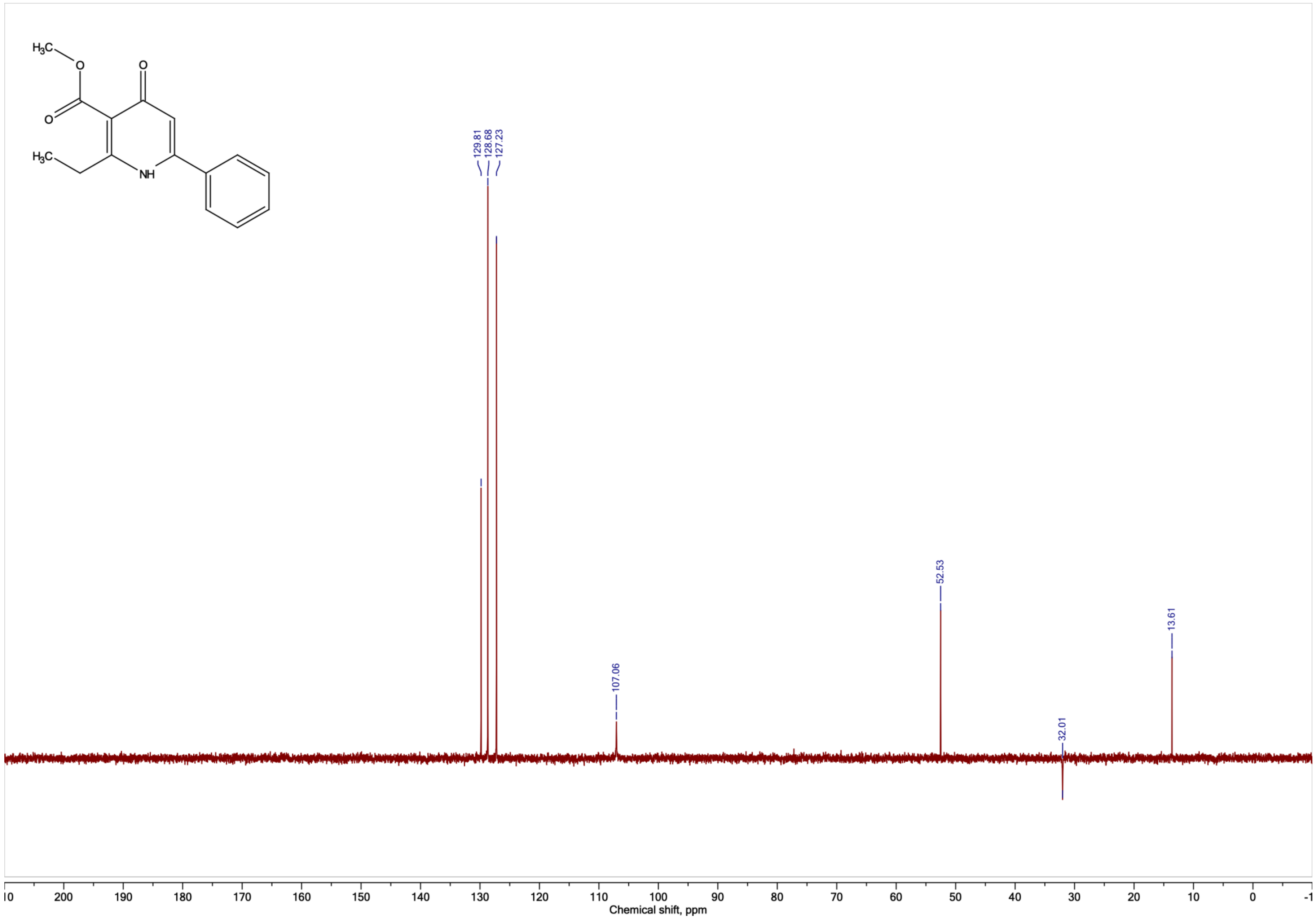

Methyl 2-(4-chlorobenzyl)-4-oxo-6-(*p*-tolyl)-1,4-dihydropyridine-3-carboxylate (2b), ^{1}H NMR, $CDCl_3$, 400 MHz

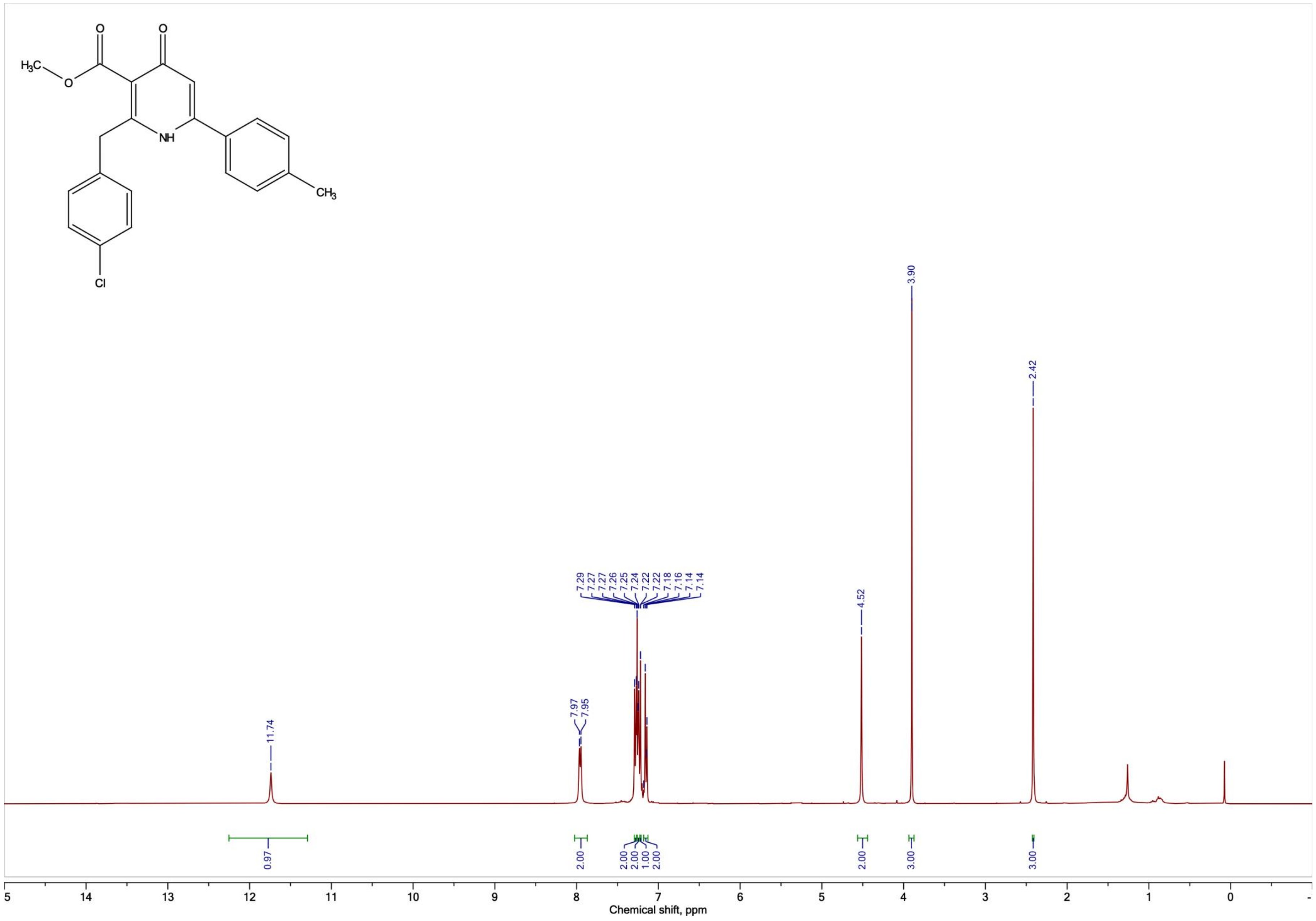

Methyl 2-(4-chlorobenzyl)-4-oxo-6-(*p*-tolyl)-1,4-dihydropyridine-3-carboxylate (2b), $^{13}C\{^1H\}$ NMR, $CDCl_3$, 101 MHz

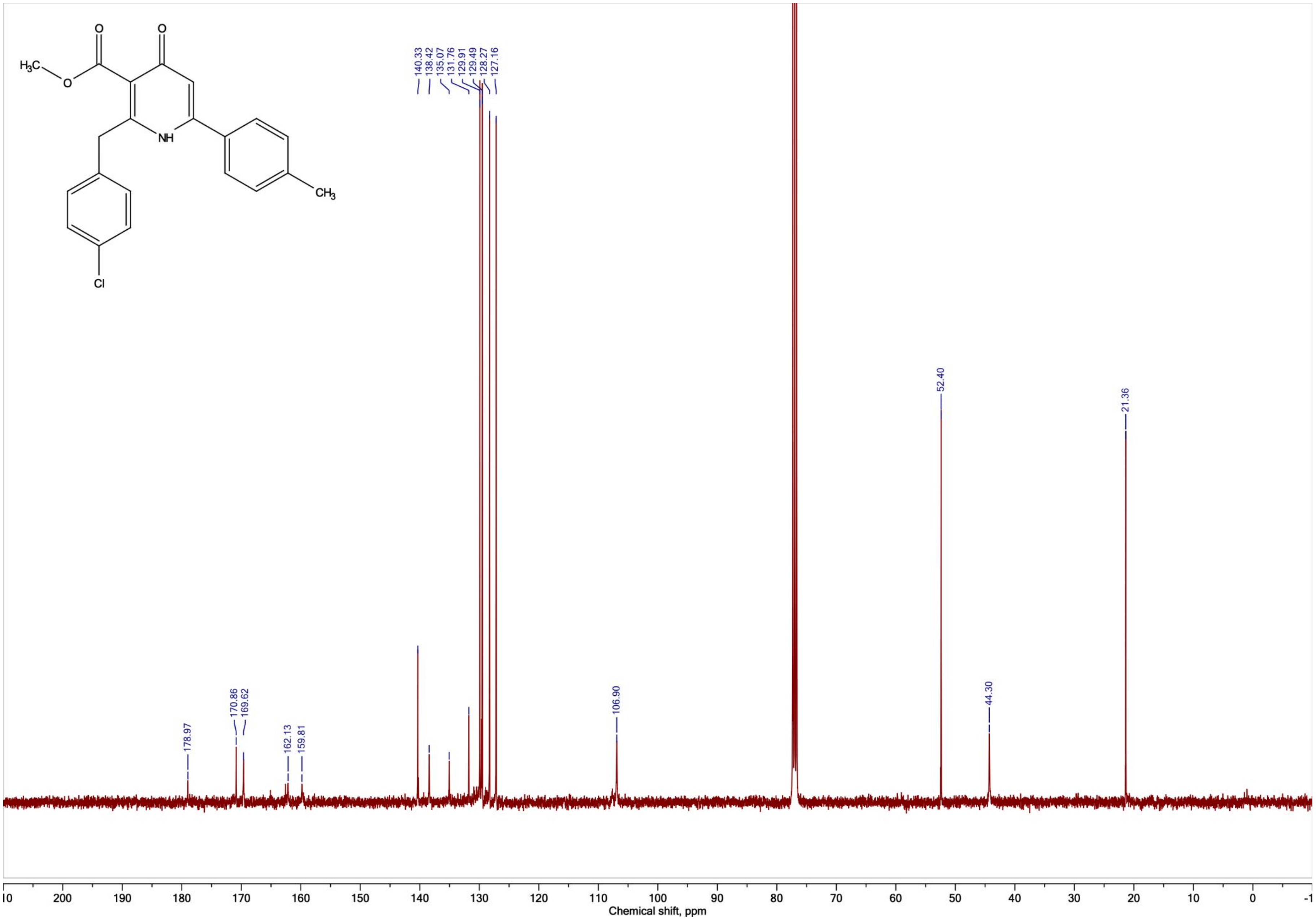

Methyl 2-(4-chlorobenzyl)-4-oxo-6-(*p*-tolyl)-1,4-dihydropyridine-3-carboxylate (2b), DEPT, $CDCl_3$, 101 MHz

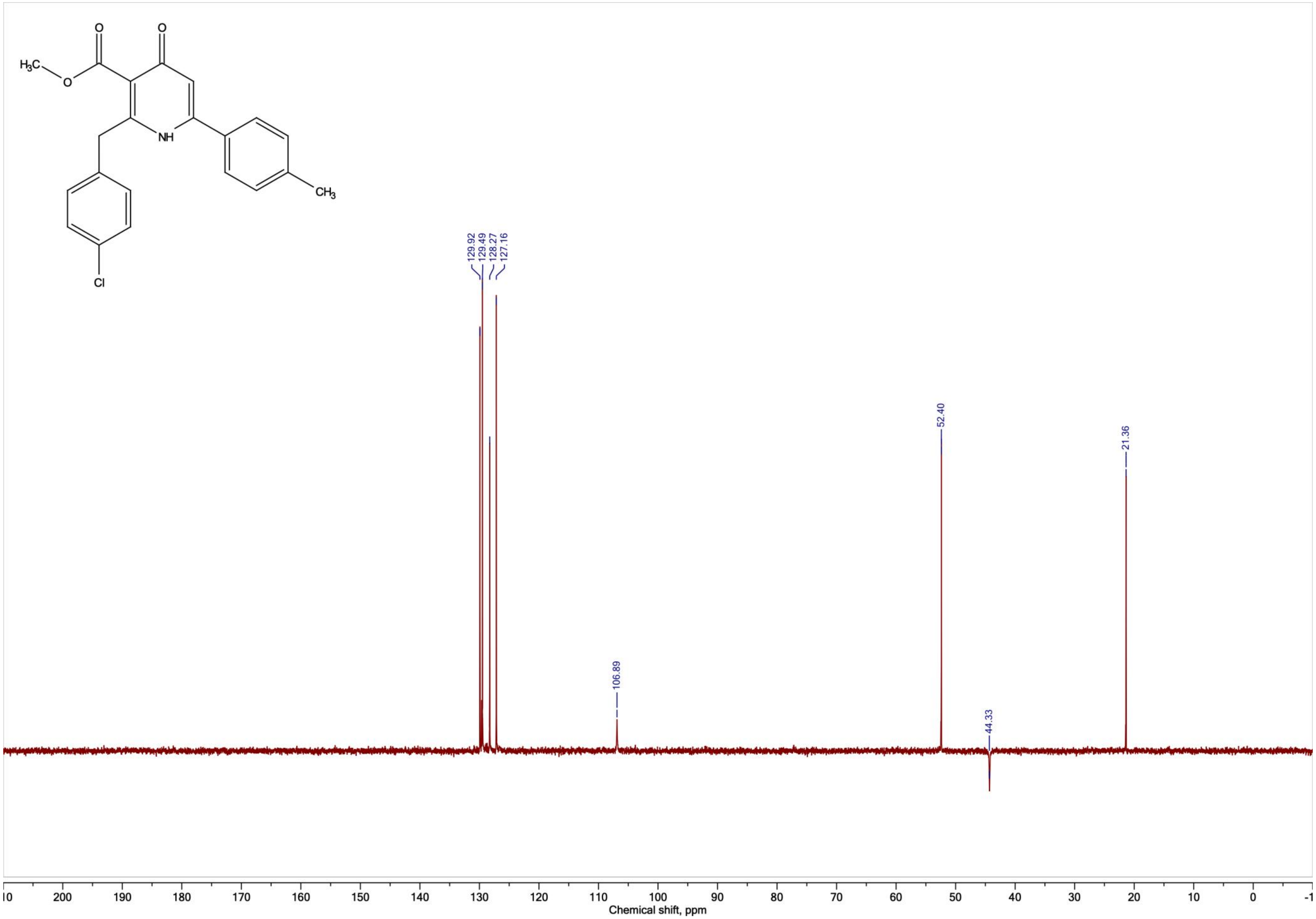

Methyl 2-(4-bromophenyl)-6-(4-(*tert*-butyl)phenyl)-4-oxo-1,4-dihydropyridine-3-carboxylate (2c), ^{1}H NMR, $CDCl_3$, 400 MHz

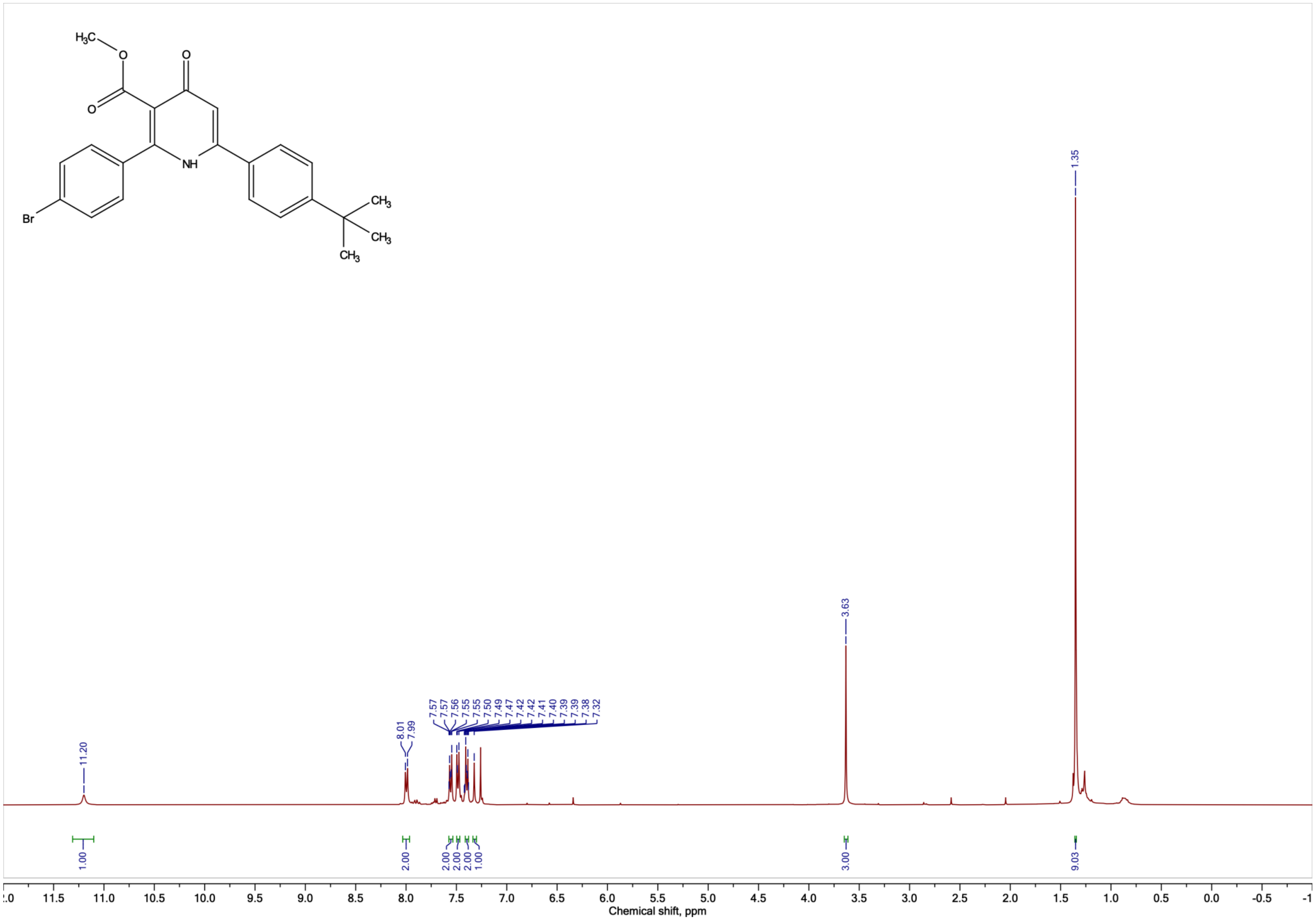

Methyl 2-(4-bromophenyl)-6-(4-(*tert*-butyl)phenyl)-4-oxo-1,4-dihydropyridine-3-carboxylate (2c), $^{13}C\{^{1}H\}$ NMR, $CDCl_3$, 101 MHz

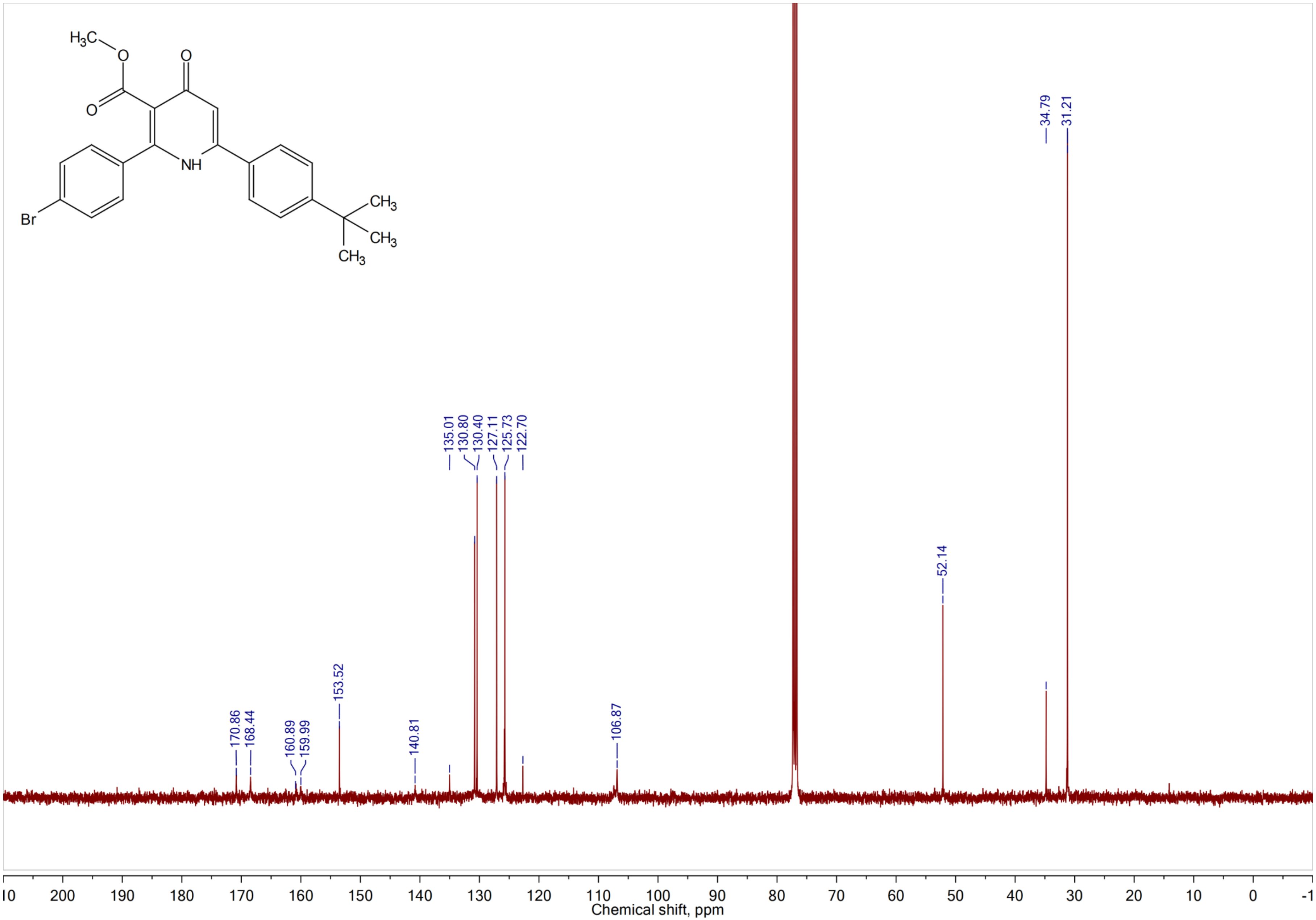

Methyl 2-(4-bromophenyl)-6-(4-(*tert*-butyl)phenyl)-4-oxo-1,4-dihydropyridine-3-carboxylate (2c), DEPT, $CDCl_3$, 101 MHz

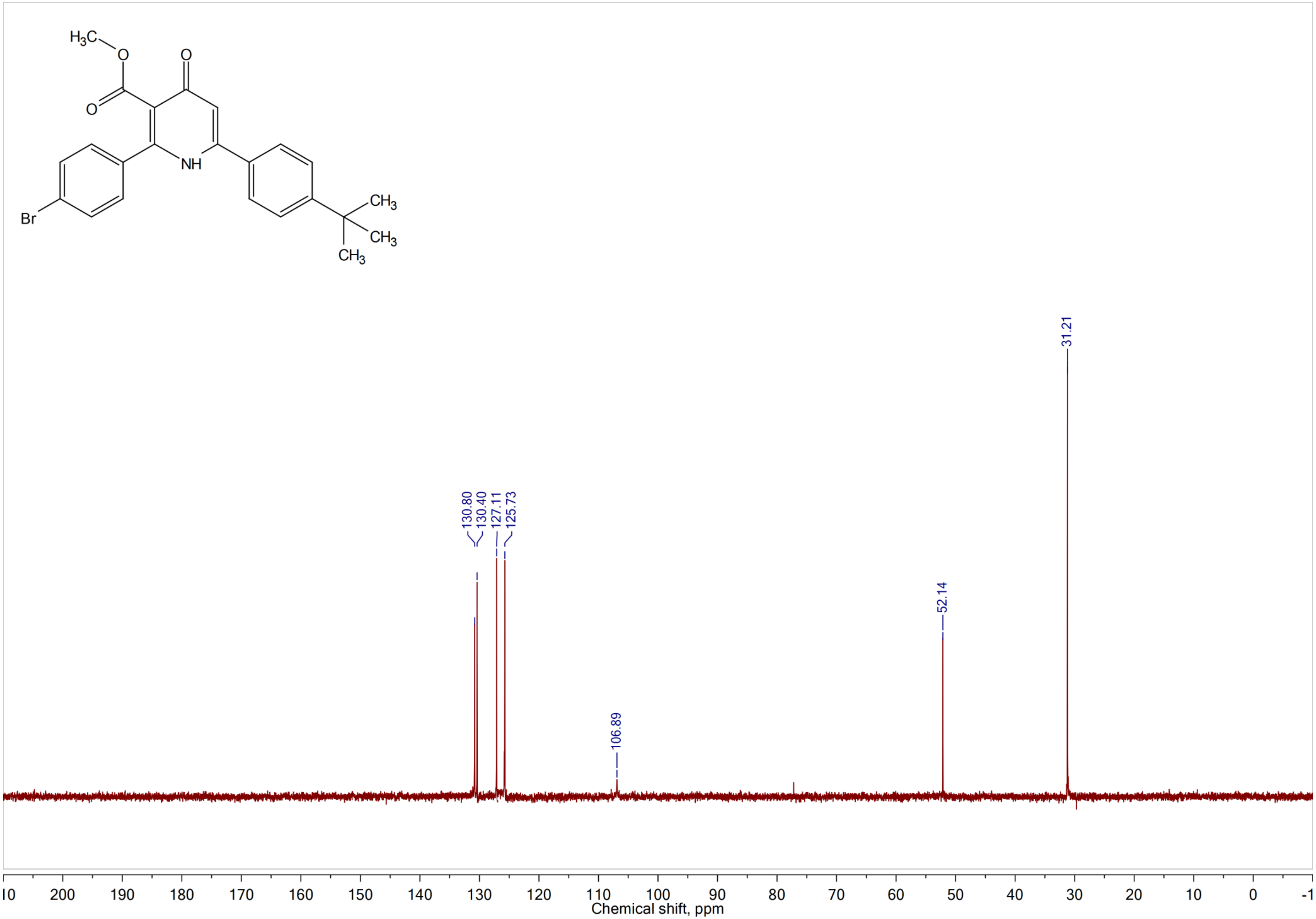

Methyl 2-(4-bromophenyl)-6-(4-methoxyphenyl)-4-oxo-1,4-dihydropyridine-3-carboxylate (2d), ^{1}H NMR, $CDCl_3$, 400 MHz

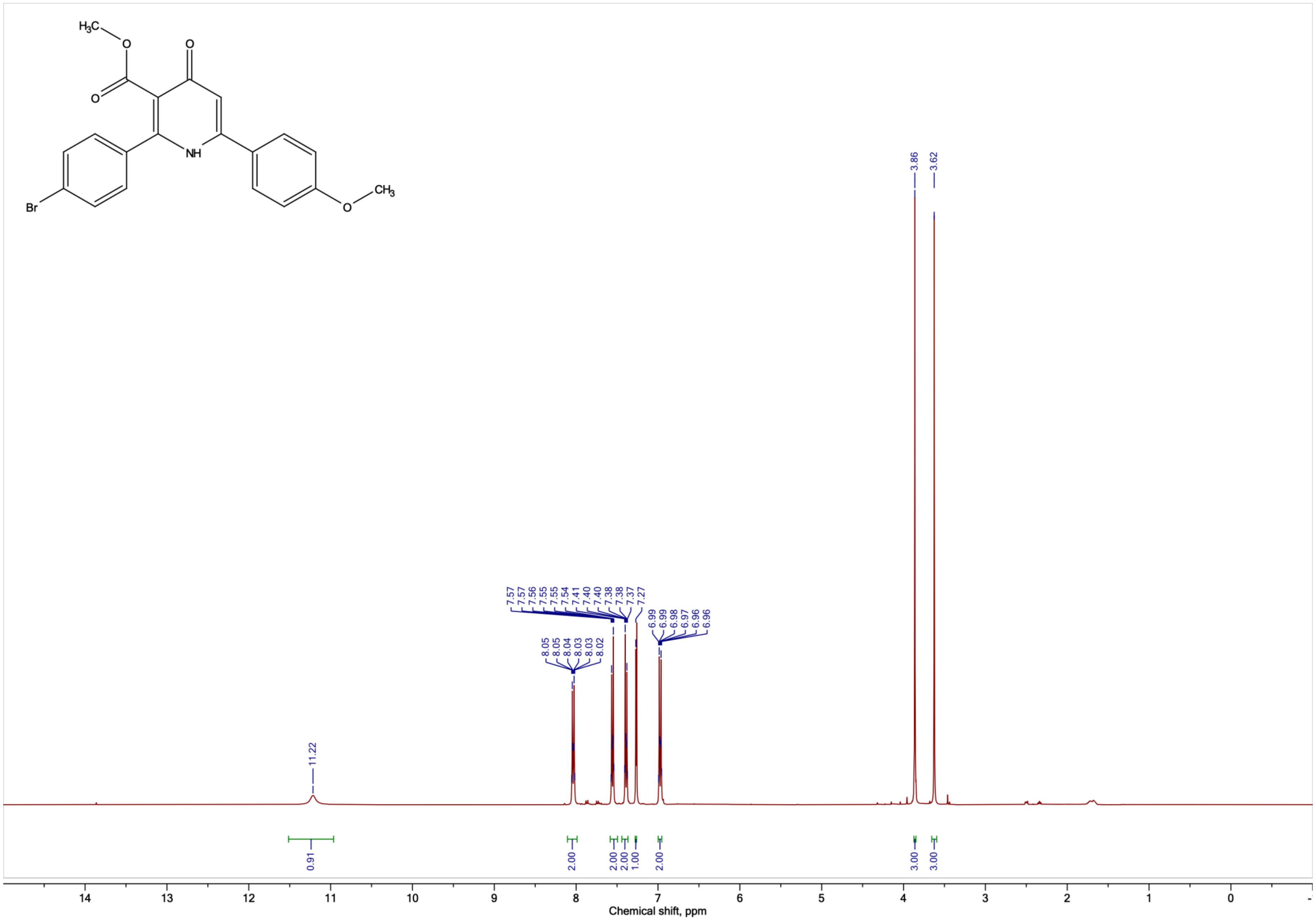

Methyl 2-(4-bromophenyl)-6-(4-methoxyphenyl)-4-oxo-1,4-dihydropyridine-3-carboxylate (2d), $^{13}C\{^1H\}$ NMR, $CDCl_3$, 101 MHz

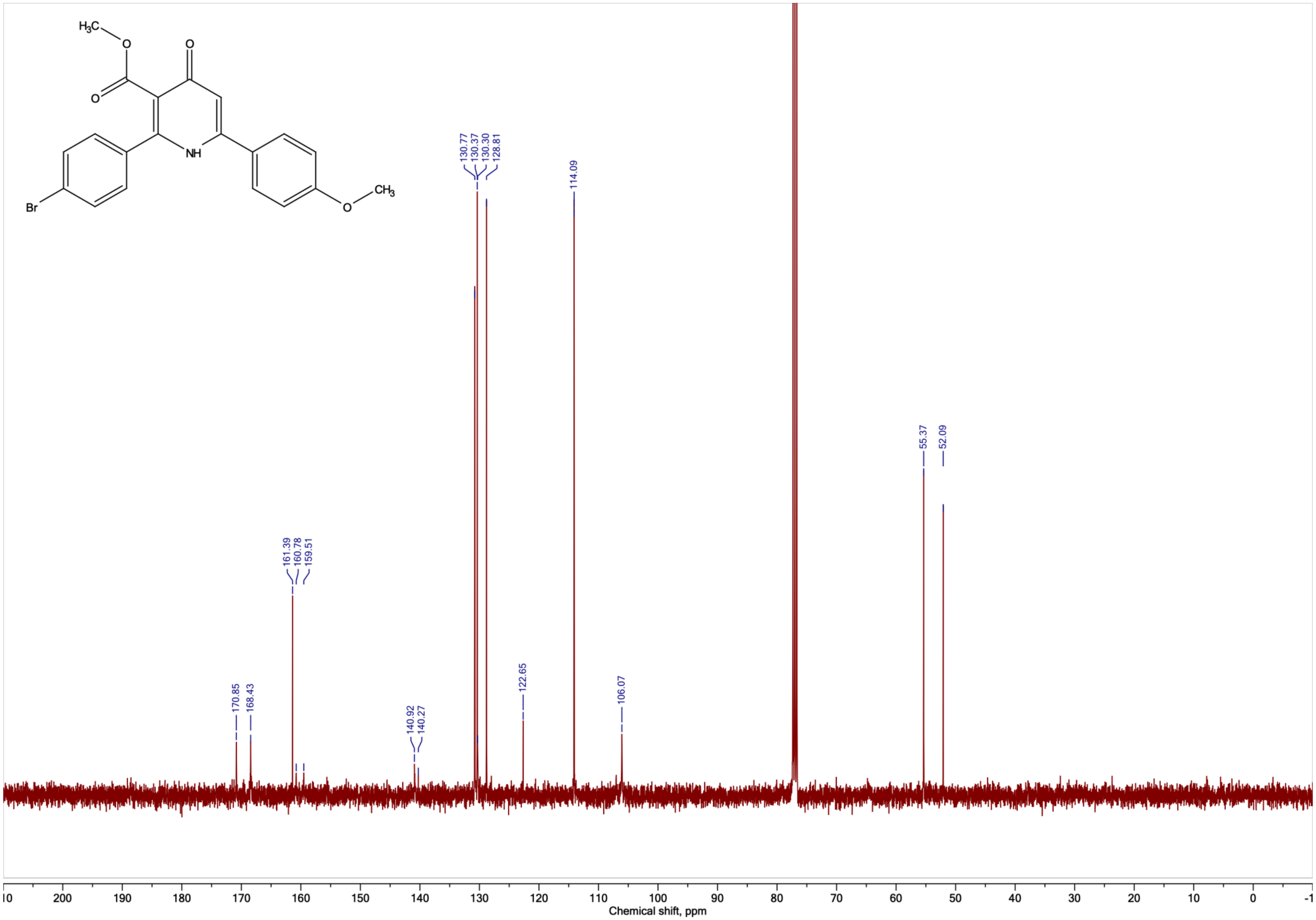

Methyl 2-(4-bromophenyl)-6-(4-methoxyphenyl)-4-oxo-1,4-dihydropyridine-3-carboxylate (2d), DEPT, $CDCl_3$, 101 MHz

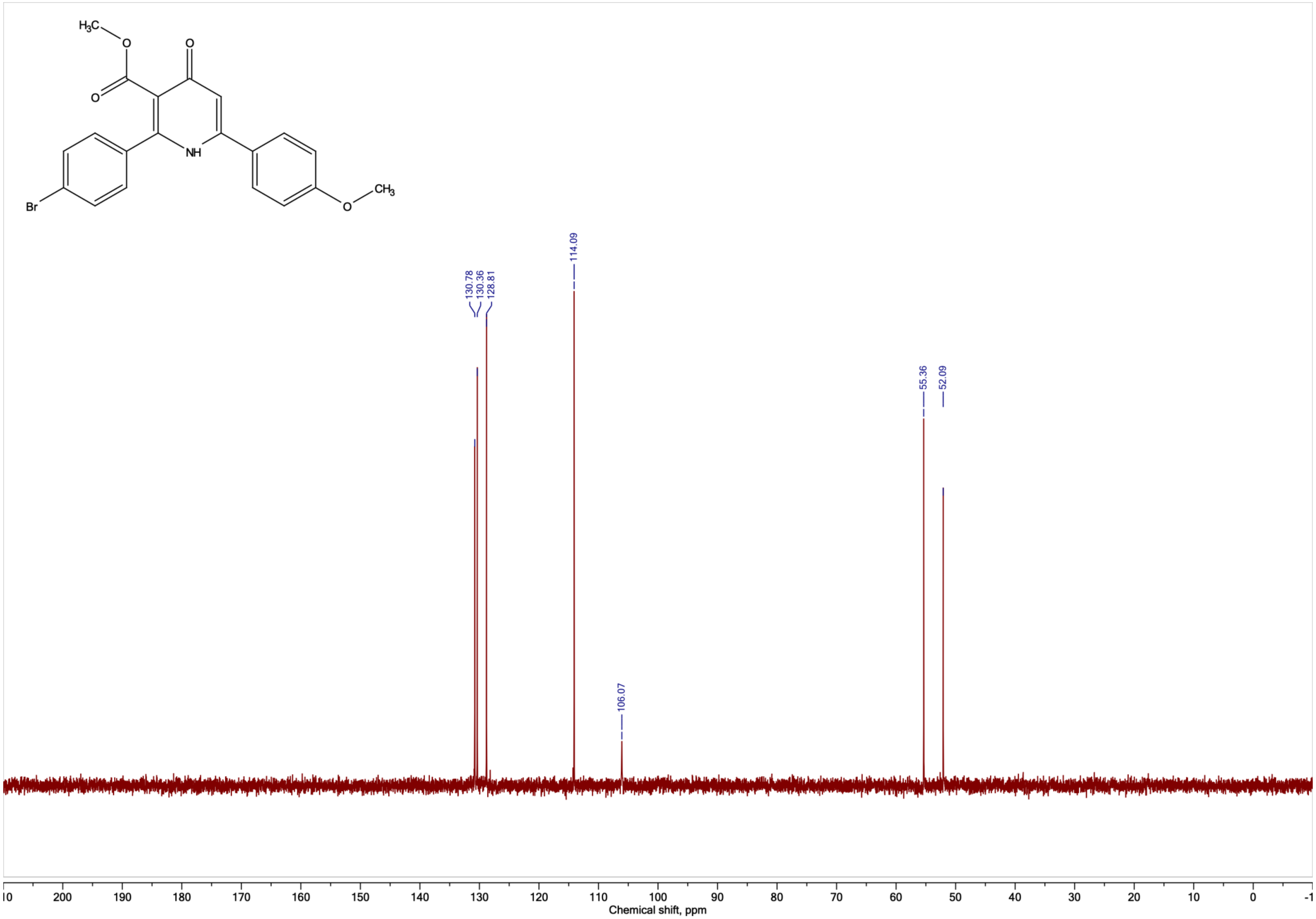

Methyl 6-(4-fluorophenyl)-4-oxo-2-phenyl-1,4-dihydropyridine-3-carboxylate (2e), ^{1}H NMR, $CDCl_3$, 400 MHz

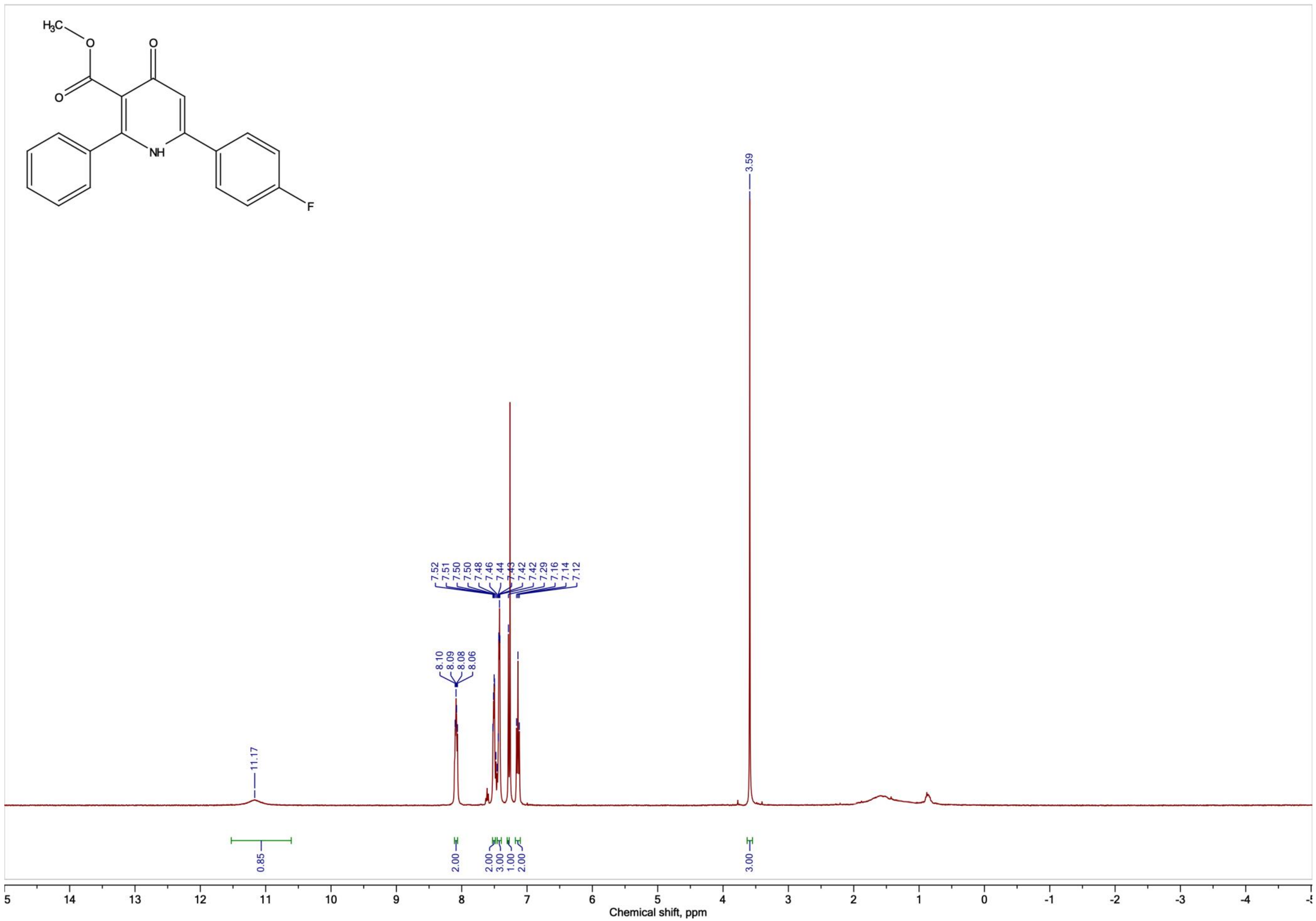

Methyl 6-(4-fluorophenyl)-4-oxo-2-phenyl-1,4-dihydropyridine-3-carboxylate (2e), $^{13}C\{^{1}H\}$ NMR, $CDCl_3$, 101 MHz

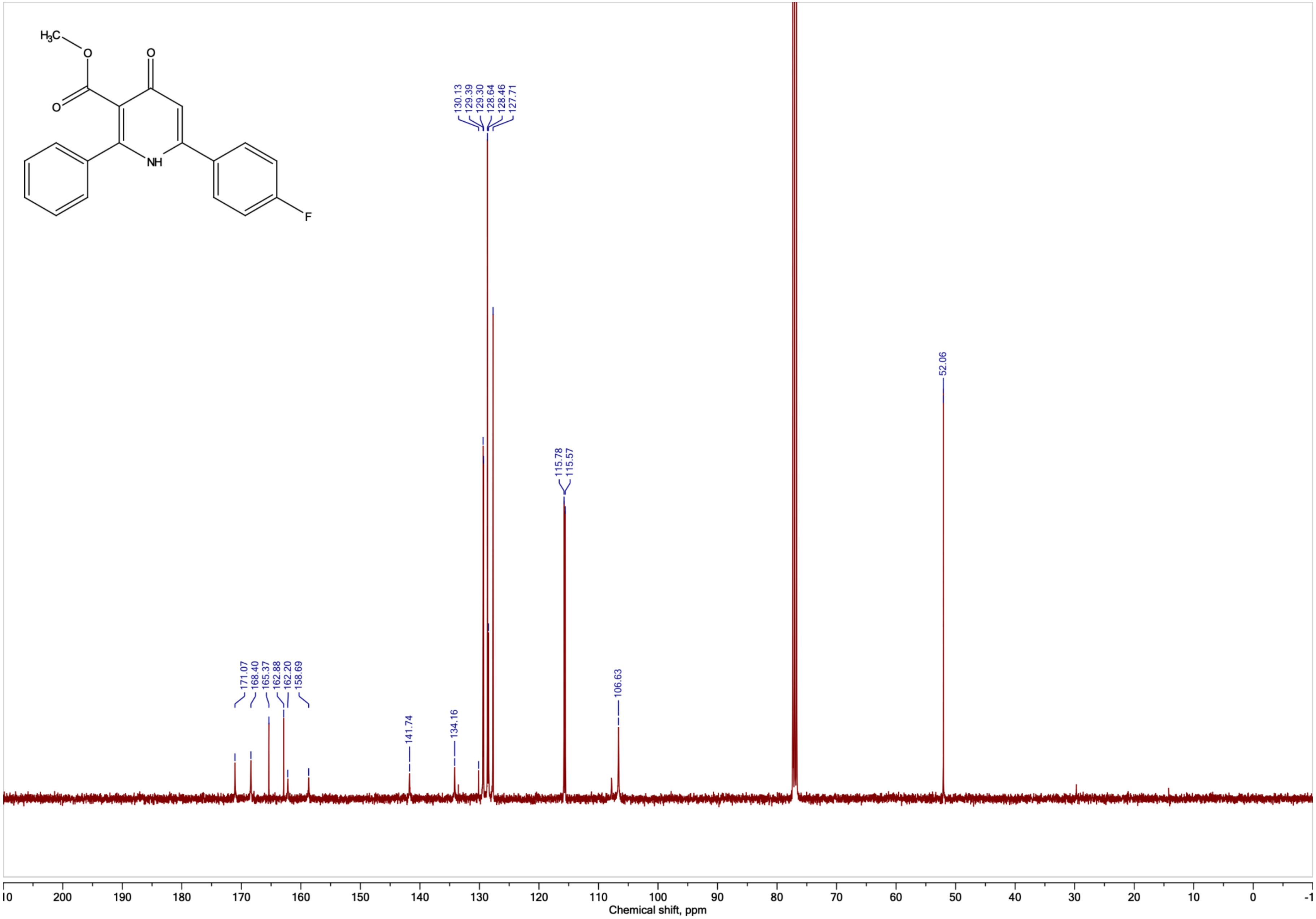

Methyl 6-(4-fluorophenyl)-4-oxo-2-phenyl-1,4-dihydropyridine-3-carboxylate (2e), DEPT, $CDCl_3$, 101 MHz

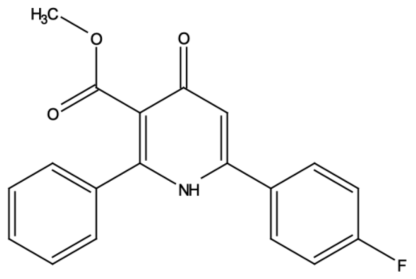

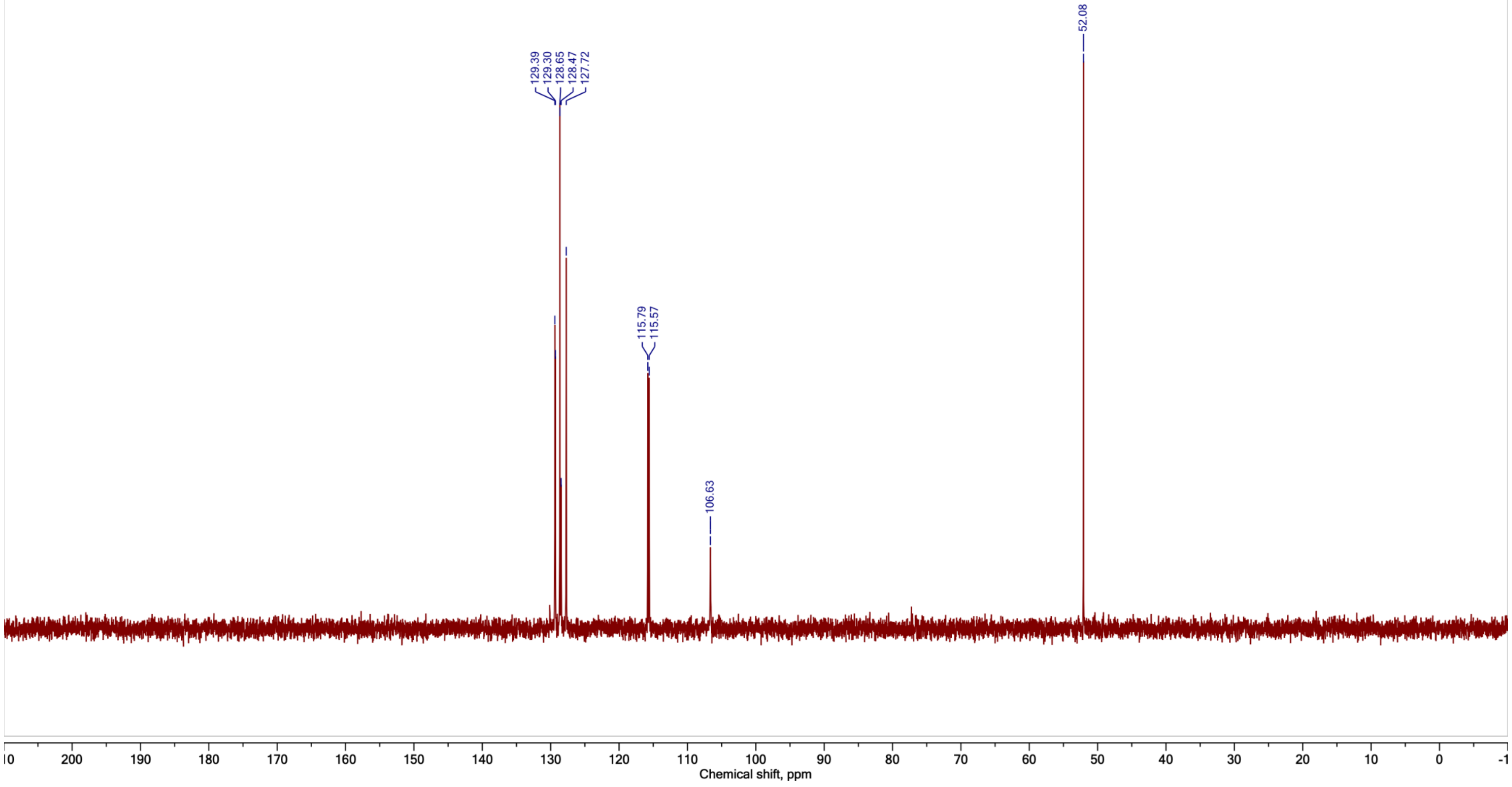

Methyl 6-(4-chlorophenyl)-4-oxo-2-phenyl-1,4-dihydropyridine-3-carboxylate (2f), ^{1}H NMR, $CDCl_3$, 400 MHz

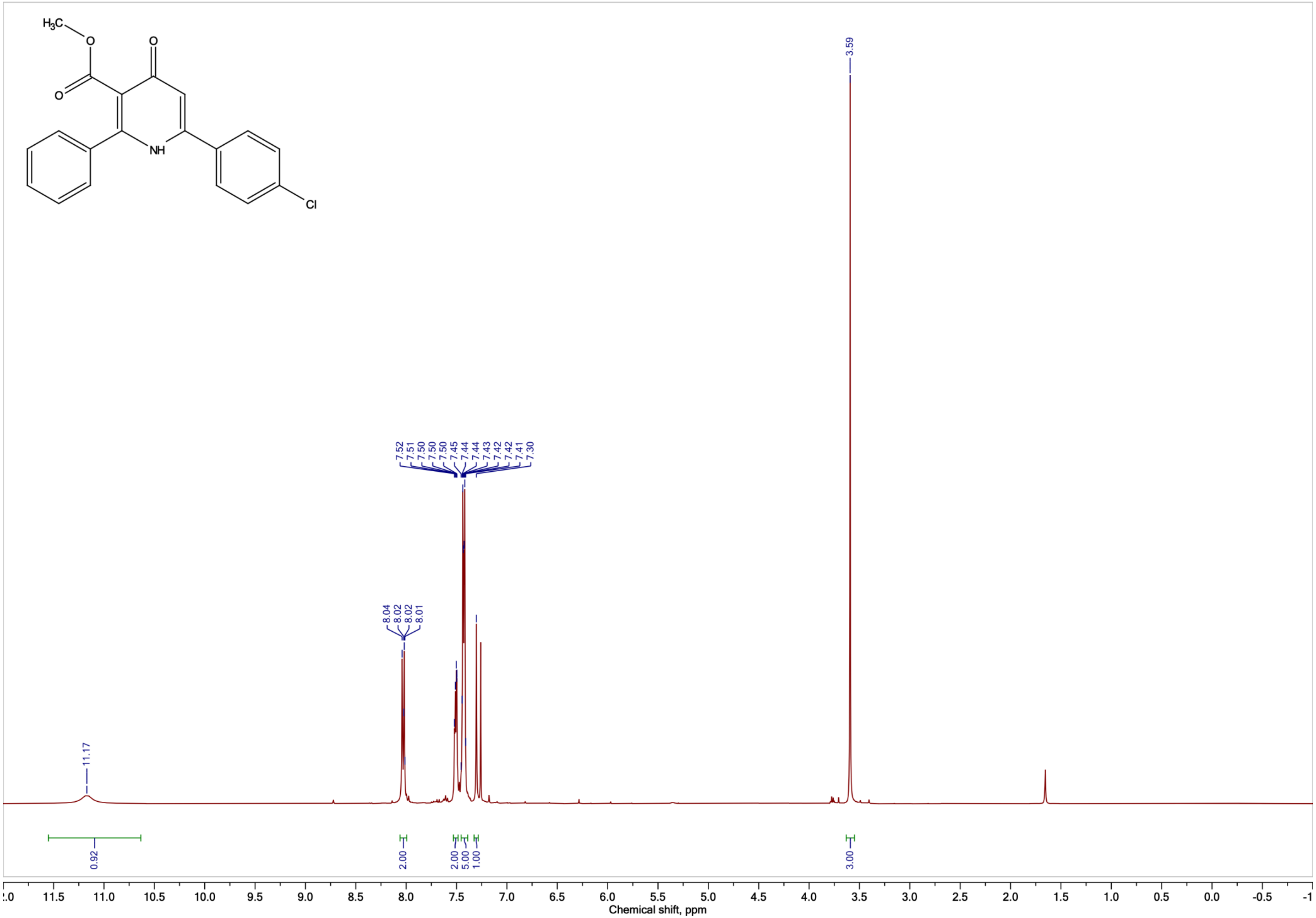

Methyl 6-(4-chlorophenyl)-4-oxo-2-phenyl-1,4-dihydropyridine-3-carboxylate (2f), $^{13}C\{^{1}H\}$ NMR, $CDCl_3$, 101 MHz

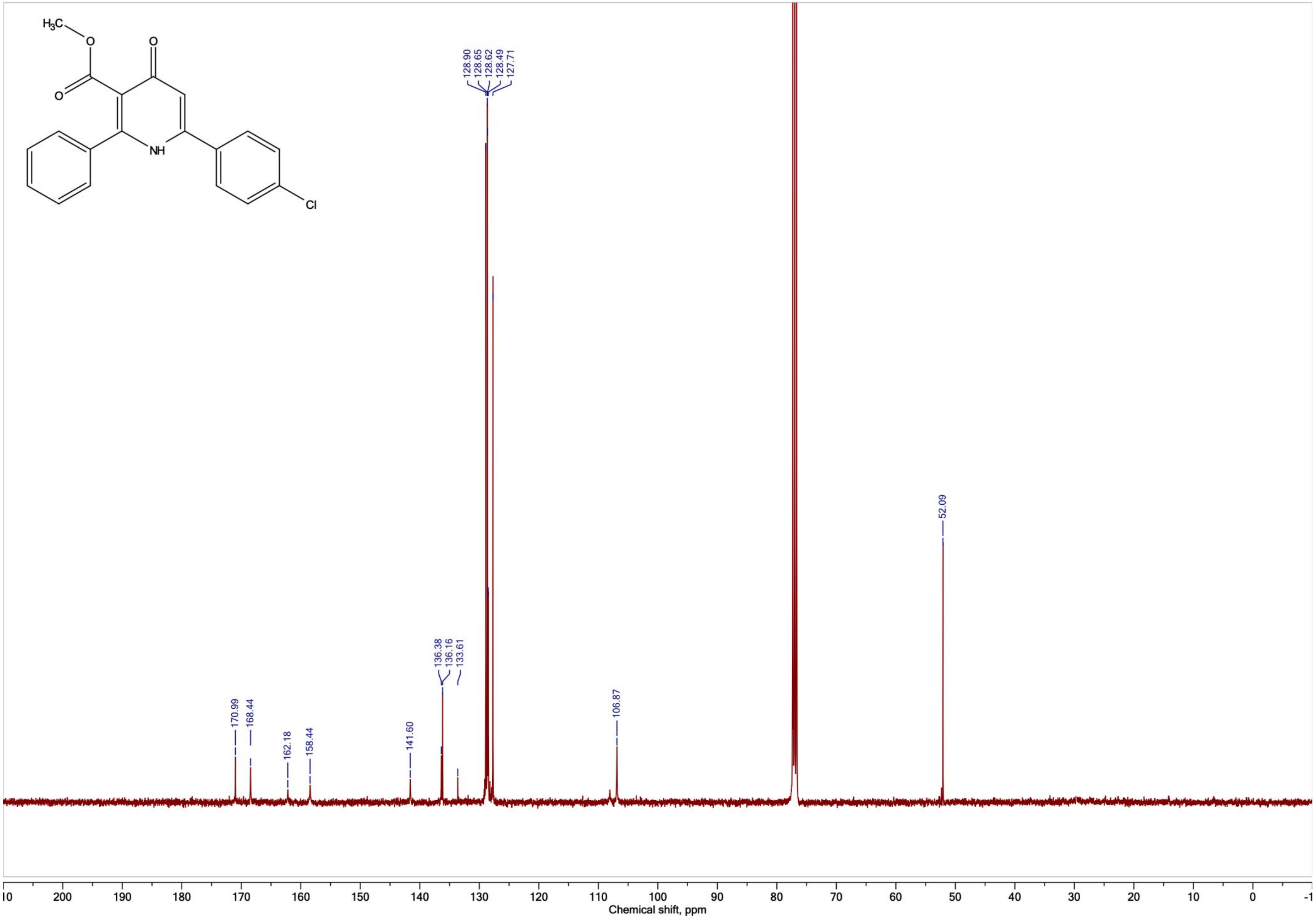

Methyl 6-(4-chlorophenyl)-4-oxo-2-phenyl-1,4-dihydropyridine-3-carboxylate (2f), DEPT, $CDCl_3$, 101 MHz

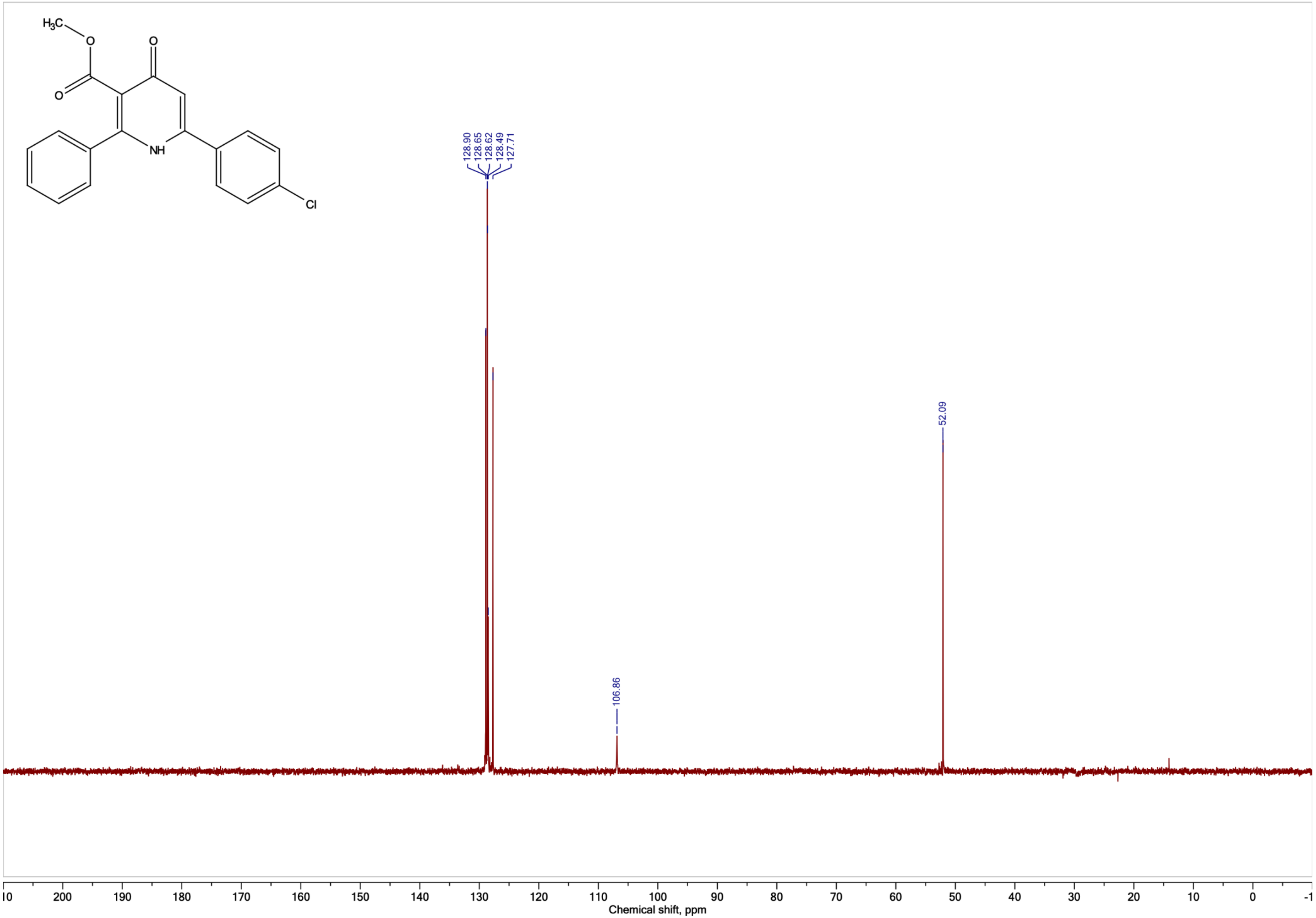

Methyl 6-(4-chlorophenyl)-4-oxo-2-(*p*-tolyl)-1,4-dihydropyridine-3-carboxylate (2g), ^{1}H NMR, $CDCl_3$, 400 MHz

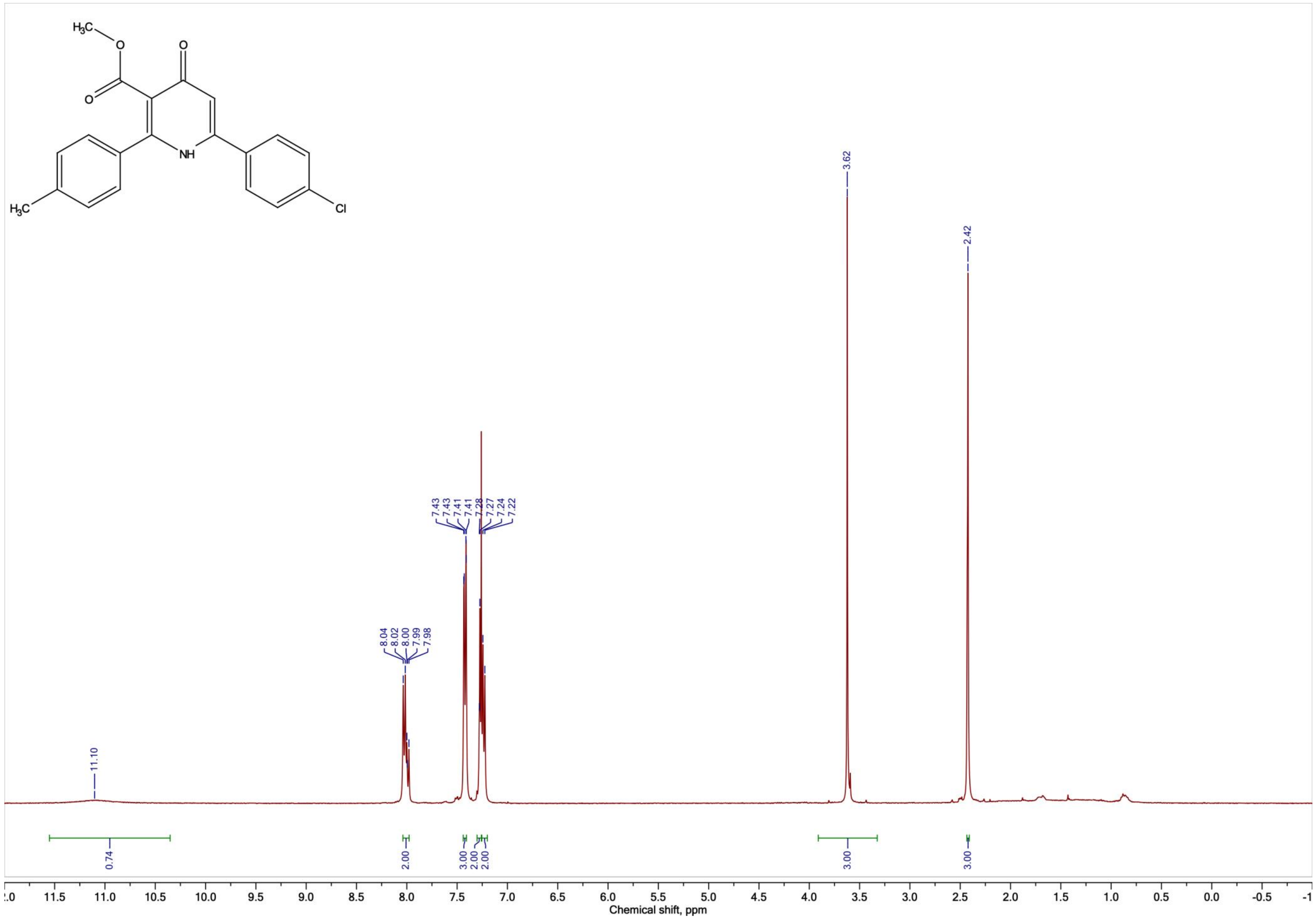

Methyl 6-(4-chlorophenyl)-4-oxo-2-(*p*-tolyl)-1,4-dihydropyridine-3-carboxylate (2g), $^{13}C\{^{1}H\}$ NMR, $CDCl_3$, 101 MHz

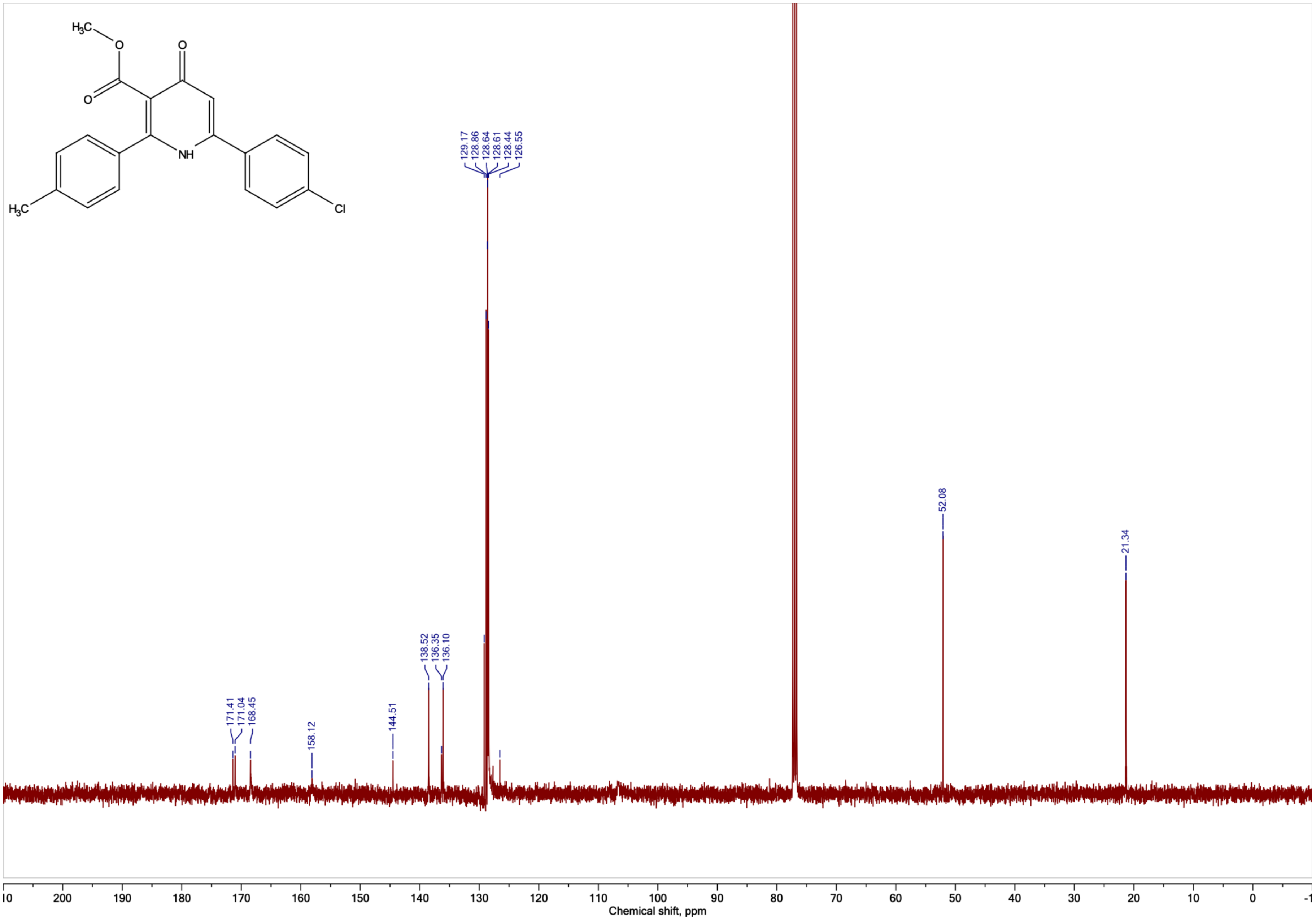

Methyl 6-(4-chlorophenyl)-4-oxo-2-(*p*-tolyl)-1,4-dihydropyridine-3-carboxylate (2g), DEPT, $CDCl_3$, 101 MHz

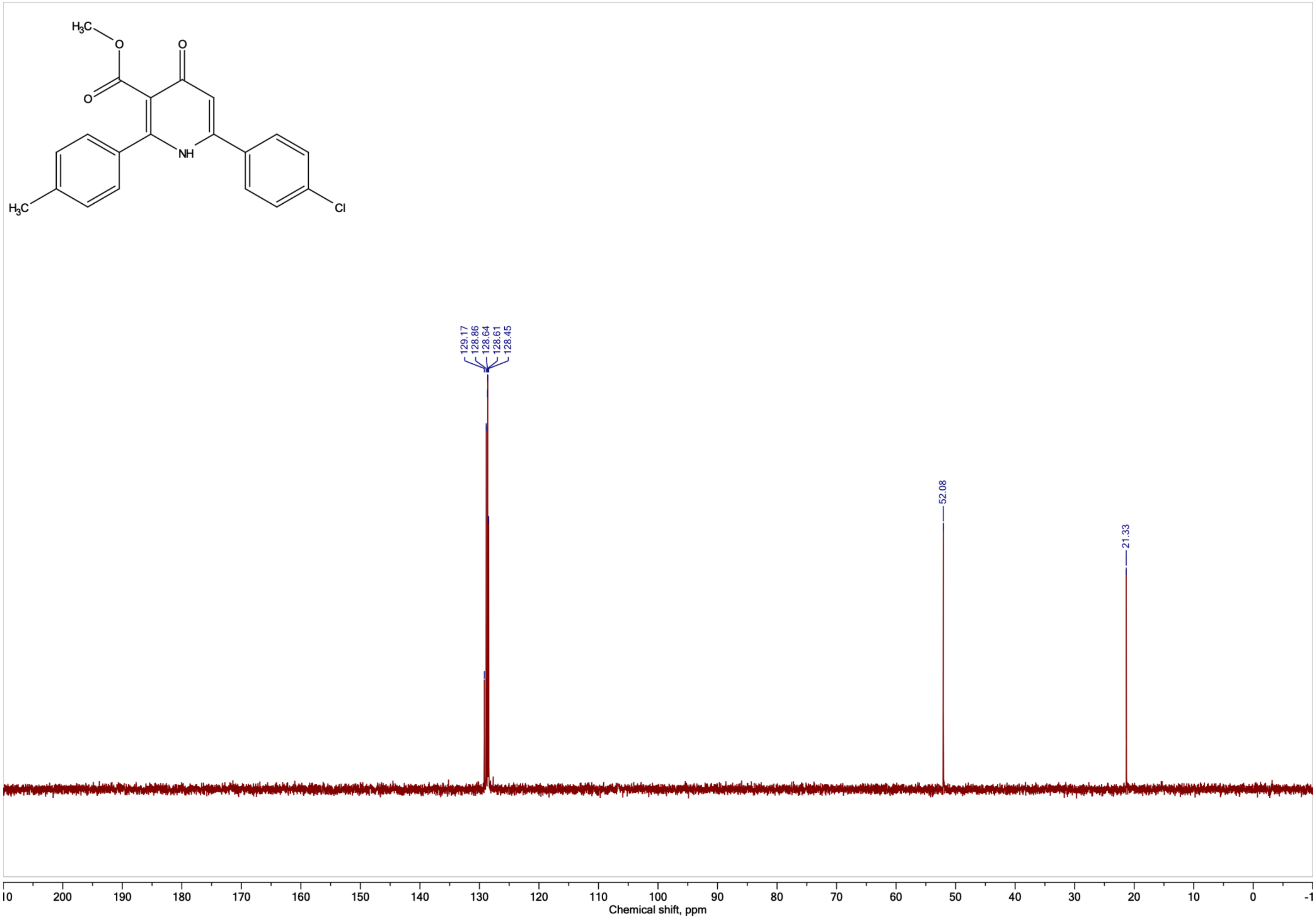

Methyl 6-(4-bromophenyl)-4-oxo-2-phenyl-1,4-dihydropyridine-3-carboxylate (2h), ^{1}H NMR, $CDCl_3$, 400 MHz

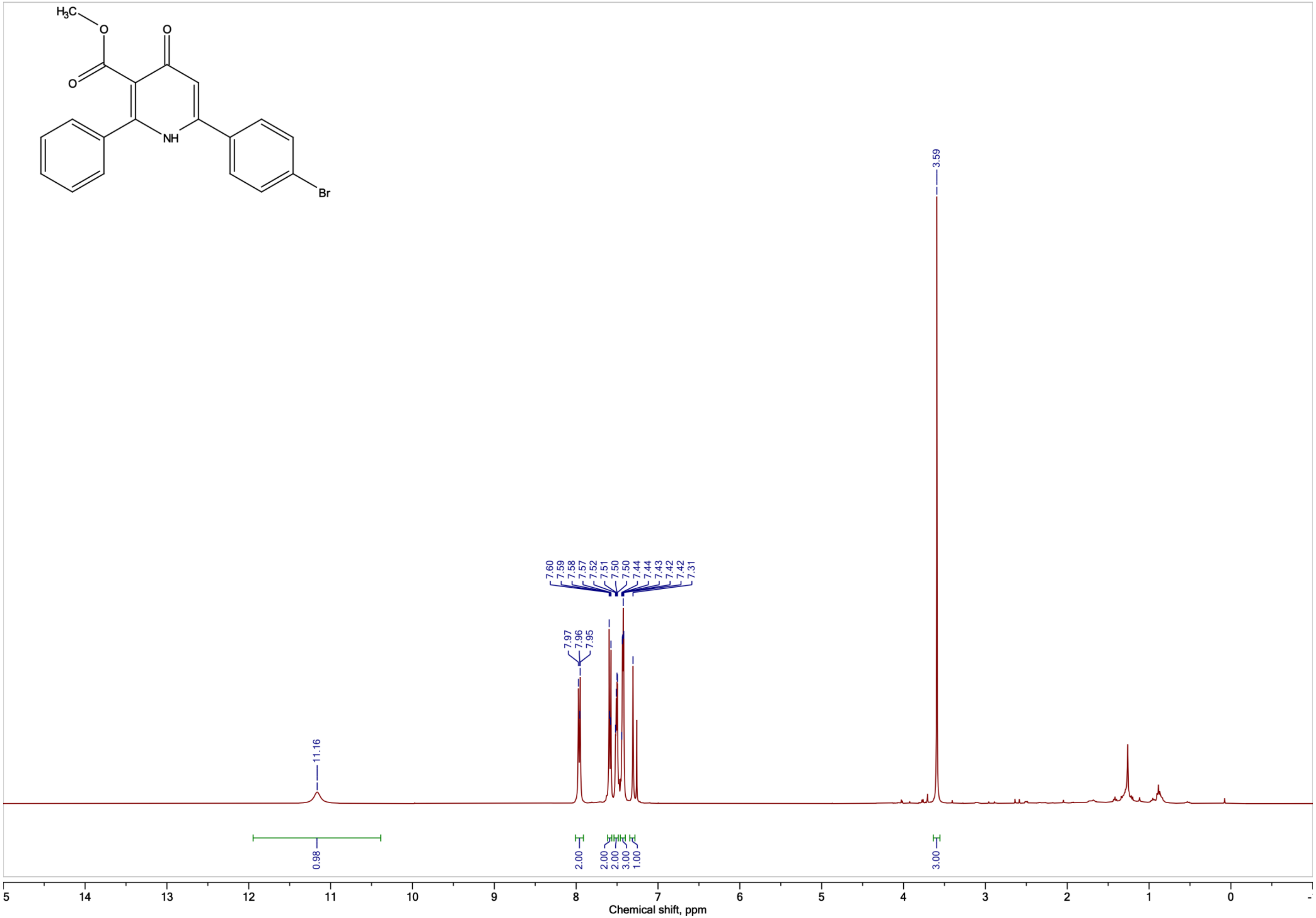

Methyl 6-(4-bromophenyl)-4-oxo-2-phenyl-1,4-dihydropyridine-3-carboxylate (2h), $^{13}C\{^1H\}$ NMR, $CDCl_3$, 101 MHz

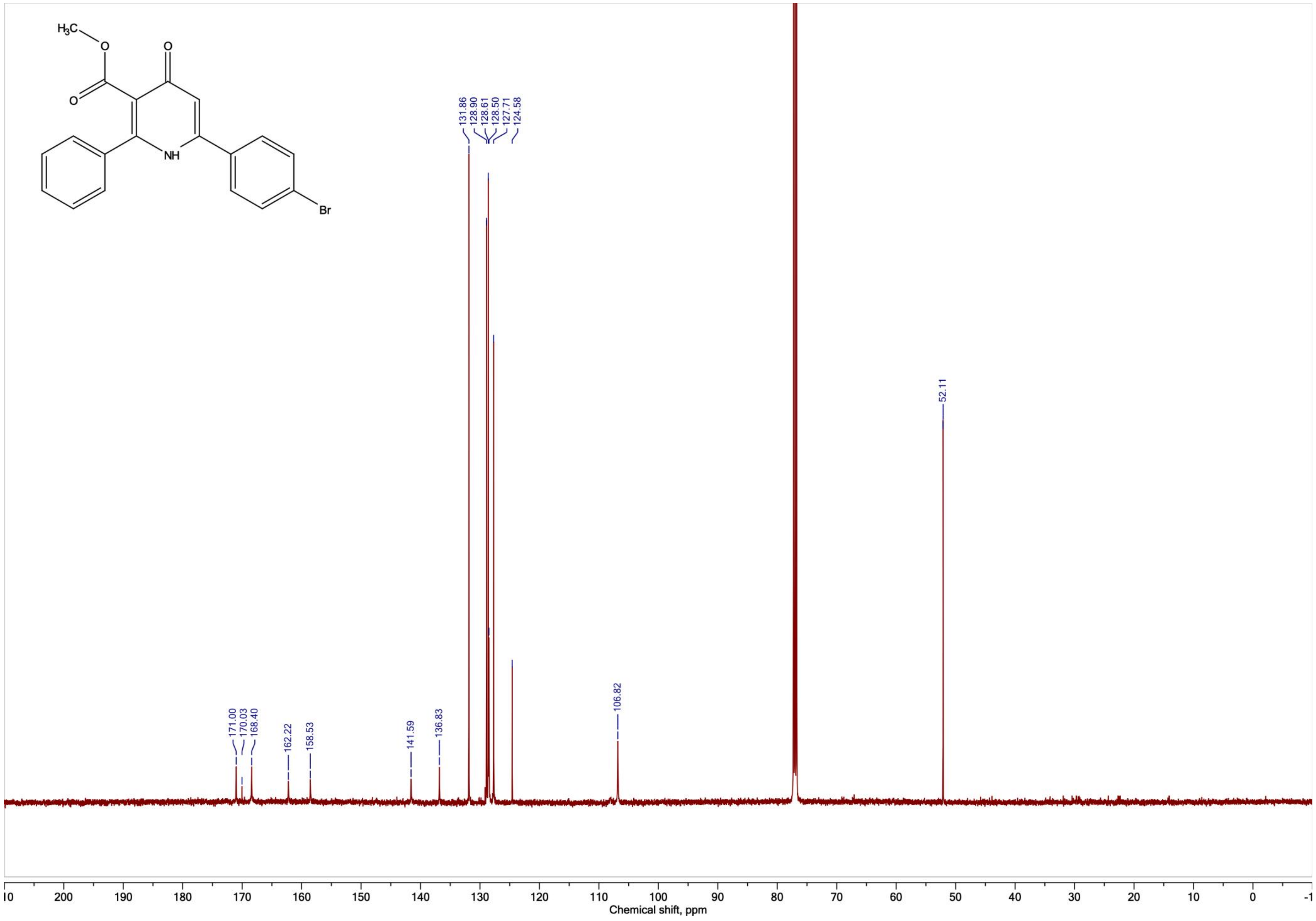

Methyl 6-(4-bromophenyl)-4-oxo-2-phenyl-1,4-dihydropyridine-3-carboxylate (2h), DEPT, $CDCl_3$, 101 MHz

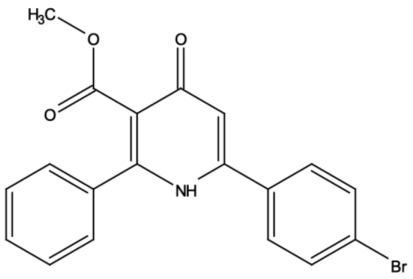

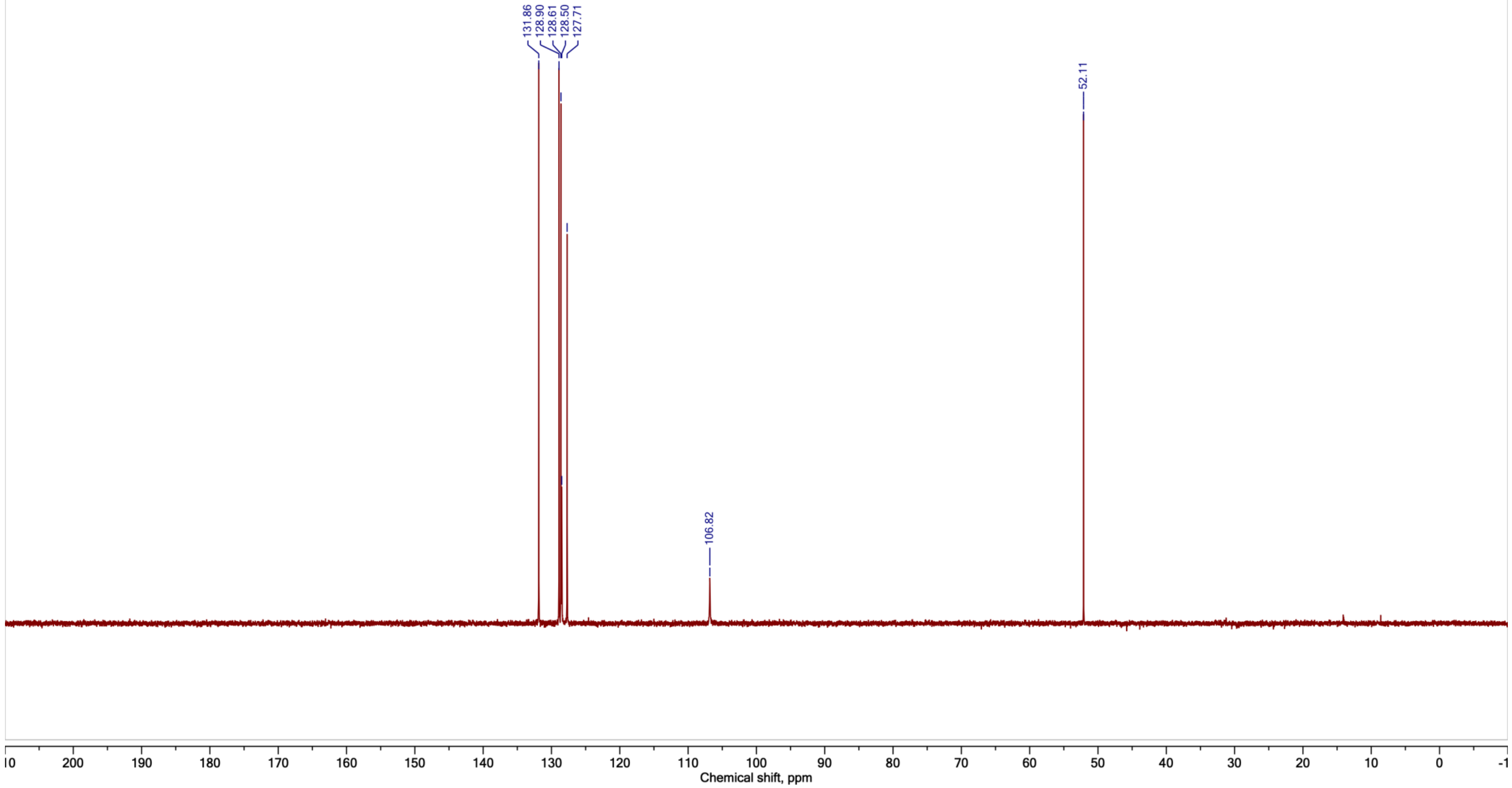

Methyl 6-(4-bromophenyl)-2-methyl-4-oxo-1,4-dihydropyridine-3-carboxylate (2i), ^{1}H NMR, $CDCl_3$, 400 MHz

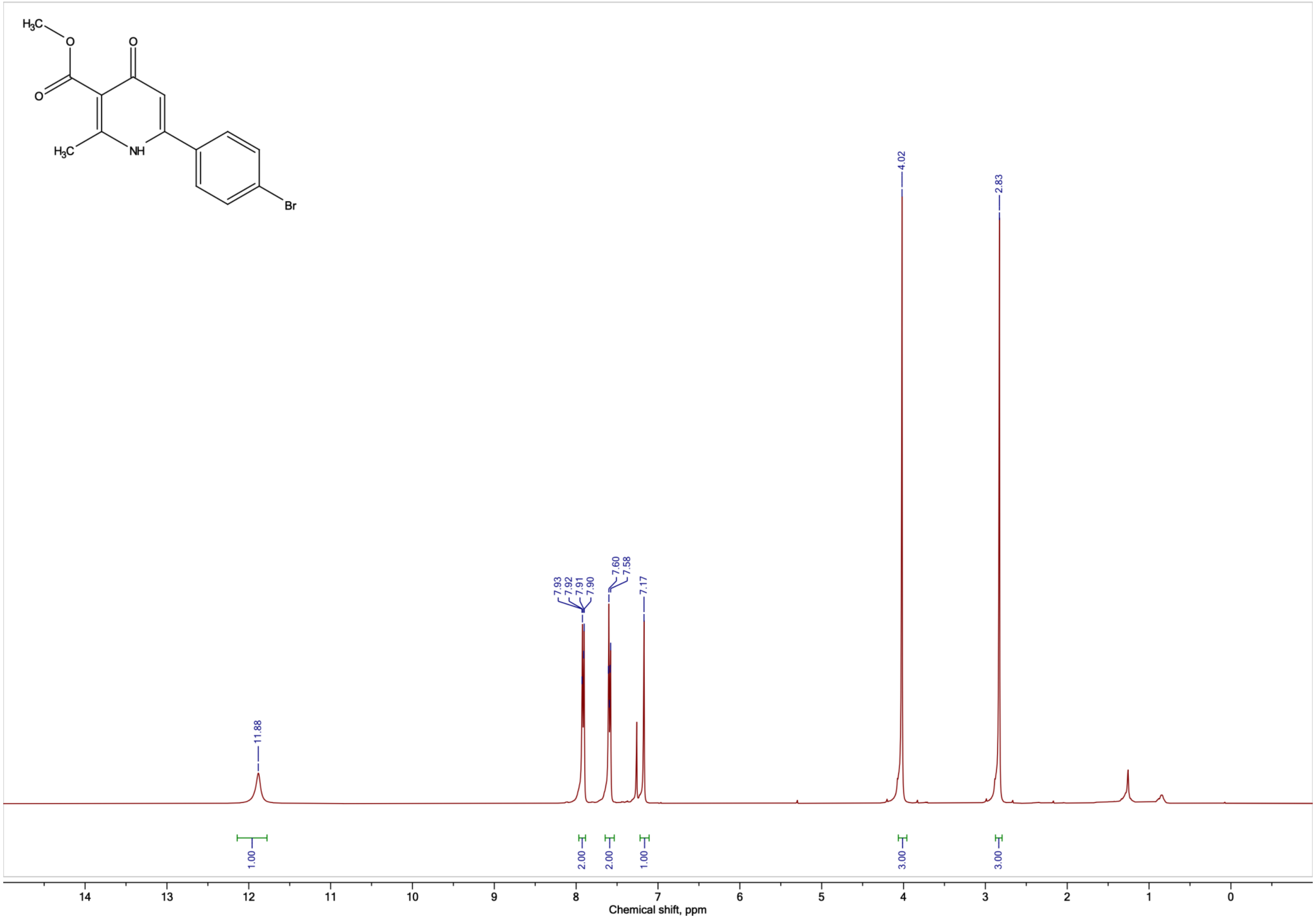

Methyl 6-(4-bromophenyl)-2-methyl-4-oxo-1,4-dihydropyridine-3-carboxylate (2i), $^{13}C\{^1H\}$ NMR, $CDCl_3$, 101 MHz

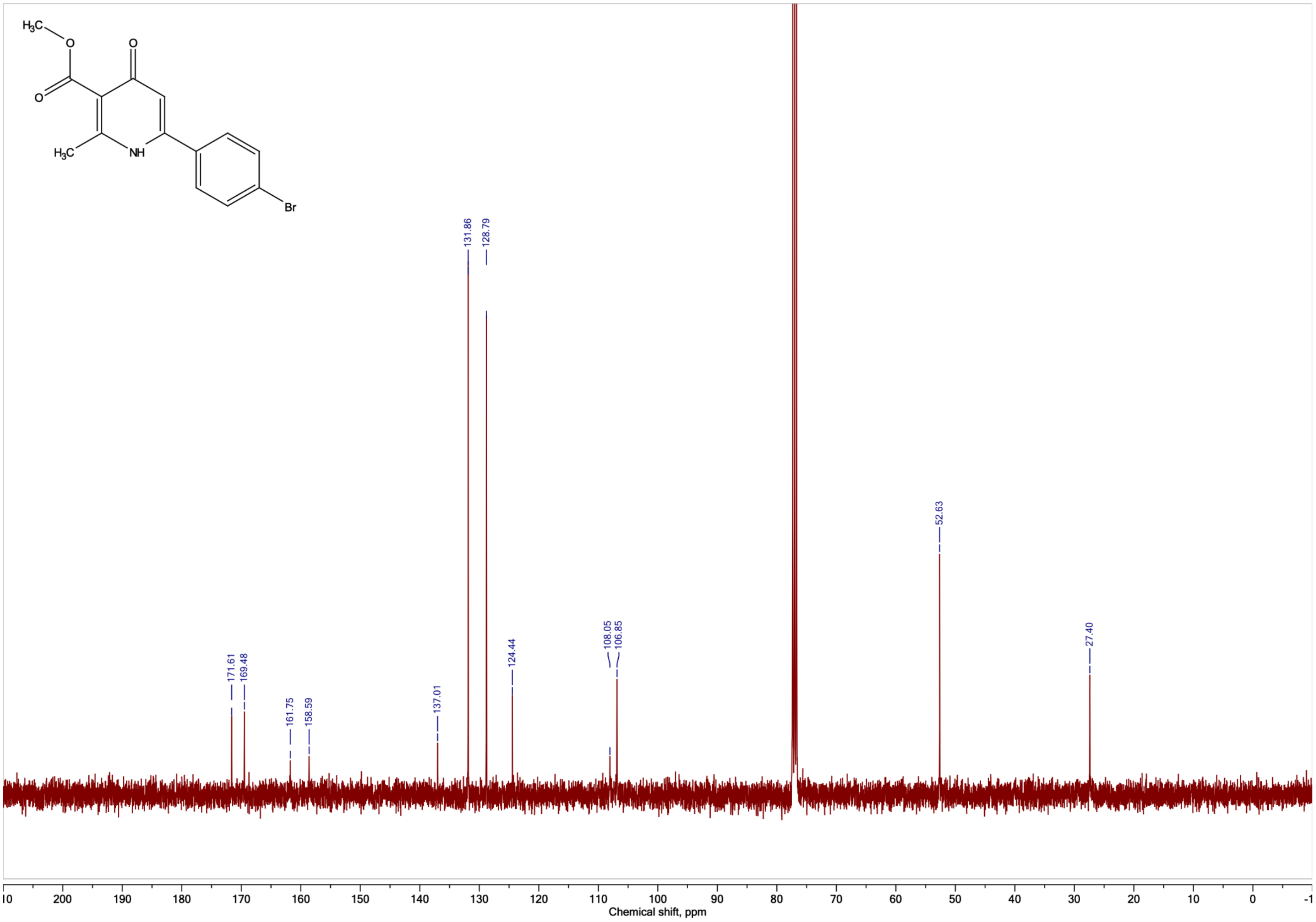

Methyl 6-(4-bromophenyl)-2-methyl-4-oxo-1,4-dihydropyridine-3-carboxylate (2i), DEPT, $CDCl_3$, 101 MHz

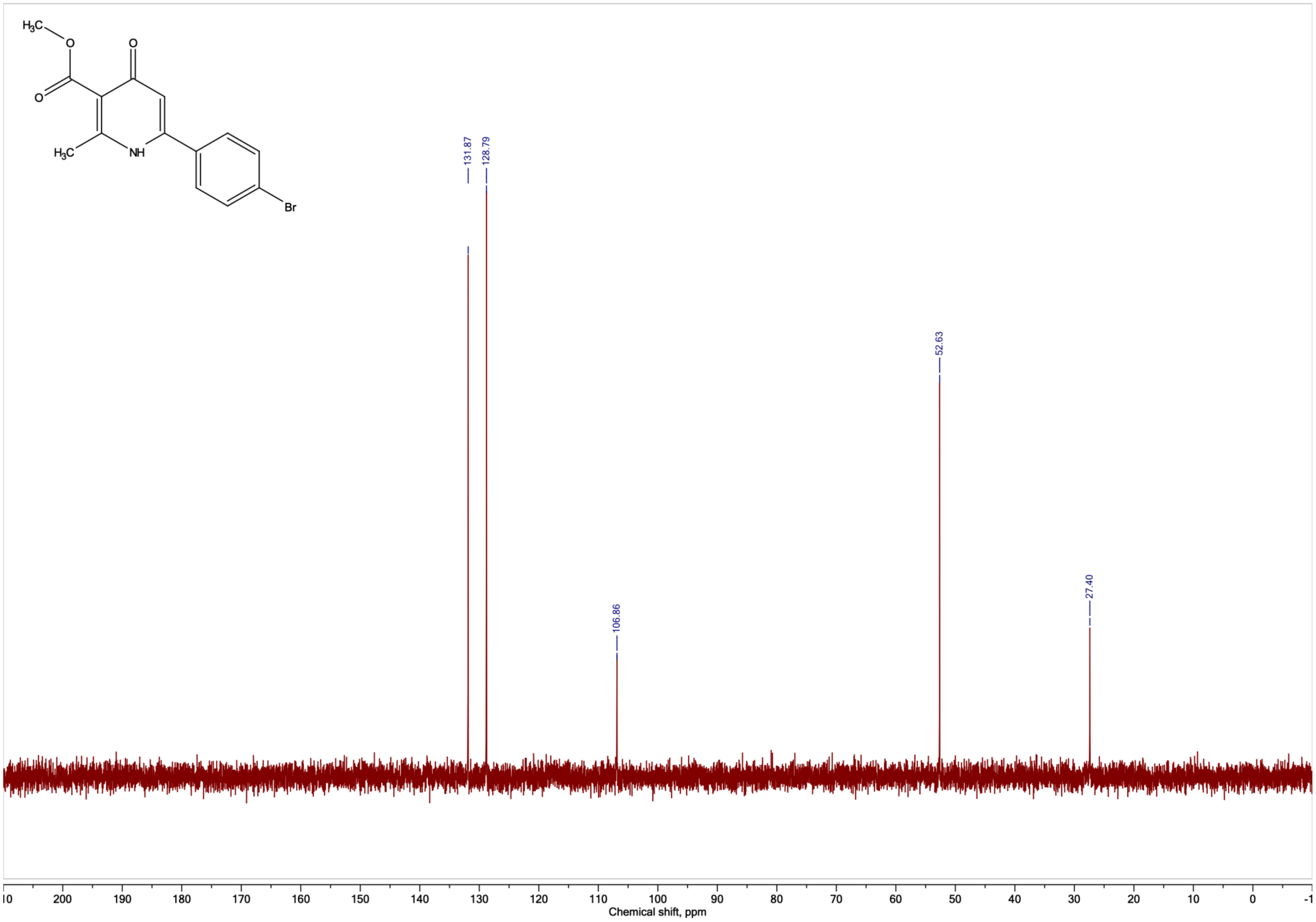

Methyl 6-benzyl-4-oxo-2-(*p*-tolyl)-1,4-dihydropyridine-3-carboxylate (2k), ^{1}H NMR, DMSO-d_6, 400 MHz

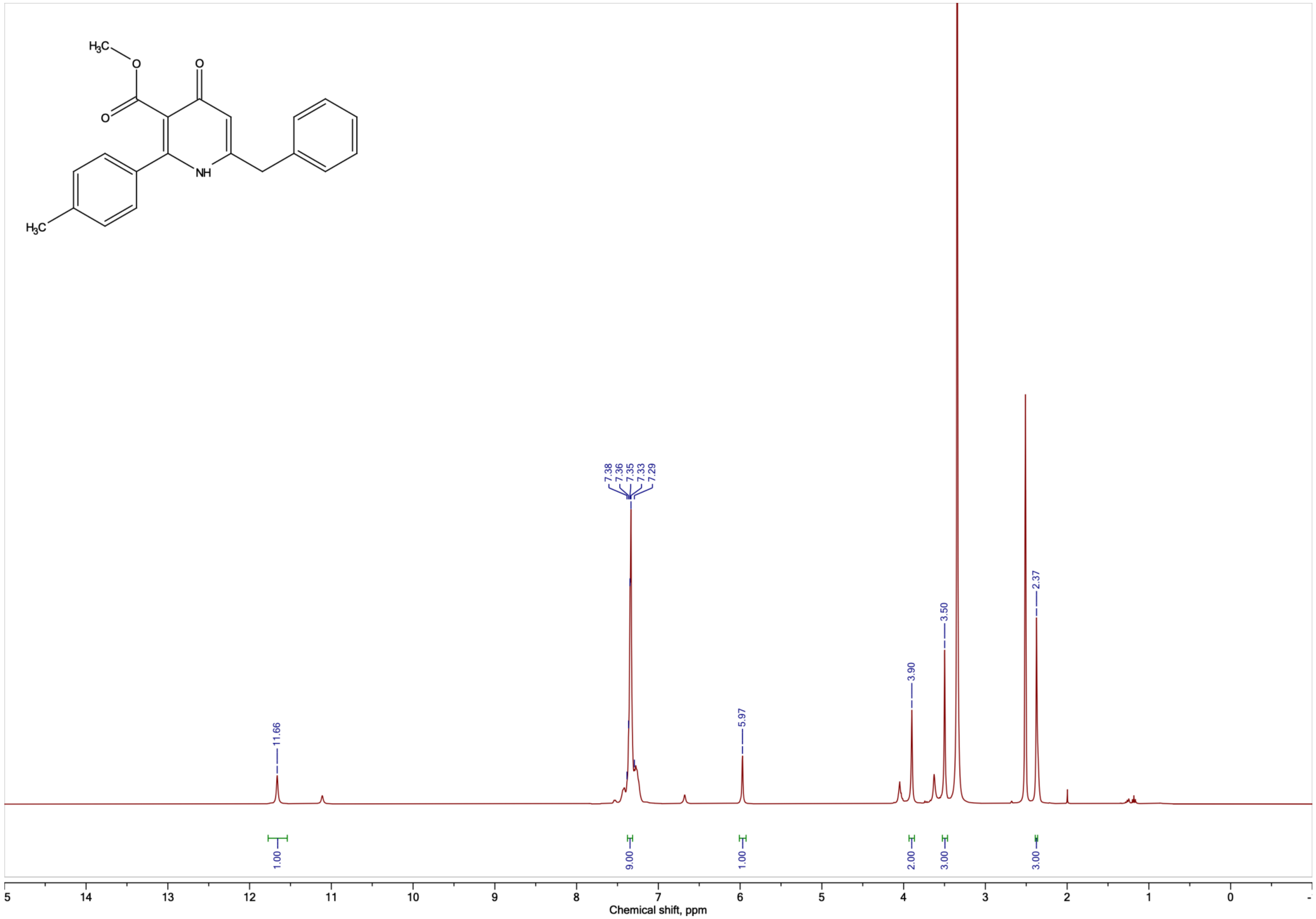

Methyl 6-benzyl-4-oxo-2-(*p*-tolyl)-1,4-dihydropyridine-3-carboxylate (2k), $^{13}C\{^{1}H\}$ NMR, DMSO-d_6, 101 MHz

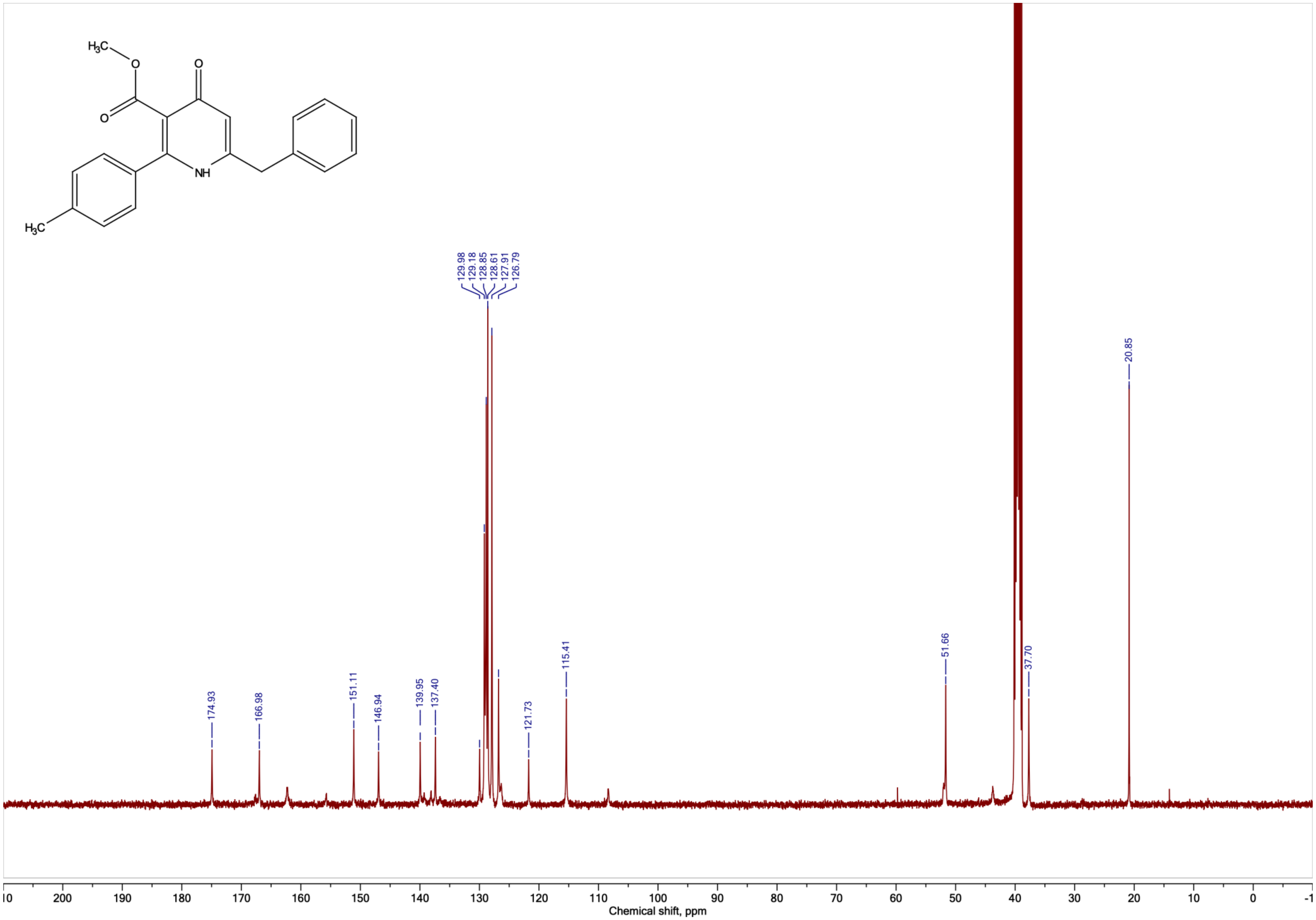

Methyl 6-benzyl-4-oxo-2-(*p*-tolyl)-1,4-dihydropyridine-3-carboxylate (2k), DEPT, DMSO-d_6, 101 MHz

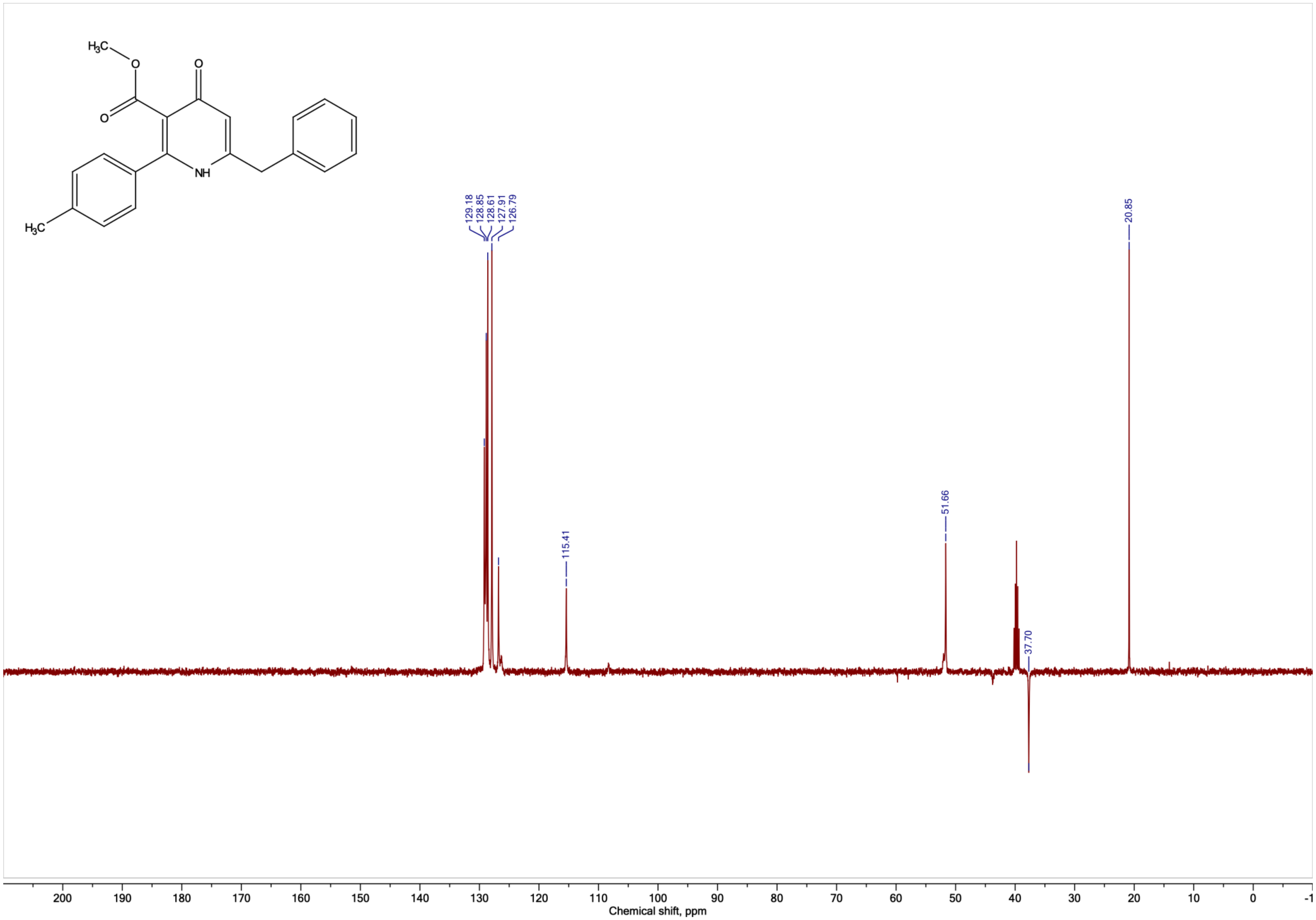

Methyl 4-oxo-6-phenyl-5-(thiophen-3-yl)-2-(*p*-tolyl)-1,4-dihydropyridine-3-carboxylate (2n), ^{1}H NMR, DMSO-d_6, 400 MHz

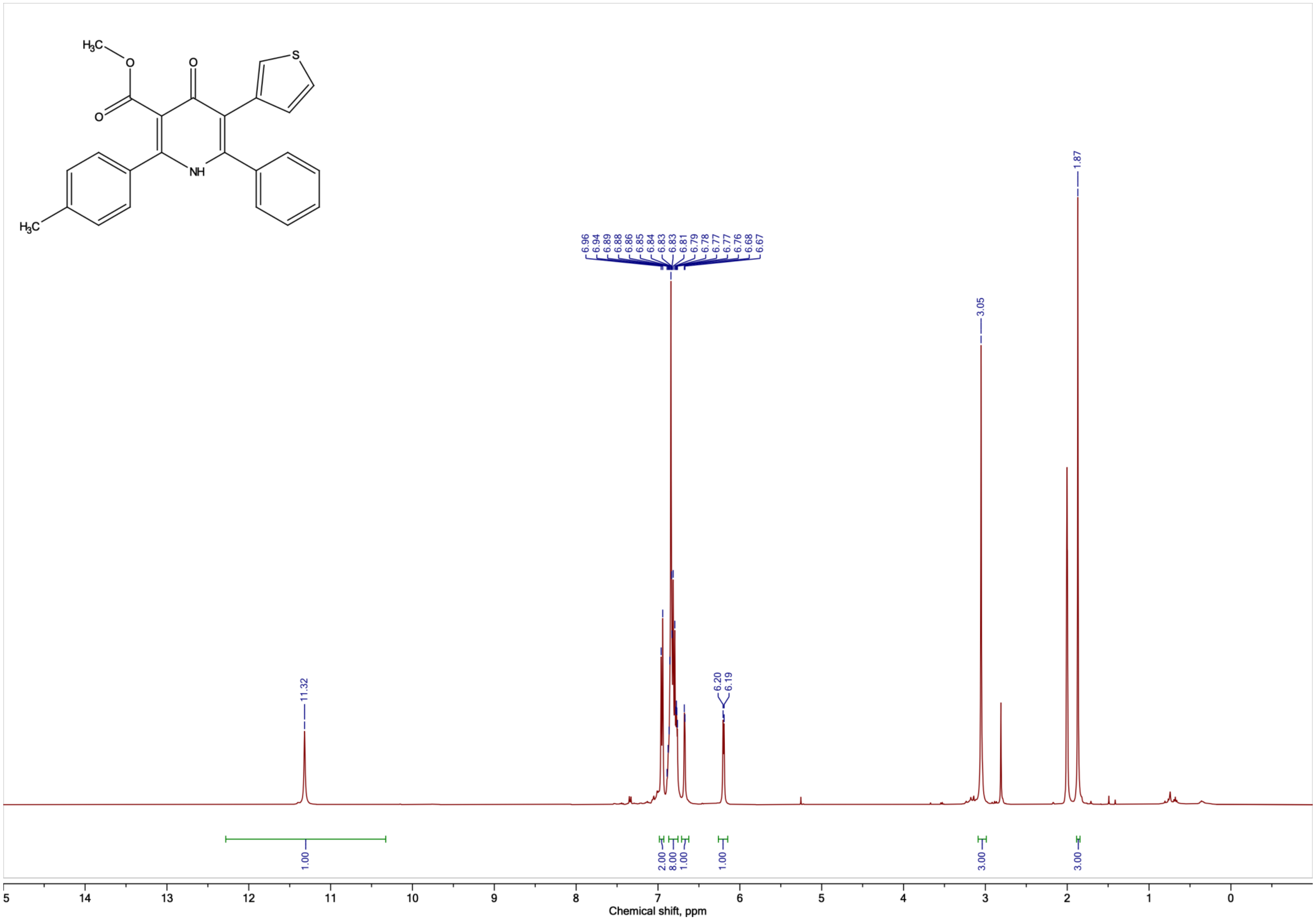

Methyl 4-oxo-6-phenyl-5-(thiophen-3-yl)-2-(*p*-tolyl)-1,4-dihydropyridine-3-carboxylate (2n), $^{13}C\{^{1}H\}$ NMR, DMSO-d_6, 101 MHz

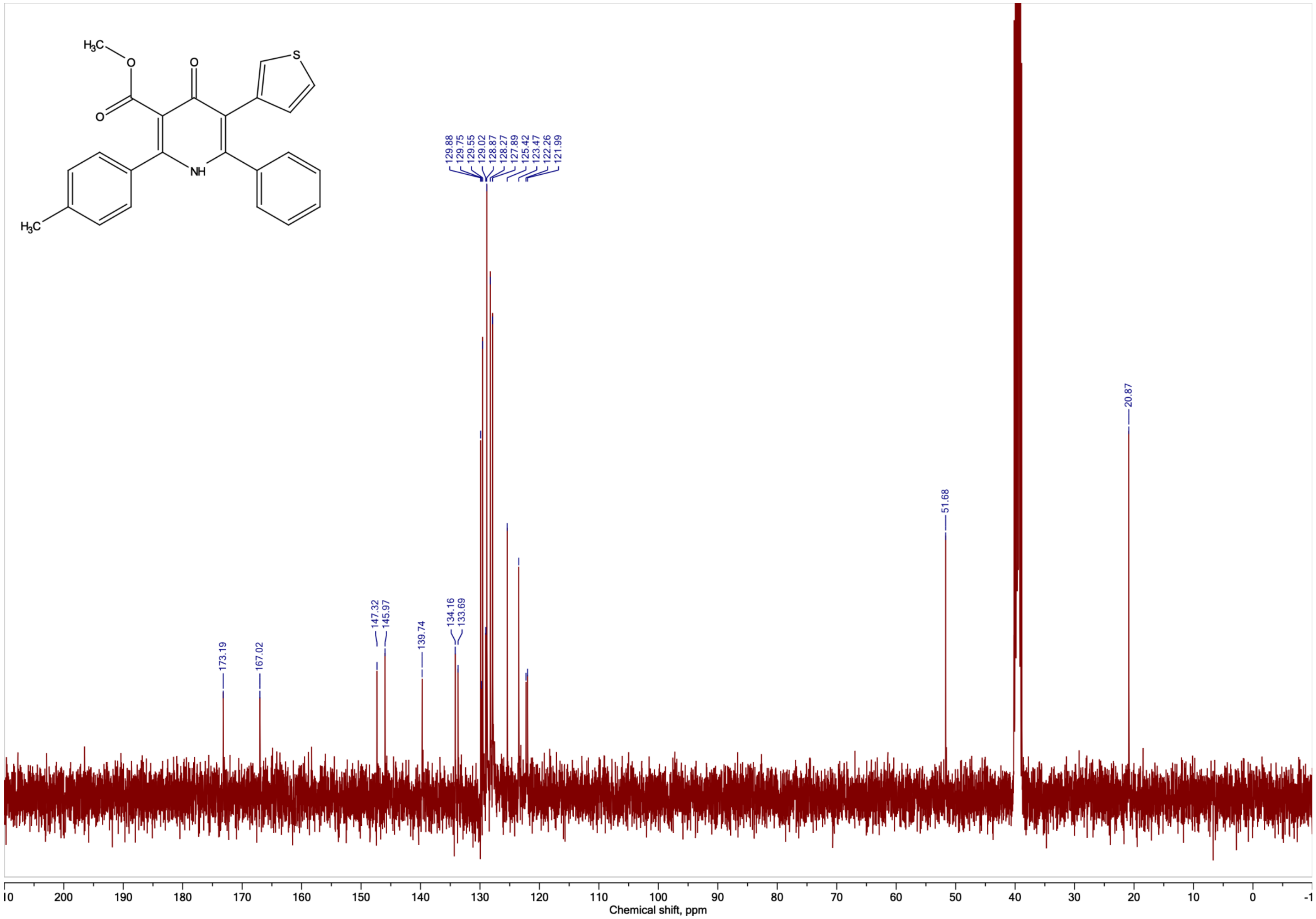

Methyl 4-oxo-6-phenyl-5-(thiophen-3-yl)-2-(*p*-tolyl)-1,4-dihydropyridine-3-carboxylate (2n), DEPT, DMSO-d_6, 101 MHz

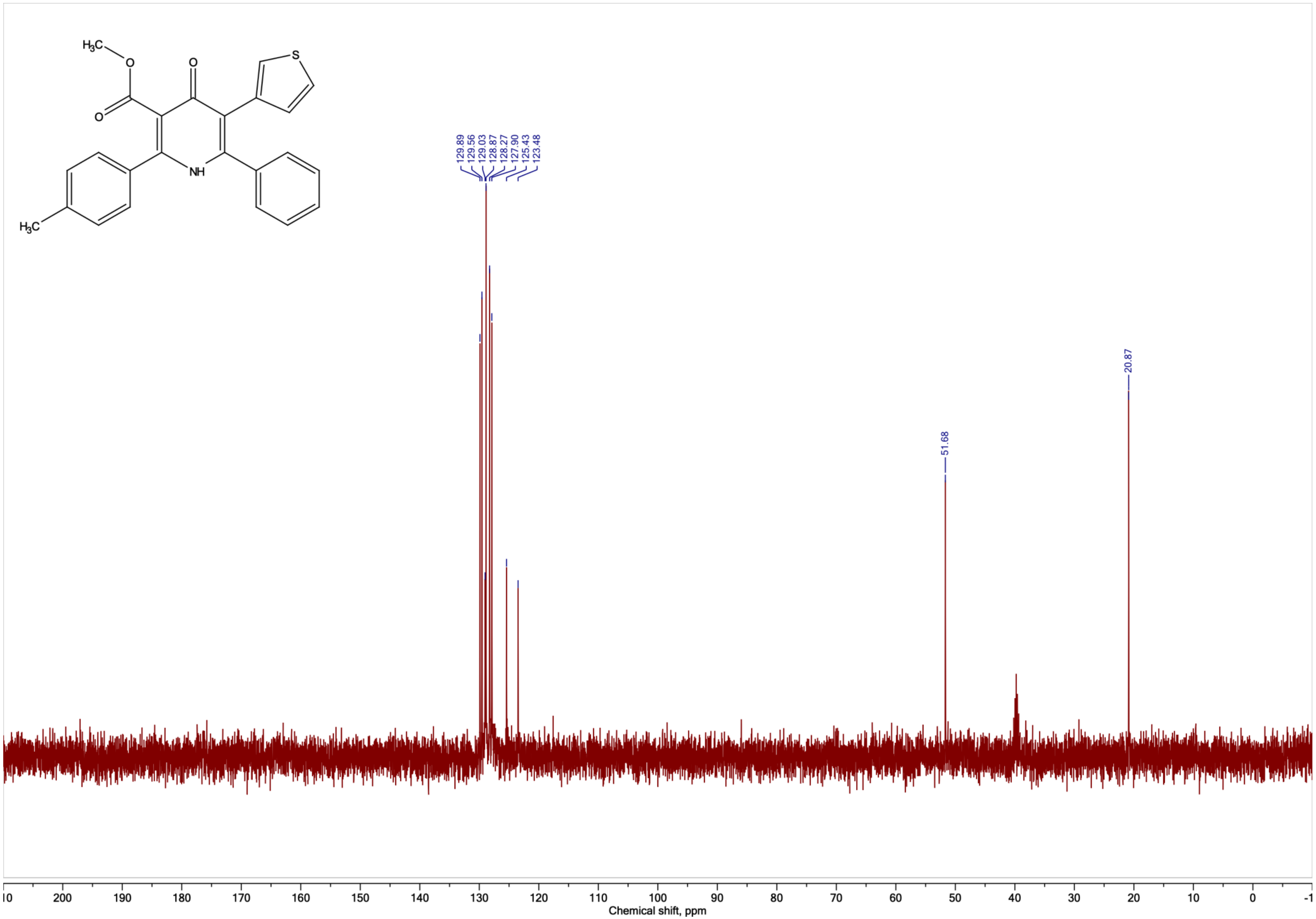

Methyl 6-(4-(*tert*-butyl)phenyl)-4-oxo-2-(*p*-tolyl)-5-(4-(trifluoromethyl)phenyl)-1,4-dihydropyridine-3-carboxylate (2o), ^{1}H NMR, DMSO-d_6, 400 MHz

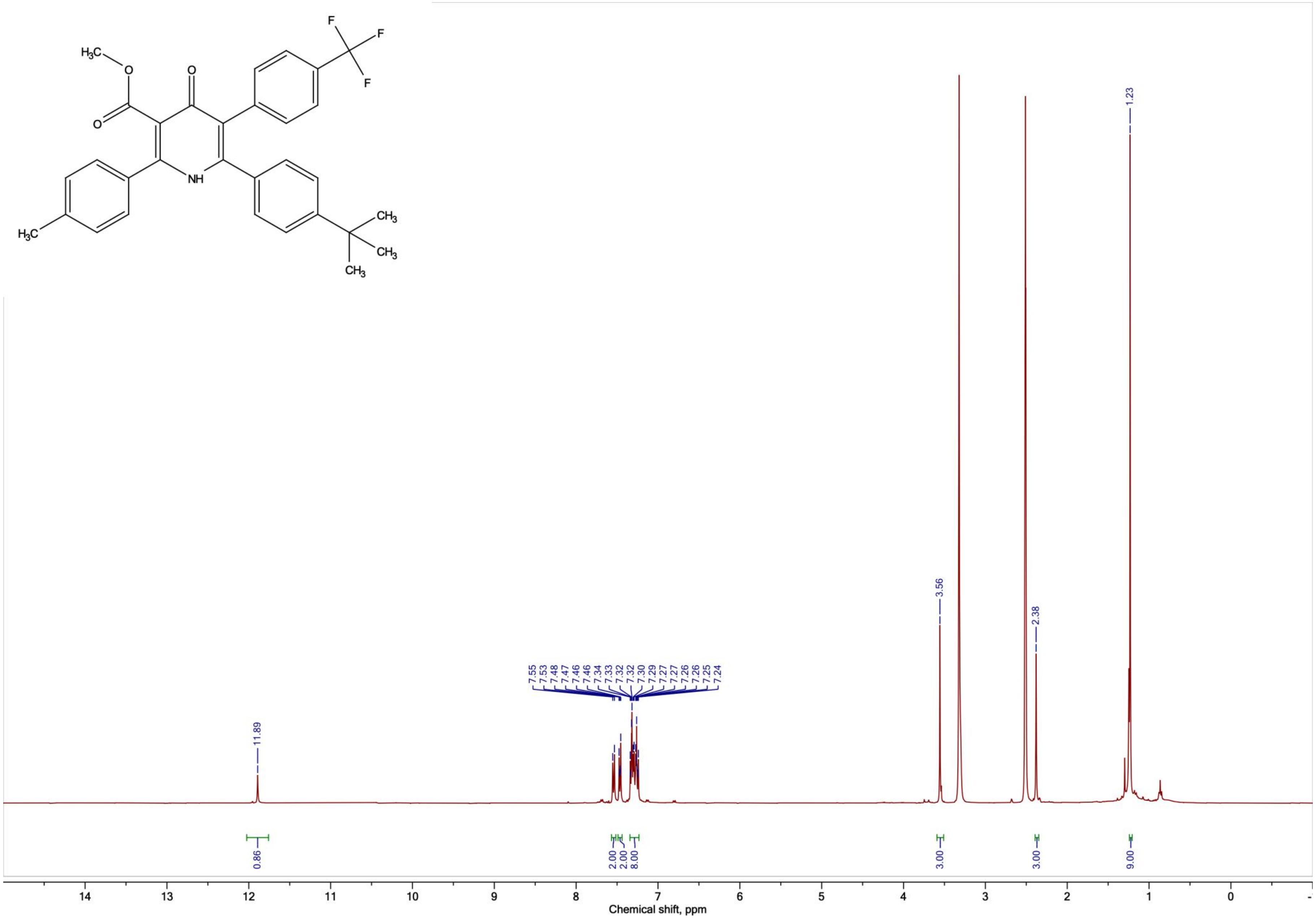

Methyl 6-(4-(*tert*-butyl)phenyl)-4-oxo-2-(*p*-tolyl)-5-(4-(trifluoromethyl)phenyl)-1,4-dihydropyridine-3-carboxylate (2o), $^{13}C\{^{1}H\}$ NMR, DMSO-d_6, 101 MHz

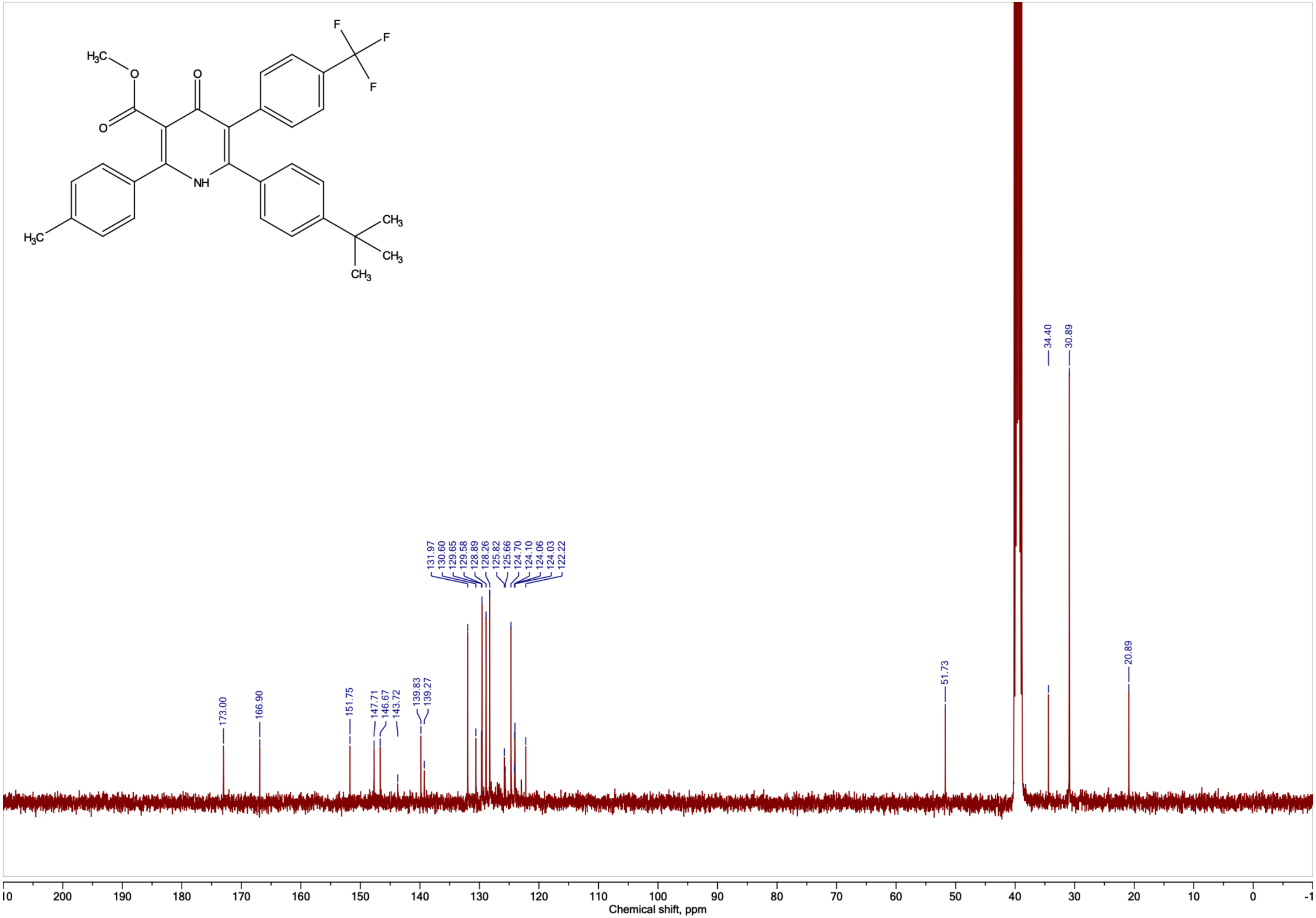

Methyl 6-(4-(*tert*-butyl)phenyl)-4-oxo-2-(*p*-tolyl)-5-(4-(trifluoromethyl)phenyl)-1,4-dihydropyridine-3-carboxylate (2o), DEPT, DMSO-d_6, 101 MHz

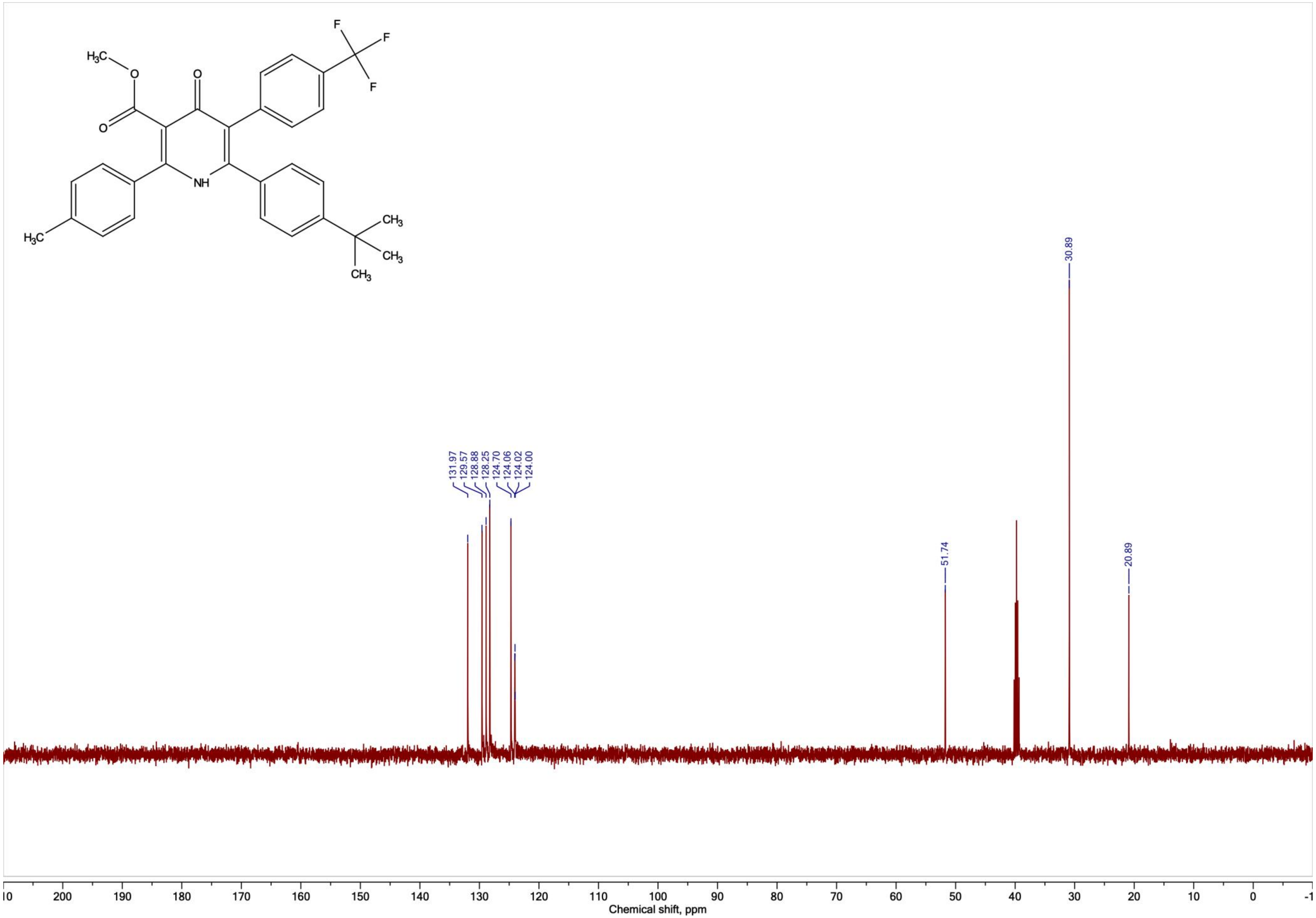

Methyl 6-(4-chlorophenyl)-4-oxo-5-phenyl-2-(*p*-tolyl)-1,4-dihydropyridine-3-carboxylate (2p), ^{1}H NMR, $CDCl_3$, 400 MHz

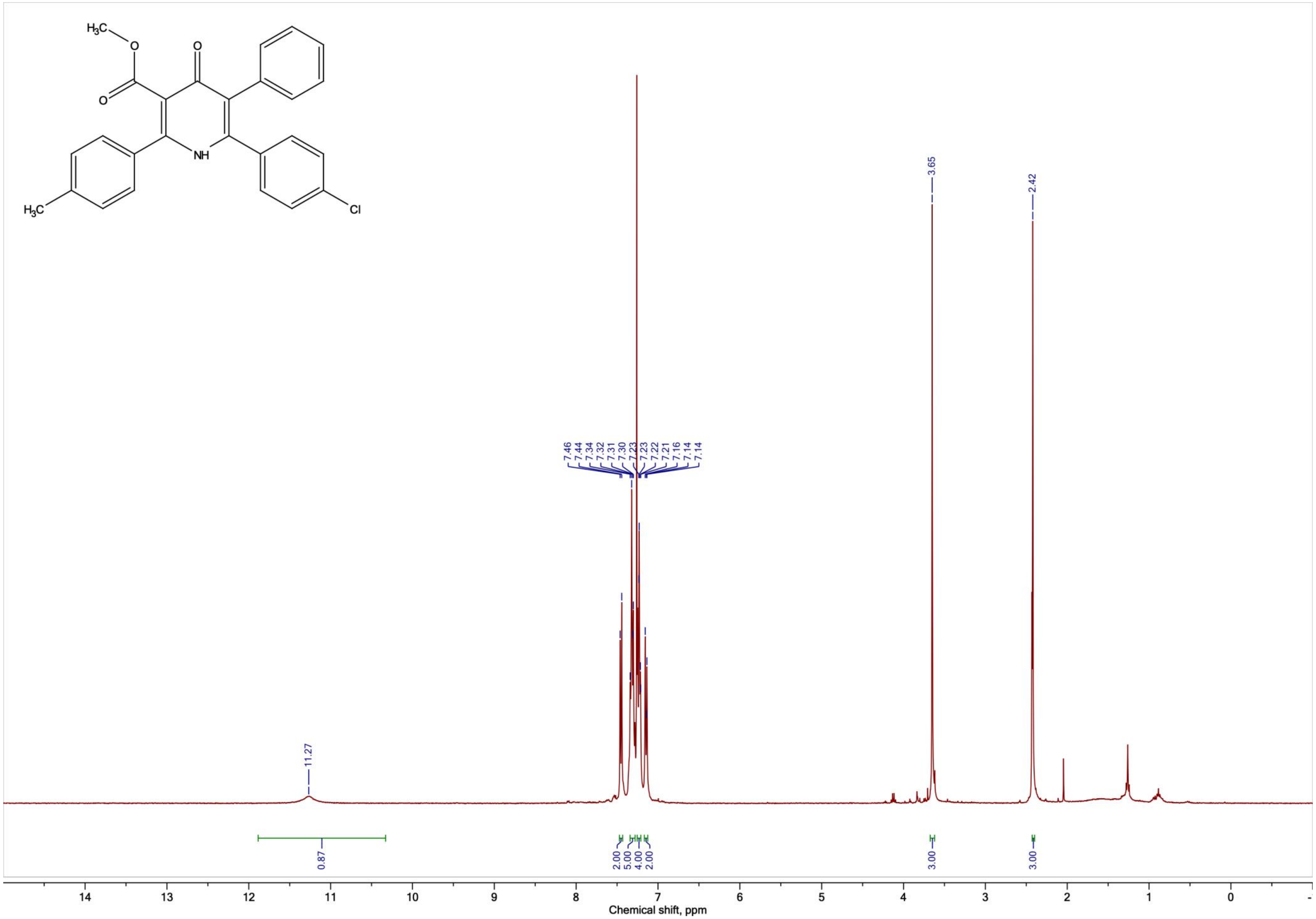

Methyl 6-(4-chlorophenyl)-4-oxo-5-phenyl-2-(*p*-tolyl)-1,4-dihydropyridine-3-carboxylate (2p), $^{13}C\{^{1}H\}$ NMR, $CDCl_3$, 101 MHz

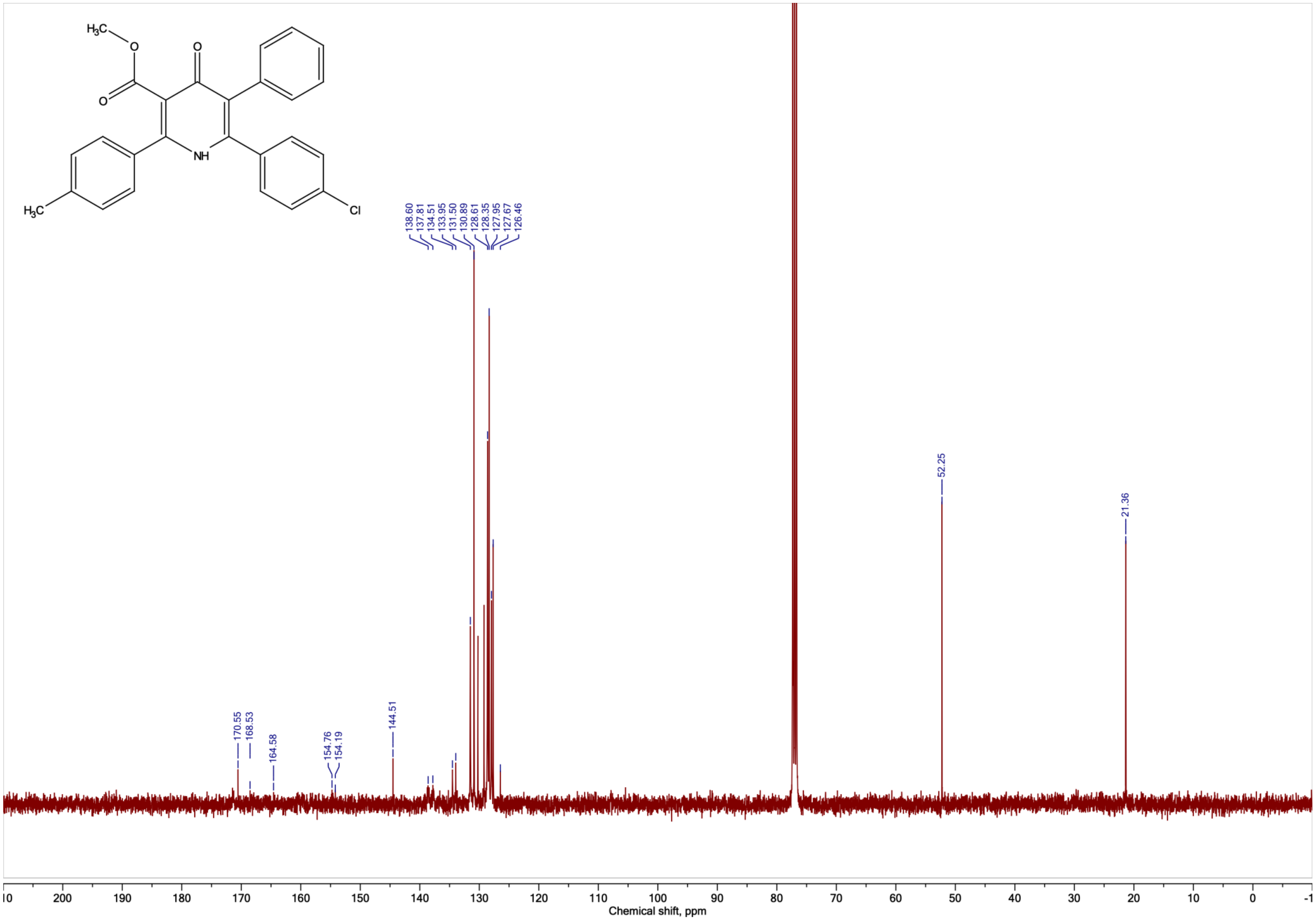

Methyl 6-(4-chlorophenyl)-4-oxo-5-phenyl-2-(*p*-tolyl)-1,4-dihydropyridine-3-carboxylate (2p), DEPT, $CDCl_3$, 101 MHz

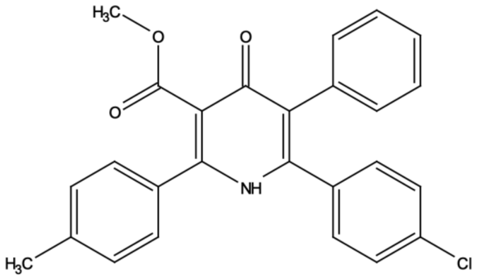

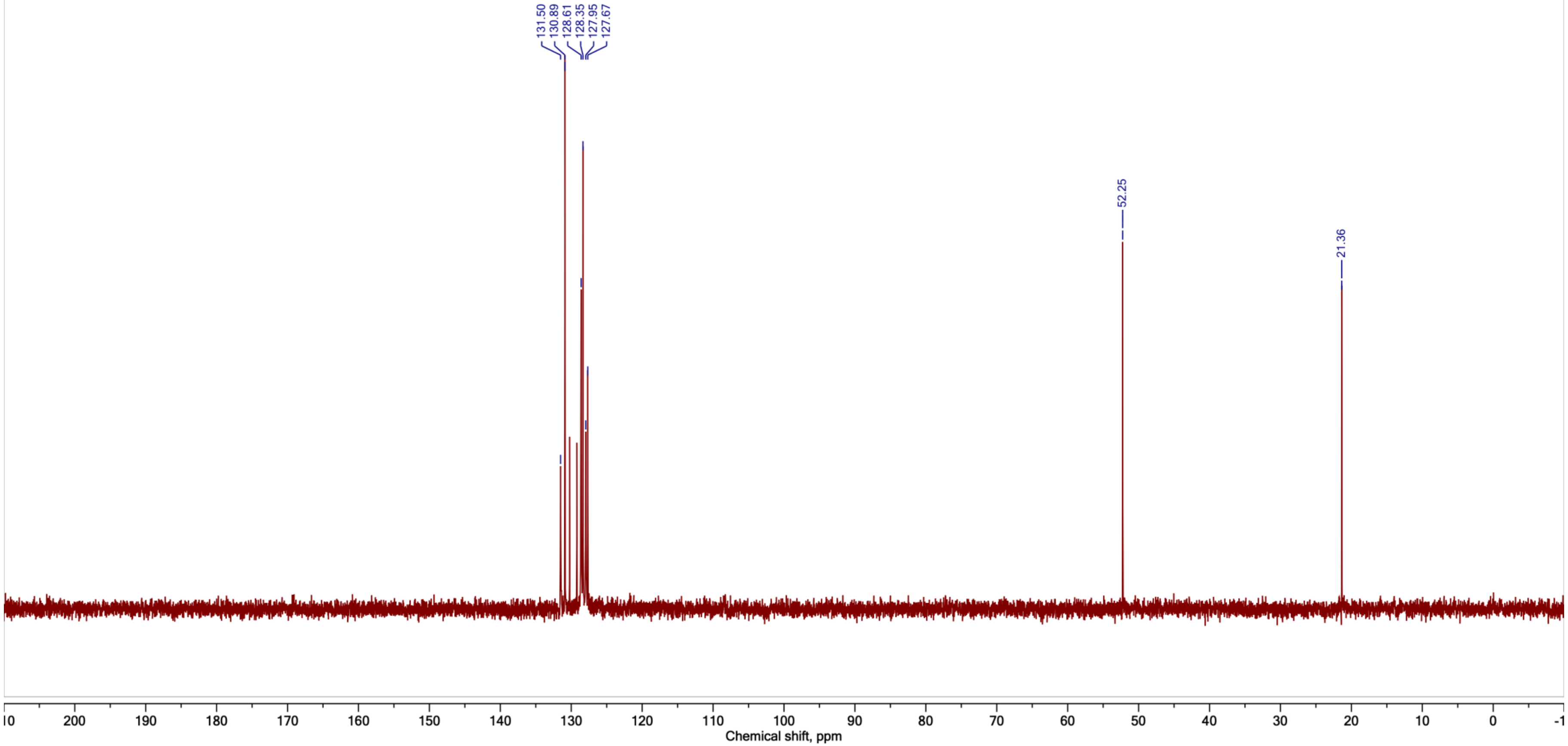

Methyl 6-(4-chlorophenyl)-5-(4-methoxyphenyl)-4-oxo-2-(*p*-tolyl)-1,4-dihydropyridine-3-carboxylate (2q), ^{1}H NMR, $CDCl_3$, 400 MHz

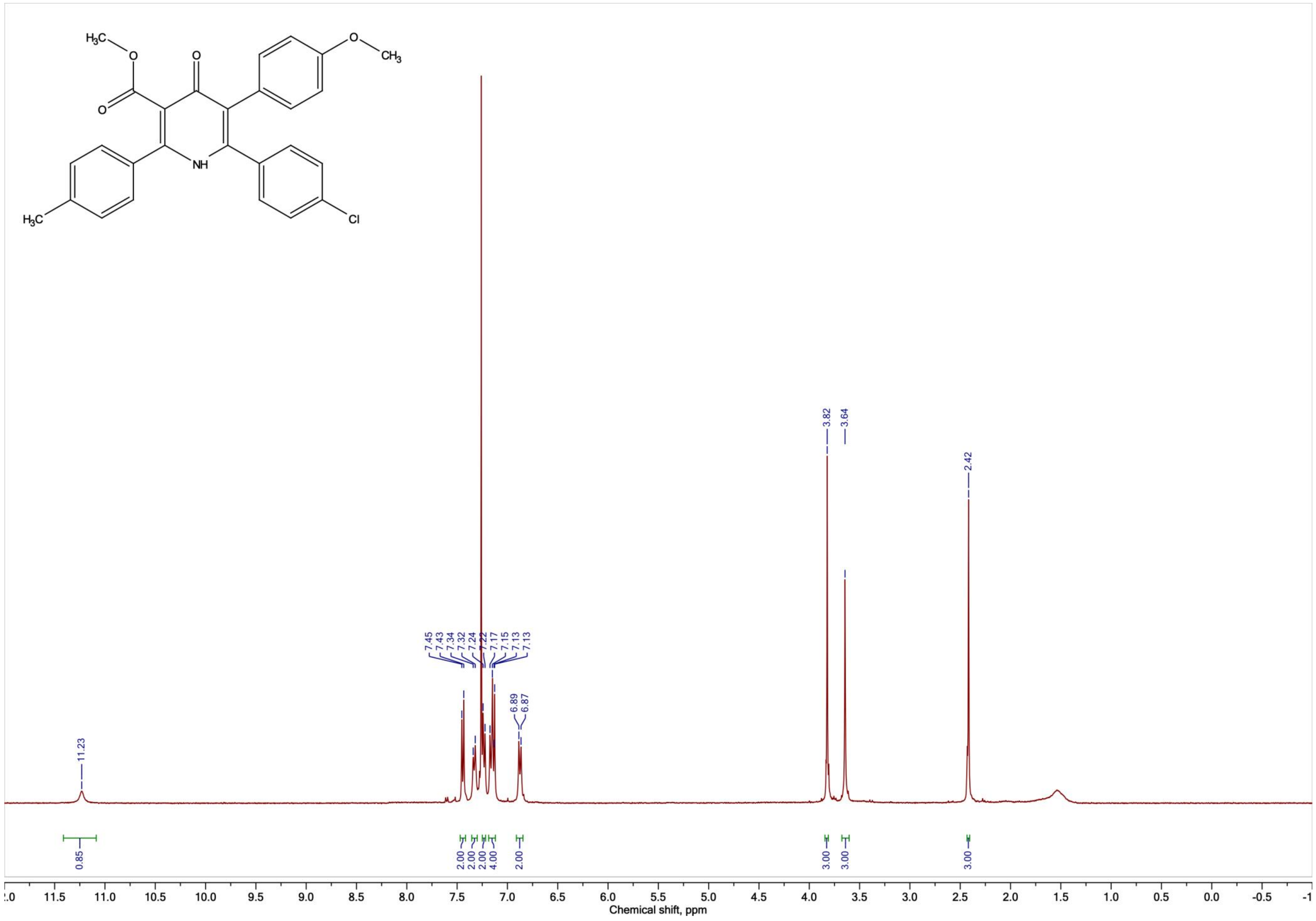

Methyl 6-(4-chlorophenyl)-5-(4-methoxyphenyl)-4-oxo-2-(*p*-tolyl)-1,4-dihydropyridine-3-carboxylate (2q), $^{13}C\{^1H\}$ NMR, $CDCl_3$, 101 MHz

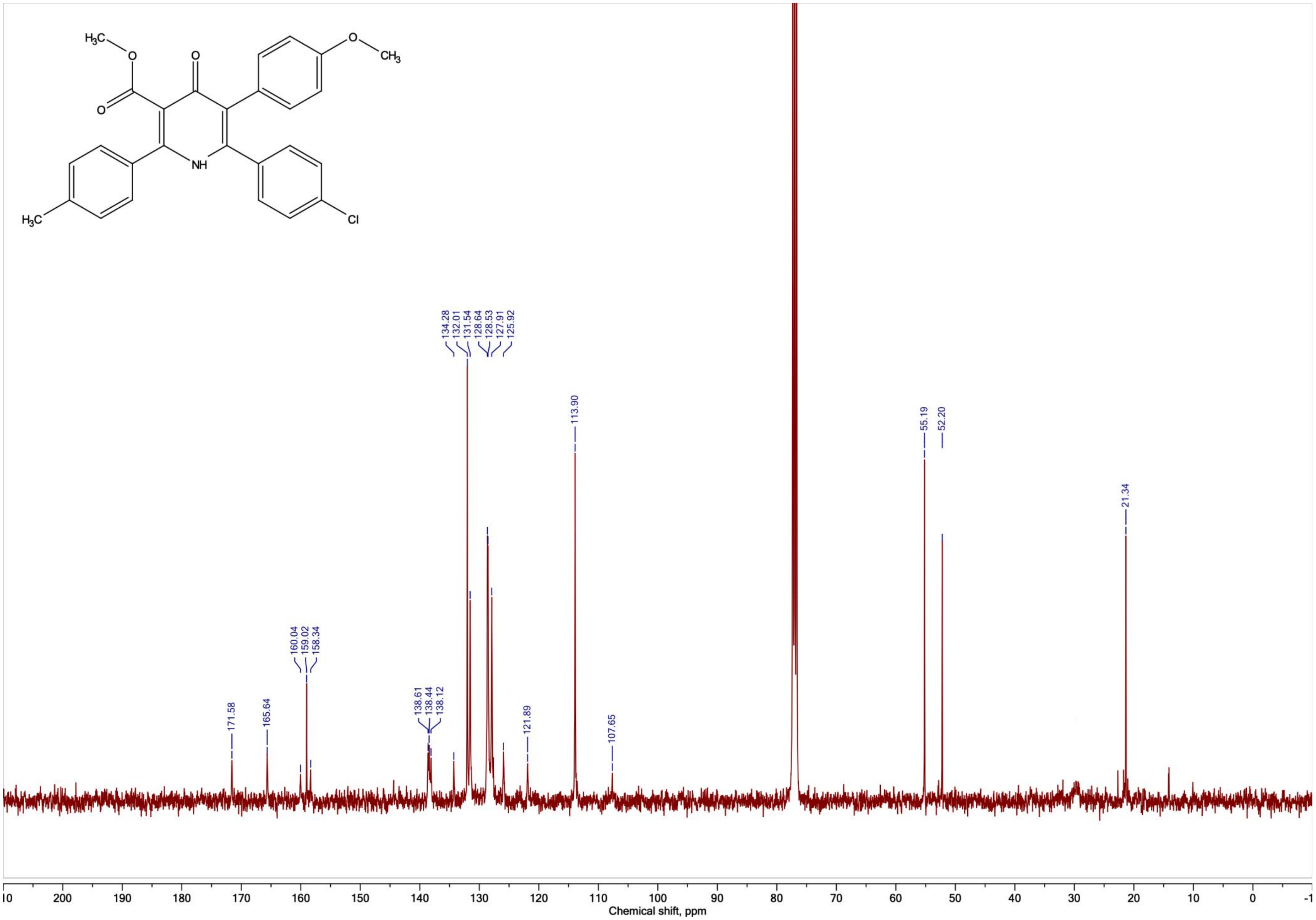

Methyl 6-(4-chlorophenyl)-5-(4-methoxyphenyl)-4-oxo-2-(*p*-tolyl)-1,4-dihydropyridine-3-carboxylate (2q), DEPT, $CDCl_3$, 101 MHz

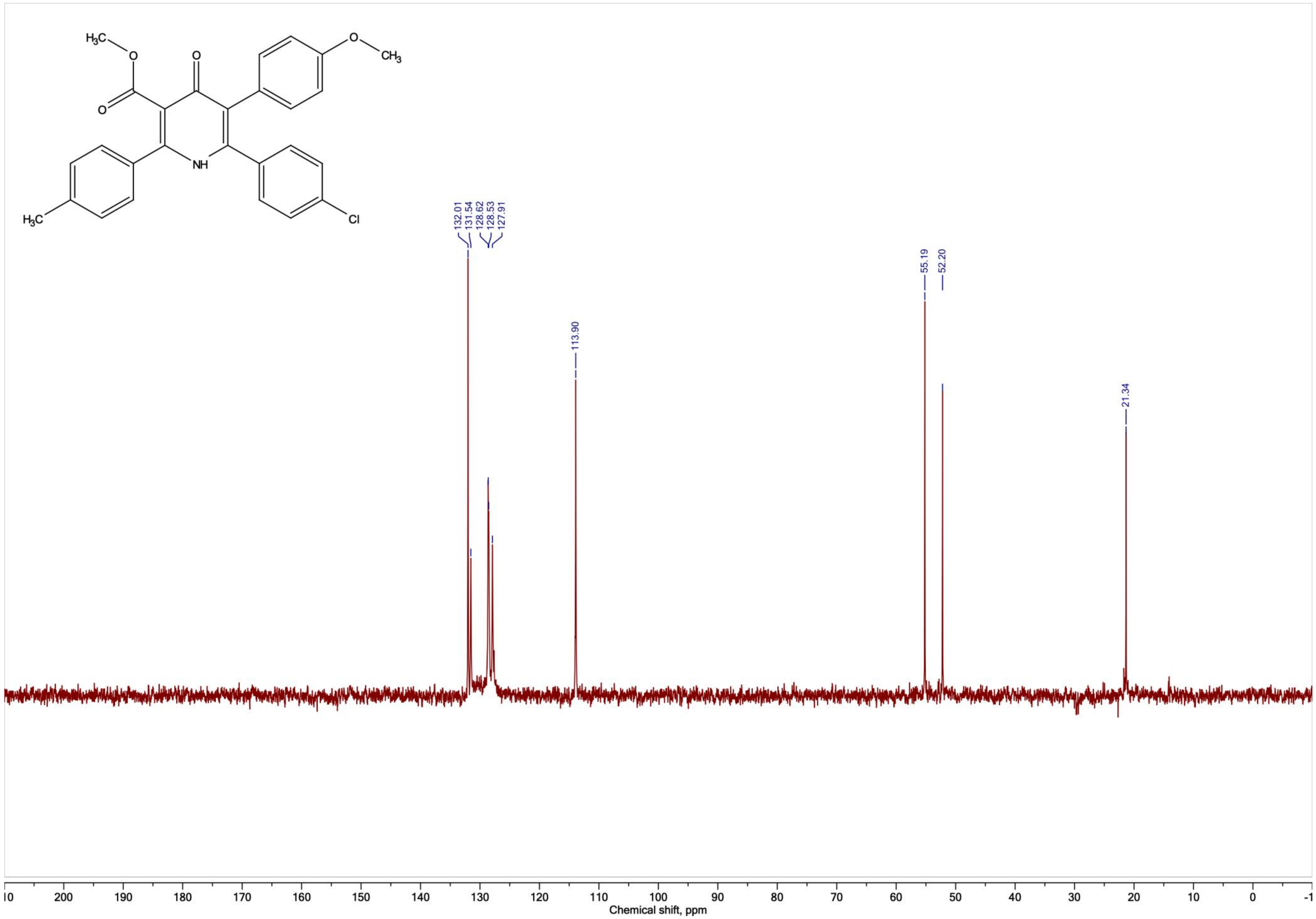

Methyl 2-(4-bromophenyl)-6-(4'-fluoro-[1,1'-biphenyl]-4-yl)-5-(4-fluorophenyl)-4-oxo-1,4-dihydropyridine-3-carboxylate (2s), ^1H NMR, $CDCl_3$, 400 MHz

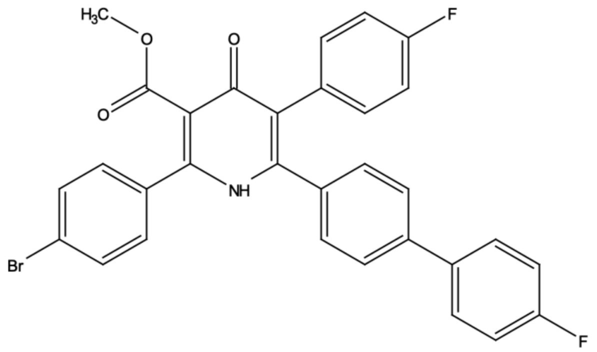

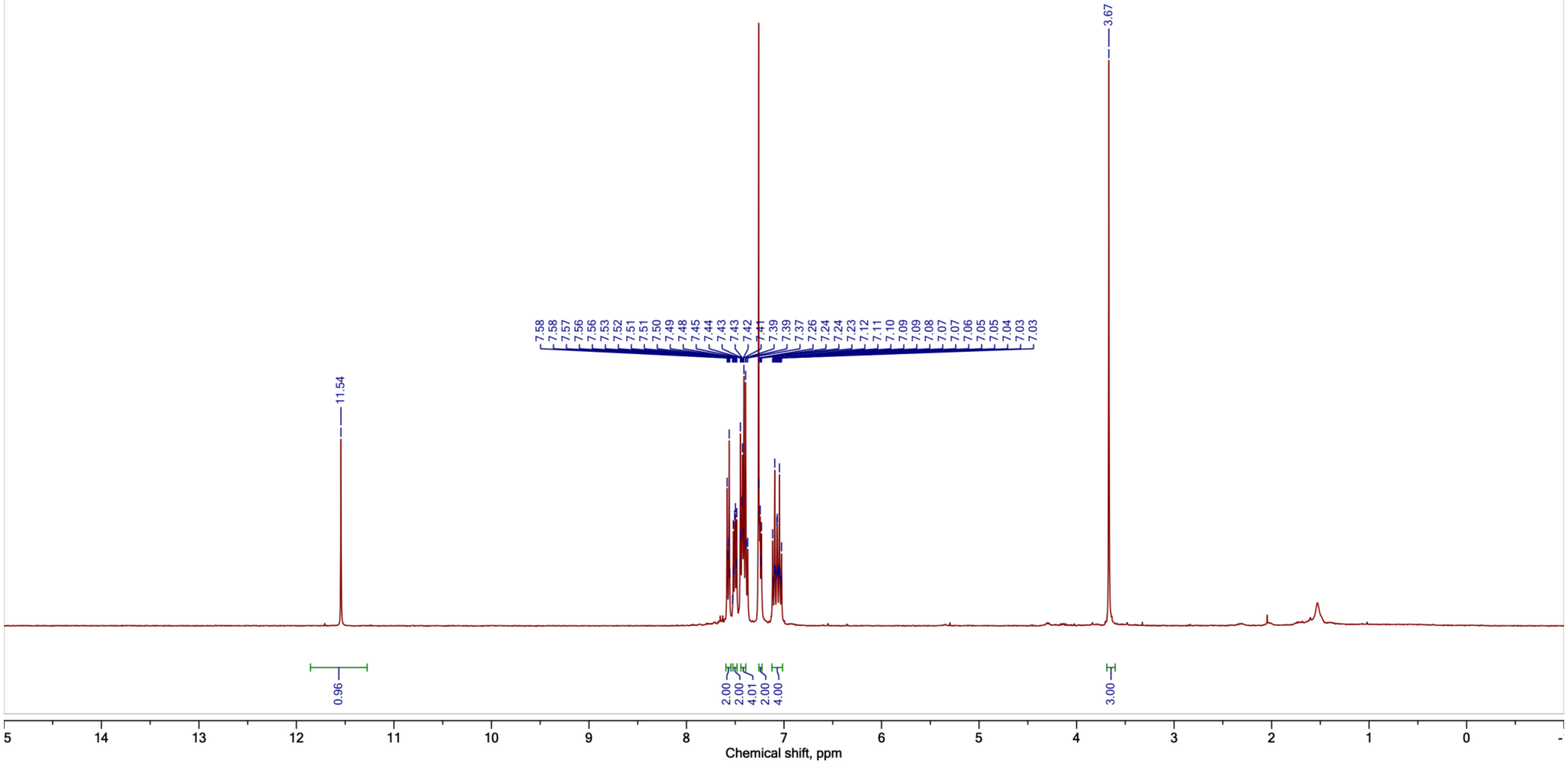

Methyl 2-(4-bromophenyl)-6-(4'-fluoro-[1,1'-biphenyl]-4-yl)-5-(4-fluorophenyl)-4-oxo-1,4-dihydropyridine-3-carboxylate (2s), $^{13}C\{^1H\}$ NMR, $CDCl_3$, 101 MHz

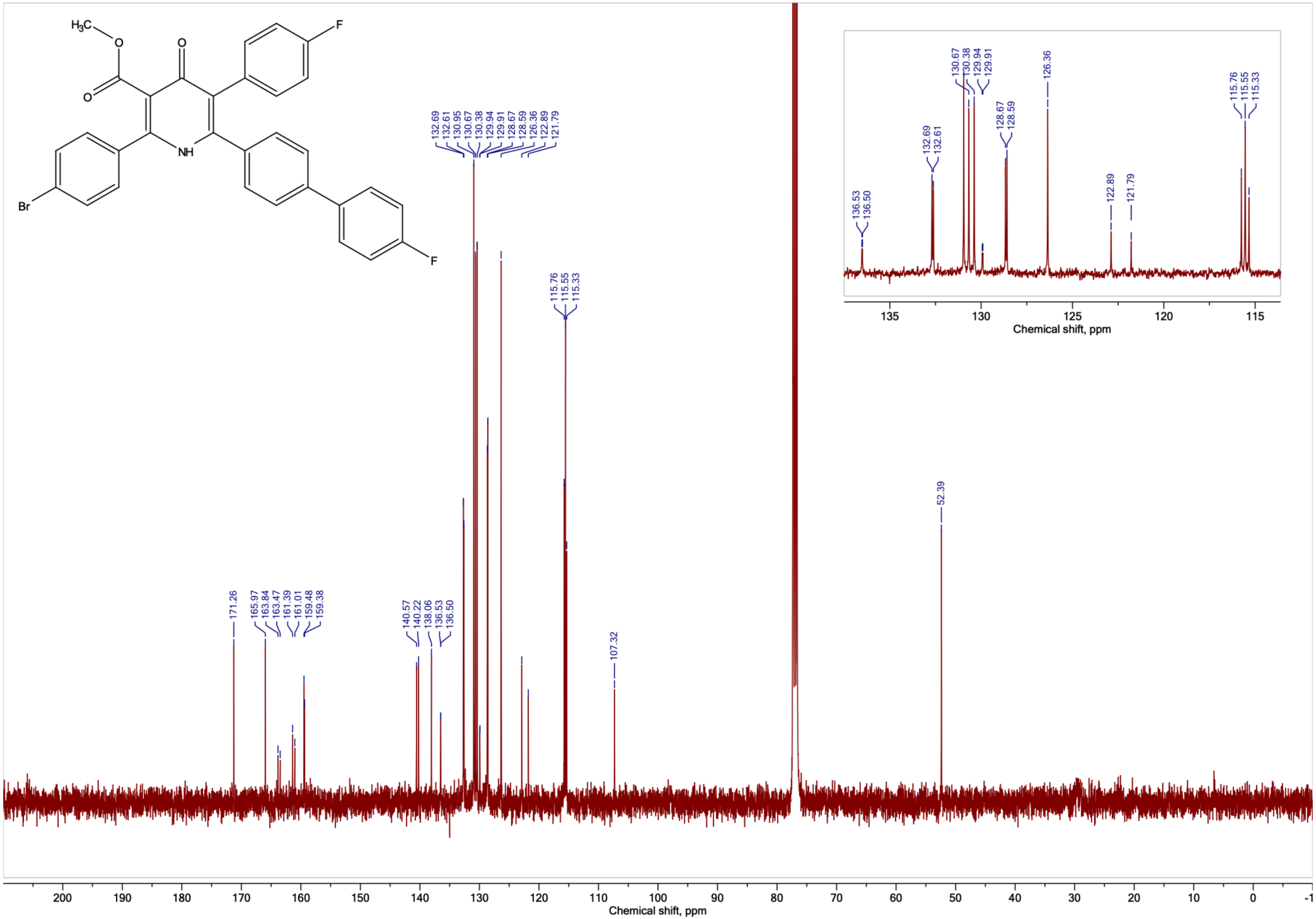

Methyl 2-(4-bromophenyl)-6-(4'-fluoro-[1,1'-biphenyl]-4-yl)-5-(4-fluorophenyl)-4-oxo-1,4-dihydropyridine-3-carboxylate (2s), DEPT, $CDCl_3$, 101 MHz

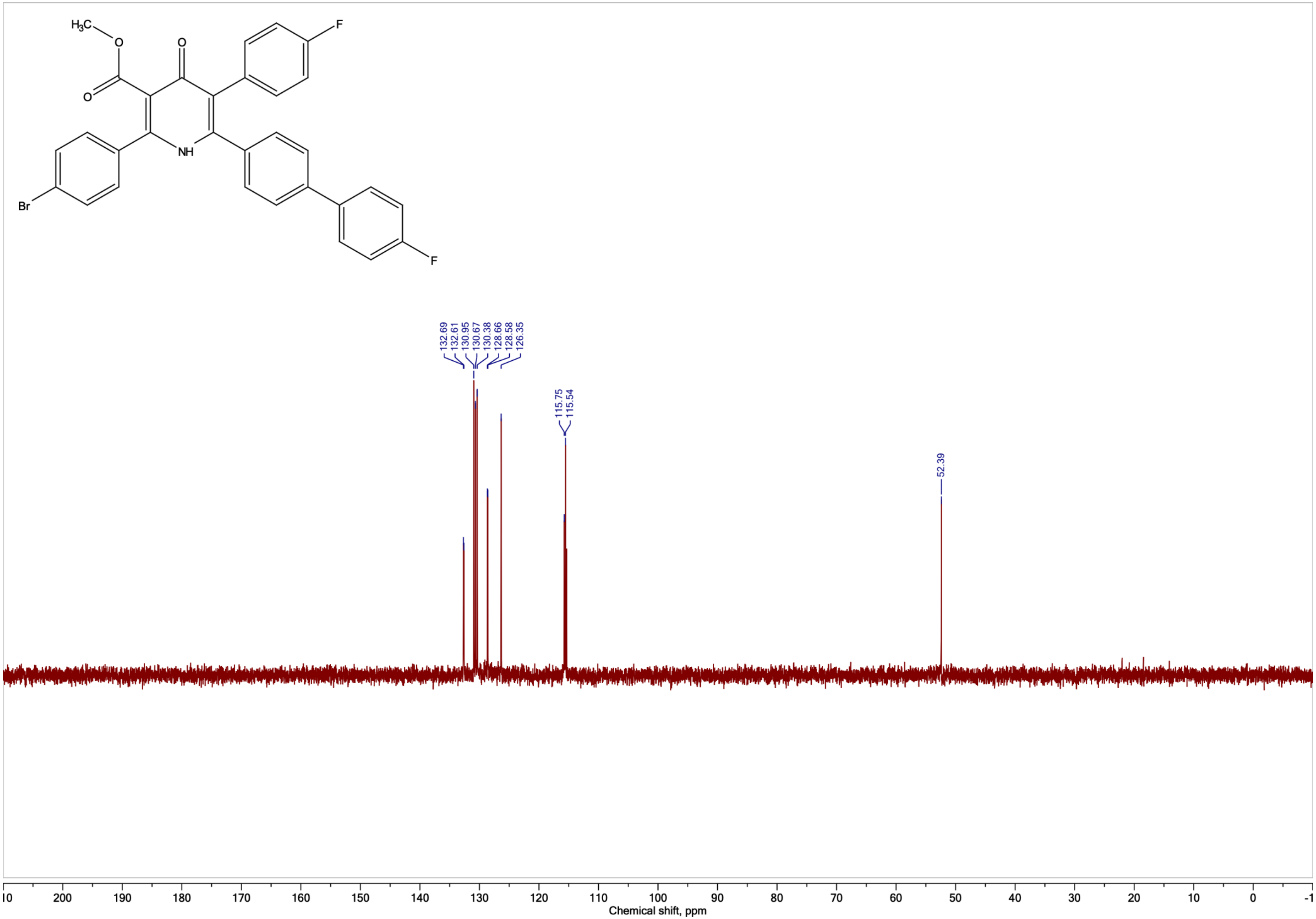

Methyl 5-amino-5-(4-chlorophenyl)-3-oxopent-4-enoate (6), ^{1}H NMR, $CDCl_3$, 400 MHz

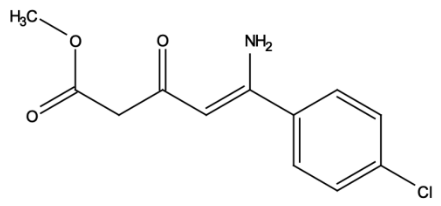

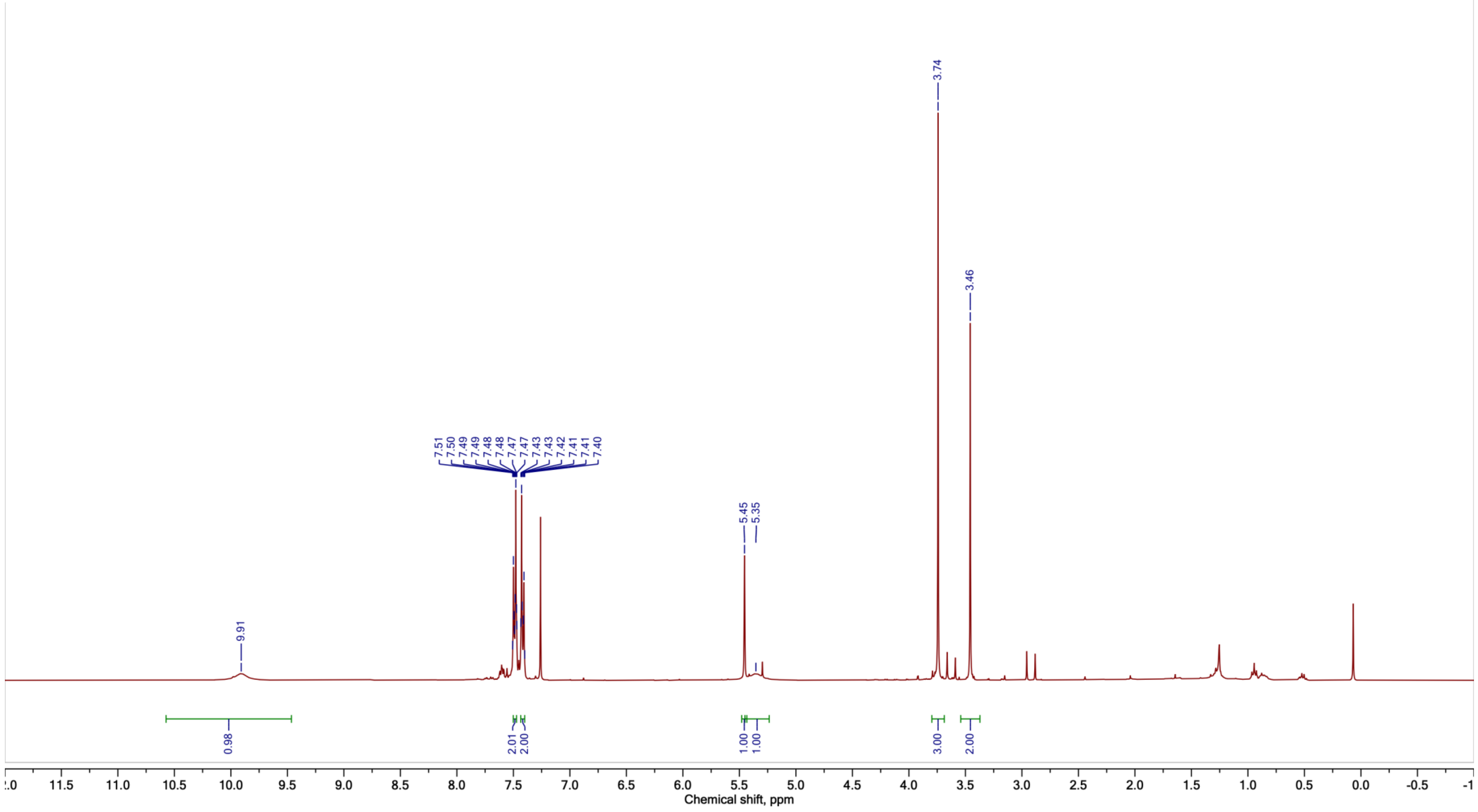

Methyl 5-amino-5-(4-chlorophenyl)-3-oxopent-4-enoate (6), $^{13}C\{^1H\}$ NMR, $CDCl_3$, 101 MHz

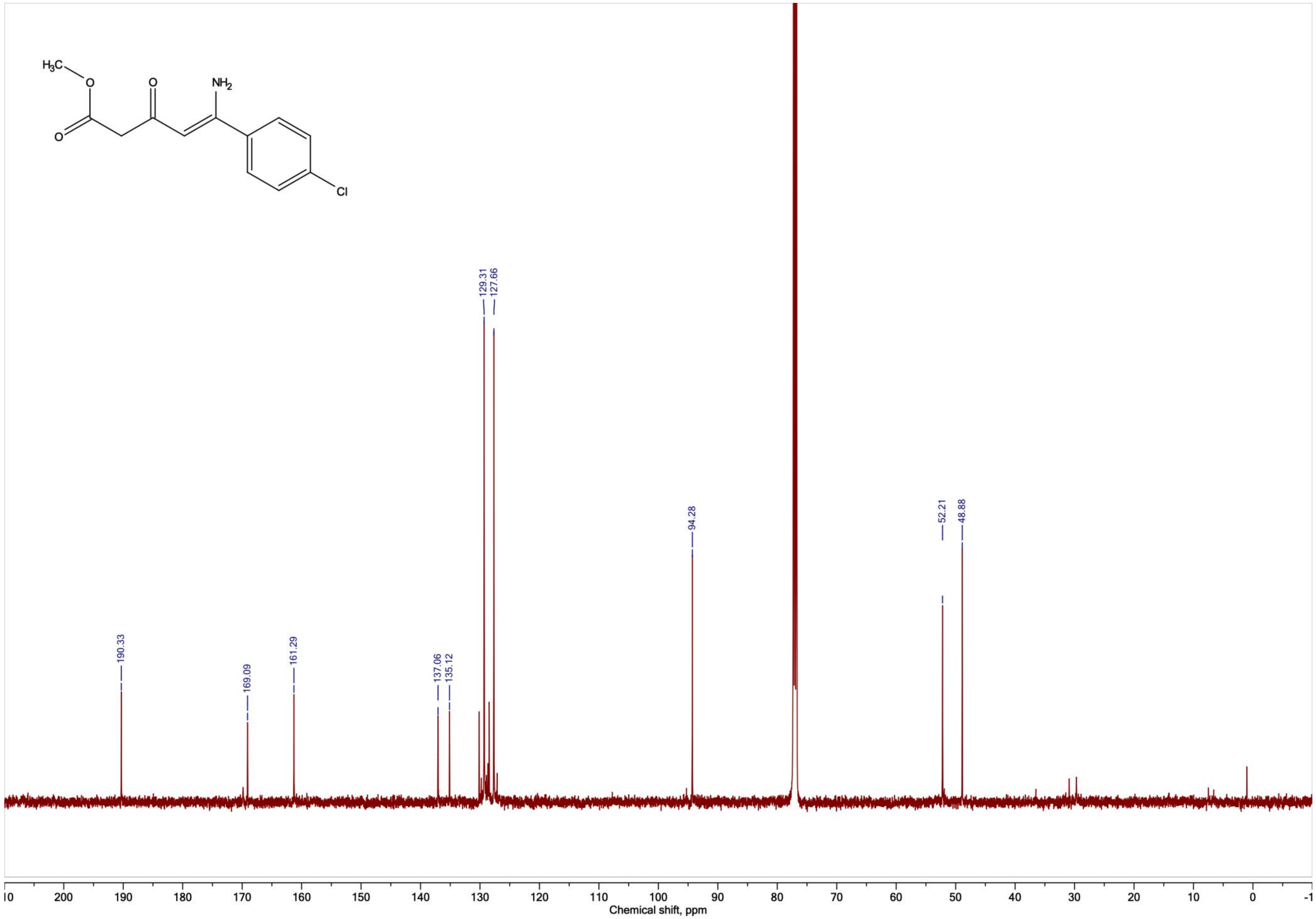

Methyl 5-amino-5-(4-chlorophenyl)-3-oxopent-4-enoate (6), DEPT, $CDCl_3$, 101 MHz

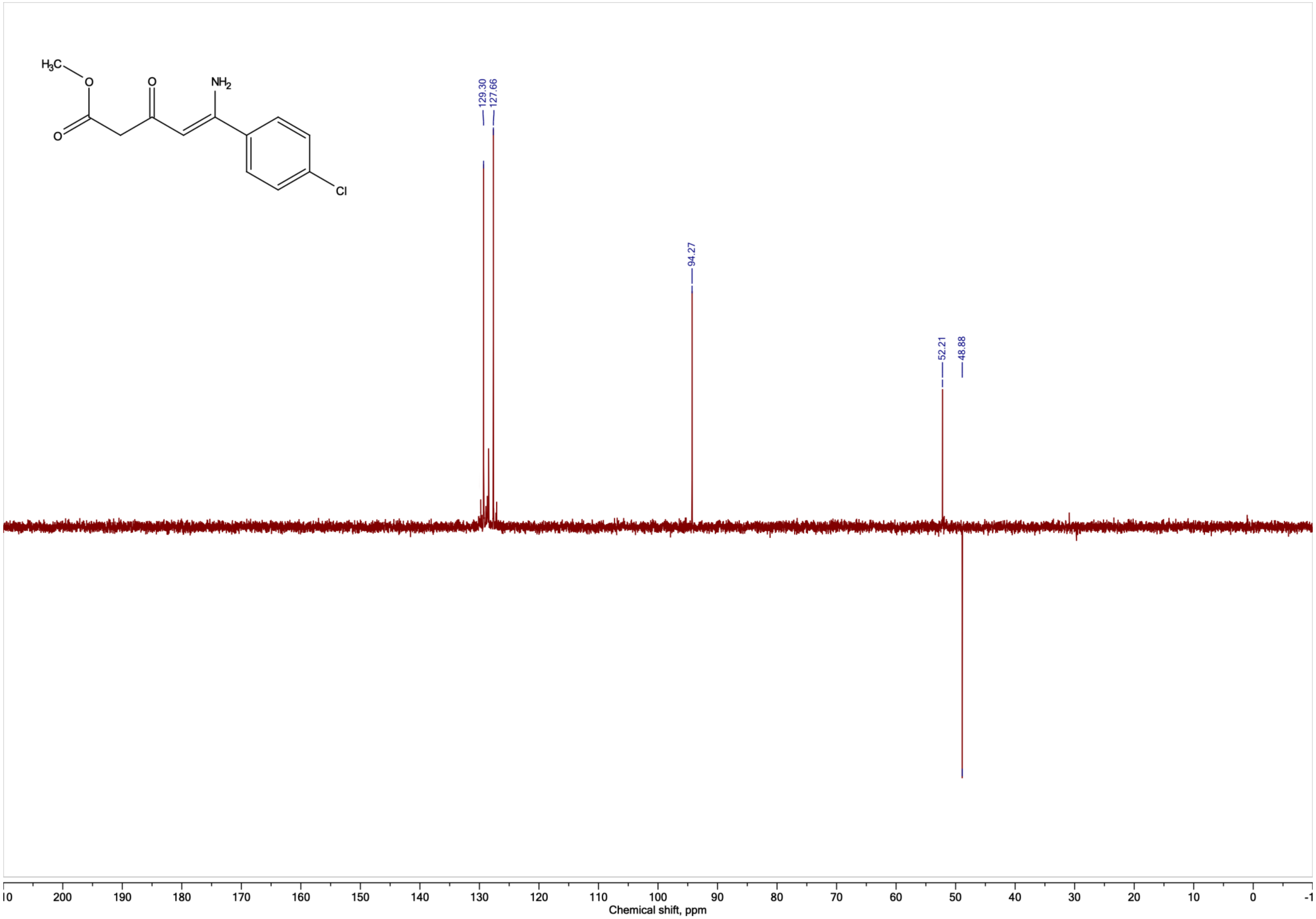

6-(4-Bromophenyl)-2-(4-(*tert*-butyl)phenyl)-3-(hydroxymethyl)pyridin-4(*1H*)-one (14), ^{1}H NMR, DMSO-d$_6$, 400 MHz

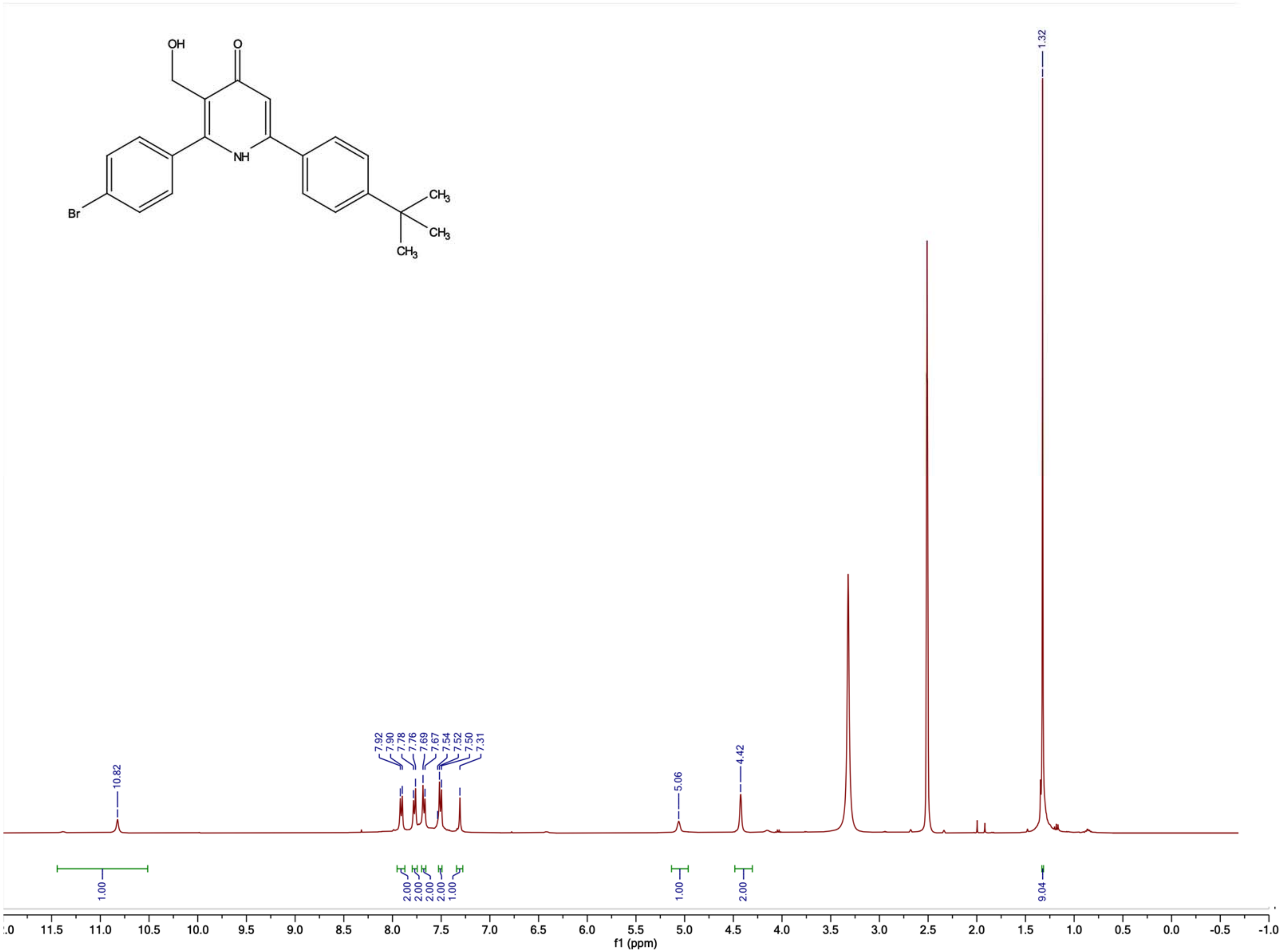

6-(4-Bromophenyl)-2-(4-(*tert*-butyl)phenyl)-3-(hydroxymethyl)pyridin-4(*1H*)-one (14), $^{13}C\{^{1}H\}$ NMR, DMSO-d$_6$, 101 MHz

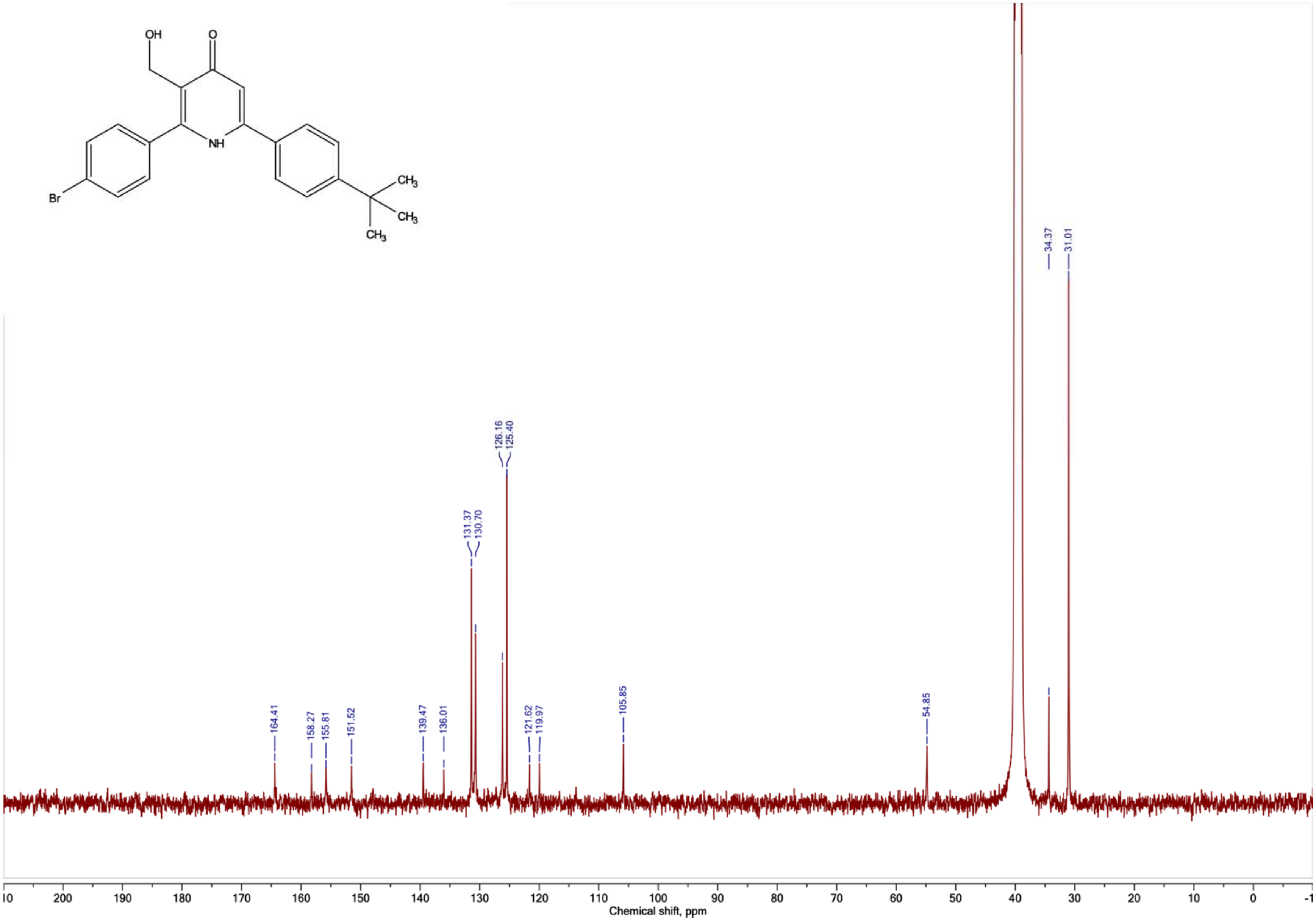

6-(4-Bromophenyl)-2-(4-(*tert*-butyl)phenyl)-3-(hydroxymethyl)pyridin-4(*1H*)-one (14), DEPT, DMSO-d$_6$, 101 MHz

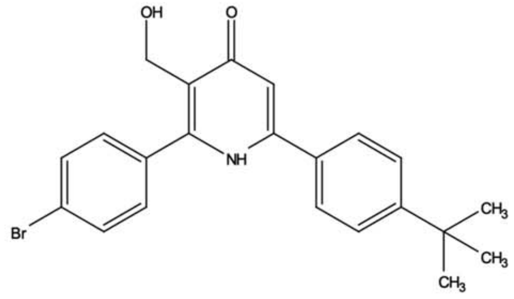

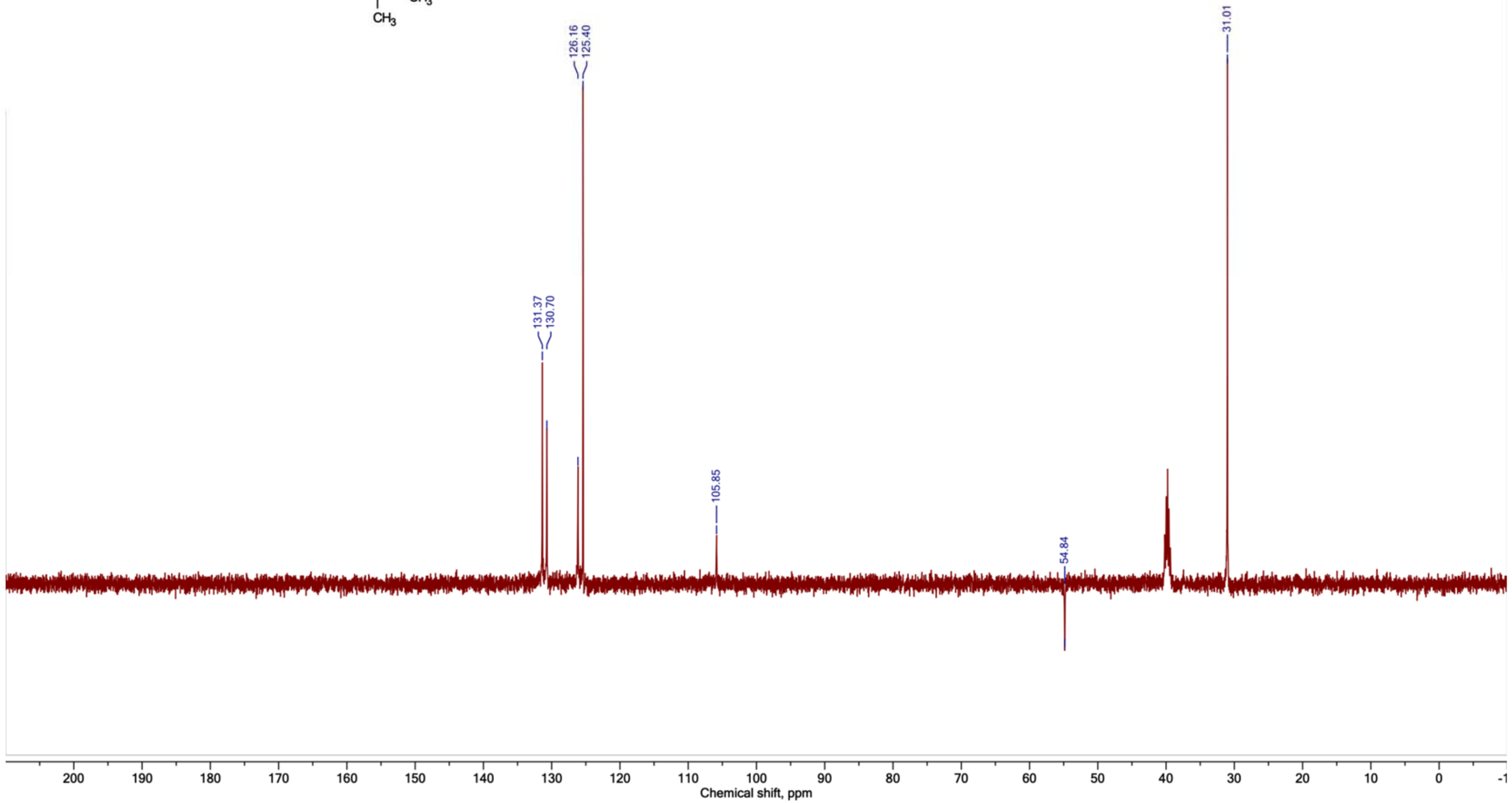

Methyl 4-bromo-6-(4-fluorophenyl)-2-phenylnicotinate (15), ^{1}H NMR, $CDCl_3$, 400 MHz

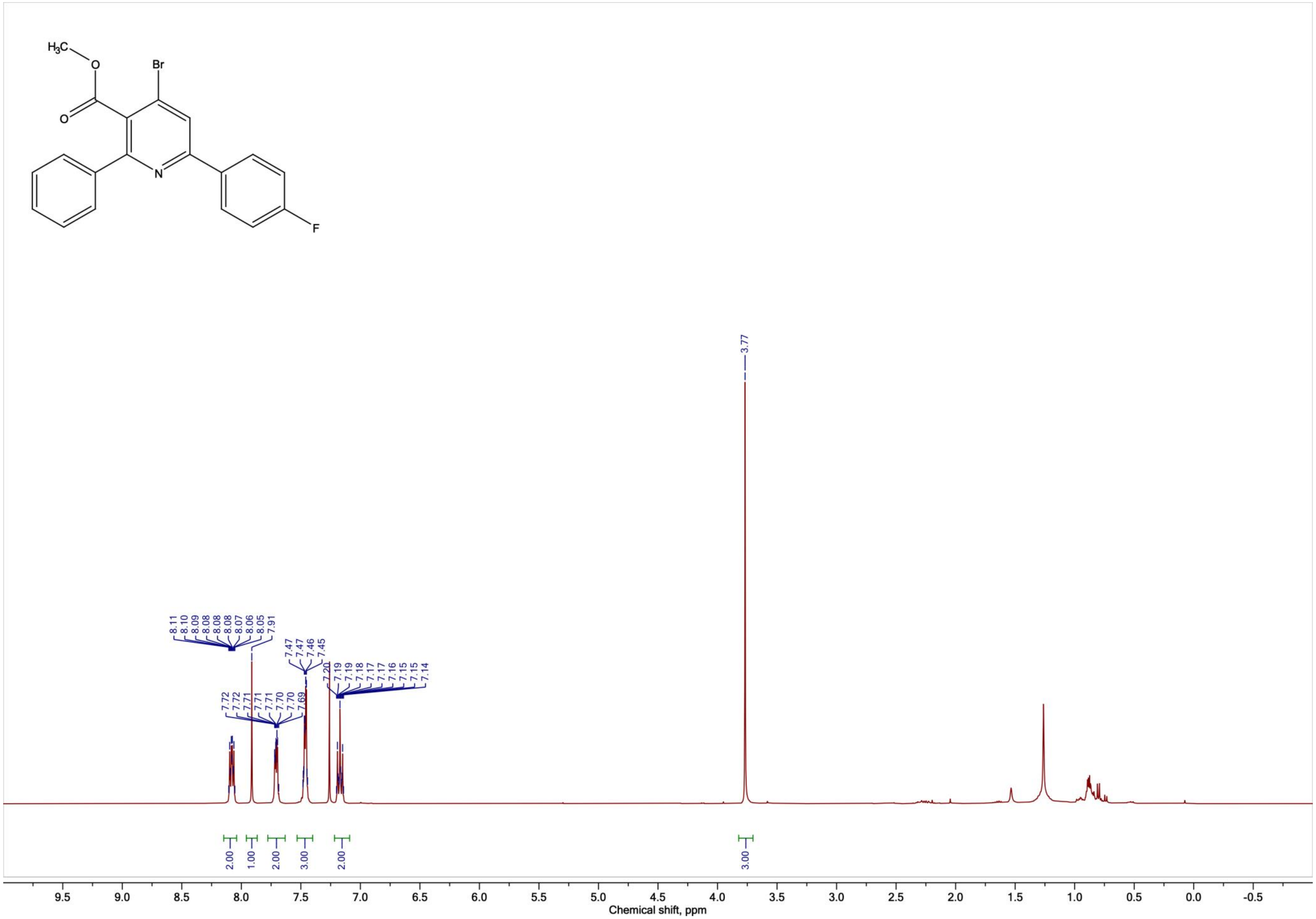

Methyl 4-bromo-6-(4-fluorophenyl)-2-phenylnicotinate (15), $^{13}C\{^1H\}$ NMR, $CDCl_3$, 101 MHz

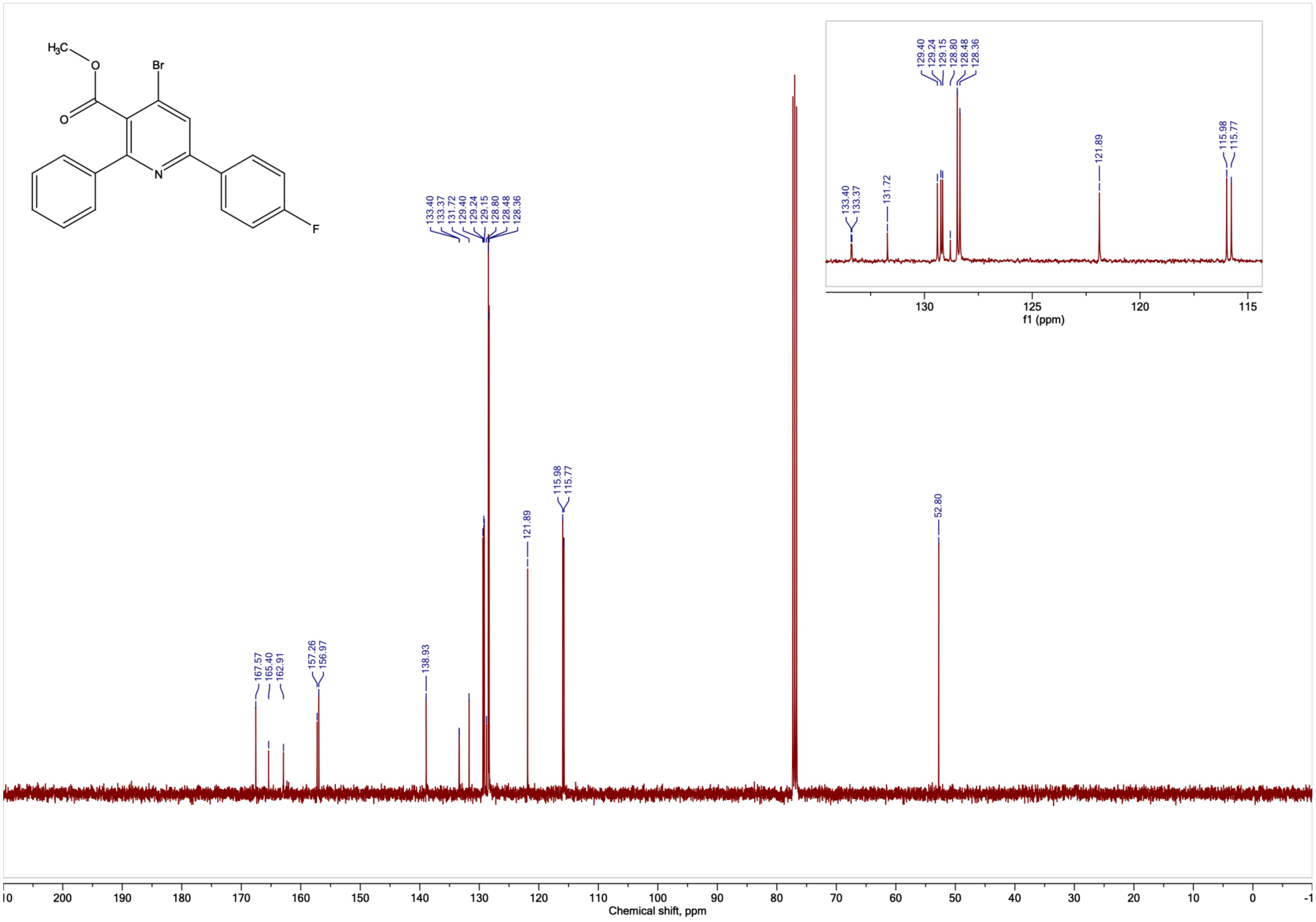

Methyl 4-bromo-6-(4-fluorophenyl)-2-phenylnicotinate (15), DEPT, $CDCl_3$, 101 MHz

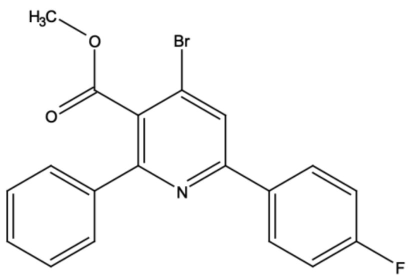

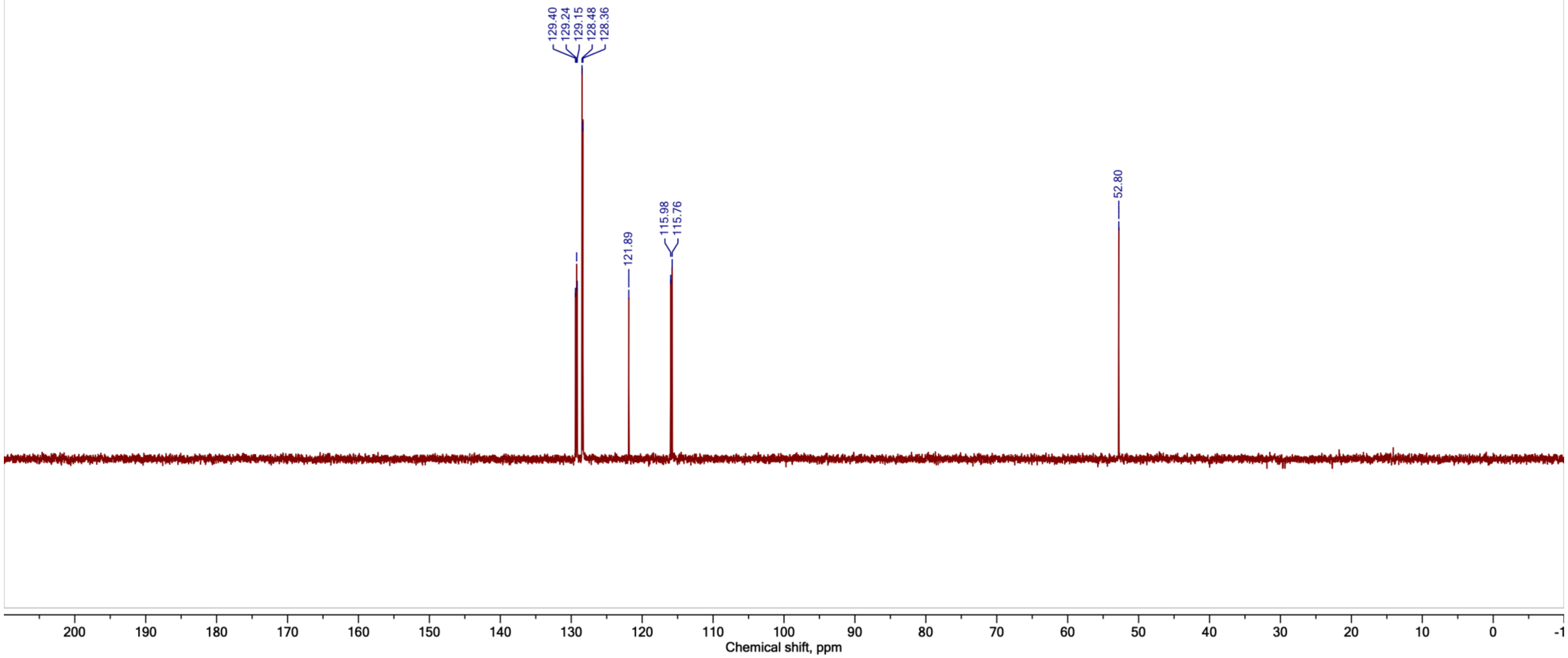

Methyl 4-(4-chlorophenyl)-6-(4-fluorophenyl)-2-phenylnicotinate (16), ^{1}H NMR, $CDCl_3$, 400 MHz

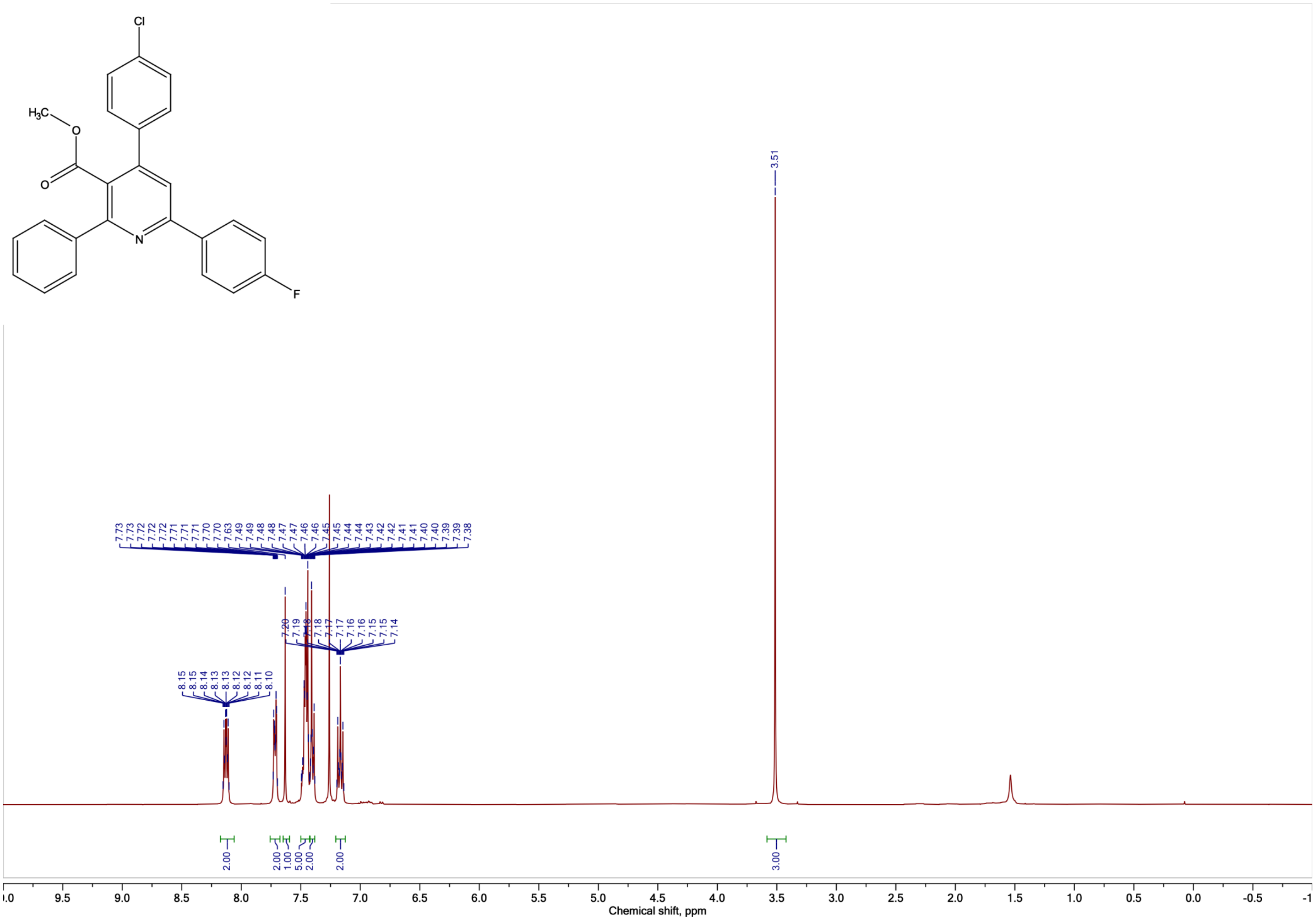

Methyl 4-(4-chlorophenyl)-6-(4-fluorophenyl)-2-phenylnicotinate (16), $^{13}C\{^{1}H\}$ NMR, $CDCl_3$, 101 MHz

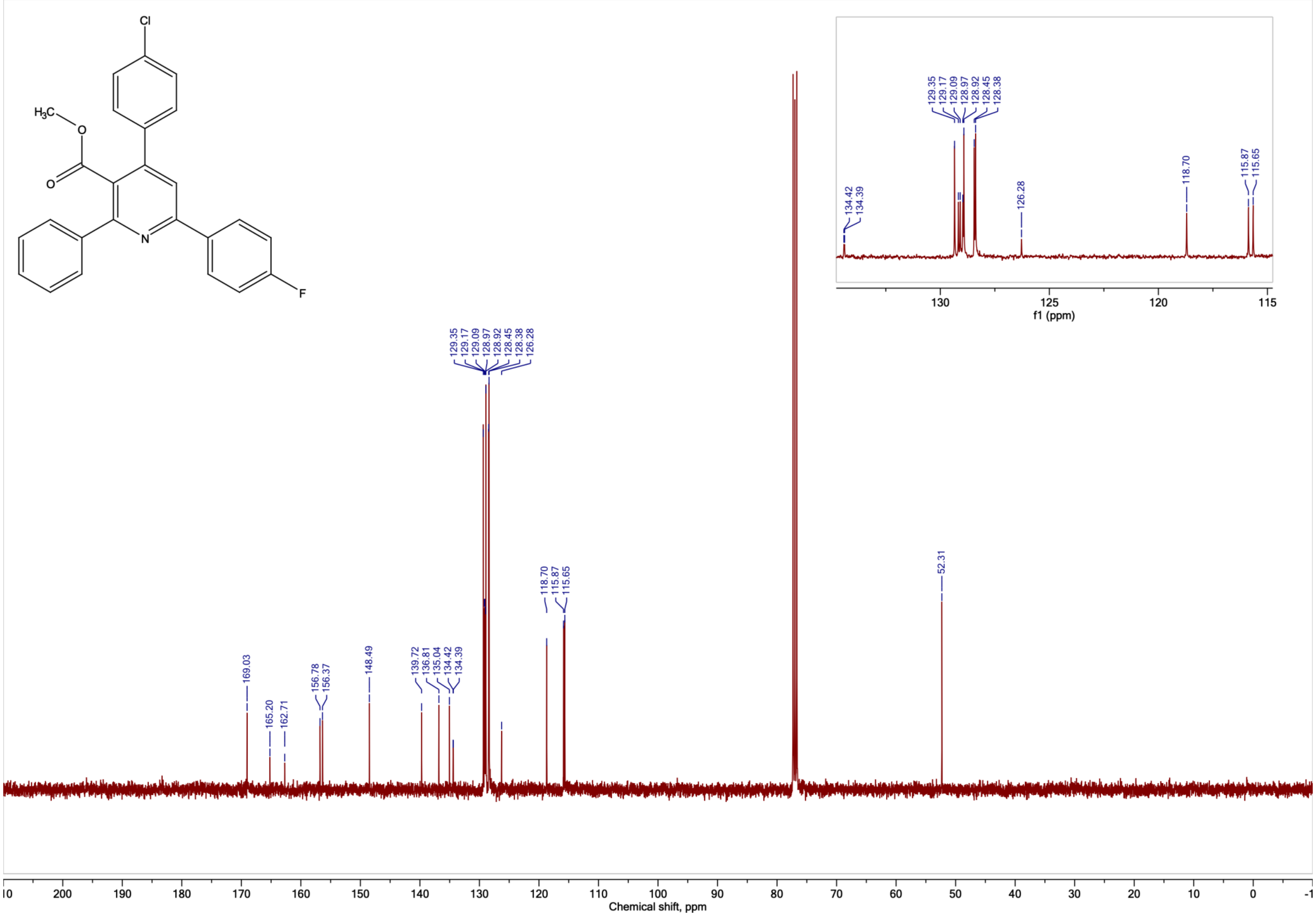

Methyl 4-(4-chlorophenyl)-6-(4-fluorophenyl)-2-phenylnicotinate (16), DEPT, $CDCl_3$, 101 MHz

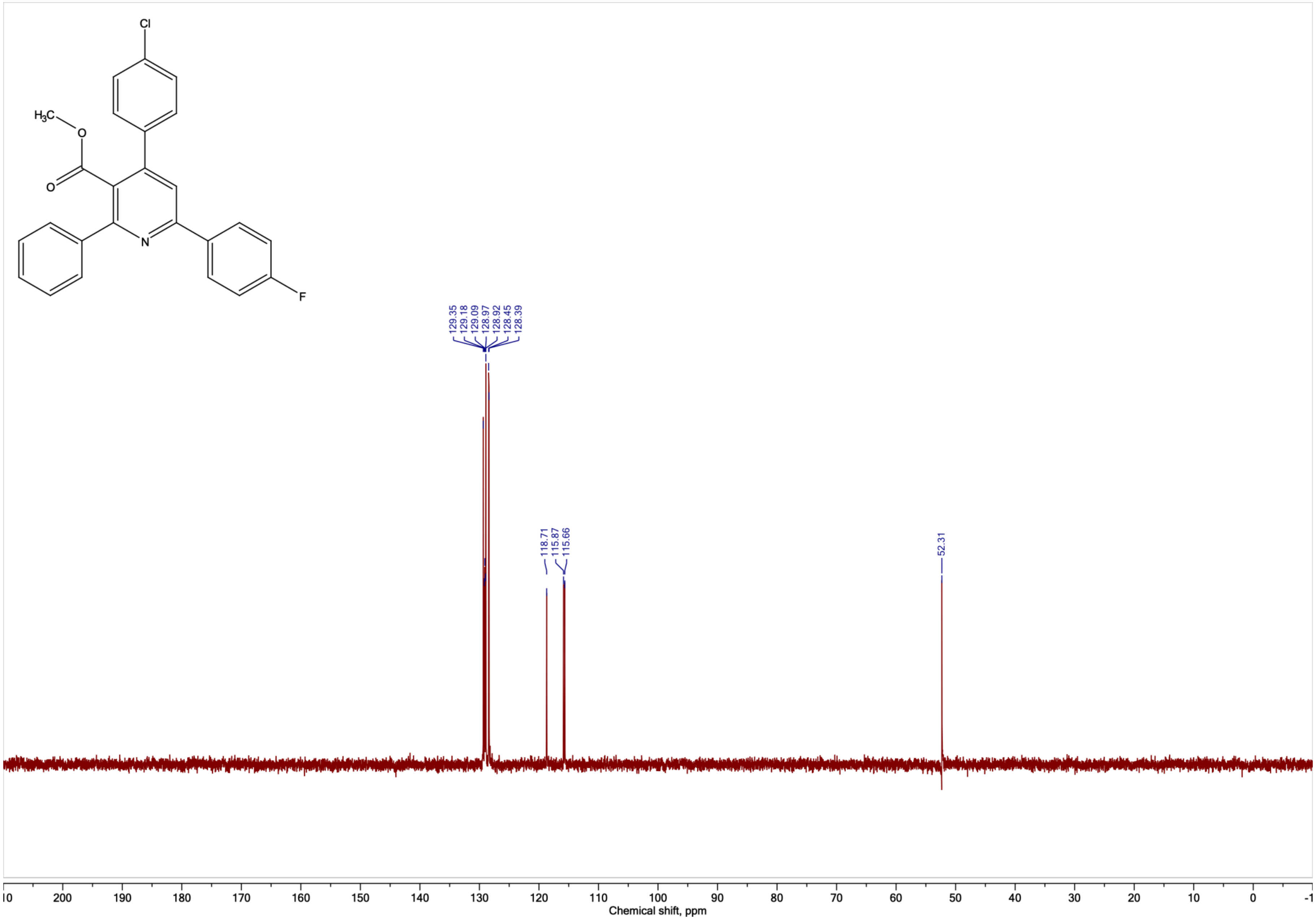

Methyl 1-(4-chlorobenzyl)-4-oxo-6-phenyl-5-(thiophen-3-yl)-2-(*p*-tolyl)-1,4-dihydropyridine-3-carboxylate (17), ^{1}H NMR, $CDCl_3$, 400 MHz

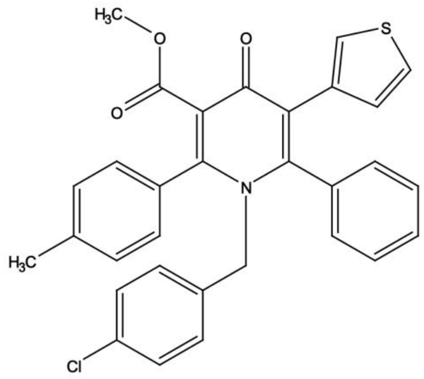

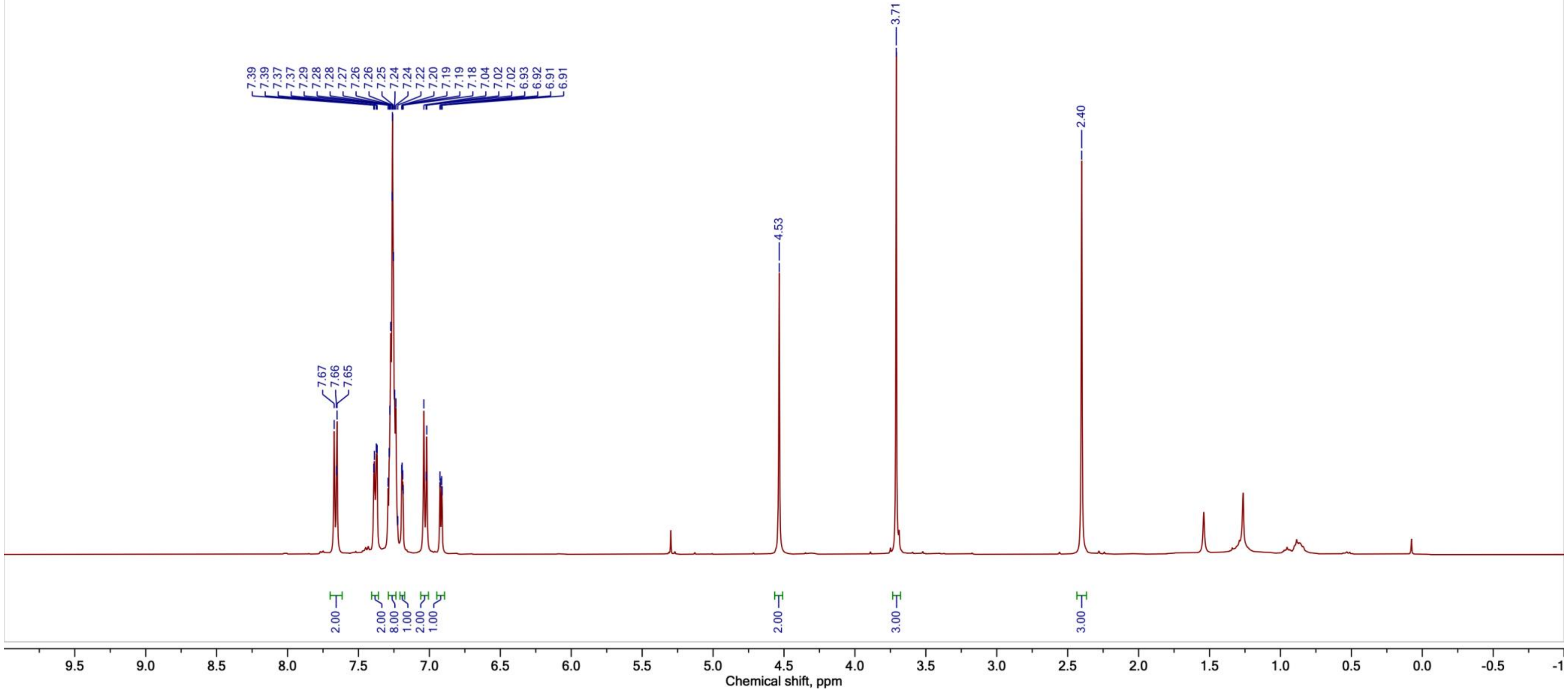

Methyl 1-(4-chlorobenzyl)-4-oxo-6-phenyl-5-(thiophen-3-yl)-2-(*p*-tolyl)-1,4-dihydropyridine-3-carboxylate (17), $^{13}C\{^{1}H\}$ NMR, $CDCl_3$, 101 MHz

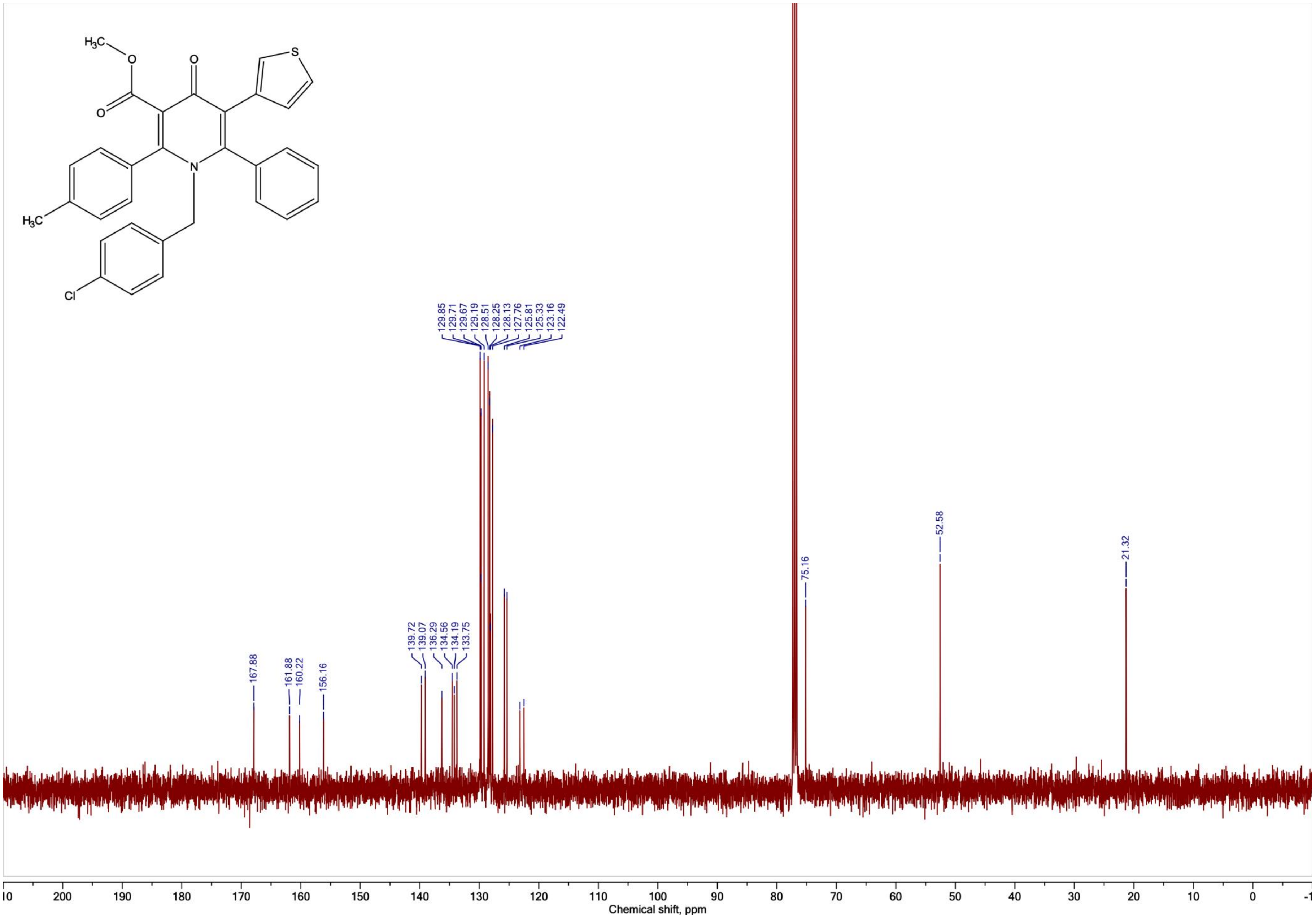

Methyl 1-(4-chlorobenzyl)-4-oxo-6-phenyl-5-(thiophen-3-yl)-2-(*p*-tolyl)-1,4-dihydropyridine-3-carboxylate (17), DEPT, $CDCl_3$, 101 MHz

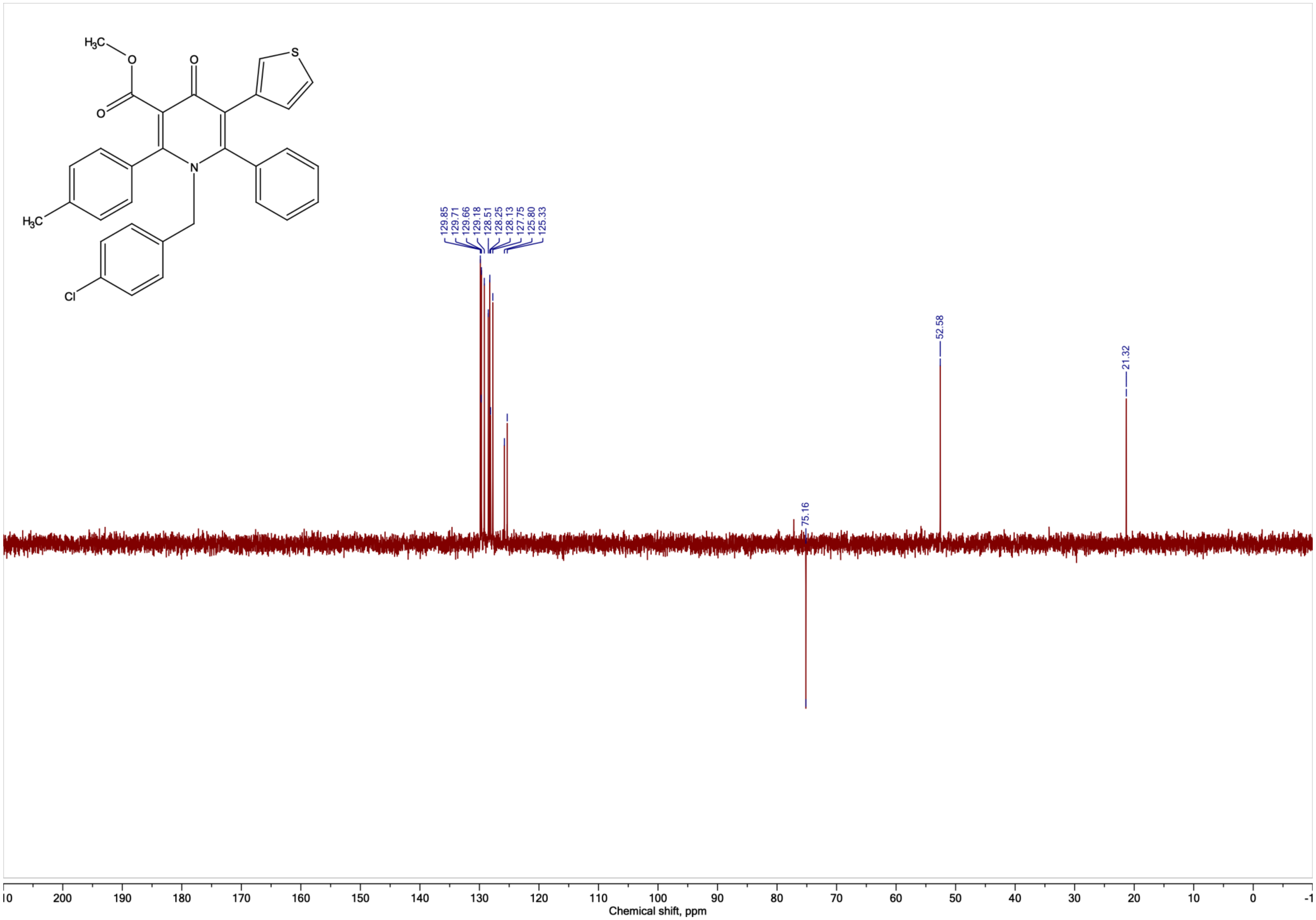

Supplement: File 1 — Experimental procedures, compound characterization data, X-ray diffraction experiment, and copies of NMR spectra of new compounds. [file Beilstein_J_Org_Chem-18-738-s001.pdf]
